# Palladium-catalysed asymmetric annulations of Morita–Baylis–Hillman carbonates with allenes or alkenes *via* migratory insertion

**DOI:** 10.1039/d5sc06910f

**Published:** 2025-10-17

**Authors:** Jin-Yu Huang, Xin-Ting Qin, Han-Wen Rao, Zhi-Chao Chen, Lei Zhu, Qin Ouyang, Wei Du, Ying-Chun Chen

**Affiliations:** a Key Laboratory of Drug-Targeting and Drug Delivery System of the Education Ministry and Sichuan Province and Sichuan Research Center for Drug Precision Industrial Technology, West China School of Pharmacy, Sichuan University Chengdu 610041 China chenzhichao@scu.edu.cn ycchen@scu.edu.cn; b College of Pharmacy, Third Military Medical University Shapingba Chongqing 400038 China ouyangq@tmmu.edu.cn

## Abstract

As one of the most versatile intermediates in organic synthesis, π-allylpalladium complexes have been extensively exploited in allylic alkylation reactions with a wide range of nucleophiles. In contrast, their engagement in the migratory insertion process remains significantly underdeveloped. Here we demonstrate that the π-allylpalladium intermediates derived from Pd^0^ and Morita–Baylis–Hillman (MBH) carbonates of activated ketones can isomerize to the corresponding η^1^-form when stabilised by a pendent carbonyl group, and undertake migratory insertion into various allenes and even styrene-type alkenes efficiently. Subsequent vinylogous deprotonation of the newly formed multifunctional π-allylpalladium species followed by isomerization and intramolecular allylic alkylation leads to skeletally diverse (3 + 2) adducts with high levels of regio- and stereoselectivity. This catalytic strategy not only achieves migratory insertion of non-zwitterionic π-allylpalladium intermediates, but also overcomes the inherent limitations that the MBH carbonates can only undergo annulations with electrophilic dipolarophiles *via* Lewis base catalysis. Mechanistic insights are further elucidated through comprehensive density functional theory calculation studies.

## Abstract 2

The palladium-catalysed asymmetric (3 + 2) annulations of Morita–Baylis–Hillman carbonates with allenes and alkenes are reported *via* a cascade oxidative addition/migratory insertion/vinylogous deprotonation/isomerisation/allyllation sequence.

## Graphical abstract

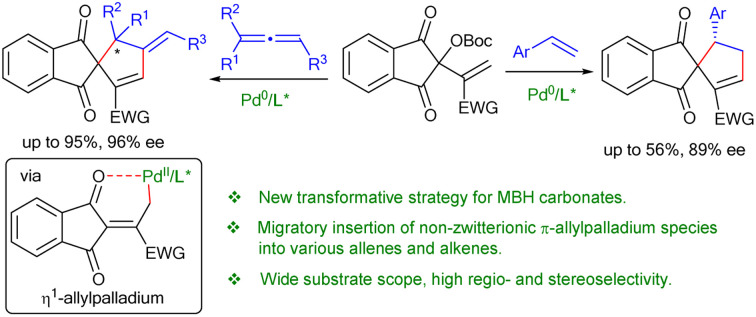

## Introduction

**Scheme 1:**
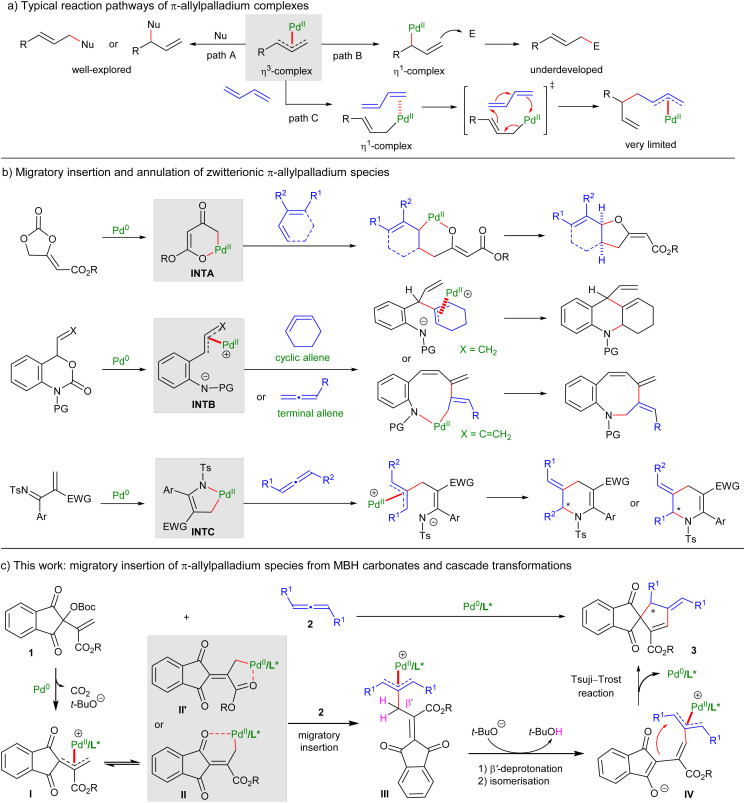
Development of migratory insertion reactions of π-allylpalladium-based species.

Owing to their ready availability and versatile reactivity, π-allylpalladium complexes have emerged as a type of cornerstone synthons in modern organic chemistry.^1^ Such robust intermediates can be readily generated through various pathways, including the oxidative addition of Pd^0^ to allylic derivatives,^2^ as well as Pd-mediated C–H activation of alkenes^3^ or hydropalladation [or Pd^0^-promoted protonation] of polyunsaturated hydrocarbons.^4^ Traditionally, π-allylpalladium species are utilised as electrophiles in the Tsuji–Trost allylic alkylation reaction (Scheme 1a, path A).^5^ Alternatively, π-allylpalladium species occasionally serve as nucleophiles to couple with electrophiles in the presence of suitable reductants (Scheme 1a, path B).^6^

Unlike aryl-Pd^II^ or alkenyl-Pd^II^ intermediates,^7^ allyl-Pd^II^ species were rarely applied in migratory insertion reactions. In fact, in the 1970s, Powell conceptually demonstrated that π-allylpalladium complexes could undertake migratory insertion into 1,3-dienes through transient formation of corresponding η^1^-intermediates (Scheme 1a, path C).^8^ However, synthetic transformations of π-allylpalladium complexes into value-added products *via* migratory insertion have been scarcely explored ever since, probably due to the reluctant isomerization of thermodynamically more stable η^3^-allylpalladium complexes to their η^1^-ones. A major breakthrough was achieved by Trost in 2018, who uncovered that specific oxatrimethylene–methane–palladium species INTA underwent a cascade migratory insertion/allylation reaction with 1,3-dienes to furnish *cis*-fused methylene tetrahydrofurans (Scheme 1b).^9^ Experimental and density functional theory (DFT) studies verified that the preinstalled ester group was instrumental for the desired migratory insertion, which not only stabilized INTA in a η^1^-form, but also effectively lowered the LUMO (lowest unoccupied molecular orbital) energy, rendering them more closely with the HOMO (highest occupied molecular orbital) energy of 1,3-diene partners.^10^ Later, Garg and Ma have accomplished migratory insertion of π-allylpalladium-contained 1,4-*C*,*N*-dipoles INTB into strained cyclic allenes and terminal allenes, respectively.^11^ Recently, we realised migratory insertion of azapalladacycles INTC, *in situ* generated from electron-deficient 1-azadienes and Pd^0^*via* oxidative addition, into racemic internal allenes, and enantioselective and regiodivergent allylation could be obtained to deliver tetrahydropyridine products.^12^ Despite such impressive progress, only limited and specially tailored zwitterionic π-allylpalladium complexes were successfully utilised in migratory insertion reactions. Alternative application of novel functionalised π-allylpalladium species, especially the non-zwitterionic ones, to undergo migratory insertion reactions with diverse unsaturated systems would be highly desirable.

The Morita–Baylis–Hillman (MBH) adducts, condensed from carbonyls and activated alkenes, have been widely used in annulations after conversion to zwitterionic allylic ylide species with organic Lewis bases, but their counterparts were inherently limited to electrophilic alkenes and dipoles.^13^ We envisioned that functionalised MBH carbonates, such as ninhydrin-derived ones 1, could be activated by Pd^0^ to form η^3^-allylpalladium complexes I.^14^ The pendent carbonyl might serve as an additional coordinating site to facilitate the isomerisation of I to the η^1^-form II or II′, which would help lower the LUMO energy and facilitate the migratory insertion into allenes 2 possibly. Notably, the vinylogous β′-protons of the resultant π-allylpalladium complexes III are highly acidic and could be readily deprotonated by the previously generated *t*-butoxide anion.^15^ Subsequent isomerisation and intramolecular Tsuji–Trost reaction would finally furnish (3 + 2) cycloadducts 3, even enantioselectively. This rational design would not only broaden the π-allylpalladium species suitable for migratory insertion reactions, but also introduce a novel catalytic strategy for the transformations of multifunctional MBH carbonates, allowing for their assemblies with electron-neutral unsaturated systems, which are not feasible *via* conventional Lewis base catalysis.

## Results and discussion

### Reaction optimisation

We initiated the investigation by examining the reaction between MBH carbonate 1a and racemic internal allene 2a in DCM at 40 °C under the catalysis of Pd(PPh_3_)_4_. To our delight, apparent conversions were observed to give the desired (3 + 2) annulation product 3a, albeit in a low yield, as side reactions promoted by Lewis basic PPh_3_ were also noted (Table 1, entry 1). Consequently, a series of chiral ligands in combination with Pd(OAc)_2_ were examined for asymmetric induction. After screenings,^16^ it was found that using pyridinyl-oxazoline L1 exhibited fair catalytic efficiency and moderate enantioselectivity (entry 2). Introducing either electron-donating or withdrawing substituents into the pyridine skeleton (L2–L4) improved the yield significantly, but the enantioselectivity was unsatisfactory (entries 3–5). Chiral bisoxazolines L5–L8 were applicable (entries 6–9), and a high ee value was obtained with L7 having bulky *tert*-butyl groups (entry 8). Unfortunately, poor catalytic efficacy was observed with ligand L9 (entry 10). Other solvents were tested but delivered diminished yields (entries 11–14). While slightly higher enantioselectivity was achieved by employing MBH carbonate 1b from ethyl acrylate (entry 15), a quick survey of palladium sources revealed that Pd(TFA)_2_ was a better choice (entries 16–18). Lowering the temperature resulted in a significantly reduced yield because of incomplete conversions (entry 19). A control experiment verified the necessity of Et_3_N (entry 20), which might act as a reductant to ensure the generation of reactive Pd^0^ from the Pd^II^ precatalyst.^17^ It should be noted that a high yield was retained by employing 1.0 equivalent of allene 2a (entry 21).

**Table 1:** Condition optimisations of the asymmetric (3 + 2) annulations of MBH carbonates 1 and racemic allene 2a^a^

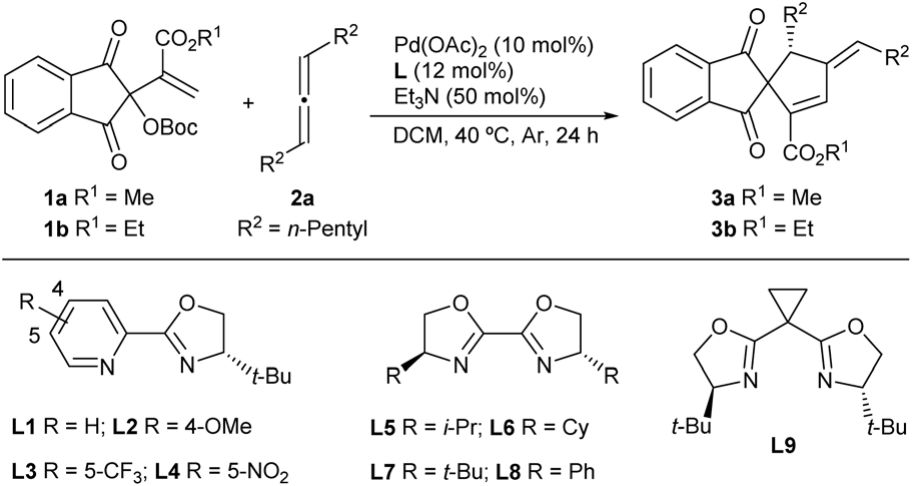					
Entry	[Pd]	L	Solvent	Yield^b^ (%)	ee^c^ (%)
1^d^	Pd(PPh_3_)_4_	—	DCM	3a, 18	—
2	Pd(OAc)_2_	L1	DCM	3a, 30	66
3	Pd(OAc)_2_	L2	DCM	3a, 70	61
4	Pd(OAc)_2_	L3	DCM	3a, 80	70
5	Pd(OAc)_2_	L4	DCM	3a, 84	73
6	Pd(OAc)_2_	L5	DCM	3a, 89	81
7	Pd(OAc)_2_	L6	DCM	3a, 86	82
8	Pd(OAc)_2_	L7	DCM	3a, 76	87
9	Pd(OAc)_2_	L8	DCM	3a, 81	33
10	Pd(OAc)_2_	L9	DCM	3a, 35	5
11	Pd(OAc)_2_	L7	DCE	3a, 63	85
12	Pd(OAc)_2_	L7	MeOH	3a, 34	89
13	Pd(OAc)_2_	L7	Toluene	3a, 17	89
14	Pd(OAc)_2_	L7	THF	3a, 74	88
15^e^	Pd(OAc)_2_	L7	DCM	3b, 81	90
16^e^	Pd(TFA)_2_	L7	DCM	3b, 93	92
17^e^	Pd_2_(dba)_3_	L7	DCM	3b, 70	81
18^e^	Pd(allyl)cp	L7	DCM	3b, 15	61
19^e^^,^^f^	Pd(TFA)_2_	L7	DCM	3b, 68	93
20^d^^,^^e^	Pd(TFA)_2_	L7	DCM	NR	—
21^e^^,^^g^	Pd(TFA)_2_	L7	DCM	3b, 84	91

a Unless noted otherwise, the reaction was performed with MBH carbonate 1a (0.1 mmol), racemic allene 2a (0.2 mmol), [Pd] (10 mol%), L (12 mol%) and Et_3_N (0.05 mmol) in DCM (1.0 mL) at 40 °C for 24 h under Ar.

b Yield of the isolated product.

c Determined by HPLC analysis on a chiral stationary phase.

d Without Et_3_N.

e 1b (0.1 mmol) was used.

f At rt.

g With 2a (0.1 mmol).

### Substrate scope investigation

**Scheme 2:**
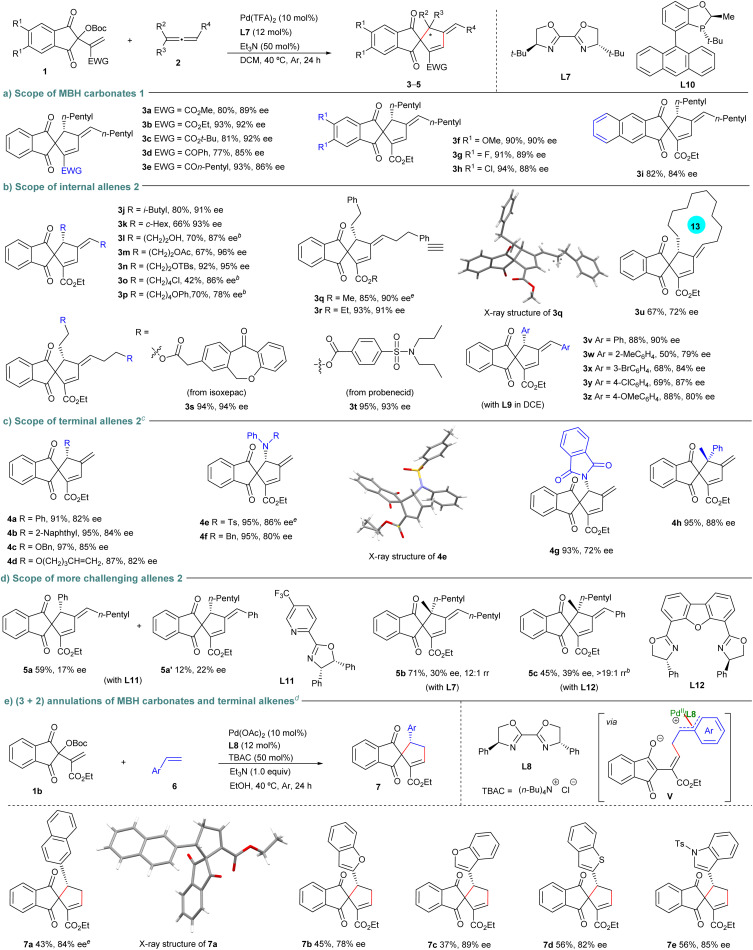
Asymmetric (3 + 2) annulations of MBH carbonates and diverse allenes^*a*^. ^*a*^On a 0.1 mmol scale. ^*b*^For 48 h. ^*c*^With L10 (12 mol%) at rt. ^*d*^With alkene 6 (0.5 mmol), Pd(OAc)_2_ (10 mol%), L8 (12 mol%), TBAC (50 mol%) and Et_3_N (1.0 equiv) in EtOH (1.0 mL).^*e*^The absolute configuration of enantiopure 3q, 4e and 7a were determined by X-ray analysis. Other products were assigned by analogy.

Consequently, we first investigated the scope of MBH carbonates 1 in the reactions with racemic allene 2a under the catalysis of Pd(TFA)_2_/L7. As summarised in Scheme 2a, comparable good yields and enantioselectivity were obtained for products 3a–c with diverse ester groups, whereas slightly diminished enantiocontrol was observed for products 3d and 3e from vinyl ketone-derived carbonates 1. In addition, high yields and ee values were attained for the MBH carbonates 1 bearing either electron-rich or -deficient groups on the phenyl ring (products 3f–h), even for the benzo[*f*]ninhydrin-derived one (product 3i). Next, a variety of racemic symmetric allenes 2 were explored. As illustrated in Scheme 2b, a wide range of 1,3-dialkyl substituted allenes 2, even those with various functionalities, all underwent the (3 + 2) annulations with MBH carbonate 1b smoothly, producing 3j–r in moderate to good yields with uniformly high enantioselectivity. Some allenes with drug motifs were also compatible (product 3s and 3t). In addition, cyclic allene worked well to yield product 3u with moderate enantioselectivity. Notably, 1,3-diaryl-substituted allenes 2 were also applicable under the catalysis of Pd(TFA)_2_/L9, giving corresponding product 3v–z in moderate to high yields and enantioselectivity.

Encouraged by these results, we next turned our attention to explore the (3 + 2) annulations of the MBH carbonate 1b with more challenging unsymmetrical allenes, as simultaneous control over enantio- and regioselectivity in the allylation step would be encountered. Delightfully, 1-aryl-, 1-alkoxyl- and 1-amino-substituted terminal allenes exhibited high reactivity in the assemblies with MBH carbonate 1b under the catalysis of Pd(TFA)_2_/L10, and products 4a–g were generally furnished in outstanding yields and regioselectivity, whereas moderate to good ee values were obtained. Even a 1,1-disubstituted allene was compatible, affording product 4h featuring a quaternary stereocenter with good data (Scheme 2c). Moderate regioselectivity could be achieved with 1-aryl-3-alkyl-allene, but both products 5a and 5a′ were obtained with poor enantioselectivity under the catalysis of Pd(TFA)_2_/L11. Notably, even trisubstituted allenes proved to be reliable partners, preferentially affording products 5b and 5c having a quaternary stereocenter with high regioselectivity, though the enantioselectivity was currently unsatisfactory (Scheme 2d).

Migratory insertion of π-allylpalladium complexes into simple alkenes represents a more challenging task. Although strained norbornenes can engage in such a process,^18^ attempts to perform migratory insertion of zwitterionic π-allylpalladium species with alkenes all proved unsuccessful due to the high energy barriers involved.^10^ Notably, the assembly of MBH carbonate 1b and 2-vinylnaphthalene 6a was successful under the catalysis of Pd(OAc)_2_/L8, with tetrabutylammonium chloride (TBAC) as an additive,^19^ furnishing (3 + 2) product 7a with good enantioselectivity, albeit in a fair yield, probably by forming dearomative intermediate V.^20^ Importantly, other benzo-fused heteroarenes were compatible (products 7b–e), as summarised in Scheme 2e. Collectively, these reactions well demonstrated the broad applicability of the current approach with respect to diversely substituted allenes as well as terminal alkenes.

**Scheme 3:**
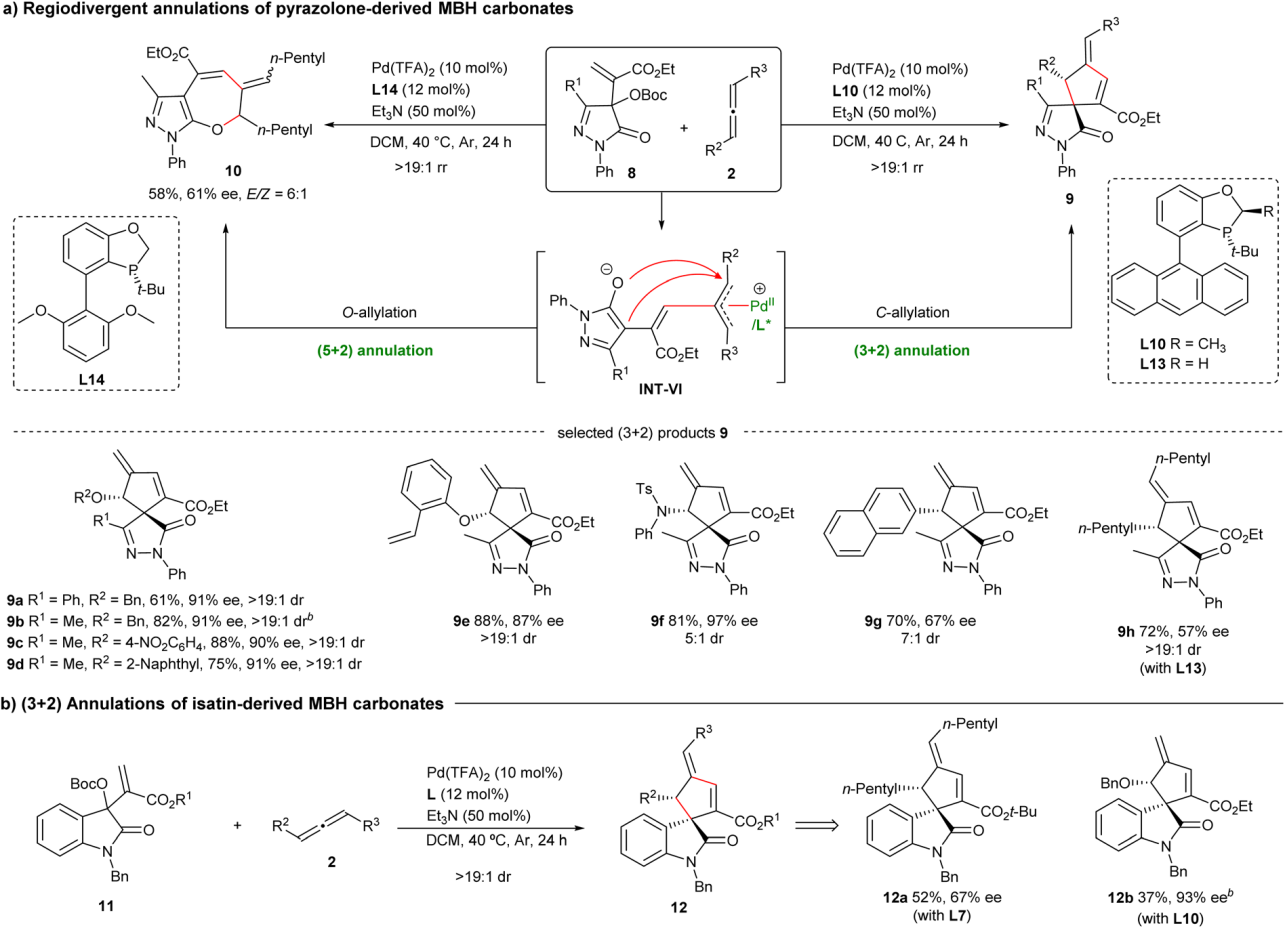
Exploration of more types of MBH carbonates^*a*^. ^*a*^On a 0.1 mmol scale. ^*b*^The absolute configurations of enantiopure 9b was determined by X-ray analysis after conversion to 18. The relative configuration of 12b was determined by X-ray analysis after derivatisation, and its absolute configuration was assigned by ECD analysis (see the SI). Other products were assigned by analogy.

To further exhibit the generality of this protocol and construct skeletally diverse products, the MBH carbonates derived from other activated ketones were investigated. As depicted in Scheme 3a, pyrazolone-based MBH carbonates 8 underwent similar (3 + 2) annulations with both terminal and internal allenes efficiently in the presence of Pd(TFA)_2_/L10 or L13, respectively, affording products 9a–h bearing two adjacent stereocenters with moderate to good enantioselectivity. Notably, a different regioselective *O*-allylation of intermediate VI occurred to deliver (5 + 2) product 10 by using Pd(TFA)_2_/L14, albeit in moderate yield and stereoselectivity. In addition, isatin-based MBH carbonates 11 were also suitable for (3 + 2) annulations with allenes, and products 12a and 12b were afforded with moderate data under the catalysis of Pd(TFA)_2_ with L7 or L10, respectively (Scheme 3b). Regrettably, some aldehyde-derived MBH carbonates and tetrasubstituted allenes were not suitable for current transformation.^16^

### Synthetic transformations

**Scheme 4:**
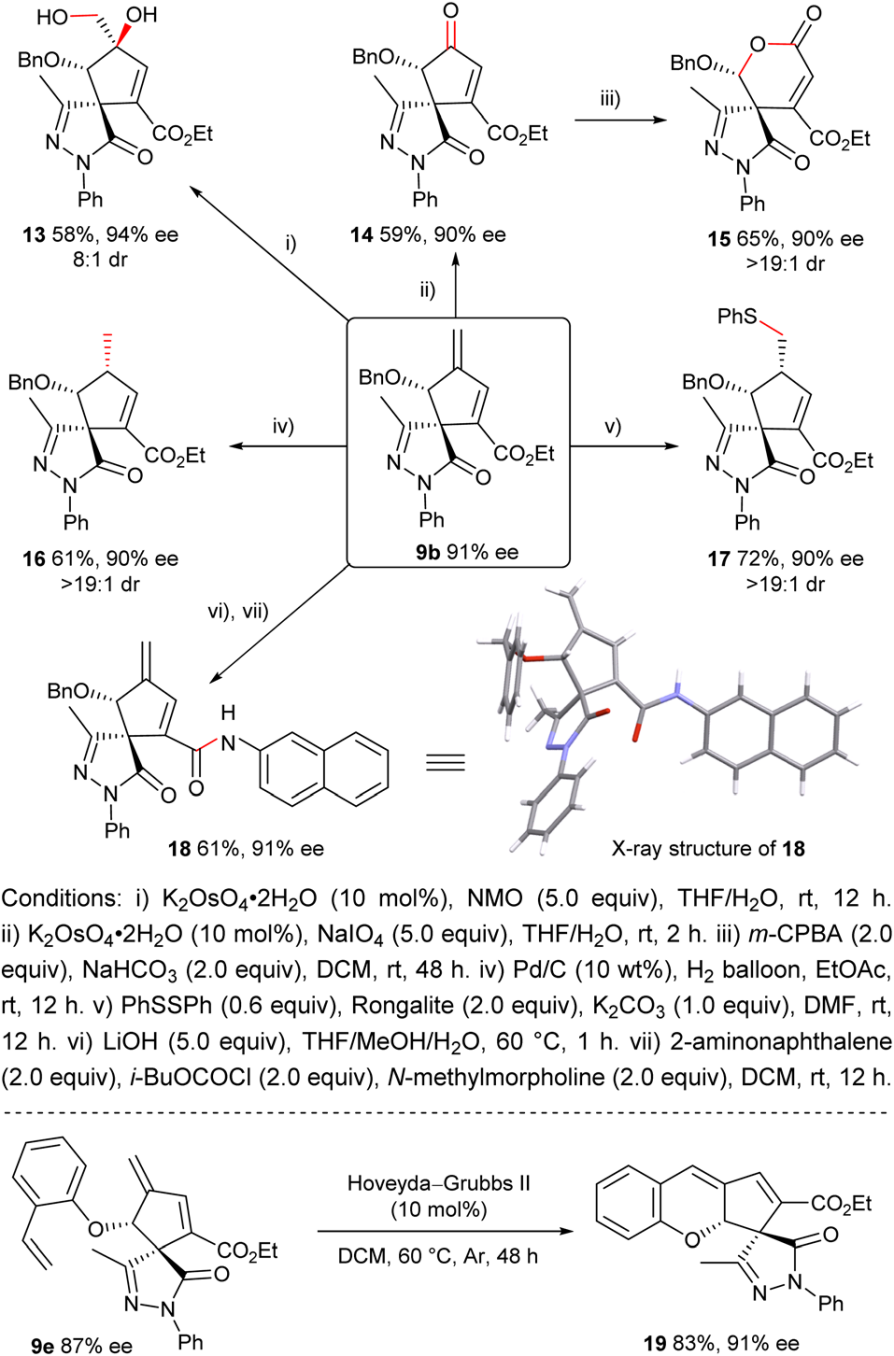
Synthetic transformations of products.

The obtained multifunctional (3 + 2) annulation adducts demonstrate versatile synthetic utility, serving as a valuable platform for accessing structurally diverse architectures. As illustrated in Scheme 4, treating product 9b with K_2_OsO_4_·2H_2_O/NMO provided diol 13 with high diastereoselectivity. Interestingly, chemoselective oxidative cleavage of terminal olefin of 9b with K_2_OsO_4_·2H_2_O/NaIO_4_ furnished ketone 14, which could be further converted to chiral lactone 15*via* Baeyer–Villiger rearrangement. Besides, the *exo*-cyclic double bond of 9b also underwent selective hydrogenation to yield 16, or sulfur-Michael addition to give 17,^21^ both with exclusive diastereoselectivity. Additionally, the ethyl ester group of 9b was amenable to hydrolysis followed by condensation with 2-aminonaphthalene to generate amide 18 with retained enantioselectivity. Moreover, a ring-closing metathesis reaction of compound 9e delivered polycyclic product 19.

### Mechanism studies

**Scheme 5:**
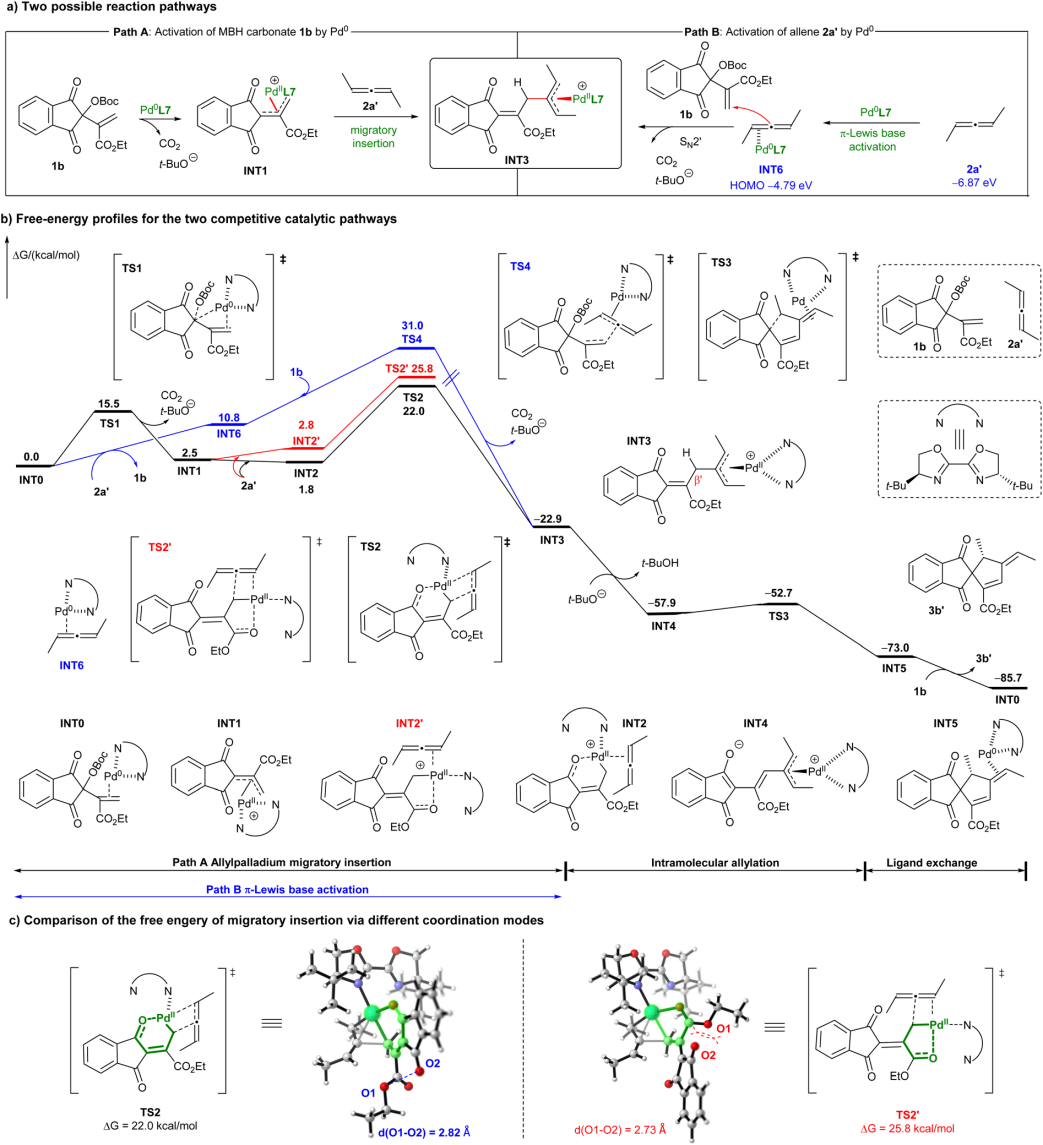
Mechanism studies on different activation pathways.

As proposed in Scheme 5a, path A, the key π-allylpalladium complex INT3 may be generated from the oxidative addition of Pd^0^ to MBH carbonate 1b, followed by migratory insertion into simplified allene 2a′.^11^ Alternatively, allene 2a′ might also be activated by Pd^0^*via* η^2^-coordination and backdonation based on a Dewar–Chatt–Duncanson model,^12,22^ which is indeed supported by DFT calculations. It is found that the HOMO energy of Pd^0^-η^2^-complex INT6 (−4.79 eV) is significantly enhanced compared to that of allene 2a′ (−6.87 eV). The nucleophilicity enhanced INT6 may attack MBH carbonate 1b to form π-allylpalladium complex INT3 in a S_N_2′ fashion (path B).

To figure out which reaction pathway is more favourable, comprehensive DFT calculations were conducted. As shown in Scheme 5b (black line), the energy barrier of oxidative addition of Pd^0^ to MBH carbonate 1b*via*TS1 is 15.5 kcal mol^−1^. Notably, the pendent carbonyl group of 1,3-indandione serves as an additional binding group to facilitate the isomerisation of η^3^-allylpalladium complex INT1 to slightly more stable η^1^-one INT2. Subsequent migratory insertion of INT2 into allene 2a′*via*TS2, with a free energy barrier of 22.0 kcal mol^−1^, constitutes the rate-determining step and is feasible under current reaction conditions. Although the ester moiety can also serve as a binding group to form INT2′, the energy barrier for subsequent migratory insertion into allene 2a′*via*TS2′ is apparently higher than that of TS2 (25.8 *vs.* 22.0 kcal mol^−1^, red line), because of apparent steric hindrance between the ester group and the 1,3-indandione skeleton in TS2′, as noted in Scheme 5c. In contrast, the attack of HOMO-raised Pd^0^-η^2^-complex INT6 on MBH carbonate 1b*via*TS4 features a significantly higher energy barrier of 31.0 kcal mol^−1^ (blue line), indicating it is dynamically infeasible at current reaction temperature. Consequently, the proposed oxidative addition/migratory insertion process is more favourable for the initial assembly of two partners. Moreover, as conjugated polyunsaturated systems are typically required for π-Lewis base catalysis,^22*b*–*d*^ the successful engagement of styrene-type alkenes 6 in current (3 + 2) annulations (Scheme 2e) provides additional and solid evidence in supporting an oxidative addition/migratory insertion mechanism.

The strong electron-withdrawing effect of both 1,3-indandione and the π-allylpalladium complex renders the β′-H of INT3 highly acidic, which can be easily deprotonated by the *in situ* generated *t*-butoxide anion to deliver the more stable intermediate INT4 with a significant exotherm of 35.0 kcal mol^−1^. In addition, an outer-sphere allylic alkylation *via*TS3, with an energy barrier of only 5.2 kcal mol^−1^, can smoothly occur to provide product 3b′ after ligand exchange.^16^

**Scheme 6:**
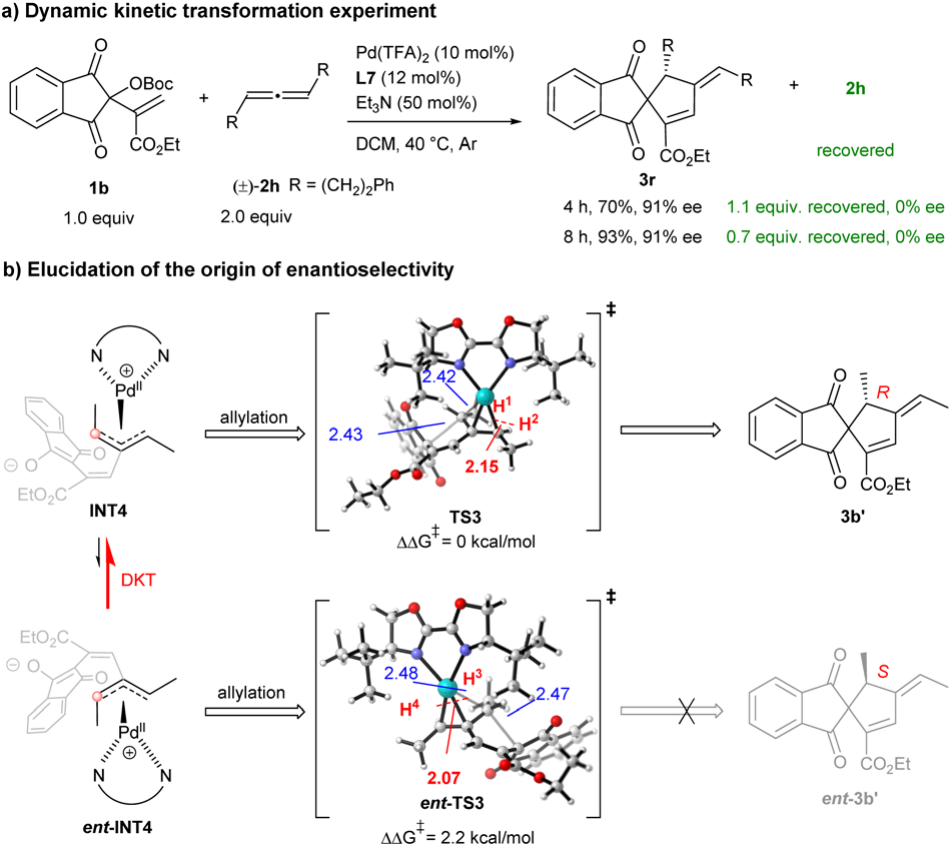
Elucidation of the enantioselectivity.

As outlined in Scheme 6a, racemic 2h was recovered when 2.0 equivalents of 2h were used, indicating that kinetic resolution of racemic allene 2h is not involved in this transformation. Instead, the dynamic kinetic transformation (DKT) of the π-allylpalladium species IV appears to be operative.^12^ Consequently, further calculations were conducted to elucidate the origin of enantioselectivity. Both INT4 and *ent*-INT4 would be generated *via* migratory insertion of INT2 into racemic 2a′ followed by deprotonation. As depicted in Scheme 6b, a comparative geometry analysis reveals that the distance between H^1^ and H^2^ in TS3 (2.15 Å) is longer than that between H^3^ and H^4^ in *ent*-TS3 (2.07 Å), indicating a greater 1,3-strain between the adjacent H atom and CH_3_ moiety in *ent*-TS3. This steric repulsion leads to the free energy of *ent*-TS3 being 2.2 kcal mol^−1^ higher than that of TS3, suggesting that *ent*-INT4 would undergo dynamic kinetic transformation (DKT) into INT4*via* π–σ–π isomerisation, thus producing (*R*)-3b′ predominantly.^23^ This theoretical prediction is in agreement with the experimental observations. The above DFT calculations identify migratory insertion as the rate-determining step, and intramolecular allylation as the enantioselectivity-determining step.

## Conclusions

In summary, with the assistance of experimental results and density functional theory calculations, we demonstrated that the functionalised π-allylpalladium complexes, generated by oxidative addition of Pd^0^ to the Morita–Baylis–Hillman carbonates from activated ketones, could readily isomerise to their η^1^-form *via* ligation with the pendent carbonyl group, which enabled migratory insertion into diverse allenes and styrene-type alkenes. Subsequent vinylogous deprotonation and intramolecular allylic alkylation furnished (3 + 2) annulation products. This protocol featured substantial substrate scope and good functional group compatibility, delivering a diversity of spirocyclic frameworks with moderate to excellent regio-, chemo-, and stereoselectivity. As a result, a novel transformative paradigm for multifunctional MBH carbonates has been established *via* migratory insertion of the *in situ* formed non-zwitterionic π-allylpalladium species, rendering their unprecedented annulations with electron-neutral unsaturated systems—previously inaccessible under Lewis base catalysis. Further expansion studies of these Morita–Baylis–Hillman carbonates with other types of unsaturated systems are under investigation, and the results will be reported in due course.

## Author contributions

The manuscript was written through contributions of all authors. All authors have given approval to the final version of the manuscript.

## Conflicts of interest

There are no conflicts to declare.

## Supplementary Material

SC-OLF-D5SC06910F-s001

SC-OLF-D5SC06910F-s002

## Data Availability

CCDC 2473163–2473167 (3q, 4e, 7a, 18 and the derivative of racemic 12b) contain the supplementary crystallographic data for this paper.^24*a*–*e*^ The data that support the findings of this study are available in the supplementary information (SI) or on request from the corresponding author. Supplementary information: experimental procedures, spectroscopic data for new compounds, NMR, HRMS spectra and HPLC chromatograms, and CIF files of enantiopure products 3q, 4e, 7a, 18 and the derivative of racemic 12b. See DOI: https://doi.org/10.1039/d5sc06910f.
