# Supplementary material for: Palladium-catalysed asymmetric annulations of Morita–Baylis–Hillman carbonates with allenes or alkenes via migratory insertion
Source: Chem Sci. 2025 Oct 17;16(47):22456–64. doi: 10.1039/d5sc06910f (PMC12550766; doi:10.1039/d5sc06910f)
Supplement: SC-016-D5SC06910F-s001 [file SC-016-D5SC06910F-s001.pdf]

Supplementary Information (SI) for Chemical Science.
This journal is © The Royal Society of Chemistry 2025

Palladium-catalysed asymmetric annulations of Morita–Baylis–Hillman carbonates with allenes or alkenes via migratory insertion

Jin-Yu Huang,[a,‡] Xin-Ting Qin,[a,‡] Han-Wen Rao,[a] Zhi-Chao Chen,[a*] Lei Zhu,[b] Qin Ouyang,[b*] Wei Du,[a] and Ying-Chun Chen[a*]

[a] Key Laboratory of Drug-Targeting and Drug Delivery System of the Education Ministry and Sichuan Province, and Sichuan Research Center for Drug Precision Industrial Technology, West China School of Pharmacy, Sichuan University, Chengdu 610041, China.

[b] College of Pharmacy, Third Military Medical University, Shapingba, Chongqing 400038, China.

‡J.-Y. H. and X.-T. Q equally contributed to this work.

Email: chenzhichao@scu.edu.cn; ouyangq@tmmu.edu.cn; ycchen@scu.edu.cn

Supplementary Information

1. General methods S1

2. Preparation and characterisation of MBH carbonates S2

3. Detailed screening conditions S5

4. General procedure for (3+2) annulations of MBH carbonates with diverse allenes or alkenes S9

5. Synthetic transformations S45

6. More substrate exploration S50

7. Crystal, ECD spectra data and structural refinements S50

8. Mechanism investigations S59

9. References S64

10. NMR, HRMS spectra and HPLC chromatograms S66

11. DFT computational calculation data S271

1. General methods

Unless otherwise noted, all reactions were carried out under ambient atmosphere; when the reactions required heating, the heat source was oil bath. ^{1}H NMR (400 or 600 MHz), ^{13}C NMR (100 or 150 MHz) and ^{19}F NMR (376 MHz) spectra were recorded on Varian INOVA-400/54, Agilent DD2-600/54 or Bruker AscendTM 400 instruments (Chemical shifts were reported in ppm from tetramethylsilane with the solvent resonance as the internal standard in $CDCl_3$ solution, unless otherwise noted). The following abbreviations were used to explain the multiplicities: s = singlet, d = doublet, t = triplet, q = quartet, dd = double doublet, dt = double triplet, td = triple doublet, m = multiplet and coupling constants (*J*) are reported in Hertz (Hz). ESI-HRMS was recorded on a Waters SYNAPT G2 or Agilent G1969-85000 using a time-of-flight mass spectrometer equipped with electrospray ionization (ESI) source. X-ray diffraction experiments were carried out on Bruker APEX-II CCD diffractometer, and the data obtained (CCDC 2473163–2473167) were deposited at the Cambridge Crystallographic Data Centre. In each case, diastereoselective, regioselective and *E/Z* ratios were determined by ^{1}H NMR analysis and enantiomeric ratio was determined by HPLC (Agilent Technologies: 1220 Infinity II, 1200 Series, 1260 Infinity) analysis on a chiral stationary phase in comparison with an authentic racemate, using a Daicel Chiralpak AD-H Column (250 × 4.6 mm), Daicel Chiralpak AS-H Column (250 × 4.6 mm), Chiralpak IA Column (250 × 4.6 mm), Chiralpak IB Column (250 × 4.6 mm), Chiralpak IC Column (250 × 4.6 mm), Chiralpak ID Column (250 × 4.6 mm), Chiralpak OD-H Column (250 × 4.6 mm)or Chiralpak OD-3 Column (250 × 4.6 mm). UV detection was monitored at 254 nm. The specific optical rotation was obtained from Rudolph Research Analytical Autopol I automatic polarimeter in $CHCl_3$ (or MeOH, MeCN) solution at 25 °C. The melting point was obtained from WRX-4 Mel-Temp apparatus. Column chromatography was performed on silica gel (300–400 mesh) eluting with ethyl acetate (EtOAc) and petroleum ether. TLC was performed on glass-backed silica plates. UV light, I_2, and solution of potassium permanganate were used to visualize products or starting materials. All chemicals were used without purification as commercially available unless otherwise noted. Petroleum ether (60–90 °C) was redistilled.

2. Preparation and characterisation of MBH carbonates

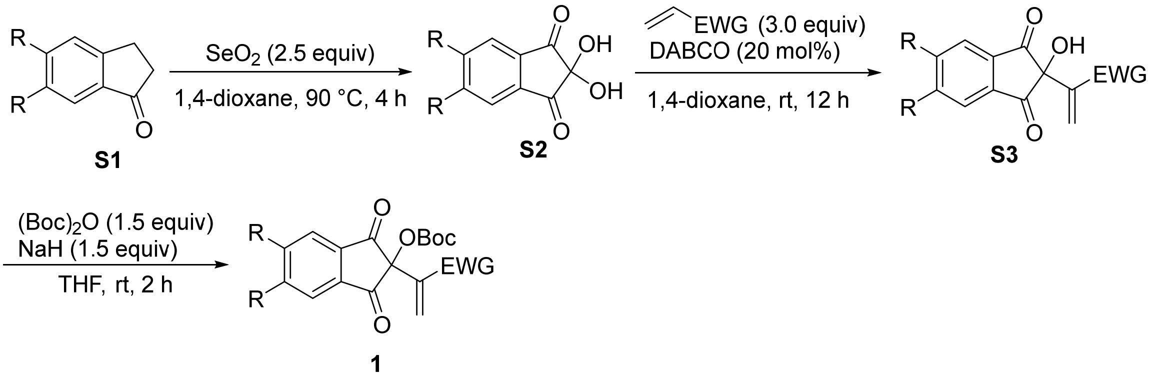

To a soulution of **S1** (20 mmol, 1.0 equiv) in 1,4-dioxane (100 mL) was added SeO_2 (50 mmol, 2.5 equiv). The mixture was stirred at 90 °C for 4 h. After completion, the mixture was cooled to room temperature and filtered through Celite. The filtrate was concentrated and purified by column chromatography on silica gel to afford **S2**.

To a soulution of **S2** (10 mmol, 1.0 equiv) in 1,4-dioxane (10 mL) was added DABCO (2 mmol, 20 mol%) and electron-deficient alkene (30 mmol, 3.0 equiv). The mixture was stirred at rt for 12 h. After completion, the mixture was concentrated and purified by column chromatography on silica gel to afford **S3**.

To a solution of **S3** (10 mmol, 1.0 equiv) in THF (20 mL) was added NaH (15 mmol, 1.5 equiv) portionwise at 0 °C. The mixture was stirred for 15 minutes, after which $(Boc)_2O$ (15 mmol, 1.5 equiv) was added dropwise. The reaction mixture was then stirred at rt for 2 h. After completion, the mixture was quenched with saturated NH_4Cl solution at 0 °C and extracted with EtOAc. The organic layers were dried over Na_2SO_4, filtered and concentrated under reduced pressure. The residue was purified by column chromatography on silica gel to give MBH carbonate **1a**–**1i**.

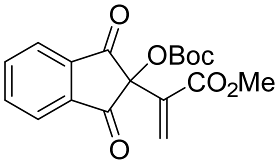

1a: as a white solid; mp 143–145 °C; ^{1}H NMR (400 MHz, $CDCl_3$): δ (ppm) 8.07–7.95 (m, 2H), 7.92–7.81 (m, 2H), 6.69 (s, 1H), 6.63 (s, 1H), 3.53 (s, 3H), 1.36 (s, 9H); ^{13}C NMR (100 MHz, $CDCl_3$): δ (ppm) 194.7, 164.3, 151.2, 141.5, 135.5, 133.7, 132.2, 123.1, 84.6, 80.1, 52.3, 27.4; HRMS (ESI-TOF) *m/z*: $[M + Na]^+$ Calcd for $C_{18}H_{18}O_7Na^+$ 369.0945; Found 369.0948.

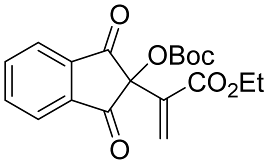

1b: as a white solid; mp 116–118 °C; ^{1}H NMR (400 MHz, $CDCl_3$): δ (ppm) 8.08–7.93 (m, 2H), 7.93–7.78 (m, 2H), 6.70 (s, 1H), 6.61 (s, 1H), 3.96 (q, J = 7.1 Hz, 2H), 1.36 (s, 9H), 1.05 (t, J = 7.1 Hz, 3H); ^{13}C NMR (100 MHz,

$CDCl_3$): δ (ppm) 194.7, 163.8, 151.2, 141.6, 135.5, 133.9, 132.0, 123.2, 84.6, 80.1, 61.5, 27.4, 13.6; HRMS (ESI-TOF) *m/z*: $[M + Na]^+$ Calcd for $C_{19}H_{20}O_7Na^+$ 383.1101; Found 383.1106

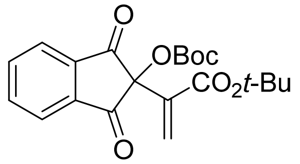

1c: as a white solid; mp 134–136 °C; ^{1}H NMR (400 MHz, $CDCl_3$): δ (ppm) 8.06–7.93 (m, 2H), 7.89–7.76 (m, 2H), 6.56 (s, 1H), 6.48 (s, 1H), 1.35 (s, 9H), 1.21 (s, 9H); ^{13}C NMR (100 MHz, $CDCl_3$): δ (ppm) 194.7, 163.0, 151.3, 141.6, 135.43, 135.38, 130.9, 123.3, 84.4, 82.6, 80.1, 27.6, 27.5; HRMS (ESI-TOF) *m/z*: $[M + Na]^+$ Calcd for $C_{21}H_{24}O_7Na^+$ 411.1414; Found 411.1419.

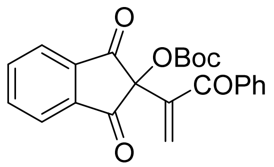

1d: as a white solid; mp 132–134 °C; ^{1}H NMR (400 MHz, $CDCl_3$): δ (ppm) 8.06–7.96 (m, 2H), 7.89–7.81 (m, 2H), 7.63 (d, J = 7.4 Hz, 2H), 7.53 (t, J = 7.4 Hz, 1H), 7.39 (t, J = 7.6 Hz, 2H), 6.83 (s, 1H), 6.11 (s, 1H), 1.39 (s, 9H); ^{13}C NMR (100 MHz, $CDCl_3$): δ (ppm) 194.62, 194.57, 151.4, 141.45, 141.42, 136.3, 135.6, 133.2, 131.8, 129.8, 128.4, 123.3, 84.7, 80.9, 27.5; HRMS (ESI-TOF) *m/z*: $[M + Na]^+$ Calcd for $C_{23}H_{20}O_6Na^+$ 415.1152; Found 415.1152.

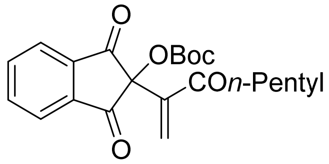

1e: as a white solid; mp 62–64 °C; ^{1}H NMR (400 MHz, $CDCl_3$): δ (ppm) 8.03–7.94 (m, 2H), 7.88–7.80 (m, 2H), 6.78 (s, 1H), 6.53 (s, 1H), 2.59 (t, J = 7.5 Hz, 2H), 1.47–1.38 (m, 2H), 1.35 (s, 9H), 1.25–1.11 (m, 4H), 0.82 (t, J = 7.1 Hz, 3H); ^{13}C NMR (100 MHz, $CDCl_3$): δ (ppm) 199.7, 194.8, 151.2, 143.1, 141.4, 135.3, 131.2, 123.1, 84.5, 80.2, 37.8, 31.1, 27.4, 23.7, 22.3, 13.8; HRMS (ESI-TOF) *m/z*: $[M + Na]^+$ Calcd for $C_{22}H_{26}O_6Na^+$ 409.1622; Found 409.1621.

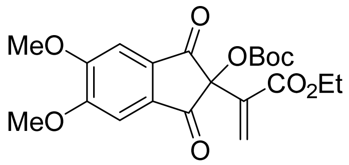

1f: as a white solid; mp 180–182 °C; ^{1}H NMR (400 MHz, $CDCl_3$): δ (ppm) 7.39 (s, 2H), 6.69 (s, 1H), 6.61 (s, 1H), 4.04 (s, 6H), 4.00 (q, J = 7.2 Hz, 2H), 1.38 (s, 9H), 1.13 (t, J = 7.2 Hz, 3H); ^{13}C NMR (100 MHz, $CDCl_3$): δ (ppm) 193.5, 164.0, 155.8, 151.2, 136.8, 134.0, 131.9, 103.7, 84.3, 80.3, 61.4, 56.6, 27.5, 13.8; HRMS (ESI-TOF) *m/z*: $[M + Na]^+$ Calcd for $C_{21}H_{24}O_9Na^+$ 443.1313; Found 443.1312.

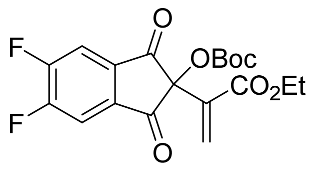

1g: as a white solid; mp 129–131 °C; ^{1}H NMR (400 MHz, $CDCl_3$): δ (ppm) 7.76 (t, J = 7.4 Hz, 2H), 6.71 (s, 1H), 6.62 (s, 1H), 4.00 (q, J = 7.1 Hz, 2H), 1.37 (s, 9H), 1.15 (t, J = 7.1 Hz, 3H); ^{13}C NMR (100 MHz, $CDCl_3$): δ (ppm) 192.6, 164.0, 155.42 (dd, J = 264.6, 15.6 Hz), 151.4, 138.8 (t, J = 5.0 Hz),

133.6, 132.3, 112.1 (dd, J = 13.7, 7.1 Hz), 85.0, 79.4, 61.7, 27.4, 13.8; ^{19}F NMR (376 MHz, $CDCl_3$): δ (ppm) –122.42; HRMS (ESI-TOF) *m/z*: $[M + Na]^+$ Calcd for $C_{19}H_{18}F_2O_7Na^+$ 419.0913; Found 419.0915.

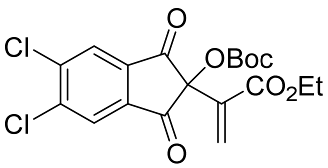

1h: as a white solid; mp 156–158 °C; ^{1}H NMR (400 MHz, $CDCl_3$): δ (ppm) 8.06 (s, 2H), 6.71 (s, 1H), 6.61 (s, 1H), 4.01 (q, J = 7.1 Hz, 2H), 1.37 (s, 9H), 1.15 (t, J = 7.1 Hz, 3H); ^{13}C NMR (100 MHz, $CDCl_3$): δ (ppm) 192.8, 164.1, 151.5, 140.5, 140.3, 133.8, 132.1, 125.0, 85.1, 79.7, 61.8, 27.5, 13.8; HRMS (ESI-TOF) *m/z*: $[M + Na]^+$ Calcd for $C_{19}H_{18}{}^{35}Cl_2O_7Na^+$ 451.0322; Found 451.0324; Calcd for $C_{19}H_{18}{}^{35}Cl{}^{37}ClO_7Na^+$ 453.0292; Found 453.0299.

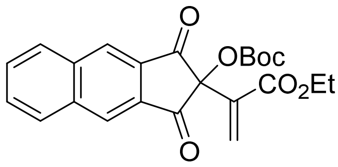

1i: as a white solid; mp 173–175 °C; ^{1}H NMR (400 MHz, $CDCl_3$): δ (ppm) 8.53 (s, 2H), 8.11 (dd, J = 6.3, 3.3 Hz, 2H), 7.70 (dd, J = 6.3, 3.2 Hz, 2H), 6.72 (s, 1H), 6.65 (s, 1H), 3.93 (q, J = 7.1 Hz, 2H), 1.35 (s, 9H), 1.02 (t, J = 7.1 Hz, 3H); ^{13}C NMR (100 MHz, $CDCl_3$): δ (ppm) 195.1, 164.0, 151.4, 136.7, 136.3, 134.5, 131.6, 130.7, 129.4, 124.2, 84.6, 80.6, 61.5, 27.4, 13.7; HRMS (ESI-TOF) *m/z*: $[M + Na]^+$ Calcd for $C_{23}H_{22}O_7Na^+$ 433.1258; Found 433.1259.

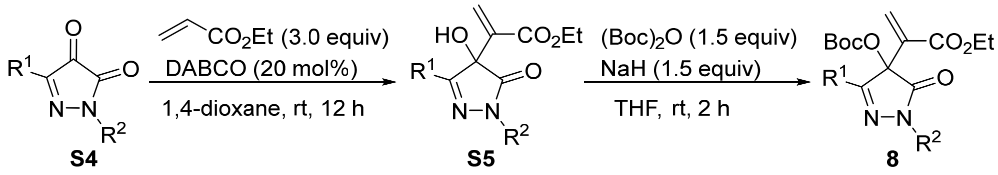

To a soulution of **S4** (10 mmol, 1.0 equiv) in 1,4-dioxane (10 mL) was added DABCO (2 mmol, 20 mol%) and ethyl acrylate (30 mmol, 3.0 equiv). The mixture was stirred at rt for 12 h. After completion, the mixture was concentrated and purified by column chromatography on silica gel to afford **S5**.

To a solution of **S5** (10 mmol, 1.0 equiv) in THF (20 mL) was added NaH (15 mmol, 1.5 equiv) portionwise at 0 °C. The mixture was stirred for 15 minutes, and $(Boc)_2O$ (15 mmol, 1.5 equiv) was added dropwise. The mixture was then stirred at rt for 2 h. After completion, the mixture was quenched by saturated NH_4Cl solution at 0 °C and extracted with EtOAc. The organic layers were dried over Na_2SO_4, filtered and concentrated under reduced pressure. The residue was purified by column chromatography on silica gel to give MBH carbonate **8**.

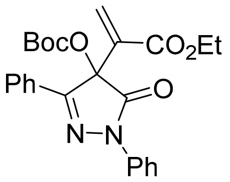

8a: as a white solid; mp 101–103 °C; ^{1}H NMR (400 MHz, $CDCl_3$): δ (ppm) 8.08–7.98 (m, 2H), 7.93–7.82 (m, 2H), 7.47–7.37 (m, 5H), 7.25–7.17 (m, 1H), 6.73 (s, 1H), 6.70 (s, 1H), 4.13–3.89 (m, 2H), 1.34 (s, 9H), 1.07 (t, J = 7.1 Hz, 3H); ^{13}C NMR (100 MHz, $CDCl_3$): δ (ppm) 168.1, 162.9, 151.5, 149.5, 138.1, 134.7, 130.52, 130.51, 129.3, 128.85, 128.78, 126.3, 125.3, 119.0, 84.8, 81.1, 61.5, 27.4, 13.7; HRMS (ESI-TOF) *m/z*: [M + Na]$^+$ Calcd for $C_{25}H_{26}N_2O_6Na^+$ 473.1683; Found 473.1686.

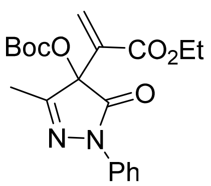

8b: as a white solid; mp 95–97 °C; ^{1}H NMR (400 MHz, $CDCl_3$): δ (ppm) 8.01–7.81 (m, 2H), 7.48–7.33 (m, 2H), 7.23–7.10 (m, 1H), 6.70 (s, 1H), 6.50 (s, 1H), 4.13 (q, J = 7.1 Hz, 2H), 2.04 (s, 3H), 1.44 (s, 9H), 1.15 (t, J = 7.1 Hz, 3H); ^{13}C NMR (100 MHz, $CDCl_3$): δ (ppm) 168.0, 162.9, 153.7, 149.6, 138.1, 133.3, 131.1, 128.8, 125.0, 118.7, 84.7, 81.7, 61.6, 27.5, 13.8, 12.9; HRMS (ESI-TOF) *m/z*: [M + Na]$^+$ Calcd for $C_{20}H_{24}N_2O_6Na^+$ 411.1527; Found 411.1534.

Ligands **L1**–**L21** were purchased from Bidepharm and used without purification. Other substrates, MBH carbonates **11**,[1] and allenes **2**[2] were prepared according to the literature procedures, and the spectroscopic data were consistent with the literature reports.

3. Detailed screening conditions

Scheme S1. Screening ligands for asymmetric (3+2) annulation of MBH carbonate 1a with allene 2a

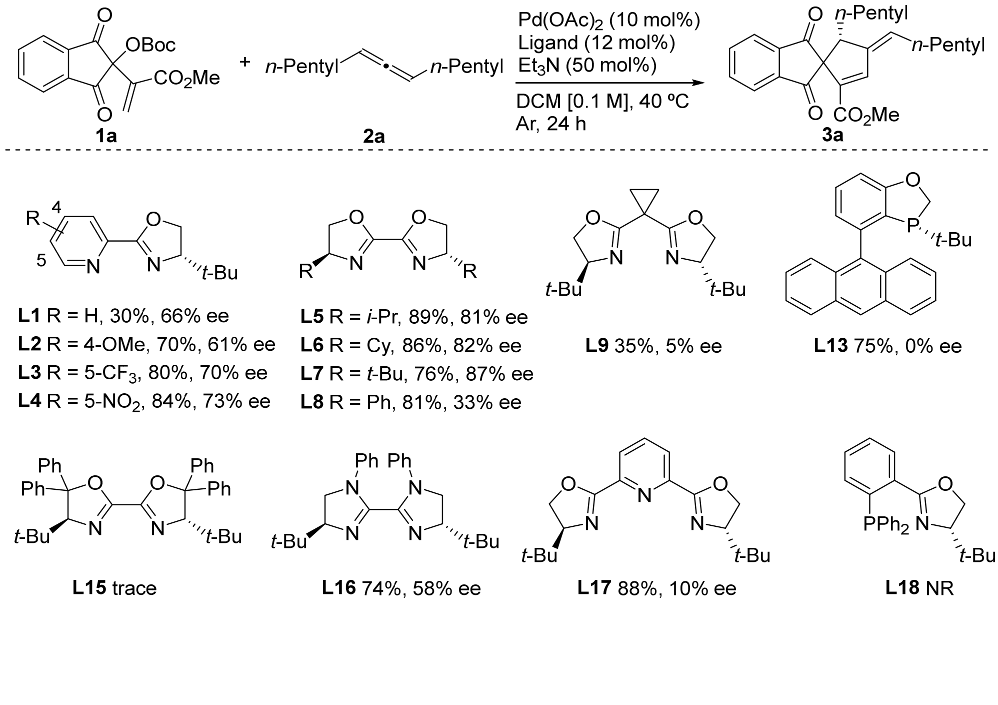

Table S1. Screening other parameters for asymmetric (3+2) annulations of MBH carbonate 1 with alkyl allene 2a[a]

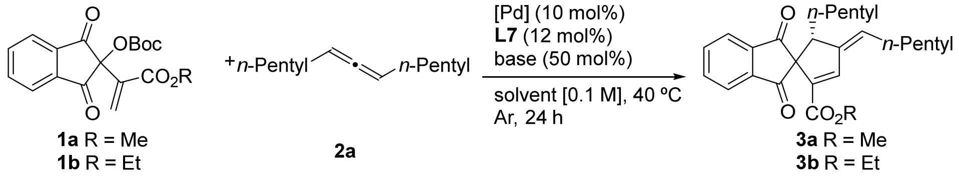

Entry	1	[Pd]	L	Base	Solvent	T (°C)	Yield(%)[b]	ee (%)[c]
1	**1a**	$Pd_2(dba)_3$	**L1**	/	DCM	40	**3a**, 76	64
2	**1a**	$Pd_2(dba)_3$	**L3**	/	DCM	40	**3a**, 80	65
3	**1a**	$Pd_2(dba)_3$	**L4**	/	DCM	40	**3a**, 81	72
4	**1a**	$Pd(OAc)_2$	**L7**	Et_3N	DCM	40	**3a**, 76	87
5	**1a**	$Pd(OAc)_2$	**L7**	Et_3N	DCE	40	**3a**, 63	85
6	**1a**	$Pd(OAc)_2$	**L7**	Et_3N	MeOH	40	**3a**, 34	89
7	**1a**	$Pd(OAc)_2$	**L7**	Et_3N	Toluene	40	**3a**, 17	89
8	**1a**	$Pd(OAc)_2$	**L7**	Et_3N	THF	40	**3a**, 74	88
9	**1a**	$Pd(OAc)_2$	**L7**	Et_3N	2-MeTHF	40	**3a**, 41	88
10	**1a**	$Pd(OAc)_2$	**L7**	Et_3N	1,4-Dioxane	40	**3a**, 74	87
11	**1a**	$Pd(OAc)_2$	**L7**	Et_3N	EtOAc	40	**3a**, 35	86
12	**1b**	$Pd(OAc)_2$	**L7**	Et_3N	DCM	40	**3b**, 81	90
13	**1b**	$Pd(TFA)_2$	**L7**	Et_3N	DCM	40	**3b**, 93	92
14	**1b**	$[Pd(allyl)Cl]_2$	**L7**	Et_3N	DCM	40	NR	/
15	**1b**	$PdCl_2$	**L7**	Et_3N	DCM	40	**3b**, 28	35
16	**1b**	Pd(allyl)Cp	**L7**	Et_3N	DCM	40	**3b**, 15	61
17	**1b**	$Pd(MeCN)_2Cl_2$	**L7**	Et_3N	DCM	40	**3b**, 36	34
18	**1b**	$Pd(PPh_3)_2Cl_2$	**L7**	Et_3N	DCM	40	**3b**, 18	4
19	**1b**	$Pd(acca)_2$	**L7**	Et_3N	DCM	40	NR	/
20	**1b**	$Pd_2(dba)_3$	**L7**	Et_3N	DCM	40	**3b**, 70	81
21	**1b**	$Pd(TFA)_2$	**L7**	/	DCM	40	NR	/
22	**1b**	$Pd(TFA)_2$	**L7**	DIPEA	DCM	40	**3b**, 55	88
23	**1b**	$Pd(TFA)_2$	**L7**	K_2CO_3	DCM	40	**3b**,12	86
24[d]	**1b**	$Pd(TFA)_2$	**L7**	Et_3N	DCM	40	**3b**, 40	92
25	**1b**	$Pd(TFA)_2$	**L7**	Et_3N	DCM	rt	**3b**, 68	93
26	**1b**	$Pd(TFA)_2$	**L7**	Et_3N	DCM	0	NR	/
27	**1b**	$Pd(TFA)_2$	**L7**	Et_3N	DCM	60	**3b**, 89	86

28[e]	**1b**	Pd(TFA)$_2$	**L7**	Et$_3$N	DCM	40	**3b**, 84	91

[a] Unless noted otherwise, the reaction was performed with MBH carbonate**1a** (0.1 mmol), allene **2a** (0.2 mmol), Pd (OAc)$_2$ (10 mol%), **L7** (12 mol%) and Et$_3$N (0.05 mmol) in dried solvent (1.0 mL) at 40 °C for 24 h. [b] Yield of the isolated product. [c] Determined by HPLC analysis on a chiral stationary phase. [d] Pd(TFA)$_2$ (5 mol%) and **L7** (6 mol%). [e] With **2a** (0.1 mmol).

Table S2. Screening conditions for asymmetric (3+2) annulation of MBH carbonate 1b with aryl allene 2m[a]

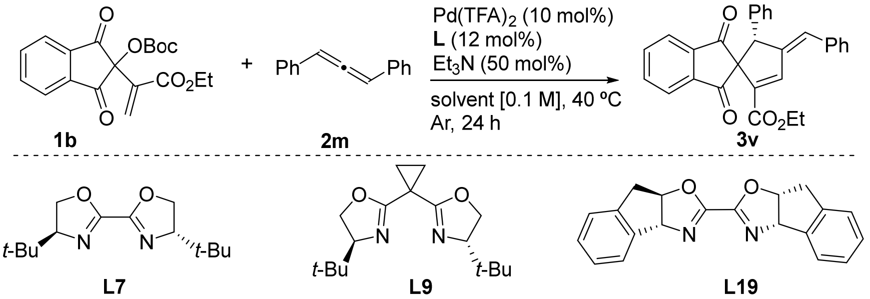

Entry	L	Solvent	Yield (%)[b]	ee (%)[c]
1	**L7**	DCM	45	3
2	**L9**	DCM	84	89
3	**L19**	DCM	53	59
4	**L9**	DCE	88	90
5	**L9**	MeCN	48	6
6	**L9**	Toluene	trace	/
7	**L9**	MTBE	trace	/

[a] Unless noted otherwise, reactions were performed with MBH carbonate **1b** (0.1 mmol), allene **2m** (0.2 mmol), Pd(TFA)$_2$ (10 mol%), **L** (12 mol%) and Et$_3$N (0.05 mmol) in dried solvent (1.0 mL) at 40 °C for 24 h. [b] Yield of the isolated product. [c] Determined by HPLC analysis on a chiral stationary phase.

Table S3. Screening conditions for asymmetric (3+2) annulation of MBH carbonate 1b with terminal allene 2t[a]

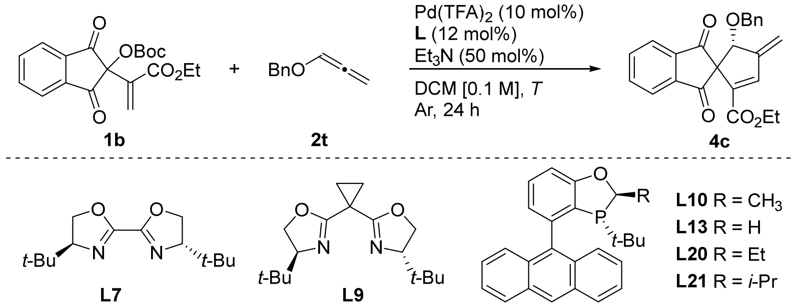

Entry	L	T (°C)	Yield (%)[b]	ee (%)[c]
1	**L7**	40	24	0
2	**L9**	40	43	19
3	**L10**	40	98	83
4	**L13**	40	80	61
5	**L20**	40	97	80
6	**L21**	40	89	73
7	**L10**	rt	97	85
8	**L10**	0	trace	/

[a] Unless noted otherwise, reactions were performed with MBH carbonate **1b** (0.1 mmol), terminal allene **2t** (0.2 mmol), $Pd(TFA)_2$ (10 mol%), **L** (12 mol%) and Et_3N (0.05 mmol) in DCM (1.0 mL) at 40 °C for 24 h. [b] Yield of the isolated product. [c] Determined by HPLC analysis on a chiral stationary phase.

Table S4. Screening conditions for asymmetric (3+2) annulation of MBH carbonate 1b with alkene 6a[a]

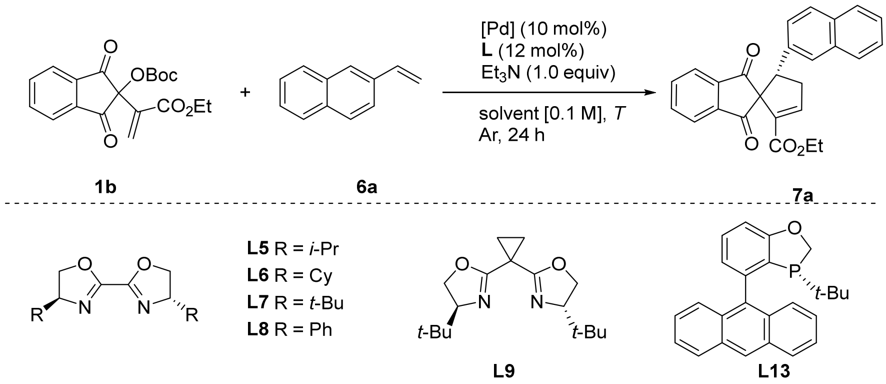

Entry	[Pd]	L	Additive	Solvent	T (°C)	Yield (%)[b]	ee (%)[c]
1	$Pd(TFA)_2$	**L5**	/	DCM	60	NR	/
2	$Pd(TFA)_2$	**L6**	/	DCM	60	12	29
3	$Pd(TFA)_2$	**L7**	/	DCM	60	27	43
4	$Pd(TFA)_2$	**L8**	/	DCM	60	20	70
5	$Pd(TFA)_2$	**L9**	/	DCM	60	NR	/
6	$Pd(TFA)_2$	**L13**	/	DCM	60	NR	/
7	$Pd(TFA)_2$	**L8**	/	EtOH	60	27	74
8	$Pd(TFA)_2$	**L8**	/	MeCN	60	trace	/
9	$Pd(TFA)_2$	**L8**	/	DCE	60	trace	/
10	$Pd(TFA)_2$	**L8**	/	EtOAc	60	trace	/
11	$Pd(TFA)_2$	**L8**	/	EtOH	40	29	74

12	$Pd(TFA)_2$	**L8**	/	EtOH	rt	NR	/
13	$Pd(OAc)_2$	**L8**	/	EtOH	40	29	76
14	$PdCl_2$	**L8**	/	EtOH	40	22	76
15	$Pd_2(dba)_3$	**L8**	/	EtOH	40	24	76
16[d]	$Pd(OAc)_2$	**L8**	/	EtOH	40	38	82
17[d]	$Pd(OAc)_2$	**L8**	TBAC	EtOH	40	43	84
18[d]	$Pd(OAc)_2$	**L8**	TBAB	EtOH	40	40	82
19[d]	$Pd(OAc)_2$	**L8**	TBAI	EtOH	40	NR	/

[a] Unless noted otherwise, reactions were performed with carbonate **1b** (0.1 mmol), alkene **6a** (0.2 mmol), palladium (10 mol%), **L** (12 mol%) and Et_3N (0.1 mmol) in dried solvent (1.0 mL) at 60 °C for 24 h. [b] Yield of the isolated product. [c] Determined by HPLC analysis on a chiral stationary phase. [d] With alkene **6a** (0.5 mmol). TBAC = tetrabutyl ammonium chloride, TBAB = tetrabutylammonium bromide, TBAI = tetrabutylammonium iodide.

4. General procedure for (3+2) annulations of MBH carbonates with diverse allenes or alkenes

4.1 General procedure for (3+2) annulations of MBH carbonates with 1,3-dialkyl-substituted allenes

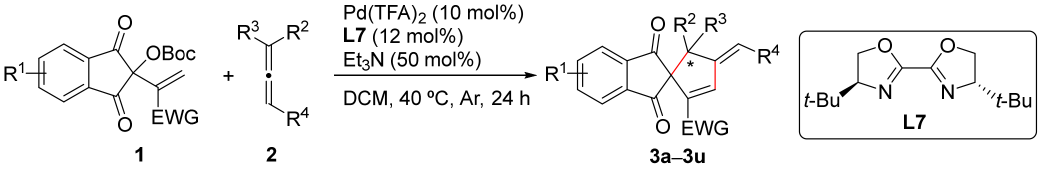

General procedure A: To an oven-dried 10 mL Schlenk tube equipped with a stir bar were added $Pd(TFA)_2$ (10 mol%), **L7** (12 mol%), Et_3N (50 mol%), MBH carbonate **1** (0.10 mmol, 1.0 equiv) and allene **2** (0.20 mmol, 2.0 equiv). The tube was evacuated and back-filled with argon for five times. Then degassed DCM (1.0 mL) was added by syringe. The mixture was stirred at 40 °C for 24 h. After completion, the mixture was concentrated and purified by flash chromatography on silica gel (EtOAc/petroleum ether) to give products **3a**–**3u**.

Synthesis of racemic 3a–3u: *Rac*-**3a**–**3u** were obtained under the catalysis of $Pd(TFA)_2$, (±)-**L7** and Et_3N.

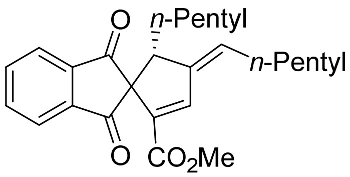

Synthesis of 3a (General procedure A): To an oven-dried 10 mL Schlenk tube equipped with a stir bar were added $Pd(TFA)_2$ (3.3 mg, 0.0099 mmol, 10 mol%), **L7** (3.0 mg, 0.012 mmol, 12 mol%), Et_3N (7.0 μL, 0.050 mmol, 50 mol%), methyl 2-(2-((*tert*-butoxycarbonyl) oxy)-

1,3-dioxo-2,3-dihydro-1*H*-inden-2-yl)acrylate **1a** (34.6 mg, 0.0999 mmol, 1.0 equiv) and trideca-6,7-diene **2a** (36.1 mg, 0.200 mmol, 2.0 equiv). The tube was evacuated and back-filled with argon for five times. Then degassed DCM (1.0 mL) was added by syringe. The mixture was stirred at 40 °C for 24 h. After completion, the mixture was concentrated and purified by flash chromatography on silica gel (EtOAc/petroleum ether) to give product **3a**: as a yellow solid, 32.7 mg (0.0801 mmol), 80% yield; mp 92–93 °C; *E/Z* >19:1; $[\alpha]_D^{25}$ = +16.0 (*c* = 1.89, in $CHCl_3$); 89% ee, determined by HPLC analysis [Chiralpak column IA, *i*-PrOH/*n*-Hexane = 5/95, flow rate: 1.0 mL/min, 254 nm, t (minor) = 9.67 min, t (major) = 10.69 min]; ^{1}H NMR (400 MHz, $CDCl_3$) δ (ppm) 8.05–7.93 (m, 2H), 7.88–7.79 (m, 2H), 7.64 (s, 1H), 5.63–5.45 (m, 1H), 3.54 (s, 3H), 3.39–3.26 (m, 1H), 2.37–2.21 (m, 2H), 1.68–1.59 (m, 1H), 1.57–1.49 (m, 1H), 1.49–1.40 (m, 2H), 1.37–1.27 (m, 4H), 1.09–0.87 (m, 8H), 0.79–0.67 (m, 1H), 0.66–0.55 (m, 3H); ^{13}C NMR (100 MHz, $CDCl_3$) δ (ppm) 202.3, 199.6, 164.4, 144.3, 144.0, 142.4, 142.1, 136.3, 135.49, 135.47, 130.2, 123.3, 123.0, 68.3, 52.1, 51.8, 31.4, 30.1, 30.0, 29.4, 28.0, 22.5, 22.0, 14.0, 13.6; HRMS (ESI-TOF) *m/z*: $[M + Na]^+$ Calcd for $C_{26}H_{32}O_4Na^+$ 431.2193; Found 431.2196.

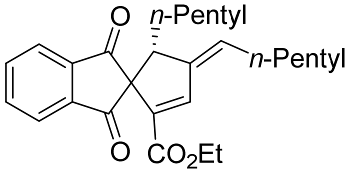

Synthesis of 3b (General procedure A): To an oven-dried 10 mL Schlenk tube equipped with a stir bar were added $Pd(TFA)_2$ (3.3 mg, 0.0099 mmol, 10 mol%), **L7** (3.0 mg, 0.012 mmol, 12 mol%), Et_3N (7.0 μL, 0.050 mmol, 50 mol%), ethyl 2-(2-((*tert*-butoxycarbonyl)oxy)-1,3-dioxo-2,3-dihydro-1*H*-inden-2-yl)acrylate **1b** (36.0 mg, 0.0999 mmol, 1.0 equiv) and trideca-6,7-diene **2a** (36.1 mg, 0.200 mmol, 2.0 equiv). The tube was evacuated and back-filled with argon for five times. Then degassed DCM (1.0 mL) was added by syringe. The mixture was stirred at 40 °C for 24 h. After completion, the mixture was concentrated and purified by flash chromatography on silica gel (EtOAc/petroleum ether) to give product **3b**: as a colorless oil, 39.2 mg (0.0927 mmol), 93% yield; *E/Z* >19:1; $[\alpha]_D^{25}$ = +10.3 (*c* = 0.59, in MeOH); 92% ee, determined by HPLC analysis [Chiralpak column IA, *i*-PrOH/*n*-Hexane = 5/95, flow rate: 1.0 mL/min, 254 nm, t (minor) = 11.06 min, t (major) = 12.09 min]; ^{1}H NMR (400 MHz, $CDCl_3$) δ (ppm) 8.05–7.97 (m, 2H), 7.88–7.82 (m, 2H), 7.66 (s, 1H), 5.64–5.40 (m, 1H), 3.95 (q, *J* = 7.2 Hz, 2H), 3.40–3.25 (m, 1H), 2.35–2.23 (m, 2H), 1.64–1.53 (m, 2H), 1.48–1.40 (m, 2H), 1.36–1.29 (m, 4H), 1.08–0.89 (m, 11H), 0.76–0.68 (m, 1H), 0.63 (t, *J* = 7.0 Hz, 3H); ^{13}C NMR (100 MHz, $CDCl_3$) δ (ppm) 202.4, 199.7, 163.7, 144.2, 144.0, 142.5, 142.2, 136.5, 135.44, 135.40, 129.9, 123.2, 123.0, 68.2, 60.8, 52.2, 31.4, 30.1, 30.0, 29.4, 28.0, 22.5, 22.0, 14.0, 13.6; HRMS (ESI-TOF) *m/z*: $[M + Na]^+$ Calcd for $C_{27}H_{34}O_4Na^+$ 445.2349; Found 445.2353.

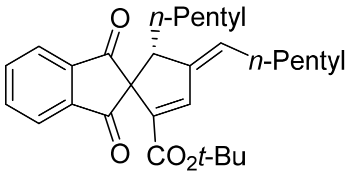

Synthesis of 3c (General procedure A): To an oven-dried 10 mL Schlenk tube equipped with a stir bar were added Pd(TFA)$_2$ (3.3 mg, 0.0099 mmol, 10 mol%), **L7** (3.0 mg, 0.012 mmol, 12 mol%), Et$_3$N (7.0 *μ*L, 0.050 mmol, 50 mol%), *tert*-butyl 2-(2-((*tert*-butoxycarbonyl) oxy)-1,3-dioxo-2,3-dihydro-1*H*-inden-2-yl)acrylate **1c** (38.8 mg, 0.0999 mmol, 1.0 equiv) and trideca-6,7-diene **2a** (36.1 mg, 0.200 mmol, 2.0 equiv). The tube was evacuated and back-filled with argon for five times. Then degassed DCM (1.0 mL) was added by syringe. The mixture was stirred at 40 °C for 24 h. After completion, the mixture was concentrated and purified by flash chromatography on silica gel (EtOAc/petroleum ether) to give product **3c**: as a yellow oil, 36.5 mg (0.0810 mmol), 81% yield; *E/Z* >19:1; $[\alpha]_D^{25}$ = +11.0 (*c* = 1.07, in CHCl$_3$); 92% ee, determined by HPLC analysis [Chiralpak column IA, *i*-PrOH/*n*-Hexane = 5/95, flow rate: 1.0 mL/min, 254 nm, t (minor) = 8.76 min, t (major) = 9.37 min]; ^{1}H NMR (400 MHz, CDCl$_3$) δ (ppm) 8.11–7.93 (m, 2H), 7.91–7.78 (m, 2H), 7.63 (s, 1H), 5.50 (t, *J* = 7.2 Hz, 1H), 3.38–3.21 (m, 1H), 2.28 (q, *J* = 7.2 Hz, 2H), 1.63–1.56 (m, 1H), 1.51 (ddd, *J* = 14.7, 9.7, 5.0 Hz, 1H), 1.46–1.39 (m, 2H), 1.36–1.29 (m, 4H), 1.28–1.21 (m, 1H), 1.10 (s, 9H), 1.05–0.95 (m, 4H), 0.93–0.89 (m, 3H), 0.75–0.66 (m, 1H), 0.63 (t, *J* = 6.9 Hz, 3H); ^{13}C NMR (100 MHz, CDCl$_3$) δ (ppm) 202.4, 199.7, 162.7, 144.1, 143.9, 142.7, 142.2, 137.9, 135.4, 135.3, 129.2, 123.3, 122.9, 81.5, 68.1, 52.6, 31.5, 31.4, 30.3, 29.9, 29.5, 28.0, 27.5, 22.5, 22.0, 14.0, 13.6; HRMS (ESI-TOF) *m/z*: $[M + Na]^+$ Calcd for $C_{29}H_{38}O_4Na^+$ 473.2662; Found 473.2652.

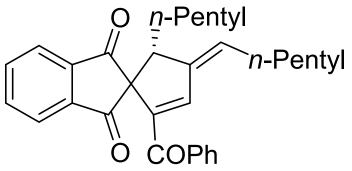

Synthesis of 3d (General procedure A): To an oven-dried 10 mL Schlenk tube equipped with a stir bar were added Pd(TFA)$_2$ (3.3 mg, 0.0099 mmol, 10 mol%), **L7** (3.0 mg, 0.012 mmol, 12 mol%), Et$_3$N (7.0 *μ*L, 0.050 mmol, 50 mol%), *tert*-butyl (1,3-dioxo-2-(3-oxo-3-phenylprop-1-en-2-yl)-2,3-dihydro-1*H*-inden-2-yl) carbonate **1d** (39.2 mg, 0.0999 mmol, 1.0 equiv) and trideca-6,7-diene **2a** (36.1 mg, 0.200 mmol, 2.0 equiv). The tube was evacuated and back-filled with argon for five times. Then degassed DCM (1.0 mL) was added by syringe. The mixture was stirred at 40 °C for 24 h. After completion, the mixture was concentrated and purified by flash chromatography on silica gel (EtOAc/petroleum ether) to give product **3d**: as a yellow oil, 35.2 mg (0.0774 mmol), 77% yield; *E/Z* >19:1; $[\alpha]_D^{25}$ = +10.6 (*c* = 0.36, in CHCl$_3$); 85% ee, determined by HPLC analysis [Chiralpak column IA, *i*-PrOH/*n*-Hexane = 10/90, flow rate: 1.0 mL/min, 254 nm, t (minor) = 8.79 min, t (major) = 10.31 min]; ^{1}H NMR (400 MHz, CDCl$_3$) δ (ppm) 8.08–7.97 (m, 2H), 7.89–7.80 (m, 2H), 7.72–7.64 (m, 2H), 7.56–7.50 (m, 1H), 7.46–7.36 (m, 3H), 5.66 (td, *J* = 7.9, 2.3 Hz, 1H), 3.43–3.30 (m, 1H), 2.33–2.17 (m,

2H), 1.73–1.62 (m, 1H), 1.58–1.52 (m, 1H), 1.49–1.39 (m, 2H), 1.35–1.28 (m, 4H), 1.15–0.97 (m, 5H), 0.92–0.87 (m, 3H), 0.84–0.72 (m, 1H), 0.65 (t, J = 6.9 Hz, 3H); ^{13}C NMR (100 MHz, $CDCl_3$) δ (ppm) 202.3, 199.5, 191.8, 145.6, 145.4, 144.8, 142.5, 142.2, 137.4, 135.4, 135.3, 132.4, 131.5, 129.0, 128.4, 123.3, 123.0, 69.1, 51.4, 31.5, 31.4, 30.1, 30.0, 29.3, 28.0, 22.5, 22.1, 14.0, 13.6; HRMS (ESI-TOF) *m/z*: $[M + Na]^+$ Calcd for $C_{31}H_{34}O_3Na^+$ 477.2400; Found 477.2403.

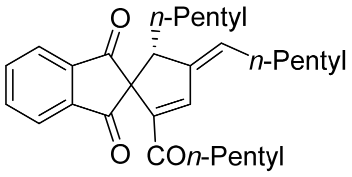

Synthesis of 3e (General procedure A): To an oven-dried 10 mL Schlenk tube equipped with a stir bar were added $Pd(TFA)_2$ (3.3 mg, 0.0099 mmol, 10 mol%), **L7** (3.0 mg, 0.012 mmol, 12 mol%), Et_3N (7.0 μL, 0.050 mmol, 50 mol%), *tert*-butyl (1,3-dioxo-2-(3-oxooct-1-en-2-yl)-2,3-dihydro-1*H*-inden-2-yl) carbonate **1e** (38.6 mg, 0.0999 mmol, 1.0 equiv) and trideca-6,7-diene **2a** (36.1 mg, 0.200 mmol, 2.0 equiv). The tube was evacuated and back-filled with argon for five times. Then degassed DCM (1.0 mL) was added by syringe. The mixture was stirred at 40 °C for 24 h. After completion, the mixture was concentrated and purified by flash chromatography on silica gel (EtOAc/petroleum ether) to give product **3e**: as a colorless oil, 41.7 mg (0.0929 mmol), 93% yield; *E/Z* >19:1; $[\alpha]_D^{25}$ = +9.2 (c = 0.61, in $CHCl_3$); 86% ee, determined by HPLC analysis [Chiralpak column AS-H, *i*-PrOH/*n*-Hexane = 5/95, flow rate: 1.0 mL/min, 254 nm, t (minor) = 5.44 min, t (major) = 6.83 min]; ^{1}H NMR (400 MHz, $CDCl_3$) δ (ppm) 8.06–7.89 (m, 2H), 7.88–7.74 (m, 2H), 7.54 (s, 1H), 5.61 (t, J = 7.5 Hz, 1H), 3.32–3.16 (m, 1H), 2.65 (t, J = 7.5 Hz, 2H), 2.32 (q, J = 7.5 Hz, 2H), 1.64–1.57 (m, 1H), 1.54–1.42 (m, 5H), 1.39–1.31 (m, 4H), 1.28–1.17 (m, 4H), 1.10–0.89 (m, 8H), 0.88–0.80 (m, 3H), 0.76–0.66 (m, 1H), 0.66–0.53 (m, 3H); ^{13}C NMR (100 MHz, $CDCl_3$) δ (ppm) 202.5, 199.8, 197.5, 146.0, 145.2, 142.3, 142.2, 142.1, 135.24, 135.21, 131.0, 123.2, 122.9, 68.3, 51.5, 38.0, 31.4, 31.2, 30.1, 29.4, 28.0, 23.6, 22.5, 22.4, 22.0, 14.0, 13.8, 13.6; HRMS (ESI-TOF) *m/z*: $[M + Na]^+$ Calcd for $C_{30}H_{40}O_3Na^+$ 471.2870; Found 471.2867.

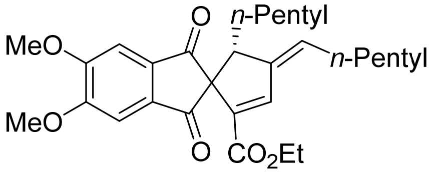

Synthesis of 3f (General procedure A): To an oven-dried 10 mL Schlenk tube equipped with a stir bar were added $Pd(TFA)_2$ (3.3 mg, 0.0099 mmol, 10 mol%), **L7** (3.0 mg, 0.012 mmol, 12 mol%), Et_3N (7.0 μL, 0.050 mmol, 50 mol%), ethyl 2-(2-((*tert*-butoxy carbonyl)oxy)-5,6-dimethoxy-1,3-dioxo-2,3-dihydro-1*H*-inden-2-yl)acrylate **1f** (42.0 mg, 0.0999 mmol, 1.0 equiv) and trideca-6,7-diene **2a** (36.1 mg, 0.200 mmol, 2.0 equiv). The tube was evacuated and back-filled with argon for five times. Then degassed DCM (1.0 mL) was added by syringe. The

mixture was stirred at 40 °C for 24 h. After completion, the mixture was concentrated and purified by flash chromatography on silica gel (EtOAc/petroleum ether) to give product **3f**: as a colorless oil, 43.2 mg (0.0896 mmol), 90% yield; *E/Z* >19:1; $[\alpha]_D^{25}$ = +15.1 (*c* = 0.65, in $CHCl_3$); 90% ee, determined by HPLC analysis [Chiralpak column IA, *i*-PrOH/*n*-Hexane = 20/80, flow rate: 1.0 mL/min, 254 nm, t (minor) = 7.51 min, t (major) = 10.03 min]; ^1H NMR (400 MHz, $CDCl_3$) δ (ppm) 7.64 (s, 1H), 7.39 (s, 1H), 7.37 (s, 1H), 5.55 (t, *J* = 7.6 Hz, 1H), 4.04 (d, *J* = 2.7 Hz, 6H), 4.02–3.96 (q, *J* = 7.2 Hz, 2H), 3.38–3.20 (m, 1H), 2.29 (q, *J* = 7.2 Hz, 2H), 1.67–1.51 (m, 2H), 1.48–1.40 (m, 2H), 1.37–1.29 (m, 4H), 1.12–0.98 (m, 8H), 0.94–0.89 (m, 3H), 0.86–0.77 (m, 1H), 0.73–0.63 (m, 3H); ^{13}C NMR (100 MHz, $CDCl_3$) δ (ppm) 201.2, 198.4, 163.9, 155.71, 155.66, 144.4, 143.8, 137.4, 137.2, 136.4, 129.7, 103.5, 103.3, 68.0, 60.7, 56.63, 56.61, 51.5, 31.5, 31.4, 30.1, 30.0, 29.4, 27.9, 22.5, 22.1, 14.0, 13.8, 13.7; HRMS (ESI-TOF) *m/z*: $[M + H]^+$ Calcd for $C_{29}H_{39}O_6^+$ 483.2741; Found 483.2739.

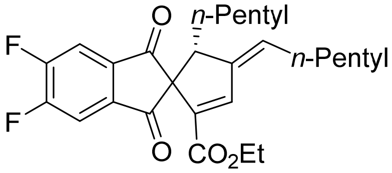

Synthesis of 3g (General procedure A): To an oven-dried 10 mL Schlenk tube equipped with a stir bar were added $Pd(TFA)_2$ (3.3 mg, 0.0099 mmol, 10 mol%), **L7** (3.0 mg, 0.012 mmol, 12 mol%), Et_3N (7.0 μL, 0.050 mmol, 50 mol%), ethyl 2-(2-((*tert*-butoxycarbonyl) oxy)-5,6-difluoro-1,3-dioxo-2,3-dihydro-1*H*-inden-2-yl)acrylate **1g** (39.6 mg, 0.0999 mmol, 1.0 equiv) and trideca-6,7-diene **2a** (36.1 mg, 0.200 mmol, 2.0 equiv). The tube was evacuated and back-filled with argon for five times. Then degassed DCM (1.0 mL) was added by syringe. The mixture was stirred at 40 °C for 24 h. After completion, the mixture was concentrated and purified by flash chromatography on silica gel (EtOAc/petroleum ether) to give product **3g**: as a colorless oil, 41.6 mg (0.0907 mmol), 91% yield; *E/Z* >19:1; $[\alpha]_D^{25}$ = +19.8 (*c* = 0.55, in MeOH); 89% ee, determined by HPLC analysis [Chiralpak column IA, *i*-PrOH/*n*-Hexane = 10/90, flow rate: 1.0 mL/min, 254 nm, t (minor) = 4.82 min, t (major) = 5.74 min]; ^1H NMR (400 MHz, $CDCl_3$) δ (ppm) 7.85–7.72 (m, 2H), 7.63 (s, 1H), 5.57 (t, *J* = 7.6 Hz, 1H), 4.00 (q, *J* = 7.1 Hz, 2H), 3.32 (d, *J* = 8.1 Hz, 1H), 2.29 (q, *J* = 7.5 Hz, 2H), 1.69–1.60 (m, 1H), 1.57–1.49 (m, 1H), 1.49–1.41 (m, 2H), 1.40–1.26 (m, 5H), 1.10–1.03 (m, 6H), 0.96–0.87 (m, 4H), 0.76–0.66 (m, 4H); ^{13}C NMR (100 MHz, $CDCl_3$) δ (ppm) 199.9, 197.3, 163.8, 155.7 (dd, *J* = 264.9, 3.9 Hz), 155.6 (dd, *J* = 265.0, 3.8 Hz), 144.0, 143.9, 139.5 (dd, *J* = 5.5, 3.6 Hz), 139.3 (dd, *J* = 5.5, 3.9 Hz)., 136.2, 130.5, 112.0 (dd, *J* = 11.9, 8.4 Hz), 111.7 (dd, *J* = 16.4, 4.0 Hz), 68.0, 61.0, 52.2, 31.45, 31.42, 30.04, 29.99, 29.4, 28.1, 22.5, 22.1, 14.0, 13.8, 13.6; ^{19}F NMR (376 MHz, $CDCl_3$) δ (ppm) –122.5; HRMS (ESI-TOF) *m/z*: $[M + Na]^+$ Calcd for

$C_{27}H_{32}F_2O_4Na^+$ 481.2161; Found 481.2167.

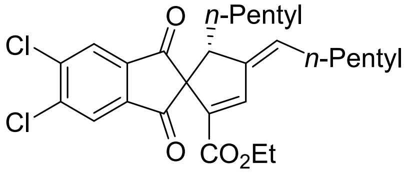

Synthesis of 3h (General procedure A): To an oven-dried 10 mL Schlenk tube equipped with a stir bar were added Pd(TFA)$_2$ (3.3 mg, 0.0099 mmol, 10 mol%), **L7** (3.0 mg, 0.012 mmol, 12 mol%), Et_3N (7.0 μL, 0.050 mmol, 50 mol%), ethyl 2-(2-((*tert*-butoxycarbonyl) oxy)-5,6-dichloro-1,3-dioxo-2,3-dihydro-1*H*-inden-2-yl)acrylate **1h** (42.9 mg, 0.0999 mmol, 1.0 equiv) and trideca-6,7-diene **2a** (36.1 mg, 0.200 mmol, 2.0 equiv). The tube was evacuated and back-filled with argon for five times. Then degassed DCM (1.0 mL) was added by syringe. The mixture was stirred at 40 °C for 24 h. After completion, the mixture was concentrated and purified by flash chromatography on silica gel (EtOAc/petroleum ether) to give product **3h**: as a colorless oil, 46.0 mg (0.0936 mmol), 94% yield; *E/Z* >19:1; $[\alpha]_D^{25}$ = +30.2 (c = 0.98, in $CHCl_3$); 88% ee, determined by HPLC analysis [Chiralpak column IA, *i*-PrOH/*n*-Hexane = 10/90, flow rate: 1.0 mL/min, 254 nm, t (minor) = 4.78 min, t (major) = 5.70 min]; ^{1}H NMR (400 MHz, $CDCl_3$) δ (ppm) 8.00 (d, J = 10.8 Hz, 2H), 7.55 (s, 1H), 5.50 (td, J = 7.9, 2.3 Hz, 1H), 3.93 (q, J = 7.1 Hz, 2H), 3.32–3.17 (m, 1H), 2.27–2.16 (m, 2H), 1.56 (td, J = 10.1, 5.3 Hz, 1H), 1.50–1.42 (m, 1H), 1.40–1.34 (m, 2H), 1.33–1.19 (m, 5H), 1.05–0.97 (m, 6H), 0.88–0.79 (m, 4H), 0.70–0.58 (m, 4H); ^{13}C NMR (100 MHz, $CDCl_3$) δ (ppm) 200.1, 197.5, 163.8, 143.93, 143.88, 141.0, 140.8, 140.74, 140.72, 136.4, 130.5, 125.0, 124.7, 68.3, 61.1, 52.3, 31.4, 31.4, 30.04, 29.97, 29.4, 28.1, 22.5, 22.0, 14.0, 13.8, 13.6; HRMS (ESI-TOF) *m/z*: $[M + Na]^+$ Calcd for $C_{27}H_{32}{}^{35}Cl_2O_4Na^+$ 513.1570; Found 513.1565; $[M + Na]^+$ Calcd for $C_{27}H_{32}{}^{35}Cl\,{}^{37}ClO_4Na^+$ 515.1540; Found 515.1547.

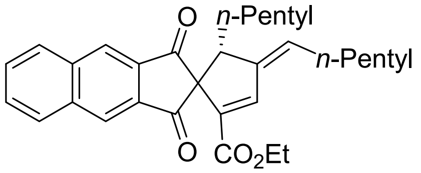

Synthesis of 3i (General procedure A): To an oven-dried 10 mL Schlenk tube equipped with a stir bar were added Pd(TFA)$_2$ (3.3 mg, 0.0099 mmol, 10 mol%), **L7** (3.0 mg, 0.012 mmol, 12 mol%), Et_3N (7.0 μL, 0.050 mmol, 50 mol%), ethyl 2-(2-((*tert*-butoxycarbonyl) oxy)-1,3-dioxo-2,3-dihydro-1*H*-cyclopenta[*b*]naphthalen-2-yl)acrylate **1i** (41.0 mg, 0.0999 mmol, 1.0 equiv) and trideca-6,7-diene **2a** (36.1 mg, 0.200 mmol, 2.0 equiv). The tube was evacuated and back-filled with argon for five times. Then degassed DCM (1.0 mL) was added by syringe. The mixture was stirred at 40 °C for 24 h. After completion, the mixture was concentrated and purified by flash chromatography on silica gel (EtOAc/petroleum ether) to give product **3i**: as a colorless oil, 38.7 mg (0.0820 mmol), 82% yield; *E/Z* >19:1; $[\alpha]_D^{25}$ = +21.6 (c = 1.92, in $CHCl_3$); 84%

ee, determined by HPLC analysis [Chiralpak column IA, *i*-PrOH/*n*-Hexane = 20/80, flow rate: 1.0 mL/min, 254 nm, t (major) = 14.97 min, t (minor) = 18.76 min]; ^{1}H NMR (400 MHz, $CDCl_3$) δ (ppm) 8.55 (d, *J* = 10.5 Hz, 2H), 8.16–8.07 (m, 2H), 7.76–7.69 (m, 2H), 7.68 (s, 1H), 5.57 (td, *J* = 7.8, 1.3 Hz, 1H), 3.94 (q, *J* = 7.1 Hz, 2H), 3.50–3.37 (m, 1H), 2.40–2.23 (m, 2H), 1.68–1.57 (m, 2H), 1.50–1.41 (m, 2H), 1.39–1.30 (m, 4H), 1.10–0.89 (m, 11H), 0.78–0.66 (m, 1H), 0.56 (t, *J* = 7.0 Hz, 3H); ^{13}C NMR (100 MHz, $CDCl_3$) δ (ppm) 202.8, 200.0, 163.8, 144.3, 143.8, 137.4, 137.3, 137.0, 136.41, 136.39, 130.60, 130.59, 129.9, 129.4, 124.2, 123.9, 69.6, 60.9, 52.8, 31.44, 31.42, 30.1, 30.0, 29.4, 28.1, 22.5, 21.9, 14.0, 13.63, 13.55; HRMS (ESI-TOF) *m/z*: [M + H]$^+$ Calcd for $C_{31}H_{37}O_4^+$ 473.2686; Found 473.2684.

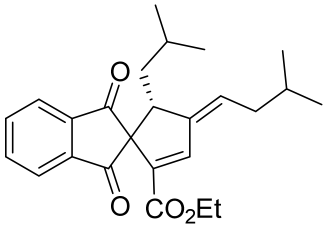

Synthesis of 3j (General procedure A): To an oven-dried 10 mL Schlenk tube equipped with a stir bar were added $Pd(TFA)_2$ (3.3 mg, 0.0099 mmol, 10 mol%), **L7** (3.0 mg, 0.012 mmol, 12 mol%), Et_3N (7.0 μL, 0.050 mmol, 50 mol%), ethyl 2-(2-((*tert*-butoxycarbonyl)oxy)-1,3-dioxo-2,3-dihydro-1*H*-inden-2-yl)acrylate **1b** (36.0 mg, 0.0999 mmol, 1.0 equiv) and 2,8-dimethylnona-4,5-diene **2b** (30.5 mg, 0.200 mmol, 2.0 equiv). The tube was evacuated and back-filled with argon for five times. Then degassed DCM (1.0 mL) was added by syringe. The mixture was stirred at 40 °C for 24 h. After completion, the mixture was concentrated and purified by flash chromatography on silica gel (EtOAc/petroleum ether) to give product **3j**: as a colorless oil, 31.6 mg (0.0801 mmol), 80% yield; *E/Z* >19:1; $[\alpha]_D^{25}$ = +17.4 (*c* = 0.62, in $CHCl_3$); 91% ee, determined by HPLC analysis [Chiralpak column IA, *i*-PrOH/*n*-Hexane = 1/99, flow rate: 1.0 mL/min, 254 nm, t (minor) = 19.37 min, t (major) = 21.35 min]; ^{1}H NMR (400 MHz, $CDCl_3$) δ (ppm) 8.06–7.94 (m, 2H), 7.91–7.79 (m, 2H), 7.65 (s, 1H), 5.55 (td, *J* = 7.9, 2.2 Hz, 1H), 4.06–3.83 (m, 2H), 3.54–3.31 (m, 1H), 2.26–2.09 (m, 2H), 1.80–1.69 (m, 1H), 1.59–1.46 (m, 1H), 1.45–1.31 (m, 1H), 0.99–0.90 (m, 10H), 0.71–0.59 (m, 6H); ^{13}C NMR (100 MHz, $CDCl_3$) δ (ppm) 202.3, 199.7, 163.7, 145.4, 144.0, 142.4, 142.1, 136.7, 135.5, 135.4, 128.5, 123.2, 122.9, 68.2, 60.8, 49.6, 39.5, 39.0, 28.9, 26.6, 23.3, 22.4, 22.3, 21.4, 13.6; HRMS (ESI-TOF) *m/z*: [M + Na]$^+$ Calcd for $C_{25}H_{30}O_4Na^+$ 417.2036; Found 417.2037.

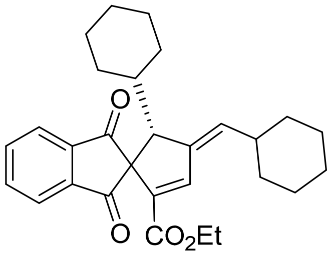

Synthesis of 3k (General procedure A): To an oven-dried 10 mL Schlenk tube equipped with a stir bar were added $Pd(TFA)_2$ (3.3 mg, 0.0099 mmol, 10 mol%), **L7** (3.0 mg, 0.012 mmol, 12 mol%), Et_3N (7.0 μL, 0.050 mmol, 50 mol%), ethyl 2-(2-((*tert*-butoxycarbonyl)oxy)-1,3-

dioxo-2,3-dihydro-1*H*-inden-2-yl)acrylate **1b** (36.0 mg, 0.0999 mmol, 1.0 equiv) and 1,3-dicyclohexylpropa-1,2-diene **2c** (40.9 mg, 0.200 mmol, 2.0 equiv). The tube was evacuated and back-filled with argon for five times. Then degassed DCM (1.0 mL) was added by syringe. The mixture was stirred at 40 °C for 24 h. After completion, the mixture was concentrated and purified by flash chromatography on silica gel (EtOAc/petroleum ether) to give product **3k**: as a colorless oil, 29.6 mg (0.0663 mmol), 66% yield; *E/Z* >19:1; $[\alpha]_D^{25}$ = –100.2 (*c* = 1.30, in $CHCl_3$); 93% ee, determined by HPLC analysis [Chiralpak column AD-H, *i*-PrOH/*n*-Hexane = 10/90, flow rate: 1.0 mL/min, 254 nm, t (minor) = 7.09 min, t (major) = 8.62 min]; ^{1}H NMR (400 MHz, $CDCl_3$) δ (ppm) 8.05–7.97 (m, 2H), 7.88–7.81 (m, 2H), 7.67 (s, 1H), 5.40 (d, *J* = 9.7 Hz, 1H), 4.05–3.92 (m, 2H), 3.12–2.94 (m, 1H), 2.55–2.40 (m, 1H), 1.86–1.69 (m, 6H), 1.65–1.52 (m, 4H), 1.51–1.44 (m, 1H), 1.39–1.30 (m, 3H), 1.24–1.08 (m, 6H), 1.01 (t, *J* = 7.1 Hz, 3H), 0.85–0.76 (m, 1H); ^{13}C NMR (100 MHz, $CDCl_3$) δ (ppm) 200.7, 199.6, 163.7, 144.3, 143.1, 141.2, 139.6, 136.8, 136.7, 135.5, 135.3, 123.6, 123.1, 68.6, 60.8, 58.7, 39.9, 39.5, 33.8, 33.1, 31.5, 29.5, 26.9, 26.5, 26.3, 25.9, 25.8, 25.7, 13.7; HRMS (ESI-TOF) *m/z*: $[M + Na]^+$ Calcd for $C_{29}H_{34}O_4Na^+$ 469.2349; Found 469.2351.

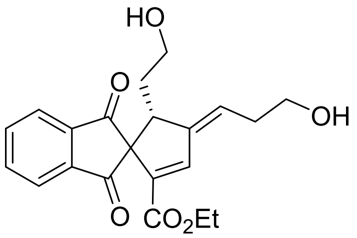

Synthesis of 3l (General procedure A): To an oven-dried 10 mL Schlenk tube equipped with a stir bar were added $Pd(TFA)_2$ (3.3 mg, 0.0099 mmol, 10 mol%), **L7** (3.0 mg, 0.012 mmol, 12 mol%), Et_3N (7.0 μL, 0.050 mmol, 50 mol%), ethyl 2-(2-((*tert*-butoxycarbonyl))-1,3-dioxo-2,3-dihydro-1*H*-inden-2-yl)acrylate **1b** (36.0 mg, 0.0999 mmol, 1.0 equiv) and hepta-3,4-diene-1,7-diol **2d** (25.6 mg, 0.200 mmol, 2.0 equiv). The tube was evacuated and back-filled with argon for five times. Then degassed DCM (1.0 mL) was added by syringe. The mixture was stirred at 40 °C for 48 h. After completion, the mixture was concentrated and purified by flash chromatography on silica gel (EtOAc/petroleum ether) to give product **3l**: as a yellow oil, 25.8 mg (0.0697 mmol), 70% yield; *E/Z* >19:1; $[\alpha]_D^{25}$ = +15.5 (*c* = 2.90, in $CHCl_3$); 87% ee, determined by HPLC analysis [Chiralpak column IC, *i*-PrOH/*n*-Hexane = 40/60, flow rate: 1.0 mL/min, 254 nm, t (minor) = 9.74 min, t (major) = 10.63 min]; ^{1}H NMR (400 MHz, $CDCl_3$) δ (ppm) 8.11–7.95 (m, 2H), 7.92–7.80 (m, 2H), 7.68 (s, 1H), 5.73–5.56 (m, 1H), 4.02–3.90 (q, *J* = 7.0 Hz, 2H), 3.73 (td, *J* = 6.2, 2.1 Hz, 2H), 3.58–3.48 (m, 1H), 3.43–3.33 (m, 1H), 3.32–3.20 (m, 1H), 2.66–2.49 (m, 2H), 2.21–1.59 (m, 4H), 0.97 (t, *J* = 7.0 Hz, 3H); ^{13}C NMR (100 MHz, $CDCl_3$) δ (ppm) 201.7, 199.8, 163.5, 146.5, 143.6, 142.23, 142.17, 137.6, 135.7, 135.6, 125.6, 123.3, 123.2, 67.9, 61.9, 61.0, 60.5, 48.2, 33.3, 32.6, 13.6; HRMS (ESI-TOF) *m/z*: $[M + Na]^+$ Calcd for $C_{21}H_{22}O_6Na^+$ 393.1309; Found

393.1308.

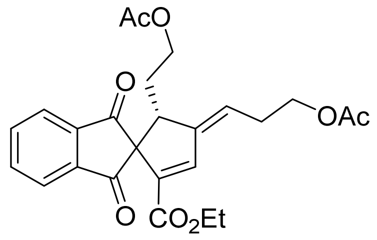

Synthesis of 3m (General procedure A): To an oven-dried 10 mL Schlenk tube equipped with a stir bar were added $Pd(TFA)_2$ (3.3 mg, 0.0099 mmol, 10 mol%), **L7** (3.0 mg, 0.012 mmol, 12 mol%), Et_3N (7.0 μL, 0.050 mmol, 50 mol%), ethyl 2-(2-((*tert*-butoxycarbonyl)oxy) -1,3-dioxo-2,3-dihydro-1*H*-inden-2-yl)acrylate **1b** (36.0 mg, 0.0999 mmol, 1.0 equiv) and hepta-3,4-diene-1,7-diyl diacetate **2e** (42.4 mg, 0.200 mmol, 2.0 equiv). The tube was evacuated and back-filled with argon for five times. Then degassed DCM (1.0 mL) was added by syringe. The mixture was stirred at 40 °C for 24 h. After completion, the mixture was concentrated and purified by flash chromatography on silica gel (EtOAc/petroleum ether) to give product **3m**: as a yellow oil, 30.3 mg (0.0667 mmol), 67% yield; *E/Z* >19:1; $[\alpha]_D^{25}$ = +18.3 (c = 0.30, in $CHCl_3$); 96% ee, determined by HPLC analysis [Chiralpak column IA, *i*-PrOH/*n*-Hexane = 20/80, flow rate: 1.0 mL/min, 254 nm, t (minor) = 14.43 min, t (major) = 15.58 min];^{1}H NMR (400 MHz, $CDCl_3$) δ (ppm) 8.08–7.96 (m, 2H), 7.92–7.82 (m, 2H), 7.64 (s, 1H), 5.62–5.46 (m, 1H), 4.22–4.14 (m, 2H), 3.97 (q, J = 7.2 Hz, 2H), 3.83–3.71 (m, 1H), 3.65–3.57 (m, 1H), 3.51–3.42 (m, 1H), 2.73–2.60 (m, 2H), 2.09 (s, 3H), 2.06–2.01 (m, 1H), 1.93–1.81 (m, 4H), 0.96 (t, J = 7.1 Hz, 3H); ^{13}C NMR (100 MHz, $CDCl_3$) δ (ppm) 201.1, 199.3, 171.1, 170.5, 163.3, 146.4, 142.9, 142.1, 138.0, 135.84, 135.78, 124.2, 123.4, 123.3, 67.7, 63.1, 61.9, 61.1, 47.7, 29.5, 28.9, 20.9, 20.6, 13.6; HRMS (ESI-TOF) *m/z*: $[M + Na]^+$ Calcd for $C_{25}H_{26}O_8Na^+$ 477.1520; Found 477.1522.

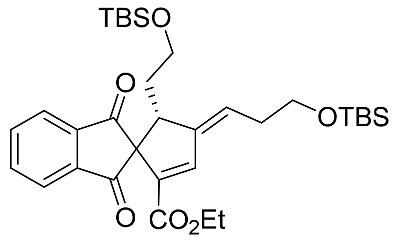

Synthesis of 3n (General procedure A): To an oven-dried 10 mL Schlenk tube equipped with a stir bar were added $Pd(TFA)_2$ (3.3 mg, 0.0099 mmol, 10 mol%), **L7** (3.0 mg, 0.012 mmol, 12 mol%), Et_3N (7.0 μL, 0.050 mmol, 50 mol%), ethyl 2-(2-((*tert*-butoxycarbonyl) oxy)-1,3-dioxo-2,3-dihydro-1*H*-inden-2-yl)acrylate **1b** (36.0 mg, 0.0999 mmol, 1.0 equiv) and 2,2,3,3,13,13,14,14-octamethyl-4,12-dioxa-3,13-disilapentadeca-7,8-diene **2f** (71.3 mg, 0.200 mmol, 2.0 equiv). The tube was evacuated and back-filled with argon for five times. Then degassed DCM (1.0 mL) was added by syringe. The mixture was stirred at 40 °C for 24 h. After completion, the mixture was concentrated and purified by flash chromatography on silica gel (EtOAc/petroleum ether) to give product **3n**: as a yellow oil, 55.2 mg (0.0922 mmol), 92% yield; *E/Z* >19:1; $[\alpha]_D^{25}$ = +24.9 (c = 2.13, in $CHCl_3$); 95% ee, determined by HPLC analysis [Chiralpak

column IC, *i*-PrOH/*n*-Hexane = 5/95, flow rate: 1.0 mL/min, 254 nm, t (minor) = 7.53 min, t (major) = 9.07 min]; ^{1}H NMR (400 MHz, $CDCl_3$) δ (ppm) 8.04–7.93 (m, 2H), 7.87–7.78 (m, 2H), 7.66 (s, 1H), 5.60 (t, J = 7.9 Hz, 1H), 3.94 (q, J = 7.1 Hz, 2H), 3.69 (t, J = 6.6 Hz, 2H), 3.57–3.48 (m, 1H), 3.33–3.21 (m, 1H), 3.11–2.98 (m, 1H), 2.51 (q, J = 7.1 Hz, 2H), 1.94–1.70 (m, 2H), 0.98–0.87 (m, 12H), 0.71 (s, 9H), 0.06 (s, 6H), –0.21 (s, 3H), –0.30 (s, 3H); ^{13}C NMR (100 MHz, $CDCl_3$) δ (ppm) 201.4, 199.8, 163.6, 146.2, 143.9, 142.3, 142.1, 137.2, 135.42, 135.39, 125.7, 123.4, 123.0, 67.9, 62.6, 60.8, 60.7, 47.5, 33.6, 33.5, 25.9, 25.7, 18.3, 18.0, 13.6, –5.3, –5.7, –5.8; HRMS (ESI-TOF) *m/z*: [M + Na]$^{+}$ Calcd for $C_{33}H_{50}O_6Si_2Na^{+}$ 621.3038; Found 621.3039.

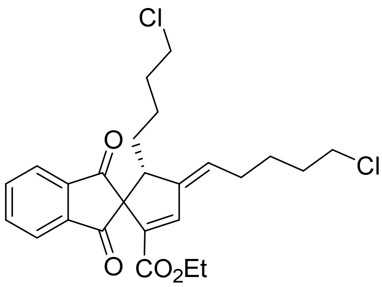

Synthesis of 3o (General procedure A): To an oven-dried 10 mL Schlenk tube equipped with a stir bar were added $Pd(TFA)_2$ (3.3 mg, 0.0099 mmol, 10 mol%), **L7** (3.0 mg, 0.012 mmol, 12 mol%), Et_3N (7.0 μL, 0.050 mmol, 50 mol%), ethyl 2-(2-((*tert*-butoxycarbonyl) oxy)-1,3-dioxo-2,3-dihydro-1*H*-inden-2-yl)acrylate **1b** (36.0 mg, 0.0999 mmol, 1.0 equiv) and 1,11-dichloroundeca-5,6-diene **2g** (44.2 mg, 0.200 mmol, 2.0 equiv). The tube was evacuated and back-filled with argon for five times. Then degassed DCM (1.0 mL) was added by syringe. The mixture was stirred at 40 °C for 48 h. After completion, the mixture was concentrated and purified by flash chromatography on silica gel (EtOAc/petroleum ether) to give product **3o**: as a yellow oil, 19.4 mg (0.0419 mmol), 42% yield; *E/Z* >19:1; $[\alpha]_D^{25}$ = –74.4 (c = 0.50, in MeCN); 86% ee, determined by HPLC analysis [Chiralpak column ID, *i*-PrOH/*n*-Hexane = 20/80, flow rate: 1.0 mL/min, 254 nm, t (major) = 18.56 min, t (minor) = 20.87 min]; ^{1}H NMR (400 MHz, $CDCl_3$) δ (ppm) 8.09–7.93 (m, 2H), 7.93–7.76 (m, 2H), 7.64 (s, 1H), 5.53 (t, J = 7.5 Hz, 1H), 4.01–3.87 (m, 2H), 3.63–3.53 (m, 2H), 3.37–3.30 (m, 1H), 3.27 (t, J = 6.4 Hz, 2H), 2.35 (q, J = 7.3 Hz, 2H), 1.88–1.78 (m, 2H), 1.68–1.49 (m, 6H), 1.19–1.05 (m, 1H), 0.99–0.83 (m, 4H).; ^{13}C NMR (100 MHz, $CDCl_3$) δ (ppm) 201.9, 199.4, 163.5, 144.6, 143.4, 142.3, 142.0, 137.1, 135.6, 128.7, 123.3, 123.1, 68.0, 60.9, 51.8, 44.8, 44.3, 32.1, 32.0, 29.4, 29.2, 26.8, 25.5, 13.5; HRMS (ESI-TOF) *m/z*: [M + H]$^{+}$ Calcd for $C_{25}H_{29}{}^{35}Cl_2O_4^{+}$ 463.1437; Found 463.1434; Calcd for $C_{25}H_{29}{}^{35}Cl^{37}ClO_4^{+}$ 465.1408; Found 465.1431.

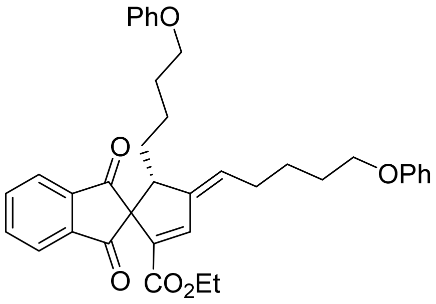

Synthesis of 3p (General procedure A): To an oven-dried 10 mL Schlenk tube equipped with a stir bar were added Pd(TFA)$_2$ (3.3 mg, 0.0099 mmol, 10 mol%), **L7** (3.0 mg, 0.012 mmol, 12 mol%), Et$_3$N (7.0 μL, 0.050 mmol, 50 mol%), ethyl 2-(2-((*tert*-butoxycarbonyl)oxy)-1,3-dioxo-2,3-dihydro-1*H*-inden-2-yl) acrylate **1b** (36.0 mg, 0.0999 mmol, 1.0 equiv) and 1,11-diphenoxyundeca-5,6-diene **2h** (67.3 mg, 0.200 mmol, 2.0 equiv). The tube was evacuated and back-filled with argon for five times. Then degassed DCM (1.0 mL) was added by syringe. The mixture was stirred at 40 °C for 48 h. After completion, the mixture was concentrated and purified by flash chromatography on silica gel (EtOAc/petroleum ether) to give product **3p**: as a yellow oil, 40.5 mg (0.0699 mmol), 70% yield; *E/Z* >19:1; $[\alpha]_D^{25}$ = –84.0 (*c* = 0.40, in MeCN); 78% ee, determined by HPLC analysis [Chiralpak column ID, *i*-PrOH/*n*-Hexane = 40/60, flow rate: 1.0 mL/min, 254 nm, t (minor) = 16.15 min, t (major) = 19.62 min]; ^{1}H NMR (400 MHz, CDCl$_3$) δ (ppm) 8.03–7.98 (m, 1H), 7.97–7.92 (m, 1H), 7.85–7.76 (m, 2H), 7.67 (s, 1H), 7.30–7.20 (m, 4H), 6.96–6.88 (m, 4H), 6.74–6.66 (m, 2H), 5.64–5.49 (m, 1H), 4.01–3.92 (m, 4H), 3.67 (td, *J* = 6.2, 1.8 Hz, 2H), 3.42–3.28 (m, 1H), 2.44–2.32 (m, 2H), 1.88–1.80 (m, 2H), 1.72–1.61 (m, 4H), 1.58–1.48 (m, 2H), 1.21–1.10 (m, 1H), 1.00–0.89 (m, 4H); ^{13}C NMR (100 MHz, CDCl$_3$) δ (ppm) 202.0, 199.5, 163.6, 159.0, 158.8, 144.6, 143.6, 142.3, 142.1, 136.9, 135.51, 135.48, 129.4, 129.3, 129.2, 123.3, 123.0, 120.51, 120.46, 114.5, 114.3, 68.1, 67.4, 66.9, 60.9, 51.9, 30.0, 29.7, 29.0, 28.8, 26.2, 25.0, 13.6; HRMS (ESI-TOF) *m/z*: [M + Na]$^+$ Calcd for $C_{37}H_{38}O_6Na^+$ 601.2561; Found 601.2563.

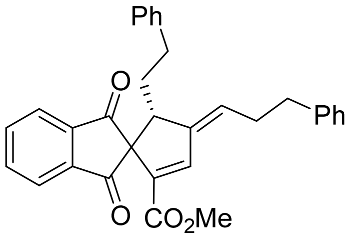

Synthesis of 3q (General procedure A): To an oven-dried 10 mL Schlenk tube equipped with a stir bar were added Pd(TFA)$_2$ (3.3 mg, 0.0099 mmol, 10 mol%), **L7** (3.0 mg, 0.012 mmol, 12 mol%), Et$_3$N (7.0 μL, 0.050 mmol, 50 mol%), methyl 2-(2-((*tert*-butoxycarbonyl) oxy)-1,3-dioxo-2,3-dihydro-1*H*-inden-2-yl)acrylate **1a** (34.6 mg, 0.0999 mmol, 1.0 equiv) and 1,7-diphenylhepta-3,4-diene **2i** (49.7 mg, 0.200 mmol, 2.0 equiv). The tube was evacuated and back-filled with argon for five times. Then degassed DCM (1.0 mL) was added by syringe. The mixture was stirred at 40 °C for 24 h. After completion, the mixture was concentrated and purified by flash chromatography on silica gel (EtOAc/petroleum ether) to give product **3q**: as a white solid, 40.7 mg (0.0854 mmol), 85% yield; mp 126–128 °C; *E/Z* >19:1; $[\alpha]_D^{25}$ = –36.9 (*c* = 0.59, in CHCl$_3$); 90% ee, determined by HPLC analysis [Chiralpak column AD-H, *i*-PrOH/*n*-Hexane =

40/60, flow rate: 1.0 mL/min, 254 nm, t (minor) = 13.90 min, t (major) = 19.45 min]; ^{1}H NMR (400 MHz, $CDCl_3$) δ (ppm) 8.09–7.99 (m, 2H), 7.90–7.82 (m, 2H), 7.57 (s, 1H), 7.34–7.26 (m, 2H), 7.23–7.14 (m, 5H), 7.13–7.08 (m, 1H), 6.88–6.79 (m, 2H), 5.57 (t, J = 7.6 Hz, 1H), 3.54 (s, 3H), 3.40–3.29 (m, 1H), 2.77–2.67 (m, 2H), 2.64–2.54 (m, 2H), 2.25–2.15 (m, 1H), 2.12–2.02 (m, 1H), 1.95–1.84 (m, 2H); ^{13}C NMR (100 MHz, $CDCl_3$) δ (ppm) 201.7, 199.3, 164.2, 144.8, 143.4, 142.3, 141.9, 141.0, 140.9, 136.7, 135.7, 135.6, 128.8, 128.38, 128.35, 128.3, 128.0, 126.0, 125.9, 123.4, 123.0, 68.1, 51.8, 51.3, 35.8, 34.4, 32.5, 31.7; HRMS (ESI-TOF) *m/z*: $[M + Na]^+$ Calcd for $C_{32}H_{28}O_4Na^+$ 499.1880; Found 499.1879.

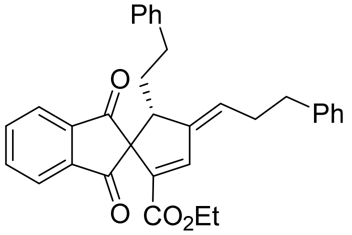

Synthesis of 3r (General procedure A): To an oven-dried 10 mL Schlenk tube equipped with a stir bar were added $Pd(TFA)_2$ (3.3 mg, 0.0099 mmol, 10 mol%), **L7** (3.0 mg, 0.012 mmol, 12 mol%), Et_3N (7.0 μL, 0.050 mmol, 50 mol%), ethyl 2-(2-((*tert*-butoxycarbonyl) oxy)-1,3-dioxo-2,3-dihydro-1*H*-inden-2-yl)acrylate **1b** (36.0 mg, 0.0999 mmol, 1.0 equiv) and 1,7-diphenyl hepta-3,4-diene **2i** (49.7 mg, 0.200 mmol, 2.0 equiv). The tube was evacuated and back-filled with argon for five times. Then degassed DCM (1.0 mL) was added by syringe. The mixture was stirred at 40 °C for 24 h. After completion, the mixture was concentrated and purified by flash chromatography on silica gel (EtOAc/petroleum ether) to give product **3r**: as a white solid, 45.4 mg (0.0925 mmol), 93% yield; mp 90–92 °C; *E/Z* >19:1; $[\alpha]_D^{25}$ = –30.0 (c = 1.34, in $CHCl_3$); 91% ee, determined by HPLC analysis [Chiralpak column OD-H, *i*-PrOH/*n*-Hexane = 5/95, flow rate: 1.0 mL/min, 254 nm, t (major) = 18.53 min, t (minor) = 23.12 min]; ^{1}H NMR (400 MHz, $CDCl_3$) δ (ppm) 8.09–8.00 (m, 2H), 7.91–7.82 (m, 2H), 7.59 (s, 1H), 7.34–7.26 (m, 2H), 7.23–7.15 (m, 5H), 7.14–7.09 (m, 1H), 6.84 (d, J = 6.9 Hz, 2H), 5.57 (t, J = 7.1 Hz, 1H), 3.96 (q, J = 7.1 Hz, 2H), 3.37 (t, J = 6.4 Hz, 1H), 2.79–2.70 (m, 2H), 2.65–2.56 (m, 2H), 2.25–2.15 (m, 1H), 2.13–2.03 (m, 1H), 1.96–1.86 (m, 2H), 0.96 (t, J = 7.1 Hz, 3H); ^{13}C NMR (100 MHz, $CDCl_3$) δ (ppm) 201.9, 199.5, 163.6, 144.7, 143.5, 142.4, 142.0, 141.1, 140.9, 137.0, 135.7, 135.6, 128.7, 128.41, 128.39, 128.3, 128.0, 126.03, 125.98, 123.4, 123.0, 68.0, 60.9, 51.4, 35.9, 34.5, 32.5, 31.8, 13.6; HRMS (ESI-TOF) *m/z*: $[M + Na]^+$ Calcd for $C_{33}H_{30}O_4Na^+$ 513.2036; Found 513.2036.

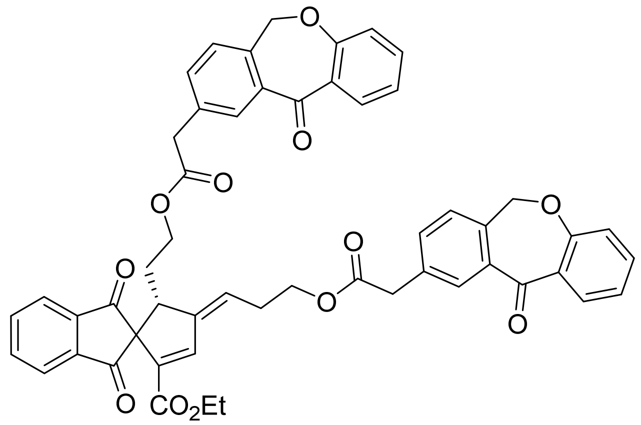

Synthesis of 3s (General procedure A): To an oven-dried 10 mL Schlenk tube equipped with a stir bar were added Pd(TFA)$_2$ (3.3 mg, 0.0099 mmol, 10 mol%), **L7** (3.0 mg, 0.012 mmol, 12 mol%), Et$_3$N (7.0 μL, 0.050 mmol, 50 mol%), ethyl 2-(2-((*tert*-butoxycarbonyl)oxy)-1,3-dioxo-2,3-dihydro-1*H*-inden-2-yl)acrylate **1b** (36.0 mg, 0.0999 mmol, 1.0 equiv) and hepta-3,4-diene-1,7-diyl bis(2-(11-oxo-6,11-dihydrodibenzo[*b*,*e*]oxepin-9-yl)acetate) **2j** (125.7 mg, 0.2000 mmol, 2.0 equiv). The tube was evacuated and back-filled with argon for five times. Then degassed DCM (1.0 mL) was added by syringe. The mixture was stirred at 40 °C for 24 h. After completion, the mixture was concentrated and purified by flash chromatography on silica gel (EtOAc/petroleum ether) to give product **3s**: as a yellow oil, 81.9 mg (0.0941 mmol), 94% yield; *E/Z* >19:1; $[\alpha]_D^{25}$ = +9.1 (*c* = 1.91, in CHCl$_3$); 94% ee, determined by HPLC analysis [Chiralpak column IA, *i*-PrOH/*n*-Hexane = 40/60, flow rate: 1.0 mL/min, 254 nm, t (major) = 93.83 min, t (minor) = 136.17 min]; ^{1}H NMR (400 MHz, CDCl$_3$) δ (ppm) 8.13 (d, *J* = 2.4 Hz, 1H), 8.05–7.95 (m, 3H), 7.92–7.82 (m, 4H), 7.61 (s, 1H), 7.58–7.51 (m, 2H), 7.49–7.42 (m, 3H), 7.38–7.30 (m, 3H), 7.07–6.95 (m, 2H), 5.56–5.42 (m, 1H), 5.18 (s, 4H), 4.27–4.11 (m, 2H), 3.96 (q, *J* = 7.1 Hz, 2H), 3.87–3.76 (m, 1H), 3.69 (s, 2H), 3.66–3.57 (m, 1H), 3.52–3.31 (m, 3H), 2.69–2.54 (m, 2H), 2.07–1.95 (m, 1H), 1.92–1.78 (m, 1H), 0.95 (t, *J* = 7.1 Hz, 3H); ^{13}C NMR (100 MHz, CDCl$_3$) δ (ppm) 200.9, 199.2, 190.8, 190.7, 171.4, 170.8, 163.3, 160.44, 160.43, 146.4, 142.9, 142.1, 142.0, 140.43, 140.40, 138.0, 136.4, 136.2, 135.9, 135.8, 135.5, 132.74, 132.70, 132.5, 132.4, 129.5, 129.22, 129.21, 127.78, 127.76, 127.4, 125.10, 125.07, 124.2, 123.5, 123.3, 121.1, 121.0, 73.6, 67.6, 63.6, 62.3, 61.1, 47.5, 40.1, 39.7, 29.5, 29.0, 13.6; HRMS (ESI-TOF) *m/z*: $[M + Na]^+$ Calcd for $C_{53}H_{42}O_{12}Na^+$ 893.2568; Found 893.2568.

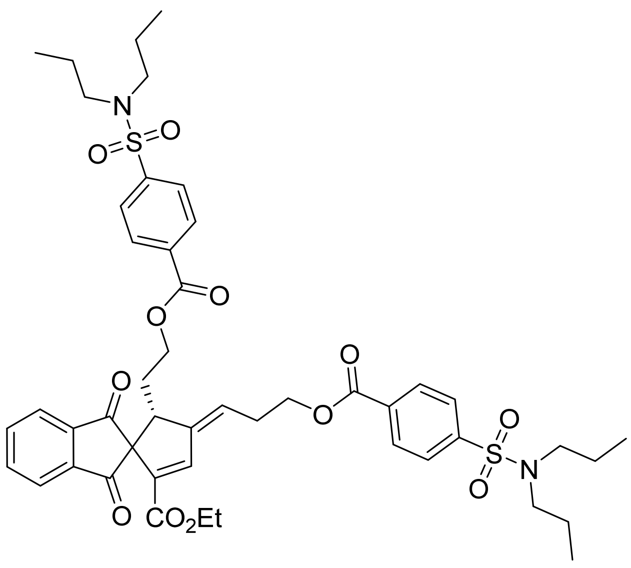

Synthesis of 3t (General procedure A): To an oven-dried 10 mL Schlenk tube equipped with a stir bar were added Pd(TFA)$_2$ (3.3 mg, 0.0099 mmol, 10 mol%), **L7** (3.0 mg, 0.012 mmol, 12 mol%), Et$_3$N (7.0 μL, 0.050 mmol, 50 mol%), ethyl 2-(2-((*tert*-butoxycarbonyl)oxy)-1,3-dioxo-2,3-dihydro-1*H*-inden-2-yl)acrylate **1b** (36.0 mg, 0.0999 mmol, 1.0 equiv) and hepta-3,4-diene-1,7-diyl bis(4-(*N*,*N*-dipropylsulfamoyl)benzoate) **2k** (132.6 mg, 0.2000 mmol, 2.0 equiv). The tube was evacuated and back-filled with argon for five times. Then degassed DCM (1.0 mL) was added by syringe. The mixture was stirred at 40 °C for 24 h. After completion, the mixture was concentrated and purified by flash chromatography on silica gel (EtOAc/petroleum ether) to give product **3t**: as a yellow oil, 85.9 mg (0.0949 mmol), 95% yield; *E/Z* >19:1; $[\alpha]_D^{25}$ = +25.7 (*c* = 1.04, in CHCl$_3$); 93% ee, determined by HPLC analysis [Chiralpak column IA, *i*-PrOH/*n*-Hexane = 40/60, flow rate: 1.0 mL/min, 254 nm, t (minor) = 25.69 min, t (major) = 28.98 min]; ^{1}H NMR (400 MHz, CDCl$_3$) δ (ppm) 8.23–8.15 (m, 2H), 8.05–7.97 (m, 3H), 7.94–7.83 (m, 7H), 7.70 (s, 1H), 5.73–5.57 (m, 1H), 4.51–4.40 (m, 2H), 4.19–4.08 (m, 1H), 3.96 (q, *J* = 7.1 Hz, 2H), 3.88–3.78 (m, 1H), 3.66–3.52 (m, 1H), 3.13–3.07 (m, 8H), 2.90–2.74 (m, 2H), 2.30–2.14 (m, 1H), 2.05–1.93 (m, 1H), 1.60–1.51 (m, 8H), 0.94 (t, *J* = 7.1 Hz, 3H), 0.87 (t, *J* = 7.4 Hz, 12H); ^{13}C NMR (100 MHz, CDCl$_3$) δ (ppm) 201.0, 199.0, 165.2, 164.6, 163.1, 146.7, 144.4, 144.2, 142.8, 142.0, 141.9, 138.3, 135.95, 135.92, 133.2, 132.9, 130.3, 130.1, 127.0, 126.9, 123.7, 123.4, 123.3, 67.6, 64.2, 62.8, 61.2, 49.92, 49.89, 47.5, 29.4, 29.0, 21.91, 21.90, 13.5, 11.1; HRMS (ESI-TOF) *m/z*: $[M + Na]^+$ Calcd for $C_{47}H_{56}N_2O_{12}S_2Na^+$ 927.3167; Found 927.3167.

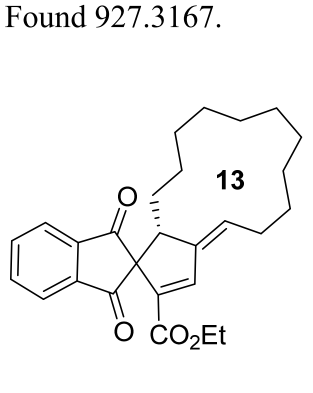

Synthesis of 3u (General procedure A): To an oven-dried 10 mL Schlenk tube equipped with a stir bar were added Pd(TFA)$_2$ (3.3 mg, 0.0099 mmol, 10 mol%), **L7** (3.0 mg, 0.012 mmol, 12 mol%), Et$_3$N (7.0 μL, 0.050 mmol, 50 mol%), ethyl 2-(2-((*tert*-butoxycarbonyl)oxy)-1,3-dioxo-2,3-dihydro-1*H*-inden-2-yl)acrylate **1b** (36.0 mg, 0.0999 mmol, 1.0 equiv) and cyclo-trideca-1,2-diene **2l** (35.6 mg, 0.200 mmol, 2.0 equiv). The tube was evacuated and back-filled with

argon for five times. Then degassed DCM (1.0 mL) was added by syringe. The mixture was stirred at 40 °C for 24 h. After completion, the mixture was concentrated and purified by flash chromatography on silica gel (EtOAc/petroleum ether) to give product **3u**: as a yellow oil, 28.1 mg (0.0668 mmol), 67% yield; *E/Z* >19:1; $[\alpha]_D^{25}$ = –43.5 (*c* = 0.63, in $CHCl_3$); 72% ee, determined by HPLC analysis [Chiralpak column AD-H, *i*-PrOH/*n*-Hexane = 20/80, flow rate: 1.0 mL/min, 254 nm, t (minor) = 6.82 min, t (major) = 8.03 min]; ^{1}H NMR (400 MHz, $CDCl_3$) δ (ppm) 8.08–7.96 (m, 2H), 7.90–7.80 (m, 2H), 7.67 (s, 1H), 5.65 (dd, *J* = 10.6, 5.4 Hz, 1H), 4.09–3.93 (m, 2H), 3.13–3.00 (m, 1H), 2.46–2.34 (m, 1H), 2.34–2.22 (m, 1H), 1.61–1.43 (m, 5H), 1.37–1.20 (m, 13H), 1.04 (t, *J* = 7.1 Hz, 3H); ^{13}C NMR (100 MHz, $CDCl_3$) δ (ppm) 200.4, 199.2, 163.8, 144.1, 143.7, 142.9, 141.5, 136.5, 135.6, 135.4, 131.4, 123.7, 123.2, 68.8, 60.9, 52.1, 31.1, 29.6, 27.7, 27.1, 26.8, 25.1, 25.0, 24.9, 24.4, 22.4, 13.7; HRMS (ESI-TOF) *m/z*: $[M + Na]^+$ Calcd for $C_{27}H_{32}O_4Na^+$ 443.2193; Found 443.2194.

4.2 General procedure for (3+2) annulations of MBH carbonates with 1,3-diaryl-substituted allenes

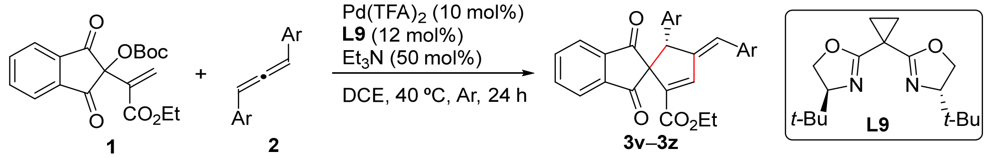

General procedure B: To an oven-dried 10 mL Schlenk tube equipped with a stir bar were added $Pd(TFA)_2$ (10 mol%), **L9** (12 mol%), Et_3N (50 mol%), MBH carbonate **1** (0.10 mmol, 1.0 equiv) and aryl allene **2** (0.20 mmol, 2.0 equiv). The tube was evacuated and back-filled with argon for five times. Then degassed DCE (1.0 mL) was added by syringe. The mixture was stirred at 40 °C for 24 h. After completion, the mixture was concentrated and purified by flash chromatography on silica gel (EtOAc/petroleum ether) to give products **3v–3z**.

Synthesis of racemic 3v–3z: *Rac*-**3v–3z** were obtained under the catalysis of $Pd(TFA)_2$, (±)-**L7** and Et_3N.

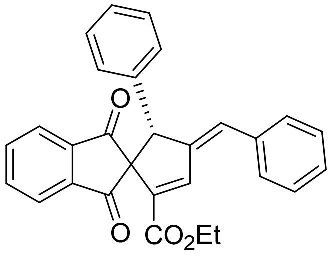

Synthesis of 3v (General procedure B): To an oven-dried 10 mL Schlenk tube equipped with a stir bar were added $Pd(TFA)_2$ (3.3 mg, 0.0099 mmol, 10 mol%), **L9** (3.6 mg, 0.012 mmol, 12 mol%), Et_3N (7.0 μL, 0.050 mmol, 50 mol%), ethyl 2-(2-((*tert*-butoxycarbonyl)oxy)-1,3-dioxo-2,3-dihydro-1*H*-inden-2-yl)acrylate **1b** (36.0 mg, 0.0999 mmol, 1.0 equiv) and 1,3-diphenylpropa-1,2-diene **2m** (38.4 mg, 0.200 mmol, 2.0 equiv). The tube was

evacuated and back-filled with argon for five times. Then degassed DCE (1.0 mL) was added by syringe. The mixture was stirred at 40 °C for 24 h. After completion, the mixture was concentrated and purified by flash chromatography on silica gel (EtOAc/petroleum ether) to give product **3v**: as a yellow oil, 43.4 mg (0.0878 mmol), 88% yield; mp 146–148 °C; *E/Z* >19:1;$[\alpha]_D^{25}$ = +15.8 (*c* = 1.81, in $CHCl_3$); 90% ee, determined by HPLC analysis [Chiralpak column IA, *i*-PrOH/*n*-Hexane = 10/90, flow rate: 1.0 mL/min, 254 nm, t (minor) = 12.42 min, t (major) = 17.42 min]; ^{1}H NMR (400 MHz, $CDCl_3$) δ (ppm) 8.06 (s, 1H), 8.00 (d, *J* = 7.5 Hz, 1H), 7.75 (td, *J* = 7.4, 1.3 Hz, 1H), 7.66 (td, *J* = 7.4, 1.1 Hz, 1H), 7.61 (d, *J* = 7.6 Hz, 1H), 7.39 (d, *J* = 4.4 Hz, 4H), 7.34–7.27 (m, 1H), 7.17–7.02 (m, 5H), 6.41 (d, *J* = 2.6 Hz, 1H), 4.81 (d, *J* = 2.6 Hz, 1H), 4.01 (q, *J* = 7.1 Hz, 2H), 0.98 (t, *J* = 7.1 Hz, 3H); ^{13}C NMR (100 MHz, $CDCl_3$) δ (ppm) 201.5, 198.3, 163.4, 144.6, 144.5, 142.3, 142.2, 138.2, 136.9, 135.9, 135.5, 135.2, 130.2, 130.1, 128.6, 128.5, 128.2, 127.8, 127.6, 123.1, 123.0, 70.8, 61.1, 59.9, 13.6; HRMS (ESI-TOF) *m/z*: $[M + Na]^+$ Calcd for $C_{29}H_{22}O_4Na^+$ 457.1410; Found 457.1407.

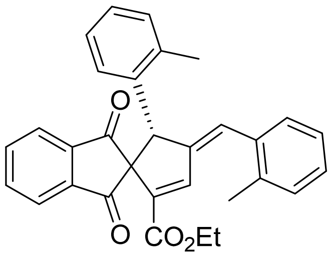

Synthesis of 3w (General procedure B): To an oven-dried 10 mL Schlenk tube equipped with a stir bar were added $Pd(TFA)_2$ (3.3 mg, 0.0099 mmol, 10 mol%), **L9** (3.6 mg, 0.012 mmol, 12 mol%), Et_3N (7.0 μL, 0.050 mmol, 50 mol%), ethyl 2-(2-((*tert*-butoxycarbonyl)oxy)-1,3-dioxo-2,3-dihydro-1*H*-inden-2-yl)acrylate **1b** (36.0 mg, 0.0999 mmol, 1.0 equiv) and 1,3-di-*o*-tolylpropa-1,2-diene **2n** (44.0 mg, 0.200 mmol, 2.0 equiv). The tube was evacuated and back-filled with argon for five times. Then degassed DCE (1.0 mL) was added by syringe. The mixture was stirred at 40 °C for 24 h. After completion, the mixture was concentrated and purified by flash chromatography on silica gel (EtOAc/petroleum ether) to give product **3w**: as a yellow oil, 23.0 mg (0.0497 mmol), 50% yield; *E/Z* >19:1; $[\alpha]_D^{25}$ = –59.7 (*c* = 0.32, in $CHCl_3$); 79% ee, determined by HPLC analysis [Chiralpak column IA, *i*-PrOH/*n*-Hexane = 10/90, flow rate: 1.0 mL/min, 254 nm, t (minor) = 8.52 min, t (major) = 9.41 min]; ^{1}H NMR (400 MHz, $CDCl_3$) δ (ppm) 8.02 (d, *J* = 7.6 Hz, 1H), 7.88 (s, 1H), 7.78 (t, *J* = 7.4 Hz, 1H), 7.70 (t, *J* = 7.4 Hz, 1H), 7.63 (d, *J* = 7.6 Hz, 1H), 7.44 (d, *J* = 7.3 Hz, 1H), 7.37 (d, *J* = 7.8 Hz, 1H), 7.28–7.23 (m, 2H), 7.20–7.11 (m, 2H), 7.02 (t, *J* = 7.4 Hz, 1H), 6.86 (d, *J* = 7.6 Hz, 1H), 6.38 (s, 1H), 5.12 (d, *J* = 2.5 Hz, 1H), 4.00 (q, *J* = 7.1 Hz, 2H), 2.20 (s, 3H), 1.94 (s, 3H), 0.98 (t, *J* = 7.1 Hz, 3H); ^{13}C NMR (100 MHz, $CDCl_3$) δ (ppm) 202.1, 198.3, 163.6, 146.7, 145.3, 142.2, 142.0, 138.1, 137.1, 136.6, 136.3, 135.7, 135.3, 134.9, 131.6, 130.1, 129.83, 129.82, 129.2, 128.0, 127.4, 126.0, 125.9, 123.1, 122.8, 70.5, 61.1, 54.5, 19.9, 19.8, 13.7; HRMS (ESI-TOF) *m/z*: $[M + Na]^+$ Calcd for $C_{31}H_{26}O_4Na^+$ 485.1723; Found 485.1724.

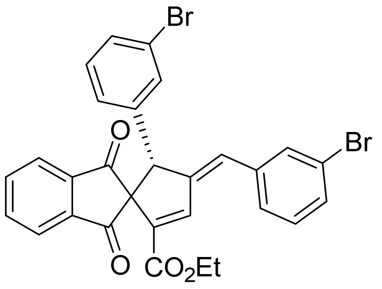

Synthesis of 3x (General procedure B): To an oven-dried 10 mL Schlenk tube equipped with a stir bar were added $Pd(TFA)_2$ (3.3 mg, 0.0099 mmol, 10 mol%), **L9** (3.6 mg, 0.012 mmol, 12 mol%), Et_3N (7.0 μL, 0.050 mmol, 50 mol%), ethyl 2-(2-((*tert*-butoxycarbonyl) oxy)-1,3-dioxo-2,3-dihydro-1*H*-inden-2-yl)acrylate **1b** (36.0 mg, 0.0999 mmol, 1.0 equiv) and 1,3-bis(3-bromophenyl)propa-1,2-diene **2o** (70.0 mg, 0.200 mmol, 2.0 equiv). The tube was evacuated and back-filled with argon for five times. Then degassed DCE (1.0 mL) was added by syringe. The mixture was stirred at 40 °C for 24 h. After completion, the mixture was concentrated and purified by flash chromatography on silica gel (EtOAc/petroleum ether) to give product **3x**: as a yellow oil, 40.0 mg (0.0675 mmol), 68% yield; *E/Z* >19:1; $[\alpha]_D^{25}$ = +17.4 (c = 0.84, in $CHCl_3$); 84% ee, determined by HPLC analysis [Chiralpak column IA, *i*-PrOH/*n*-Hexane = 10/90, flow rate: 1.0 mL/min, 254 nm, t (minor) = 11.77 min, t (major) = 15.00 min]; ^{1}H NMR (400 MHz, $CDCl_3$) δ (ppm) 8.02 (d, J = 7.6 Hz, 1H), 7.96 (s, 1H), 7.81 (t, J = 7.4 Hz, 1H), 7.77–7.64 (m, 2H), 7.50 (s, 1H), 7.45 (d, J = 7.8 Hz, 1H), 7.34 (d, J = 7.7 Hz, 1H), 7.30–7.25 (m, 2H), 7.20 (s, 1H), 7.09–6.96 (m, 2H), 6.29 (s, 1H), 4.73 (s, 1H), 4.02 (q, J = 6.9 Hz, 2H), 0.99 (t, J = 6.9 Hz, 3H); ^{13}C NMR (100 MHz, $CDCl_3$) δ (ppm) 200.9, 197.8, 163.1, 145.6, 143.6, 142.2, 142.1, 139.3, 138.7, 138.2, 135.8, 135.6, 133.1, 131.4, 131.0, 130.9, 130.2, 129.9, 128.8, 128.5, 127.0, 123.3, 123.2, 122.8, 122.3, 70.5, 61.3, 59.0, 13.6; HRMS (ESI-TOF) *m/z*: $[M + Na]^+$ Calcd for $C_{29}H_{20}{}^{79}Br_2O_4Na^+$ 612.9621; Found 612.9627; Calcd for $C_{29}H_{20}{}^{79}Br^{81}BrO_4Na^+$ 614.9600; Found 614.9595.

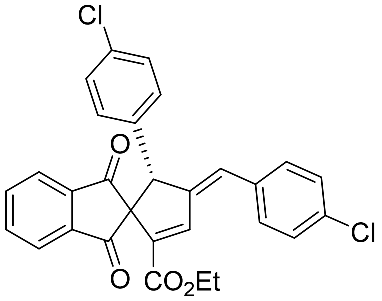

Synthesis of 3y (General procedure B): To an oven-dried 10 mL Schlenk tube equipped with a stir bar were added $Pd(TFA)_2$ (3.3 mg, 0.0099 mmol, 10 mol%), **L9** (3.6 mg, 0.012 mmol, 12 mol%), Et_3N (7.0 μL, 0.050 mmol, 50 mol%), ethyl 2-(2-((*tert*-butoxycarbonyl) oxy)-1,3-dioxo-2,3-dihydro-1*H*-inden-2-yl)acrylate **1b** (36.0 mg, 0.0999 mmol, 1.0 equiv) and 1,3-bis(4-chlorophenyl)propa-1,2-diene **2p** (52.2 mg, 0.200 mmol, 2.0 equiv). The tube was evacuated and back-filled with argon for five times. Then degassed DCE (1.0 mL) was added by syringe. The mixture was stirred at 40 °C for 24 h. After completion, the mixture was concentrated and purified by flash chromatography on silica gel (EtOAc/petroleum ether) to give product **3y**: as a yellow oil, 34.7 mg (0.0691 mmol), 69% yield; mp 124–126 °C; *E/Z* >19:1; $[\alpha]_D^{25}$ = +79.1 (c = 1.27, in $CHCl_3$); 87% ee, determined by HPLC analysis [Chiralpak column IA, *i*-PrOH/*n*-Hexane = 10/90, flow rate: 1.0 mL/min, 254 nm, t (minor) = 20.49

min, t (major) = 25.16 min]; ^{1}H NMR (400 MHz, $CDCl_3$) δ (ppm) 8.03–7.97 (m, 1H), 7.96 (s, 1H), 7.83–7.75 (m, 1H), 7.75–7.70 (m, 1H), 7.70–7.65 (m, 1H), 7.39–7.34 (m, 2H), 7.33–7.28 (m, 2H), 7.11 (d, *J* = 8.8 Hz, 2H), 7.02 (d, *J* = 7.8 Hz, 2H), 6.28 (d, *J* = 2.6 Hz, 1H), 4.76 (d, *J* = 2.6 Hz, 1H), 4.01 (q, J = 7.1 Hz, 2H), 0.98 (t, *J* = 7.1 Hz, 3H); ^{13}C NMR (100 MHz, $CDCl_3$) δ (ppm) 201.1, 198.1, 163.2, 145.1, 143.8, 142.2, 142.1, 138.9, 135.8, 135.6, 135.2, 134.4, 133.8, 133.7, 131.6, 129.7, 128.9, 128.7, 128.5, 123.3, 123.1, 70.5, 61.3, 59.0, 13.6; HRMS (ESI-TOF) *m/z*: [M + Na]$^+$ Calcd for $C_{29}H_{20}{}^{35}Cl_2O_4Na^+$ 525.0631; Found 525.0628; Calcd for $C_{29}H_{20}{}^{35}Cl^{37}ClO_4Na^+$ 527.0601; Found 527.0597.

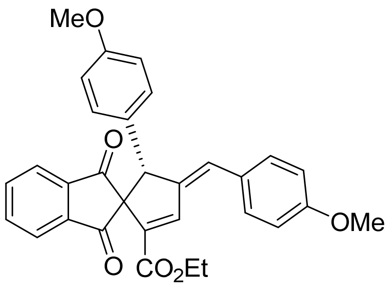

Synthesis of 3z (General procedure B): To an oven-dried 10 mL Schlenk tube equipped with a stir bar were added $Pd(TFA)_2$ (3.3 mg, 0.0099 mmol, 10 mol%), **L8** (3.6 mg, 0.012 mmol, 12 mol%), Et_3N (7.0 μL, 0.050 mmol, 50 mol%), ethyl 2-(2-((*tert*-butoxy carbonyl) oxy)-1,3-dioxo-2,3-dihydro-1*H*-inden-2-yl)acrylate **1b** (36.0 mg, 0.0999 mmol, 1.0 equiv) and 1,3-bis(4-methoxyphenyl) propa-1,2-diene **2q** (50.4 mg, 0.200 mmol, 2.0 equiv). The tube was evacuated and back-filled with argon for five times. Then degassed DCE (1.0 mL) was added by syringe. The mixture was stirred at 40 °C for 24 h. After completion, the mixture was concentrated and purified by flash chromatography on silica gel (EtOAc/petroleum ether) to give product **3z**: as a yellow oil, 43.4 mg (0.0878 mmol), 88% yield; *E/Z* >19:1; $[\alpha]_D^{25}$ = +29.2 (*c* = 0.93, in $CHCl_3$); 80% ee, determined by HPLC analysis [Chiralpak column IA, *i*-PrOH/*n*-Hexane = 40/60, flow rate: 1.0 mL/min, 254 nm, t (minor) = 10.00 min, t (major) = 12.95 min]; ^{1}H NMR (400 MHz, $CDCl_3$) δ (ppm) 8.10–8.03 (m, 1H), 8.02–7.95 (m, 1H), 7.80–7.72 (m, 1H), 7.72–7.60 (m, 2H), 7.38–7.28 (m, 2H), 6.99 (d, *J* = 8.1 Hz, 2H), 6.95–6.89 (m, 2H), 6.65 (d, *J* = 8.9 Hz, 2H), 6.31 (d, *J* = 2.6 Hz, 1H), 4.76 (d, *J* = 2.6 Hz, 1H), 4.01 (q, *J* = 7.1 Hz, 2H), 3.84 (s, 3H), 3.69 (s, 3H), 0.98 (t, *J* = 7.1 Hz, 3H); ^{13}C NMR (100 MHz, $CDCl_3$) δ (ppm) 201.9, 198.7, 163.6, 159.3, 158.8, 144.8, 143.0, 142.3, 142.2, 137.4, 135.5, 135.2, 131.3, 129.9, 129.71, 129.69, 128.1, 123.1, 122.9, 114.1, 113.5, 70.8, 61.0, 59.4, 55.3, 55.0, 13.6; HRMS (ESI-TOF) *m/z*: [M + Na]$^+$ Calcd for $C_{31}H_{26}O_6Na^+$ 517.1622; Found 517.1621.

4.3 General procedure for (3+2) annulations of MBH carbonates with terminal allenes

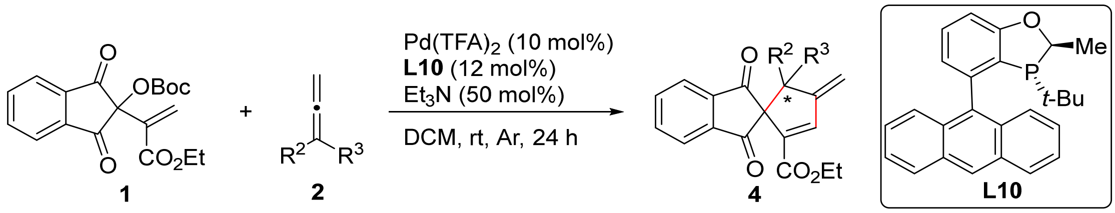

General procedure C: To an oven-dried 10 mL Schlenk tube equipped with a stir bar were added $Pd(TFA)_2$ (10 mol%), **L10** (12 mol%), Et_3N (50 mol%), MBH carbonate **1** (0.10 mmol, 1.0 equiv) and terminal allene **2** (0.20 mmol, 2.0 equiv). The tube was evacuated and back-filled with argon for five times. Then degassed DCM (1.0 mL) was added by syringe. The mixture was stirred at rt for 24 h. After completion, the mixture was concentrated and purified by flash chromatography on silica gel (EtOAc/petroleum ether) to give product **4**.

Synthesis of racemic 4: *Rac*-**4** was obtained under the catalysis of $Pd(TFA)_2$, (±)-**L13** and Et_3N.

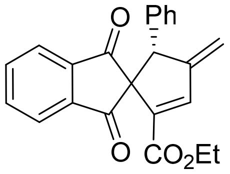

Synthesis of 4a (General procedure C): To an oven-dried 10 mL Schlenk tube equipped with a stir bar were added $Pd(TFA)_2$ (3.3 mg, 0.0099 mmol, 10 mol%), **L10** (4.6 mg, 0.012 mmol, 12 mol%), Et_3N (7.0 μL, 0.050 mmol, 50 mol%), ethyl 2-(2-((*tert*-butoxycarbonyl)oxy)-1,3-dioxo-2,3-dihydro-1*H*-inden-2-yl) acrylate **1b** (36.0 mg, 0.0999 mmol, 1.0 equiv) and propa-1,2-dien-1-ylbenzene **2r** (23.2 mg, 0.200 mmol, 2.0 equiv). The tube was evacuated and back-filled with argon for five times. Then degassed DCM (1.0 mL) was added by syringe. The mixture was stirred at rt for 24 h. After completion, the mixture was concentrated and purified by flash chromatography on silica gel (EtOAc/petroleum ether) to give product **4a**: as a yellow oil, 32.5 mg (0.0907 mmol), 91% yield; $[\alpha]_D^{25}$ = +81.3 (c = 1.65, in $CHCl_3$); 82% ee, determined by HPLC analysis [Chiralpak column IA, *i*-PrOH/*n*-Hexane = 20/80, flow rate: 1.0 mL/min, 254 nm, t (major) = 7.12 min, t (minor) = 7.83 min]; ^1H NMR (400 MHz, $CDCl_3$) δ (ppm) 8.04–7.94 (m, 1H), 7.75 (td, J = 7.4, 1.2 Hz, 1H), 7.66 (td, J = 7.4, 1.1 Hz, 1H), 7.62–7.54 (m, 2H), 7.12–7.06 (m, 3H), 7.04–6.96 (m, 2H), 5.60 (d, J = 3.1 Hz, 1H), 5.08 (d, J = 2.6 Hz, 1H), 4.64 (t, J = 2.9 Hz, 1H), 4.01 (q, J = 7.1 Hz, 2H), 1.00 (t, J = 7.1 Hz, 3H); ^{13}C NMR (100 MHz, $CDCl_3$) δ (ppm) 201.4, 198.3, 163.3, 151.7, 147.9, 142.2, 142.1, 137.2, 135.5, 135.4, 135.2, 130.0, 128.1, 127.6, 123.1, 123.0, 115.3, 71.2, 61.0, 58.5, 13.6; HRMS (ESI-TOF) *m/z*: $[M + Na]^+$ Calcd for $C_{23}H_{18}O_4Na^+$ 381.1097; Found 381.1093.

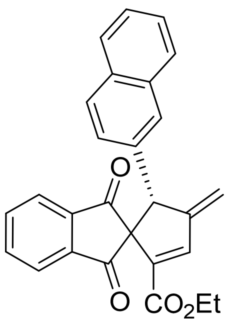

Synthesis of 4b (General procedure C): To an oven-dried 10 mL Schlenk tube equipped with a stir bar were added $Pd(TFA)_2$ (3.3 mg, 0.0099 mmol, 10 mol%), **L10** (4.6 mg, 0.012 mmol, 12 mol%), Et_3N (7.0 μL, 0.050 mmol, 50 mol%), ethyl 2-(2-((*tert*-butoxycarbonyl)oxy)-1,3-dioxo-2,3-dihydro-1*H*-inden-2-yl) acrylate **1b** (36.0 mg, 0.0999 mmol, 1.0 equiv) and 2-(propa-1,2-dien-1-yl) naphthalene **2s** (33.2 mg, 0.200 mmol, 2.0 equiv). The tube was evacuated and back-filled with argon for five times. Then degassed DCM (1.0 mL) was added by syringe. The mixture was stirred at rt for 24 h. After completion, the mixture was concentrated and purified by flash chromatography on silica gel (EtOAc/petroleum ether) to give product **4b**: as a yellow oil, 50.2 mg (0.0953 mmol), 95% yield; $[\alpha]_D^{25}$ = +69.6 (*c* = 0.43, in $CHCl_3$); 84% ee, determined by HPLC analysis [Chiralpak column IA, *i*-PrOH/*n*-Hexane = 20/80, flow rate: 1.0 mL/min, 254 nm, t (major) = 9.04 min, t (minor) = 10.92 min]; ^{1}H NMR (400 MHz, $CDCl_3$) δ (ppm) 7.99 (d, *J* = 7.7 Hz, 1H), 7.73–7.68 (m, 2H), 7.64–7.55 (m, 4H), 7.54–7.48 (m, 2H), 7.42–7.34 (m, 2H), 7.17 (dd, *J* = 8.5, 1.8 Hz, 1H), 5.63 (d, *J* = 3.1 Hz, 1H), 5.08 (d, *J* = 1.8 Hz, 1H), 4.81 (t, *J* = 2.8 Hz, 1H), 4.01 (q, *J* = 7.2 Hz, 2H), 1.00 (t, *J* = 7.1 Hz, 3H); ^{13}C NMR (100 MHz, $CDCl_3$) δ (ppm) 201.5, 198.4, 163.3, 151.9, 147.9, 142.2, 142.1, 137.4, 135.6, 135.3, 133.1, 132.9, 132.6, 129.4, 127.81, 127.78, 127.6, 127.5, 126.0, 125.9, 123.2, 123.0, 115.5, 71.1, 61.1, 58.5, 13.6; HRMS (ESI-TOF) *m/z*: $[M + H]^+$ Calcd for $C_{27}H_{21}O_4^+$ 409.1434; Found 409.1429.

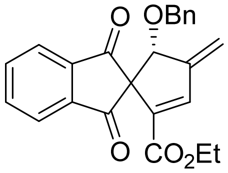

Synthesis of 4c (General procedure C): To an oven-dried 10 mL Schlenk tube equipped with a stir bar were added $Pd(TFA)_2$ (3.3 mg, 0.0099 mmol, 10 mol%), **L10** (4.6 mg, 0.012 mmol, 12 mol%), Et_3N (7.0 μL, 0.050 mmol, 50 mol%), ethyl 2-(2-((*tert*-butoxycarbonyl)oxy)-1,3-dioxo-2,3-dihydro-1*H*-inden-2-yl) acrylate **1b** (36.0 mg, 0.0999 mmol, 1.0 equiv) and ((propa-1,2-dien-1-yloxy)methyl)benzene **2t** (29.3 mg, 0.200 mmol, 2.0 equiv). The tube was evacuated and back-filled with argon for five times. Then degassed DCM (1.0 mL) was added by syringe. The mixture was stirred at rt for 24 h. After completion, the mixture was concentrated and purified by flash chromatography on silica gel (EtOAc/petroleum ether) to give product **4c**: as a colorless oil, 37.6 mg (0.0968 mmol), 97% yield; $[\alpha]_D^{25}$ = +56.2 (*c* = 1.90, in $CHCl_3$); 85% ee, determined by HPLC analysis [Chiralpak column IA, *i*-PrOH/*n*-Hexane = 20/80, flow rate: 1.0 mL/min, 254 nm, t (major) = 9.69 min, t (minor) = 11.89 min]; ^{1}H NMR (400 MHz, $CDCl_3$) δ (ppm) 8.07–7.96 (m, 2H), 7.90–7.82 (m, 2H), 7.39 (s, 1H), 7.22–7.11 (m, 3H), 7.02–6.93 (m, 2H), 5.51 (d, *J* = 2.8 Hz, 1H), 5.42 (d, *J* = 1.4 Hz, 1H), 5.03 (t, *J* = 2.5

Hz, 1H), 4.29–4.19 (m, 2H), 4.03–3.91 (m, 2H), 0.98 (t, J = 7.1 Hz, 3H); ^{13}C NMR (100 MHz, $CDCl_3$) δ (ppm) 200.9, 195.8, 163.0, 149.2, 145.1, 142.6, 142.2, 136.33, 136.31, 135.7, 135.4, 128.3, 128.1, 128.0, 123.6, 123.2, 114.8, 86.0, 74.3, 68.9, 61.1, 13.6; HRMS (ESI-TOF) *m/z*: $[M + Na]^+$ Calcd for $C_{24}H_{20}O_5Na^+$ 411.1203; Found 411.1205.

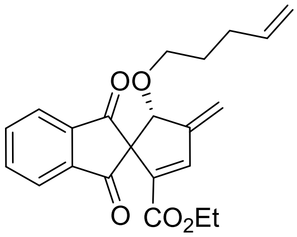

Synthesis of 4d (General procedure C): To an oven-dried 10 mL Schlenk tube equipped with a stir bar were added $Pd(TFA)_2$ (3.3 mg, 0.0099 mmol, 10 mol%), **L10** (4.6 mg, 0.012 mmol, 12 mol%), Et_3N (7.0 μL, 0.050 mmol, 50 mol%), ethyl 2-(2-((*tert*-butoxycarbonyl)oxy)-1,3-dioxo-2,3-dihydro-1*H*-inden-2-yl)acrylate **1b** (36.0 mg, 0.0999 mmol, 1.0 equiv) and 5-(propa-1,2-dien-1-yloxy)pent-1-ene **2u** (24.8 mg, 0.200 mmol, 2.0 equiv). The tube was evacuated and back-filled with argon for five times. Then degassed DCM (1.0 mL) was added by syringe. The mixture was stirred at rt for 24 h. After completion, the mixture was concentrated and purified by flash chromatography on silica gel (EtOAc/petroleum ether) to give product **4d**: as a colorless oil, 31.9 mg (0.0871 mmol), 87% yield; $[\alpha]_D^{25}$ = +88.4 (c = 0.19, in $CHCl_3$); 82% ee, determined by HPLC analysis [Chiralpak column OD-3, *i*-PrOH/*n*-Hexane = 10/90, flow rate: 1.0 mL/min, 254 nm, t (major) = 8.62 min, t (minor) = 14.95 min]; ^{1}H NMR (400 MHz, $CDCl_3$) δ (ppm) 8.11–7.99 (m, 2H), 7.87 (dd, J = 5.7, 3.1 Hz, 2H), 7.40 (s, 1H), 5.59–5.46 (m, 2H), 5.44 (d, J = 1.3 Hz, 1H), 4.92 (t, J = 2.5 Hz, 1H), 4.85–4.71 (m, 2H), 3.99 (q, J = 7.1 Hz, 2H), 3.44–3.26 (m, 1H), 3.11–2.90 (m, 1H), 1.93–1.68 (m, 2H), 1.39 (p, J = 6.9 Hz, 2H), 1.00 (t, J = 7.1 Hz, 3H); ^{13}C NMR (100 MHz, $CDCl_3$) δ (ppm) 201.2, 195.8, 163.1, 149.2, 145.1, 142.6, 142.3, 137.5, 136.3, 135.7, 135.5, 123.6, 123.2, 114.9, 114.7, 87.6, 72.1, 69.0, 61.1, 29.7, 28.6, 13.6; HRMS (ESI-TOF) *m/z*: $[M + Na]^+$ Calcd for $C_{22}H_{22}O_5Na^+$ 389.1359; Found 389.1358.

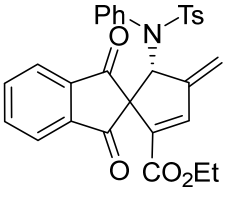

Synthesis of 4e (General procedure C): To an oven-dried 10 mL Schlenk tube equipped with a stir bar were added $Pd(TFA)_2$ (3.3 mg, 0.0099 mmol, 10 mol%), **L10** (4.6 mg, 0.012 mmol, 12 mol%), Et_3N (7.0 μL, 0.050 mmol, 50 mol%), ethyl 2-(2-((*tert*-butoxycarbonyl)oxy)-1,3-dioxo-2,3-dihydro-1*H*-inden-2-yl) acrylate **1b** (36.0 mg, 0.0999 mmol, 1.0 equiv) and 4-methyl-*N*-phenyl-*N*-(propa-1,2-dien-1-yl)benzene sulfonamide **2v** (57.1 mg, 0.200 mmol, 2.0 equiv). The tube was evacuated and back-filled with argon for five times. Then degassed DCM (1.0 mL) was added by syringe. The mixture was stirred at rt for 24 h. After completion, the mixture was concentrated and purified by flash chromatography on silica

gel (EtOAc/petroleum ether) to give product **4e**: as a yellow solid, 50.2 mg (0.0953 mmol), 95% yield; mp 140–134 °C; $[\alpha]_D^{25}$ = –210.2 (*c* = 1.08, in $CHCl_3$); 86% ee, determined by HPLC analysis [Chiralpak column IA, *i*-PrOH/*n*-Hexane = 40/60, flow rate: 1.0 mL/min, 254 nm, t (minor) = 9.26 min, t (major) = 23.67 min]; ^{1}H NMR (400 MHz, $CDCl_3$) δ (ppm) 8.06–7.96 (m, 2H), 7.90–7.81 (m, 2H), 7.50 (d, *J* = 8.4 Hz, 2H), 7.30–7.22 (m, 3H), 7.21–7.16 (m, 4H), 7.13 (s, 1H), 5.72 (t, *J* = 1.9 Hz, 1H), 5.50 (d, *J* = 2.0 Hz, 1H), 5.09 (s, 1H), 4.03–3.86 (m, 2H), 2.39 (s, 3H), 0.92 (t, *J* = 7.1 Hz, 3H); ^{13}C NMR (100 MHz, $CDCl_3$) δ (ppm) 199.6, 196.2, 162.8, 146.8, 145.7, 144.2, 143.8, 141.2, 138.6, 137.0, 136.8, 135.8, 135.0, 131.4, 129.5, 128.3, 128.2, 127.8, 123.4, 123.3, 119.3, 69.1, 68.5, 61.2, 21.5, 13.5; HRMS (ESI-TOF) *m/z*: $[M + Na]^+$ Calcd for $C_{30}H_{25}NO_6SNa^+$ 550.1295; Found 550.1287.

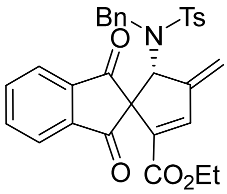

Synthesis of 4f (General procedure C): To an oven-dried 10 mL Schlenk tube equipped with a stir bar were added $Pd(TFA)_2$ (3.3 mg, 0.0099 mmol, 10 mol%), **L10** (4.6 mg, 0.012 mmol, 12 mol%), Et_3N (7.0 μL, 0.050 mmol, 50 mol%), ethyl 2-(2-((*tert*-butoxycarbonyl)oxy)-1,3-dioxo-2,3-dihydro-1*H*-inden-2-yl) acrylate **1b** (36.0 mg, 0.0999 mmol, 1.0 equiv) and *N*-benzyl-4-methyl-*N*-(propa-1,2-dien-1-yl)benzene sulfonamide **2w** (59.8 mg, 0.200 mmol, 2.0 equiv). The tube was evacuated and back-filled with argon for five times. Then degassed DCM (1.0 mL) was added by syringe. The mixture was stirred at rt for 24 h. After completion, the mixture was concentrated and purified by flash chromatography on silica gel (EtOAc/petroleum ether) to give product **4f**: as a yellow oil, 51.4 mg (0.0950 mmol), 95% yield; $[\alpha]_D^{25}$ = –50.0 (*c* = 2.56, in $CHCl_3$); 80% ee, determined by HPLC analysis [Chiralpak column AD-H, *i*-PrOH/*n*-Hexane = 40/60, flow rate: 1.0 mL/min, 254 nm, t (minor) = 11.74 min, t (major) = 25.38 min]; ^{1}H NMR (400 MHz, $CDCl_3$) δ (ppm) 7.97–7.91 (m, 1H), 7.82–7.74 (m, 3H), 7.21–7.15 (m, 3H), 7.12–7.04 (m, 5H), 6.92 (d, *J* = 8.1 Hz, 2H), 5.43 (d, *J* = 2.2 Hz, 1H), 5.35 (t, *J* = 2.1 Hz, 1H), 4.89 (d, *J* = 1.9 Hz, 1H), 4.70 (d, *J* = 16.2 Hz, 1H), 4.37 (d, *J* = 16.2 Hz, 1H), 3.96–3.82 (m, 2H), 2.23 (s, 3H), 0.87 (t, *J* = 7.1 Hz, 3H); ^{13}C NMR (100 MHz, $CDCl_3$) δ (ppm) 199.7, 197.3, 162.7, 146.8, 145.5, 143.5, 143.3, 142.0, 138.8, 137.4, 137.2, 135.4, 135.2, 129.3, 128.7, 127.8, 127.1, 127.0, 123.5, 123.4, 118.5, 69.0, 67.8, 61.2, 50.9, 21.4, 13.5; HRMS (ESI-TOF) *m/z*: $[M + Na]^+$ Calcd for $C_{31}H_{27}NO_6SNa^+$ 564.1451; Found 564.1454.

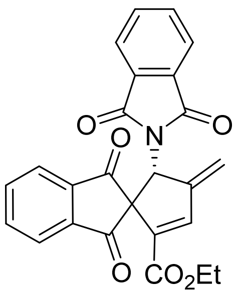

Synthesis of 4g (General procedure C): To an oven-dried 10 mL Schlenk tube equipped with a stir bar were added $Pd(TFA)_2$ (3.3 mg, 0.0099 mmol, 10 mol%), **L10** (4.6 mg, 0.012 mmol, 12 mol%), Et_3N (7.0 μL, 0.050 mmol, 50 mol%), ethyl 2-(2-((*tert*-butoxycarbonyl)oxy)-1,3-dioxo-2,3-dihydro-1*H*-inden-2-yl) acrylate **1b** (36.0 mg, 0.0999 mmol, 1.0 equiv) and 2-(propa-1,2-dien-1-yl) isoindoline-1,3-dione **2x** (37.0 mg, 0.200 mmol, 2.0 equiv). The tube was evacuated and back-filled with argon for five times. Then degassed DCM (1.0 mL) was added by syringe. The mixture was stirred at rt for 24 h. After completion, the mixture was concentrated and purified by flash chromatography on silica gel (EtOAc/petroleum ether) to give product **4g**: as a yellow oil, 39.8 mg (0.0932 mmol), 93% yield; $[\alpha]_D^{25}$ = –141.6 (*c* = 1.09, in $CHCl_3$); 72% ee, determined by HPLC analysis [Chiralpak column IB, *i*-PrOH/*n*-Hexane = 20/80, flow rate: 1.0 mL/min, 254 nm, t (major) = 18.04 min, t (minor) = 20.66 min]; ^1H NMR (400 MHz, $CDCl_3$) δ (ppm) 8.06 (d, *J* = 7.4 Hz, 1H), 7.89–7.73 (m, 5H), 7.72–7.67 (m, 2H), 7.62 (s, 1H), 5.69 (d, *J* = 2.2 Hz, 1H), 5.54 (t, *J* = 2.0 Hz, 1H), 5.33 (s, 1H), 4.00 (q, *J* = 7.1 Hz, 2H), 1.03 (t, *J* = 7.1 Hz, 3H); ^{13}C NMR (100 MHz, $CDCl_3$) δ (ppm) 199.0, 196.1, 163.1, 146.9, 146.7, 142.5, 141.4, 137.9, 135.9, 135.7, 134.2, 123.8, 123.5, 123.3, 116.4, 67.9, 61.2, 57.2, 13.6; HRMS (ESI-TOF) *m/z*: $[M + Na]^+$ Calcd for $C_{20}H_{17}NO_6Na^+$ 450.0948; Found 450.0948.

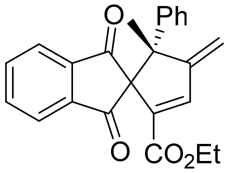

Synthesis of 4h (General procedure C): To an oven-dried 10 mL Schlenk tube equipped with a stir bar were added $Pd(TFA)_2$ (3.3 mg, 0.0099 mmol, 10 mol%), **L10** (4.6 mg, 0.012 mmol, 12 mol%), Et_3N (7.0 μL, 0.050 mmol, 50 mol%), ethyl 2-(2-((*tert*-butoxycarbonyl)oxy)-1,3-dioxo-2,3-dihydro-1*H*-inden-2-yl) acrylate **1b** (36.0 mg, 0.0999 mmol, 1.0 equiv) and buta-2,3-dien-2-ylbenzene **2y** (26.0 mg, 0.200 mmol, 2.0 equiv). The tube was evacuated and back-filled with argon for five times. Then degassed DCM (1.0 mL) was added by syringe. The mixture was stirred at rt for 24 h. After completion, the mixture was concentrated and purified by flash chromatography on silica gel (EtOAc/petroleum ether) to give product **4h**: as a yellow oil, 50.2 mg (0.0953 mmol), 95% yield; $[\alpha]_D^{25}$ = –137.9 (*c* = 0.95, in $CHCl_3$); 88% ee, determined by HPLC analysis [Chiralpak column IB, *i*-PrOH/*n*-Hexane = 20/80, flow rate: 1.0 mL/min, 254 nm, t (major) = 8.04 min, t (minor) = 14.10 min]; ^1H NMR (400 MHz, $CDCl_3$) δ (ppm) 8.01 (dt, *J* = 7.7, 1.0 Hz, 1H), 7.78 (td, *J* = 7.5, 1.1 Hz, 1H), 7.66 (td, *J* = 7.5, 1.1 Hz, 1H), 7.59 (s, 1H), 7.45 (dt, *J* = 7.6, 1.0 Hz, 1H), 7.13–7.05 (m, 3H), 7.04–6.95 (m, 2H), 5.69 (s, 1H), 5.11 (s, 1H), 4.04 (q, *J* = 7.1 Hz, 2H), 1.64 (s, 3H), 1.07 (t, *J* = 7.1 Hz, 3H); ^{13}C NMR (100 MHz, $CDCl_3$) δ

(ppm) 198.6, 197.9, 163.5, 158.7, 147.2, 142.9, 142.5, 140.7, 136.4, 135.3, 135.1, 128.5, 127.7, 127.0, 122.91, 122.86, 115.9, 75.1, 61.0, 58.7, 27.6, 13.8; HRMS (ESI-TOF) *m/z*: $[M + H]^+$ Calcd for $C_{24}H_{21}O_4^+$ 373.1434; Found 373.1432.

4.4 General procedure for (3+2) annulations of MBH carbonates with other allenes

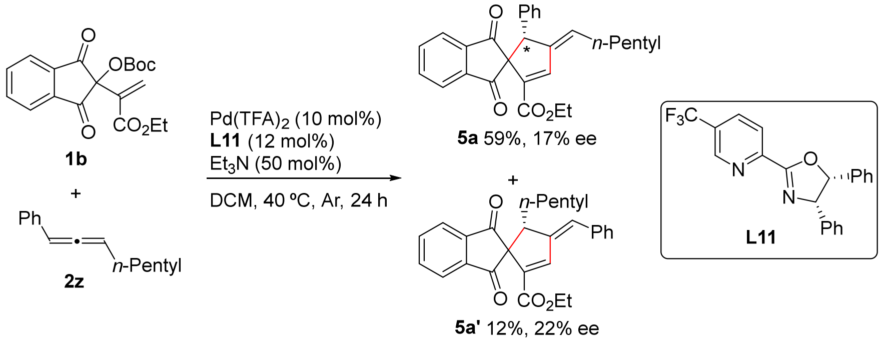

Synthesis of 5a and 5a′: To an oven-dried 10 mL Schlenk ube equipped with a stir bar were added $Pd(TFA)_2$ (3.3 mg, 0.0099 mmol, 10 mol%), **L11** (4.6 mg, 0.012 mmol, 12 mol%), Et_3N (7.0 μL, 0.050 mmol, 50 mol%), ethyl 2-(2-((*tert*-butoxycarbonyl)oxy)-1,3-dioxo-2,3-dihydro-1*H*-inden-2-yl)acrylate **1b** (36.0 mg, 0.0999 mmol, 1.0 equiv) and octa-1,2-dien-1-ylbenzene **2z** (37.3 mg, 0.201 mmol, 2.0 equiv). The tube was evacuated and back-filled with argon for five times. Then degassed DCM (1.0 mL) was added by syringe. The mixture was stirred at rt for 24 h. After completion, the mixture was concentrated and purified by flash chromatography on silica gel (EtOAc/petroleum ether) to give product **5a** and **5a′** as inseparable regioisomers: as a yellow oil, 30.6 mg (0.0714 mmol), 71% yield; *E*:*Z* >19:1; 5:1 rr, determined by ^{1}H NMR analysis; $[\alpha]_D^{25}$ = +16.4 (*c* = 1.22, in $CHCl_3$); **5a**: 17% ee, determined by HPLC analysis [Chiralpak column AS-H, *i*-PrOH/*n*-Hexane = 20/80, flow rate: 1.0 mL/min, 254 nm, t (minor) = 5.35 min, t (major) = 11.86 min]; ^{1}H NMR (400 MHz, $CDCl_3$) δ (ppm) 7.98 (d, *J* = 7.7 Hz, 1H), 7.86–7.82 (m, 1H), 7.77–7.71 (m, 1H), 7.68–7.62 (m, 1H), 7.59–7.55 (m, 1H), 7.10–7.05 (m, 3H), 7.01–6.94 (m, 2H), 5.53–5.32 (m, 1H), 4.62 (q, *J* = 2.2 Hz, 1H), 4.00 (q, *J* = 7.1 Hz, 2H), 2.46–2.21 (m, 2H), 1.47–1.38 (m, 2H), 1.36–1.26 (m, *J* = 4.2, 3.5 Hz, 4H), 1.00 (t, *J* = 7.2 Hz, 3H), 0.93–0.87 (m, 3H); ^{13}C NMR (100 MHz, $CDCl_3$) δ (ppm) 194.9, 191.7, 156.6, 137.3, 135.8, 135.25, 135.20, 129.2, 128.4, 128.3, 128.1, 125.7, 123.1, 121.0, 120.4, 116.0, 115.9, 63.9, 53.9, 51.8, 24.4, 23.1, 22.2, 15.5, 7.0, 6.7; HRMS (ESI-TOF) *m/z*: $[M + H]^+$ Calcd for $C_{28}H_{29}O_4^+$ 429.2060; Found 429.2057. **5a′**: 22% ee, determined by HPLC analysis [Chiralpak column AS-H, *i*-PrOH/*n*-Hexane = 20/80, flow rate: 1.0 mL/min, 254 nm, t (major) = 9.13 min, t (minor) = 15.25 min].

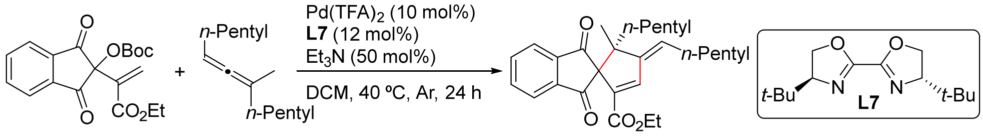

1b **2aa** **5b** 71%, 30% ee, 12:1 rr

Synthesis of 5b: To an oven-dried 10 mL Schlenk tube equipped with a stir bar were added Pd(TFA)$_2$ (3.3 mg, 0.0099 mmol, 10 mol%), **L7** (3.0 mg, 0.012 mmol, 12 mol%), Et$_3$N (7.0 μL, 0.050 mmol, 50 mol%), ethyl 2-(2-((*tert*-butoxycarbonyl)oxy)-1,3-dioxo-2,3-dihydro-1*H*-inden-2-yl)acrylate **1b** (36.0 mg, 0.0999 mmol, 1.0 equiv) and 6-methyltrideca-6,7-diene **2aa** (38.9 mg, 0.200 mmol, 2.0 equiv). The tube was evacuated and back-filled with argon for five times. Then degassed DCM (1.0 mL) was added by syringe. The mixture was stirred at rt for 24 h. After completion, the mixture was concentrated and purified by flash chromatography on silica gel (EtOAc/petroleum ether) to give product **5b**: as a yellow oil, 30.6 mg (0.0714 mmol), 71% yield; *E*:*Z* >19:1; 12:1 rr, determined by ^{1}H NMR analysis of crude product; $[\alpha]_D^{25} = -11.1$ ($c = 1.26$, in CHCl$_3$); 30% ee, determined by HPLC analysis [Chiralpak column IC, *i*-PrOH/*n*-Hexane = 5/95, flow rate: 1.0 mL/min, 254 nm, t (minor) = 14.54 min, t (major) = 20.61 min]; ^{1}H NMR (400 MHz, CDCl$_3$) δ (ppm) 8.04–7.92 (m, 2H), 7.89–7.78 (m, 2H), 7.66 (s, 1H), 5.38 (t, *J* = 7.8 Hz, 1H), 4.00 (qd, *J* = 7.1, 1.8 Hz, 2H), 2.37–2.20 (m, 2H), 1.64–1.42 (m, 4H), 1.37–1.29 (m, *J* = 4.4, 3.9 Hz, 4H), 1.24–0.98 (m, 11H), 0.93–0.87 (m, 3H), 0.82–0.67 (m, 4H); ^{13}C NMR (100 MHz, CDCl$_3$) δ (ppm) 199.8, 199.6, 163.9, 149.4, 143.2, 143.0, 142.3, 135.6, 135.31, 135.28, 129.5, 123.1, 122.8, 72.5, 60.8, 55.2, 39.1, 32.1, 31.4, 29.8, 29.5, 24.7, 22.6, 22.5, 22.4, 14.0, 13.9, 13.7; HRMS (ESI-TOF) *m/z*: [M + Na]$^+$ Calcd for $C_{28}H_{36}O_4Na^+$ 459.2506; Found 459.2513.

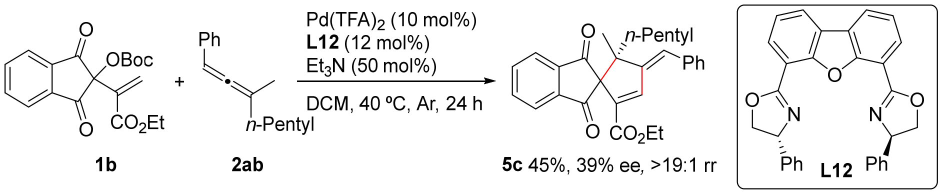

1b **2ab** **5c** 45%, 39% ee, >19:1 rr

Synthesis of 5c: To an oven-dried 10 mL Schlenk tube equipped with a stir bar were added Pd(TFA)$_2$ (3.3 mg, 0.0099 mmol, 10 mol%), **L12** (4.6 mg, 0.012 mmol, 12 mol%), Et$_3$N (7.0 μL, 0.050 mmol, 50 mol%), ethyl 2-(2-((*tert*-butoxycarbonyl)oxy)-1,3-dioxo-2,3-dihydro-1*H*-inden-2-yl)acrylate **1b** (36.0 mg, 0.0999 mmol, 1.0 equiv) and (3-methylocta-1,2-dien-1-yl) benzene **2ab** (40.0 mg, 0.200 mmol, 2.0 equiv). The tube was evacuated and back-filled with argon for five times. Then degassed DCM (1.0 mL) was added by syringe. The mixture was stirred at rt for 48 h. After completion, the mixture was concentrated and purified by flash chromatography on silica gel (EtOAc/petroleum ether)

to give product **5c**: as a yellow oil, 19.9 mg (0.0714 mmol), 45% yield; *E*:*Z* >19:1; >19:1 rr; $[\alpha]_D^{25}$ = –10.3 (*c* = 0.35, in $CHCl_3$); 39% ee, determined by HPLC analysis [Chiralpak column IC, *i*-PrOH/*n*-Hexane = 10/90, flow rate: 1.0 mL/min, 254 nm, t (minor) = 9.37 min, t (minor) = 12.64 min]; ^1H NMR (400 MHz, $CDCl_3$) δ (ppm) 8.07–7.96 (m, 2H), 7.89–7.79 (m, 3H), 7.43–7.34 (m, 4H), 7.33–7.27 (m, 1H), 6.40 (s, 1H), 4.01 (q, *J* = 7.0 Hz, 2H), 1.76–1.61 (m, 2H), 1.31–1.24 (m, 1H), 1.21 (s, 3H), 1.19–1.06 (m, 4H), 1.01 (t, *J* = 7.1 Hz, 3H), 0.88–0.79 (m, 1H), 0.79–0.73 (m, 3H); ^{13}C NMR (100 MHz, $CDCl_3$) δ (ppm) 199.4, 199.2, 163.7, 151.4, 143.5, 143.2, 142.3, 138.6, 137.2, 135.42, 135.40, 128.7, 128.5, 127.5, 127.3, 123.2, 122.9, 72.3, 61.0, 56.4, 39.3, 32.1, 24.8, 22.6, 22.4, 13.9, 13.7; HRMS (ESI-TOF) *m/z*: $[M + Na]^+$ Calcd for $C_{29}H_{30}O_4Na^+$ 465.2036; Found 465.2043.

4.5 General procedure for (3+2) annulations between MBH carbonate 1b and alkenes 6

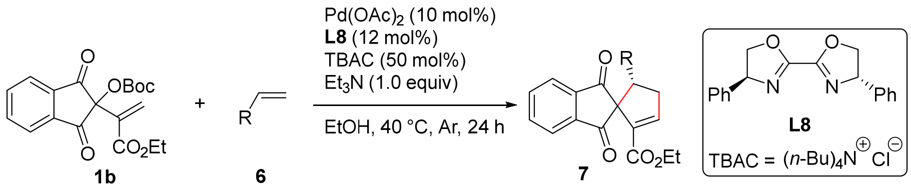

General procedure D: To an oven-dried 10 mL Schlenk tube equipped with a stir bar were added $Pd(OAc)_2$ (10 mol%), **L8** (12 mol%), TBAC (tetrabutylammonium chloride, 50 mol%), Et_3N (0.10 mmol, 1.0 equiv), MBH carbonate **1b** (0.10 mmol, 1.0 equiv) and alkenes **6** (0.50 mmol, 5.0 equiv). The tube was evacuated and back-filled with argon for five times. Then degassed EtOH (1.0 mL) was added by syringe. The mixture was stirred at 40 °C for 24 h. After completion, the mixture was concentrated and purified by flash chromatography on silica gel (EtOAc/petroleum ether) to give product **7**.

Synthesis of racemic 7: *Rac*-**7** was obtained under the catalysis of $Pd(OAc)_2$, (±)-**L7**, TBAC and Et_3N.

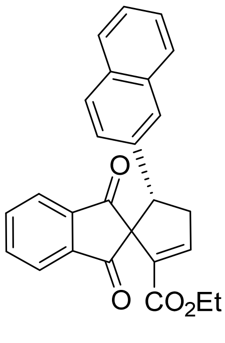

Synthesis of 7a (General procedure D): To an oven-dried 10 mL Schlenk tube equipped with a stir bar were added $Pd(OAc)_2$ (2.2 mg, 0.010 mmol, 10 mol%), **L8** (3.6 mg, 0.012 mmol, 12 mol%), TBAC (13.9 mg, 0.0500 mmol, 50 mol%), Et_3N (14.0 μL, 0.100 mmol, 1.0 equiv), ethyl 2-(2-((*tert*-butoxycarbonyl)oxy)-1,3-dioxo-2,3-dihydro-1*H*-inden-2-yl)acrylate **1b** (36.0 mg, 0.0999 mmol, 1.0 equiv) and 2-vinylnaphthalene **6a** (77.0 mg, 0.499 mmol, 5.0 equiv). The tube was evacuated and back-filled with argon for five times. Then degassed EtOH (1.0 mL) was added

by syringe. The mixture was stirred at 40 °C for 24 h. After completion, the mixture was concentrated and purified by flash chromatography on silica gel (EtOAc/petroleum ether) to give product **7a**: as a yellow solid, 17.2 mg (0.0434 mmol), 43% yield; mp 154–156 °C; $[\alpha]_D^{25}$ = +23.7 (*c* = 0.73, in $CHCl_3$); 84% ee, determined by HPLC analysis [Chiralpak column IA, *i*-PrOH/*n*-Hexane = 20/80, flow rate: 1.0 mL/min, 254 nm, t (major) = 8.49 min, t (minor) = 9.95 min]; ^1H NMR (400 MHz, $CDCl_3$) δ (ppm) 8.00–7.91 (m, 1H), 7.71–7.61 (m, 3H), 7.60–7.50 (m, 4H), 7.45 (t, *J* = 2.5 Hz, 1H), 7.40–7.33 (m, 2H), 7.17 (dd, *J* = 8.5, 1.9 Hz, 1H), 4.39 (dd, *J* = 10.6, 8.1 Hz, 1H), 4.07–3.89 (m, 2H), 3.55–3.41 (m, 1H), 3.09–2.95 (m, 1H), 0.98 (t, *J* = 7.1 Hz, 3H); ^{13}C NMR (100 MHz, $CDCl_3$) δ (ppm) 202.1, 200.0, 162.9, 149.4, 142.8, 141.7, 135.8, 135.4, 135.3, 133.8, 132.9, 132.5, 127.9, 127.8, 127.6, 127.4, 126.3, 126.0, 125.9, 123.1, 122.8, 70.5, 60.8, 54.7, 37.8, 13.6; HRMS (ESI-TOF) *m/z*: $[M + Na]^+$ Calcd for $C_{26}H_{20}O_4Na^+$ 419.1254; Found 419.1249.

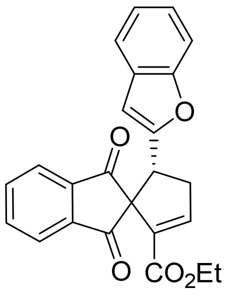

Synthesis of 7b (General procedure D): To an oven-dried 10 mL Schlenk tube equipped with a stir bar were added $Pd(OAc)_2$ (2.2 mg, 0.010 mmol, 10 mol%), **L8** (3.6 mg, 0.012 mmol, 12 mol%), TBAC (13.9 mg, 0.0500 mmol, 50 mol%), Et_3N (14.0 μL, 0.100 mmol, 1.0 equiv), ethyl 2-(2-((*tert*-butoxycarbonyl)oxy)-1,3-dioxo-2,3-dihydro-1*H*-inden-2-yl)acrylate **1b** (36.0 mg, 0.0999 mmol, 1.0 equiv) and 2-vinylbenzofuran **6b** (72.1 mg, 0.500 mmol, 5.0 equiv). The tube was evacuated and back-filled with argon for five times. Then degassed EtOH (1.0 mL) was added by syringe. The mixture was stirred at 40 °C for 24 h. After completion, the mixture was concentrated and purified by flash chromatography on silica gel (EtOAc/petroleum ether) to give product **7b**: as a yellow solid, 17.2 mg (0.0446 mmol), 45% yield; mp 109–111 °C; $[\alpha]_D^{25}$ = +33.7 (*c* = 0.67, in MeOH); 78% ee, determined by HPLC analysis [Chiralpak column AD-H, *i*-PrOH/*n*-Hexane = 20/80, flow rate: 1.0 mL/min, 254 nm, t (minor) = 22.61 min, t (major) = 25.53 min]; ^1H NMR (400 MHz, $CDCl_3$) δ (ppm) 8.13–8.06 (m, 1H), 7.88–7.77 (m, 1H), 7.76–7.67 (m, 2H), 7.43–7.32 (m, 2H), 7.13–7.02 (m, 2H), 6.97–6.88 (m, 1H), 6.44 (s, 1H), 4.42–4.33 (m, 1H), 4.05–3.90 (m, 2H), 3.38–3.27 (m, 1H), 3.18–3.06 (m, 1H), 0.96 (t, *J* = 7.1 Hz, 3H); ^{13}C NMR (100 MHz, $CDCl_3$) δ (ppm) 201.1, 198.8, 162.6, 154.6, 154.4, 148.4, 142.8, 141.7, 136.0, 135.4, 135.3, 127.9, 123.9, 123.1, 122.6, 120.8, 110.5, 104.3, 68.6, 61.0, 47.1, 36.1, 13.6; HRMS (ESI-TOF) *m/z*: $[M + Na]^+$ Calcd for $C_{24}H_{18}O_5Na^+$ 409.1046; Found 409.1045.

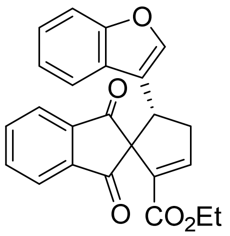

Synthesis of 7c (General procedure D): To an oven-dried 10 mL Schlenk tube equipped with a stir bar were added $Pd(OAc)_2$ (2.2 mg, 0.010 mmol, 10 mol%), **L8** (3.6 mg, 0.012 mmol, 12 mol%), TBAC (13.9 mg, 0.0500 mmol, 50 mol%), Et_3N (14.0 μL, 0.100 mmol, 1.0 equiv), ethyl 2-(2-((*tert*-butoxycarbonyl)oxy)-1,3-dioxo-2,3-dihydro-1*H*-inden-2-yl)acrylate **1b** (36.0 mg, 0.0999 mmol, 1.0 equiv) and 3-vinylbenzofuran **6c** (72.1 mg, 0.500 mmol, 5.0 equiv). The tube was evacuated and back-filled with argon for five times. Then degassed EtOH (1.0 mL) was added by syringe. The mixture was stirred at 40 °C for 24 h. After completion, the mixture was concentrated and purified by flash chromatography on silica gel (EtOAc/petroleum ether) to give product **7c**: as a yellow oil, 14.3 mg (0.0370 mmol), 37% yield; $[\alpha]_D^{25}$ = +229.4 (*c* = 0.53, in $CHCl_3$); 89% ee, determined by HPLC analysis [Chiralpak column IA, *i*-PrOH/*n*-Hexane = 40/60, flow rate: 1.0 mL/min, 254 nm, t (minor) = 9.34 min, t (major) = 10.95 min]; ^{1}H NMR (400 MHz, $CDCl_3$) δ (ppm) 7.95–7.88 (m, 1H), 7.68–7.55 (m, 3H), 7.47 (s, 1H), 7.41 (t, *J* = 2.5 Hz, 1H), 7.25 (d, *J* = 7.0 Hz, 2H), 7.13–7.06 (m, 1H), 7.04–6.97 (m, 1H), 4.40 (dd, *J* = 10.8, 8.1 Hz, 1H), 4.09–3.90 (m, 2H), 3.35–3.18 (m, 1H), 3.15–2.94 (m, 1H), 0.99 (t, *J* = 7.1 Hz, 3H); ^{13}C NMR (100 MHz, $CDCl_3$) δ (ppm) 202.1, 199.8, 162.8, 154.8, 149.1, 143.6, 143.0, 141.3, 136.4, 135.4, 135.3, 126.7, 124.4, 123.1, 122.8, 122.4, 119.8, 116.6, 111.2, 69.3, 60.9, 44.0, 38.1, 13.6; HRMS (ESI-TOF) *m/z*: $[M + Na]^+$ Calcd for $C_{24}H_{18}O_5Na^+$ 409.1046; Found 409.1042.

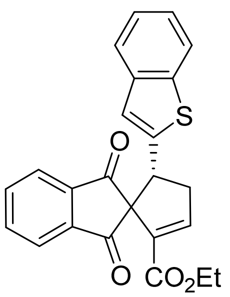

Synthesis of 7d (General procedure D): To an oven-dried 10 mL Schlenk tube equipped with a stir bar were added $Pd(OAc)_2$ (2.2 mg, 0.010 mmol, 10 mol%), **L8** (3.6 mg, 0.012 mmol, 12 mol%), TBAC (13.9 mg, 0.0500 mmol, 50 mol%), Et_3N (14.0 μL, 0.100 mmol, 1.0 equiv), ethyl 2-(2-((*tert*-butoxycarbonyl)oxy)-1,3-dioxo-2,3-dihydro-1*H*-inden-2-yl)acrylate **1b** (36.0 mg, 0.0999 mmol, 1.0 equiv) and 2-vinylbenzo[*b*]thiophene **6d** (80.1 mg, 0.500 mmol, 5.0 equiv). The tube was evacuated and back-filled with argon for five times. Then degassed EtOH (1.0 mL) was added by syringe. The mixture was stirred at 40 °C for 24 h. After completion, the mixture was concentrated and purified by flash chromatography on silica gel (EtOAc/petroleum ether) to give product **7d**: as a yellow solid, 22.4 mg (0.0556 mmol), 56% yield; mp 112–114 °C; $[\alpha]_D^{25}$ = +16.1 (*c* = 1.00, in $CHCl_3$); 82% ee, determined by HPLC analysis [Chiralpak column AD-H, *i*-PrOH/*n*-Hexane = 20/80, flow rate: 1.0 mL/min, 254 nm, t (major) = 16.82 min, t (minor) = 20.18 min]; ^{1}H NMR (400 MHz, $CDCl_3$) δ (ppm) 8.03 (dt, *J* = 7.6, 1.0 Hz, 1H), 7.80–7.65 (m, 3H), 7.61–7.53 (m,

2H), 7.38 (t, J = 2.5 Hz, 1H), 7.25–7.15 (m, 2H), 7.00 (s, 1H), 4.56–4.47 (m, 1H), 4.02–3.92 (m, 2H), 3.47–3.34 (m, 1H), 3.18–3.06 (m, 1H), 0.97 (t, J = 7.1 Hz, 3H); ^{13}C NMR (150 MHz, $CDCl_3$) δ (ppm) 201.3, 199.4, 162.6, 148.6, 143.1, 141.8, 140.2, 139.2, 139.1, 136.2, 135.6, 135.5, 124.2, 124.1, 123.4, 123.3, 123.1, 123.0, 122.0, 69.6, 60.9, 49.6, 39.1, 13.6; HRMS (ESI-TOF) *m/z*: $[M + H]^+$ Calcd for $C_{24}H_{19}O_4S^+$ 403.0999; Found 403.0994.

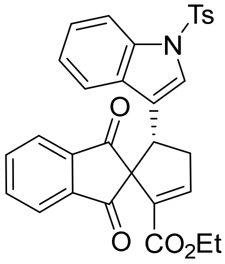

Synthesis of 7e (General procedure D): To an oven-dried 10 mL Schlenk tube equipped with a stir bar were added $Pd(OAc)_2$ (2.2 mg, 0.010 mmol, 10 mol%), **L8** (3.6 mg, 0.012 mmol, 12 mol%), TBAC (13.9 mg, 0.0500 mmol, 50 mol%), Et_3N (14.0 μL, 0.100 mmol, 1.0 equiv), ethyl 2-(2-((*tert*-butoxycarbonyl)oxy)-1,3-dioxo-2,3-dihydro-1*H*-inden-2-yl)acrylate **1b** (36.0 mg, 0.0999 mmol, 1.0 equiv) and 1-tosyl-3-vinyl-1*H*-indole **6e** (148.7 mg, 0.5000 mmol, 5.0 equiv). The tube was evacuated and back-filled with argon for five times. Then degassed EtOH (1.0 mL) was added by syringe. The mixture was stirred at 40 °C for 24 h. After completion, the mixture was concentrated and purified by flash chromatography on silica gel (EtOAc/petroleum ether) to give product **7e**: as a yellow solid, 30.1 mg (0.0558 mmol), 56% yield; mp 76–78 °C; $[\alpha]_D^{25}$ = +11.6 (c = 2.04, in $CHCl_3$); 85% ee, determined by HPLC analysis [Chiralpak column AD-H, *i*-PrOH/*n*-Hexane = 20/80, flow rate: 1.0 mL/min, 254 nm, t (minor) = 26.00 min, t (major) = 30.40 min]; ^{1}H NMR (400 MHz, $CDCl_3$) δ (ppm) 7.88–7.78 (m, 1H), 7.77–7.71 (m, 1H), 7.69–7.61 (m, 2H), 7.58–7.51 (m, 1H), 7.47–7.37 (m, 3H), 7.26–7.19 (m, 3H), 7.18–7.12 (m, 1H), 7.12–7.05 (m, 1H), 7.01–6.91 (m, 1H), 4.46–4.35 (m, 1H), 4.04–3.91 (m, 2H), 3.36–3.21 (m, 1H), 3.11–2.96 (m, 1H), 2.38 (s, 3H), 0.98 (t, J = 7.1 Hz, 3H); ^{13}C NMR (100 MHz, $CDCl_3$) δ (ppm) 202.1, 199.4, 162.8, 149.0, 144.6, 142.8, 141.0, 136.0, 135.12, 135.08, 135.0, 134.4, 129.8, 129.7, 126.8, 125.4, 124.7, 123.0, 122.7, 122.6, 119.4, 118.5, 113.2, 69.6, 60.9, 44.5, 38.3, 21.5, 13.6; HRMS (ESI-TOF) *m/z*: $[M + Na]^+$ Calcd for $C_{31}H_{25}NO_6SNa^+$ 562.1295; Found 562.1300.

4.6 General procedure for (3+2) annulations of other MBH carbonates and allenes

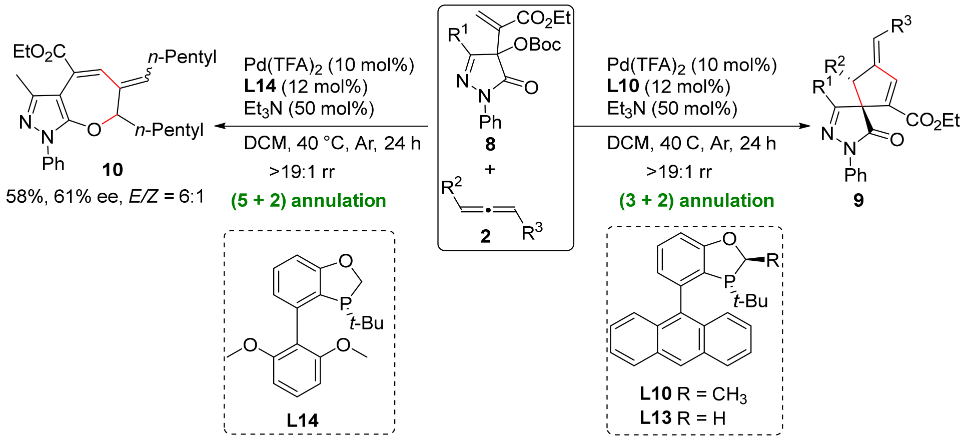

General procedure E: To an oven-dried 10 mL Schlenk tube equipped with a stir bar were added $Pd(TFA)_2$ (10 mol%), **L** (12 mol%), Et_3N (50 mol%), pyrazolone-derived MBH carbonate **8** (0.10 mmol, 1.0 equiv) and allene **2** (0.20 mmol, 2.0 equiv). The tube was evacuated and back-filled with argon for five times. Then degassed DCM (1.0 mL) was added by syringe. The mixture was stirred at 40 °C for 24 h. After completion, the mixture was concentrated and purified by flash chromatography on silica gel (EtOAc/petroleum ether) to give product **9** or **10**.

Synthesis of racemic 9 or 10: *Rac*-**9** or **10** was obtained under the catalysis of $Pd(TFA)_2$, (±)-**L7** or (±)-**L13** and Et_3N, respectively.

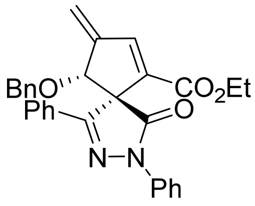

Synthesis of 9a (General procedure E): To an oven-dried 10 mL Schlenk tube equipped with a stir bar were added $Pd(TFA)_2$ (3.3 mg, 0.0099 mmol, 10 mol%), **L10** (4.6 mg, 0.012 mmol, 12 mol%), Et_3N (7.0 μL, 0.050 mmol, 1.0 equiv), ethyl 2-(4-((*tert*-butoxycarbonyl)oxy)-5-oxo-1,3-diphenyl-4,5-dihydro -1*H*-pyrazol-4-yl)acrylate **8a** (45.0 mg, 0.0999 mmol, 1.0 equiv) and ((propa-1,2-dien-1-yloxy)methyl) benzene **2t** (29.2 mg, 0.200 mmol, 2.0 equiv). The tube was evacuated and back-filled with argon for five times. Then degassed DCM (1.0 mL) was added by syringe. The mixture was stirred at 40 °C for 24 h. After completion, the mixture was concentrated and purified by flash chromatography on silica gel (EtOAc/petroleum ether) to give product **9a**: as a colorless oil, 29.1 mg (0.0609 mmol), 61% yield; $[\alpha]_D^{25}$ = +32.8 (c = 0.76, in $CHCl_3$); 91% ee, determined by HPLC analysis [Chiralpak column AD-H, *i*-PrOH/*n*-Hexane = 20/80, flow rate: 1.0 mL/min, 254 nm, t (minor) = 9.94 min, t (major) = 18.65 min]; ^1H NMR (400 MHz, $CDCl_3$) δ (ppm) 8.11–8.02 (m, 2H), 7.88–7.78 (m, 2H), 7.49–7.44 (m, 2H), 7.40–7.33 (m, 4H), 7.25 (s, 2H), 7.23–7.15 (m, 3H), 7.09–7.01 (m, 2H), 5.67 (d, J = 2.6 Hz, 1H),

5.59 (d, J = 2.2 Hz, 1H), 5.22 (t, J = 2.5 Hz, 1H), 4.53 (d, J = 11.6 Hz, 1H), 4.36 (d, J = 11.6 Hz, 1H), 4.07–3.98 (m, 2H), 1.04 (t, J = 7.2 Hz, 3H); ^{13}C NMR (100 MHz, $CDCl_3$) δ (ppm) 174.7, 162.4, 156.9, 150.0, 144.8, 138.2, 136.5, 136.3, 131.4, 130.1, 128.9, 128.4, 128.14, 128.08, 127.6, 125.3, 119.0, 116.7, 87.1, 73.5, 67.9, 61.2, 13.8; HRMS (ESI-TOF) *m/z*: $[M + Na]^+$ Calcd for $C_{30}H_{26}N_2O_4Na^+$ 501.1785; Found 501.1780.

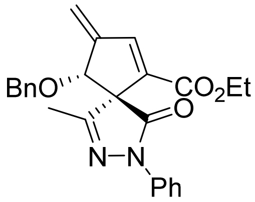

Synthesis of 9b (General procedure E): To an oven-dried 10 mL Schlenk tube equipped with a stir bar were added $Pd(TFA)_2$ (3.3 mg, 0.0099 mmol, 10 mol%), **L10** (4.6 mg, 0.012 mmol, 12 mol%), Et_3N (7.0 μL, 0.050 mmol, 1.0 equiv), ethyl 2-(4-((*tert*-butoxycarbonyl)oxy)-3-methyl-5-oxo-1-phenyl-4,5-dihydro-1*H*-pyrazol-4-yl)acrylate **8b** (45.0 mg, 0.0999 mmol, 1.0 equiv) and ((propa-1,2-dien-1-yloxy)methyl) benzene **2t** (29.2 mg, 0.200 mmol, 2.0 equiv). The tube was evacuated and back-filled with argon for five times. Then degassed DCM (1.0 mL) was added by syringe. The mixture was stirred at 40 °C for 24 h. After completion, the mixture was concentrated and purified by flash chromatography on silica gel (EtOAc/petroleum ether) to give product **9b**: as a colorless oil, 34.0 mg (0.0817 mmol), 82% yield; $[\alpha]_D^{25}$ = –84.3 (c = 1.46, in $CHCl_3$); 91% ee, determined by HPLC analysis [Chiralpak column AD-H, *i*-PrOH/*n*-Hexane = 20/80, flow rate: 1.0 mL/min, 254 nm, t (minor) = 8.81 min, t (major) = 16.43 min]; ^{1}H NMR (400 MHz, $CDCl_3$) δ (ppm) 7.98–7.90 (m, 2H), 7.47–7.39 (m, 2H), 7.38 (s, 1H), 7.32–7.23 (m, 5H), 7.23–7.17 (m, 1H), 5.52 (d, J = 2.8 Hz, 1H), 5.47 (d, J = 1.8 Hz, 1H), 5.13 (t, J = 2.6 Hz, 1H), 4.51 (d, J = 11.3 Hz, 1H), 4.41 (d, J = 11.3 Hz, 1H), 4.17–4.06 (m, 2H), 2.09 (s, 3H), 1.10 (t, J = 7.1 Hz, 3H); ^{13}C NMR (100 MHz, $CDCl_3$) δ (ppm) 174.2, 162.4, 158.9, 148.7, 144.8, 138.2, 136.5, 135.0, 128.9, 128.5, 128.20, 128.16, 125.0, 118.8, 115.3, 86.9, 73.6, 68.1, 61.3, 15.4, 13.9; HRMS (ESI-TOF) *m/z*: $[M + Na]^+$ Calcd for $C_{25}H_{24}N_2O_4Na^+$ 439.1628; Found 439.1630.

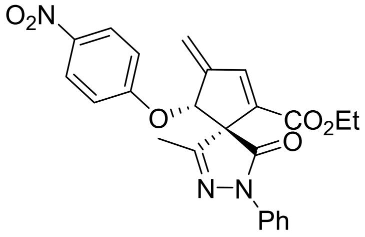

Synthesis of 9c (General procedure E): To an oven-dried 10 mL Schlenk tube equipped with a stir bar were added $Pd(TFA)_2$ (3.3 mg, 0.0099 mmol, 10 mol%), **L10** (4.6 mg, 0.012 mmol, 12 mol%), Et_3N (7.0 μL, 0.050 mmol, 1.0 equiv), ethyl 2-(4-((*tert*-butoxycarbonyl)oxy) -3-methyl-5-oxo-1-phenyl-4,5-dihydro-1*H*-pyrazol-4-yl)acrylate **8b** (45.0 mg, 0.0999 mmol, 1.0 equiv) and 1-nitro-4-(propa-1,2-dien-1-yloxy)benzene **2ac** (35.4 mg, 0.200 mmol, 2.0 equiv). The tube was evacuated and back-filled with argon for five times. Then degassed DCM (1.0 mL) was added by syringe. The mixture was stirred at 40 °C for 24 h. After

completion, the mixture was concentrated and purified by flash chromatography on silica gel (EtOAc/petroleum ether) to give product **9c**: as a yellow oil, 39.3 mg (0.878 mmol), 88% yield; $[\alpha]_D^{25}$ = –38.9 (*c* = 0.75, in $CHCl_3$); 90% ee, determined by HPLC analysis [Chiralpak column IA, *i*-PrOH/*n*-Hexane = 40/60, flow rate: 1.0 mL/min, 254 nm, t (minor) = 10.04 min, t (major) = 12.97 min]; ^{1}H NMR (400 MHz, $CDCl_3$) δ (ppm) 8.10–8.02 (m, 2H), 7.72–7.62 (m, 2H), 7.48 (s, 1H), 7.40–7.31 (m, 2H), 7.22–7.13 (m, 1H), 7.02–6.94 (m, 2H), 5.92 (t, *J* = 2.5 Hz, 1H), 5.69 (d, *J* = 2.7 Hz, 1H), 5.56 (d, *J* = 2.5 Hz, 1H), 4.22–4.08 (m, 2H), 2.09 (s, 3H), 1.13 (t, *J* = 7.1 Hz, 3H); ^{13}C NMR (100 MHz, $CDCl_3$) δ (ppm) 173.3, 161.9, 161.5, 156.8, 146.9, 144.6, 142.6, 137.4, 135.2, 128.9, 126.0, 125.6, 118.9, 116.4, 115.4, 83.8, 67.5, 61.6, 15.3, 13.9; HRMS (ESI-TOF) *m/z*: $[M + Na]^+$ Calcd for $C_{24}H_{21}N_3O_4Na^+$ 470.1323; Found 470.1327.

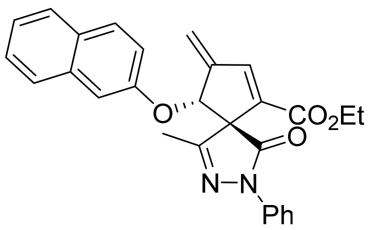

Synthesis of 9d (General procedure E): To an oven-dried 10 mL Schlenk tube equipped with a stir bar were added $Pd(TFA)_2$ (3.3 mg, 0.0099 mmol, 10 mol%), **L10** (4.6 mg, 0.012 mmol, 12 mol%), Et_3N (7.0 μL, 0.050 mmol, 1.0 equiv), ethyl 2-(4-((*tert*-butoxycarbonyl)oxy) -3-methyl-5-oxo-1-phenyl-4,5-dihydro-1*H*-pyrazol-4-yl)acrylate **8b** (45.0 mg, 0.0999 mmol, 1.0 equiv) and 2-(propa-1,2-dien-1-yloxy)naphthalene **2ad** (35.4 mg, 0.200 mmol, 2.0 equiv). The tube was evacuated and back-filled with argon for five times. Then degassed DCM (1.0 mL) was added by syringe. The mixture was stirred at 40 °C for 24 h. After completion, the mixture was concentrated and purified by flash chromatography on silica gel (EtOAc/petroleum ether) to give product **9d**: as a yellow oil, 34.1 mg (0.0754 mmol), 75% yield; $[\alpha]_D^{25}$ = +25.1 (*c* = 1.01 in $CHCl_3$); 91% ee, determined by HPLC analysis [Chiralpak column ID, *i*-PrOH/*n*-Hexane = 40/60, flow rate: 1.0 mL/min, 254 nm, t (minor) = 12.05 min, t (major) = 14.23 min]; ^{1}H NMR (400 MHz, $CDCl_3$) δ (ppm) 7.72–7.64 (m, 2H), 7.62–7.55 (m, 2H), 7.50 (s, 1H), 7.46–7.39 (m, 1H), 7.30–7.21 (m, 4H), 7.16–7.05 (m, 3H), 5.97 (t, *J* = 2.4 Hz, 1H), 5.67 (d, *J* = 2.7 Hz, 1H), 5.63 (d, *J* = 1.9 Hz, 1H), 4.21–4.07 (m, 2H), 2.11 (s, 3H), 1.13 (t, *J* = 7.1 Hz, 3H); ^{13}C NMR (100 MHz, $CDCl_3$) δ (ppm) 173.9, 162.3, 157.3, 154.7, 148.1, 145.0, 137.6, 135.3, 134.1, 129.8, 129.5, 128.5, 127.4, 127.0, 126.4, 125.1, 124.3, 119.2, 118.2, 116.2, 109.1, 84.0, 67.8, 61.4, 15.4, 13.9; HRMS (ESI-TOF) *m/z*: $[M + Na]^+$ Calcd for $C_{28}H_{24}N_2O_4Na^+$ 475.1628; Found 475.1636.

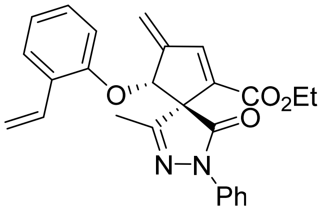

Synthesis of 9e (General procedure E): To an oven-dried 10 mL Schlenk tube equipped with a stir bar were added Pd(TFA)$_2$ (3.3 mg, 0.0099 mmol, 10 mol%), **L10** (4.6 mg, 0.012 mmol, 12 mol%), Et$_3$N (7.0 μL, 0.050 mmol, 1.0 equiv), ethyl 2-(4-((*tert*-butoxycarbonyl)oxy)-3-methyl-5-oxo-1-phenyl-4,5-dihydro-1*H*-pyrazol-4-yl)acrylate **8b** (45.0 mg, 0.0999 mmol, 1.0 equiv) and 1-(propa-1,2-dien-1-yloxy)-2-vinylbenzene **2ae** (31.6 mg, 0.200 mmol, 2.0 equiv). The tube was evacuated and back-filled with argon for five times. Then degassed DCM (1.0 mL) was added by syringe. The mixture was stirred at 40 °C for 24 h. After completion, the mixture was concentrated and purified by flash chromatography on silica gel (EtOAc/petroleum ether) to give product **9e**: as a yellow oil, 37.8 mg (0.0882 mmol), 88% yield; $[\alpha]_D^{25}$ = –69.0 (c = 1.22, in CHCl$_3$); 87% ee, determined by HPLC analysis [Chiralpak column AD-H, *i*-PrOH/*n*-Hexane = 5/95, flow rate: 1.0 mL/min, 254 nm, t (minor) = 39.10 min, t (major) = 44.94 min]; ^{1}H NMR (400 MHz, CDCl$_3$) δ (ppm) 7.77–7.68 (m, 2H), 7.48 (s, 1H), 7.43 (dd, J = 7.6, 1.7 Hz, 1H), 7.39–7.32 (m, 2H), 7.19–7.14 (m, 1H), 7.03–6.94 (m, 2H), 6.88 (t, J = 7.5 Hz, 1H), 6.79 (d, J = 8.3 Hz, 1H), 5.87 (t, J = 2.5 Hz, 1H), 5.70 (dd, J = 17.7, 1.4 Hz, 1H), 5.65 (d, J = 2.7 Hz, 1H), 5.58 (d, J = 1.7 Hz, 1H), 5.26 (dd, J = 11.1, 1.4 Hz, 1H), 4.18–4.09 (m, 2H), 2.08 (s, 3H), 1.13 (t, J = 7.1 Hz, 3H); ^{13}C NMR (100 MHz, CDCl$_3$) δ (ppm) 173.7, 162.2, 157.3, 153.9, 148.2, 144.9, 137.7, 135.3, 130.9, 129.0, 128.7, 127.1, 126.7, 125.2, 122.2, 119.3, 116.1, 115.1, 112.6, 84.1, 67.9, 61.5, 15.4, 13.9; HRMS (ESI-TOF) m/z: $[M + Na]^+$ Calcd for $C_{26}H_{24}N_2O_4Na^+$ 451.1628; Found 451.1632.

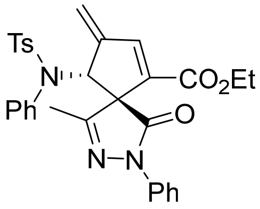

Synthesis of 9f (General procedure E): To an oven-dried 10 mL Schlenk tube equipped with a stir bar were added Pd(TFA)$_2$ (3.3 mg, 0.0099 mmol, 10 mol%), **L10** (4.6 mg, 0.012 mmol, 12 mol%), Et$_3$N (7.0 μL, 0.050 mmol, 1.0 equiv), ethyl 2-(4-((*tert*-butoxycarbonyl)oxy)-3-methyl-5-oxo-1-phenyl-4,5-dihydro-1*H*-pyrazol-4-yl)acrylate **8b** (45.0 mg, 0.0999 mmol, 1.0 equiv) and 4-methyl-*N*-phenyl-*N*-(propa-1,2-dien-1-yl)benzenesulfonamide **2v** (57.1 mg, 0.200 mmol, 2.0 equiv). The tube was evacuated and back-filled with argon for five times. Then degassed DCM (1.0 mL) was added by syringe. The mixture was stirred at 40 °C for 24 h. After completion, the mixture was concentrated and purified by flash chromatography on silica gel (EtOAc/petroleum ether) to give product **9f**: as a yellow oil, 45.1 mg (0.0812 mmol), 81% yield; 5:1 dr, determined by ^{1}H NMR analysis of crude product; $[\alpha]_D^{25}$ = –63.1 (c = 1.29, in CHCl$_3$); 97% ee, determined by HPLC analysis [Chiralpak column IA, *i*-PrOH/*n*-Hexane = 20/80, flow rate: 1.0 mL/min, 254 nm, t (minor) = 11.19 min, t (major)

= 14.56 min]; ^{1}H NMR (400 MHz, $CDCl_3$) δ (ppm) 7.81–7.73 (m, 2H), 7.59–7.52 (m, 2H), 7.45–7.33 (m, 4H), 7.27–7.23 (m, 3H), 7.05–6.93 (m, 4H), 6.26 (t, *J* = 2.8 Hz, 1H), 5.90 (d, *J* = 2.7 Hz, 1H), 5.81 (d, *J* = 3.1 Hz, 1H), 4.14–3.98 (m, 2H), 2.25 (s, 3H), 1.06 (t, *J* = 7.1 Hz, 3H), 0.91 (s, 3H); ^{13}C NMR (100 MHz, $CDCl_3$) δ (ppm) 172.8, 161.7, 157.7, 147.1, 144.5, 143.8, 138.0, 136.8, 136.3, 135.5, 133.3, 129.5, 129.4, 128.8, 128.7, 127.9, 125.1, 118.8, 116.7, 70.6, 68.0, 61.4, 21.5, 14.3, 13.8; HRMS (ESI-TOF) *m/z*: $[M + Na]^+$ Calcd for $C_{31}H_{29}N_3O_5SNa^+$ 578.1720; Found 578.1724.

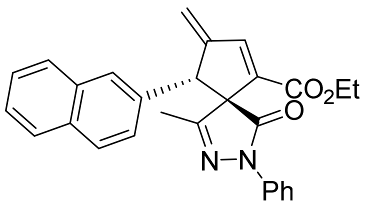

Synthesis of 9g (General procedure E): To an oven-dried 10 mL Schlenk tube equipped with a stir bar were added $Pd(TFA)_2$ (3.3 mg, 0.0099 mmol, 10 mol%), **L10** (4.6 mg, 0.012 mmol, 12 mol%), Et_3N (7.0 μL, 0.050 mmol, 1.0 equiv), ethyl 2-(4-((*tert*-butoxycarbonyl)oxy) -3-methyl-5-oxo-1-phenyl-4,5-dihydro-1*H*-pyrazol-4-yl)acrylate **8b** (45.0 mg, 0.0999 mmol, 1.0 equiv) and 2-(propa-1,2-dien-1-yl)naphthalene **2s** (33.2 mg, 0.200 mmol, 2.0 equiv). The tube was evacuated and back-filled with argon for five times. Then degassed DCM (1.0 mL) was added by syringe. The mixture was stirred at 40 °C for 24 h. After completion, the mixture was concentrated and purified by flash chromatography on silica gel (EtOAc/petroleum ether) to give product **9g**: as a yellow oil, 30.5 mg (0.0699 mmol), 70% yield; 7:1 dr, determined by ^{1}H NMR analysis of crude product; $[\alpha]_D^{25}$ = –110.4 (*c* = 1.29, in $CHCl_3$); 67% ee, determined by HPLC analysis [Chiralpak column IB, *i*-PrOH/*n*-Hexane = 40/60, flow rate: 1.0 mL/min, 254 nm, t (minor) = 5.51 min, t (major) = 6.98 min]; ^{1}H NMR (400 MHz, $CDCl_3$) δ (ppm) 7.86–7.81 (m, 2H), 7.81–7.77 (m, 1H), 7.77–7.71 (m, 3H), 7.61 (s, 1H), 7.49–7.43 (m, 2H), 7.42–7.35 (m, 2H), 7.26–7.24 (m, 1H), 7.22–7.15 (m, 1H), 5.80 (d, *J* = 2.9 Hz, 1H), 5.40 (d, *J* = 1.8 Hz, 1H), 4.97 (t, *J* = 2.8 Hz, 1H), 4.18–4.08 (m, 2H), 1.62 (s, 3H), 1.13 (t, *J* = 7.1 Hz, 3H); ^{13}C NMR (100 MHz, $CDCl_3$) δ (ppm) 174.5, 162.6, 158.7, 149.6, 148.2, 138.0, 135.5, 133.1, 132.9, 132.7, 128.8, 128.6, 127.8, 127.6, 127.5, 126.4, 126.3, 125.1, 119.2, 116.6, 71.0, 61.3, 58.8, 15.5, 13.9; HRMS (ESI-TOF) *m/z*: $[M + Na]^+$ Calcd for $C_{28}H_{24}N_2O_3Na^+$ 459.1679; Found 459.1686.

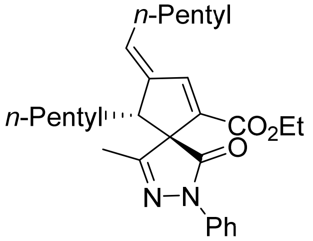

Synthesis of 9h (General procedure E): To an oven-dried 10 mL Schlenk tube equipped with a stir bar were added $Pd(TFA)_2$ (3.3 mg, 0.0099 mmol, 10 mol%), **L13** (4.4 mg, 0.012 mmol, 12 mol%), Et_3N (7.0 μL, 0.050 mmol, 1.0 equiv), ethyl 2-(4-((*tert*-butoxycarbonyl)oxy)-3-methyl-5-oxo-1-phenyl-4,5-dihydro-1*H*-pyrazol-4-yl)acrylate **8b** (45.0 mg, 0.0999 mmol,

1.0 equiv) and trideca-6,7-diene **2a** (36.0 mg, 0.200 mmol, 2.0 equiv). The tube was evacuated and back-filled with argon for five times. Then degassed DCM (1.0 mL) was added by syringe. The mixture was stirred at 40 °C for 24 h. After completion, the mixture was concentrated and purified by flash chromatography on silica gel (EtOAc/petroleum ether) to give product **9h**: as a yellow oil, 33.5 mg (0.0721 mmol), 72% yield; *E/Z* >19:1; $[\alpha]_D^{25}$ = –59.3 (*c* = 1.60, in $CHCl_3$); 57% ee, determined by HPLC analysis [Chiralpak column IA, *i*-PrOH/*n*-Hexane = 5/95, flow rate: 1.0 mL/min, 254 nm, t (minor) = 12.11 min, t (major) = 19.77 min]; ^{1}H NMR (400 MHz, $CDCl_3$) δ (ppm) 7.92–7.87 (m, 2H), 7.67 (s, 1H), 7.43–7.37 (m, 2H), 7.16 (t, *J* = 1.3 Hz, 1H), 5.63–5.56 (m, 1H), 4.10 (q, *J* = 7.1 Hz, 2H), 3.47–3.31 (m, 1H), 2.32–2.25 (m, 2H), 1.96 (s, 3H), 1.48–1.39 (m, 2H), 1.38–1.27 (m, 6H), 1.26–1.14 (m, 6H), 1.10 (t, *J* = 7.1 Hz, 3H), 0.91 (t, *J* = 6.8 Hz, 3H), 0.80–0.75 (m, 3H); ^{13}C NMR (100 MHz, $CDCl_3$) δ (ppm) 174.9, 162.9, 159.2, 143.9, 143.2, 138.3, 135.0, 129.4, 128.8, 124.9, 119.0, 68.1, 61.0, 53.4, 31.7, 31.4, 29.8, 29.4, 28.5, 27.0, 22.5, 22.2, 15.4, 14.0, 13.9, 13.8; HRMS (ESI-TOF) *m/z*: $[M + H]^+$ Calcd for $C_{28}H_{39}N_2O_3^+$ 451.2955; Found 451.2954.

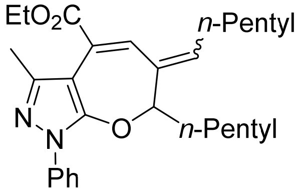

Synthesis of 10 (General procedure E): To an oven-dried 10 mL Schlenk tube equipped with a stir bar were added $Pd(TFA)_2$ (3.3 mg, 0.0099 mmol, 10 mol%), **L14** (4.0 mg, 0.012 mmol, 12 mol%), Et_3N (7.0 μL, 0.050 mmol, 1.0 equiv), ethyl 2-(4-((*tert*-butoxycarbonyl)oxy)-3-methyl-5-oxo-1-phenyl-4,5-dihydro-1*H*-pyrazol-4-yl)acrylate **8b** (45.0 mg, 0.0999 mmol, 1.0 equiv) and trideca-6,7-diene **2a** (36.0 mg, 0.200 mmol, 2.0 equiv). The tube was evacuated and back-filled with argon for five times. Then degassed DCM (1.0 mL) was added by syringe. The mixture was stirred at 40 °C for 24 h. After completion, the mixture was concentrated and purified by flash chromatography on silica gel (EtOAc/petroleum ether) to give product **10**: as a yellow oil, 25.9 mg (0.0575 mmol), 58% yield; *E/Z* = 6:1; $[\alpha]_D^{25}$ = +81.5 (*c* = 1.35, in $CHCl_3$); 61% ee, determined by HPLC analysis [Chiralpak column IA, *i*-PrOH/*n*-Hexane = 5/95, flow rate: 1.0 mL/min, 254 nm, t (major) = 6.35 min, t (minor) = 7.69 min]; ^{1}H NMR (400 MHz, $CDCl_3$) δ (ppm) 7.71–7.64 (m, 2H), 7.43–7.38 (m, 2H), 7.28–7.24 (m, 1H), 6.77 (s, 1H), 5.63 (t, *J* = 7.5 Hz, 1H), 4.67 (t, *J* = 7.4 Hz, 1H), 4.41–4.31 (m, 2H), 2.30–2.21 (m, 5H), 1.71–1.60 (m, 1H), 1.57–1.49 (m, 1H), 1.48–1.42 (m, 2H), 1.39 (t, *J* = 7.1 Hz, 3H), 1.34–1.29 (m, 4H), 1.16–1.06 (m, 6H), 0.92–0.87 (m, 3H), 0.80–0.72 (m, 3H); ^{13}C NMR (100 MHz, $CDCl_3$) δ (ppm) 169.2, 150.7, 146.0, 138.04, 138.03, 134.5, 128.7, 126.3, 125.9, 123.7, 122.1, 101.6, 85.7, 61.4, 32.0, 31.4, 31.1, 28.8, 28.0, 25.3, 22.5, 22.3, 15.0, 14.2, 14.0, 13.8; HRMS (ESI-TOF) *m/z*: $[M + H]^+$ Calcd for $C_{28}H_{39}N_2O_3^+$ 451.2995; Found 451.2993.

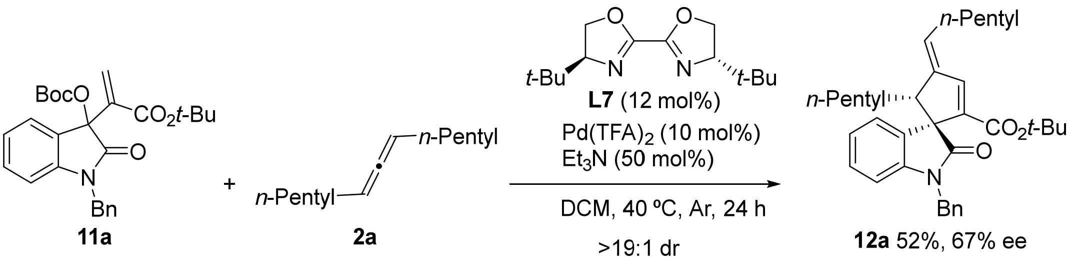

Synthesis of 12a (General procedure E): To an oven-dried 10 mL Schlenk tube equipped with a stir bar were added Pd(TFA)$_2$ (3.3 mg, 0.0099 mmol, 10 mol%), **L7** (4.0 mg, 0.012 mmol, 12 mol%), Et$_3$N (7.0 μL, 0.050 mmol, 1.0 equiv), *tert*-butyl 2-(1-benzyl-3-((*tert*-butoxycarbonyl) oxy)-2-oxoindolin-3-yl)acrylate **11a** (46.5 mg, 0.0999 mmol, 1.0 equiv) and trideca-6,7-diene **2a** (36.0 mg, 0.200 mmol, 2.0 equiv). The tube was evacuated and back-filled with argon for five times. Then degassed DCM (1.0 mL) was added by syringe. The mixture was stirred at 40 °C for 24 h. After completion, the mixture was concentrated and purified by flash chromatography on silica gel (EtOAc/petroleum ether) to give product **12a**: as a as a colorless oil, 27.6 mg (0.0523 mmol), 52% yield; $[\alpha]_D^{25}$ = +22.8 (*c* = 0.40, in CHCl$_3$); 67% ee, determined by HPLC analysis [Chiralpak column AD-H, *i*-PrOH/*n*-Hexane = 10/90, flow rate: 1.0 mL/min, 254 nm, t (minor) = 8.52 min, t (major) = 10.21 min]; ^{1}H NMR (400 MHz, CDCl$_3$) δ (ppm) 7.57 (s, 1H), 7.45–7.41 (m, 2H), 7.33–7.29 (m, 2H), 7.28–7.26 (m, 1H), 7.18–7.13 (m, 1H), 6.95–6.89 (m, 1H), 6.87 (dd, *J* = 7.5, 1.5 Hz, 1H), 6.77 (d, *J* = 7.8 Hz, 1H), 5.56–5.45 (m, 1H), 5.28 (d, *J* = 15.4 Hz, 1H), 4.55 (d, *J* = 15.3 Hz, 1H), 3.49–3.32 (m, 1H), 2.36–2.24 (m, 2H), 1.47–1.42 (m, 2H), 1.36–1.30 (m, 5H), 1.20 (s, 9H), 1.14–1.08 (m, 1H), 1.01–0.80 (m, 9H), 0.58 (t, *J* = 6.9 Hz, 4H); ^{13}C NMR (100 MHz, CDCl$_3$) δ (ppm) 179.4, 162.9, 144.4, 143.3, 141.6, 140.1, 136.1, 129.5, 128.6, 128.3, 128.1, 128.0, 127.5, 124.3, 121.8, 108.5, 80.8, 62.2, 52.7, 44.4, 31.8, 31.5, 29.77, 29.75, 29.6, 27.8, 26.9, 22.5, 21.9, 14.1, 13.7; HRMS (ESI-TOF) m/z: $[M + H]^+$ Calcd for $C_{35}H_{46}NO_3^+$ 528.3472; Found 528.3471.

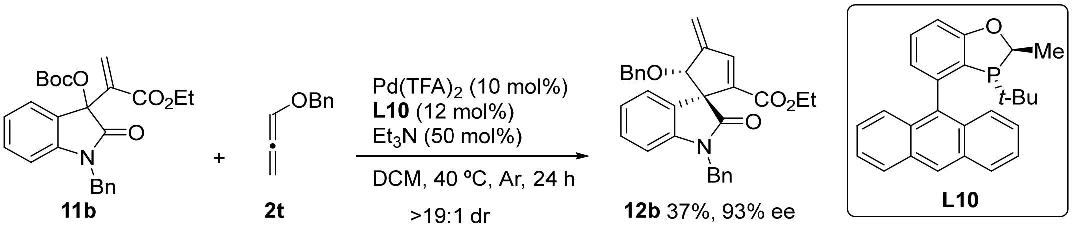

Synthesis of 12b (General procedure E): To an oven-dried 10 mL Schlenk tube equipped with a stir bar were added Pd(TFA)$_2$ (3.3 mg, 0.0099 mmol, 10 mol%), **L10** (4.0 mg, 0.012 mmol, 12 mol%), Et$_3$N (7.0 μL, 0.050 mmol, 1.0 equiv), ethyl 2-(1-benzyl-3-((*tert*-butoxycarbonyl)oxy)-2-

oxoindolin-3-yl) acrylate **12b** (43.7 mg, 0.0999 mmol, 1.0 equiv) and ((propa-1,2-dien-1-yloxy)methyl)benzene **2t** (36.0 mg, 0.200 mmol, 2.0 equiv). The tube was evacuated and back-filled with argon for five times. Then degassed DCM (1.0 mL) was added by syringe. The mixture was stirred at 40 °C for 24 h. After completion, the mixture was concentrated and purified by flash chromatography on silica gel (EtOAc/petroleum ether) to give product **12b**: as a colorless oil, 17.2 mg (0.0369 mmol), 37% yield; $[\alpha]_D^{25}$ = +44.6 (*c* = 0.92, in $CHCl_3$); 93% ee, determined by HPLC analysis [Chiralpak column IA, *i*-PrOH/*n*-Hexane = 20/80, flow rate: 1.0 mL/min, 254 nm, t (minor) = 9.79 min, t (major) = 12.35 min]; ^{1}H NMR (400 MHz, $CDCl_3$) δ (ppm) 7.46–7.42 (m, 2H), 7.36 (s, 1H), 7.31–7.26 (m, 3H), 7.24–7.22 (m, 1H), 7.20–7.17 (m, 3H), 7.09 (d, *J* = 6.7 Hz, 1H), 6.99 (t, *J* = 7.6 Hz, 1H), 6.84–6.77 (m, 3H), 5.54 (d, *J* = 2.6 Hz, 1H), 5.47 (d, *J* = 2.2 Hz, 1H), 5.10 (t, *J* = 2.4 Hz, 1H), 5.04 (d, *J* = 15.5 Hz, 1H), 4.93 (d, *J* = 15.5 Hz, 1H), 4.12–4.03 (m, 2H), 3.99–3.88 (m, 2H), 1.05 (t, *J* = 7.1 Hz, 3H); ^{13}C NMR (100 MHz, $CDCl_3$) δ (ppm) 178.3, 162.9, 149.7, 143.14, 143.11, 138.7, 136.9, 135.9, 128.69, 128.65, 128.2, 127.9, 127.75, 127.73, 127.65, 126.8, 125.4, 122.5, 115.2, 109.0, 85.9, 72.7, 62.6, 60.7, 44.4, 13.9; HRMS (ESI-TOF) *m/z*: $[M + H]^+$ Calcd for $C_{30}H_{28}NO_4^+$ 466.2013; Found 466.2009.

5. Synthetic transformations

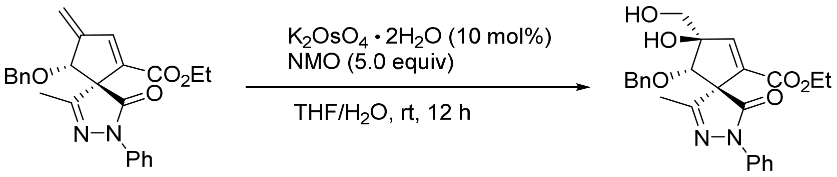

9b 91% ee

13 58%, 94% ee
8:1 dr

To a solution of **9b** (41.6 mg, 0.0999 mmol, 1.0 equiv) in THF/H_2O (0.8 mL/0.2 mL) were added $K_2OsO_4 \cdot 2H_2O$ (3.7 mg, 0.010 mmol, 10 mol%) and 4-methylmorpholine *N*-oxide (NMO, 58.5 mg, 0.499 mmol, 5.0 equiv). The mixture was stirred at rt for 12 h. After complete consumption of **9b** (monitored by TLC), the mixture was diluted with saturated aqueous sodium thiosulfate, extracted with EtOAc (3.0 mL), the organic layers were dried over Na_2SO_4 and concentrated. The residue was purified by flash chromatography on silica gel (EtOAc/petroleum ether = 1/1) to give the product **13**: as a colorless oil, 26.1 mg (0.0579 mmol), 58% yield; 8:1 dr, determined by ^{1}H NMR analysis; $[\alpha]_D^{25}$ = –118.0 (*c* = 1.91, in $CHCl_3$); 94% ee, determined by HPLC analysis [Chiralpak column AD-H, *i*-PrOH/*n*-Hexane = 40/60, flow rate: 1.0 mL/min, 254 nm, t (major) = 5.01 min, t (minor) = 5.86 min]; ^{1}H NMR (400 MHz, $CDCl_3$) δ (ppm) 7.97–7.91 (m, 2H), 7.48–7.42 (m, 2H), 7.37–7.29 (m, 3H),

7.27–7.22 (m, 3H), 7.09 (s, 1H), 4.58 (s, 1H), 4.50 (d, J = 3.1 Hz, 2H), 4.20 (s, 1H), 4.14 (q, J = 7.2 Hz, 2H), 4.07 (dd, J = 11.8, 4.1 Hz, 1H), 3.82 (dd, J = 11.7, 7.6 Hz, 1H), 2.60 (t, J = 6.7 Hz, 1H), 2.20 (s, 3H), 1.17 (t, J = 7.1 Hz, 3H); ^{13}C NMR (100 MHz, $CDCl_3$) δ (ppm) 173.8, 161.9, 158.7, 148.6, 137.8, 135.9, 134.1, 128.9, 128.7, 128.6, 128.2, 125.4, 119.0, 93.4, 85.7, 74.8, 69.0, 64.6, 61.6, 17.2, 13.8; HRMS (ESI-TOF) *m/z*: $[M + H]^+$ Calcd for $C_{25}H_{27}N_2O_6^+$ 451.1864; Found 451.1861. *The relative configuration of **13** was determind by NOEDS NMR analysis.*

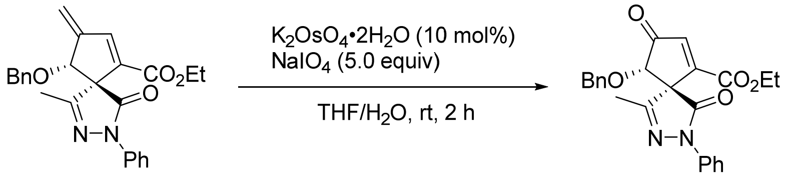

9b 91% ee

14 59%, 90% ee

To a solution of **9b** (41.6 mg, 0.0999 mmol, 1.0 equiv) in THF/H_2O (0.8 mL/0.2 mL) were added $K_2OsO_4 \cdot 2H_2O$ (3.7 mg, 0.010 mmol, 10 mol%) and $NaIO_4$ (108.0 mg, 0.5000 mmol, 5.0 equiv). The mixture was stirred at rt for 2 h. After complete consumption of **9b** (monitored by TLC), the mixture was quenched by water, extracted with EtOAc (3.0 mL), the organic layers were dried over Na_2SO_4 and concentrated. The residue was purified by flash chromatography on silica gel (EtOAc/petroleum ether = 1/10) to give the product **14**: as a yellow oil, 24.7 mg (0.0590 mmol), 59% yield; $[\alpha]_D^{25}$ = +65.6 (c = 0.31, in $CHCl_3$); 90% ee, determined by HPLC analysis [Chiralpak column IA, *i*-PrOH/*n*-Hexane = 40/60, flow rate: 1.0 mL/min, 254 nm, t (minor) = 6.44 min, t (major) = 9.08 min]; ^{1}H NMR (400 MHz, $CDCl_3$) δ (ppm) 7.92–7.83 (m, 2H), 7.47–7.41 (m, 2H), 7.26–7.19 (m, 6H), 7.12 (s, 1H), 4.74 (d, J = 11.9 Hz, 1H), 4.64 (d, J = 11.9 Hz, 1H), 4.60 (s, 1H), 4.28–4.12 (m, 2H), 2.05 (s, 3H), 1.16 (t, J = 7.1 Hz, 3H); ^{13}C NMR (100 MHz, $CDCl_3$) δ (ppm) 201.6, 171.5, 161.1, 156.8, 155.6, 138.9, 137.8, 135.7, 128.9, 128.6, 128.5, 128.1, 125.4, 118.8, 85.1, 74.2, 65.1, 62.8, 15.5, 13.8; HRMS (ESI-TOF) *m/z*: $[M + Na]^+$ Calcd for $C_{24}H_{22}N_2O_5Na^+$ 441.1421; Found 441.1424.

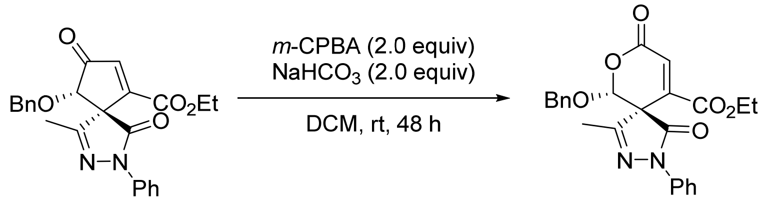

14 90% ee

15 65%, 90% ee

To a solution of **14** (20.9 mg, 0.0499 mmol, 1.0 equiv) in DCM (1.0 mL) were added 3-chloroperbenzoic acid (*m*-CPBA, 17.2 mg, 0.0997 mmol, 2.0 equiv) and $NaHCO_3$ (8.4 mg, 0.10 mmol, 2.0 equiv). The mixture was stirred at rt for 48 h. After complete consumption of **14** (monitored by

TLC), the mixture was concentrated and purified by flash chromatography on silica gel (EtOAc/petroleum ether = 1/10) to give the product **15**: as a colorless oil, 14.1 mg (0.0650 mmol), 65% yield; $[\alpha]_D^{25}$ = +48.4 (*c* = 0.34, in $CHCl_3$); 90% ee, determined by HPLC analysis [Chiralpak column IA, *i*-PrOH/*n*-Hexane = 40/60, flow rate: 1.0 mL/min, 254 nm, t (minor) = 6.85 min, t (major) = 19.65 min]; ^1H NMR (400 MHz, $CDCl_3$) δ (ppm) 7.87–7.79 (m, 2H), 7.45–7.39 (m, 2H), 7.27–7.22 (m, 4H), 7.20–7.15 (m, 2H), 7.11 (s, 1H), 5.64 (s, 1H), 4.98 (d, *J* = 11.9 Hz, 1H), 4.71 (d, *J* = 11.9 Hz, 1H), 4.26–4.15 (m, 2H), 2.14 (s, 3H), 1.16 (t, *J* = 7.1 Hz, 3H); ^{13}C NMR (100 MHz, $CDCl_3$) δ (ppm) 169.6, 161.8, 160.8, 154.4, 140.6, 137.6, 134.8, 129.1, 128.9, 128.6, 128.5, 128.0, 125.6, 119.0, 101.6, 72.2, 63.1, 59.9, 15.6, 13.7; HRMS (ESI-TOF) *m/z*: $[M + Na]^+$ Calcd for $C_{24}H_{22}N_2O_6Na^+$ 457.1370; Found 457.1374.

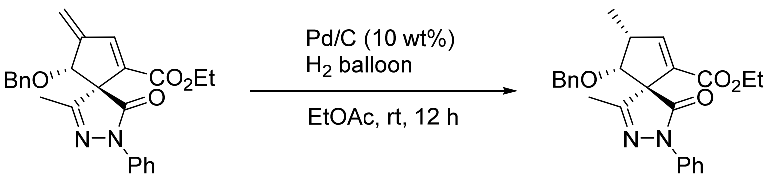

9b 91% ee

16 61%, 90% ee

To a solution of **9b** (41.6 mg, 0.0999 mmol, 1.0 equiv) in EtOAc (1.0 mL) was added Pd/C (4.2 mg, 10 wt%). The resultant mixture was degassed, charged with hydrogen balloon and stirred at rt for 12 h. After complete consumption of **9b** (monitored by TLC), the mixture was concentrated and purified by flash chromatography on silica gel (EtOAc/petroleum ether = 1/10) to give the product **16**: as a yellow oil, 25.4 mg (0.0607 mmol), 61% yield; $[\alpha]_D^{25}$ = –137.9 (*c* = 0.39, in $CHCl_3$); 90% ee, determined by HPLC analysis [Chiralpak column AD-H, *i*-PrOH/*n*-Hexane = 40/60, flow rate: 1.0 mL/min, 254 nm, t (major) = 10.61 min, t (minor) = 12.02 min];^1H NMR (400 MHz, $CDCl_3$) δ (ppm) 7.98–7.87 (m, 2H), 7.45–7.38 (m, 2H), 7.28–7.25 (m, 3H), 7.23–7.17 (m, 3H), 7.16 (d, *J* = 2.7 Hz, 1H), 4.78 (d, *J* = 8.3 Hz, 1H), 4.40 (s, 2H), 4.08 (q, *J* = 7.1 Hz, 2H), 3.41–3.27 (m, 1H), 2.19 (s, 3H), 1.24 (d, *J* = 7.5 Hz, 3H), 1.09 (t, *J* = 7.1 Hz, 3H); ^{13}C NMR (100 MHz, $CDCl_3$) δ (ppm) 175.3, 162.3, 158.8, 153.2, 138.3, 137.0, 130.9, 128.8, 128.4, 127.9, 127.5, 124.9, 118.8, 87.0, 73.1, 69.2, 61.1, 43.1, 17.0, 14.2, 13.9; HRMS (ESI-TOF) *m/z*: $[M + Na]^+$ Calcd for $C_{25}H_{26}N_2O_4Na^+$ 441.1785; Found 441.1794. *The relative configuration of* ***16*** *was determind by 2D NMR analysis.*

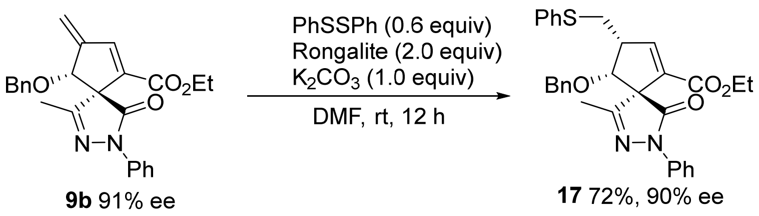

To a solution of **9b** (41.6 mg, 0.0999 mmol, 1.0 equiv) in DMF (1.0 mL) were added PhSSPh (13.1 mg, 0.0601 mmol, 0.6 equiv), sodium hydroxymethanesulphinate (Rongalite, 31.0 mg, 0.199 mmol, 2.0 equiv) and K_2CO_3 (13.8 mg, 0.0998 mmol, 1.0 equiv). The resultant mixture was stirred at rt for 12 h. After complete consumption of **9b** (monitored by TLC), the mixture was concentrated and purified by flash chromatography on silica gel (EtOAc/petroleum ether = 1/10) to give the product **17**: as a yellow oil, 37.8 mg (0.0718 mmol), 72% yield; $[\alpha]_D^{25}$ = –10.3 (*c* = 0.58, in MeCN); 90% ee, determined by HPLC analysis [Chiralpak column AD-H, *i*-PrOH/*n*-Hexane = 40/60, flow rate: 1.0 mL/min, 254 nm, t (minor) = 7.11 min, t (minor) = 7.69 min]; ^{1}H NMR (400 MHz, $CDCl_3$) δ (ppm) 7.97–7.92 (m, 2H), 7.44–7.39 (m, 2H), 7.38–7.34 (m, 2H), 7.33–7.26 (m, 5H), 7.23–7.17 (m, 4H), 7.09 (d, *J* = 2.0 Hz, 1H), 4.55 (d, *J* = 7.6 Hz, 1H), 4.39 (d, *J* = 11.2 Hz, 1H), 4.27 (d, *J* = 11.1 Hz, 1H), 4.11–4.02 (m, 2H), 3.37–3.29 (m, 1H), 3.23 (dd, *J* = 13.1, 5.3 Hz, 1H), 2.96 (dd, *J* = 13.1, 8.7 Hz, 1H), 2.14 (s, 3H), 1.09 (t, *J* = 7.1 Hz, 3H); ^{13}C NMR (100 MHz, $CDCl_3$) δ (ppm) 174.3, 161.8, 158.7, 148.1, 138.2, 136.5, 135.4, 132.8, 129.7, 129.1, 128.9, 128.5, 128.21, 128.20, 126.6, 125.0, 118.8, 90.5, 73.4, 68.6, 61.2, 51.4, 35.5, 15.8, 13.8; HRMS (ESI-TOF) *m/z*: $[M + Na]^+$ Calcd for $C_{31}H_{30}N_2O_4SNa^+$ 549.1818; Found 549.1828. *The relative configuration of **17** was determind by NOEDS NMR analysis.*

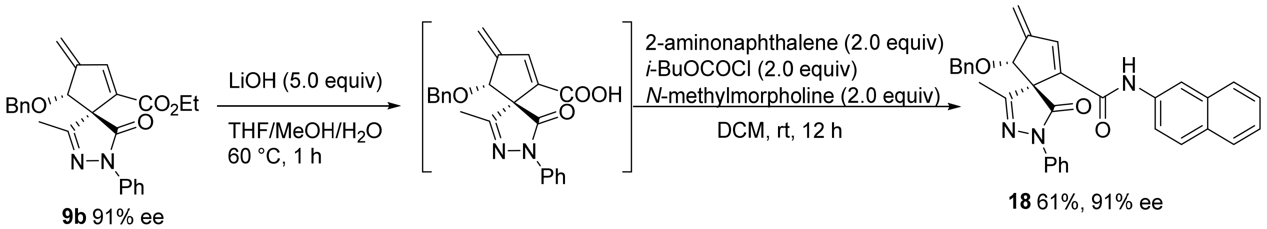

To a solution of **9b** (41.6 mg, 0.0999 mmol, 1.0 equiv) in THF/MeOH/H_2O (1.0 mL/ 0.5 mL/ 0.5 mL) was added LiOH (12.0 mg, 0.500 mmol, 5.0 equiv). Then the resulting mixture was stirred at 60 °C for 1 h. After complete consumption of **9b** (monitored by TLC), the mixture was quench by *conc.* HCl and extracted with EtOAc (3.0 mL). The combined organic layers were washed with brine, dried over Na_2SO_4, filtered, and concentrated in vacuo. The residue was used in next step without further purification.

The residue was dissolved in DCM (2 mL), followed by the sequential addition of *i*-BuOCOCl (26 μL, 0.20 mmol, 2.0 equiv) and *N*-methylmorpholine (22 μL, 0.20 mmol, 2.0 equiv) at 0 °C. The reaction mixture was stirred for 30 minutes, after which 2-aminonaphthalene (28.6 mg, 0.200 mmol, 2.0 equiv) was added. The mixture was then stirred at room temperature for 12 h. After complete reaction as monitored by TLC, the mixture was concentrated and purified by flash chromatography on silica gel (EtOAc/petroleum ether = 4/1) to give the product **18**: as a yellow solid, 31.3 mg (0.0609 mmol), 61% yield; mp 124–125 °C; $[\alpha]_D^{25}$ = +94.0 (*c* = 1.02, in $CHCl_3$); 91% ee, determined by HPLC analysis [Chiralpak column IA, *i*-PrOH/*n*-Hexane = 40/60, flow rate: 1.0 mL/min, 254 nm, t (minor) = 4.86 min, t (minor) = 6.35 min]; ^{1}H NMR (400 MHz, $CDCl_3$) δ (ppm) 8.93 (s, 1H), 8.12 (d, *J* = 7.8 Hz, 2H), 7.62 (s, 1H), 7.51 (t, *J* = 8.0 Hz, 2H), 7.45 (d, *J* = 8.2 Hz, 1H), 7.40–7.29 (m, 7H), 7.21–7.12 (m, 3H), 7.11–7.02 (m, 1H), 6.88 (d, *J* = 8.2 Hz, 1H), 5.18 (d, *J* = 2.4 Hz, 1H), 4.97–4.82 (m, 2H), 4.67 (d, *J* = 11.9 Hz, 1H), 4.34 (d, *J* = 11.9 Hz, 1H), 2.13 (s, 3H); ^{13}C NMR (100 MHz, $CDCl_3$) δ (ppm) 176.4, 161.3, 160.7, 149.0, 139.9, 138.7, 138.1, 136.3, 134.8, 133.3, 130.2, 129.0, 128.7, 128.54, 128.46, 127.7, 127.6, 127.0, 125.7, 125.5, 124.4, 119.64, 119.61, 116.7, 113.9, 85.3, 73.1, 69.9, 15.4.; HRMS (ESI-TOF) *m/z*: $[M + H]^+$ Calcd for $C_{33}H_{28}N_3O_3^+$ 514.2125; Found 514.2120.

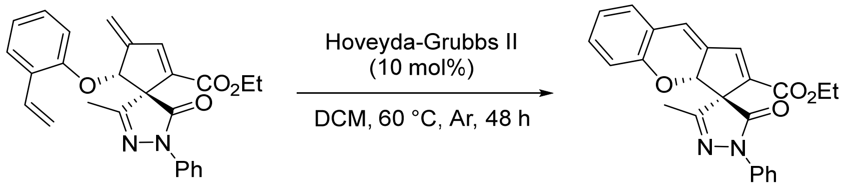

9e 87% ee

19 83%, 91% ee

To a solution of **9e** (42.8 mg, 0.0999 mmol, 1.0 equiv) in DCM (1.0 mL) was added Hoveyda-Grubbs II (6.3 mg, 0.010 mmol, 10 mol%). Then the resulting mixture was degassed, and charged with Ar and stirred at 60 °C for 48 h. After complete consumption of **9e** (monitored by TLC), the mixture was concentrated and purified by flash chromatography on silica gel (EtOAc/petroleum ether = 10/1) to give the product **19**: as a yellow oil, 33.3 mg (0.0832 mmol), 83% yield; $[\alpha]_D^{25}$ = –89.8 (*c* = 1.02, in $CHCl_3$); 91% ee, determined by HPLC analysis [Chiralpak column IB, *i*-PrOH/*n*-Hexane = 40/60, flow rate: 1.0 mL/min, 254 nm, t (minor) = 5.18 min, t (major) = 6.70 min]; ^{1}H NMR (400 MHz, $CDCl_3$) δ (ppm) 8.06–7.94 (m, 2H), 7.55 (s, 1H), 7.46–7.40 (m, 2H), 7.23–7.16 (m, 3H), 7.03–6.97 (m, 1H), 6.90 (d, *J* = 8.0 Hz, 1H), 6.78 (d, *J* = 2.4 Hz, 1H), 5.39 (d, *J* = 2.4 Hz, 1H), 4.21–4.09 (m, 2H), 2.04 (s, 3H), 1.12 (t, *J* = 7.1 Hz, 3H); ^{13}C NMR (100 MHz, $CDCl_3$) δ (ppm) 173.0, 162.4, 157.1, 153.4, 141.5, 138.2, 135.64, 135.57, 130.5, 128.8, 128.4, 125.0, 122.8, 122.6, 122.5, 118.7, 117.0, 81.7, 67.2, 61.5, 15.2, 13.9; HRMS (ESI-TOF) *m/z*: $[M + Na]^+$ Calcd for $C_{24}H_{20}N_2O_4Na^+$

423.1315; Found 423.1321.

6. More substrate exploration

To construct diverse frameworks, more types of MBH carbonates, allenes and alkenes were investigated under palladium catalysis. Unfortunately, no obvious conversions or complex reaction profiles were generally observed, as outlined below.

6.1 Exploration of more MBH carbonates

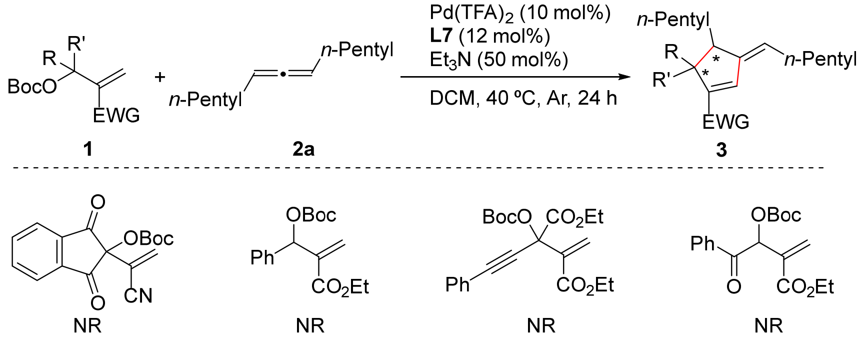

6.2 Exploration of more allenes and alkenes

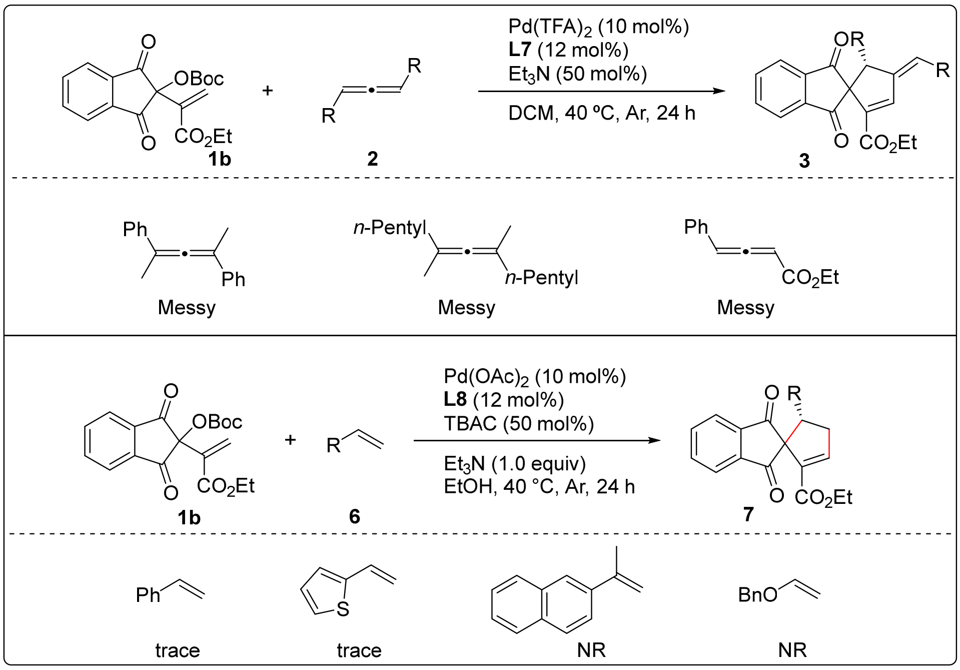

7. Crystal, ECD spectra data and structural refinements

7.1 Crystal data and structural refinement for enantiopure 3q

Preparation of the single crystals of enantiopure **3q**: **3q** (20.0 mg) was dissolved in THF (1.0 mL) and *n*-hexane (2.0 mL). The mixture was heated until a clear solution was formed, which was

kept aside at room temperature to obtain crystals. The crystals were subjected to the single crystal X-ray diffraction analysis for the determination of the absolute configuration of **3q**. The data were collected by a Bruker APEX-II CCD equipped with a Cu radiation source (Kα = 1.54178 Å) at 150.0 K. CCDC 2473163 (**3q**) contains the supplementary crystallographic data for this paper. These data can be obtained free of charge via www.ccdc.cam.ac.uk/data_request/cif.

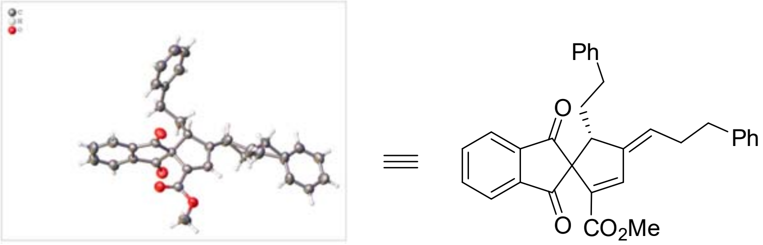

(ellipsoid contour probability 50%)

Identification code	**3q**
Empirical formula	$C_{32}H_{28}O_4$
Formula weight	476.54
Temperature/K	150.0
Crystal system	orthorhombic
Space group	$P2_12_12_1$
a/Å	10.7281(7)
b/Å	11.3273(8)
c/Å	20.3145(14)
α/°	90
β/°	90
γ/°	90
Volume/Å^3	2468.6(3)
Z	4
ρ_{calc}g/cm^3	1.282
μ/mm^{-1}	0.666
F(000)	1008.0
Crystal size/mm^3	0.5 × 0.19 × 0.18
Radiation	CuKα (λ = 1.54178)
2Θ range for data collection/°	8.706 to 137.09
Index ranges	-12 ≤ h ≤ 12, -13 ≤ k ≤ 13, -24 ≤ l ≤ 24

Reflections collected	100443
Independent reflections	4532 [R_{int} = 0.0598, R_{sigma} = 0.0207]
Data/restraints/parameters	4532/31/345
Goodness-of-fit on F^2	1.036
Final R indexes [I>=2σ (I)]	R_1 = 0.0349, wR_2 = 0.0949
Final R indexes [all data]	R_1 = 0.0352, wR_2 = 0.0951
Largest diff. peak/hole / e Å^{-3}	0.19/-0.21
Flack parameter	0.01(4)

7.2 Crystal data and structural refinement for enantiopure 4e

Preparation of the single crystals of enantiopure **4e**: **4e** (20.0 mg) was dissolved in THF (1.0 mL) and *n*-hexane (2.0 mL). The mixture was heated until a clear solution was formed, which was kept aside at room temperature to obtain crystals. The crystals were subjected to the single crystal X-ray diffraction analysis for the determination of the absolute configuration of **4e**. The data were collected by a Bruker APEX-II CCD equipped with a Mo radiation source (Kα = 0.71073 Å) at 302.0 K. CCDC 2473164 (**4e**) contains the supplementary crystallographic data for this paper. These data can be obtained free of charge via www.ccdc.cam.ac.uk/data_request/cif.

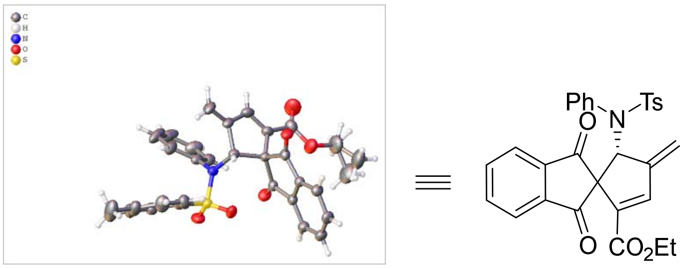

(ellipsoid contour probability 50%)

Identification code	**4e**
Empirical formula	$C_{30}H_{25}NO_6S$
Formula weight	527.57
Temperature/K	302.0
Crystal system	trigonal
Space group	$P3_2$
a/Å	16.834(5)
b/Å	16.834(5)

c/Å	8.235(3)
α/°	90
β/°	90
γ/°	120
Volume/Å^3	2021.1(14)
Z	3
ρ_{calc}g/cm^3	1.300
μ/mm^{-1}	0.164
F(000)	828.0
Crystal size/mm^3	0.35 × 0.06 × 0.05
Radiation	MoKα (λ = 0.71073)
2Θ range for data collection/°	4.84 to 55.294
Index ranges	-21 ≤ h ≤ 21, -21 ≤ k ≤ 21, -10 ≤ l ≤ 9
Reflections collected	16041
Independent reflections	5967 [R_{int} = 0.0842, R_{sigma} = 0.0990]
Data/restraints/parameters	5967/19/356
Goodness-of-fit on F^2	1.029
Final R indexes [I>=2σ (I)]	R_1 = 0.0537, wR_2 = 0.1122
Final R indexes [all data]	R_1 = 0.0795, wR_2 = 0.1281
Largest diff. peak/hole / e Å^{-3}	0.35/-0.27
Flack parameter	0.02(8)

7.3 Crystal data and structural refinement for enantiopure 7a

Preparation of the single crystals of enantiopure **7a**: **7a** (20.0 mg) was dissolved in THF (1.0 mL) and *n*-hexane (2.0 mL). The mixture was heated until a clear solution was formed, which was kept aside at room temperature to obtain crystals. The crystals were subjected to the single crystal X-ray diffraction analysis for the determination of the absolute configuration of **7a**. The data were collected by a Bruker APEX-II CCD equipped with a Cu radiation source (Kα = 1.54178 Å) at 150.0 K. CCDC 2473165 (**7a**) contains the supplementary crystallographic data for this paper. These data can be obtained free of charge via www.ccdc.cam.ac.uk/data_request/cif.

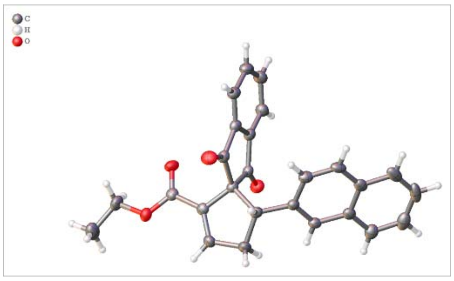

≡

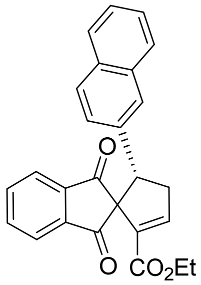

(ellipsoid contour probability 50%)

Identification code	**7a**
Empirical formula	$C_{26}H_{20}O_4$
Formula weight	396.42
Temperature/K	150.0
Crystal system	monoclinic
Space group	$P2_1$
a/Å	8.9065(8)
b/Å	6.1518(6)
c/Å	18.3134(17)
α/°	90
β/°	97.437(4)
γ/°	90
Volume/Å^3	994.97(16)
Z	2
ρ_{calc}g/cm^3	1.323
μ/mm^{-1}	0.717
F(000)	416.0
Crystal size/mm^3	0.38 × 0.07 × 0.05
Radiation	CuKα (λ = 1.54178)
2Θ range for data collection/°	4.866 to 144.35
Index ranges	$-9 \le h \le 10$, $-7 \le k \le 6$, $-21 \le l \le 22$
Reflections collected	7482
Independent reflections	3539 [R_{int} = 0.0424, R_{sigma} = 0.0454]
Data/restraints/parameters	3539/1/272
Goodness-of-fit on F^2	1.089
Final R indexes [I>=2σ (I)]	R_1 = 0.0381, wR_2 = 0.1009

Final R indexes [all data]	R_1 = 0.0407, wR_2 = 0.1024
Largest diff. peak/hole / e Å^{-3}	0.23/-0.20
Flack parameter	-0.02(15)

7.4 Crystal data and structural refinement for enantiopure 18

Preparation of the single crystals of enantiopure **18**: **18** (20.0 mg) was dissolved in toluene (1.0 mL) and *n*-hexane (2.0 mL). The mixture was heated until a clear solution was formed, which was kept aside at room temperature to obtain crystals. The crystals were subjected to the single crystal X-ray diffraction analysis for the determination of the absolute configuration of **18**. The data were collected by a Bruker APEX-II CCD equipped with a Cu radiation source (Kα = 1.54178 Å) at 151.0 K. CCDC 2473166 (**18**) contains the supplementary crystallographic data for this paper. These data can be obtained free of charge via www.ccdc.cam.ac.uk/data_request/cif.

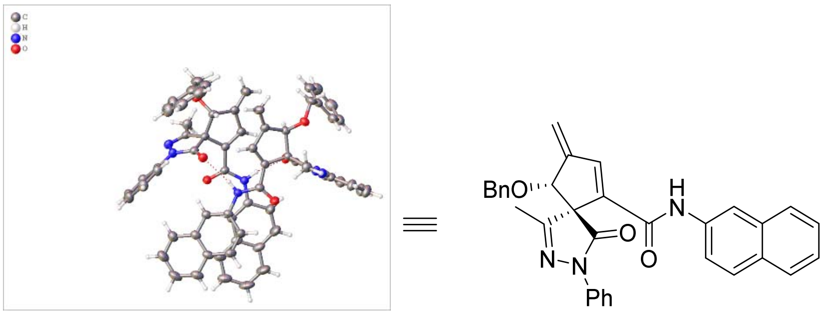

Identification code	**18**
Empirical formula	$C_{33}H_{27}N_3O_3$
Formula weight	513.57
Temperature/K	151.0
Crystal system	orthorhombic
Space group	$C222_1$
a/Å	17.8476(5)
b/Å	27.8909(8)
c/Å	23.5021(7)
α/°	90
β/°	90
γ/°	90
Volume/Å^3	11699.0(6)

Z	16
ρ_{calc}g/cm^3	1.166
μ/mm^{-1}	0.604
F(000)	4320.0
Crystal size/mm^3	0.43 × 0.35 × 0.32
Radiation	CuKα (λ = 1.54178)
2Θ range for data collection/°	5.878 to 144.076
Index ranges	-20 ≤ h ≤ 15, -29 ≤ k ≤ 33, -28 ≤ l ≤ 26
Reflections collected	39736
Independent reflections	10831 [R_{int} = 0.0732, R_{sigma} = 0.0449]
Data/restraints/parameters	10831/0/705
Goodness-of-fit on F^2	1.052
Final R indexes [I>=2σ (I)]	R_1 = 0.0349, wR_2 = 0.0892
Final R indexes [all data]	R_1 = 0.0390, wR_2 = 0.0920
Largest diff. peak/hole / e Å^{-3}	0.13/-0.19
Flack parameter	0.14(9)

7.5 Structural refinement for 12b

The absolute configuration of compound **12b** was determined by derivatisation to **20**.

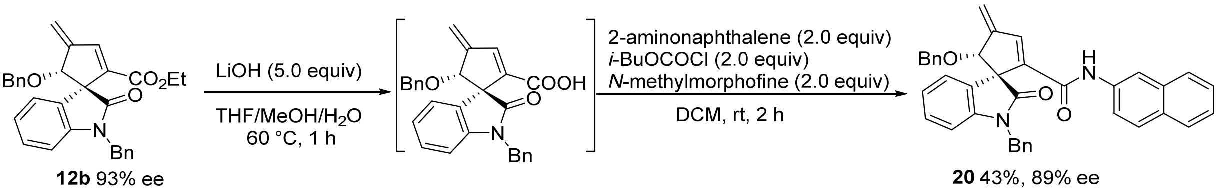

To a solution of **12b** (46.6 mg, 0.100 mmol, 1.0 equiv) in THF/MeOH/H_2O (1.0 mL/0.5 mL/0.5 mL) was added LiOH (12.0 mg, 0.500 mmol, 5.0 equiv). The resultant mixture was stirred at 60 °C for 1 h. After complete consumption of **12b** (monitored by TLC), the mixture was quenched by *conc.* HCl and and extracted with EtOAc. The combined organic layers were washed with brine, dried over Na_2SO_4, filtered, and concentrated in vacuo. The residue was used in next step without further purification.

To the solution of above residue in DCM (2 mL) were added *i*-BuOCOCl (26 μL, 0.20 mmol, 2.0 equiv) and *N*-methylmorphofine (22 μL, 0.20 mmol, 2.0 equiv) at 0 °C. The mixture was stirred for 30 min before 2-aminonaphthalene (28.6 mg, 0.200 mmol, 2.0 equiv) was added, and stirred at rt for additional 12 h. The reaction mixture was concentrated and purified by flash chromatography on

silica gel (EtOAc/petroleum ether = 4/1) to give the product **20**: as a yellow solid, 20.2 mg (0.0434 mmol), 43% yield; mp 115–117 °C; $[\alpha]_D^{25}$ = –15.8 (*c* = 0.81, in $CHCl_3$); 89% ee, determined by HPLC analysis [Chiralpak column IA, *i*-PrOH/*n*-Hexane = 40/60, flow rate: 1.0 mL/min, 254 nm, t (major) = 7.42 min, t (minor) = 8.36 min]; ^{1}H NMR (400 MHz, $CDCl_3$) δ (ppm) 8.36 (s, 1H), 7.93 (s, 1H), 7.60 (d, *J* = 7.9 Hz, 1H), 7.54–7.47 (m, 2H), 7.42–7.38 (m, 1H), 7.36–7.31 (m, 2H), 7.28–7.19 (m, 10H), 7.14 (d, *J* = 7.2 Hz, 1H), 7.02 (t, *J* = 7.6 Hz, 1H), 6.88–6.81 (m, 3H), 5.36–5.25 (m, 2H), 5.20 (d, *J* = 15.7 Hz, 1H), 5.07–4.97 (m, 2H), 4.04 (d, *J* = 11.3 Hz, 1H), 3.99 (d, *J* = 11.3 Hz, 1H); ^{13}C NMR (100 MHz, $CDCl_3$) δ (ppm) 179.2, 161.0, 149.6, 142.8, 137.8, 136.9, 135.6, 135.0, 133.5, 130.4, 128.8, 128.3, 128.2, 128.0, 127.9, 127.63, 127.60, 127.2, 126.0, 125.1, 124.6, 122.9, 119.8, 116.7, 114.1, 109.6, 85.5, 72.6, 63.7, 44.5; HRMS (ESI-TOF) *m/z*: $[M + H]^+$ Calcd for $C_{38}H_{31}N_2O_3^+$ 563.2329; Found 563.2327.

Preparation of the single crystals of **20**: **20** (20.0 mg) was dissolved in toluene (1.0 mL) and *n*-hexane (2.0 mL). The mixture was heated until a clear solution was formed, which was kept aside at room temperature to obtain crystals. The crystals were subjected to the single crystal X-ray diffraction analysis for the determination of the relative configuration of **20**. The data were collected by a Bruker APEX-II CCD equipped with a Cu radiation source (Kα = 1.54178 Å) at 200.0 K. CCDC 2473167 (**20**) contains the supplementary crystallographic data for this paper. These data can be obtained free of charge via www.ccdc.cam.ac.uk/data_request/cif.

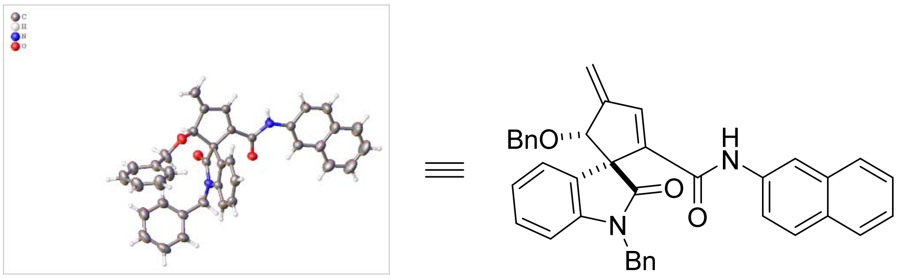

Identification code	**20**
Empirical formula	$C_{38}H_{30}N_2O_3$
Formula weight	562.64
Temperature/K	200.0
Crystal system	orthorhombic
Space group	Pbca
a/Å	20.9949(14)
b/Å	10.6889(7)

c/Å	26.4893(18)
α/°	90
β/°	90
γ/°	90
Volume/Å^3	5944.5(7)
Z	8
ρ_{calc}g/cm^3	1.257
μ/mm^{-1}	0.632
F(000)	2368.0
Crystal size/mm^3	0.5 × 0.22 × 0.05
Radiation	CuKα (λ = 1.54178)
2Θ range for data collection/°	7.892 to 136.804
Index ranges	-25 ≤ h ≤ 25, -12 ≤ k ≤ 12, -31 ≤ l ≤ 31
Reflections collected	125013
Independent reflections	5444 [R_{int} = 0.0697, R_{sigma} = 0.0216]
Data/restraints/parameters	5444/0/388
Goodness-of-fit on F^2	1.055
Final R indexes [I>=2σ (I)]	R_1 = 0.0396, wR_2 = 0.1066
Final R indexes [all data]	R_1 = 0.0437, wR_2 = 0.1104
Largest diff. peak/hole / e Å^{-3}	0.22/-0.18

ECD spectrum and structural refinement for enantioenriched 20

It was not successful to obtain suitable single crystals of enantiopure **20** for X-ray analysis. However, relative configuration of **20** has been determined by X-ray analysis. As a result, the absolute configuration of **20** was assigned by the quantum chemical calculations of electronic circular dichroism (ECD). In this work, all geometry optimizations were carried out using Gaussian 09.[3] Geometries of minima were optimized using the B3LYP-D3 functional[4] with basis set 6-31G(d) in gas phase. Vibrational frequency calculations were performed for all the stationary points to confirm if each optimized structure is a local minimum, as well as deriving the thermochemical corrections for the enthalpies and free energies. The integration grids defined by the 'Int=Ultrafine' keyword were used for all calculations.

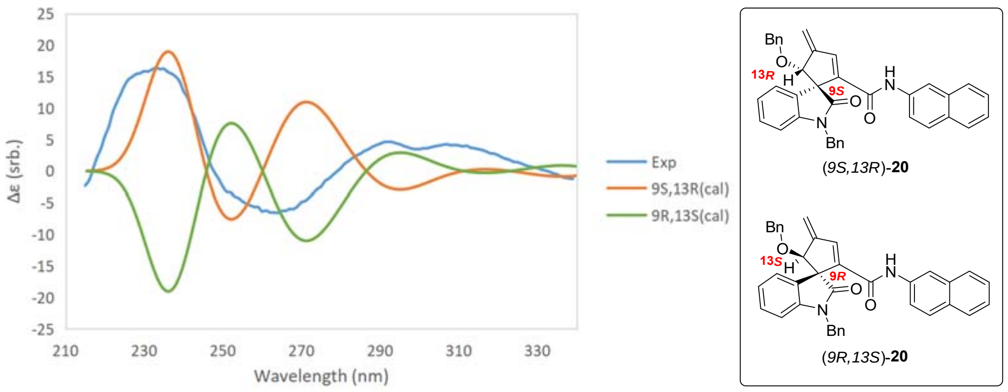

Figure S1. The ECD spectra of compound **20** (exp), calculated Boltzmann-averaged ECD spectra of (*9S,13R*)-**20** *[9S,13R* (calc)], and calculated Boltzmann-averaged ECD spectra of (*9R,13S*)-**20** [*9R, 13S* (calc)].

As depicted in Figure S1, the experimental ECD spectrum of enantioenriched **20** matches quite well with the calculated one of (*9S,13R*)-**20**. Therefore, the stereogenic center of **20** is probably in (*9S*,*13R*)-configuration.

8. Mechanism investigations

8.1 Kinetic study

High yield was still attained when 1.0 equivalent of racemic allene **2a** was used. In addition, the recovered allene **2h** remained racemic throughout the reaction. These results collectively indicate that kinetic resolution of the racemic allene (±)-**2** is not involved in the current (3+2) annulations.

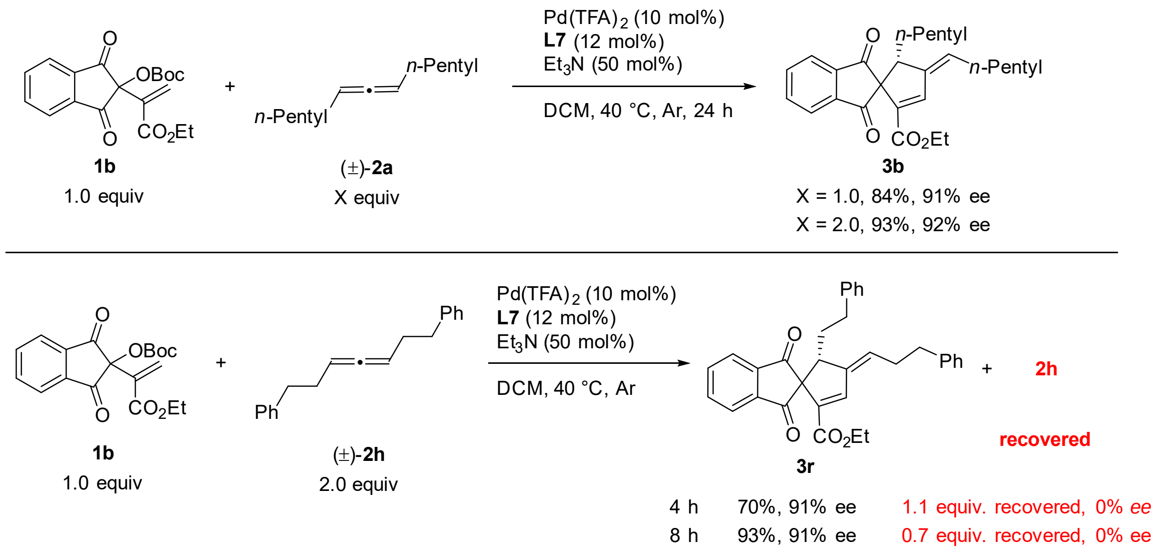

8.2 Computational details

In this work all geometry optimisations and single-point energy calculations were carried out using Gaussian 09.[3] Geometries of intermediates and transition states were optimized using the

B3LYP-D3 functional[4] with basis set 6-31G(d) (SDD for Pd atom) in the gas phase. Vibrational frequency calculations were performed for all the stationary points to confirm if each optimized structure is a local minimum or a transition state structure, as well as deriving the thermochemical corrections for the enthalpies and free energies. Solvation energy corrections were calculated in DCM solvent with the SMD continuum solvation model[5] based on the gas-phase optimized geometries. To gain more accurate results, the B3LYP-D3 functional with a large basis set of 6-311++G(d,p) (SDD for Pd atom) was used for solvation single-point energy calculations. The integration grids defined by the Int=Ultrafine keyword were used for all calculations.

8.3 Calculated results

8.3.1 Comparison of different reaction pathways

To further elucidate the catalytic mechanism for this Pd^0-catalysed (3+2) annulation reaction, DFT calculations were conducted. As proposed in Figure S2a, the key π-allylpalladium complex **INT3** may be generated from oxidative addition of Pd^0 to MBH carbonate **1b** followed by migration insertion into simplified allene **2a'**. On the other hand, allene **2a'** might also be activated by Pd^0 via η^2-coordination and backdonation based on a Dewar−Chatt−Duncanson model, which is indeed supported by density functional theory (DFT) calculations. It is found that the HOMO (highest occupied molecular orbital) energy of Pd^0-η^2-complex **INT6** (−4.79 eV) is significantly enhanced compared to that of allene **2a'** (−6.87 eV) (Figure S2b). The nucleophilicity-enhanced **INT6** may attack MBH carbonate **1b** to form π-allylpalladium complex **INT3** in a S$_N$2' fashion.

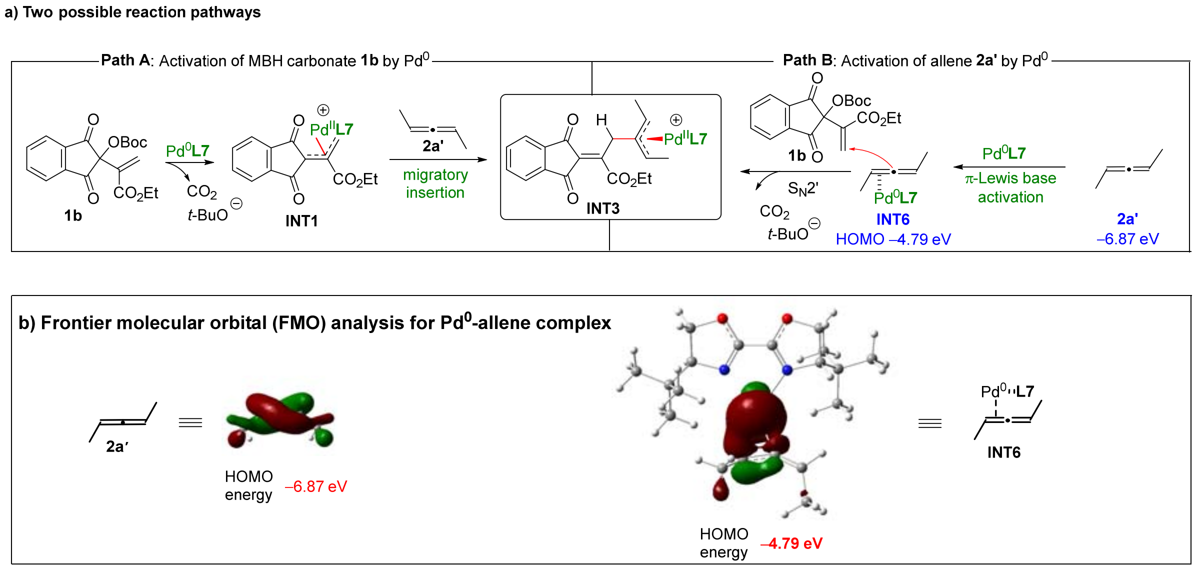

Figure S2. Possible reaction pathways and FMO analysis of Pd^0-allene complex

To figure out which reaction pathway is more favorable, comprehensive DFT calculations were conducted. As shown in Figure S3 (black line), the energy barrier of oxidative addition of Pd^0 and MBH carbonate **1b** via **TS1** is 15.5 kcal/mol. Notably, the pendent carbonyl group of 1,3-indandione

serves as an additional binding group to facilitate the isomerization of η^3-allylpalladium complex **INT1** to slightly more stable η^1-one **INT2**. Subsequent migratory insertion of **INT2** into allene **2a'** via **TS2**, with a free energy barrier of 22.0 kcal/mol, constitutes the rate-determining step and is feasible under current reaction conditions. Although the ester moiety can also serve as a binding group to form **INT2'**, but the energy barrier for subsequent migratory insertion into allene **2a'** via **TS2'** is apparently higher than that of **TS2** (25.8 vs 22.0 kcal/mol, red line of Figure S3), because of apparent steric hindrance between ester group and the 1,3-indandione skeleton in **TS2'**, as noted in Figure S4. In contrast, the attack of HOMO-raised Pd^0-η^2-complex **INT6** on MBH carbonate **1b** via **TS4** features a significantly higher energy barrier of 31.0 kcal/mol (Figure S3, blue line), indicating it is dynamically infeasible at current reaction temperature. Consequently, the proposed oxidative addition/migratory insertion process is more favorable for the initial assembly of two partners.

The strong electron-withdrawing effect of both 1,3-indandione and allylpalladium complex render the β′-H of **INT3** is highly acidic, which can be easily deprotonated by in situ generated *t*-butoxide anion to deliver the more stable intermediate **INT4** with a significant exotherm of 35.0 kcal/mol. In addition, an outer-sphere allylic alkylation via **TS3**, with an energy barrier of only 5.2 kcal/mol, can smoothly occur to provide product **3b'** after ligand exchange.

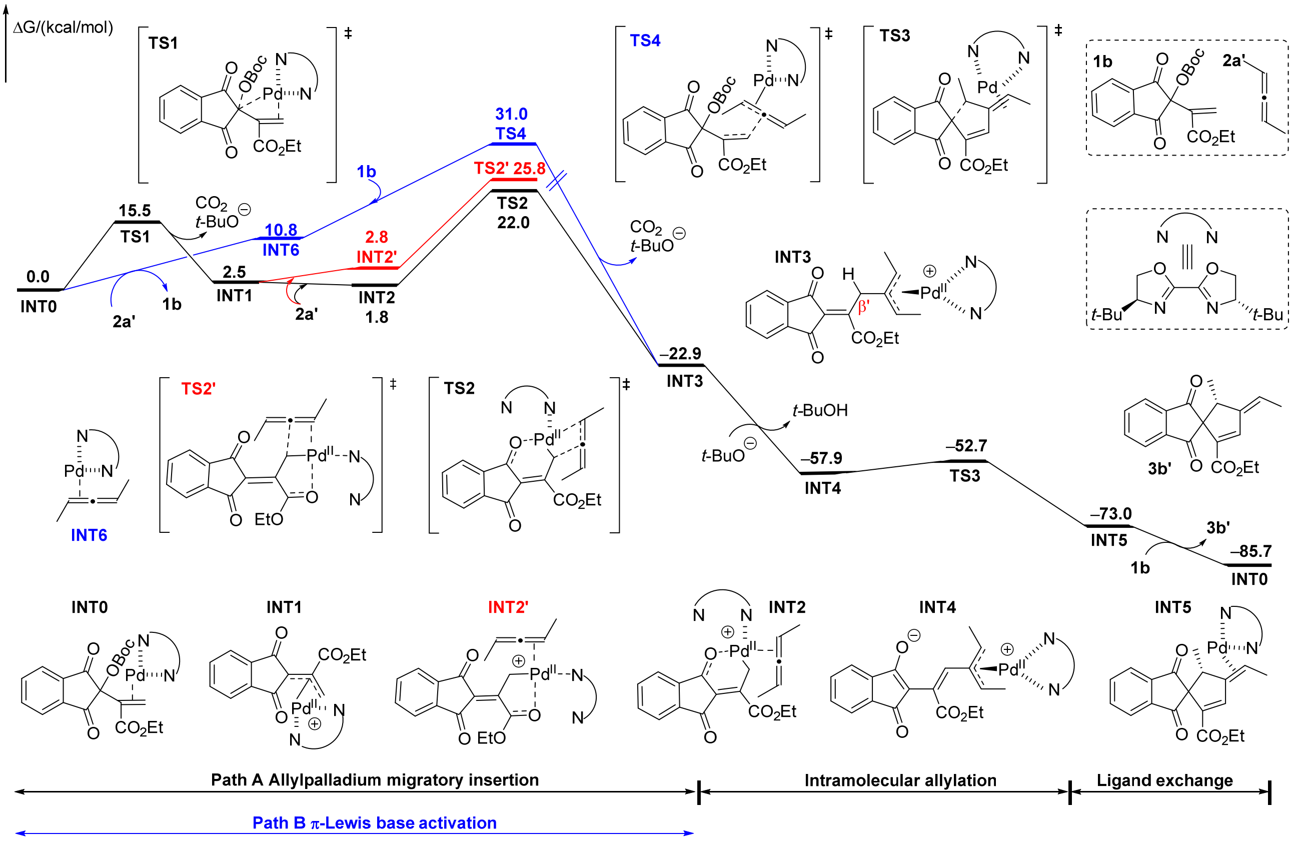

Figure S3. Free energy profiles for the competitive pathways

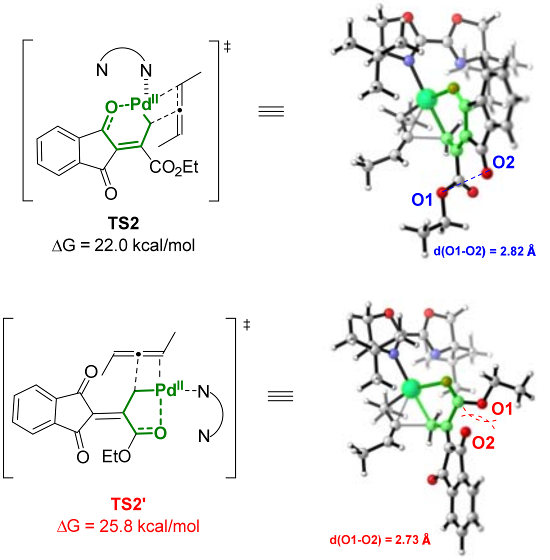

Figure S4. Comparison of the free energy of migratory insertion via different coordination modes

As shown in Figure S5, we further investigated the key intramolecular Tsuji–Trost reaction of **INT4** by considering both reductive elimination and inner-sphere allylation pathways. The reductive elimination proceeds via **TS3-RE**, which exhibits a higher energy barrier (22.3 kcal/mol) than **TS3**. Additionally, computational studies on the inner-sphere allylation pathway revealed that the transition states for *syn*- and *anti*-attack geometries (**TS3-inner-*syn*** and **TS3-inner-*anti***) are 19.2 and 16.0 kcal/mol higher in energy, respectively, relative to that of **TS3**.

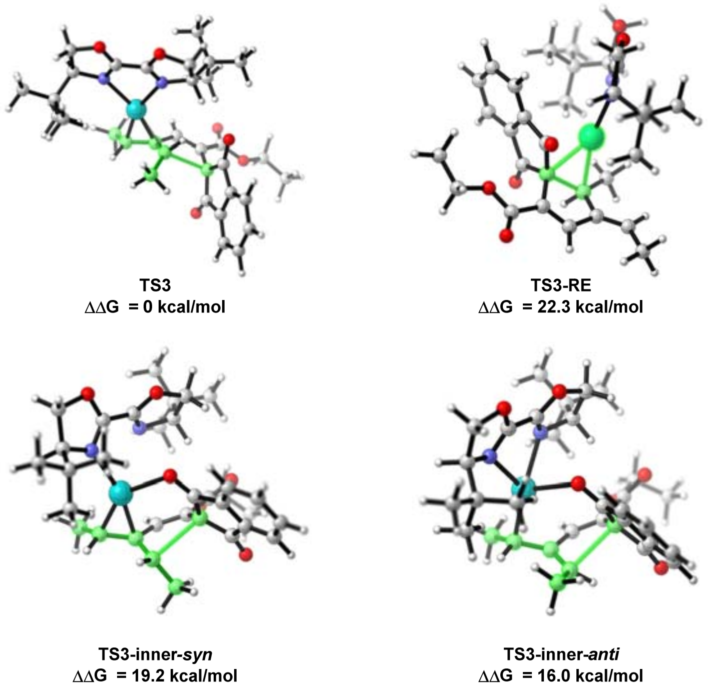

Figure S5. Reductive elimination and inner-sphere allylation modes for the intramolecular Tsuji–Trost reaction

8.3.2 Stereoselectivity analysis

8.3.2.1 Analysis of the conformational of η^3-allylpalladium complex INT4

Calculated results indicates that the allylpalladium migratory insertion is the rate-determining step in the catalytic cycle. However, due to the π-σ-π isomerization of allylpalladium species **INT4**,[6] the enantioselective determining step is intramolecular allylation event (**TS3**). The four-quadrant graph of **INT4** is shown in Figure S6, which reveals that the *t*-Bu group and the methyl group are in different quadrants, making **INT4** (*syn*, *anti*) as the most stable π-allylpalladium isomer.

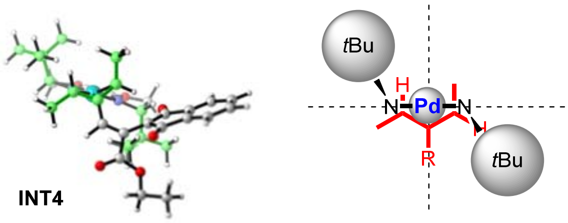

Figure S6. Four quadrant graph of **INT4**

8.3.2.2 Origins of stereoselectivity for the annulation reaction of 1b and 2a′

The origin of stereoselectivity was studied on the model reaction of allene **2a′** and MBH substrate **1b**.

Enantioselective regulation: The related free energy of ***ent*-TS3** is 2.2 kcal/mol higher than that of **TS3**. It suggests the (*R*)-**3b′** should be majorly obtained, due to the steric effect of ligands and corroborating with the experimental observation. Geometry analysis indicates the distance between H^1 and H^2 in **TS3** (2.15 Å) is longer than that between H^3 and H^4 in ***ent*-TS3** (2.07 Å), indicating a significant 1,3-strain between adjacent H atom and CH_3 moiety in ***ent*-TS3**, which leads to its high related free energy (Figure S7). In addition, the regioselectivity of nucleophilic attack process was compared. In fact, the product via **TS3′** would be less favored since a higher energy (8.0 kcal/mol) was observed in transition state **TS3′** in comparison with **TS3**.

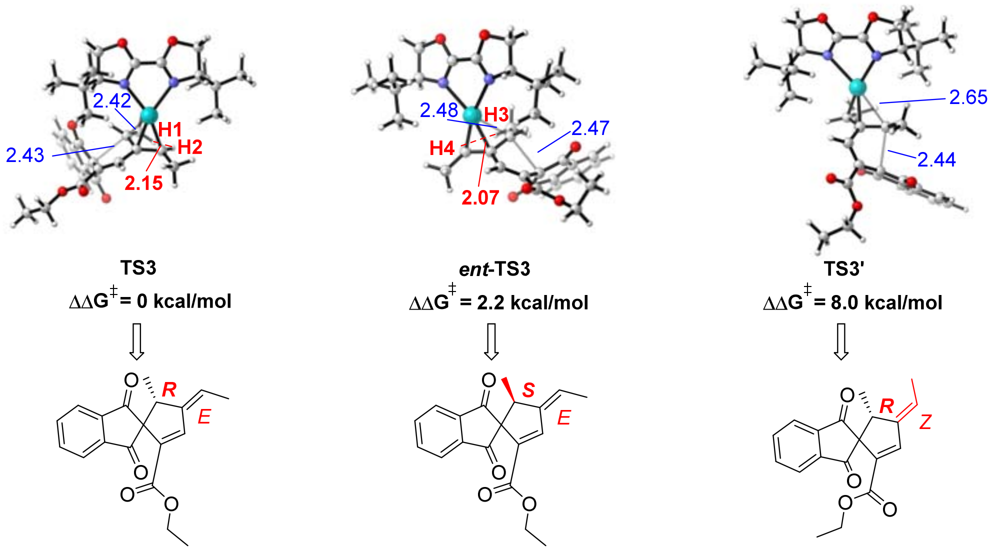

Figure S7. Origins of stereoselectivity for the annulation reaction of **1b** and **2a′**

9. References

[1] (*a*) Y. M. Chung, Y. J. Im and J. N. Kim, *Bull. Korean Chem. Soc.*, 2002, **23**, 1651–1654; (*b*) P. Chen, Z.-C. Chen, Y. Li, Q. Ouyang, W. Du and Y.-C. Chen, *Angew. Chem., Int. Ed.*, 2019, **58**, 4036–4040.

[2] R.-J. Yan, Y. Hu, L. Zhu, J. Zhang, Q. Wang, J.-Y. Huang, Z.-C. Chen, Q. Ouyang, W. Du and Y.-C. Chen, *ACS Catal.*, 2024, **14**, 12824−12832.

[3] M. J. Frisch, G. W. Trucks, H. B. Schlegel, G. E. Scuseria, M. A. Robb, J. R. Cheeseman, G. Scalmani, V. Barone, B. Mennucci, G. A. Petersson, H. Nakatsuji, M. Caricato, X. Li, H. P. Hratchian, A. F. Izmaylov, J. Bloino, G. Zheng, J. L. Sonnenberg, M. Hada, M. Ehara, K. Toyota, R. Fukuda, J. Hasegawa, M. Ishida, T. Nakajima, Y. Honda, O. Kitao, H. Nakai, T. Vreven, J. A., Jr. Montgomery, J. E. Peralta, F. Ogliaro, M. Bearpark, J. J. Heyd, E. Brothers, K. N. Kudin, V. N. Staroverov, R. Kobayashi, J. Normand, K. Raghavachari, A. Rendell, J. C. Burant, S. S. Iyengar, J. Tomasi, M. Cossi, N. Rega, J. M. Millam, M. Klene, J. E. Knox, J. B. Cross, V. Bakken, C. Adamo, J. Jaramillo, R. Gomperts, R. E. Stratmann, O. Yazyev, A. J. Austin, R. Cammi, C. Pomelli, J. W. Ochterski, R. L. Martin, K. Morokuma, V. G. Zakrzewski, G. A. Voth, P. Salvador, J. J. Dannenberg, S. Dapprich, A. D. Daniels, O. Farkas, J. B. Foresman, J. V. Ortiz, J. Cioslowski and D. J. Fox, Gaussian 09, revision D.01; Gaussian, Inc.: Wallingford, CT, **2009**.

[4] (*a*) A. D. Becke, *Phys. Rev. A*, 1988, **38***,* 3098−3100; (*b*) A. D. Becke, *J. Chem. Phys.*, 1993, **98**, 5648−5652; (c) S. Grimme, S. Ehrlich and L. Goerigk, *J. Comput. Chem.*, 2011, **32**, 1456−1465.

[5] A. V. Marenich, C. J. Cramer and D. G. Truhlar, *J. Phys. Chem. B*, 2009, **113**, 6378−6396.

[6] (*a*) J. Liu, Z. Dong, W.-B. Cao, C. Zheng and S.-L. You, *J. Am. Chem. Soc.*, 2025, **147**, 2776−2785; (*b*) P. Li, E. Zheng, G. Li, Y. Luo, X. Huo, S. Ma and W. Zhang, *Science*, 2024, **385**, 972−979; (*c*) R. W. Hoffmann, *Chem. Rev.*, 1989, **89**, 1841−1860.

10. NMR, HRMS spectra and HPLC chromatograms

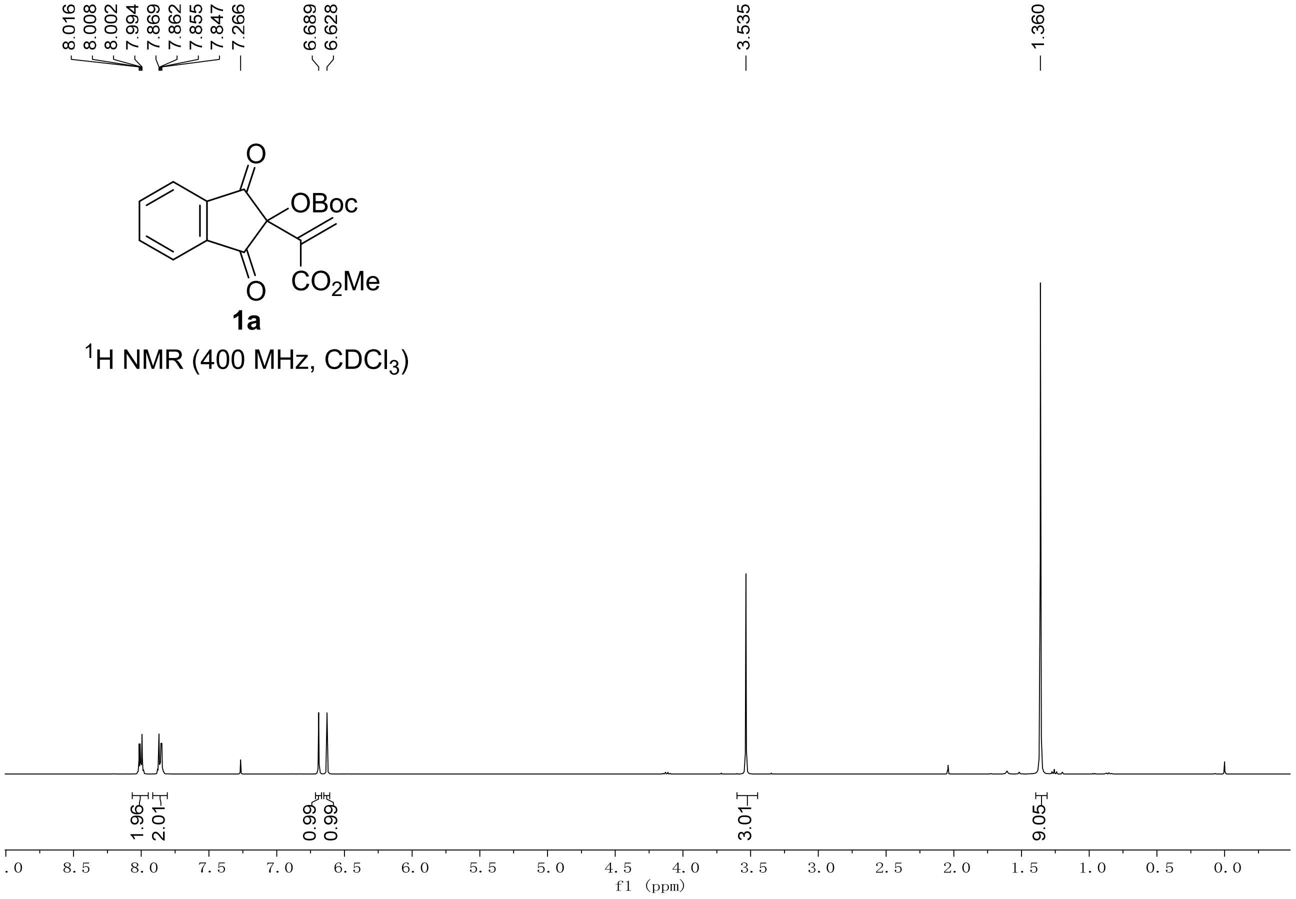

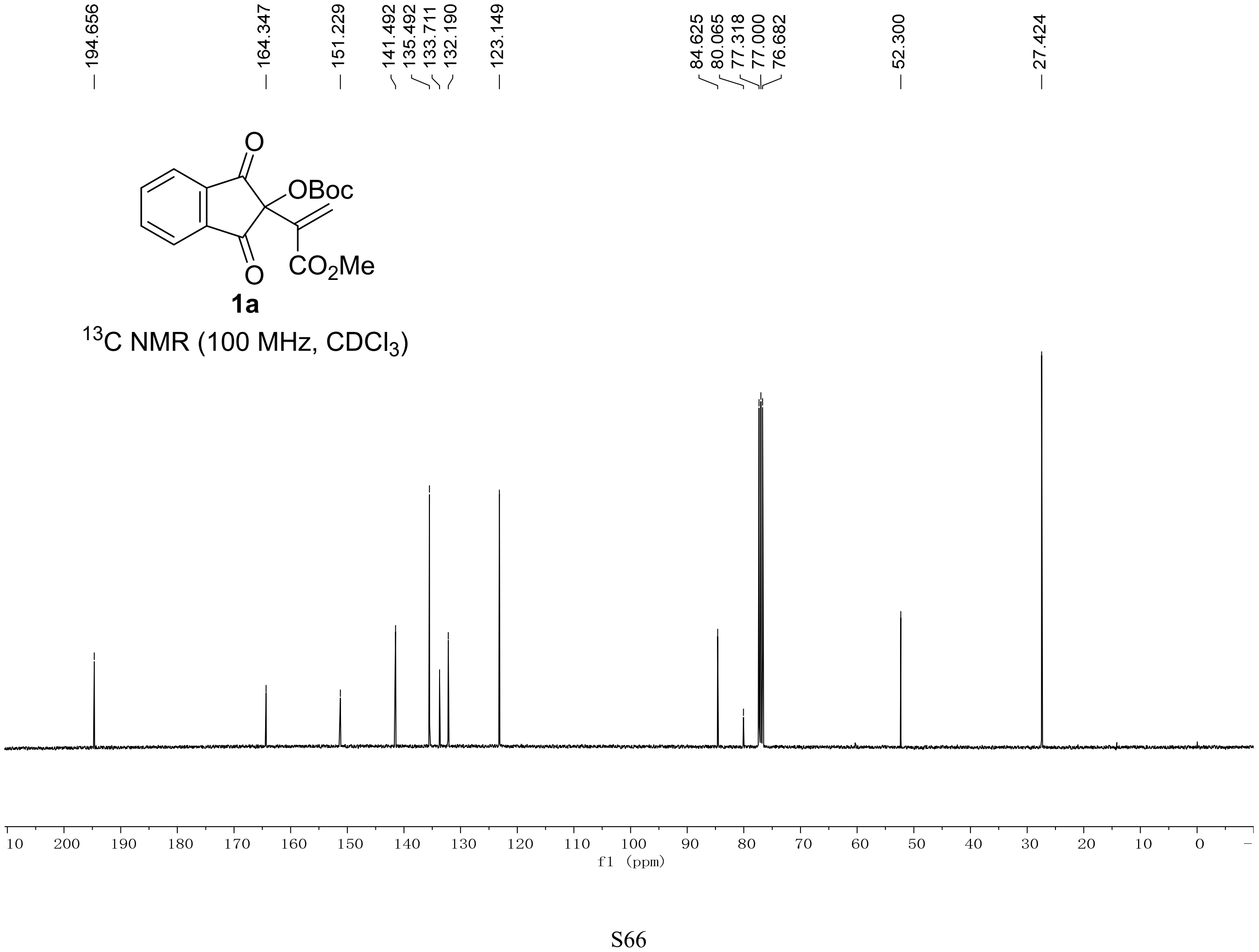

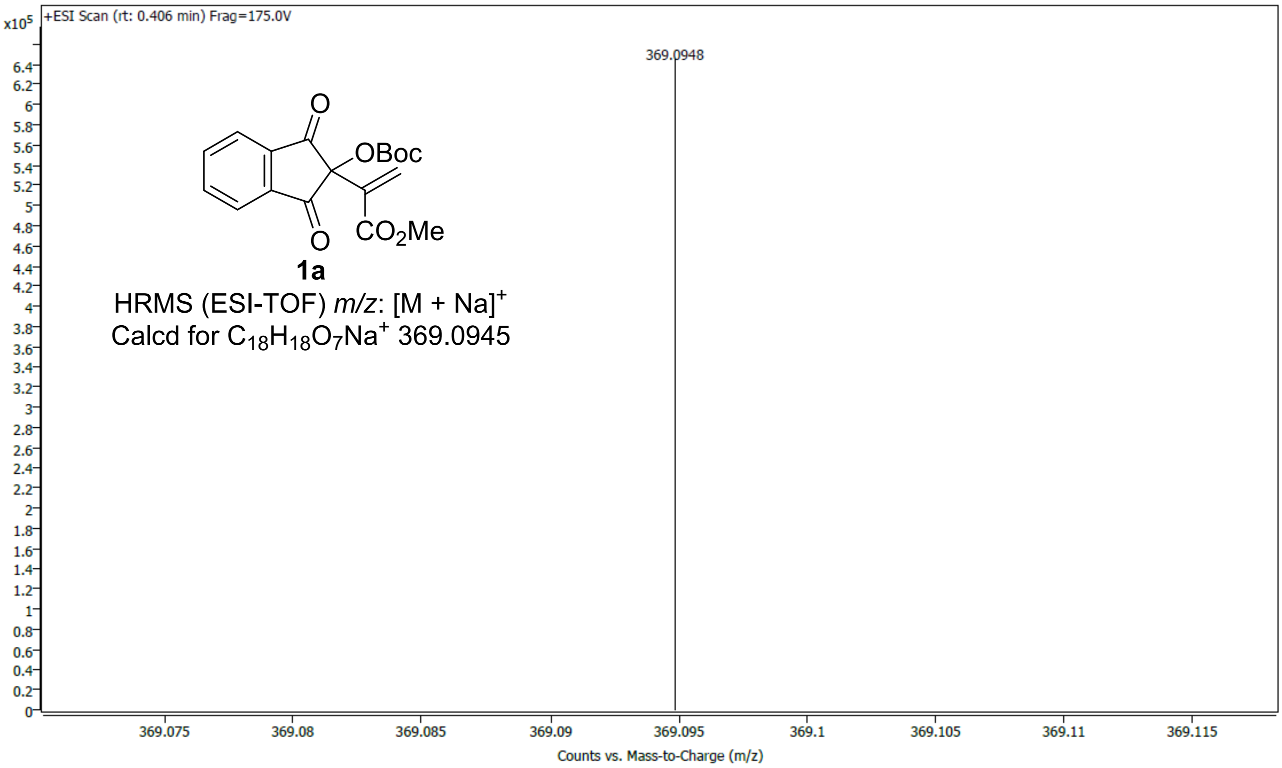

+ESI Scan (rt: 0.406 min) Frag=175.0V
x10^5
369.0948
OBoc
CO2Me
1a
HRMS (ESI-TOF) m/z: [M + Na]+
Calcd for C18H18O7Na+ 369.0945
Counts vs. Mass-to-Charge (m/z)

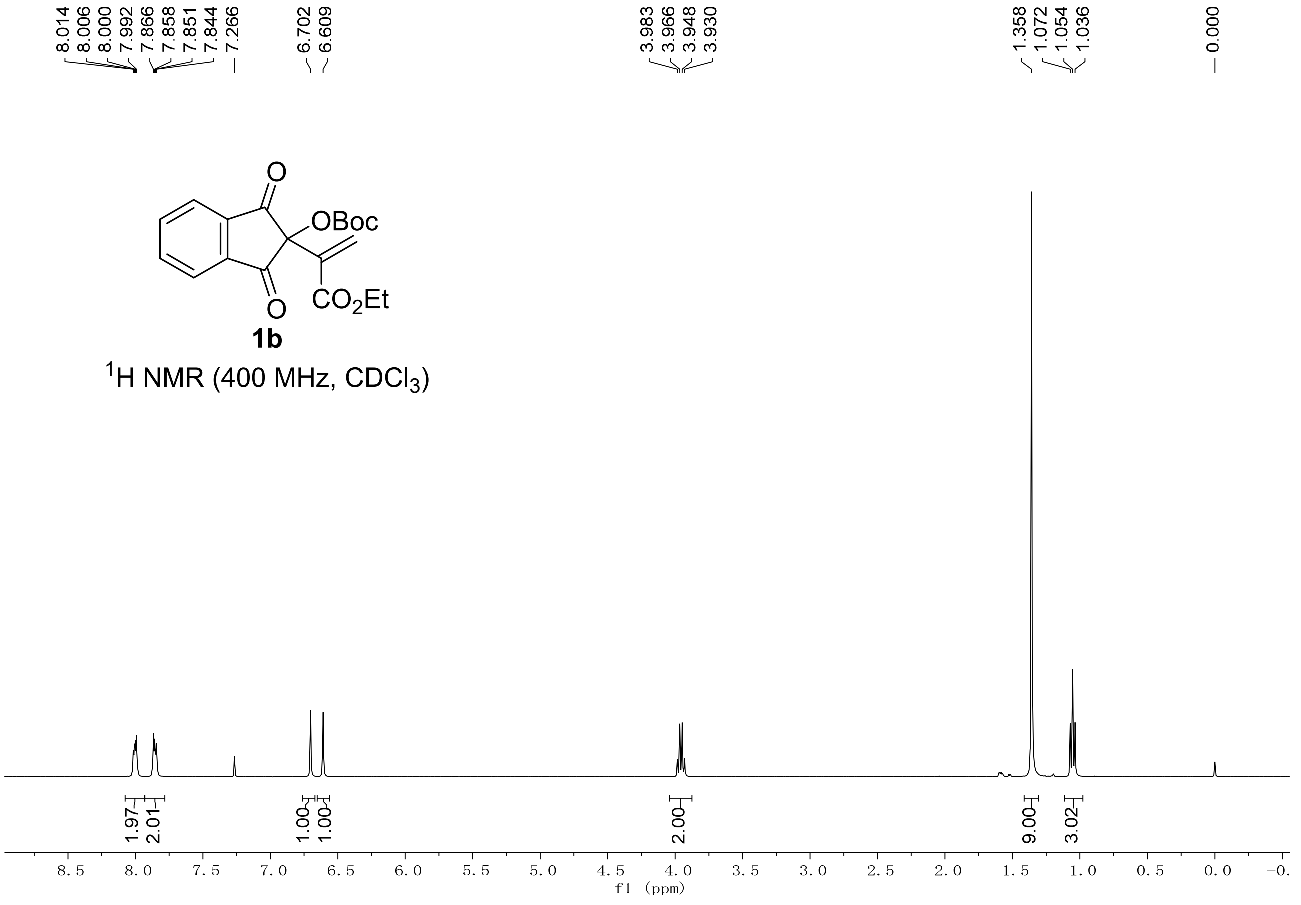
8.014
8.006
8.000
7.992
7.866
7.858
7.851
7.844
7.266
6.702
6.609
3.983
3.966
3.948
3.930
1.358
1.072
1.054
1.036
0.000
O
OBoc
O
CO2Et
1b
1H NMR (400 MHz, CDCl3)
1.97
2.01
1.00
1.00
2.00
9.00
3.02
f1 (ppm)

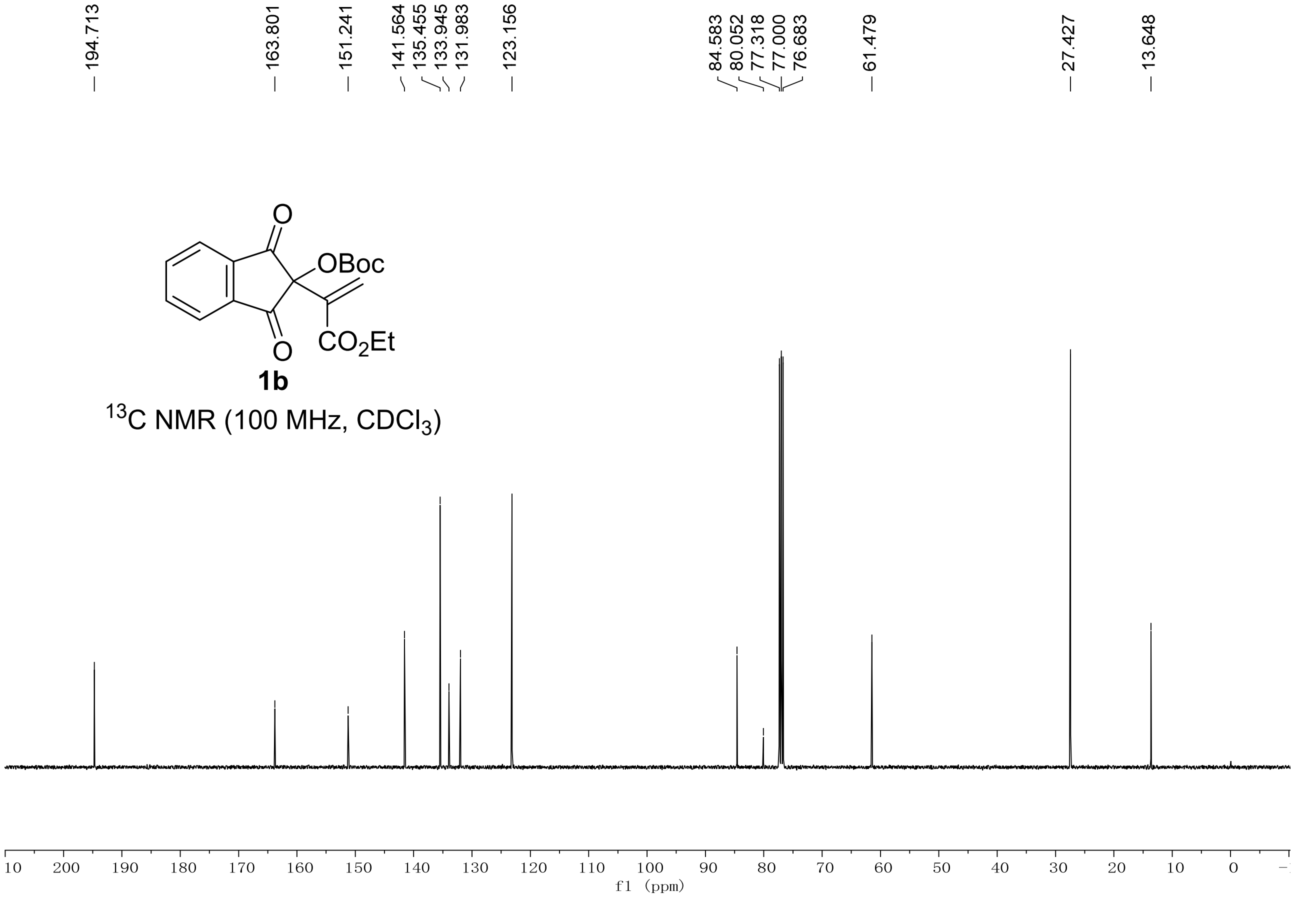
194.713
163.801
151.241
141.564
135.455
133.945
131.983
123.156
84.583
80.052
77.318
77.000
76.683
61.479
27.427
13.648
O
OBoc
O
CO2Et
1b
13C NMR (100 MHz, CDCl3)
f1 (ppm)

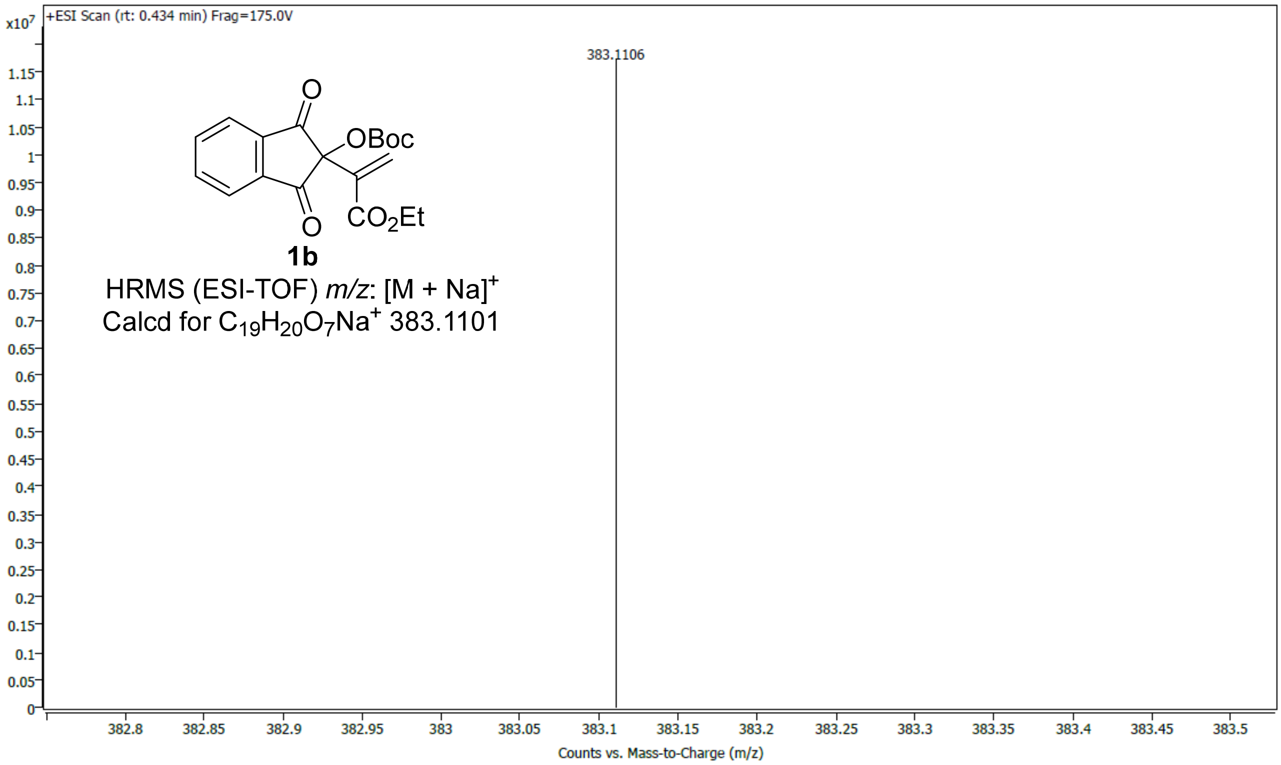
+ESI Scan (rt: 0.434 min) Frag=175.0V
x10^7
383.1106
OBoc
CO2Et
1b
HRMS (ESI-TOF) m/z: [M + Na]+
Calcd for C19H20O7Na+ 383.1101
Counts vs. Mass-to-Charge (m/z)

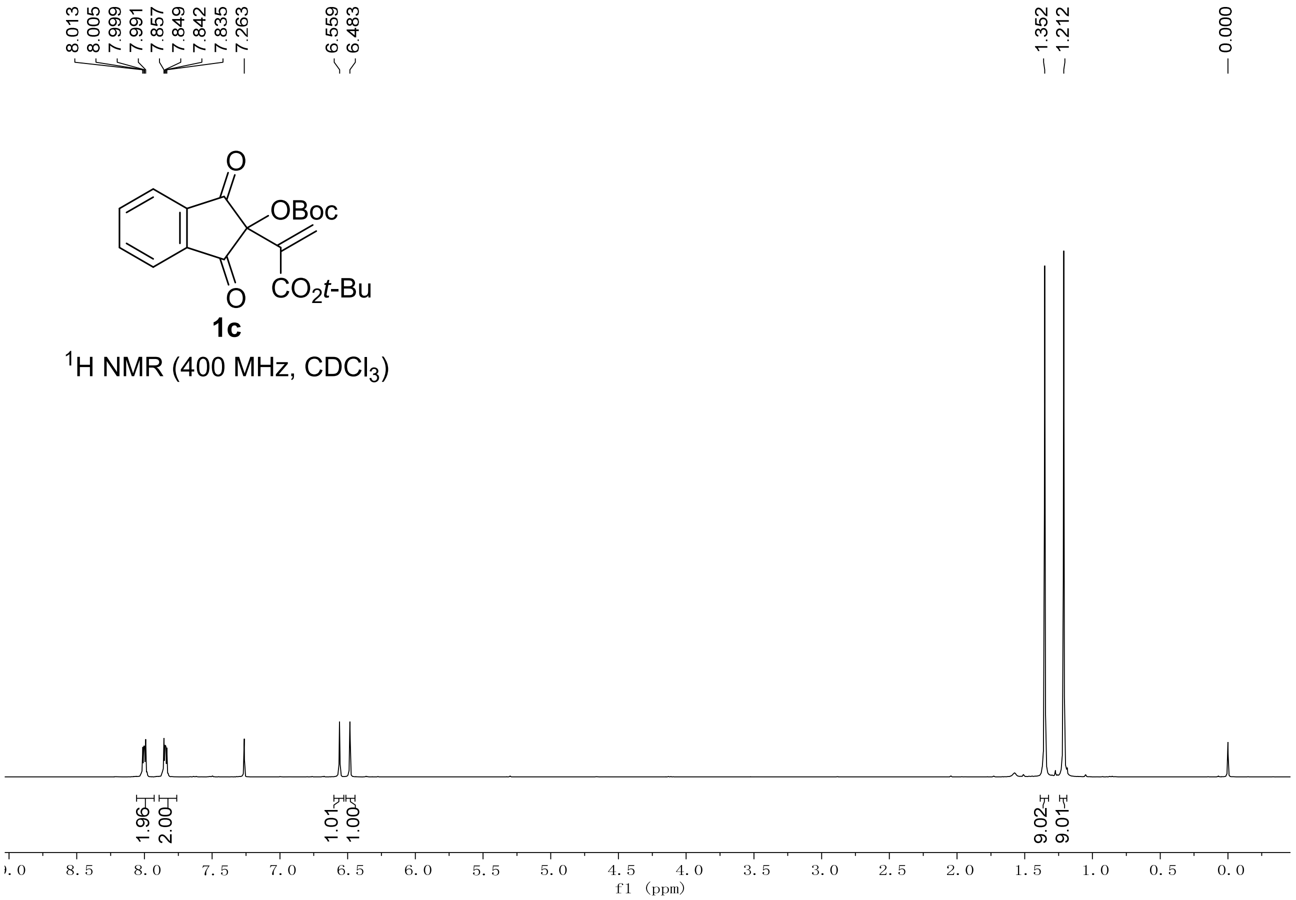
8.013
8.005
7.999
7.991
7.857
7.849
7.842
7.835
7.263
6.559
6.483
1.352
1.212
0.000
OBoc
CO_2t-Bu
1c
^{1}H NMR (400 MHz, $CDCl_3$)
1.96
2.00
1.01
1.00
9.02
9.01
f1 (ppm)

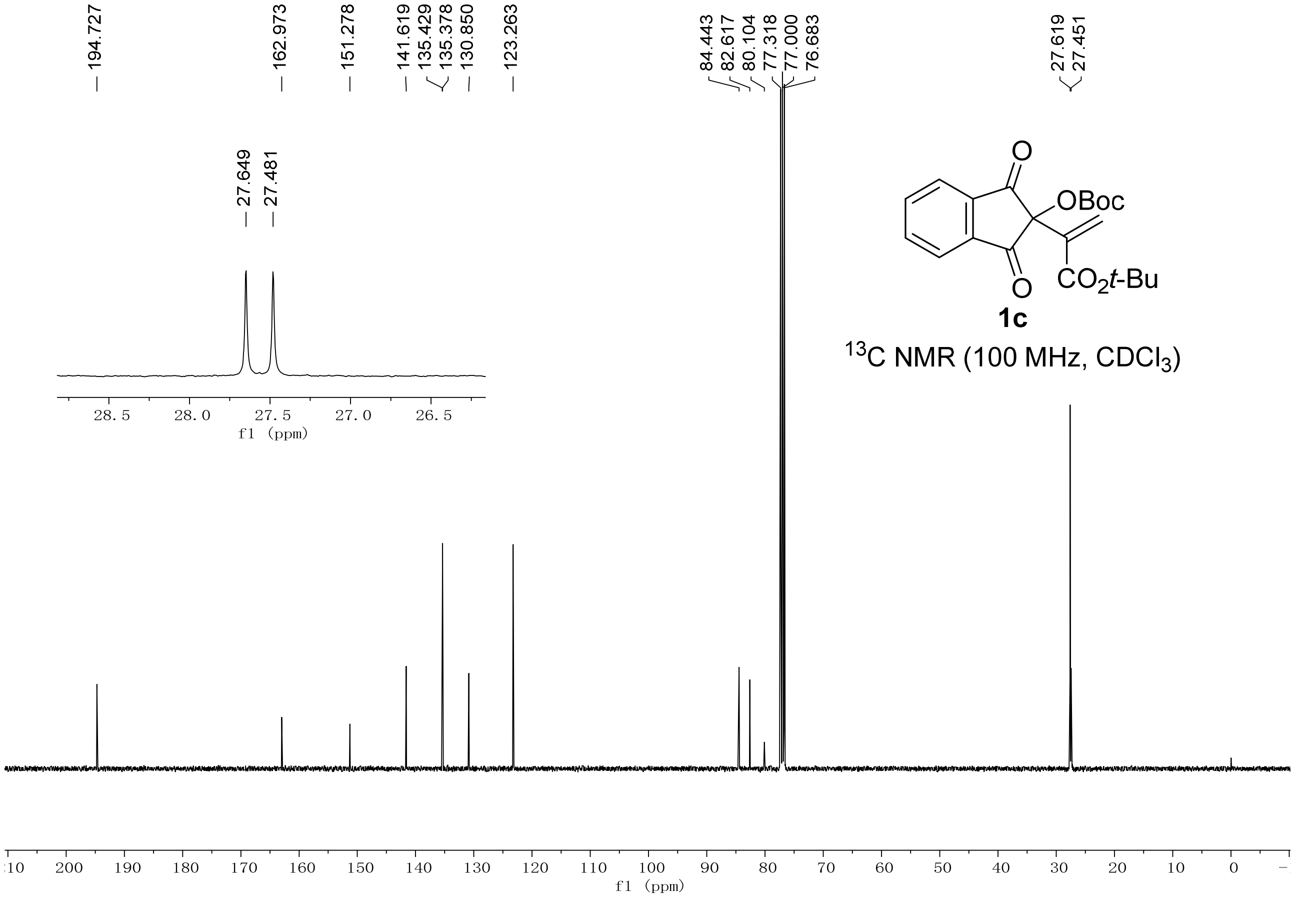
194.727
162.973
151.278
141.619
135.429
135.378
130.850
123.263
84.443
82.617
80.104
77.318
77.000
76.683
27.619
27.451
27.649
27.481
f1 (ppm)
OBoc
CO_2t-Bu
1c
^{13}C NMR (100 MHz, $CDCl_3$)
f1 (ppm)

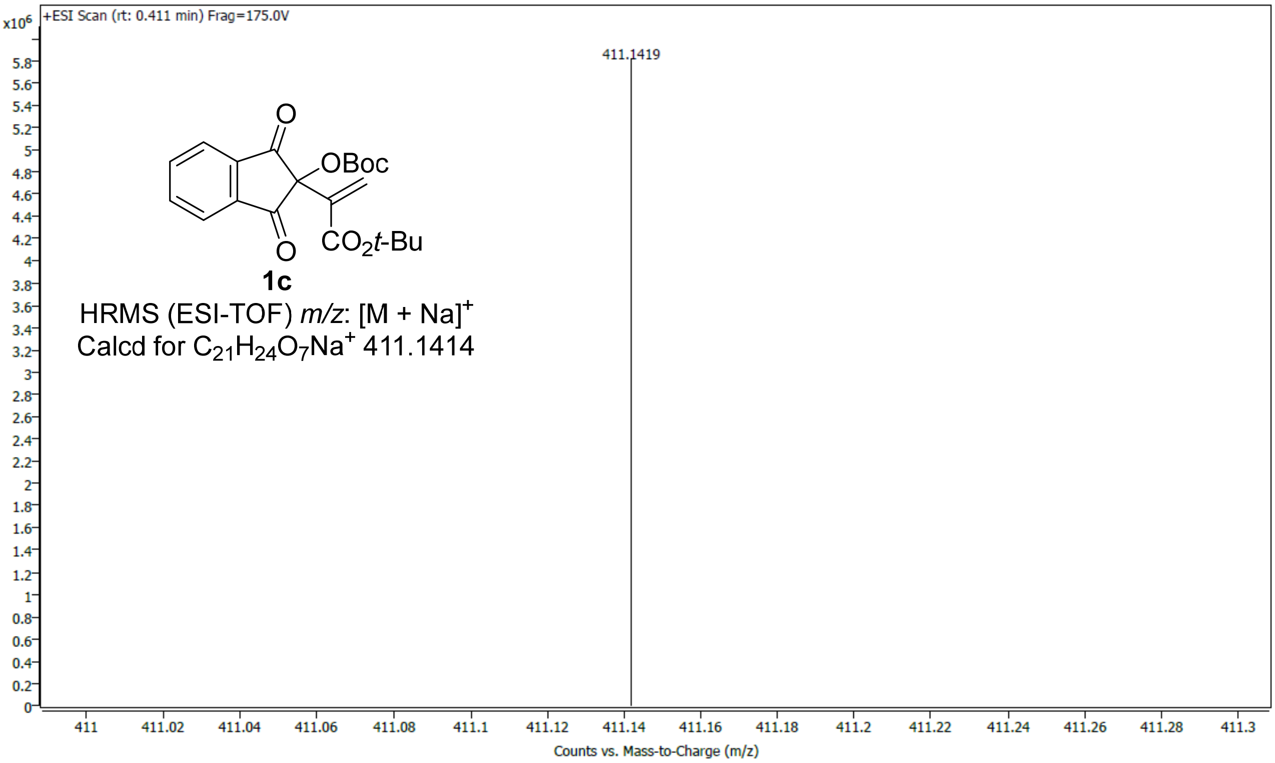

x10^6
+ESI Scan (rt: 0.411 min) Frag=175.0V
411.1419
OBoc
CO2t-Bu
1c
HRMS (ESI-TOF) m/z: [M + Na]+
Calcd for C21H24O7Na+ 411.1414
Counts vs. Mass-to-Charge (m/z)

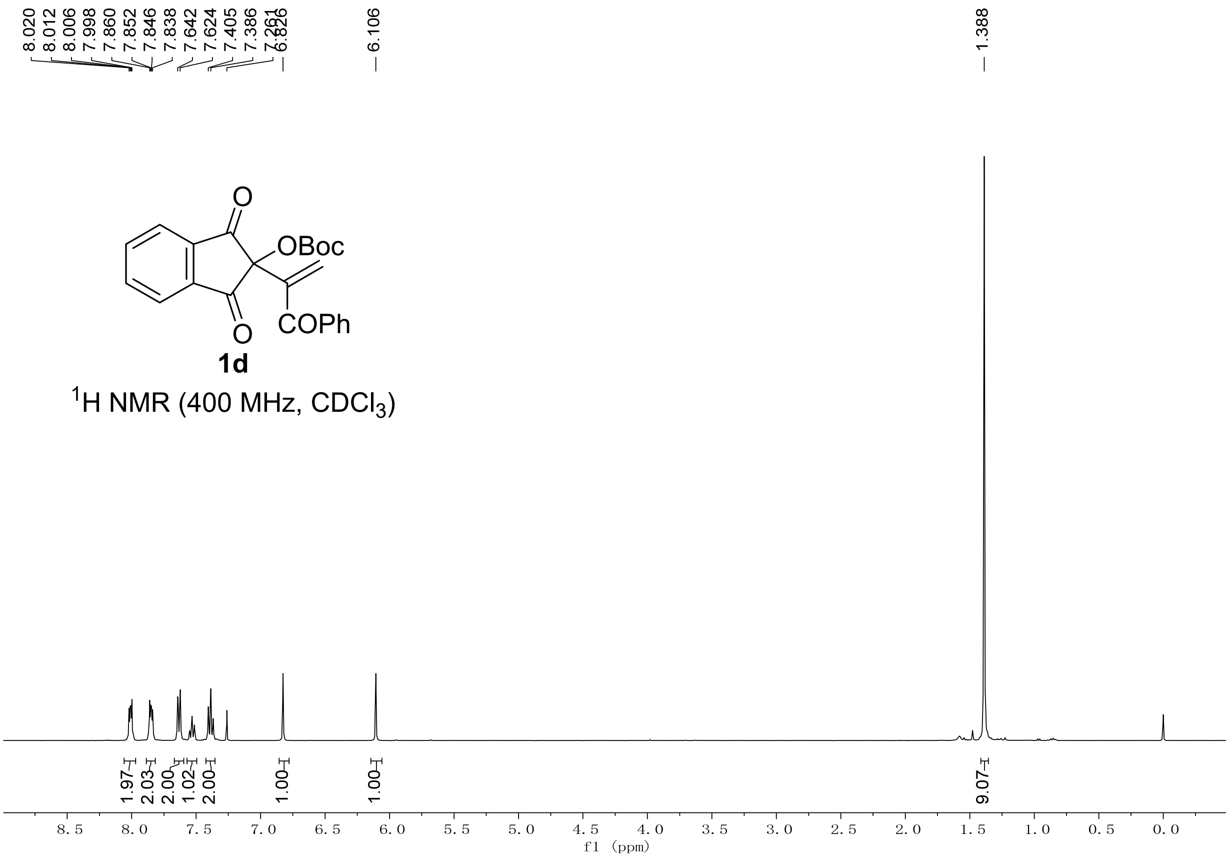
8.020
8.012
8.006
7.998
7.860
7.852
7.846
7.838
7.642
7.624
7.405
7.386
7.261
6.826
6.106
1.388
OBoc
COPh
1d
1H NMR (400 MHz, CDCl3)
1.97
2.03
2.00
1.02
2.00
1.00
1.00
9.07
f1 (ppm)

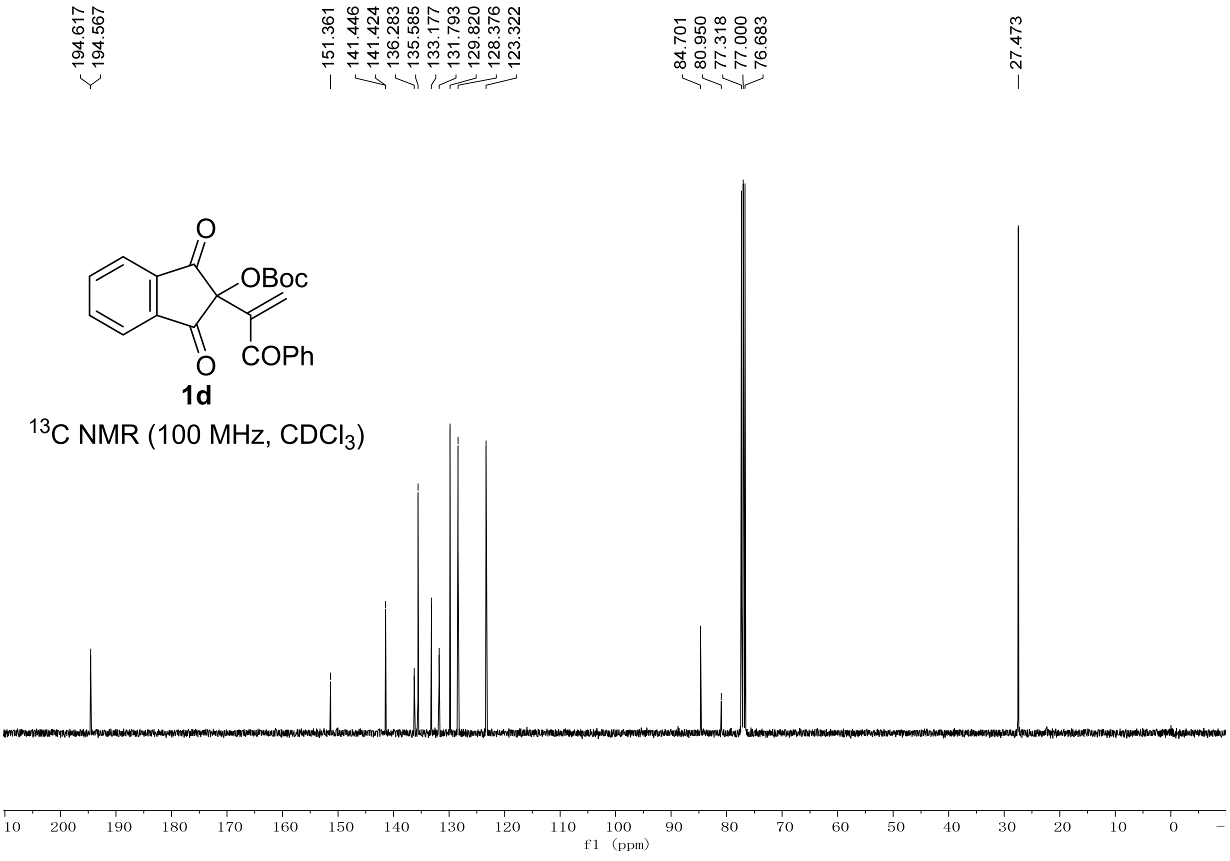
194.617
194.567
151.361
141.446
141.424
136.283
135.585
133.177
131.793
129.820
128.376
123.322
84.701
80.950
77.318
77.000
76.683
27.473
OBoc
COPh
1d
13C NMR (100 MHz, CDCl3)
f1 (ppm)

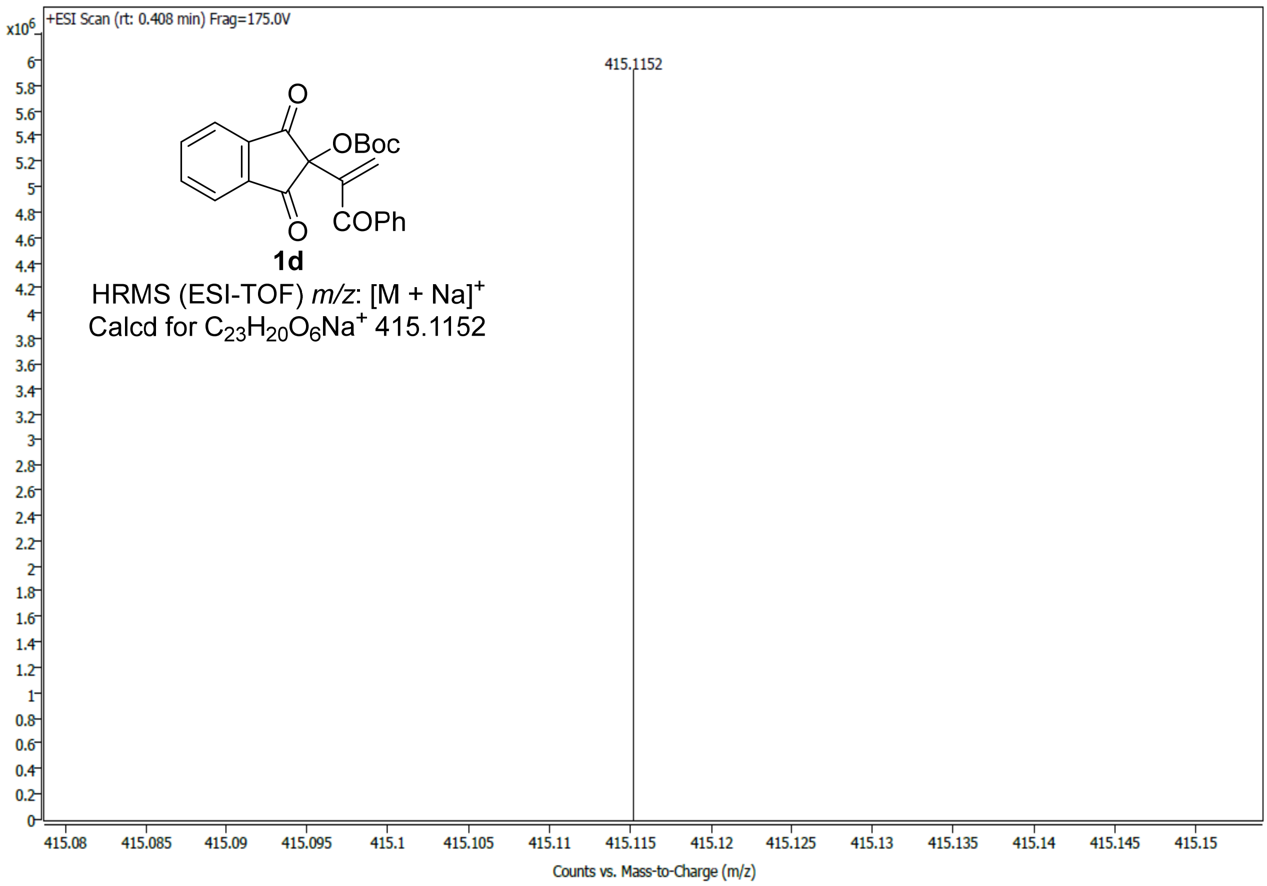
+ESI Scan (rt: 0.408 min) Frag=175.0V
x10^6
415.1152
OBoc
COPh
1d
HRMS (ESI-TOF) m/z: [M + Na]+
Calcd for C23H20O6Na+ 415.1152
Counts vs. Mass-to-Charge (m/z)

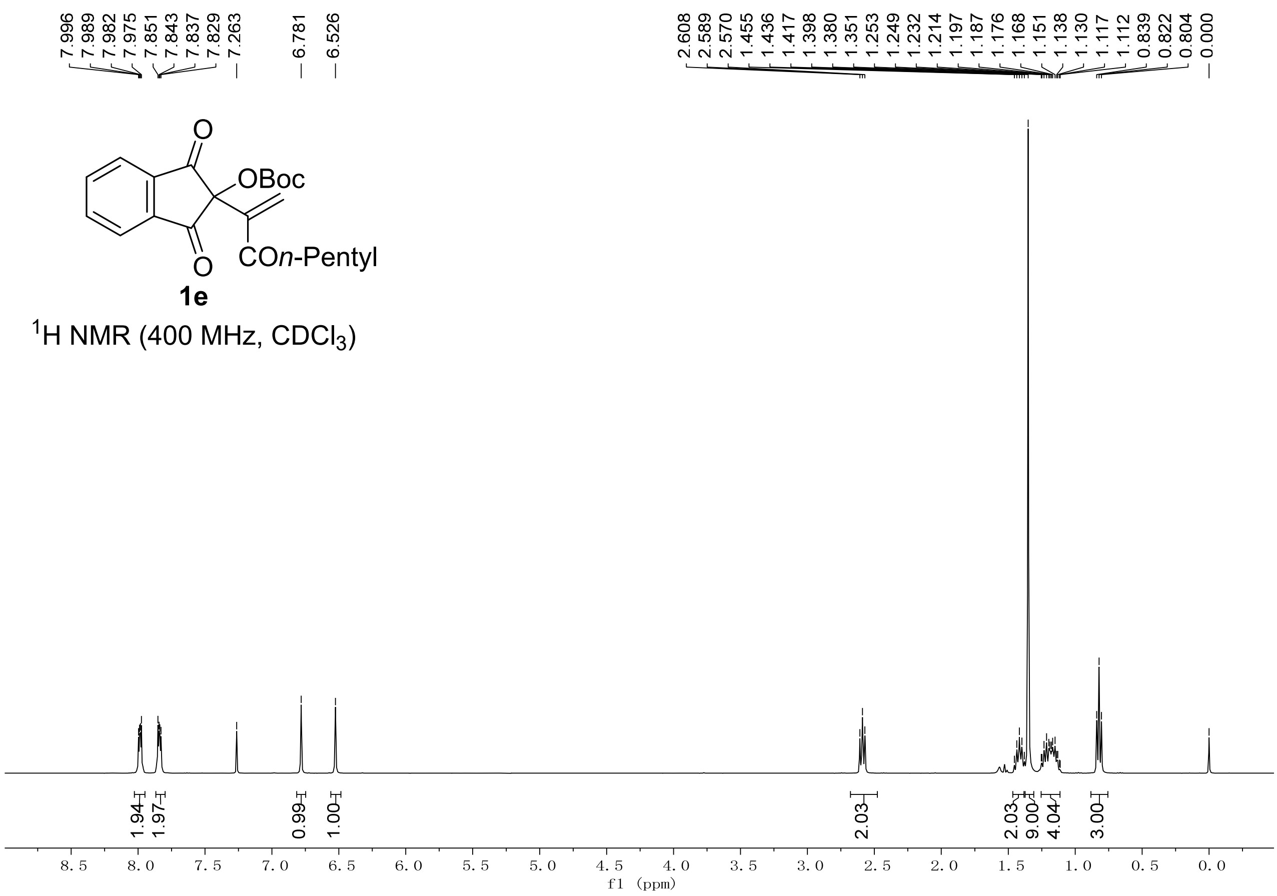
7.996
7.989
7.982
7.975
7.851
7.843
7.837
7.829
7.263
6.781
6.526
2.608
2.589
2.570
1.455
1.436
1.417
1.398
1.380
1.351
1.253
1.249
1.232
1.214
1.197
1.187
1.176
1.168
1.151
1.138
1.130
1.117
1.112
0.839
0.822
0.804
0.000
O
OBoc
O
COn-Pentyl
1e
1H NMR (400 MHz, CDCl3)
1.94
1.97
0.99
1.00
2.03
2.03
9.00
4.04
3.00
8.5 8.0 7.5 7.0 6.5 6.0 5.5 5.0 4.5 4.0 3.5 3.0 2.5 2.0 1.5 1.0 0.5 0.0
f1 (ppm)

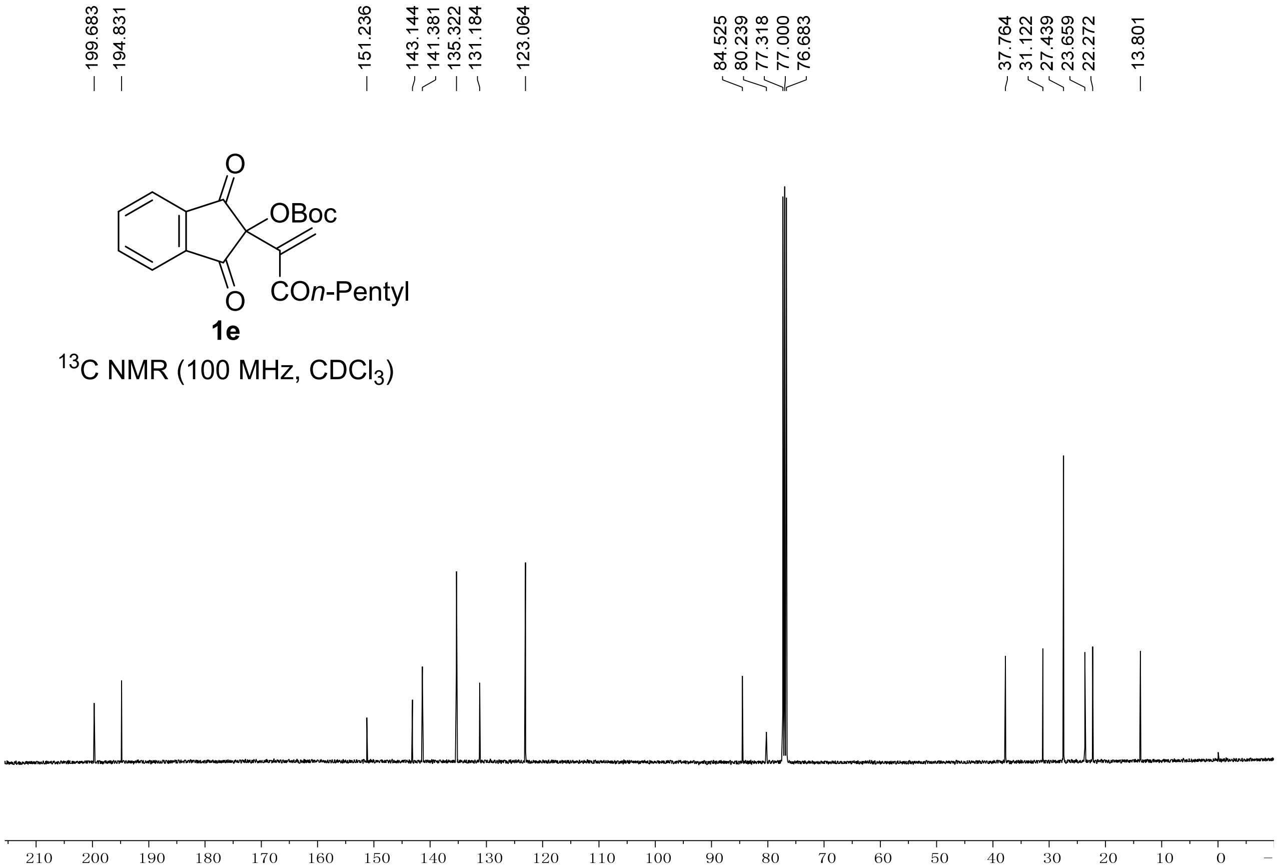
199.683
194.831
151.236
143.144
141.381
135.322
131.184
123.064
84.525
80.239
77.318
77.000
76.683
37.764
31.122
27.439
23.659
22.272
13.801
O
OBoc
O
COn-Pentyl
1e
13C NMR (100 MHz, CDCl3)
210 200 190 180 170 160 150 140 130 120 110 100 90 80 70 60 50 40 30 20 10 0
f1 (ppm)

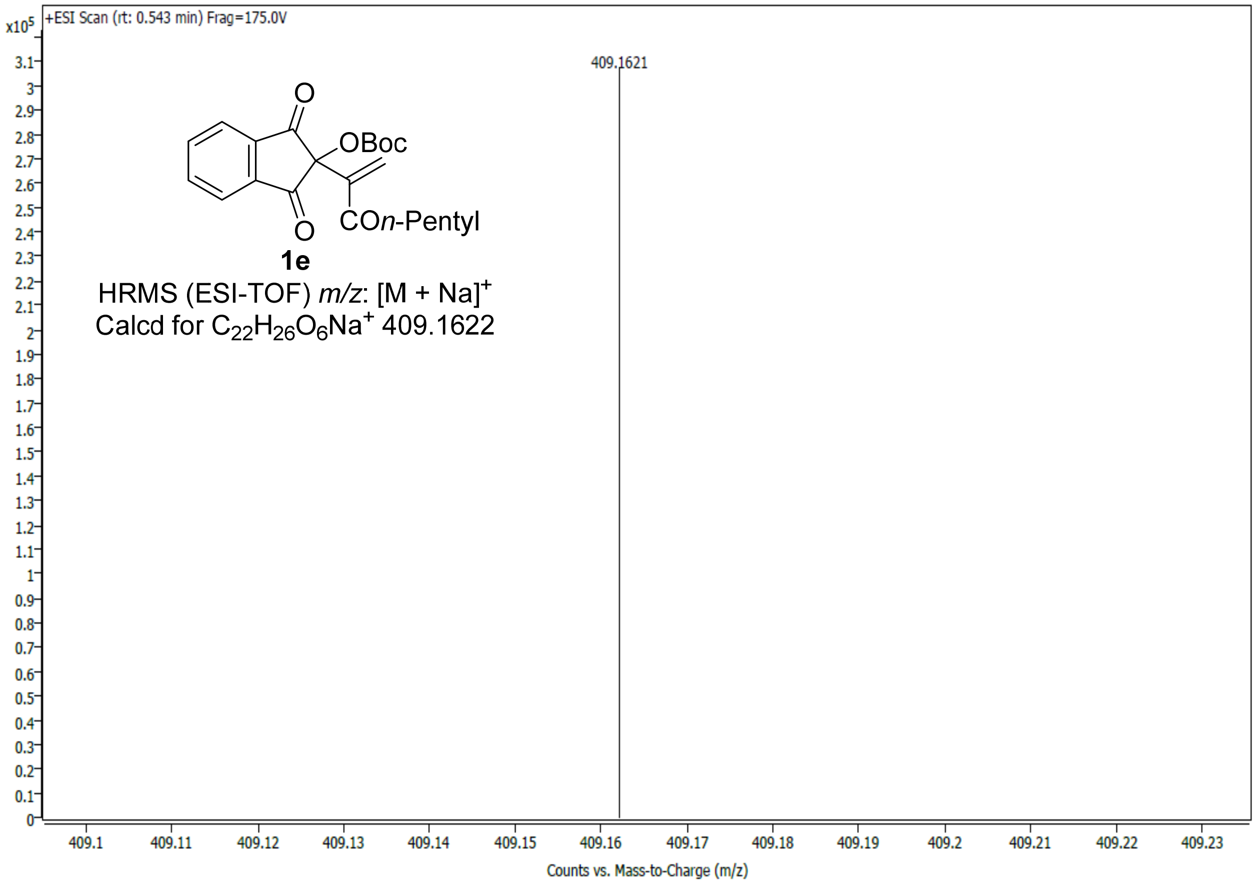

+ESI Scan (rt: 0.543 min) Frag=175.0V
x10^5
409.1621
OBoc
COn-Pentyl
1e
HRMS (ESI-TOF) m/z: [M + Na]+
Calcd for C22H26O6Na+ 409.1622
Counts vs. Mass-to-Charge (m/z)

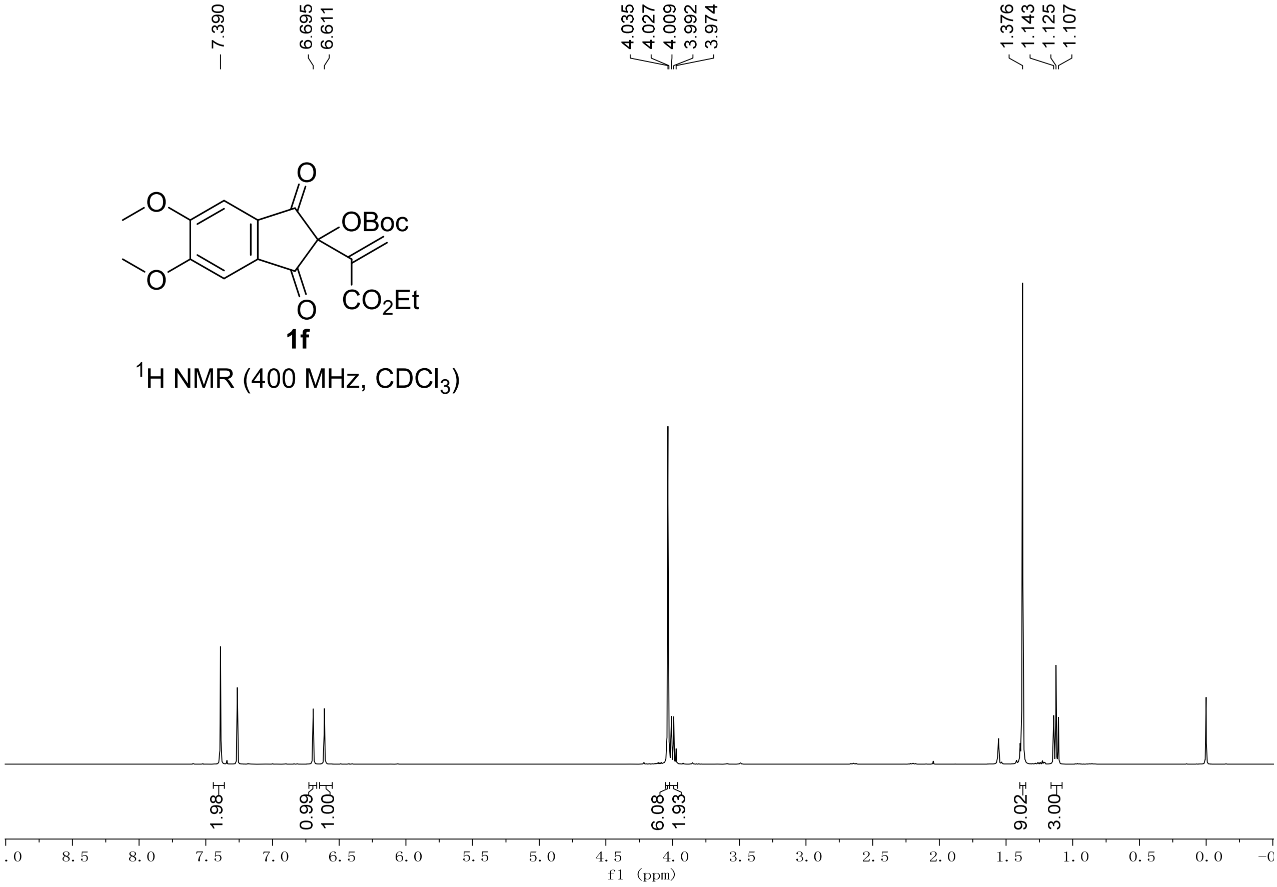
7.390
6.695
6.611
4.035
4.027
4.009
3.992
3.974
1.376
1.143
1.125
1.107
OBoc
CO2Et
1f
1H NMR (400 MHz, CDCl3)
1.98
0.99
1.00
6.08
1.93
9.02
3.00
f1 (ppm)

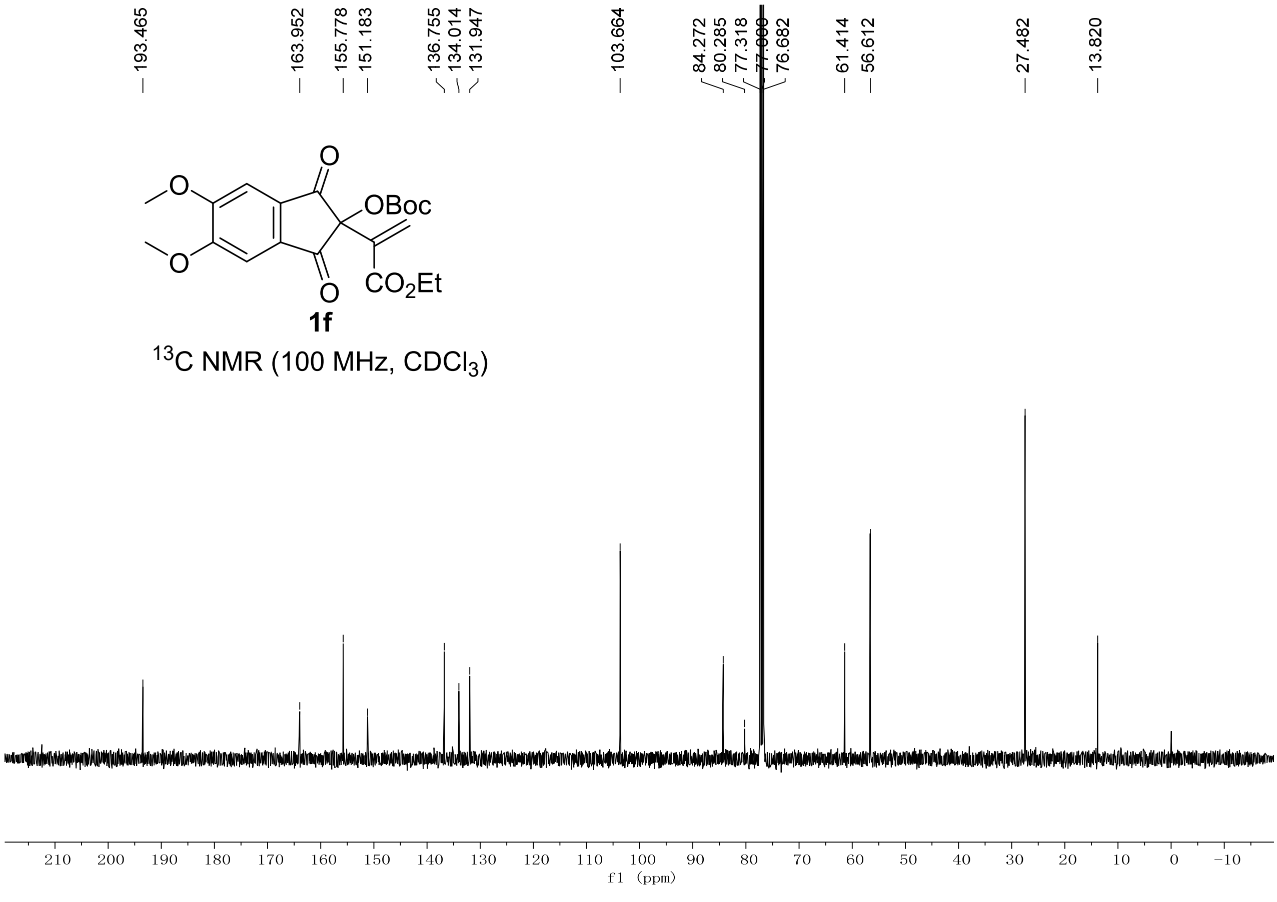
193.465
163.952
155.778
151.183
136.755
134.014
131.947
103.664
84.272
80.285
77.318
76.682
61.414
56.612
27.482
13.820
OBoc
CO2Et
1f
13C NMR (100 MHz, CDCl3)
f1 (ppm)

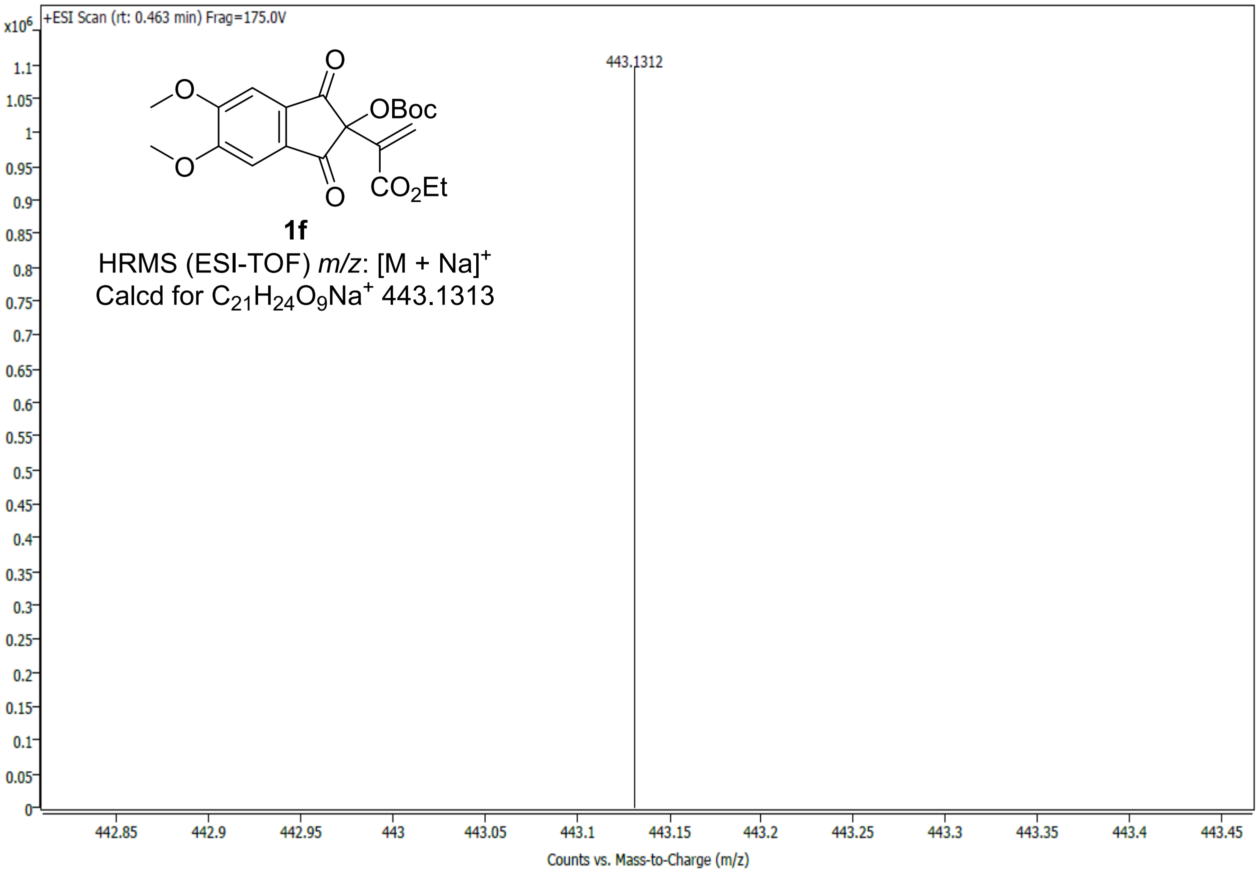
+ESI Scan (rt: 0.463 min) Frag=175.0V
x10^6
443.1312
OBoc
CO2Et
1f
HRMS (ESI-TOF) m/z: [M + Na]+
Calcd for C21H24O9Na+ 443.1313
Counts vs. Mass-to-Charge (m/z)

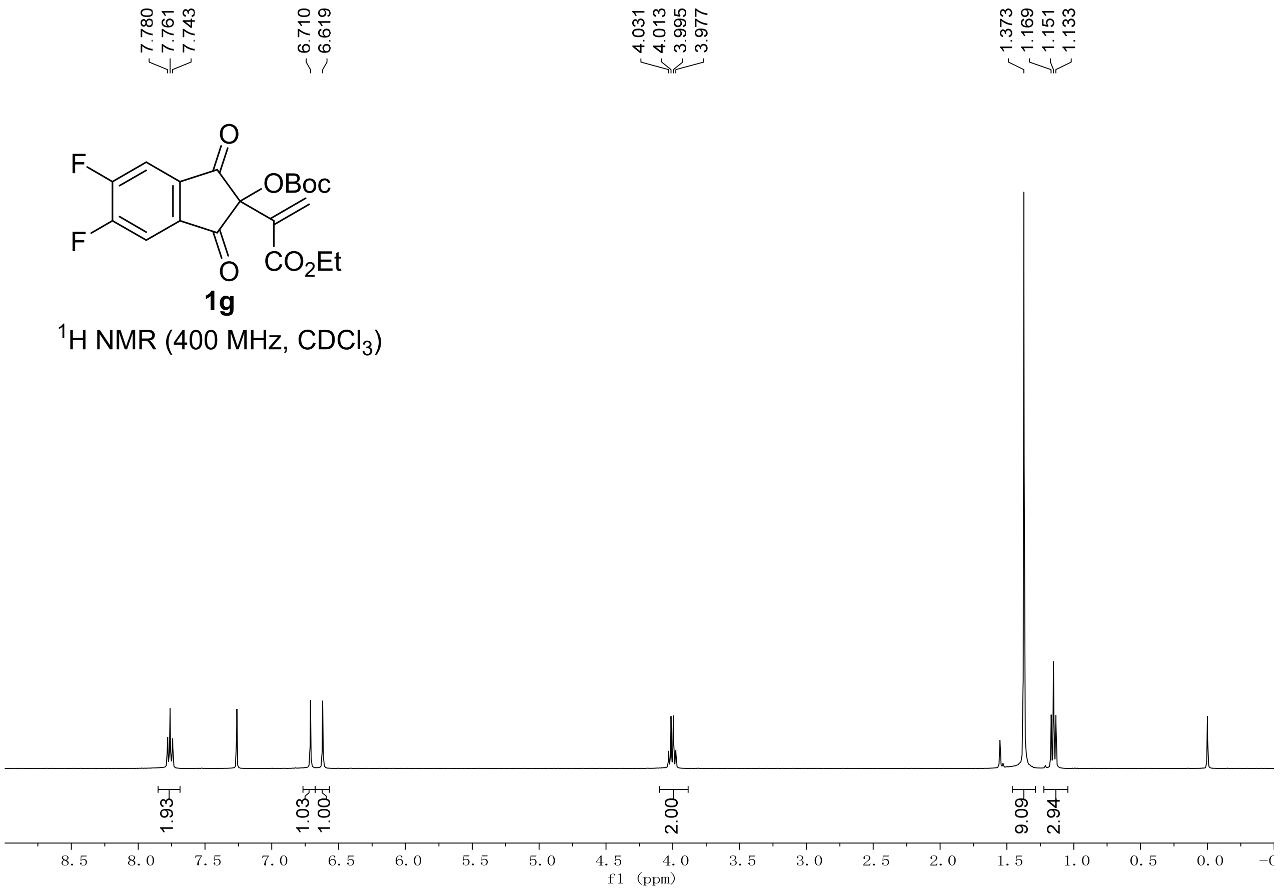
7.780
7.761
7.743
6.710
6.619
4.031
4.013
3.995
3.977
1.373
1.169
1.151
1.133
F
F
O
O
OBoc
CO_2Et
1g
^{1}H NMR (400 MHz, $CDCl_3$)
1.93
1.03
1.00
2.00
9.09
2.94
f1 (ppm)

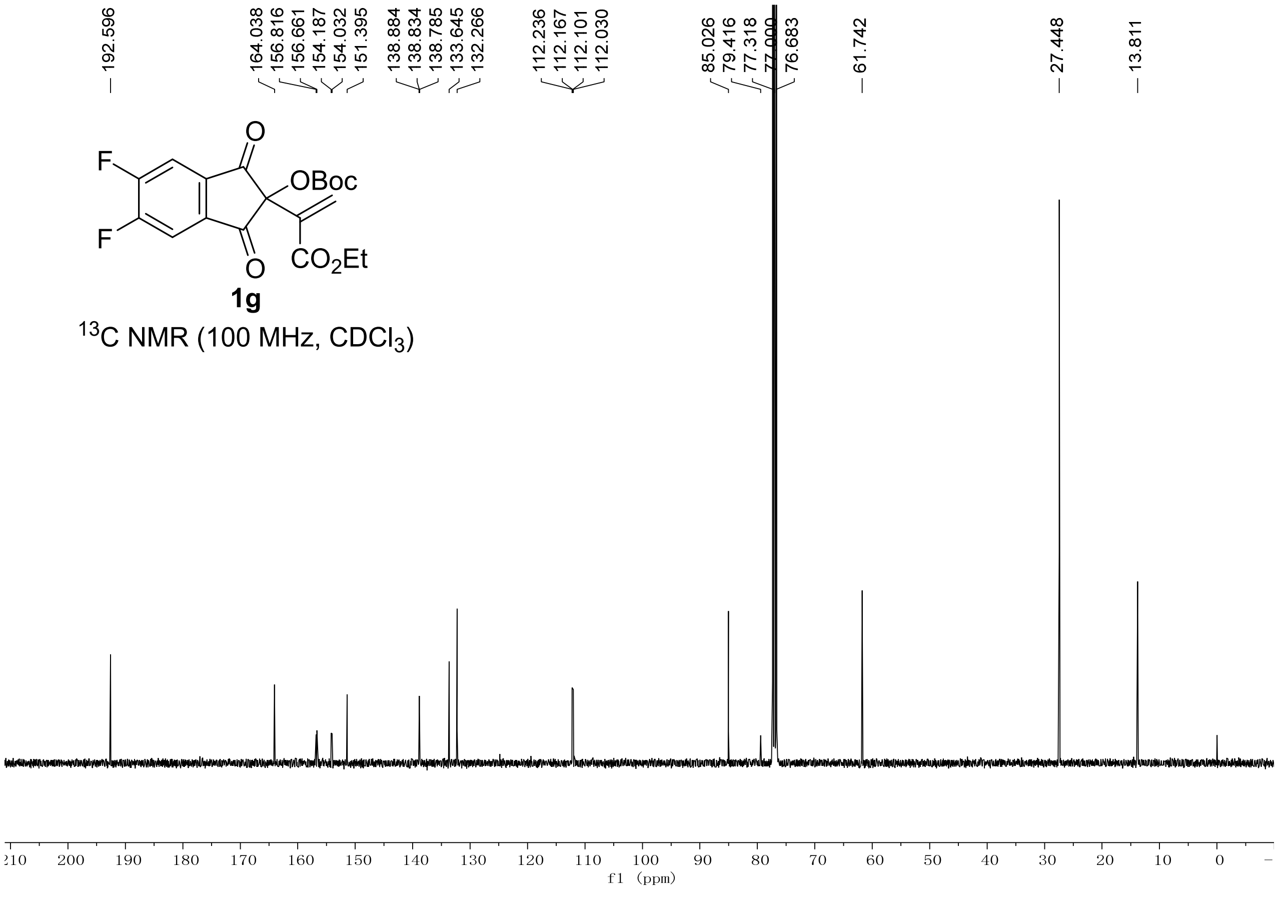
192.596
164.038
156.816
156.661
154.187
154.032
151.395
138.884
138.834
138.785
133.645
132.266
112.236
112.167
112.101
112.030
85.026
79.416
77.318
77.000
76.683
61.742
27.448
13.811
F
F
O
O
OBoc
CO_2Et
1g
^{13}C NMR (100 MHz, $CDCl_3$)
f1 (ppm)

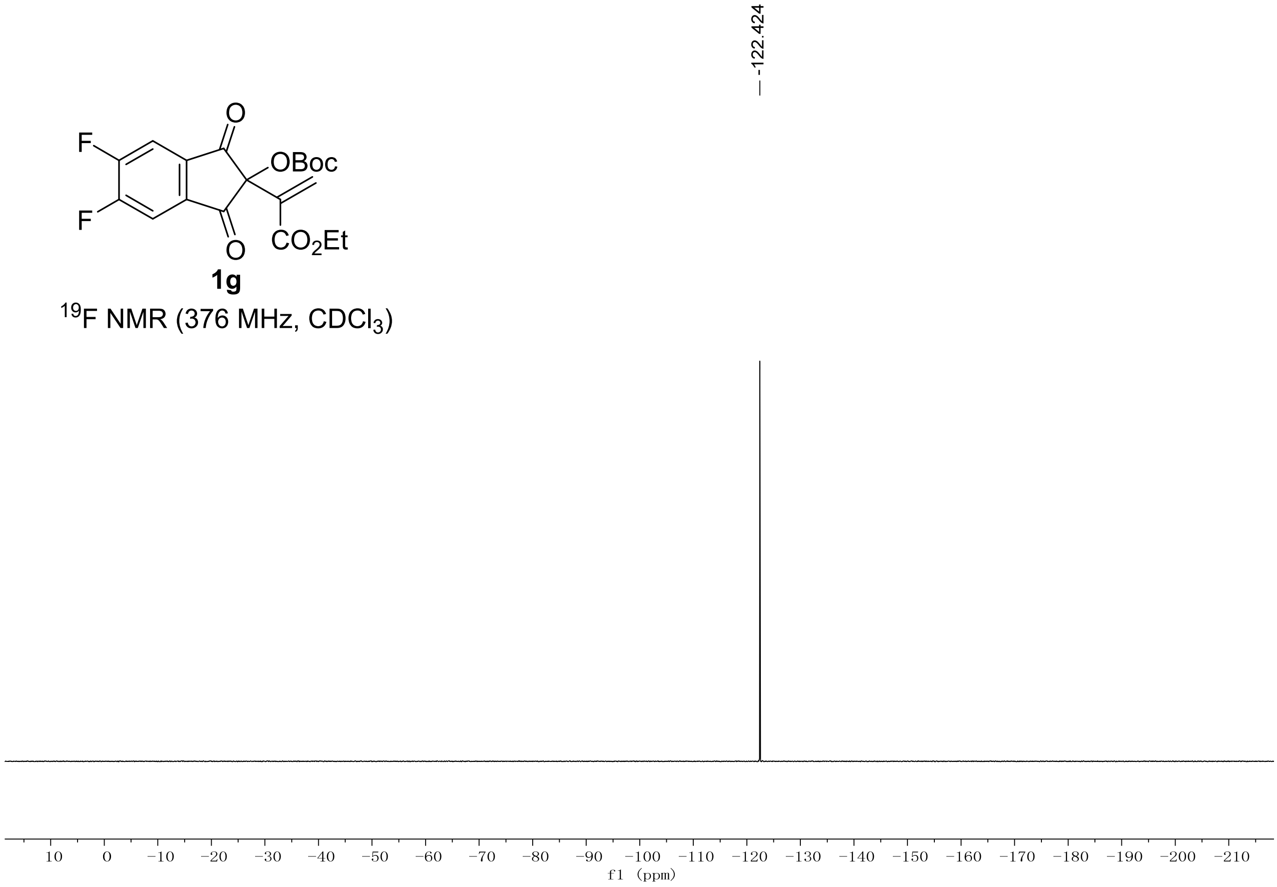
-122.424
F
F
O
O
OBoc
CO2Et
1g
19F NMR (376 MHz, CDCl3)
10 0 −10 −20 −30 −40 −50 −60 −70 −80 −90 −100 −110 −120 −130 −140 −150 −160 −170 −180 −190 −200 −210
f1 (ppm)

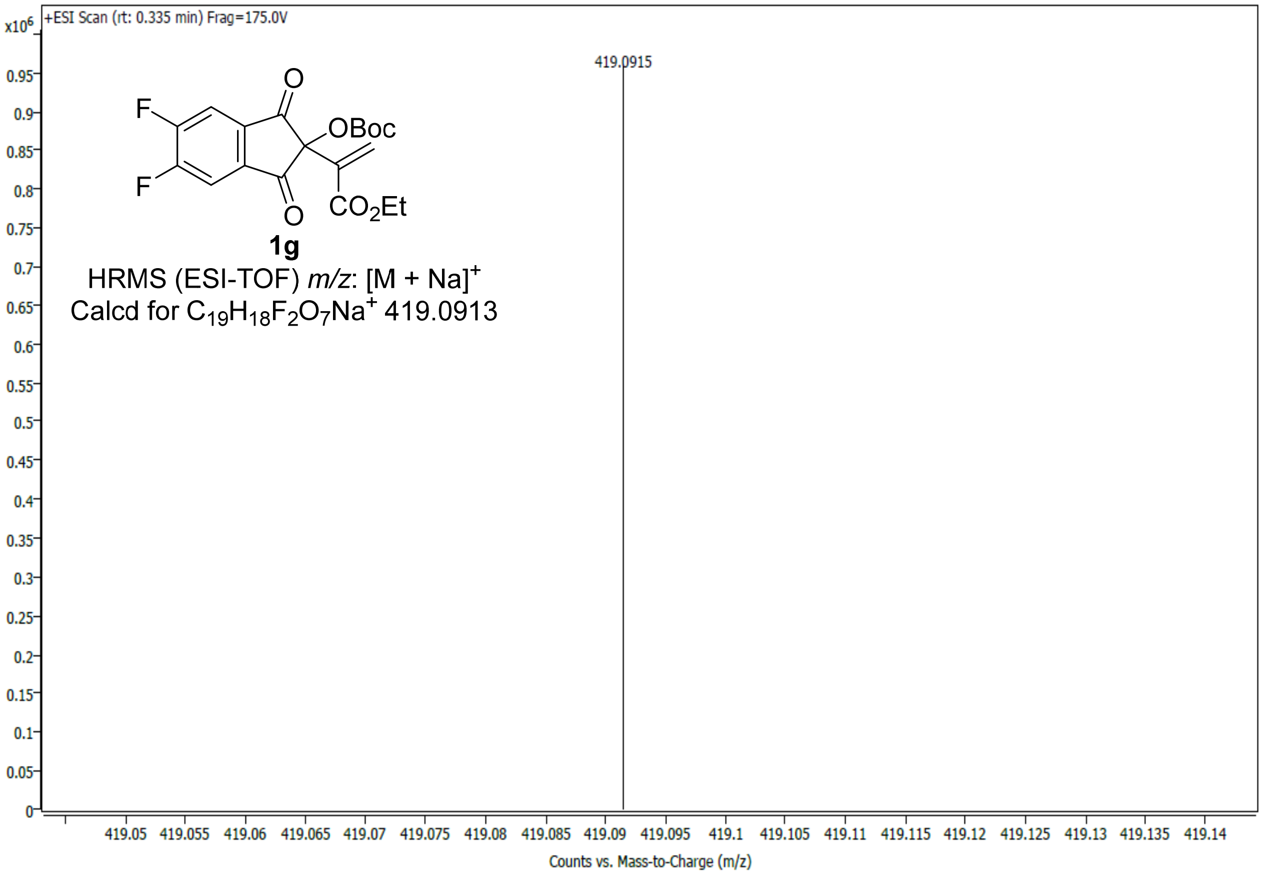
+ESI Scan (rt: 0.335 min) Frag=175.0V
x10^6
419.0915
F
F
O
O
OBoc
CO2Et
1g
HRMS (ESI-TOF) m/z: [M + Na]+
Calcd for C19H18F2O7Na+ 419.0913
0.95 0.9 0.85 0.8 0.75 0.7 0.65 0.6 0.55 0.5 0.45 0.4 0.35 0.3 0.25 0.2 0.15 0.1 0.05 0
419.05 419.055 419.06 419.065 419.07 419.075 419.08 419.085 419.09 419.095 419.1 419.105 419.11 419.115 419.12 419.125 419.13 419.135 419.14
Counts vs. Mass-to-Charge (m/z)

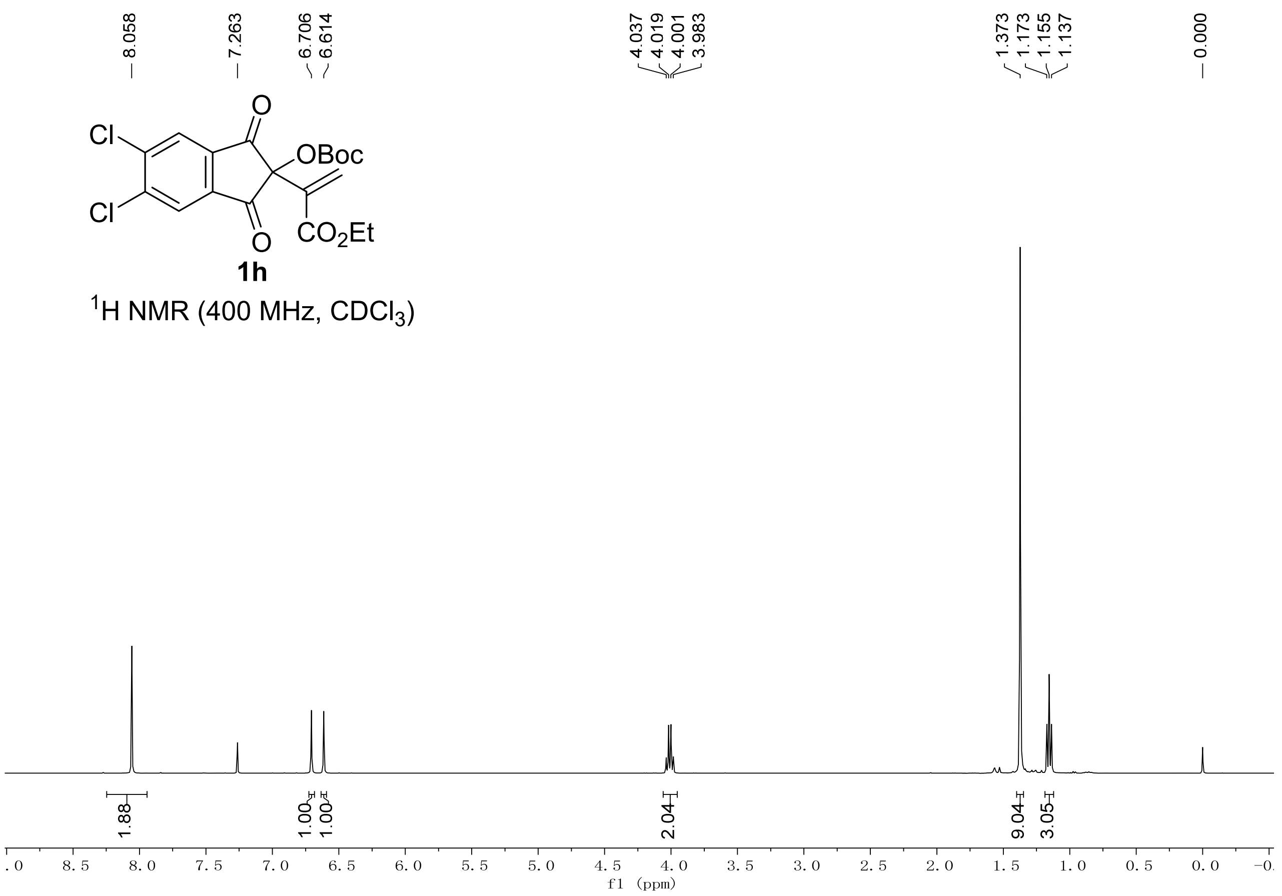

8.058
7.263
6.706
6.614
4.037
4.019
4.001
3.983
1.373
1.173
1.155
1.137
0.000
Cl
Cl
O
O
OBoc
CO2Et
1h
1H NMR (400 MHz, CDCl3)
1.88
1.00
1.00
2.04
9.04
3.05
f1 (ppm)

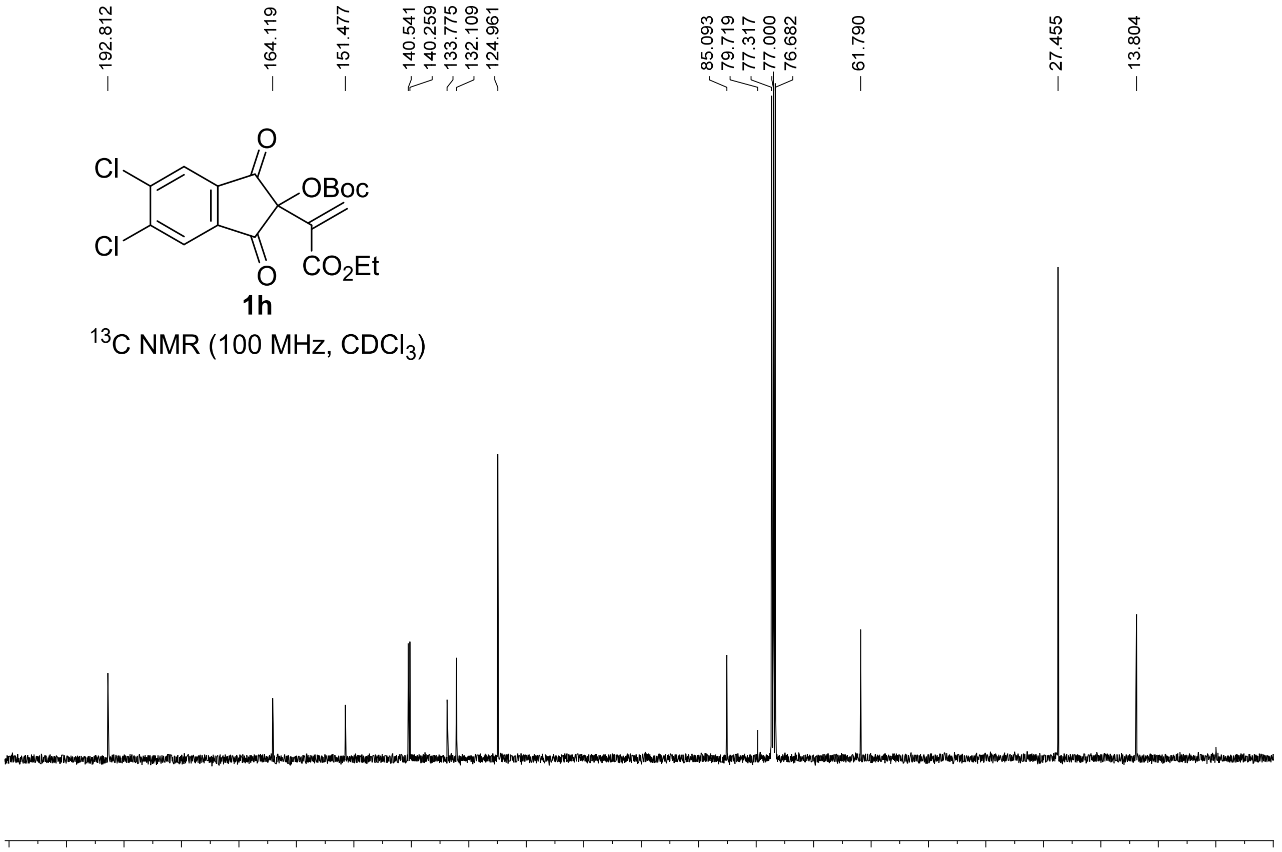

192.812
164.119
151.477
140.541
140.259
133.775
132.109
124.961
85.093
79.719
77.317
77.000
76.682
61.790
27.455
13.804
Cl
Cl
O
O
OBoc
CO2Et
1h
13C NMR (100 MHz, CDCl3)
f1 (ppm)

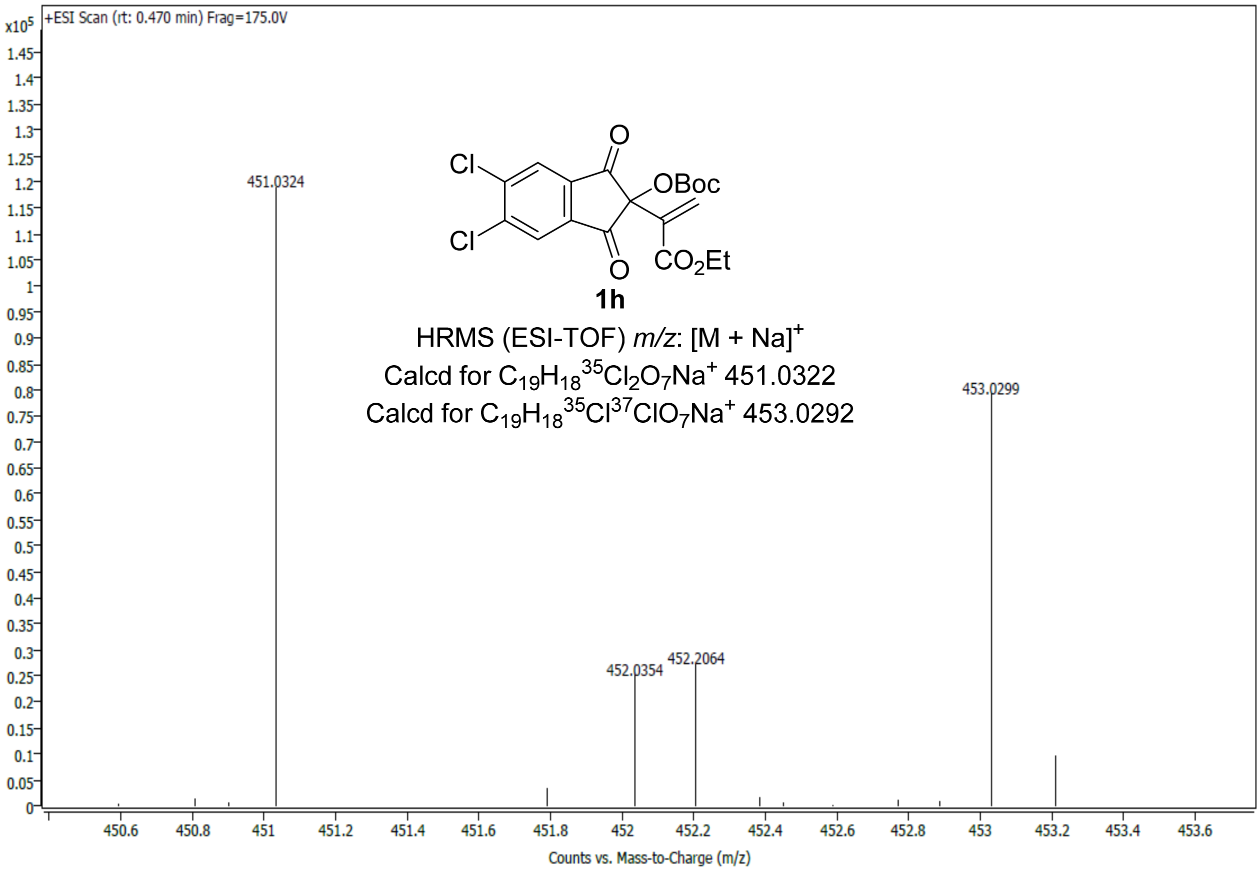
+ESI Scan (rt: 0.470 min) Frag=175.0V
x10^5
451.0324
452.0354
452.2064
453.0299
Cl
Cl
O
O
OBoc
CO2Et
1h
HRMS (ESI-TOF) m/z: [M + Na]+
Calcd for C19H18 35Cl2O7Na+ 451.0322
Calcd for C19H18 35Cl 37ClO7Na+ 453.0292
Counts vs. Mass-to-Charge (m/z)

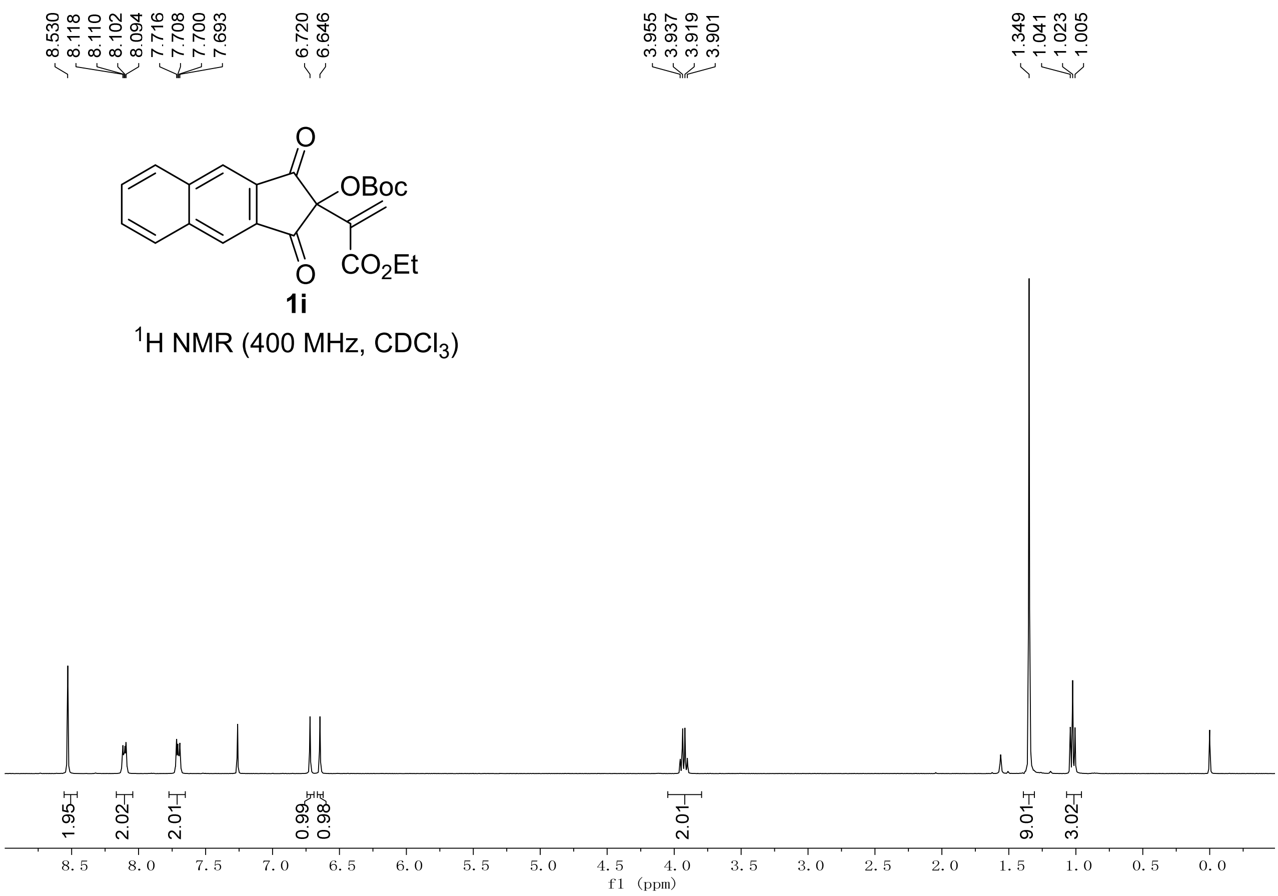
8.530
8.118
8.110
8.102
8.094
7.716
7.708
7.700
7.693
6.720
6.646
3.955
3.937
3.919
3.901
1.349
1.041
1.023
1.005
O
OBoc
O
CO_2Et
1i
^1H NMR (400 MHz, $CDCl_3$)
1.95
2.02
2.01
0.99
0.98
2.01
9.01
3.02
f1 (ppm)

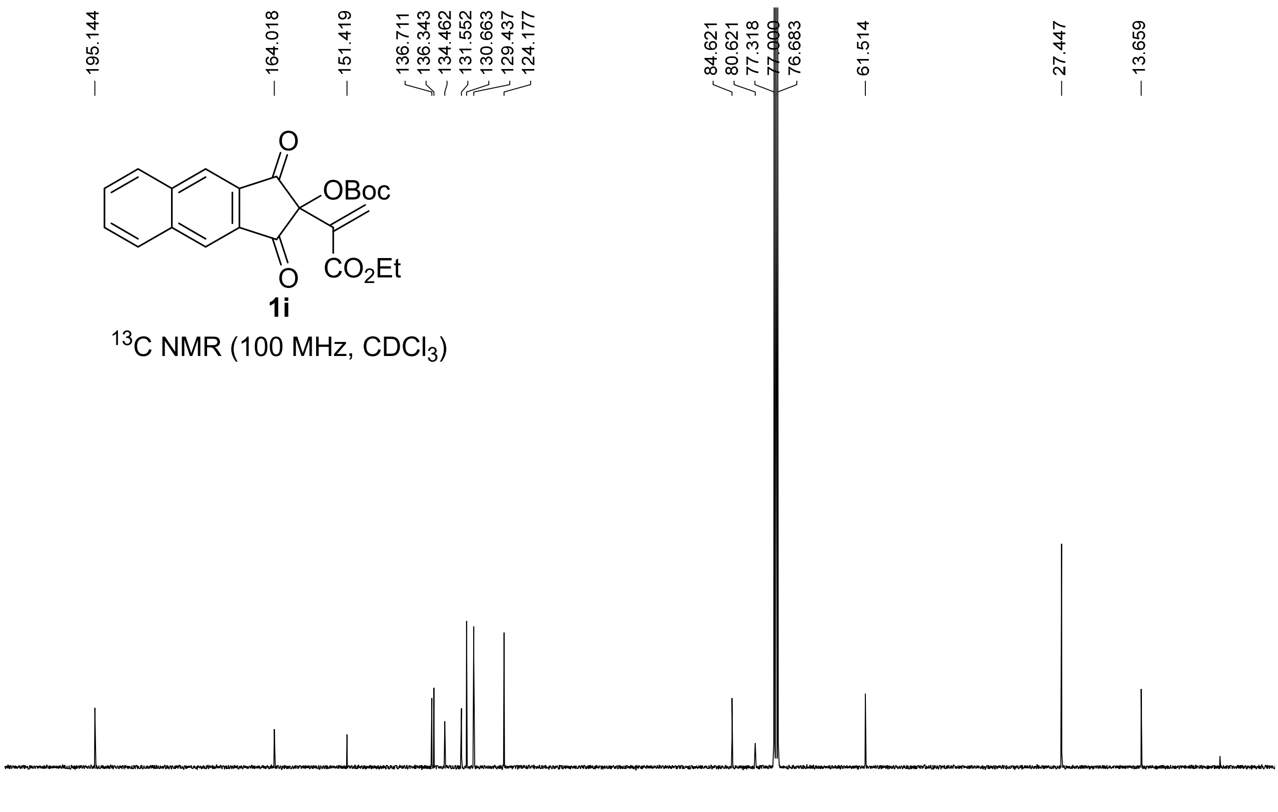
195.144
164.018
151.419
136.711
136.343
134.462
131.552
130.663
129.437
124.177
84.621
80.621
77.318
77.000
76.683
61.514
27.447
13.659
O
OBoc
O
CO_2Et
1i
^{13}C NMR (100 MHz, $CDCl_3$)
f1 (ppm)

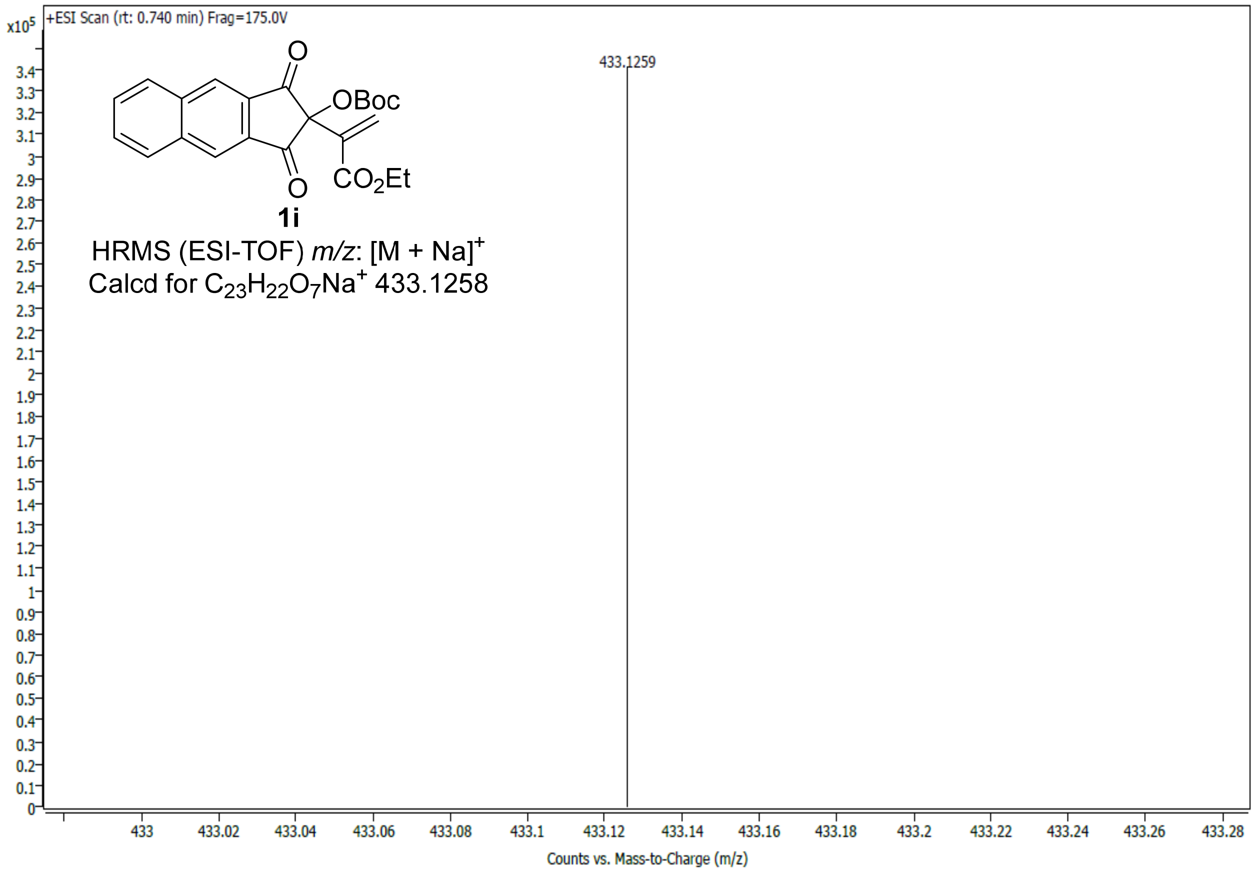
+ESI Scan (rt: 0.740 min) Frag=175.0V
x10^5
OBoc
CO2Et
1i
HRMS (ESI-TOF) m/z: [M + Na]+
Calcd for C23H22O7Na+ 433.1258
433.1259
Counts vs. Mass-to-Charge (m/z)

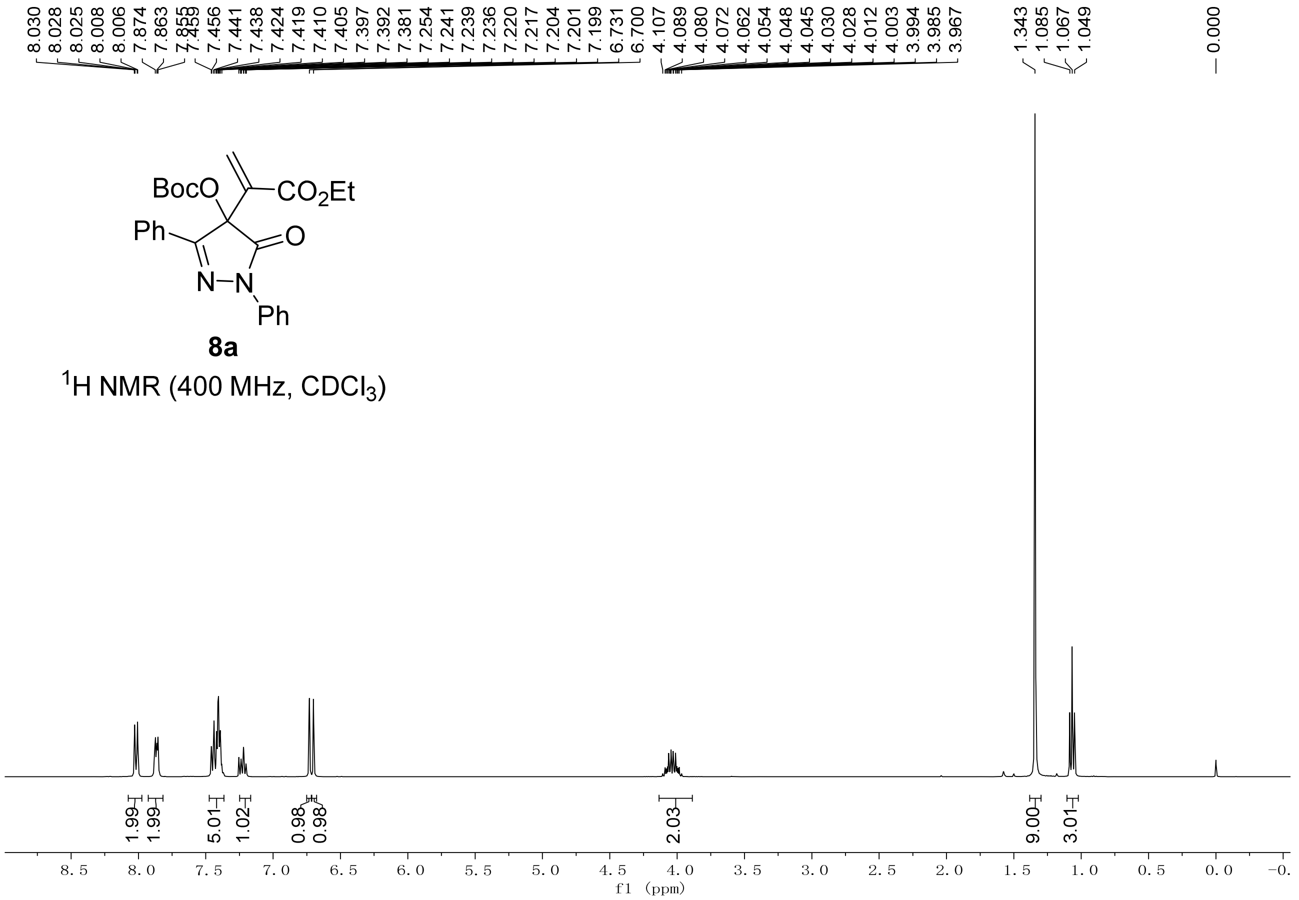
8.030 8.028 8.025 8.008 8.006 7.874 7.863 7.855 7.459 7.456 7.441 7.438 7.424 7.419 7.410 7.405 7.397 7.392 7.381 7.254 7.241 7.239 7.236 7.220 7.217 7.204 7.201 7.199 6.731 6.700 4.107 4.089 4.080 4.072 4.062 4.054 4.048 4.045 4.030 4.028 4.012 4.003 3.994 3.985 3.967 1.343 1.085 1.067 1.049 0.000
BocO
CO2Et
Ph
O
N–N
Ph
8a
1H NMR (400 MHz, CDCl3)
1.99 1.99 5.01 1.02 0.98 0.98 2.03 9.00 3.01
f1 (ppm)

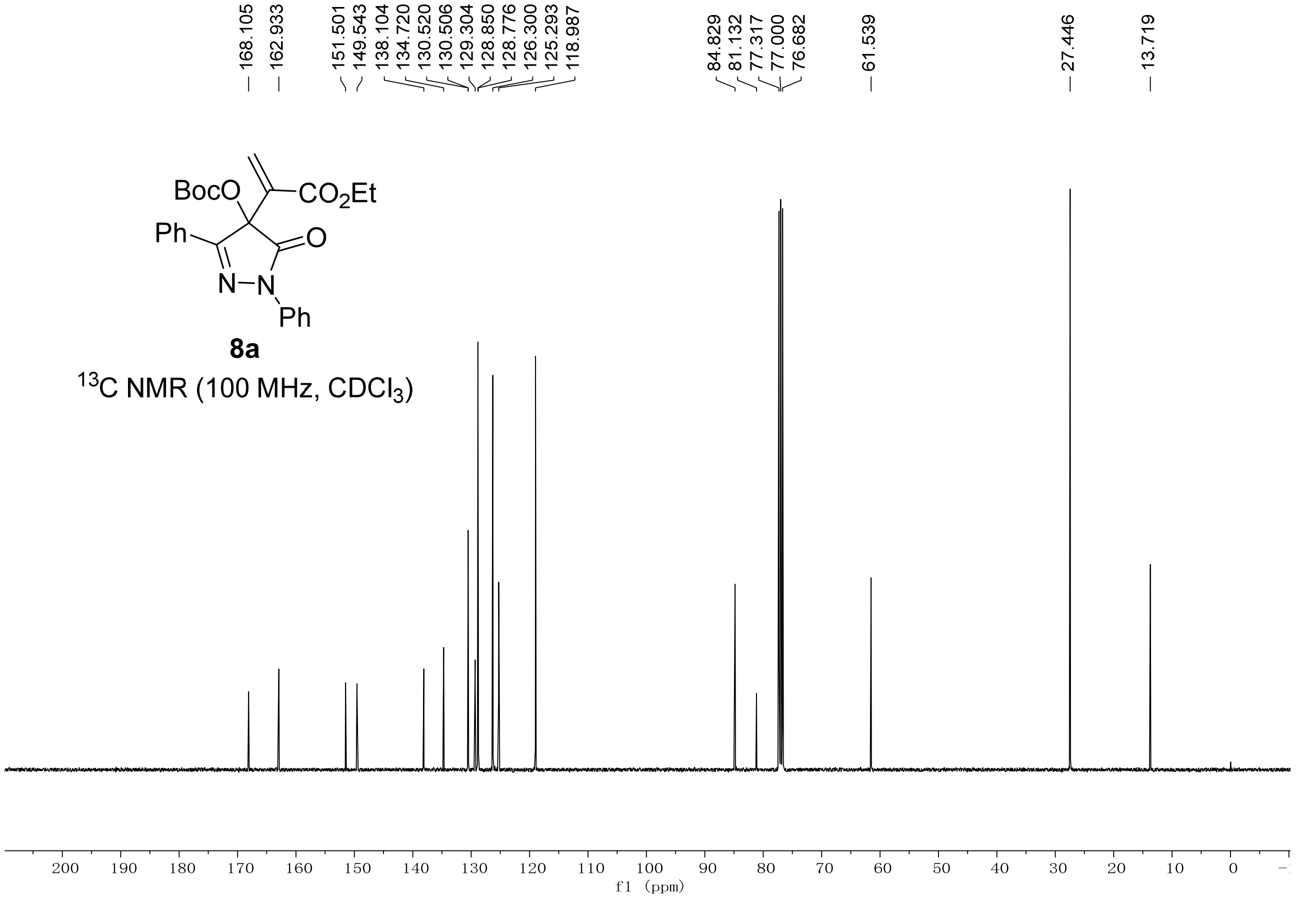
168.105 162.933 151.501 149.543 138.104 134.720 130.520 130.506 129.304 128.850 128.776 126.300 125.293 118.987 84.829 81.132 77.317 77.000 76.682 61.539 27.446 13.719
BocO
CO2Et
Ph
O
N–N
Ph
8a
13C NMR (100 MHz, CDCl3)
f1 (ppm)

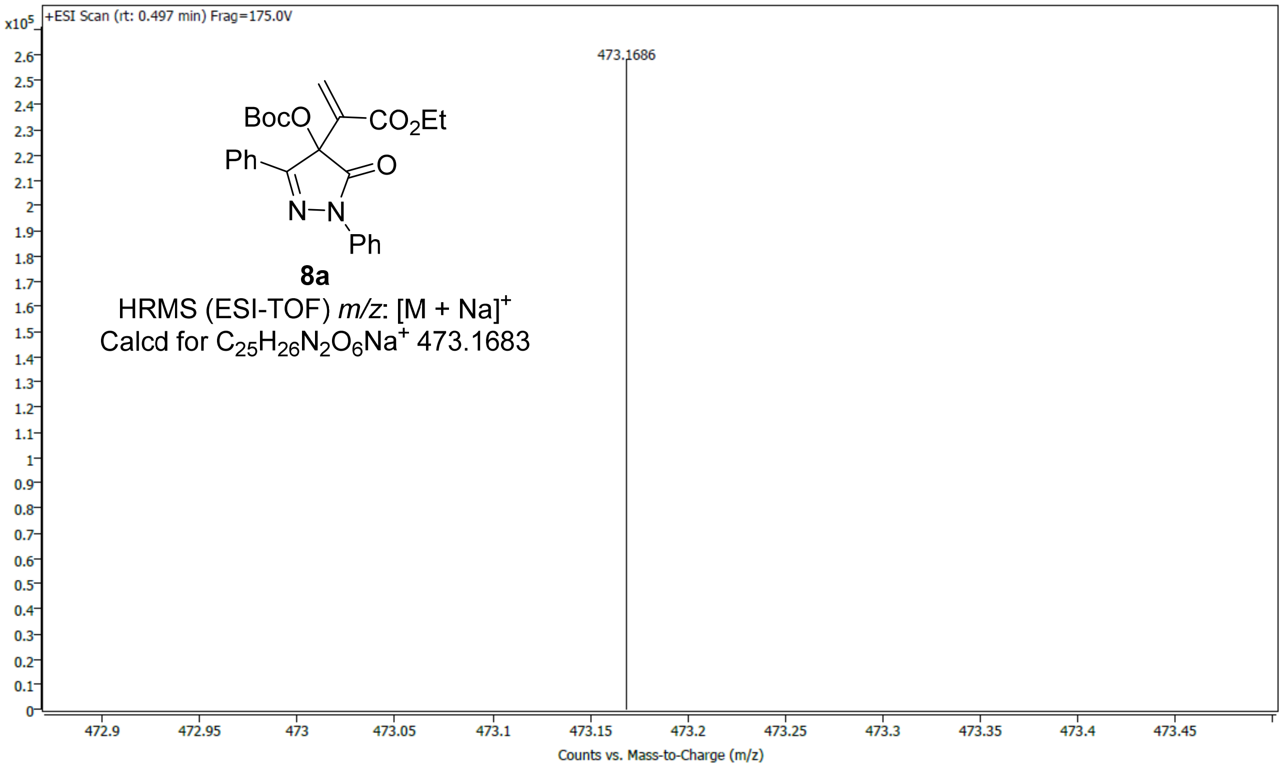
+ESI Scan (rt: 0.497 min) Frag=175.0V
x10^5
473.1686
BocO
CO2Et
Ph
O
N–N
Ph
8a
HRMS (ESI-TOF) m/z: [M + Na]+
Calcd for C25H26N2O6Na+ 473.1683
Counts vs. Mass-to-Charge (m/z)

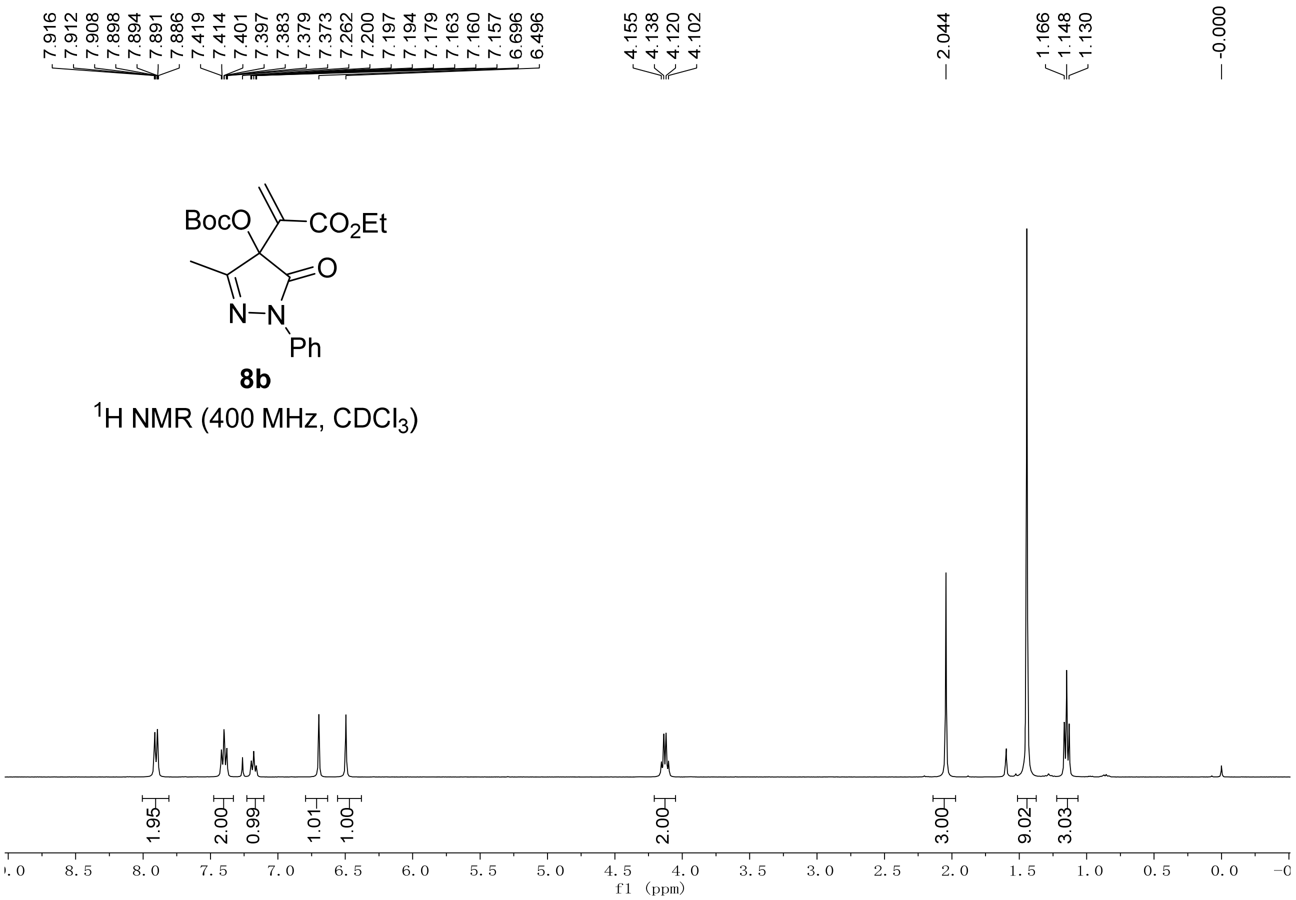
7.916
7.912
7.908
7.898
7.894
7.891
7.886
7.419
7.414
7.401
7.397
7.383
7.379
7.373
7.262
7.200
7.197
7.194
7.179
7.163
7.160
7.157
6.696
6.496
4.155
4.138
4.120
4.102
2.044
1.166
1.148
1.130
-0.000
BocO
CO2Et
O
N–N
Ph
8b
1H NMR (400 MHz, CDCl3)
1.95
2.00
0.99
1.01
1.00
2.00
3.00
9.02
3.03
f1 (ppm)

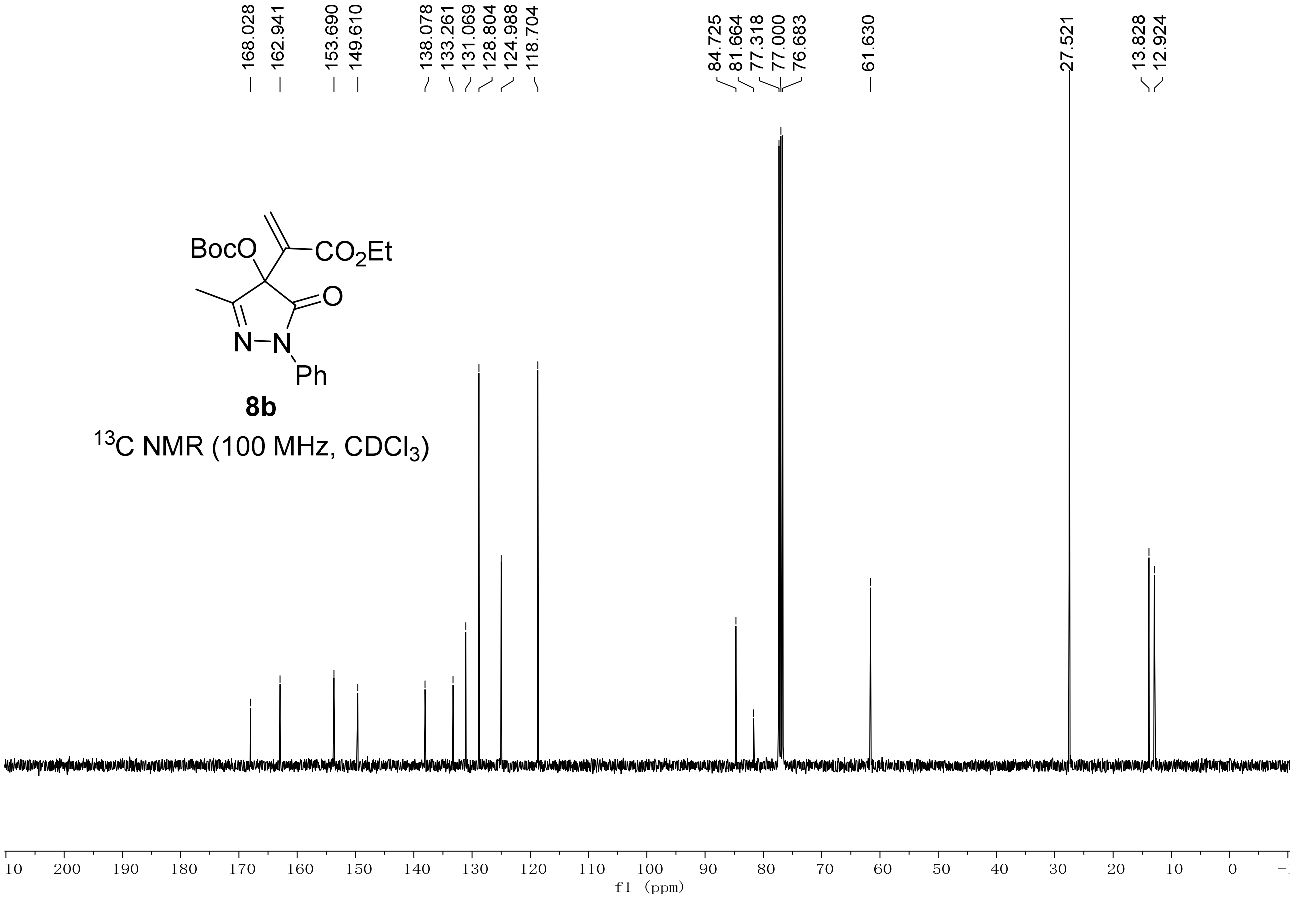
168.028
162.941
153.690
149.610
138.078
133.261
131.069
128.804
124.988
118.704
84.725
81.664
77.318
77.000
76.683
61.630
27.521
13.828
12.924
BocO
CO2Et
O
N–N
Ph
8b
13C NMR (100 MHz, CDCl3)
f1 (ppm)

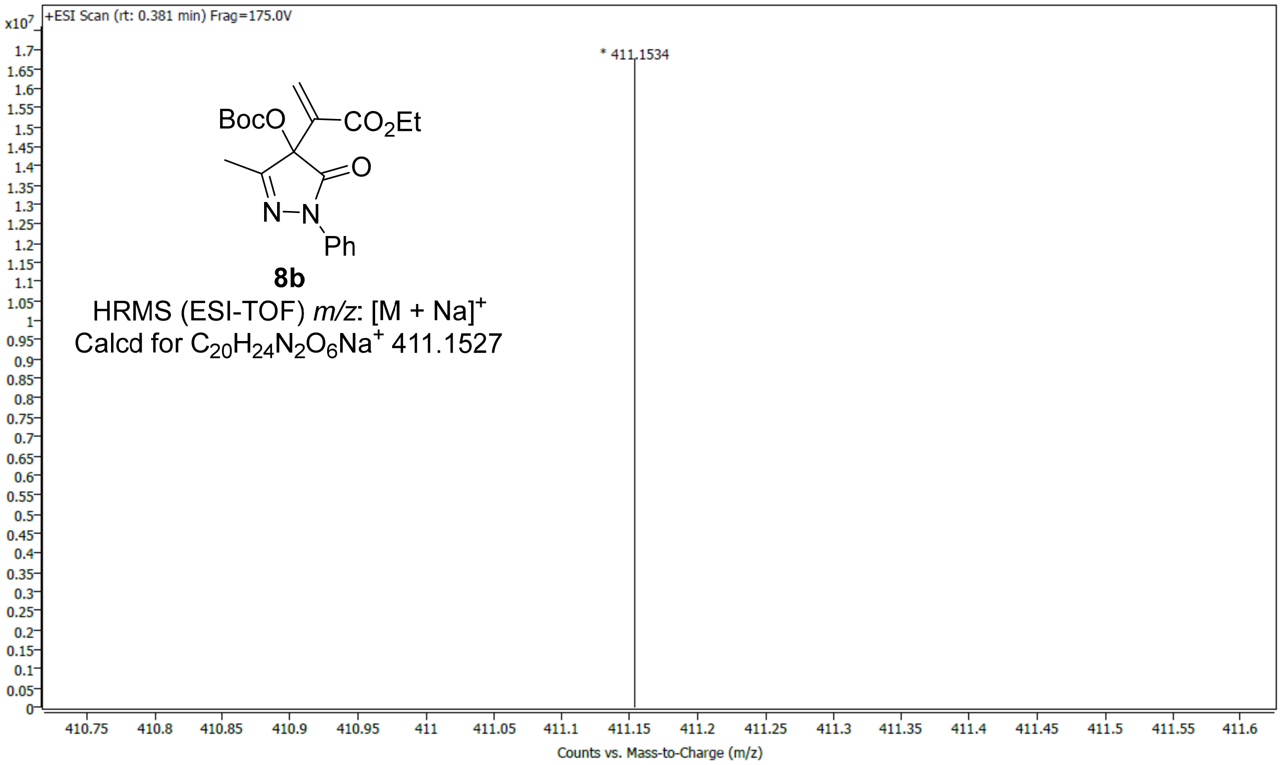
x10^7
+ESI Scan (rt: 0.381 min) Frag=175.0V
* 411.1534
BocO
CO2Et
O
N–N
Ph
8b
HRMS (ESI-TOF) m/z: [M + Na]+
Calcd for C20H24N2O6Na+ 411.1527
Counts vs. Mass-to-Charge (m/z)

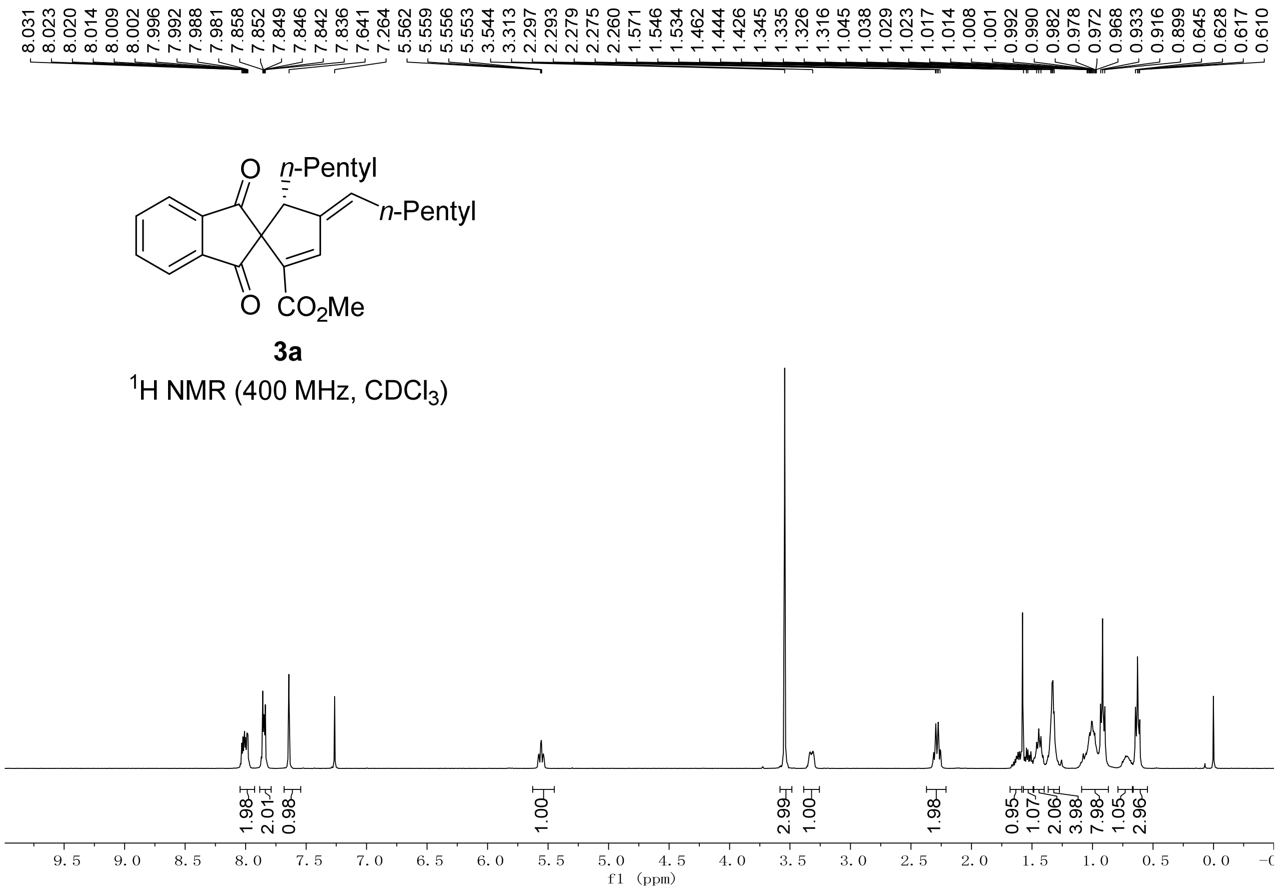
8.031 8.023 8.020 8.014 8.009 8.002 7.996 7.992 7.988 7.981 7.858 7.852 7.849 7.846 7.842 7.836 7.641 7.264 5.562 5.559 5.556 5.553 3.544 3.313 2.297 2.293 2.279 2.275 2.260 1.571 1.546 1.534 1.462 1.444 1.426 1.345 1.335 1.326 1.316 1.045 1.038 1.029 1.023 1.017 1.014 1.008 1.001 0.992 0.990 0.982 0.978 0.972 0.968 0.933 0.916 0.899 0.645 0.628 0.617 0.610
O
n-Pentyl
n-Pentyl
O
CO2Me
3a
1H NMR (400 MHz, CDCl3)
1.98 2.01 0.98 1.00 2.99 1.00 1.98 0.95 1.07 2.06 3.98 7.98 1.05 2.96
9.5 9.0 8.5 8.0 7.5 7.0 6.5 6.0 5.5 5.0 4.5 4.0 3.5 3.0 2.5 2.0 1.5 1.0 0.5 0.0
f1 (ppm)

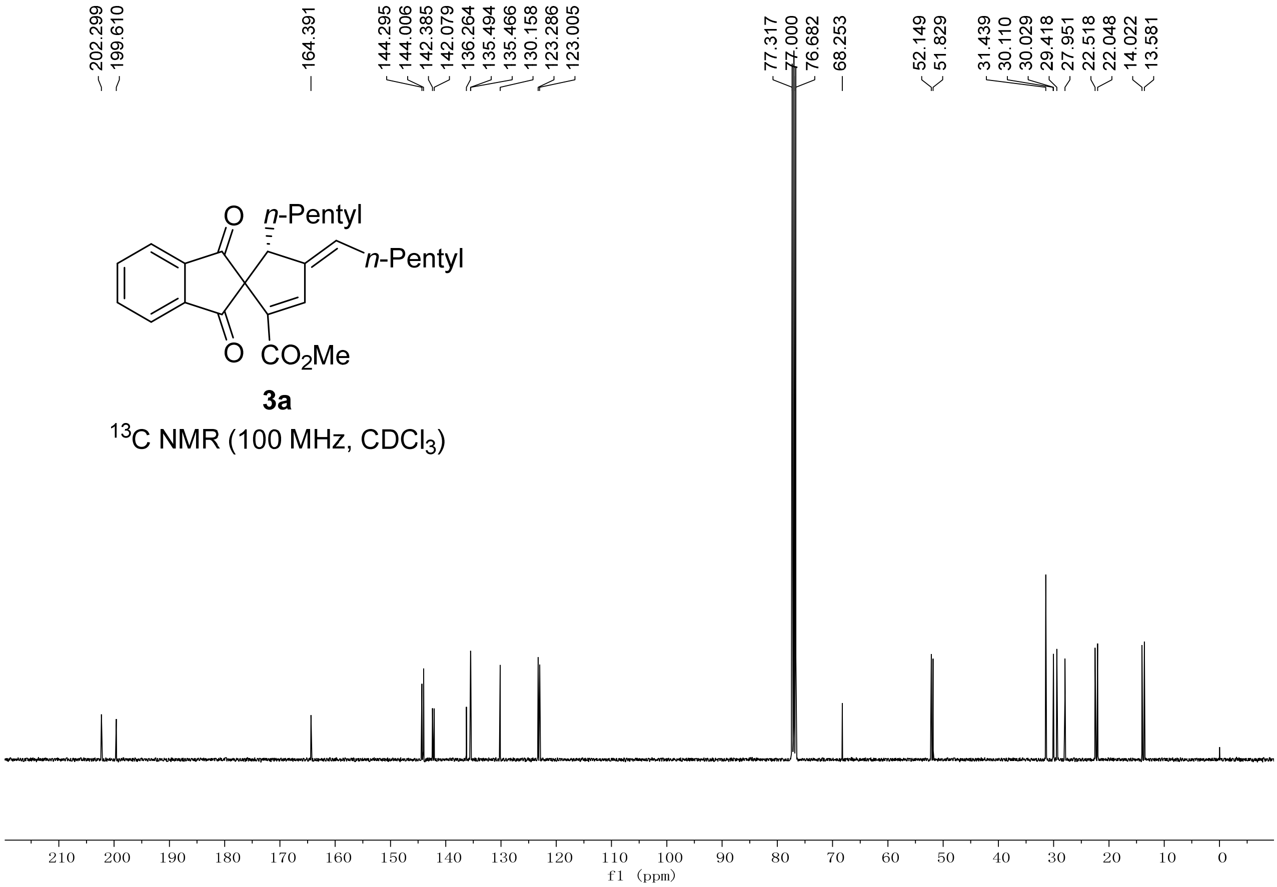
202.299 199.610 164.391 144.295 144.006 142.385 142.079 136.264 135.494 135.466 130.158 123.286 123.005 77.317 77.000 76.682 68.253 52.149 51.829 31.439 30.110 30.029 29.418 27.951 22.518 22.048 14.022 13.581
O
n-Pentyl
n-Pentyl
O
CO2Me
3a
13C NMR (100 MHz, CDCl3)
210 200 190 180 170 160 150 140 130 120 110 100 90 80 70 60 50 40 30 20 10 0
f1 (ppm)

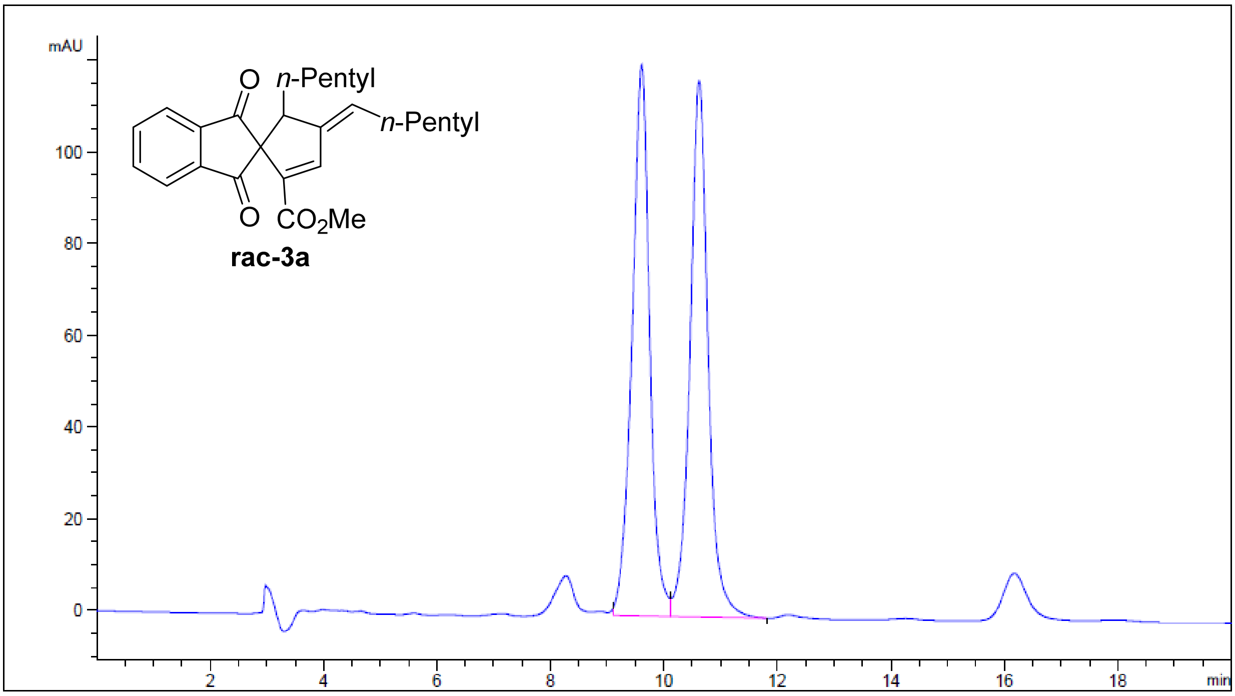

Peak #	RetTime [min]	Type	Width [min]	Area mAU *s	Area %
1	9.608	BV	0.3201	2610.16919	50.0639
2	10.622	VB	0.3297	2603.50854	49.9361

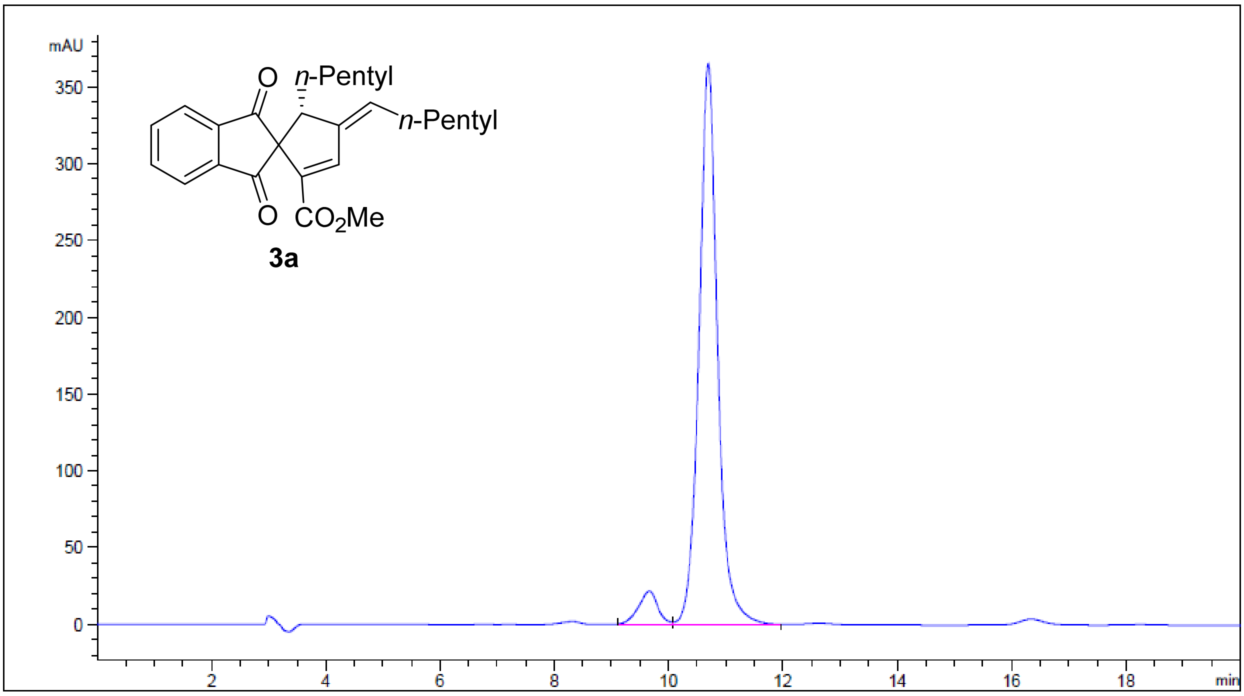

Peak #	RetTime [min]	Type	Width [min]	Area mAU *s	Area %
1	9.666	BV	0.3501	499.07703	5.7003
2	10.694	VB	0.3358	8256.14941	94.2997

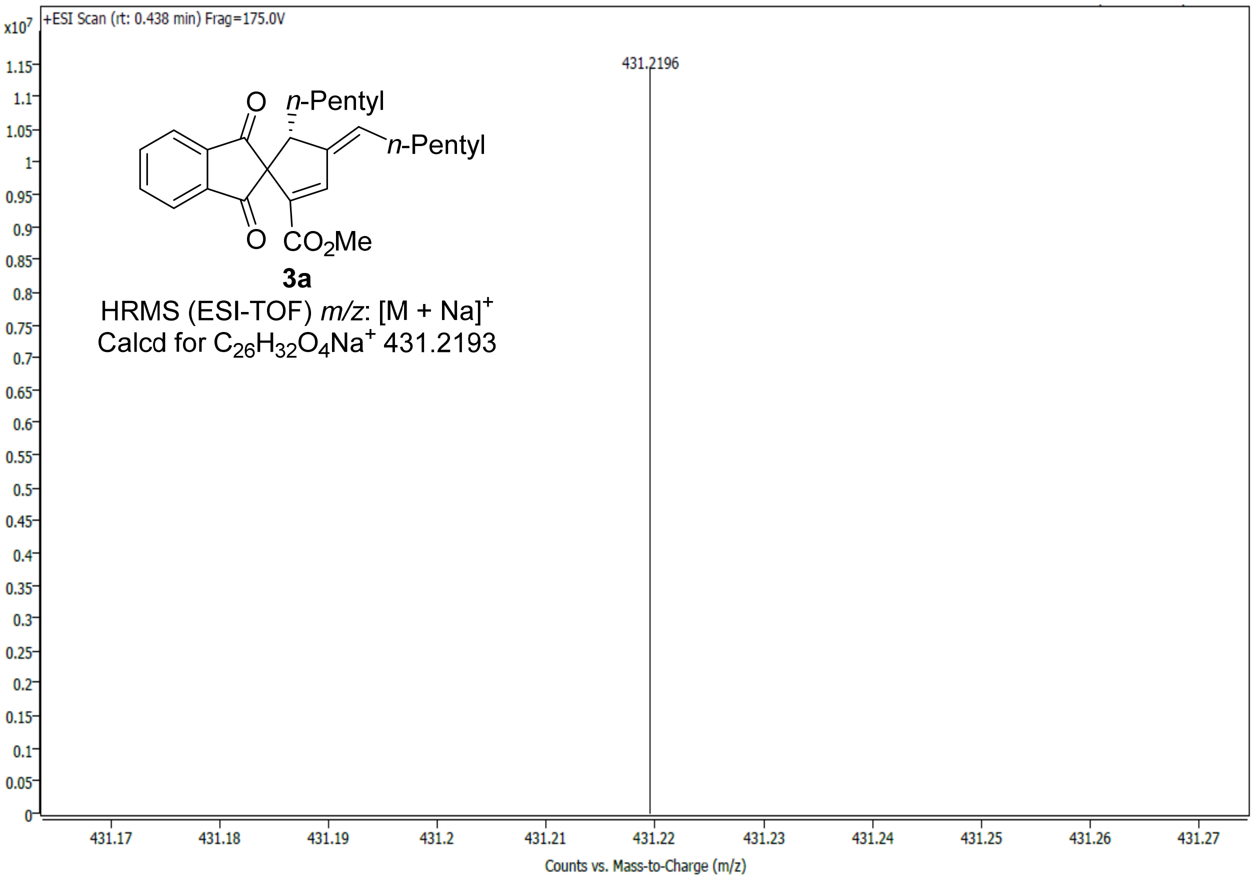
+ESI Scan (rt: 0.438 min) Frag=175.0V
x10^7
431.2196
n-Pentyl
n-Pentyl
O
O
CO2Me
3a
HRMS (ESI-TOF) m/z: [M + Na]+
Calcd for C26H32O4Na+ 431.2193
Counts vs. Mass-to-Charge (m/z)

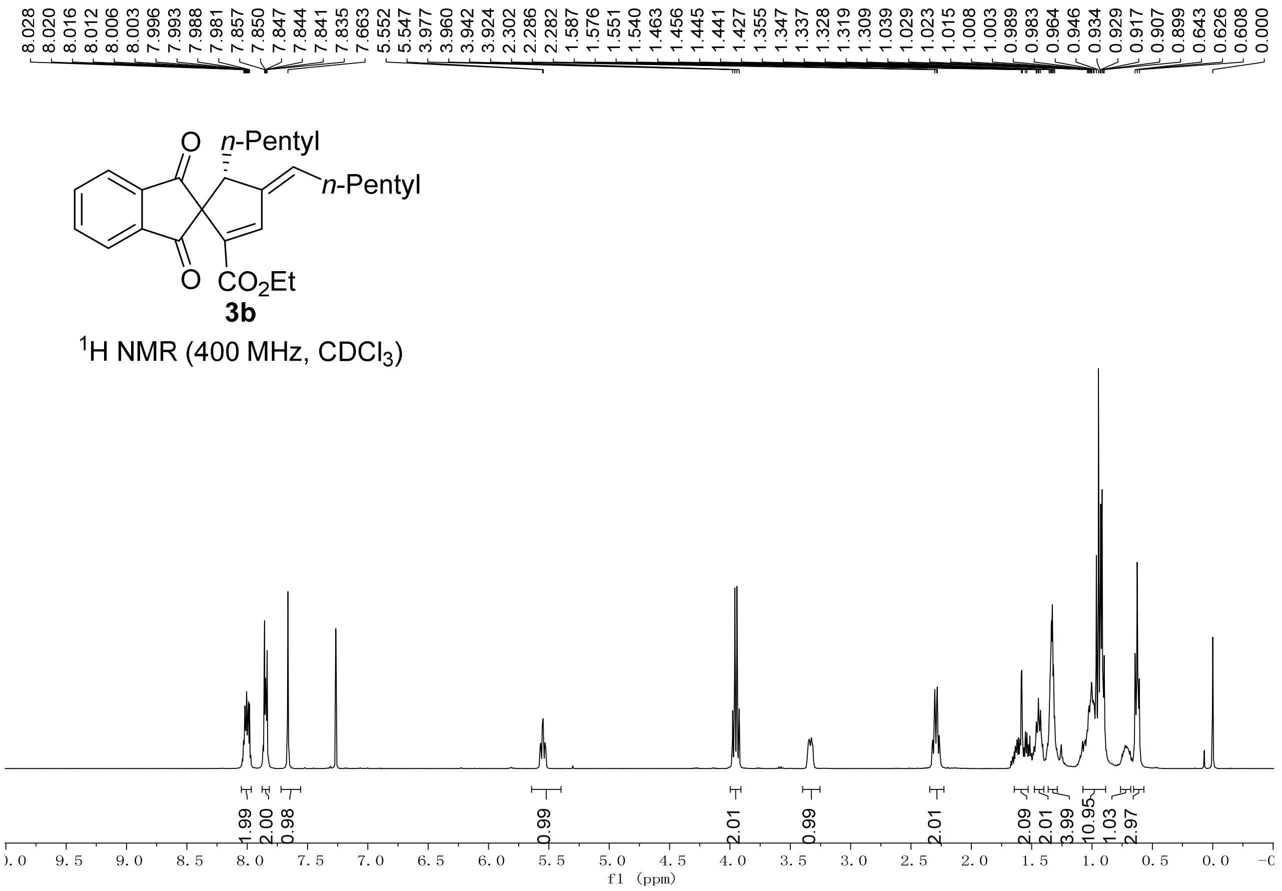
8.028
8.020
8.016
8.012
8.006
8.003
7.996
7.993
7.988
7.981
7.857
7.850
7.847
7.844
7.841
7.835
7.663
5.552
5.547
3.977
3.960
3.942
3.924
2.302
2.286
2.282
1.587
1.576
1.551
1.540
1.463
1.456
1.445
1.441
1.427
1.355
1.347
1.337
1.328
1.319
1.309
1.039
1.029
1.023
1.015
1.008
1.003
0.989
0.983
0.964
0.946
0.934
0.929
0.917
0.907
0.899
0.643
0.626
0.608
0.000
O
n-Pentyl
n-Pentyl
O
CO2Et
3b
1H NMR (400 MHz, CDCl3)
1.99
2.00
0.98
0.99
2.01
0.99
2.01
2.09
2.01
3.99
10.95
1.03
2.97
f1 (ppm)

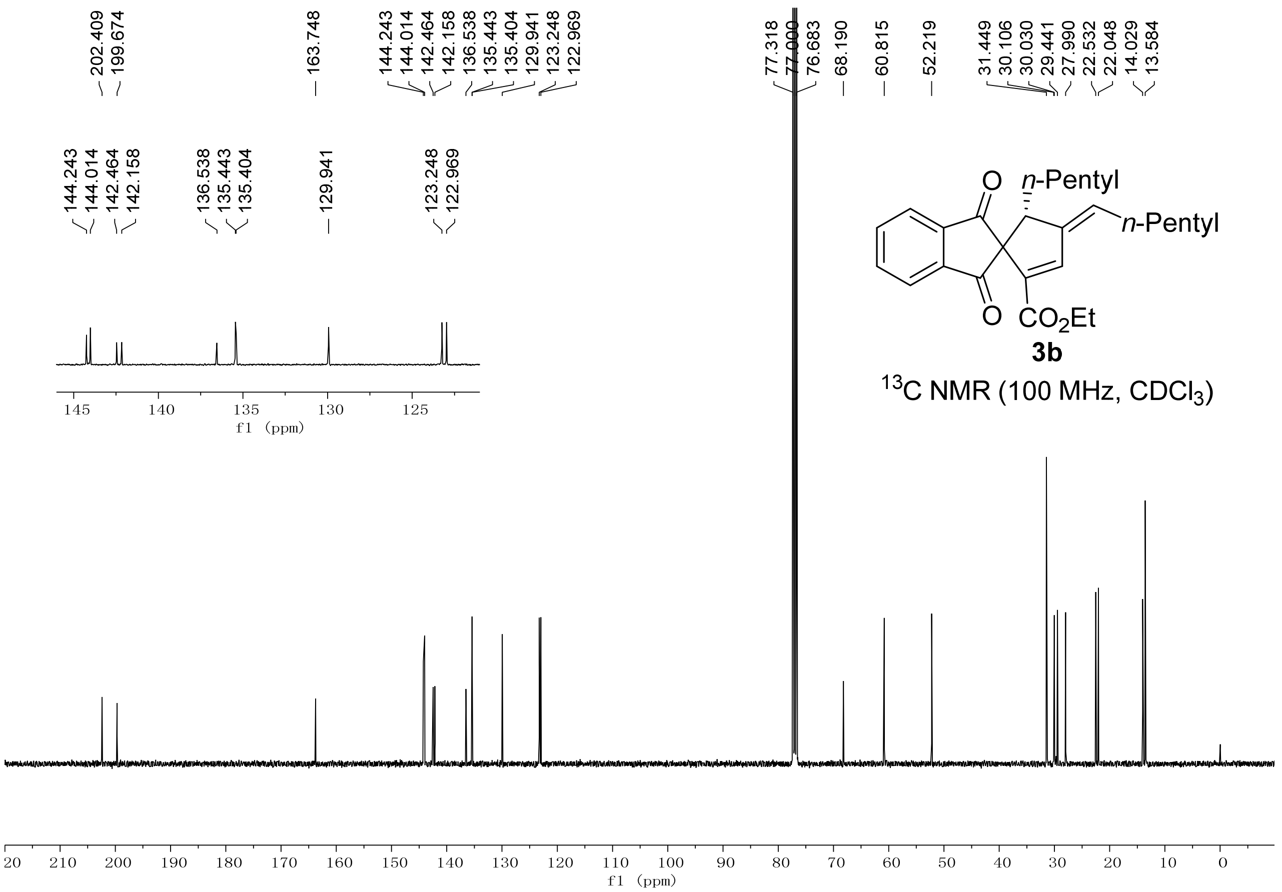
202.409
199.674
163.748
144.243
144.014
142.464
142.158
136.538
135.443
135.404
129.941
123.248
122.969
77.318
77.000
76.683
68.190
60.815
52.219
31.449
30.106
30.030
29.441
27.990
22.532
22.048
14.029
13.584
O
n-Pentyl
n-Pentyl
O
CO2Et
3b
13C NMR (100 MHz, CDCl3)
f1 (ppm)

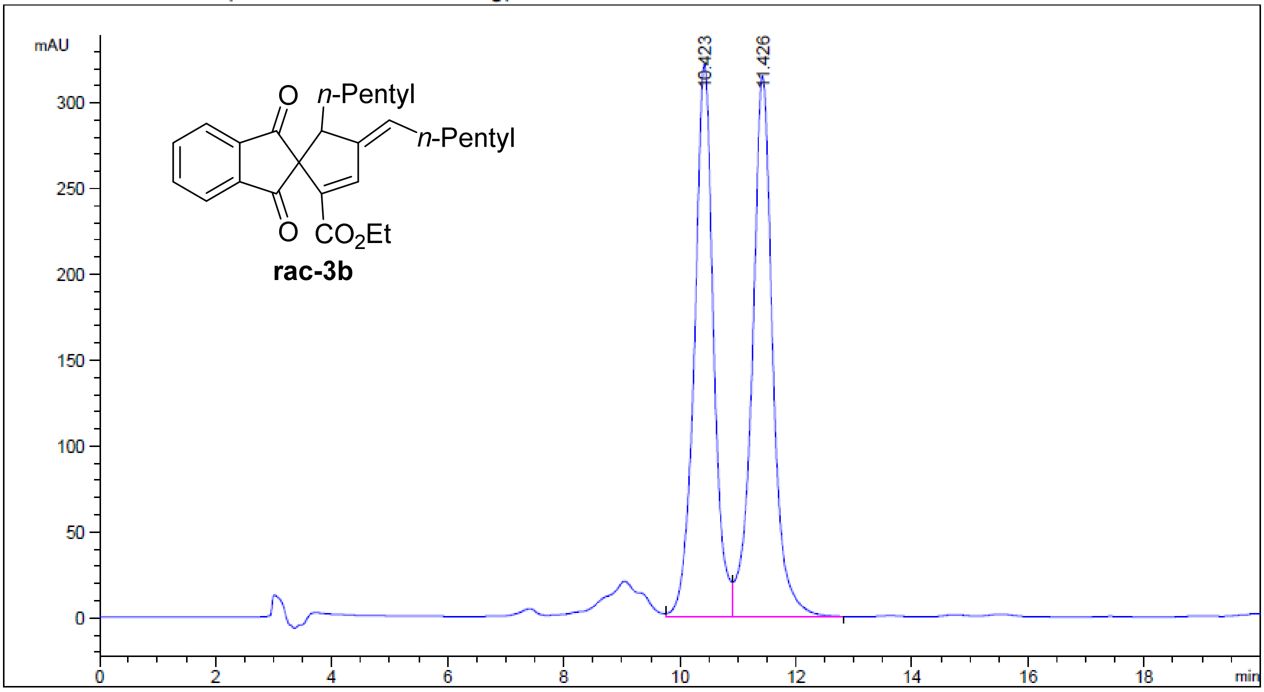

Peak #	RetTime [min]	Type	Width [min]	Area mAU *s	Height [mAU]	Area %
1	10.423	BV	0.3411	7359.95801	321.73007	48.9031
2	11.426	VBA	0.3623	7690.12012	314.69327	51.0969

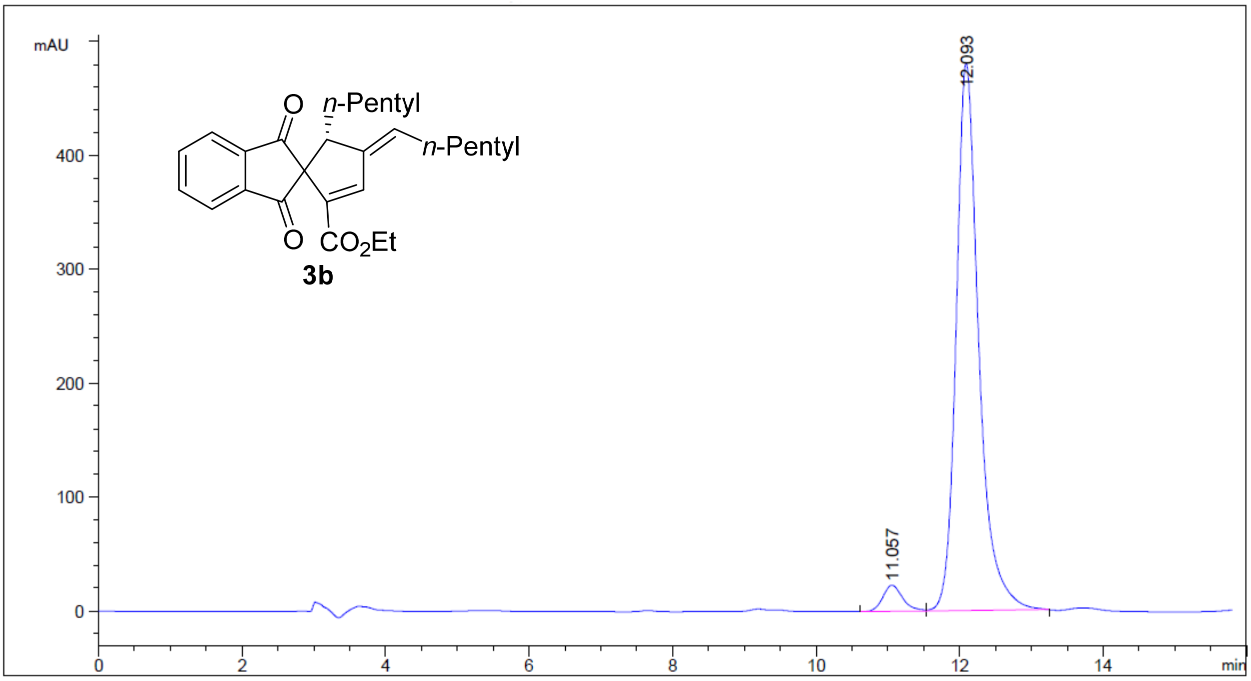

Peak #	RetTime [min]	Type	Width [min]	Area mAU *s	Height [mAU]	Area %
1	11.057	BV	0.3013	446.20990	23.07481	4.0661
2	12.093	VBA	0.3318	1.05277e4	479.69836	95.9339

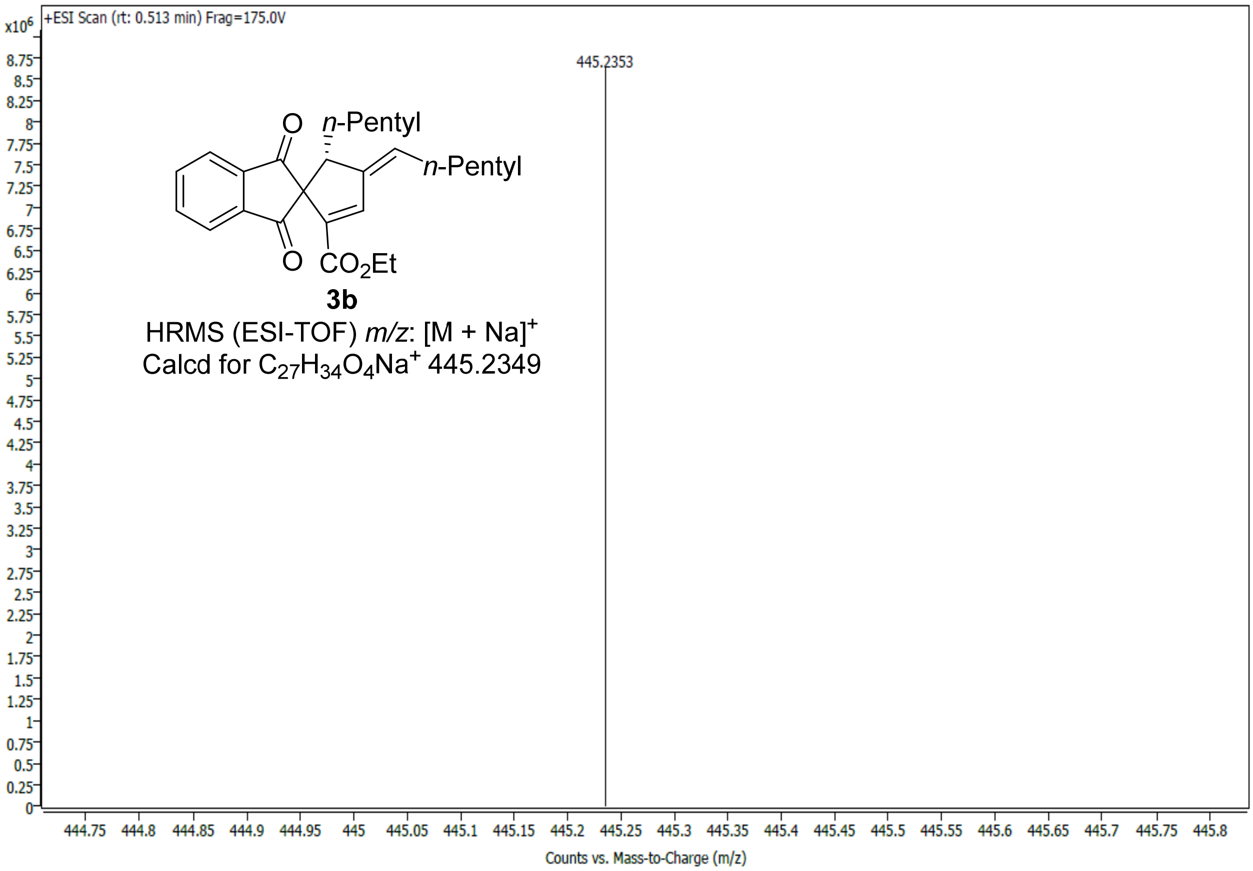
+ESI Scan (rt: 0.513 min) Frag=175.0V
x10^6
445.2353
O
n-Pentyl
n-Pentyl
O
CO2Et
3b
HRMS (ESI-TOF) m/z: [M + Na]+
Calcd for C27H34O4Na+ 445.2349
Counts vs. Mass-to-Charge (m/z)

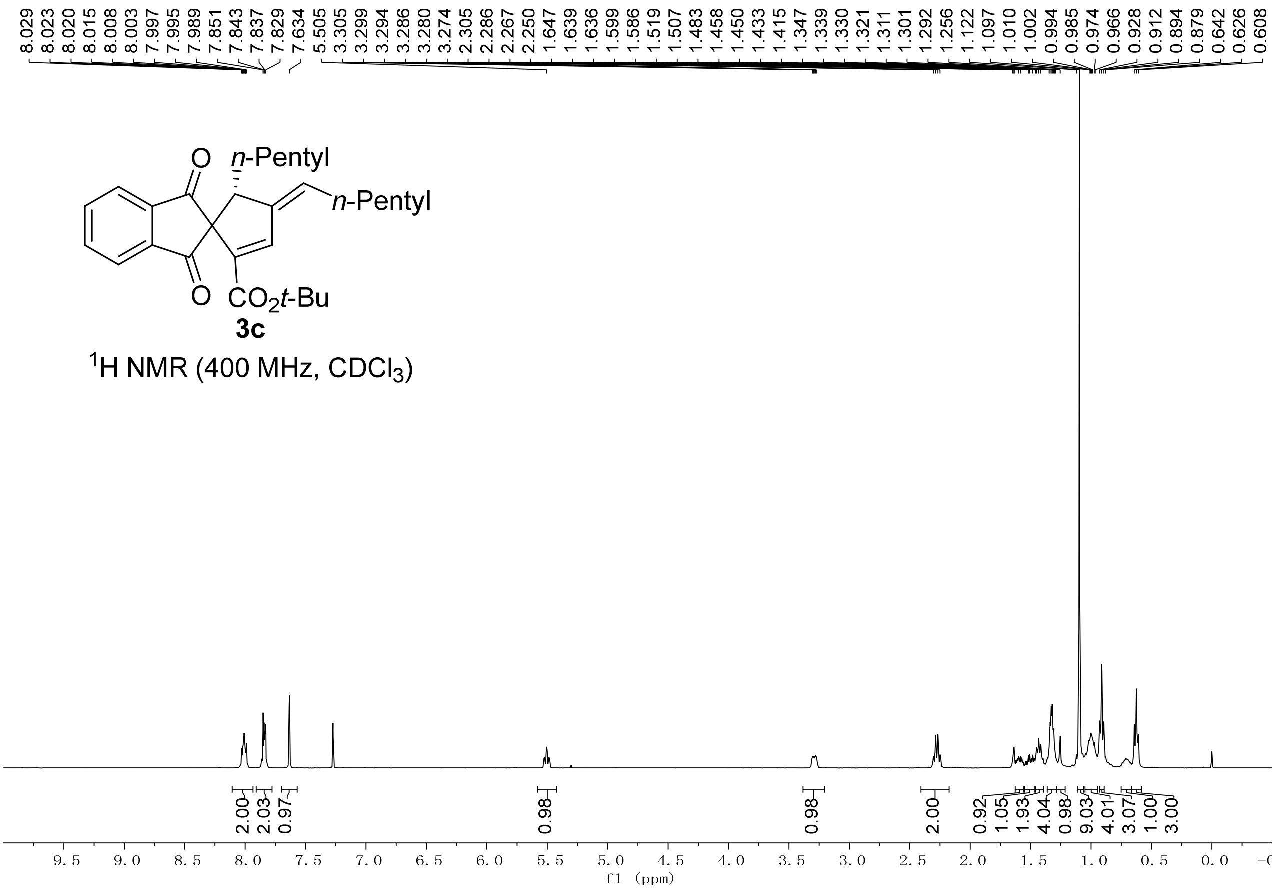
8.029
8.023
8.020
8.015
8.008
8.003
7.997
7.995
7.989
7.851
7.843
7.837
7.829
7.634
5.505
3.305
3.299
3.294
3.286
3.280
3.274
2.305
2.286
2.267
2.250
1.647
1.639
1.636
1.599
1.586
1.519
1.507
1.483
1.458
1.450
1.433
1.415
1.347
1.339
1.330
1.321
1.311
1.301
1.292
1.256
1.122
1.097
1.010
1.002
0.994
0.985
0.974
0.966
0.928
0.912
0.894
0.879
0.642
0.626
0.608
O
n-Pentyl
n-Pentyl
O
CO2t-Bu
3c
1H NMR (400 MHz, CDCl3)
2.00
2.03
0.97
0.98
0.98
2.00
0.92
1.05
1.93
4.04
0.98
9.03
4.01
3.07
1.00
3.00
f1 (ppm)

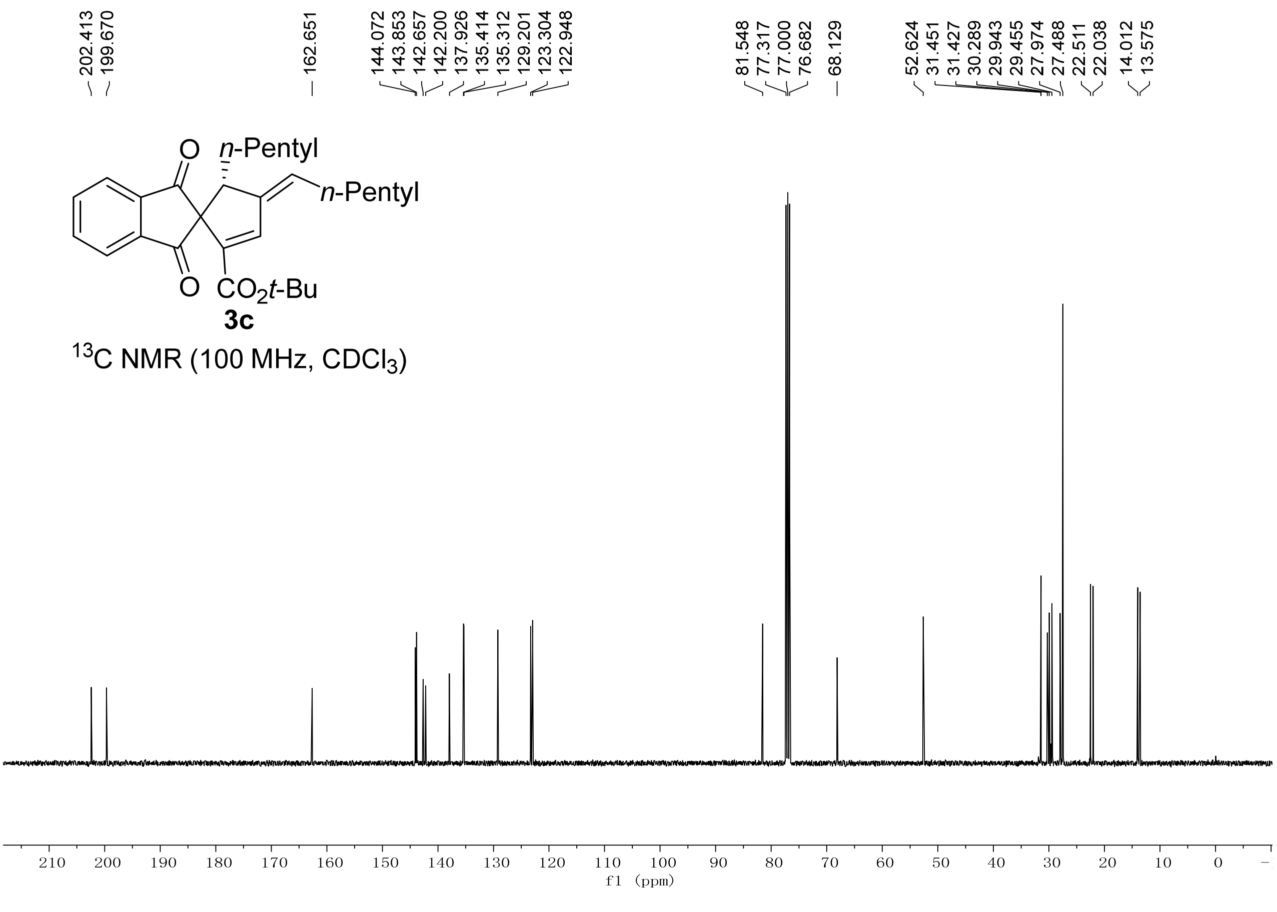
202.413
199.670
162.651
144.072
143.853
142.657
142.200
137.926
135.414
135.312
129.201
123.304
122.948
81.548
77.317
77.000
76.682
68.129
52.624
31.451
31.427
30.289
29.943
29.455
27.974
27.488
22.511
22.038
14.012
13.575
O
n-Pentyl
n-Pentyl
O
CO2t-Bu
3c
13C NMR (100 MHz, CDCl3)
f1 (ppm)

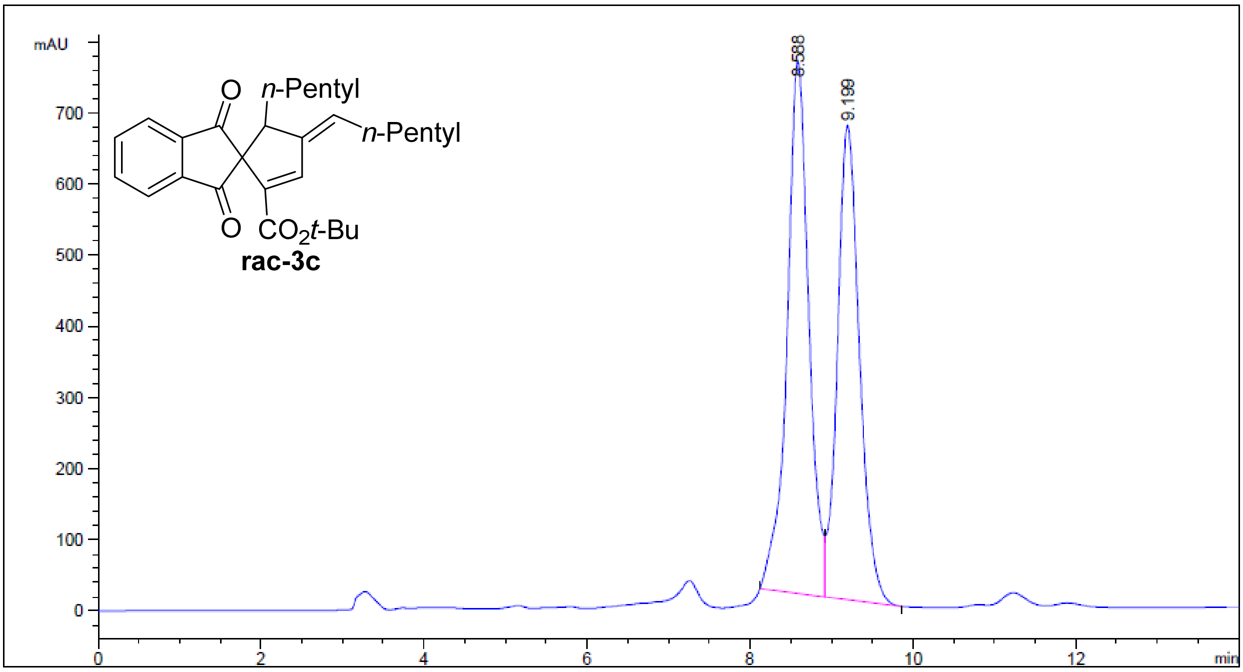

Peak #	RetTime [min]	Type	Width [min]	Area mAU *s	Height [mAU]	Area %
1	8.588	BV	0.2818	1.39205e4	745.74219	52.5413
2	9.199	VBA	0.2900	1.25739e4	666.46576	47.4587

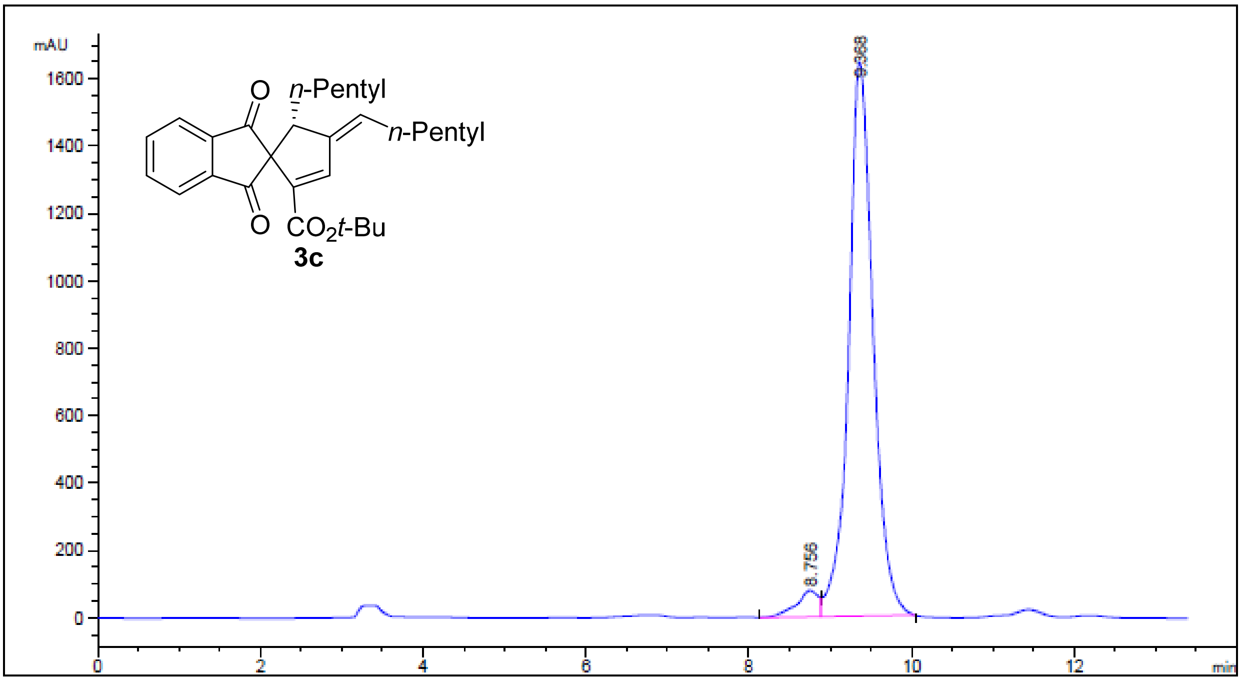

Peak #	RetTime [min]	Type	Width [min]	Area mAU *s	Height [mAU]	Area %
1	8.756	BV	0.2704	1458.59497	78.06558	4.0663
2	9.368	VBA	0.3143	3.44120e4	1642.77612	95.9337

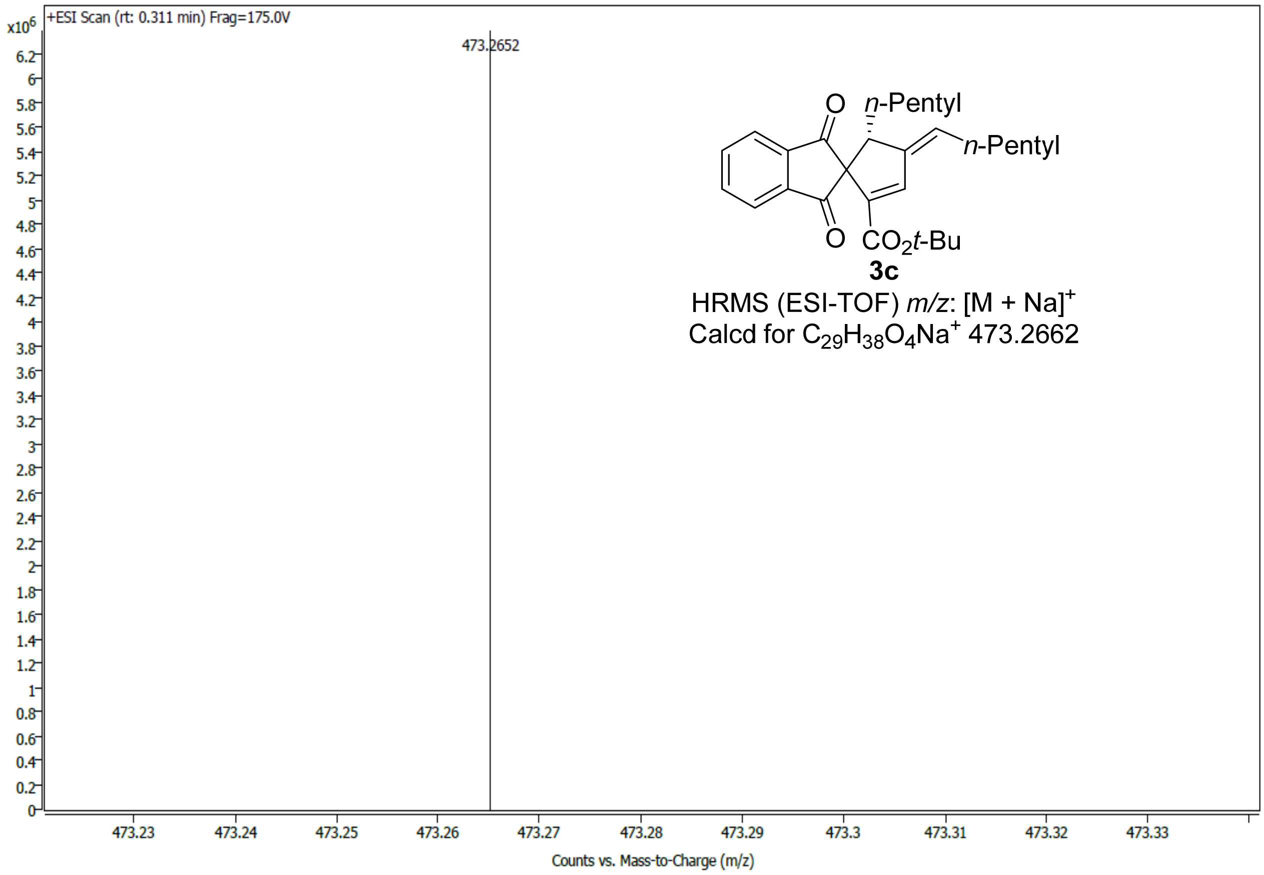

+ESI Scan (rt: 0.311 min) Frag=175.0V
x10^6
473.2652
O
n-Pentyl
n-Pentyl
O
CO2t-Bu
3c
HRMS (ESI-TOF) m/z: [M + Na]+
Calcd for C29H38O4Na+ 473.2662
473.23
473.24
473.25
473.26
473.27
473.28
473.29
473.3
473.31
473.32
473.33
Counts vs. Mass-to-Charge (m/z)

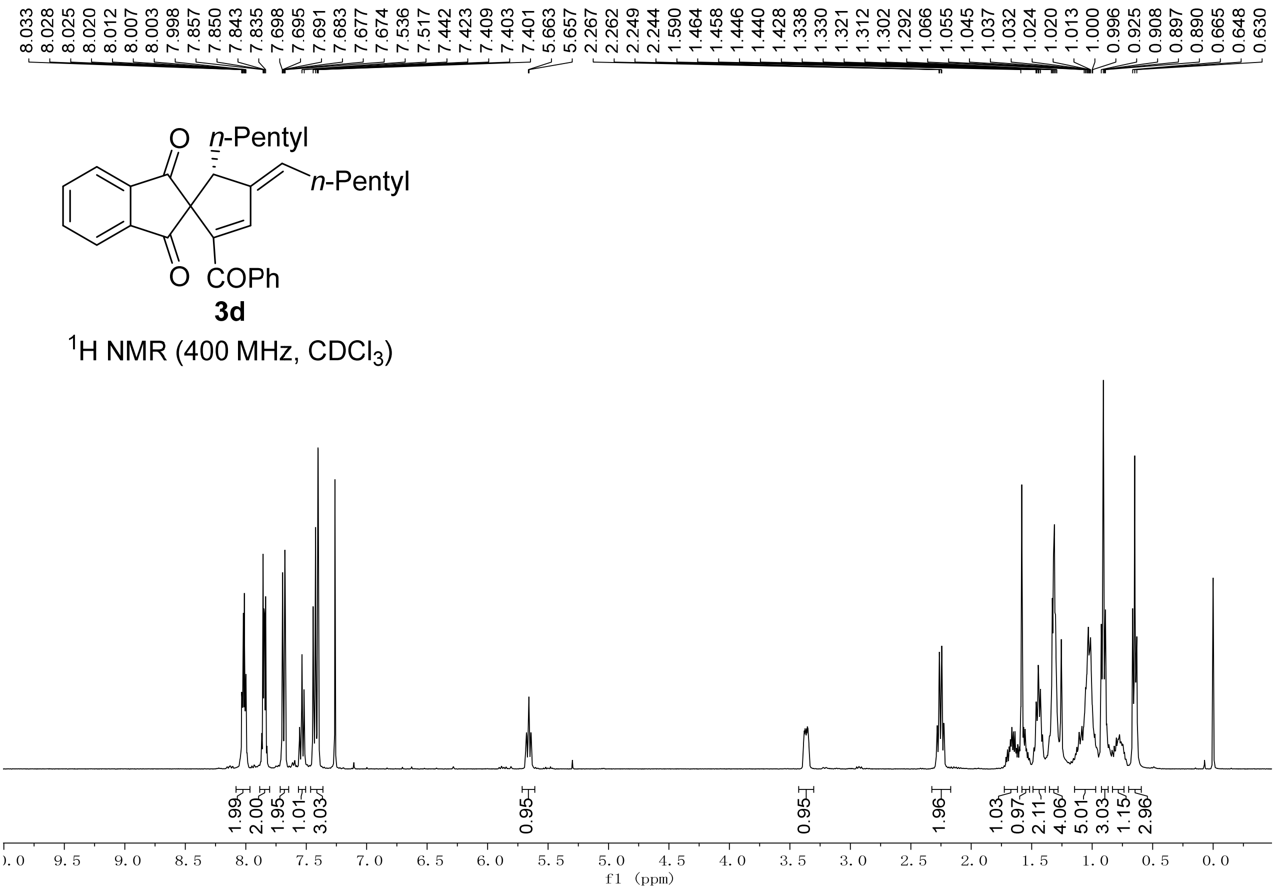
8.033
8.028
8.025
8.020
8.012
8.007
8.003
7.998
7.857
7.850
7.843
7.835
7.698
7.695
7.691
7.683
7.677
7.674
7.536
7.517
7.442
7.423
7.409
7.403
7.401
5.663
5.657
2.267
2.262
2.249
2.244
1.590
1.464
1.458
1.446
1.440
1.428
1.338
1.330
1.321
1.312
1.302
1.292
1.066
1.055
1.045
1.037
1.032
1.024
1.020
1.013
1.000
0.996
0.925
0.908
0.897
0.890
0.665
0.648
0.630
O
n-Pentyl
n-Pentyl
O
COPh
3d
1H NMR (400 MHz, CDCl3)
1.99
2.00
1.95
1.01
3.03
0.95
0.95
1.96
1.03
0.97
2.11
4.06
5.01
3.03
1.15
2.96
9.5 9.0 8.5 8.0 7.5 7.0 6.5 6.0 5.5 5.0 4.5 4.0 3.5 3.0 2.5 2.0 1.5 1.0 0.5 0.0
f1 (ppm)

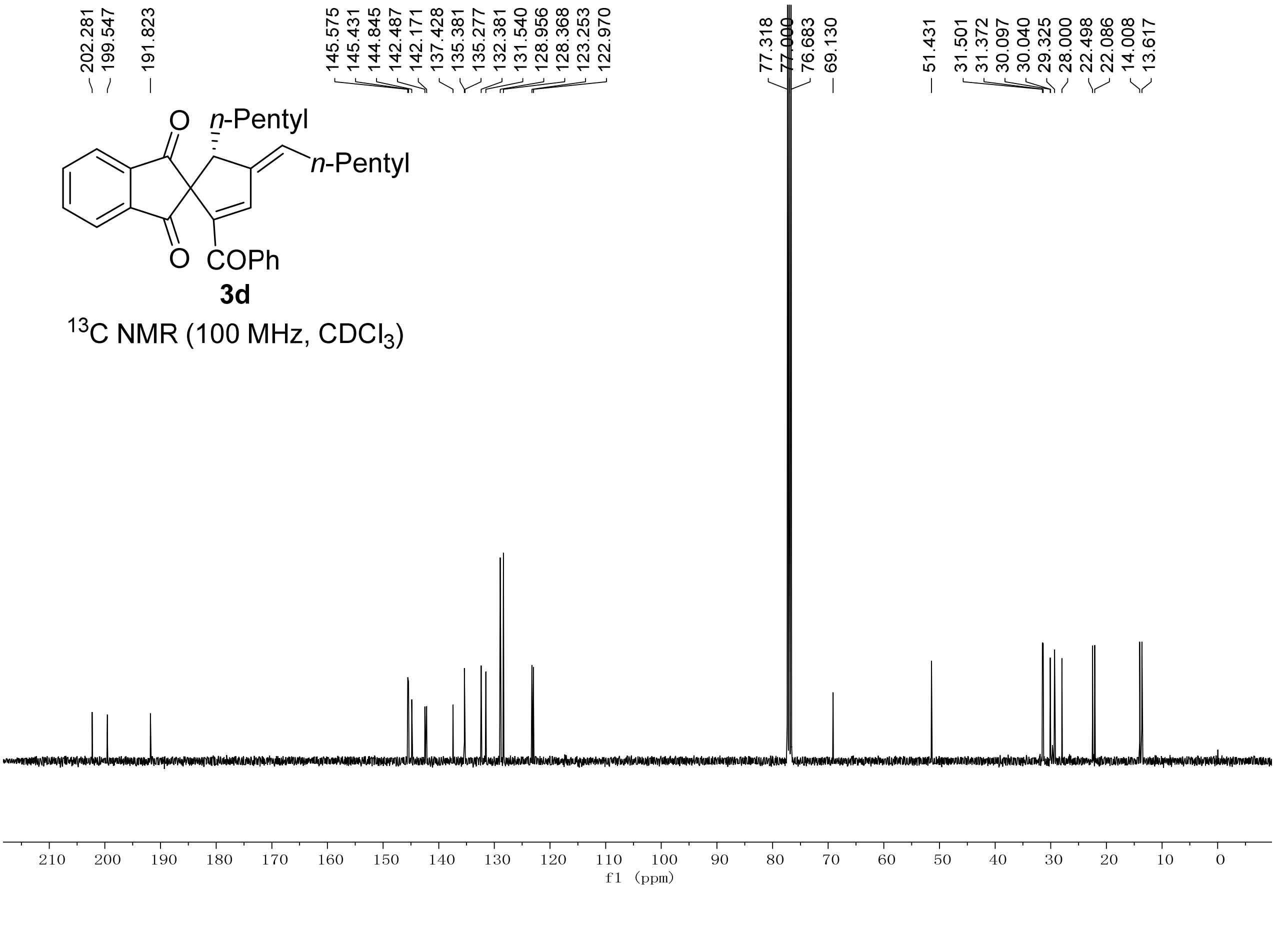
202.281
199.547
191.823
145.575
145.431
144.845
142.487
142.171
137.428
135.381
135.277
132.381
131.540
128.956
128.368
123.253
122.970
77.318
77.000
76.683
69.130
51.431
31.501
31.372
30.097
30.040
29.325
28.000
22.498
22.086
14.008
13.617
O
n-Pentyl
n-Pentyl
O
COPh
3d
13C NMR (100 MHz, CDCl3)
210 200 190 180 170 160 150 140 130 120 110 100 90 80 70 60 50 40 30 20 10 0
f1 (ppm)

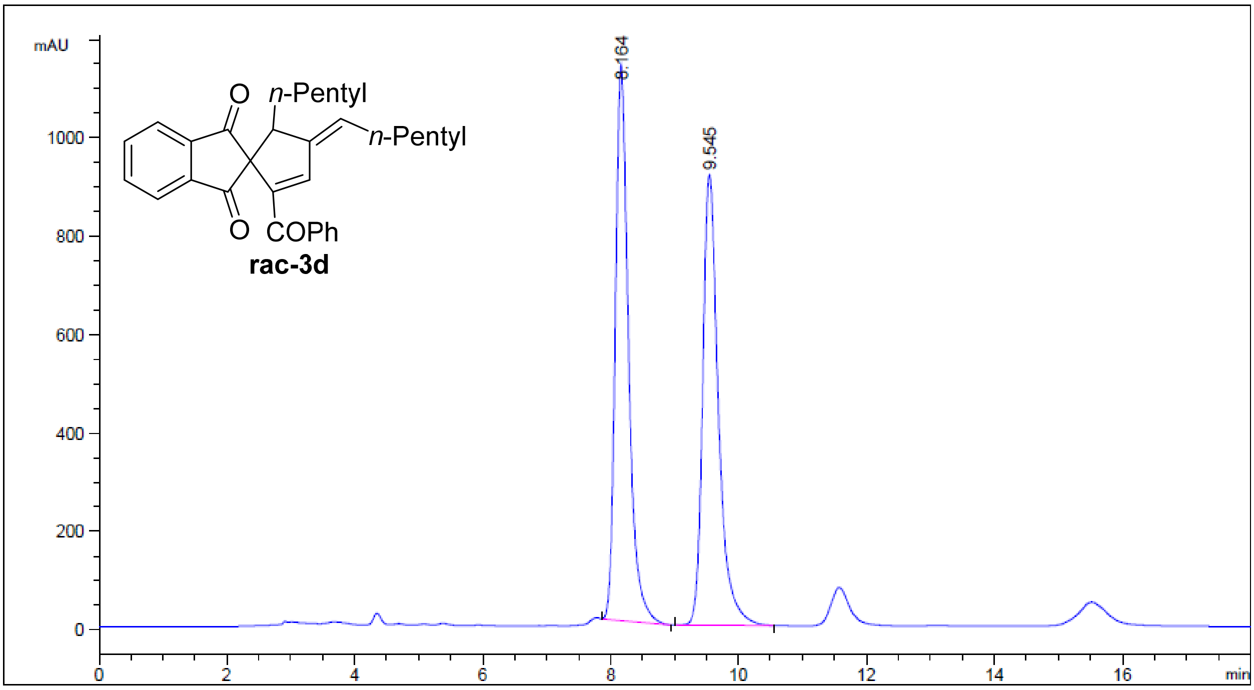

Peak #	RetTime [min]	Type	Width [min]	Area mAU *s	Height [mAU]	Area %
1	8.164	BB	0.2162	1.61530e4	1130.80164	50.8815
2	9.545	VBA	0.2555	1.55933e4	915.70392	49.1185

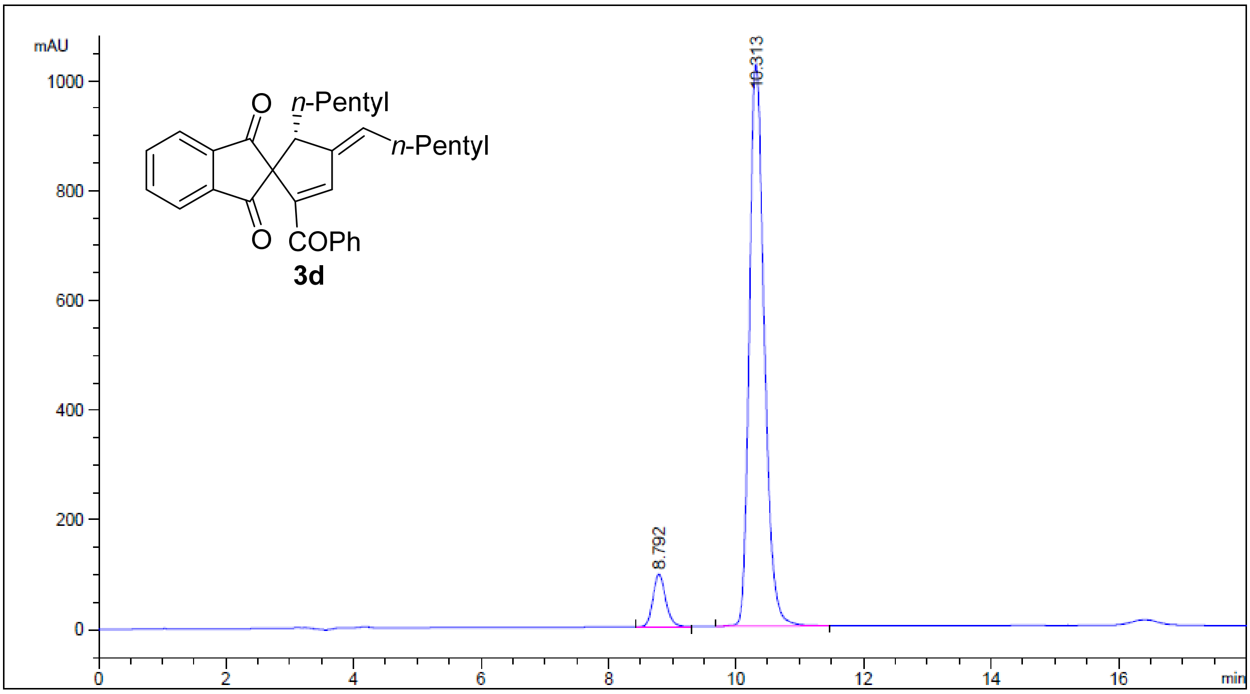

Peak #	RetTime [min]	Type	Width [min]	Area mAU *s	Height [mAU]	Area %
1	8.792	VB	0.2155	1333.23438	95.38301	7.3965
2	10.313	BB	0.2515	1.66919e4	1023.13593	92.6035

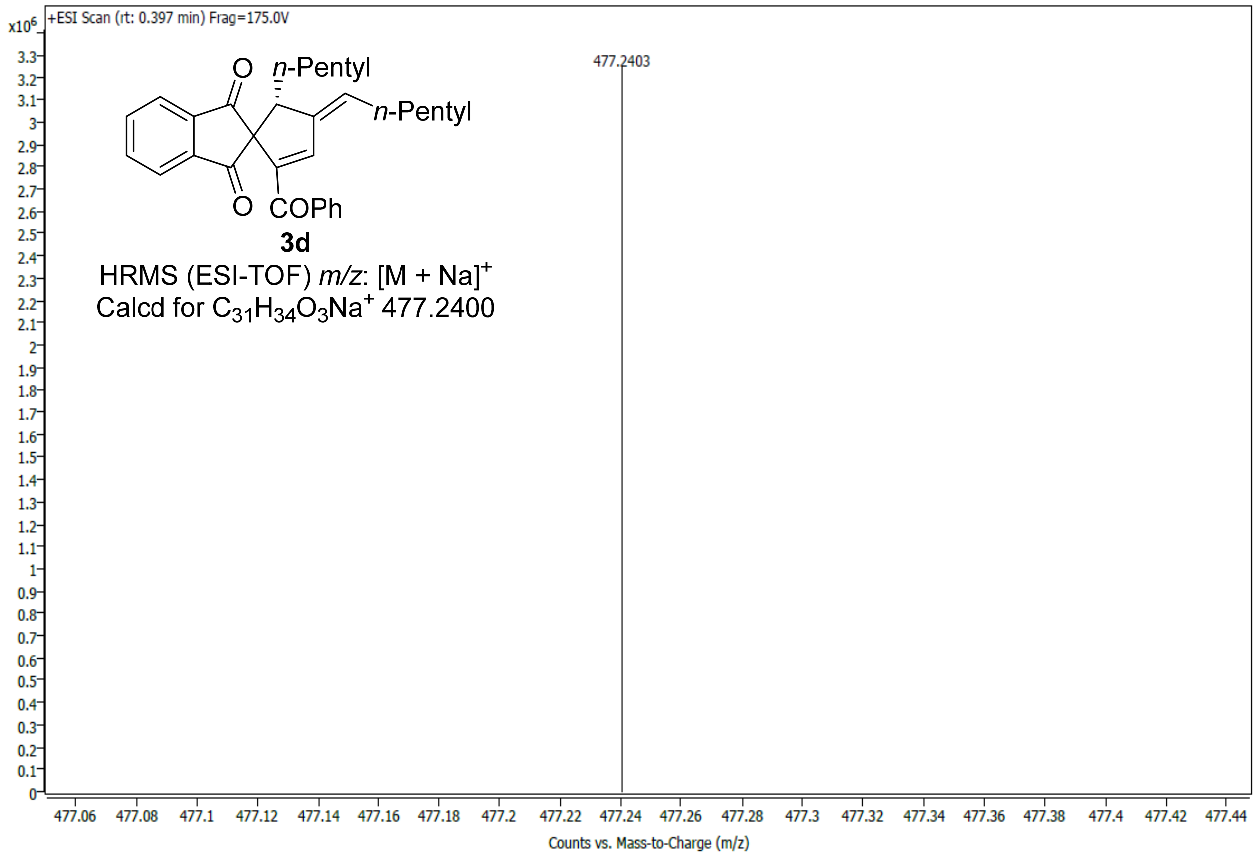

x10^6
+ESI Scan (rt: 0.397 min) Frag=175.0V
O
n-Pentyl
n-Pentyl
O
COPh
3d
HRMS (ESI-TOF) m/z: [M + Na]+
Calcd for C31H34O3Na+ 477.2400
477.2403
Counts vs. Mass-to-Charge (m/z)

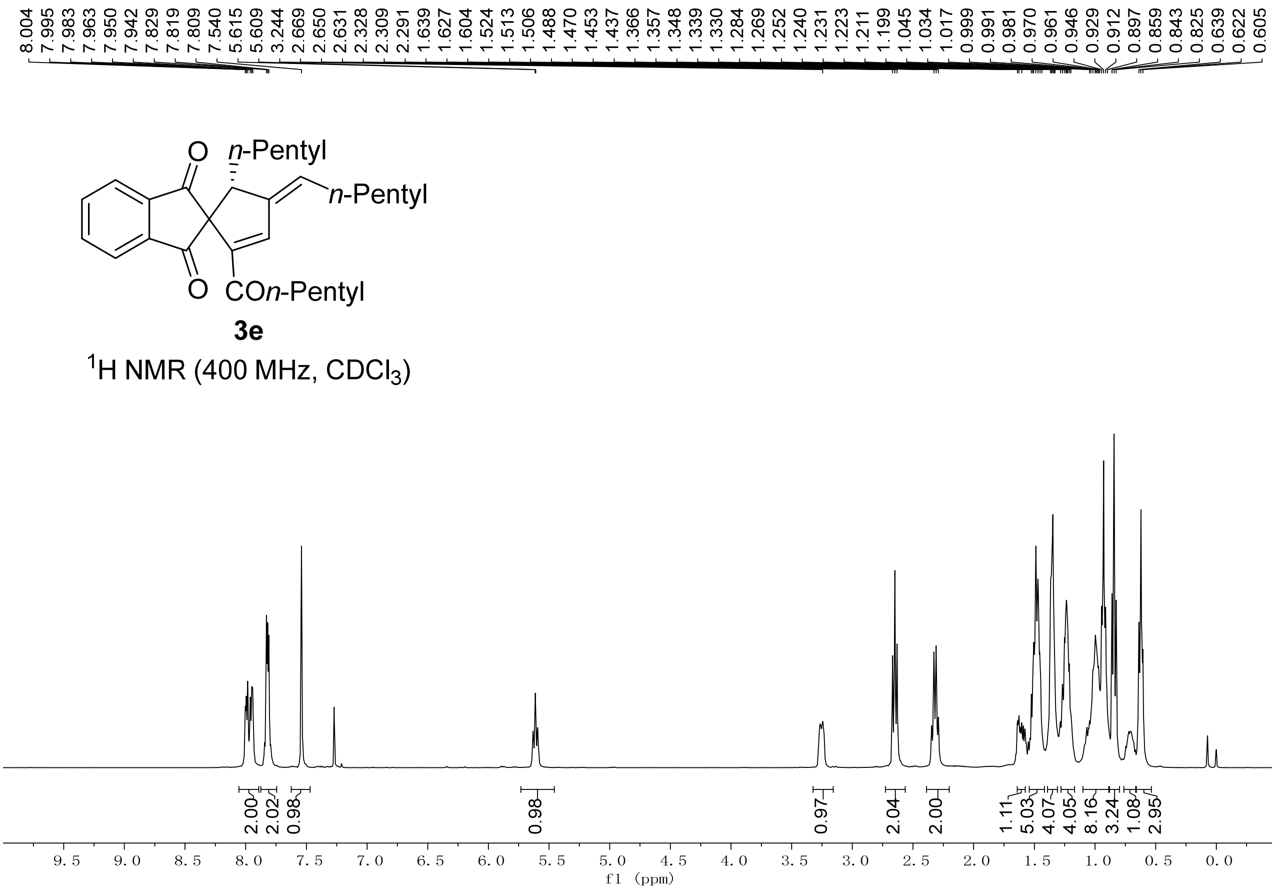

3e

^{1}H NMR (400 MHz, $CDCl_3$)

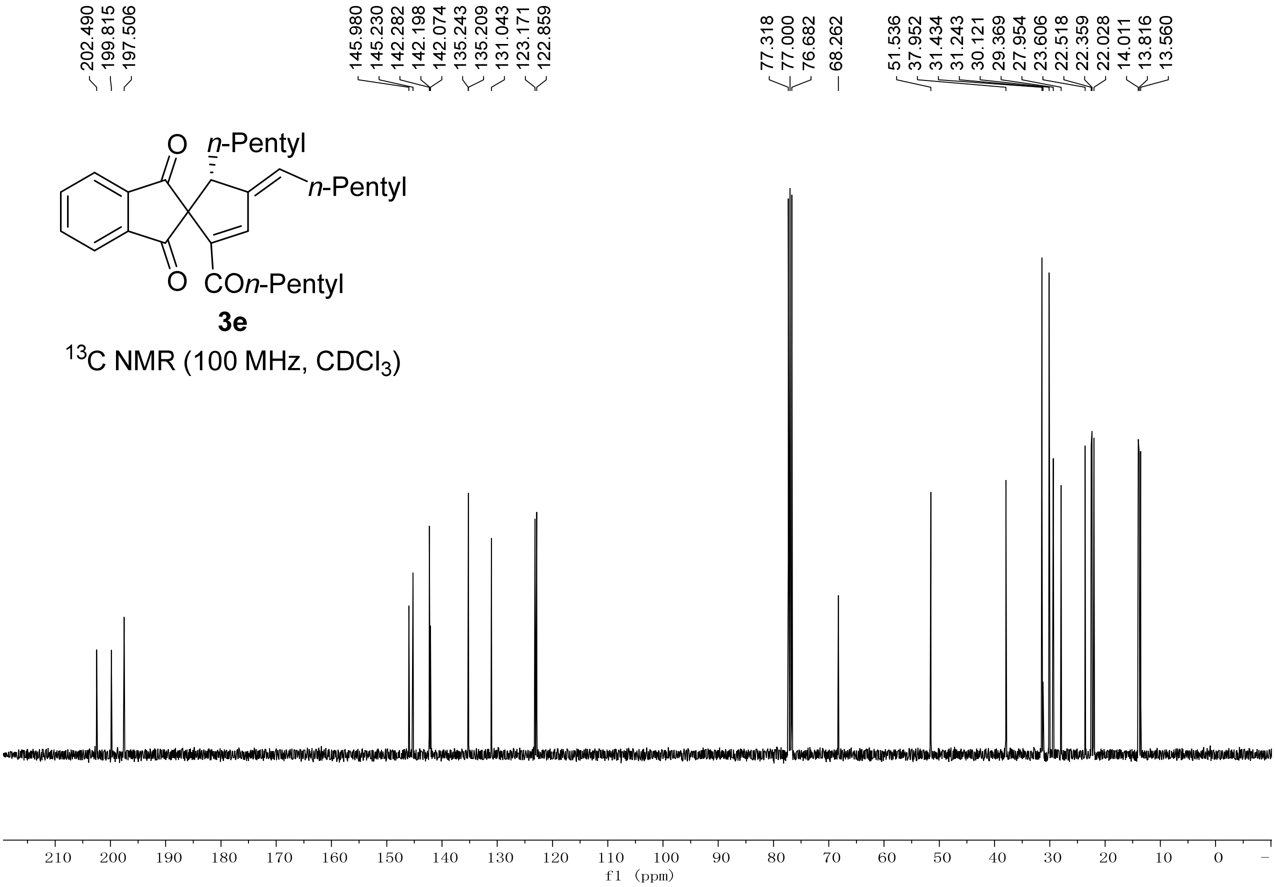

3e

^{13}C NMR (100 MHz, $CDCl_3$)

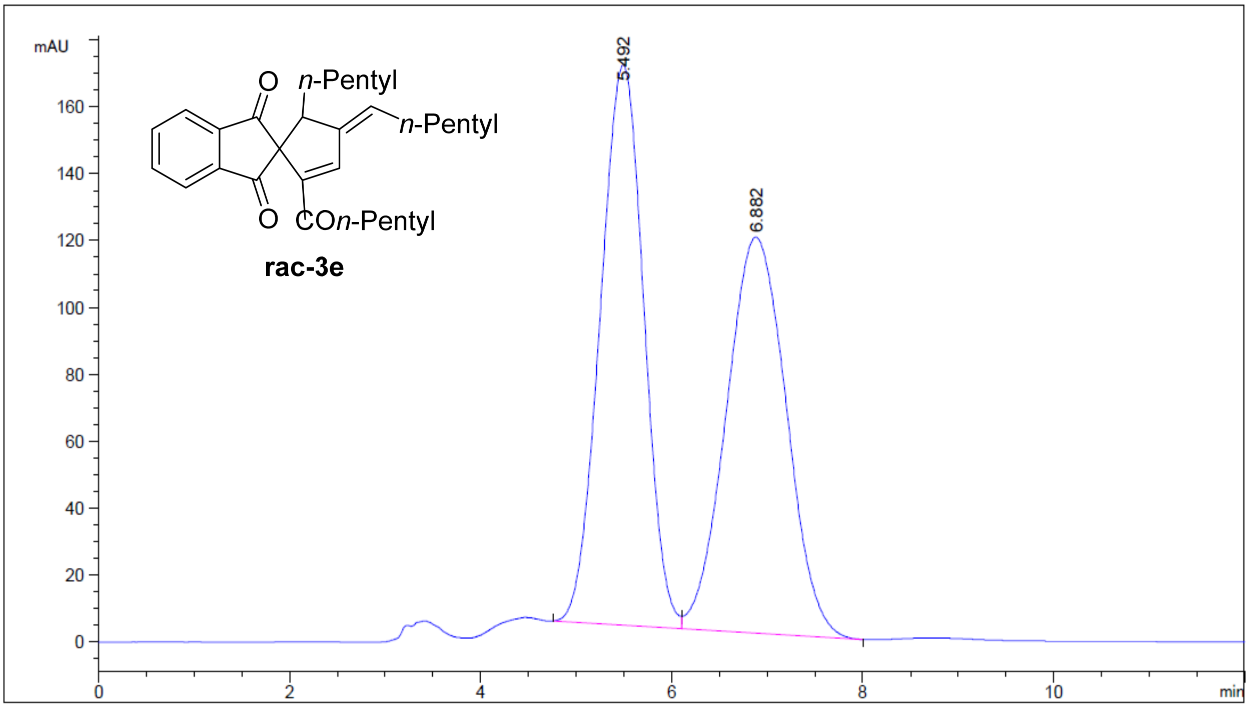

Peak #	RetTime [min]	Type	Width [min]	Area mAU *s	Height [mAU]	Area %
1	5.492	BV	0.5043	5311.76465	167.34761	50.6835
2	6.882	VBA	0.7018	5168.49707	118.32802	49.3165

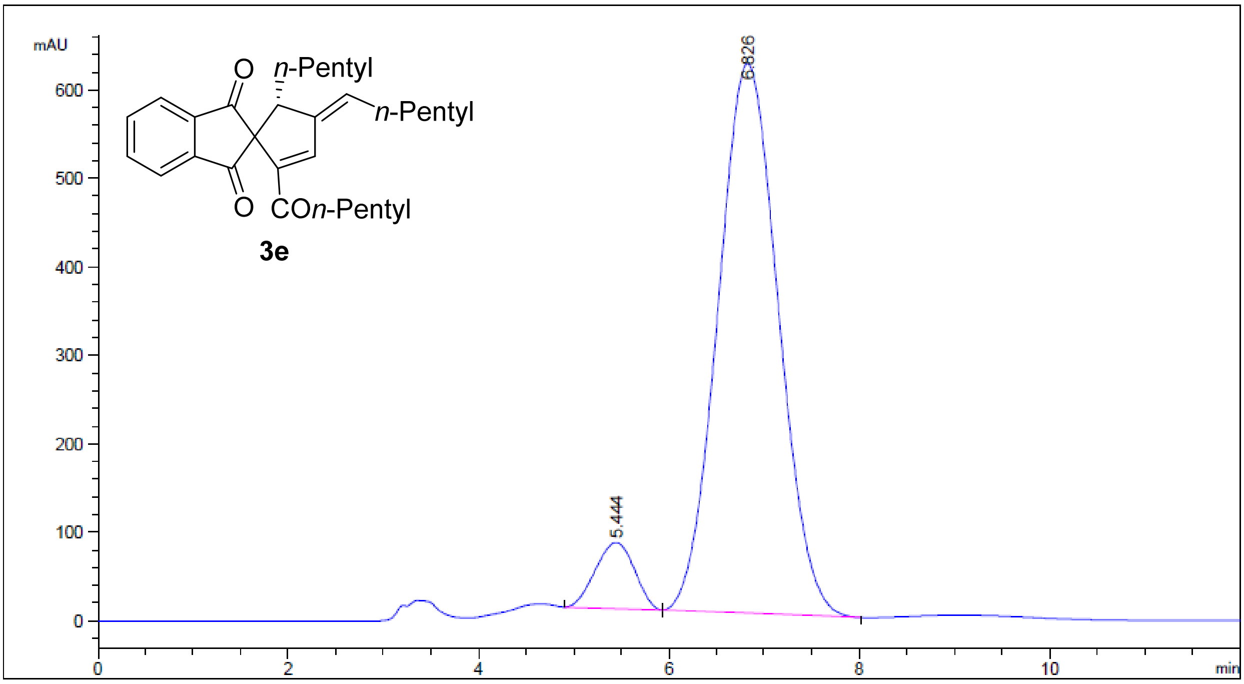

Peak #	RetTime [min]	Type	Width [min]	Area mAU *s	Height [mAU]	Area %
1	5.444	BB	0.4608	2123.64282	74.48172	7.2187
2	6.826	BBA	0.6876	2.72948e4	620.88416	92.7813

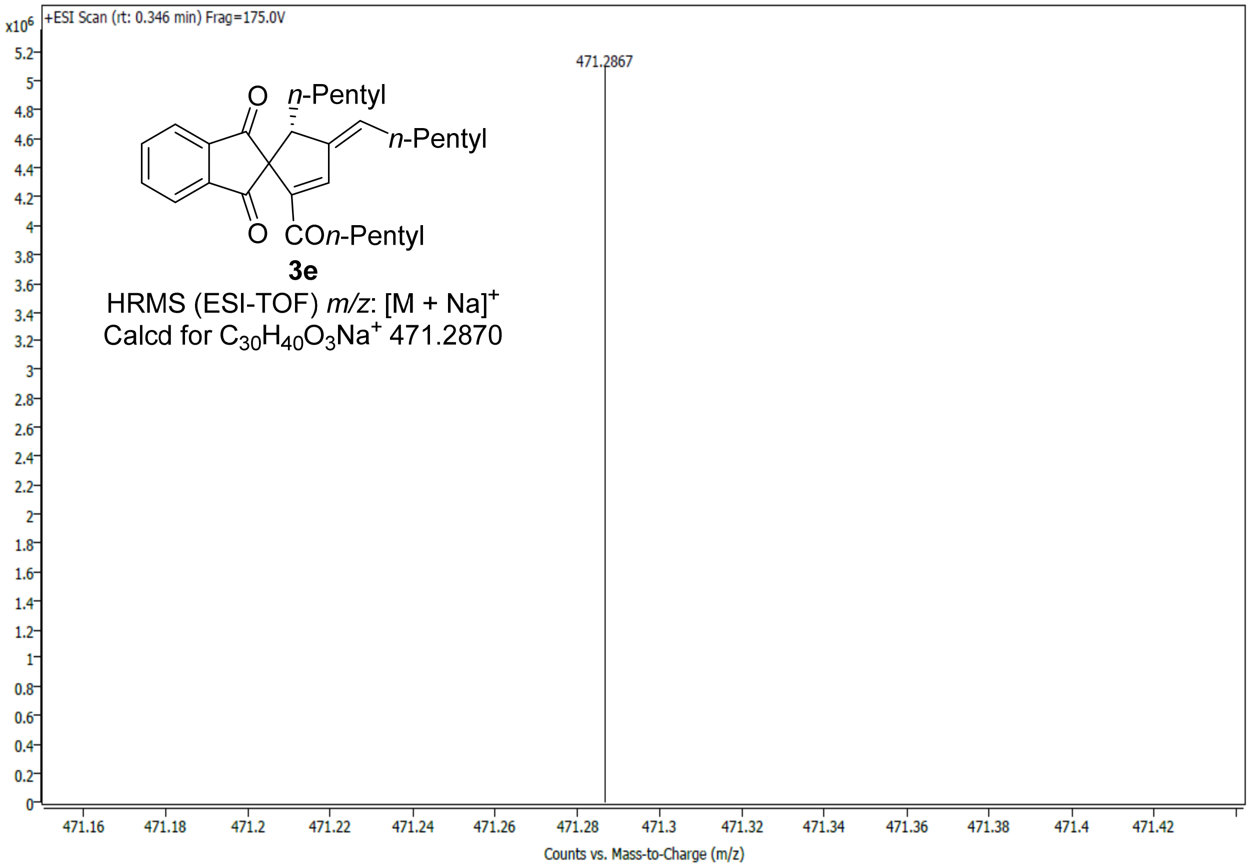

+ESI Scan (rt: 0.346 min) Frag=175.0V
x10^6
471.2867
n-Pentyl
n-Pentyl
COn-Pentyl
3e
HRMS (ESI-TOF) m/z: [M + Na]+
Calcd for C30H40O3Na+ 471.2870
Counts vs. Mass-to-Charge (m/z)

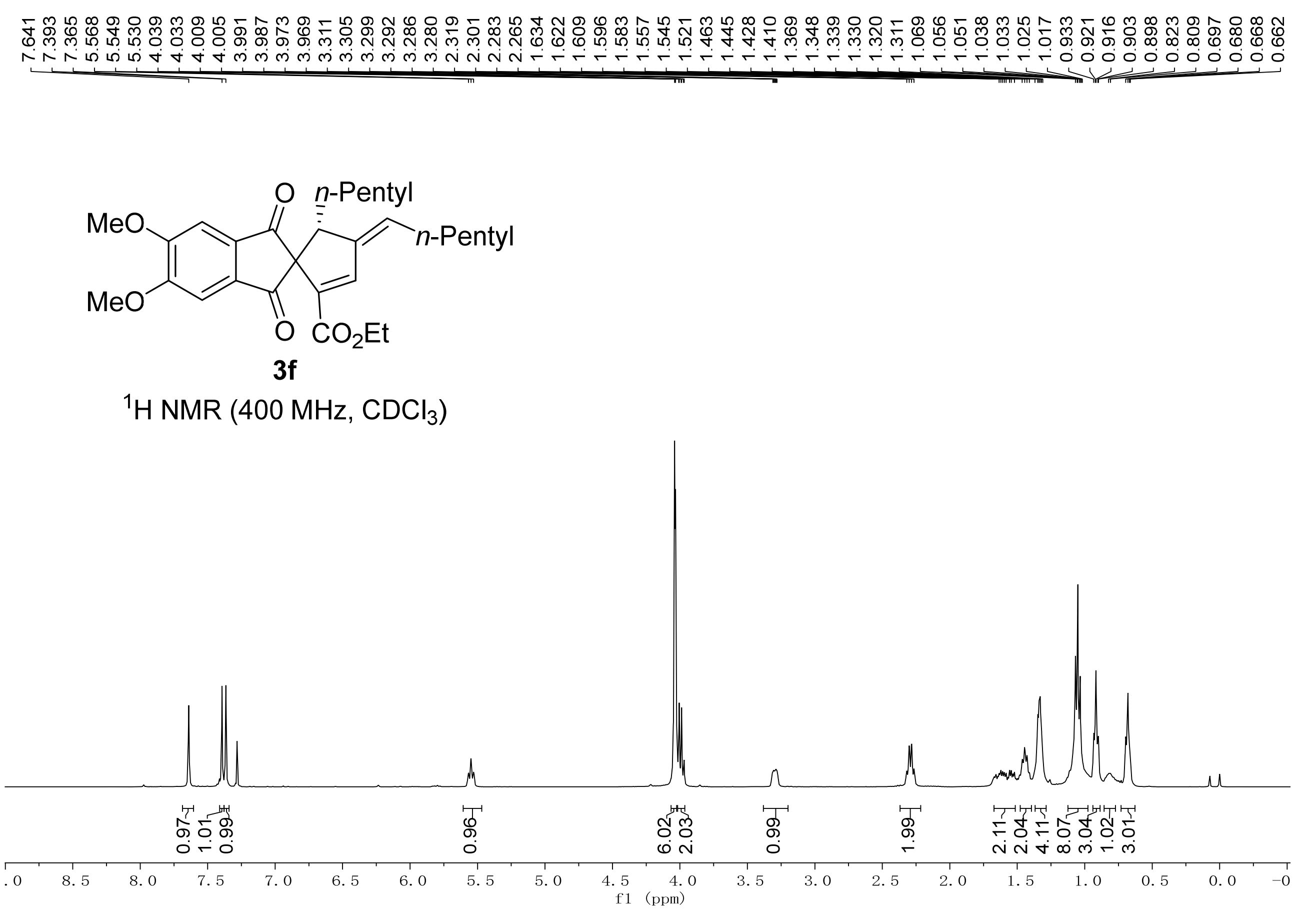
7.641 7.393 7.365 5.568 5.549 5.530 4.039 4.033 4.009 4.005 3.991 3.987 3.973 3.969 3.311 3.305 3.299 3.292 3.286 3.280 2.319 2.301 2.283 2.265 1.634 1.622 1.609 1.596 1.583 1.557 1.545 1.521 1.463 1.445 1.428 1.410 1.369 1.348 1.339 1.330 1.320 1.311 1.069 1.056 1.051 1.038 1.033 1.025 1.017 0.933 0.921 0.916 0.903 0.898 0.823 0.809 0.697 0.680 0.668 0.662
MeO
MeO
O
O
n-Pentyl
n-Pentyl
CO2Et
3f
1H NMR (400 MHz, CDCl3)
0.97 1.01 0.99 0.96 6.02 2.03 0.99 1.99 2.11 2.04 4.11 8.07 3.04 1.02 3.01
f1 (ppm)

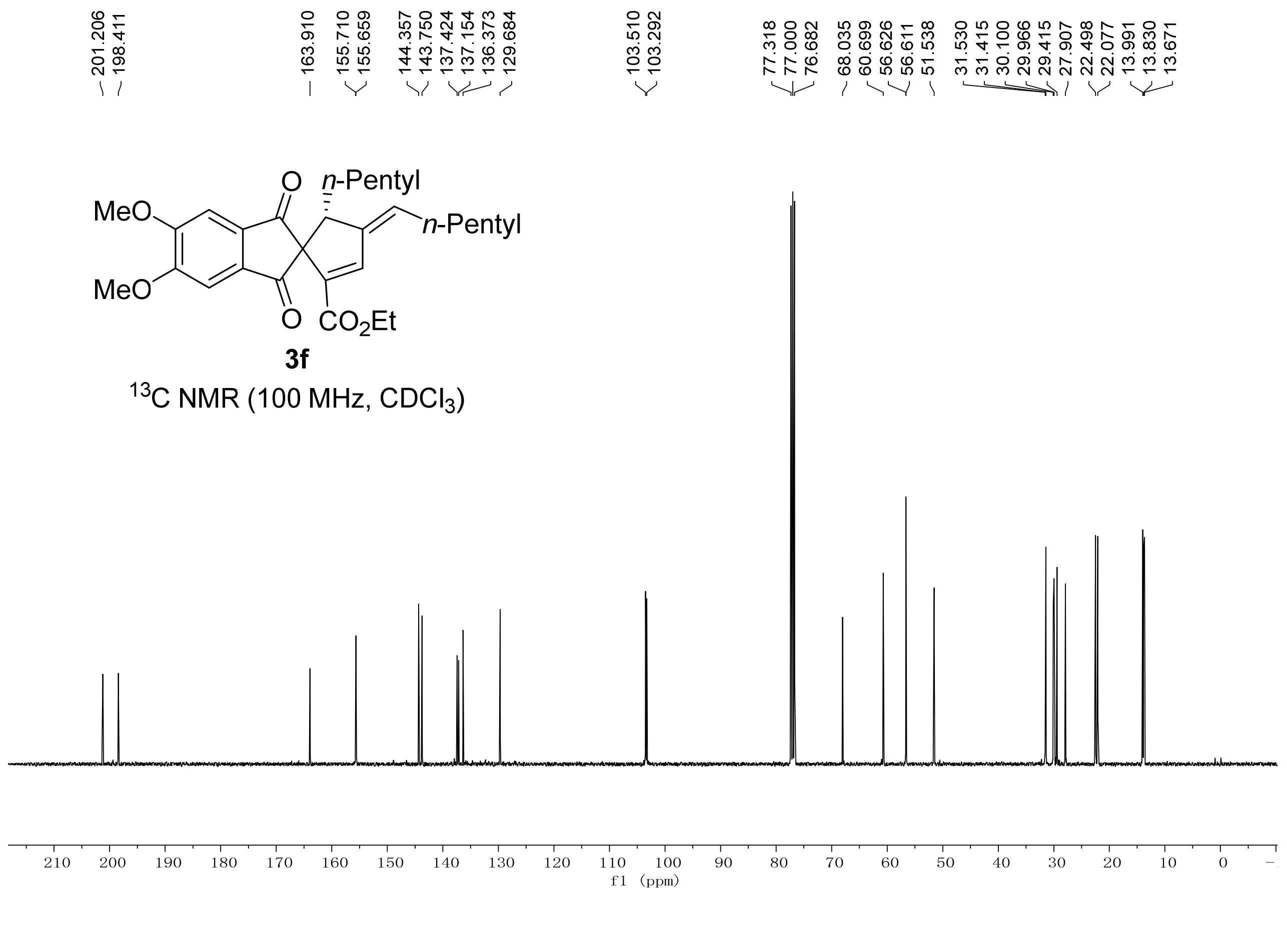
201.206 198.411 163.910 155.710 155.659 144.357 143.750 137.424 137.154 136.373 129.684 103.510 103.292 77.318 77.000 76.682 68.035 60.699 56.626 56.611 51.538 31.530 31.415 30.100 29.966 29.415 27.907 22.498 22.077 13.991 13.830 13.671
MeO
MeO
O
O
n-Pentyl
n-Pentyl
CO2Et
3f
13C NMR (100 MHz, CDCl3)
f1 (ppm)

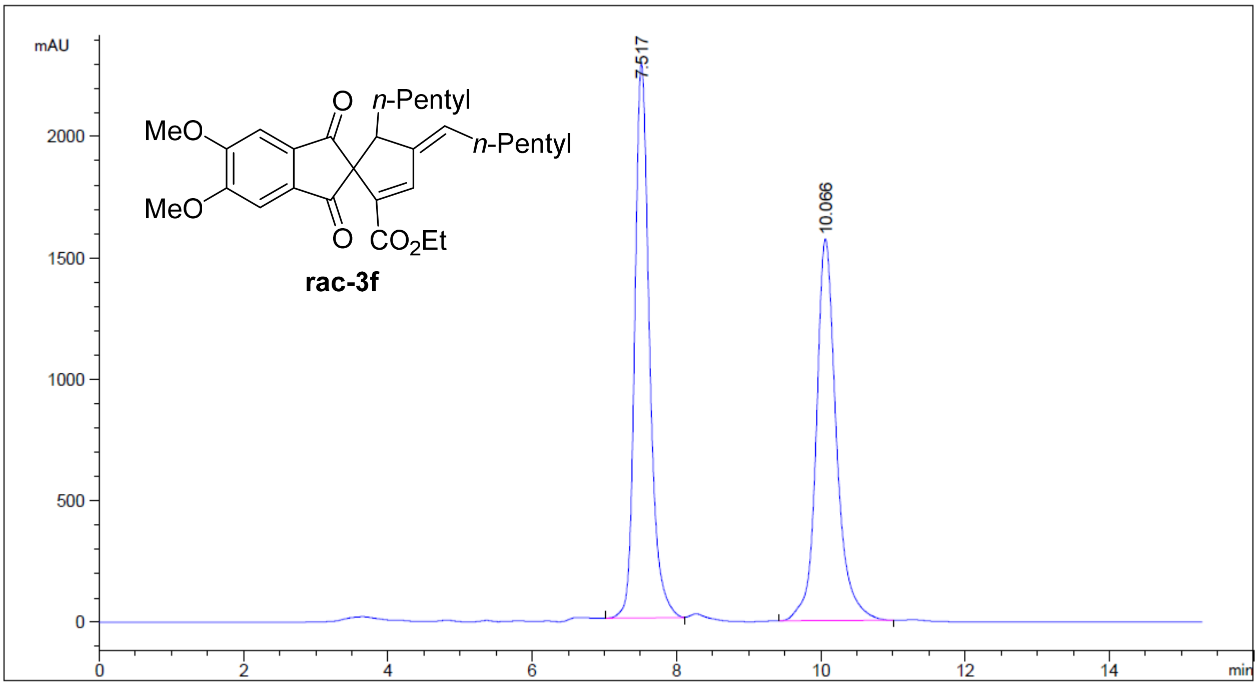

Peak #	RetTime [min]	Type	Width [min]	Area mAU *s	Height [mAU]	Area %
1	7.517	BBA	0.2183	3.23523e4	2275.73804	51.3894
2	10.066	BBA	0.2913	3.06029e4	1571.23364	48.6106

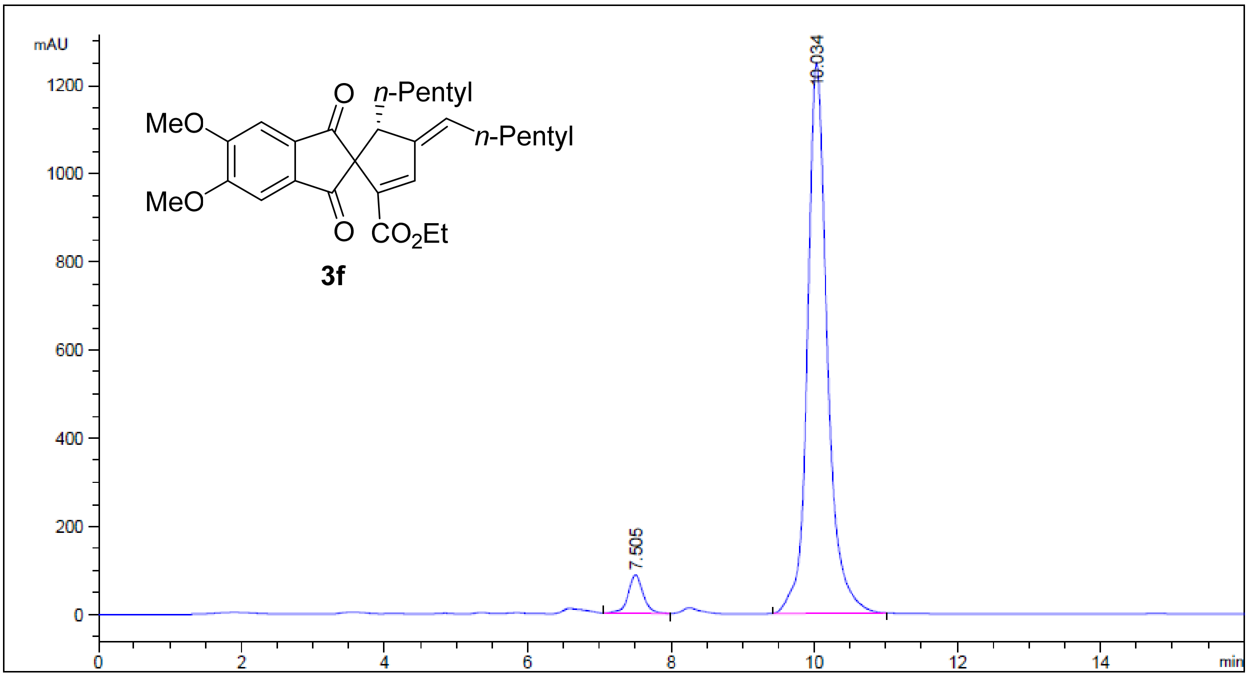

Peak #	RetTime [min]	Type	Width [min]	Area mAU *s	Height [mAU]	Area %
1	7.505	BB	0.2231	1272.33655	86.97343	5.0133
2	10.034	BBA	0.2953	2.41069e4	1247.78516	94.9867

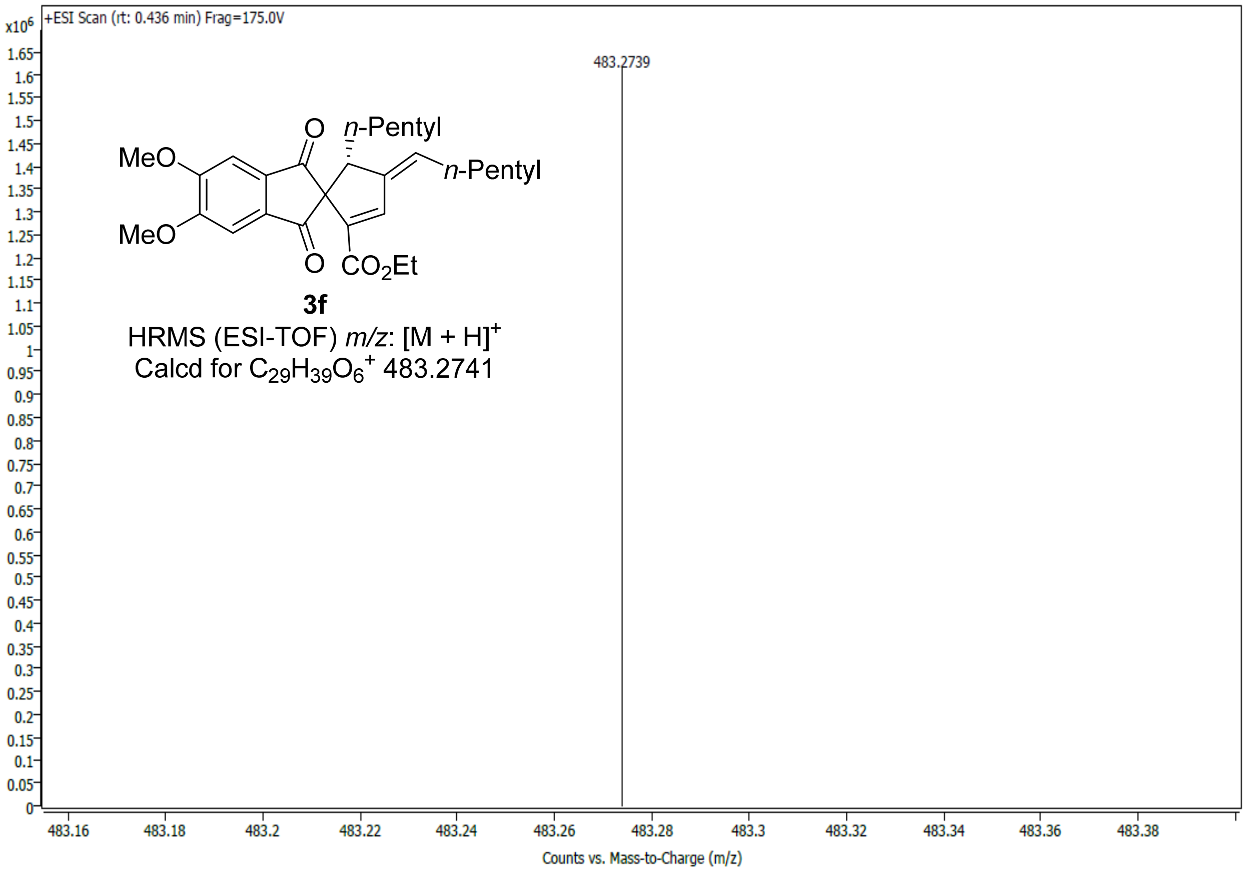

+ESI Scan (rt: 0.436 min) Frag=175.0V
x10^6
483.2739
MeO
MeO
O
O
n-Pentyl
n-Pentyl
CO2Et
3f
HRMS (ESI-TOF) m/z: [M + H]+
Calcd for C29H39O6+ 483.2741
Counts vs. Mass-to-Charge (m/z)

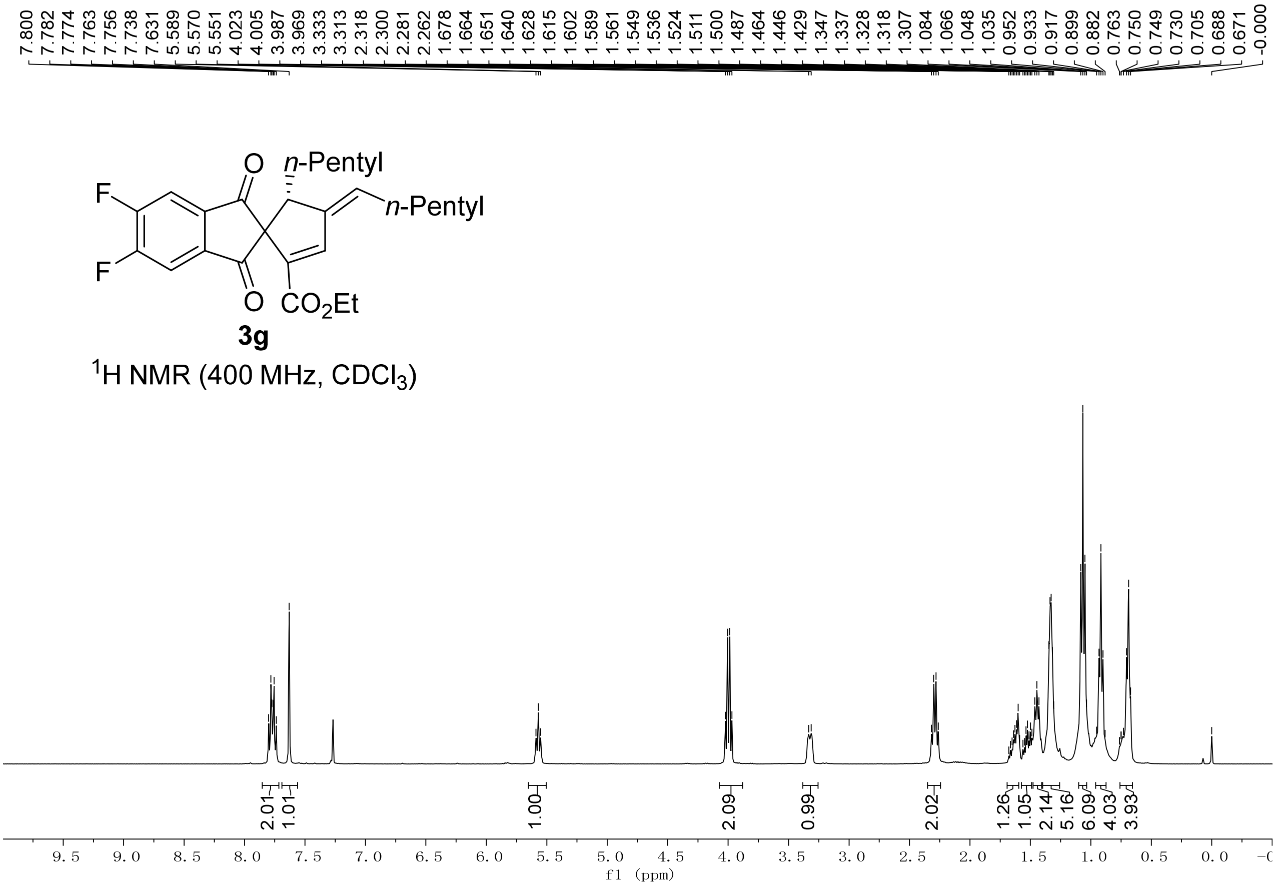
7.800 7.782 7.774 7.763 7.756 7.738 7.631 5.589 5.570 5.551 4.023 4.005 3.987 3.969 3.333 3.313 2.318 2.300 2.281 2.262 1.678 1.664 1.651 1.640 1.628 1.615 1.602 1.589 1.561 1.549 1.536 1.524 1.511 1.500 1.487 1.464 1.446 1.429 1.347 1.337 1.328 1.318 1.307 1.084 1.066 1.048 1.035 0.952 0.933 0.917 0.899 0.882 0.763 0.750 0.749 0.730 0.705 0.688 0.671 -0.000
O
n-Pentyl
F
n-Pentyl
F
O
CO2Et
3g
1H NMR (400 MHz, CDCl3)
2.01 1.01 1.00 2.09 0.99 2.02 1.26 1.05 2.14 5.16 6.09 4.03 3.93
9.5 9.0 8.5 8.0 7.5 7.0 6.5 6.0 5.5 5.0 4.5 4.0 3.5 3.0 2.5 2.0 1.5 1.0 0.5 0.0
f1 (ppm)

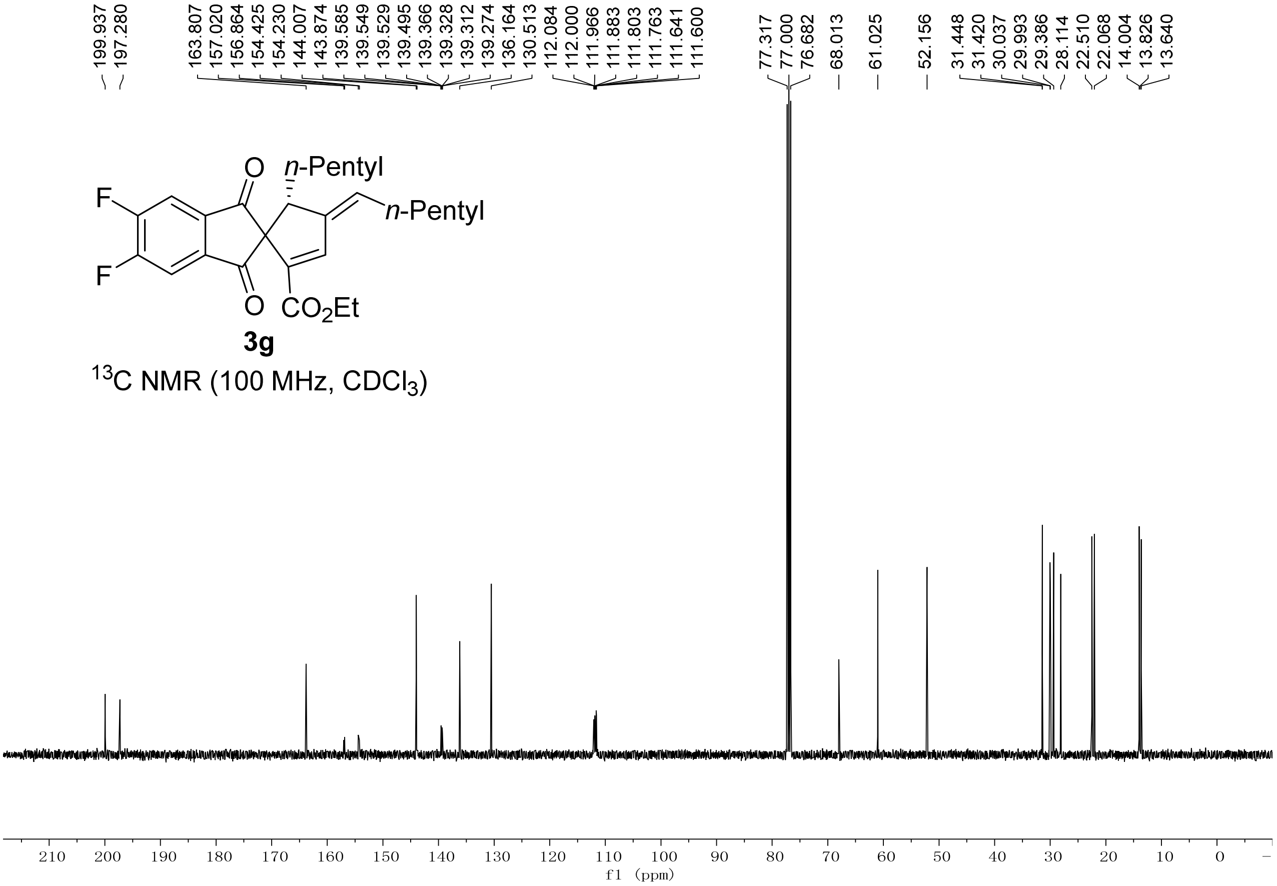
199.937 197.280 163.807 157.020 156.864 154.425 154.230 144.007 143.874 139.585 139.549 139.529 139.495 139.366 139.328 139.312 139.274 136.164 130.513 112.084 112.000 111.966 111.883 111.803 111.763 111.641 111.600 77.317 77.000 76.682 68.013 61.025 52.156 31.448 31.420 30.037 29.993 29.386 28.114 22.510 22.068 14.004 13.826 13.640
O
n-Pentyl
F
n-Pentyl
F
O
CO2Et
3g
13C NMR (100 MHz, CDCl3)
210 200 190 180 170 160 150 140 130 120 110 100 90 80 70 60 50 40 30 20 10 0
f1 (ppm)

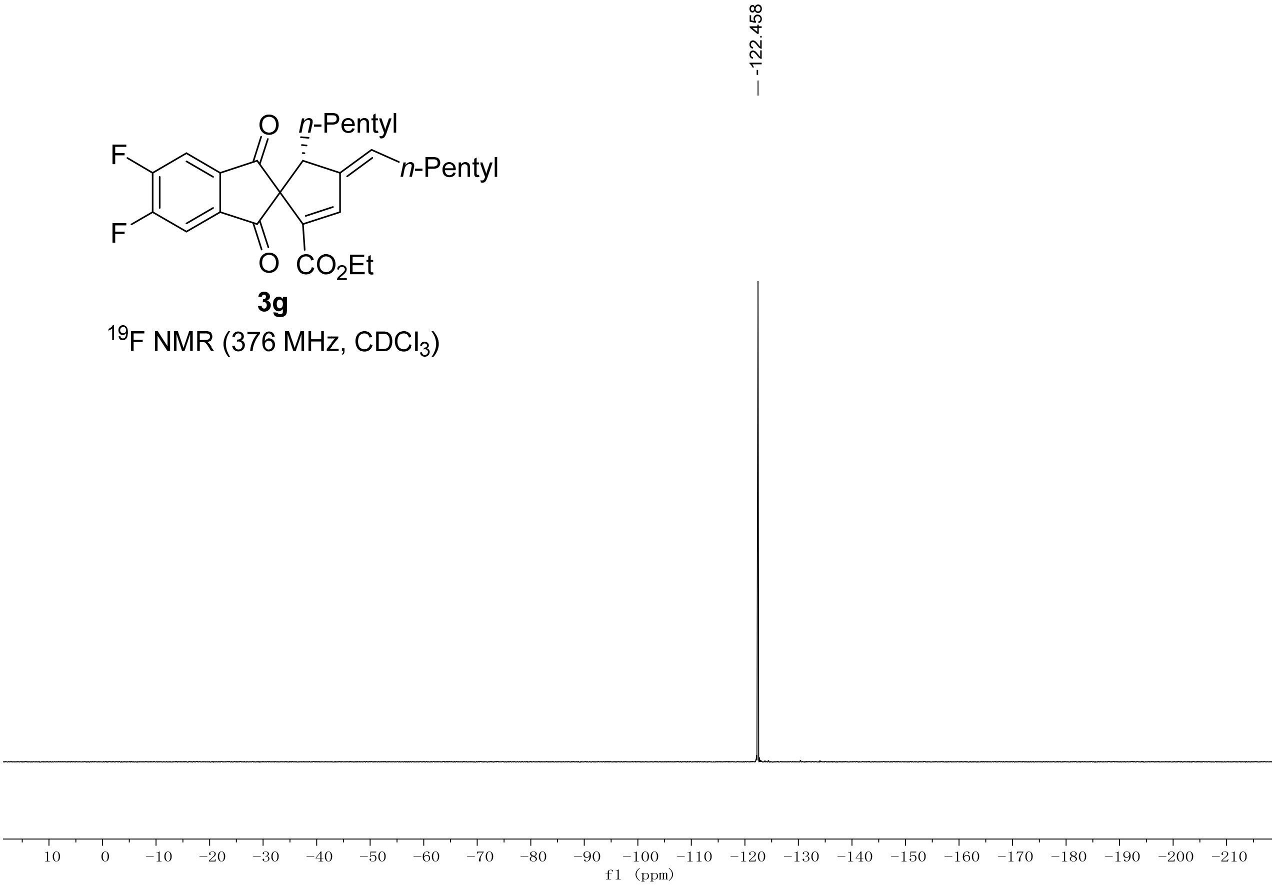
-122.458
O
n-Pentyl
F
n-Pentyl
F
O
CO2Et
3g
19F NMR (376 MHz, CDCl3)
10 0 −10 −20 −30 −40 −50 −60 −70 −80 −90 −100 −110 −120 −130 −140 −150 −160 −170 −180 −190 −200 −210
f1 (ppm)

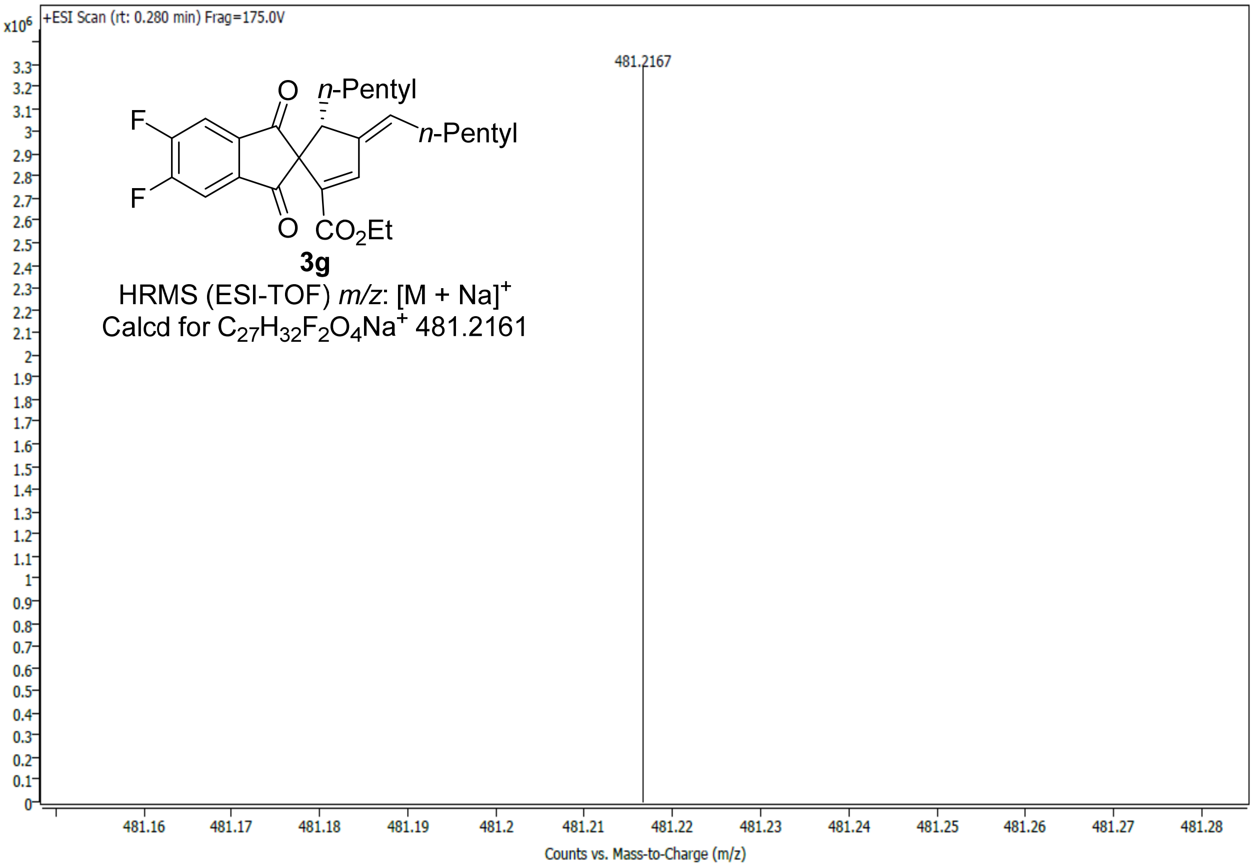
+ESI Scan (rt: 0.280 min) Frag=175.0V
x10^6
481.2167
3g
HRMS (ESI-TOF) m/z: [M + Na]+
Calcd for C27H32F2O4Na+ 481.2161
481.16 481.17 481.18 481.19 481.2 481.21 481.22 481.23 481.24 481.25 481.26 481.27 481.28
Counts vs. Mass-to-Charge (m/z)

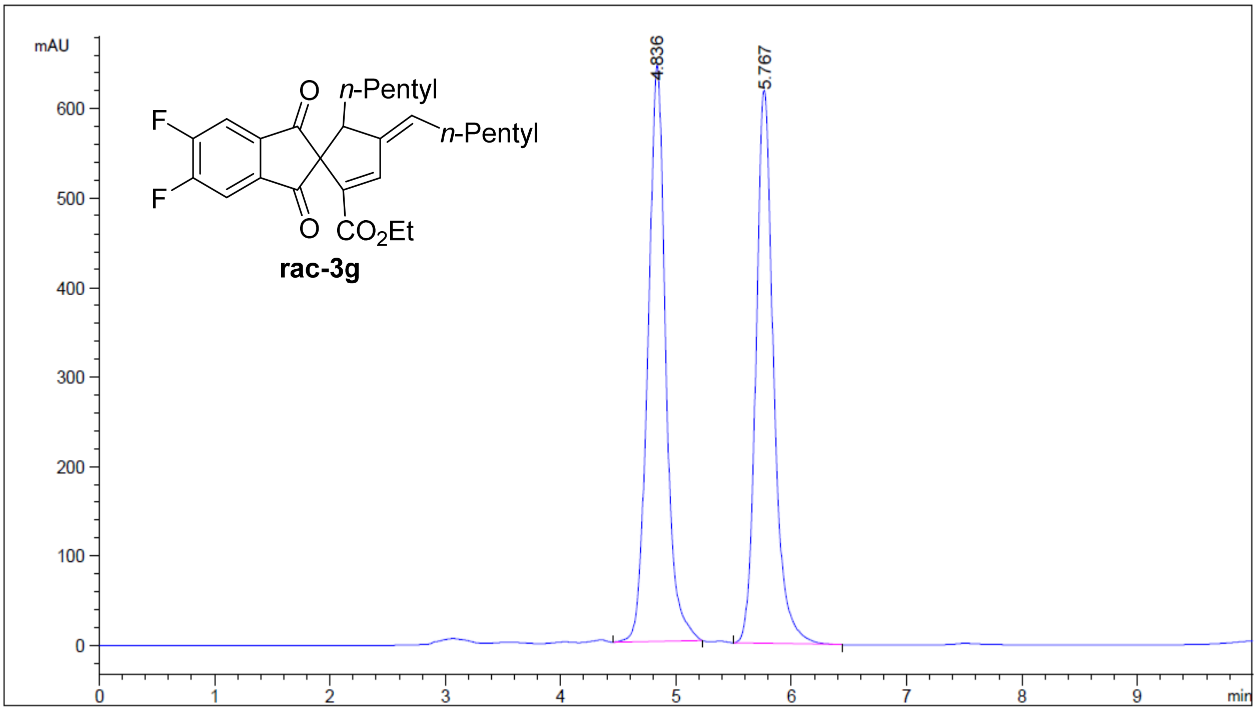

Peak #	RetTime [min]	Type	Width [min]	Area mAU *s	Height [mAU]	Area %
1	4.836	BBA	0.1661	6903.54785	642.68915	51.2263
2	5.767	BBA	0.1717	6573.02832	613.09570	48.7737

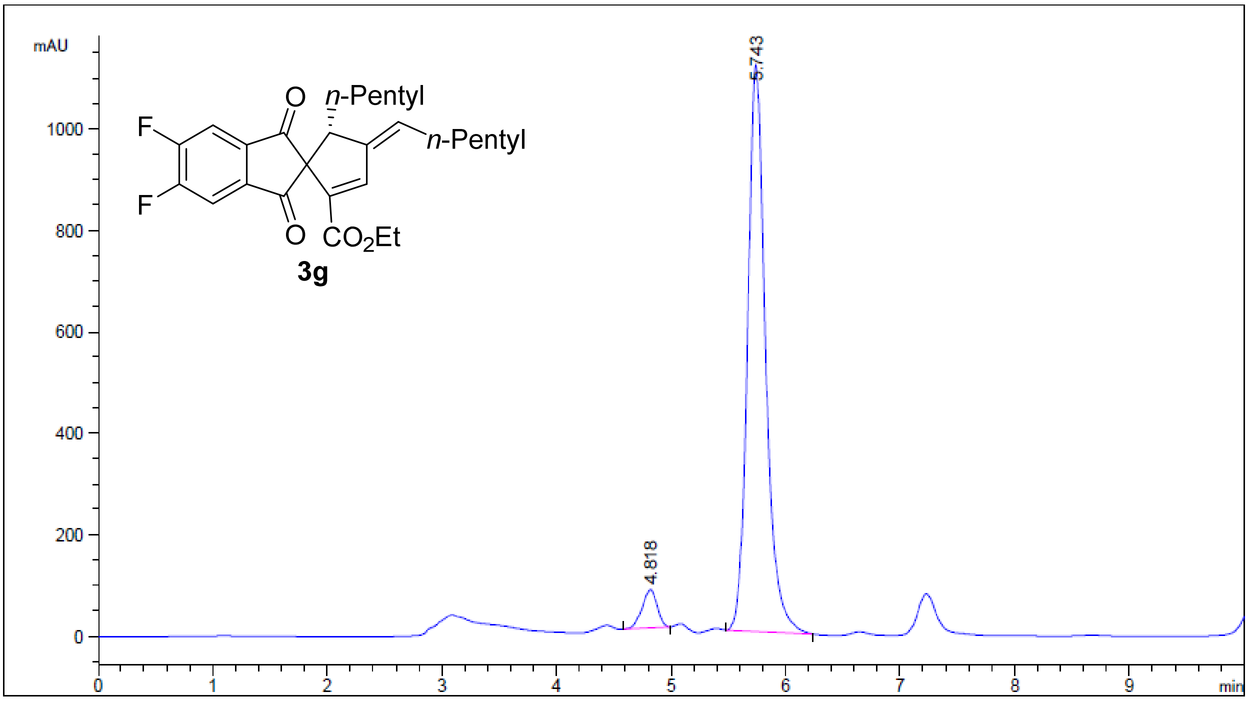

Peak #	RetTime [min]	Type	Width [min]	Area mAU *s	Height [mAU]	Area %
1	4.818	BBA	0.1475	686.71259	75.04794	5.3370
2	5.743	BBA	0.1741	1.21803e4	1114.06628	94.6630

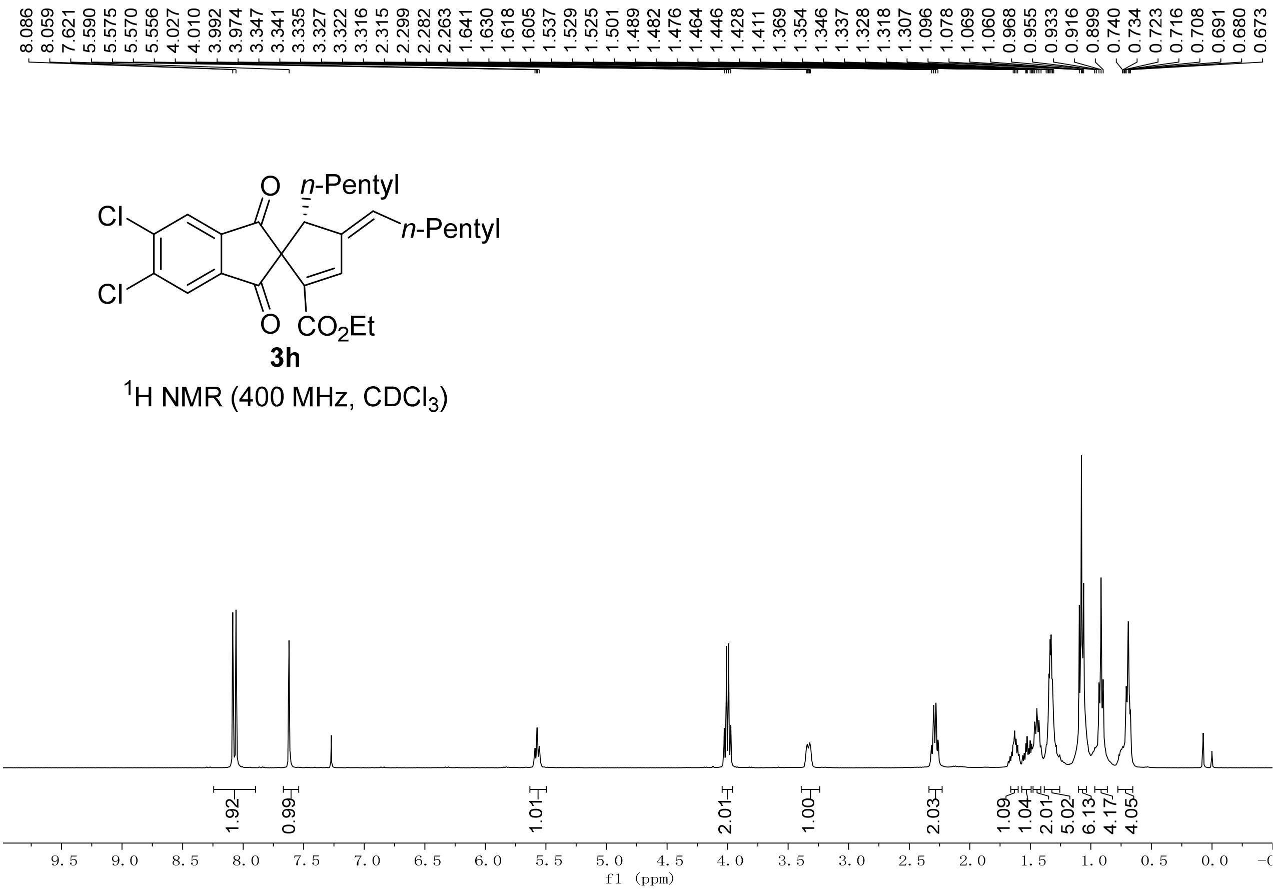
8.086 8.059 7.621 5.590 5.575 5.570 5.556 4.027 4.010 3.992 3.974 3.347 3.341 3.335 3.327 3.322 3.316 2.315 2.299 2.282 2.263 1.641 1.630 1.618 1.605 1.537 1.529 1.525 1.501 1.489 1.482 1.476 1.464 1.446 1.428 1.411 1.369 1.354 1.346 1.337 1.328 1.318 1.307 1.096 1.078 1.069 1.060 0.968 0.955 0.933 0.916 0.899 0.740 0.734 0.723 0.716 0.708 0.691 0.680 0.673
Cl
Cl
O
O
n-Pentyl
n-Pentyl
CO2Et
3h
1H NMR (400 MHz, CDCl3)
1.92 0.99 1.01 2.01 1.00 2.03 1.09 1.04 2.01 5.02 6.13 4.17 4.05
f1 (ppm)

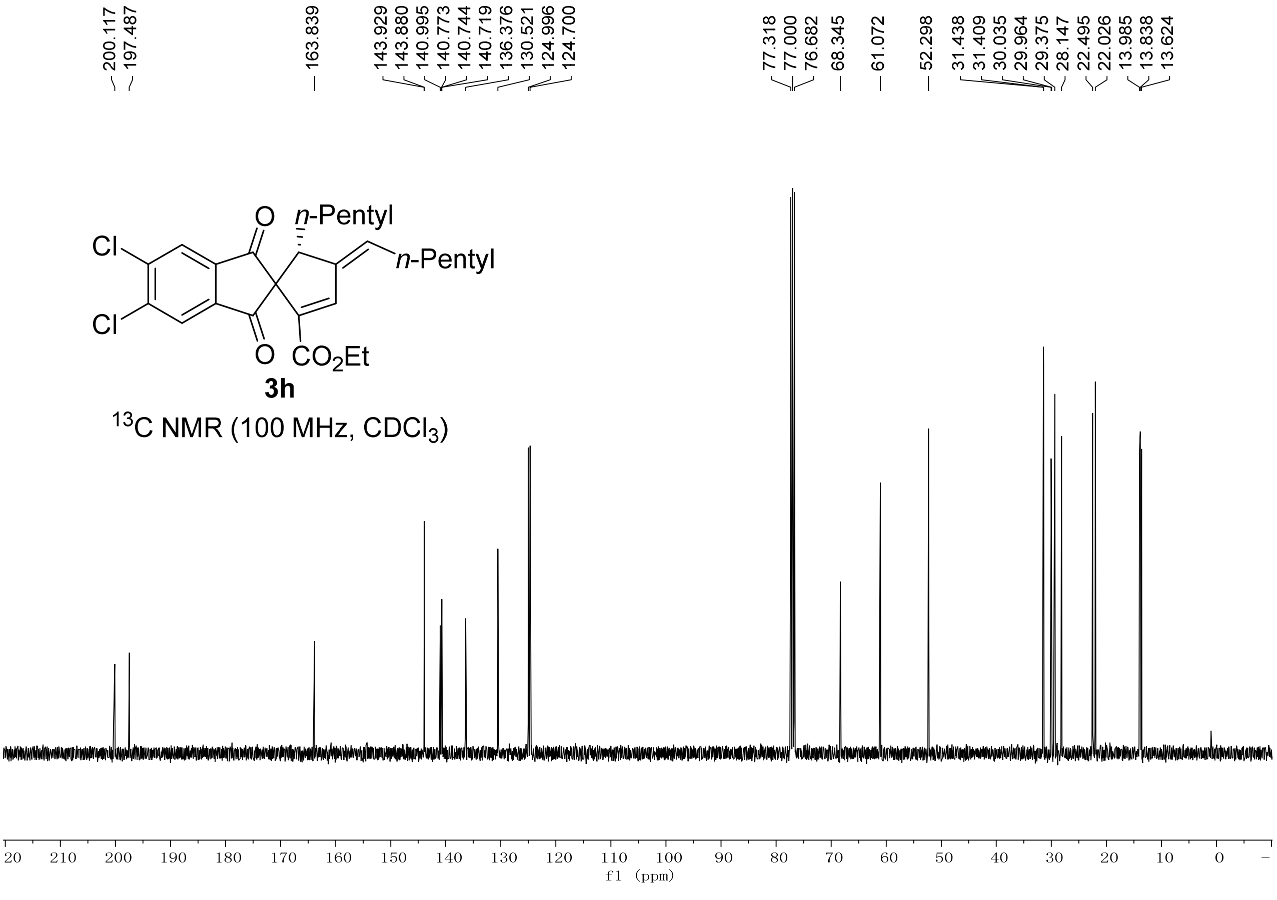
200.117 197.487 163.839 143.929 143.880 140.995 140.773 140.744 140.719 136.376 130.521 124.996 124.700 77.318 77.000 76.682 68.345 61.072 52.298 31.438 31.409 30.035 29.964 29.375 28.147 22.495 22.026 13.985 13.838 13.624
Cl
Cl
O
O
n-Pentyl
n-Pentyl
CO2Et
3h
13C NMR (100 MHz, CDCl3)
f1 (ppm)

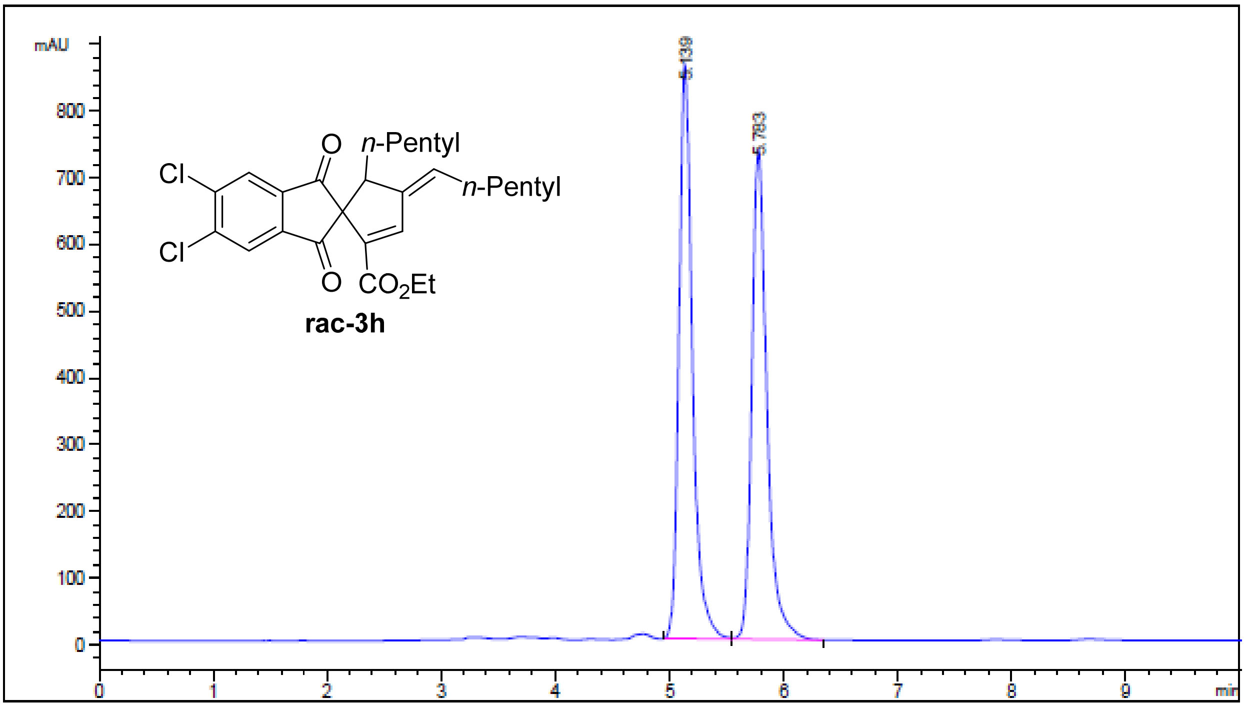

Peak #	RetTime [min]	Type	Width [min]	Area mAU *s	Height [mAU]	Area %
1	5.139	BV	0.1310	7047.24951	856.20929	51.1244
2	5.783	VBA	0.1447	6737.27295	716.08264	48.8756

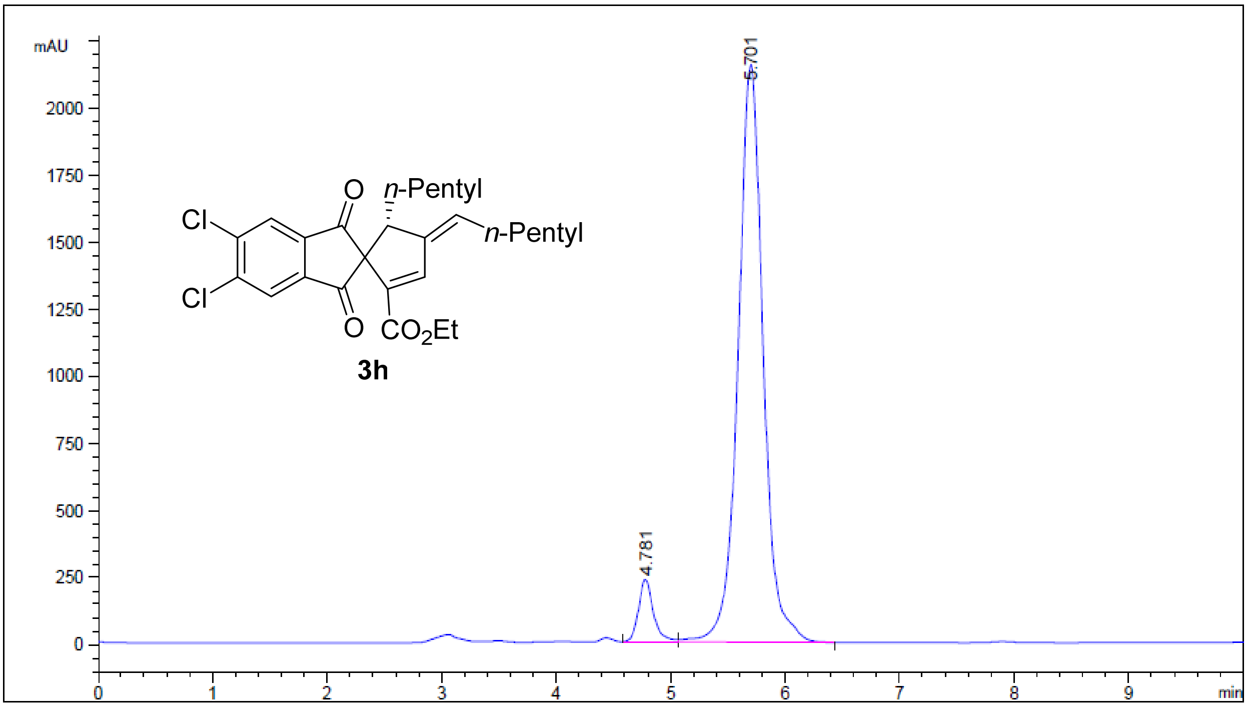

Peak #	RetTime [min]	Type	Width [min]	Area mAU *s	Height [mAU]	Area %
1	4.781	BV	0.1467	2089.36646	230.10304	6.0064
2	5.701	VBA	0.2360	3.26961e4	2146.06689	93.9936

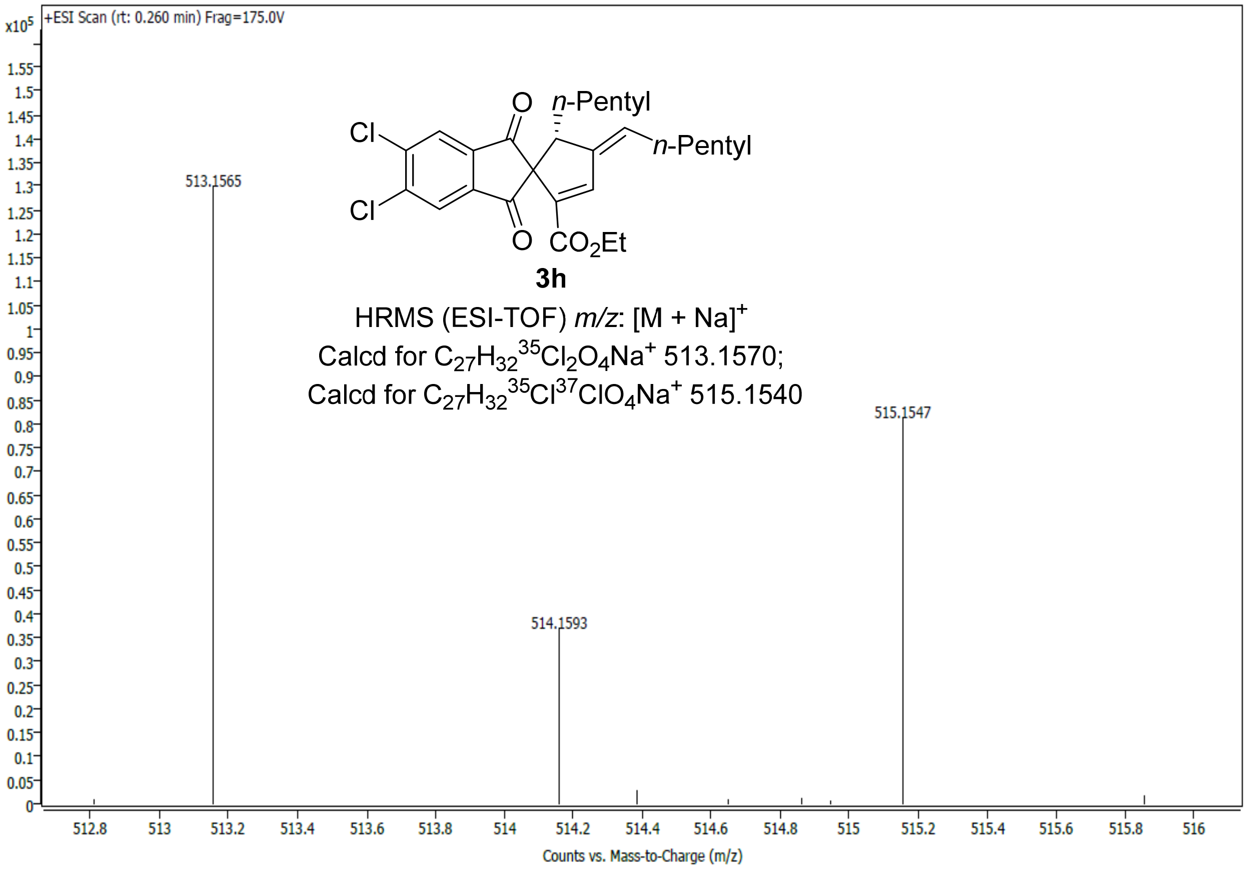

+ESI Scan (rt: 0.260 min) Frag=175.0V
x10^5
513.1565
514.1593
515.1547
Counts vs. Mass-to-Charge (m/z)
3h
HRMS (ESI-TOF) m/z: [M + Na]+
Calcd for C27H32 35Cl2O4Na+ 513.1570;
Calcd for C27H32 35Cl37ClO4Na+ 515.1540

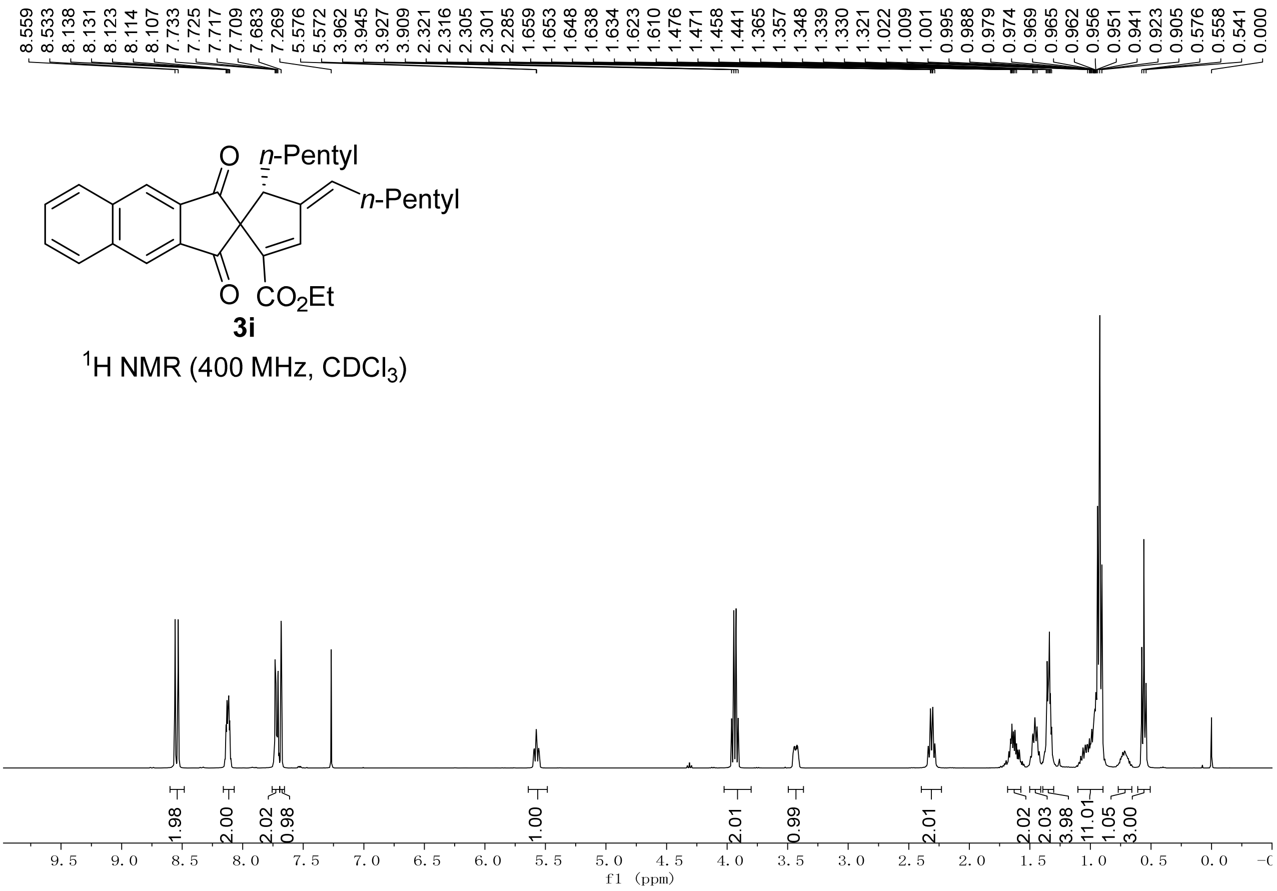
8.559
8.533
8.138
8.131
8.123
8.114
8.107
7.733
7.725
7.717
7.709
7.683
7.269
5.576
5.572
3.962
3.945
3.927
3.909
2.321
2.316
2.305
2.301
2.285
1.659
1.653
1.648
1.638
1.634
1.623
1.610
1.476
1.471
1.458
1.441
1.365
1.357
1.348
1.339
1.330
1.321
1.022
1.009
1.001
0.995
0.988
0.979
0.974
0.969
0.965
0.962
0.956
0.951
0.941
0.923
0.905
0.576
0.558
0.541
0.000
O
n-Pentyl
n-Pentyl
O
CO2Et
3i
1H NMR (400 MHz, CDCl3)
1.98
2.00
2.02
0.98
1.00
2.01
0.99
2.01
2.02
2.03
3.98
11.01
1.05
3.00
f1 (ppm)

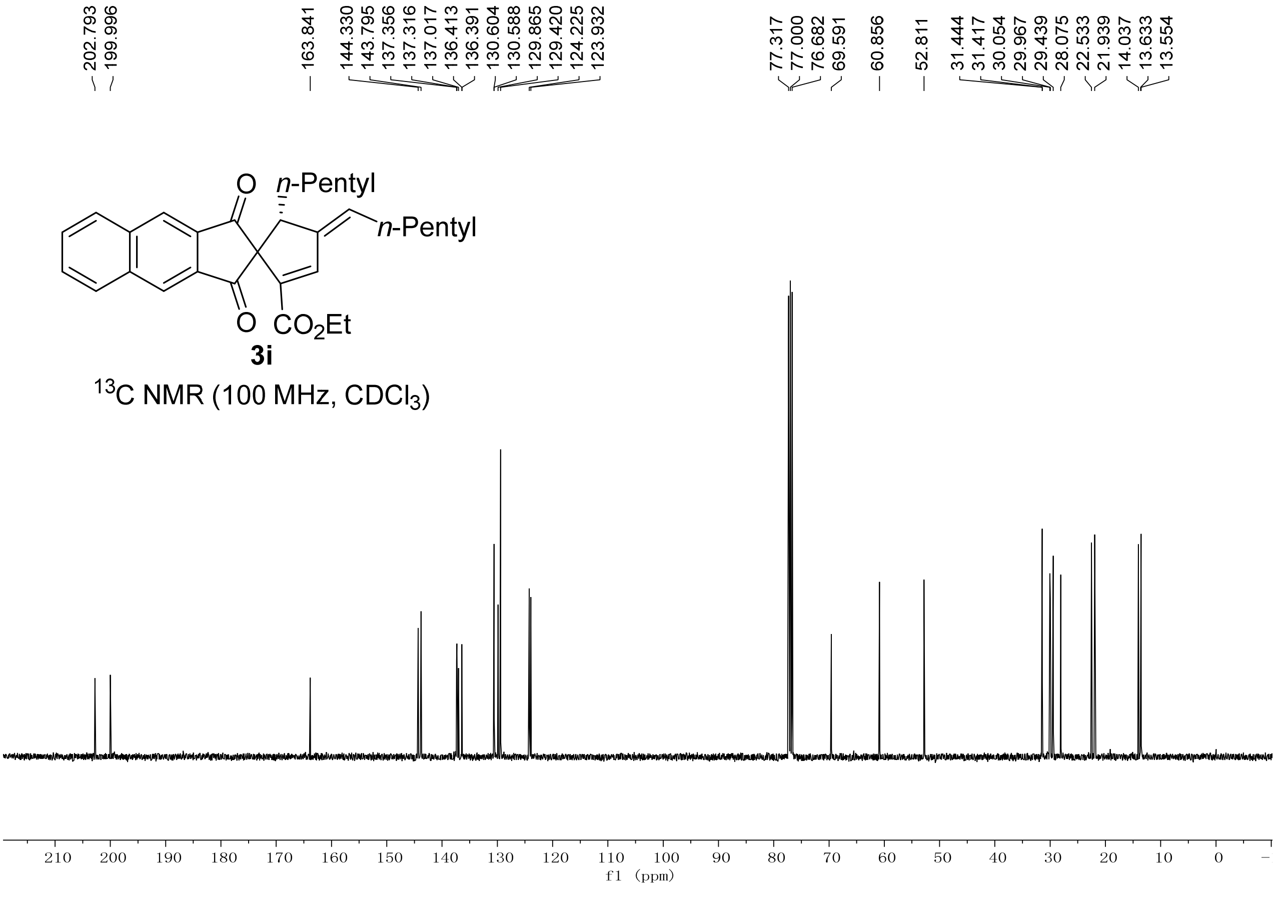
202.793
199.996
163.841
144.330
143.795
137.356
137.316
137.017
136.413
136.391
130.604
130.588
129.865
129.420
124.225
123.932
77.317
77.000
76.682
69.591
60.856
52.811
31.444
31.417
30.054
29.967
29.439
28.075
22.533
21.939
14.037
13.633
13.554
O
n-Pentyl
n-Pentyl
O
CO2Et
3i
13C NMR (100 MHz, CDCl3)
f1 (ppm)

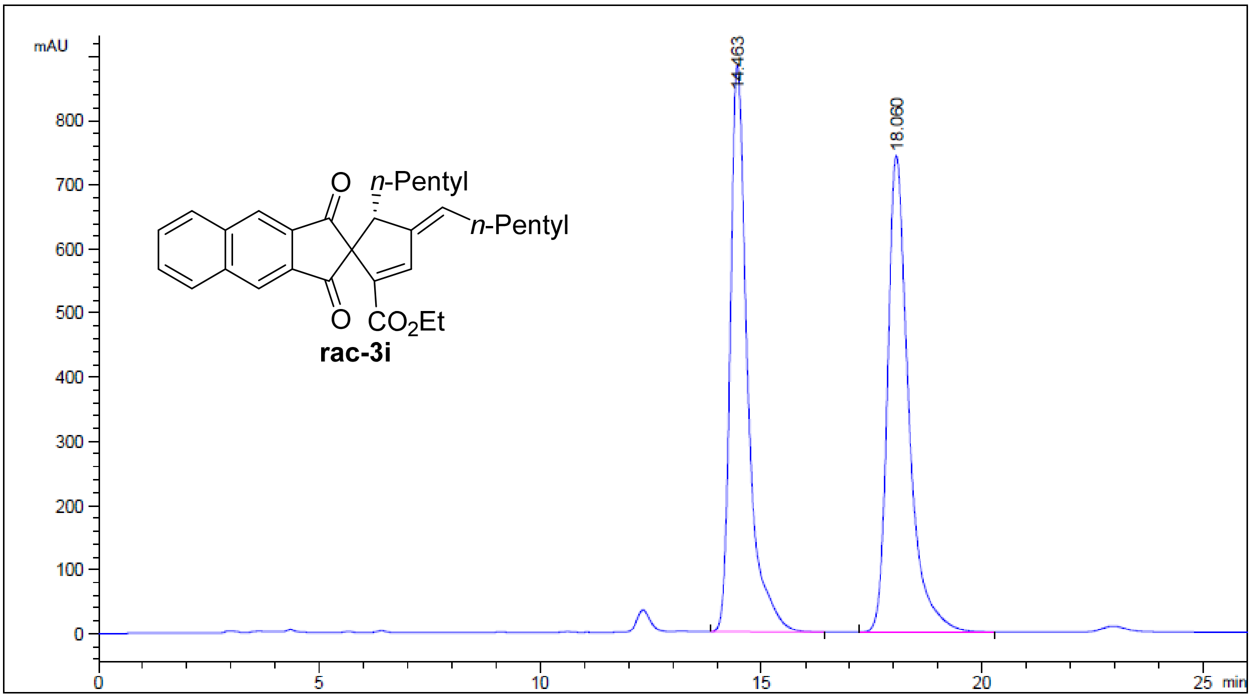

Peak #	RetTime [min]	Type	Width [min]	Area mAU *s	Height [mAU]	Area %
1	14.463	BB	0.4070	2.41184e4	884.35760	49.4644
2	18.060	BB	0.4976	2.46407e4	742.85956	50.5356

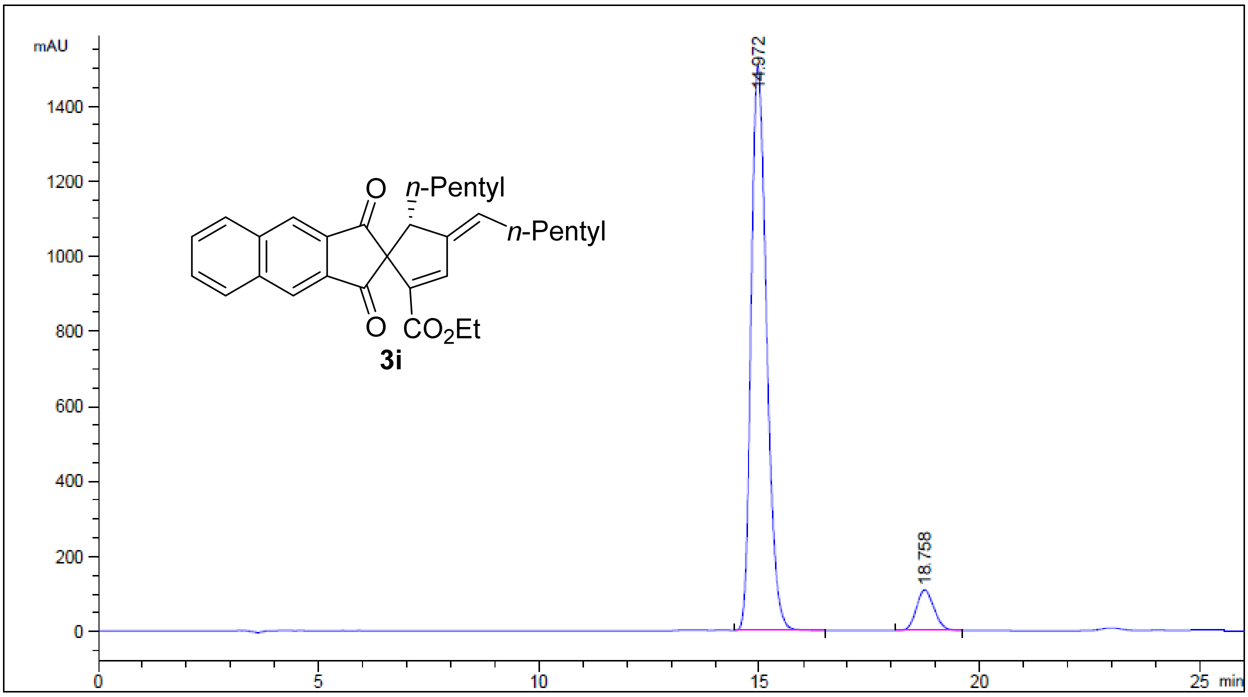

Peak #	RetTime [min]	Type	Width [min]	Area mAU *s	Height [mAU]	Area %
1	14.972	BB	0.3808	3.67958e4	1507.84827	92.2347
2	18.758	BB	0.4396	3097.88525	108.89393	7.7653

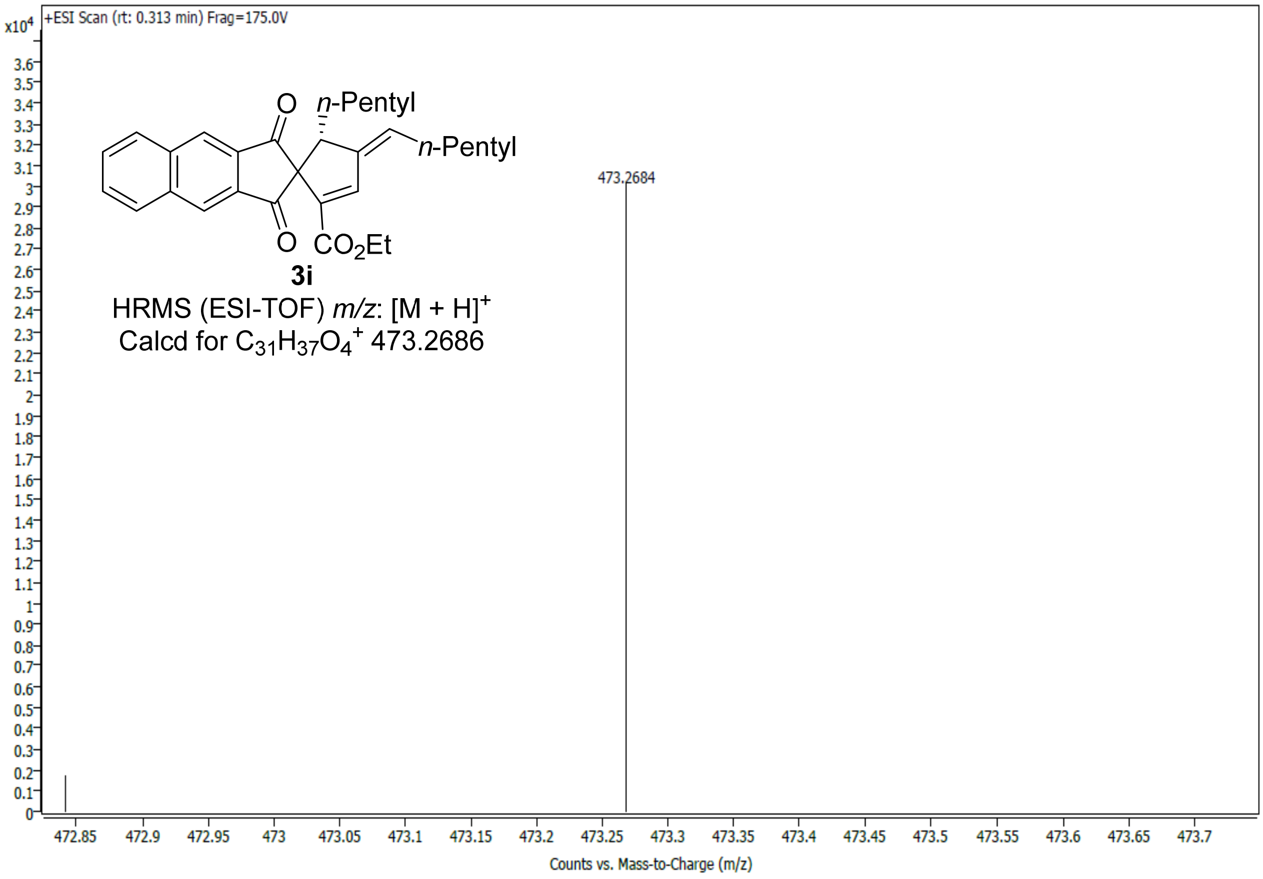
+ESI Scan (rt: 0.313 min) Frag=175.0V
x10^4
473.2684
O
n-Pentyl
n-Pentyl
O
CO2Et
3i
HRMS (ESI-TOF) m/z: [M + H]+
Calcd for C31H37O4+ 473.2686
Counts vs. Mass-to-Charge (m/z)

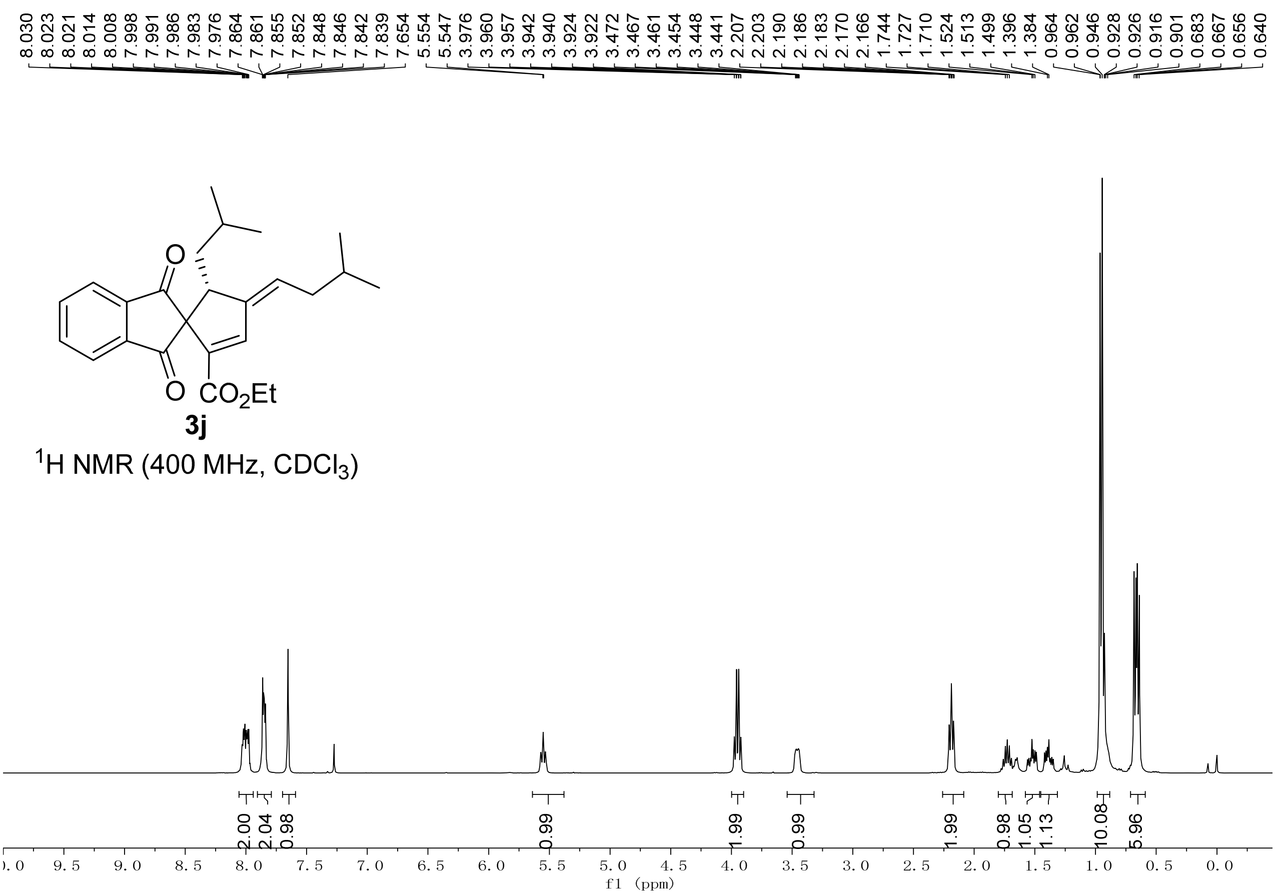

3j

^1H NMR (400 MHz, $CDCl_3$)

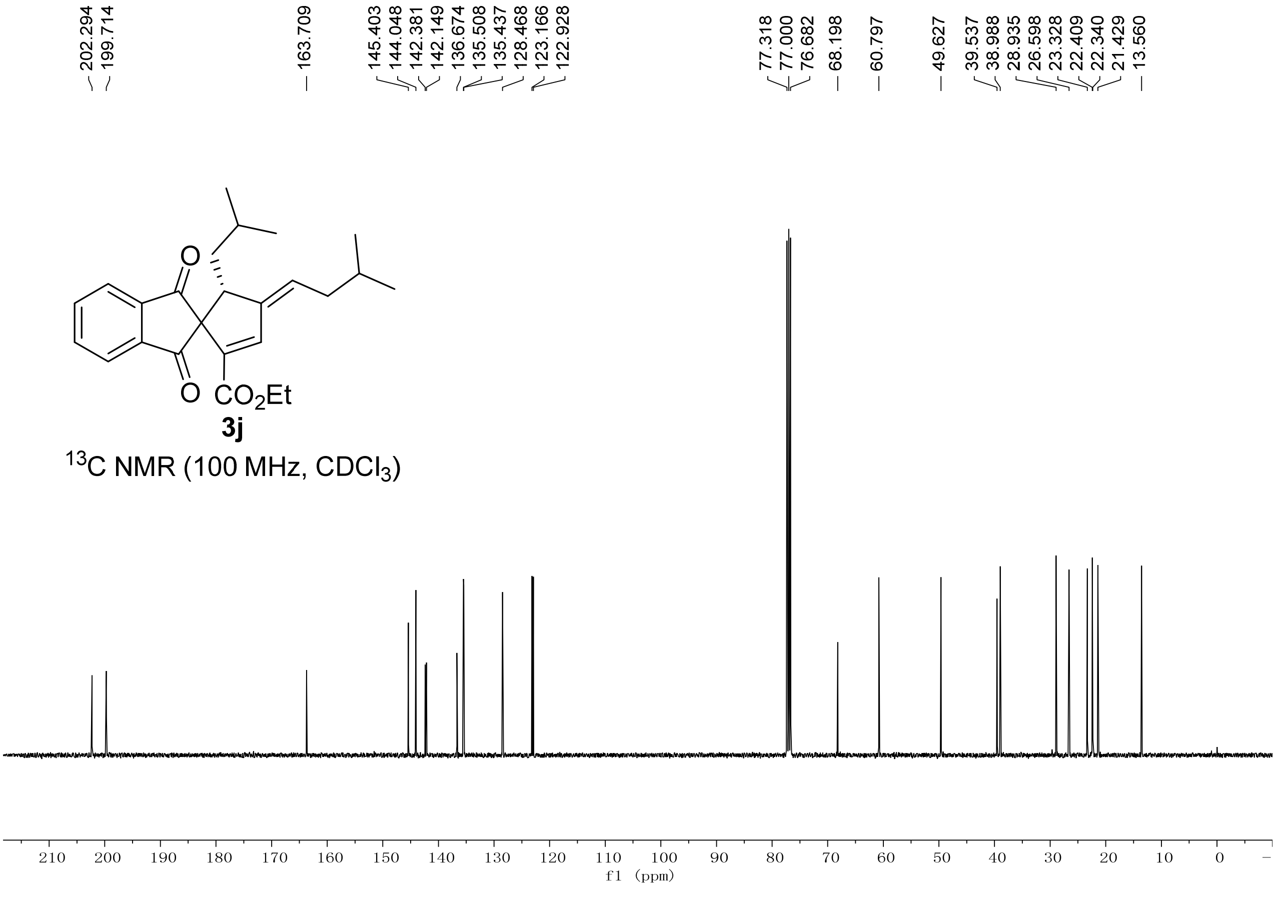

3j

^{13}C NMR (100 MHz, $CDCl_3$)

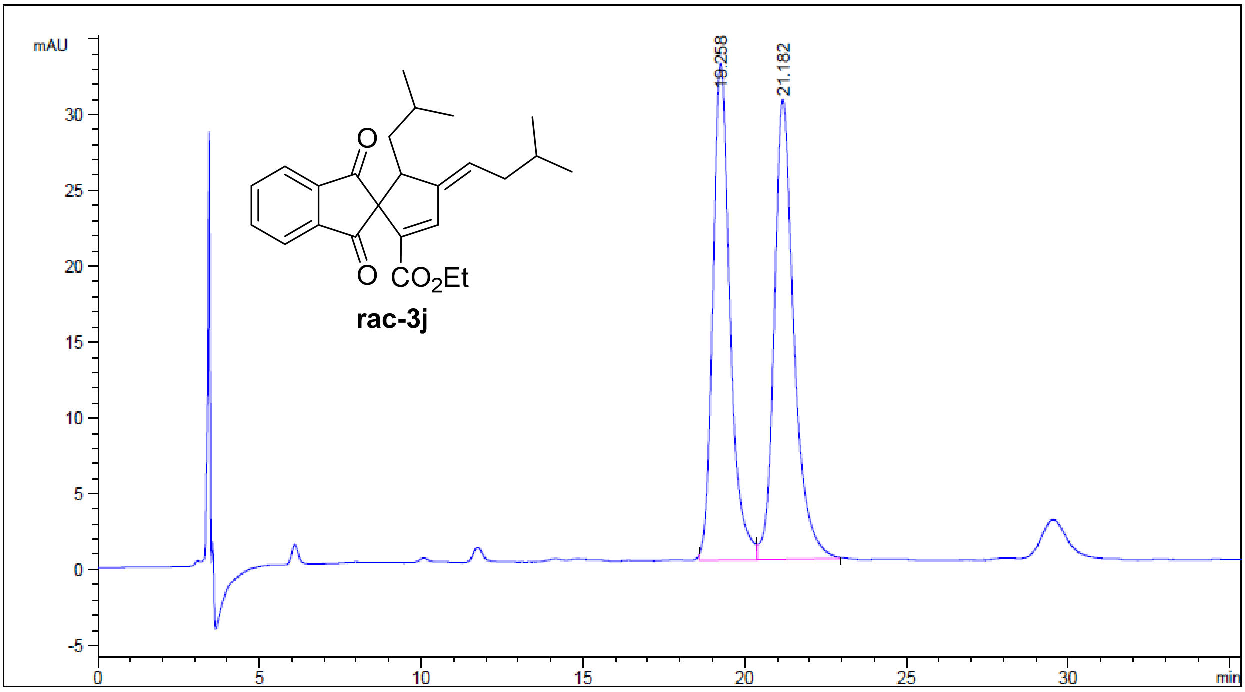

Peak #	RetTime [min]	Type	Width [min]	Area mAU *s	Height [mAU]	Area %
1	19.258	BV	0.5735	1205.44250	32.42207	48.3932
2	21.182	VB	0.6365	1285.49341	30.19384	51.6068

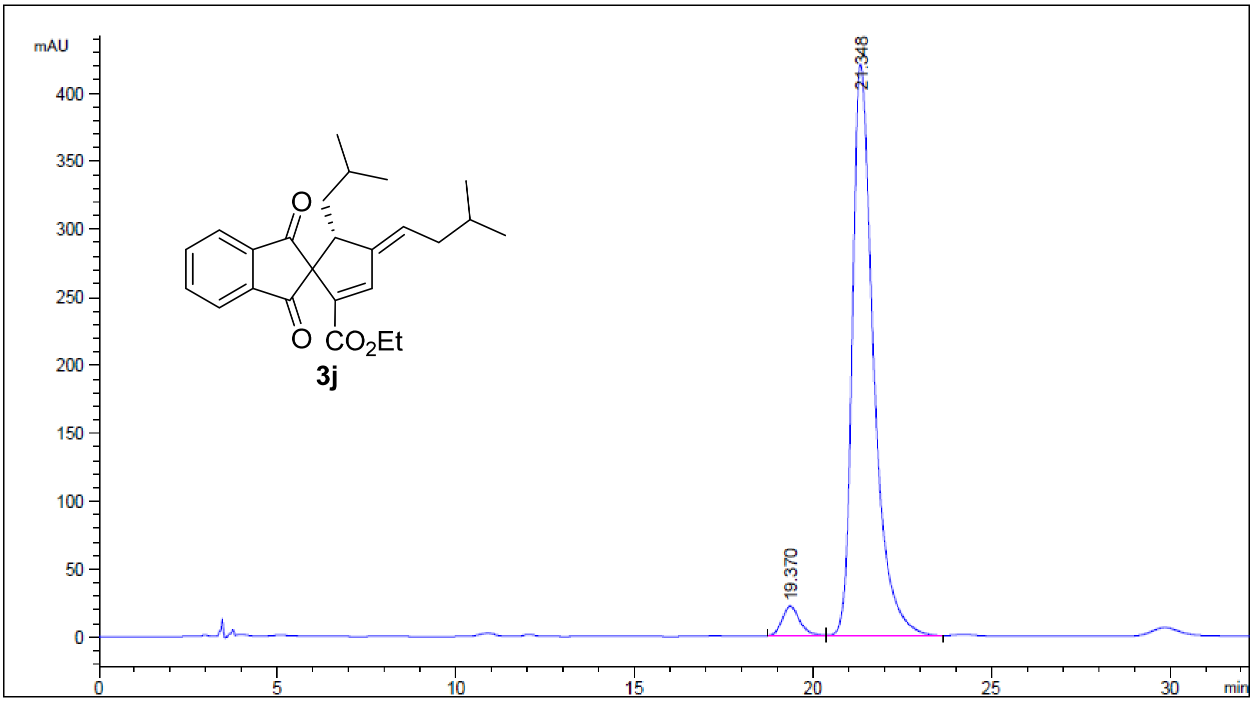

Peak #	RetTime [min]	Type	Width [min]	Area mAU *s	Height [mAU]	Area %
1	19.370	BV	0.5393	805.30505	22.21862	4.4048
2	21.348	VBA	0.6242	1.74773e4	420.90695	95.5952

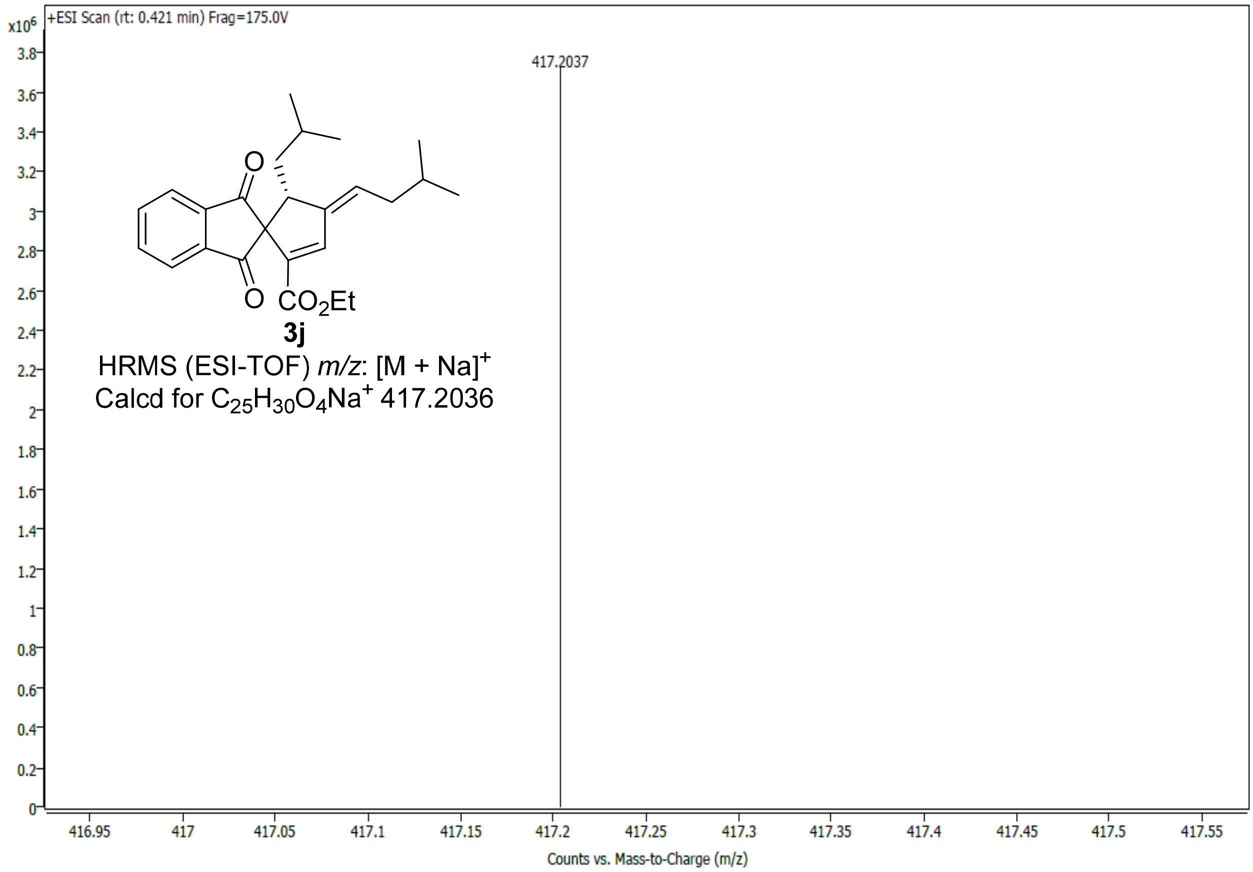
+ESI Scan (rt: 0.421 min) Frag=175.0V
x10^6
417.2037
O
O
CO2Et
3j
HRMS (ESI-TOF) m/z: [M + Na]+
Calcd for C25H30O4Na+ 417.2036
Counts vs. Mass-to-Charge (m/z)

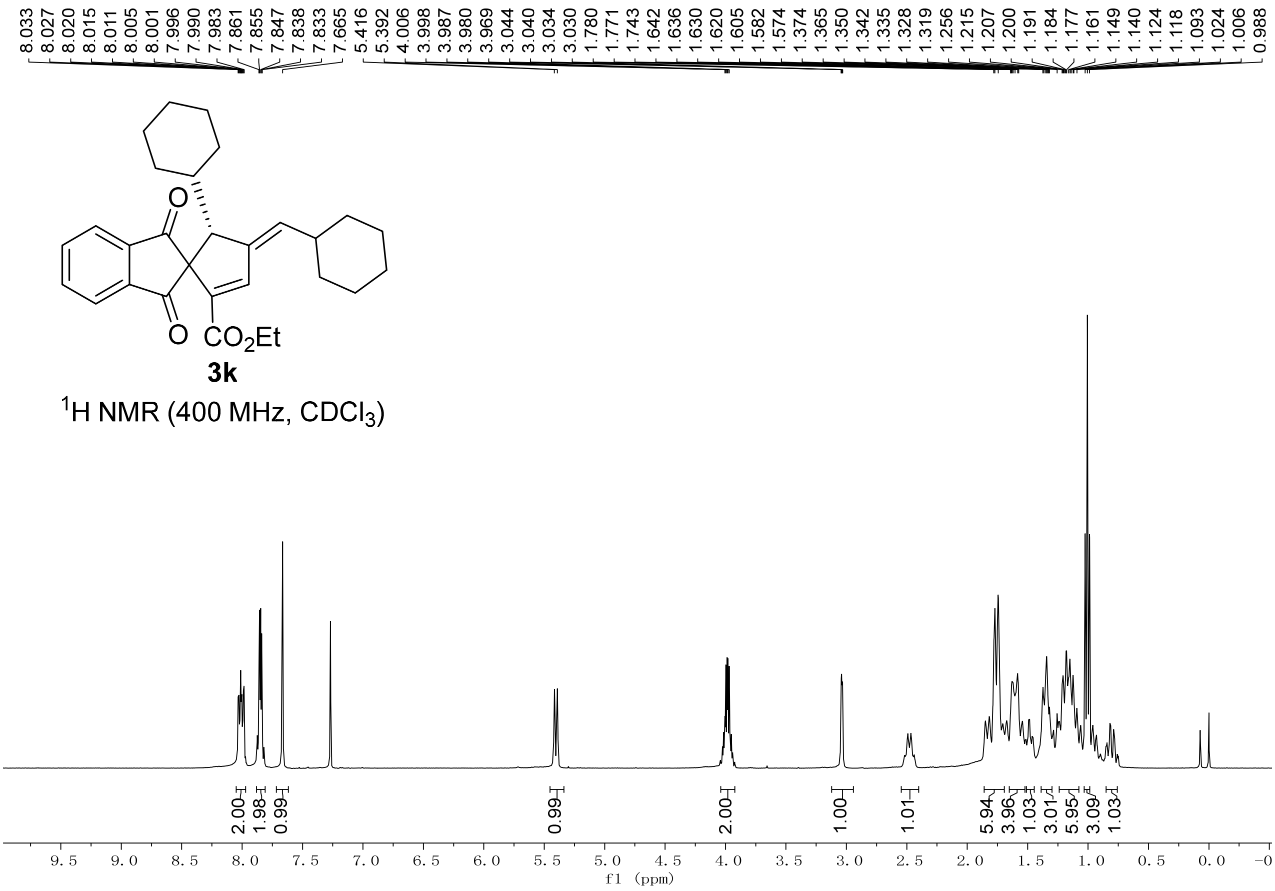
8.033 8.027 8.020 8.015 8.011 8.005 8.001 7.996 7.990 7.983 7.861 7.855 7.847 7.838 7.833 7.665 5.416 5.392 4.006 3.998 3.987 3.980 3.969 3.044 3.040 3.034 3.030 1.780 1.771 1.743 1.642 1.636 1.630 1.620 1.605 1.582 1.574 1.374 1.365 1.350 1.342 1.335 1.328 1.319 1.256 1.215 1.207 1.200 1.191 1.184 1.177 1.161 1.149 1.140 1.124 1.118 1.093 1.024 1.006 0.988
O
O
CO_2Et
3k
^1H NMR (400 MHz, $CDCl_3$)
2.00
1.98
0.99
0.99
2.00
1.00
1.01
5.94
3.96
1.03
3.01
5.95
3.09
1.03
9.5 9.0 8.5 8.0 7.5 7.0 6.5 6.0 5.5 5.0 4.5 4.0 3.5 3.0 2.5 2.0 1.5 1.0 0.5 0.0 −0
f1 (ppm)

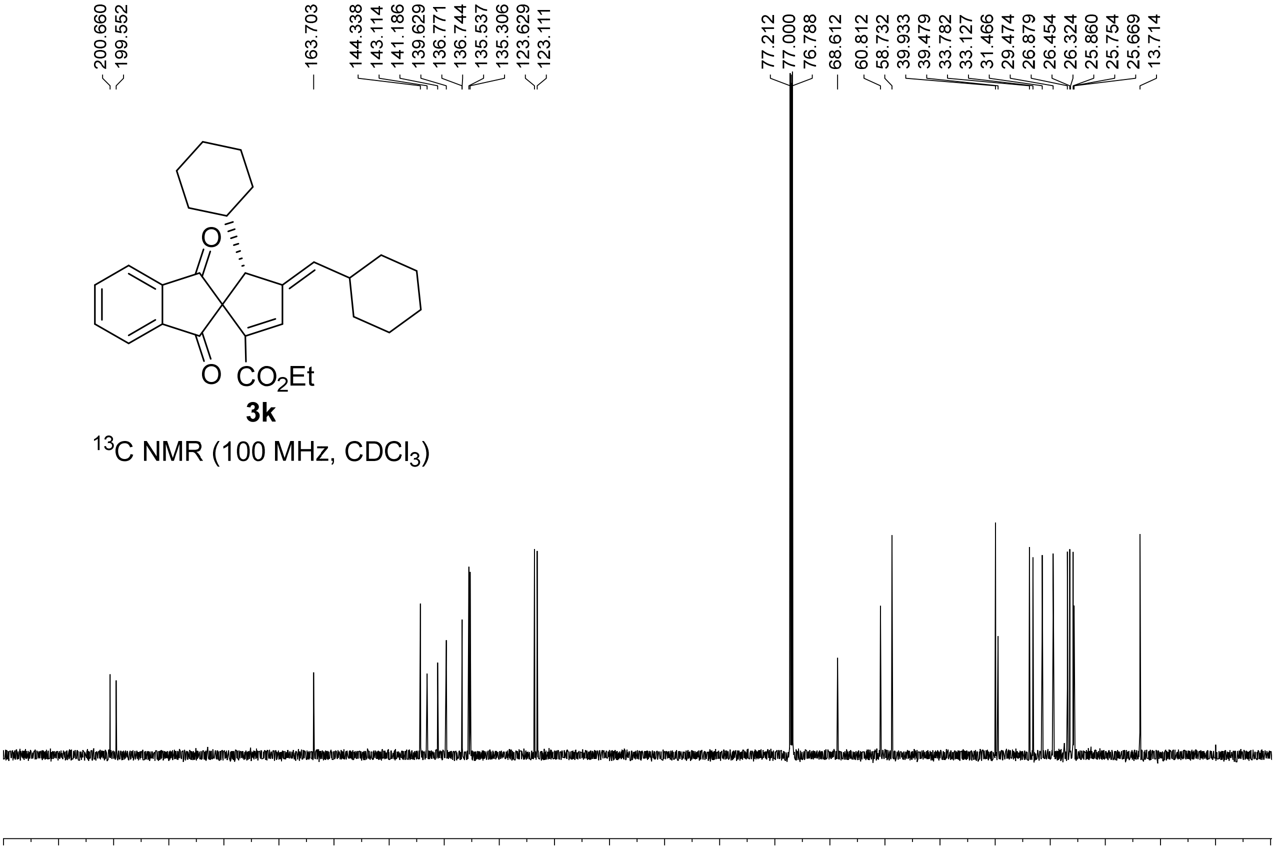
200.660 199.552
163.703
144.338 143.114 141.186 139.629 136.771 136.744 135.537 135.306 123.629 123.111
77.212 77.000 76.788
68.612
60.812 58.732 39.933 39.479 33.782 33.127 31.466 29.474 26.879 26.454 26.324 25.860 25.754 25.669 13.714
O
O
CO_2Et
3k
^{13}C NMR (100 MHz, $CDCl_3$)
20 210 200 190 180 170 160 150 140 130 120 110 100 90 80 70 60 50 40 30 20 10 0 −
f1 (ppm)

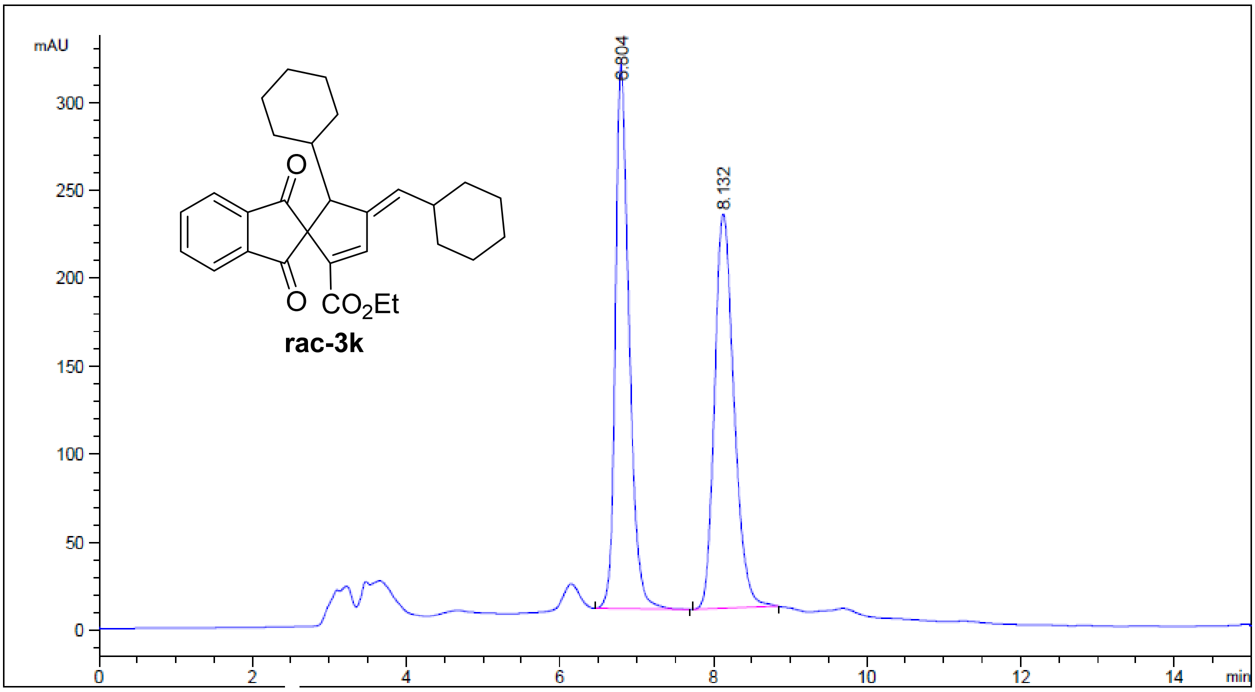

Peak #	RetTime [min]	Type	Width [min]	Area mAU *s	Height [mAU]	Area %
1	6.804	BB	0.1920	3837.53711	307.98605	49.4166
2	8.132	BBA	0.2752	3928.14941	223.20433	50.5834

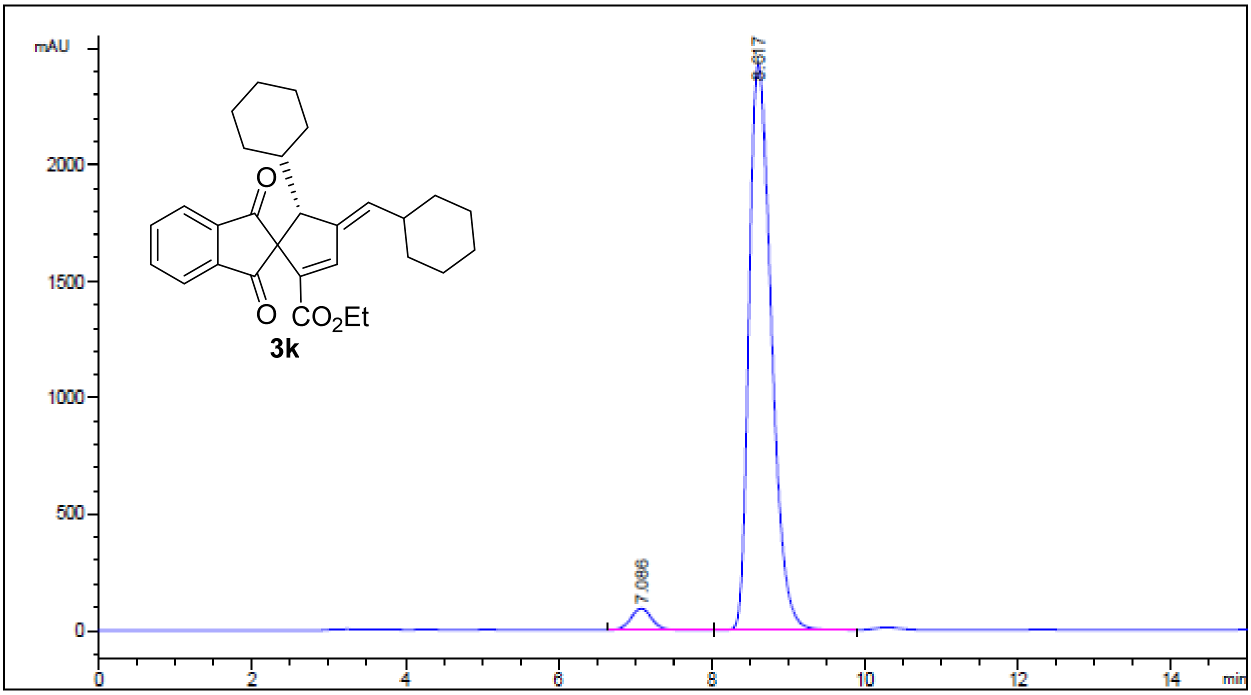

Peak #	RetTime [min]	Type	Width [min]	Area mAU *s	Height [mAU]	Area %
1	7.086	BB	0.3018	1845.45154	94.01748	3.6411
2	8.617	BV	0.3132	4.88390e4	2429.11499	96.3589

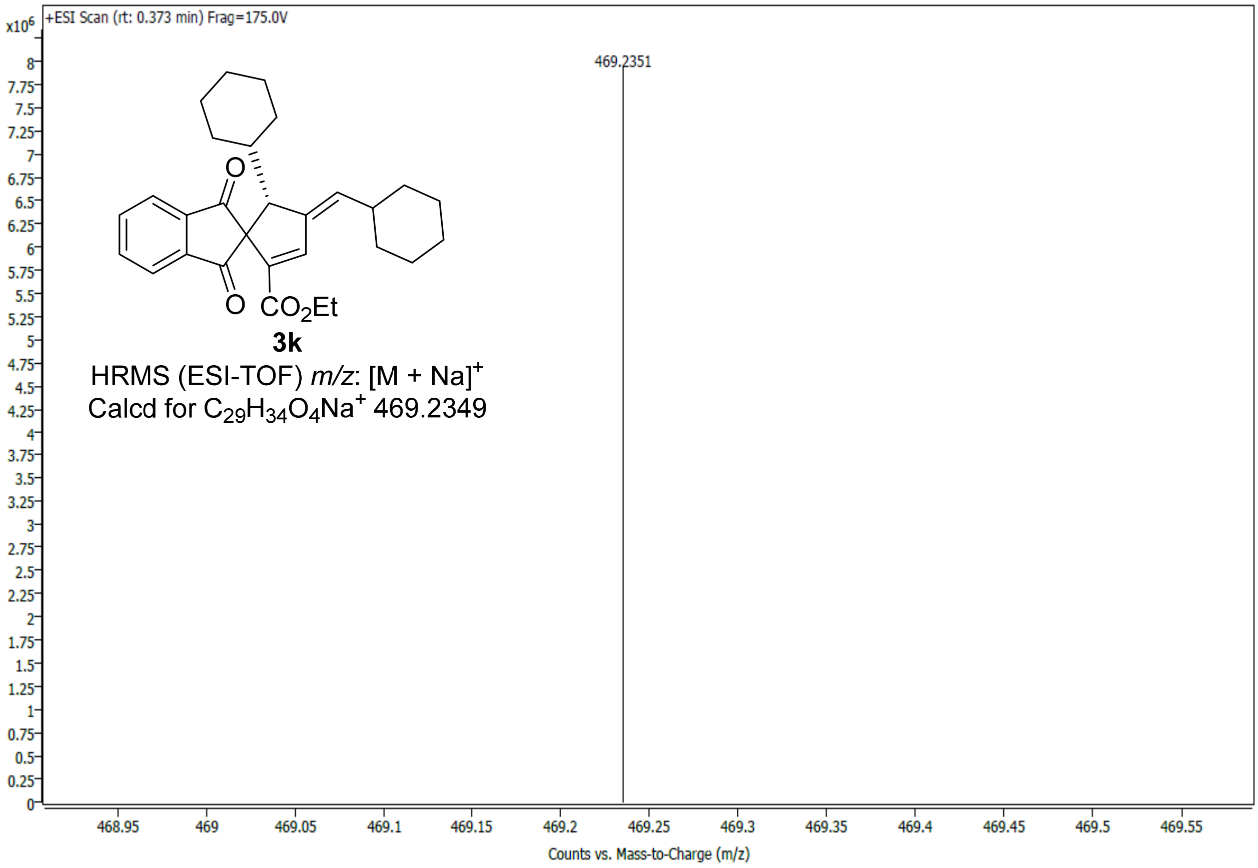
+ESI Scan (rt: 0.373 min) Frag=175.0V
x10^6
469.2351
O
O
CO2Et
3k
HRMS (ESI-TOF) m/z: [M + Na]+
Calcd for C29H34O4Na+ 469.2349
Counts vs. Mass-to-Charge (m/z)

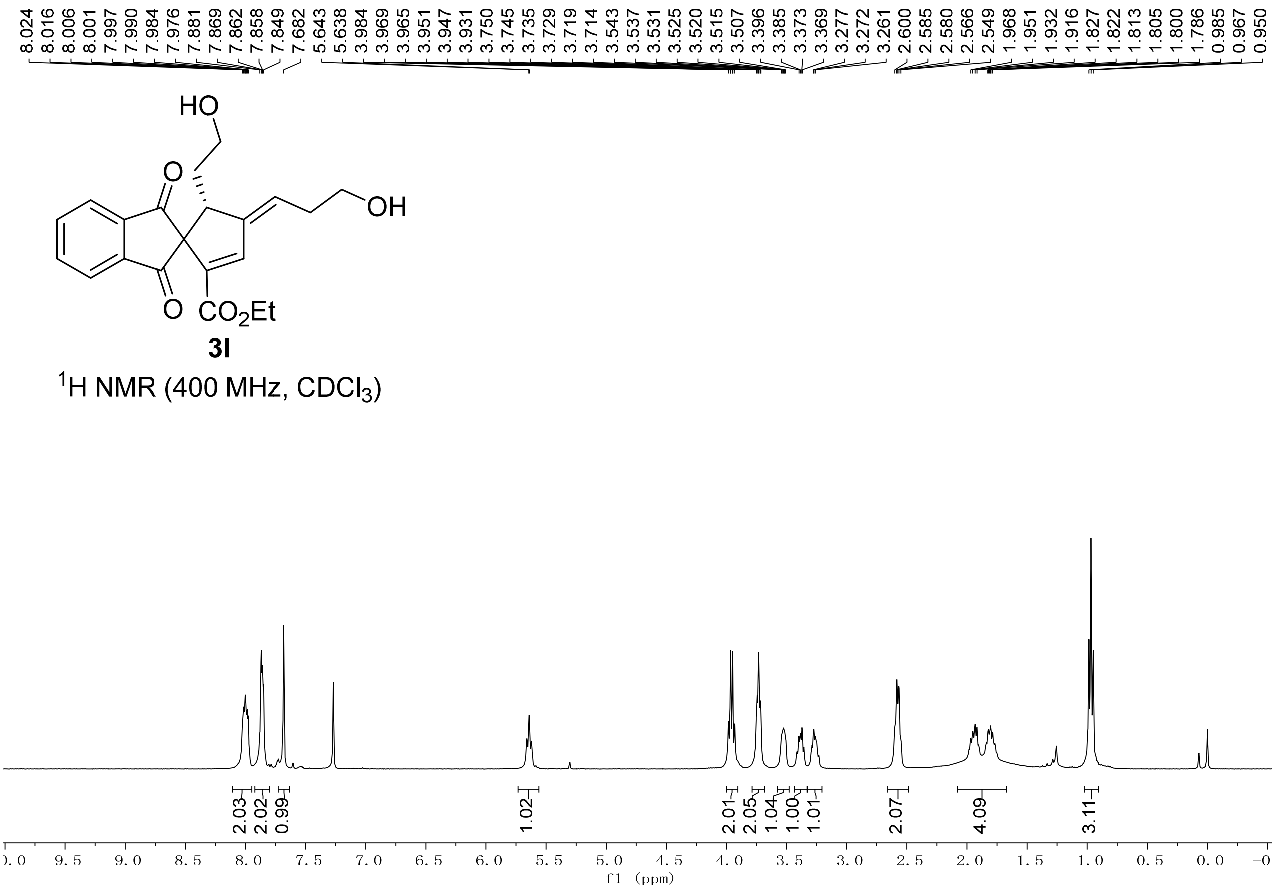
8.024
8.016
8.006
8.001
7.997
7.990
7.984
7.976
7.881
7.869
7.862
7.858
7.849
7.682
5.643
5.638
3.984
3.969
3.965
3.951
3.947
3.931
3.750
3.745
3.735
3.729
3.719
3.714
3.543
3.537
3.531
3.525
3.520
3.515
3.507
3.396
3.385
3.373
3.369
3.277
3.272
3.261
2.600
2.585
2.580
2.566
2.549
1.968
1.951
1.932
1.916
1.827
1.822
1.813
1.805
1.800
1.786
0.985
0.967
0.950
HO
O
OH
O
CO2Et
3l
1H NMR (400 MHz, CDCl3)
2.03
2.02
0.99
1.02
2.01
2.05
1.04
1.00
1.01
2.07
4.09
3.11
9.5
9.0
8.5
8.0
7.5
7.0
6.5
6.0
5.5
5.0
4.5
4.0
3.5
3.0
2.5
2.0
1.5
1.0
0.5
0.0
f1 (ppm)

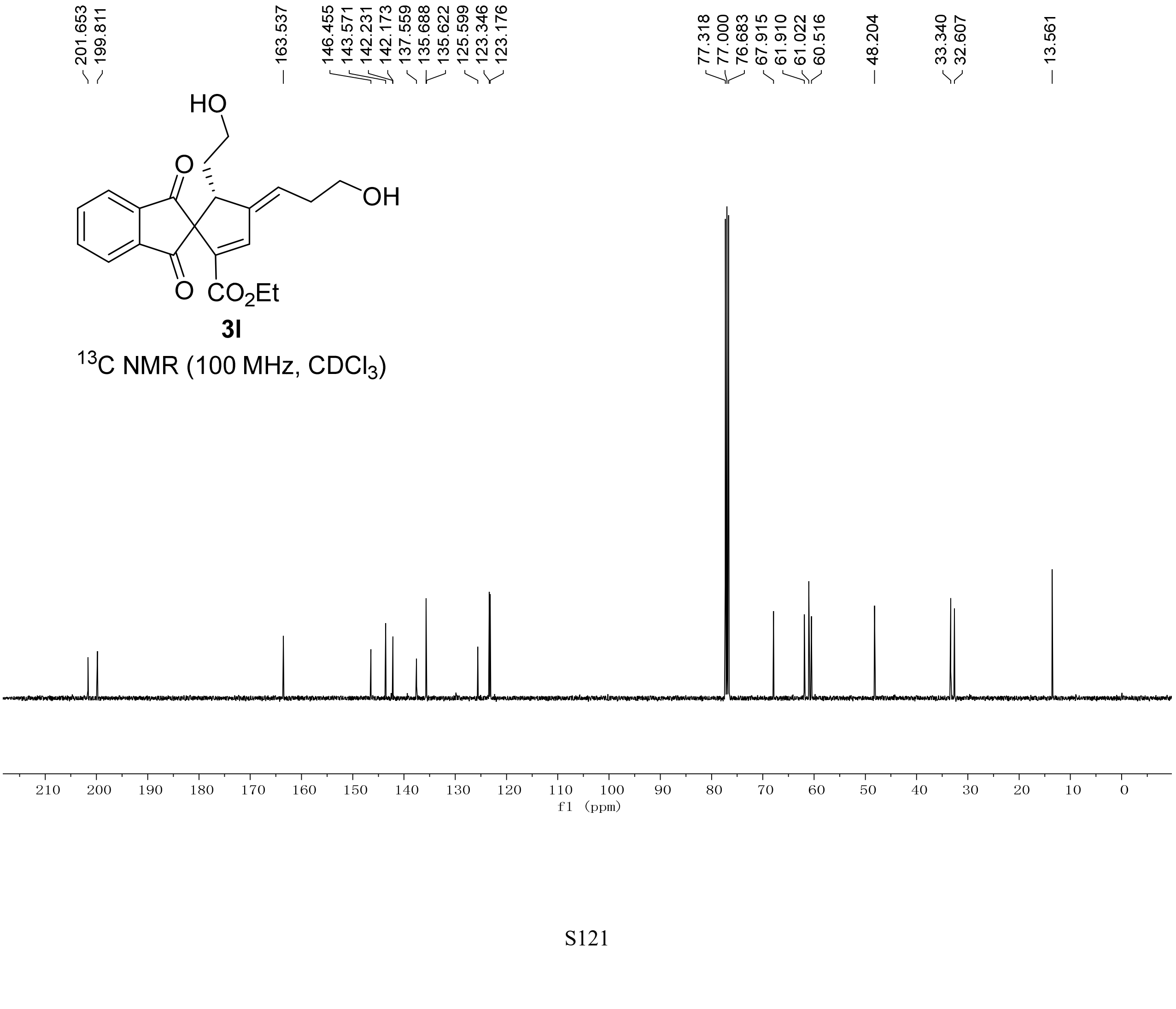
201.653
199.811
163.537
146.455
143.571
142.231
142.173
137.559
135.688
135.622
125.599
123.346
123.176
77.318
77.000
76.683
67.915
61.910
61.022
60.516
48.204
33.340
32.607
13.561
HO
O
OH
O
CO2Et
3l
13C NMR (100 MHz, CDCl3)
210
200
190
180
170
160
150
140
130
120
110
100
90
80
70
60
50
40
30
20
10
0
f1 (ppm)

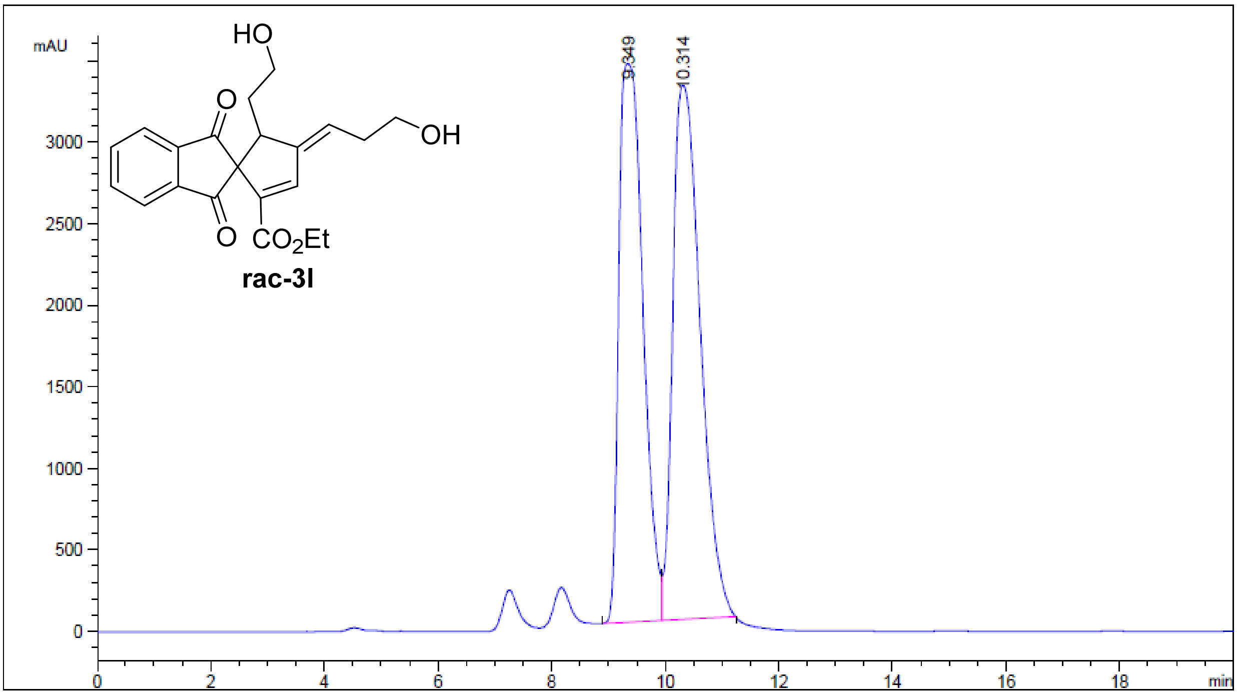

Peak #	RetTime [min]	Type	Width [min]	Area mAU *s	Height [mAU]	Area %
1	9.349	BV	0.4613	1.01110e5	3420.81152	47.7503
2	10.314	VBA	0.5225	1.10638e5	3272.65552	52.2497

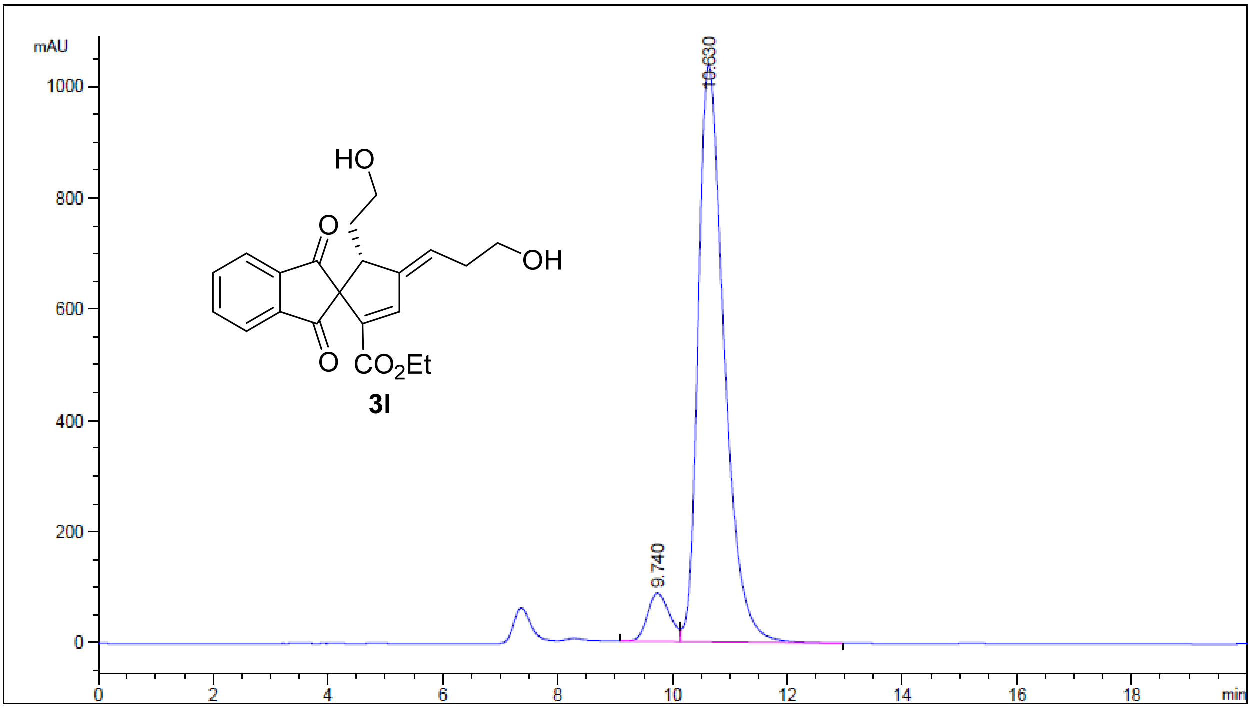

Peak #	RetTime [min]	Type	Width [min]	Area mAU *s	Height [mAU]	Area %
1	9.740	BV	0.4099	2305.18262	87.32944	6.5820
2	10.630	VB	0.4812	3.27172e4	1038.37830	93.4180

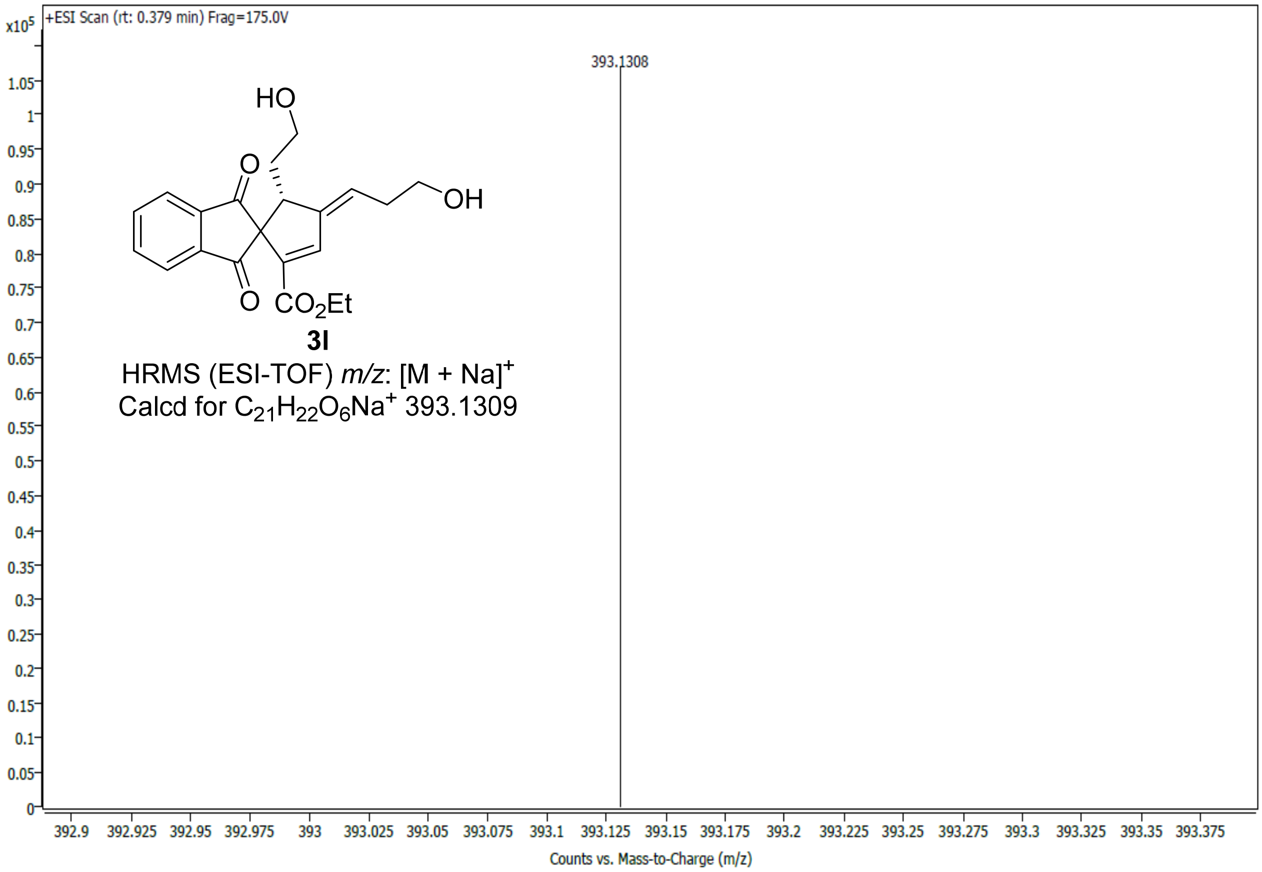
+ESI Scan (rt: 0.379 min) Frag=175.0V
x10^5
393.1308
HO
OH
O
O
CO2Et
3l
HRMS (ESI-TOF) m/z: [M + Na]+
Calcd for C21H22O6Na+ 393.1309
Counts vs. Mass-to-Charge (m/z)

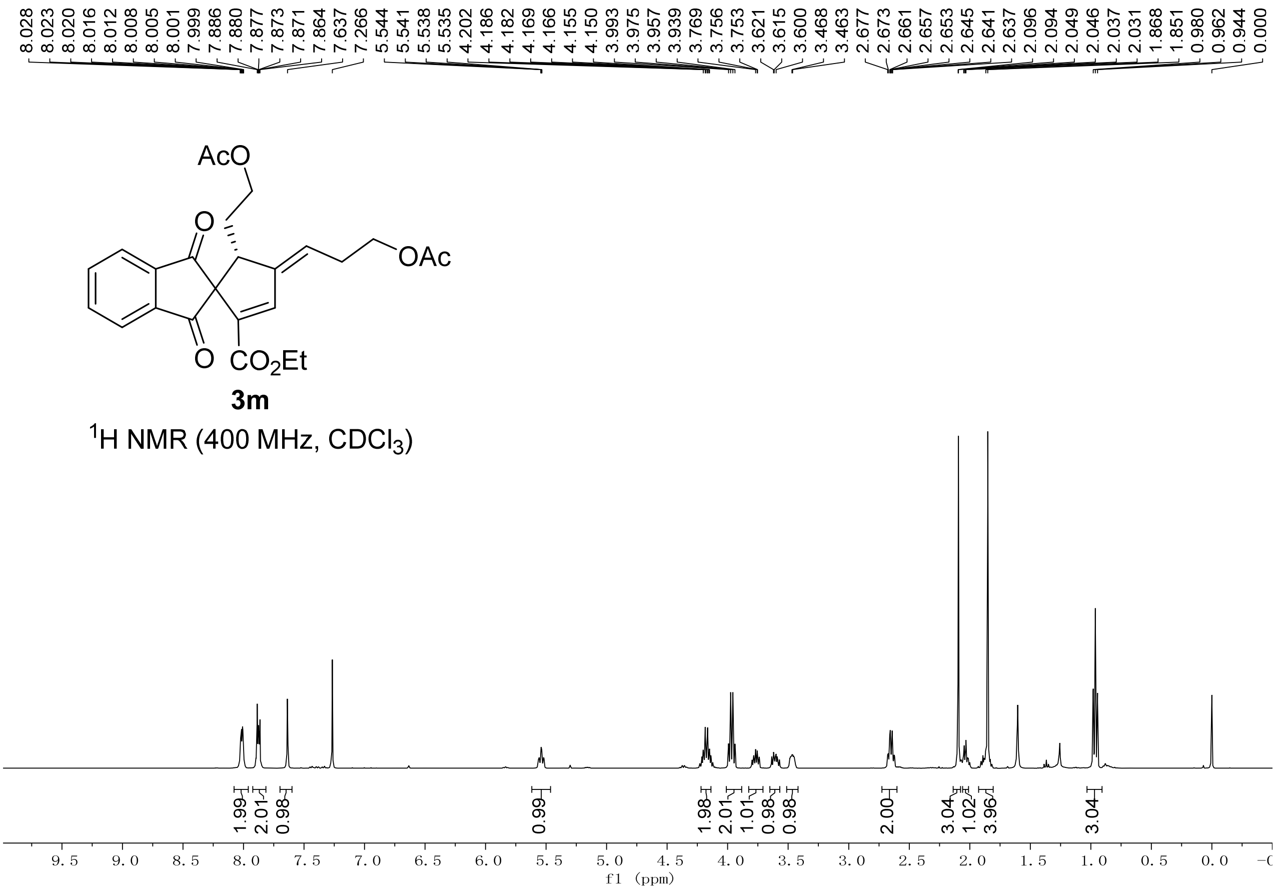
8.028 8.023 8.020 8.016 8.012 8.008 8.005 8.001 7.999 7.886 7.880 7.877 7.873 7.871 7.864 7.637 7.266 5.544 5.541 5.538 5.535 4.202 4.186 4.182 4.169 4.166 4.155 4.150 3.993 3.975 3.957 3.939 3.769 3.756 3.753 3.621 3.615 3.600 3.468 3.463 2.677 2.673 2.661 2.657 2.653 2.645 2.641 2.637 2.096 2.094 2.049 2.046 2.037 2.031 1.868 1.851 0.980 0.962 0.944 0.000
AcO
O
OAc
O
CO2Et
3m
1H NMR (400 MHz, CDCl3)
1.99 2.01 0.98 0.99 1.98 2.01 1.01 0.98 0.98 2.00 3.04 1.02 3.96 3.04
9.5 9.0 8.5 8.0 7.5 7.0 6.5 6.0 5.5 5.0 4.5 4.0 3.5 3.0 2.5 2.0 1.5 1.0 0.5 0.0
f1 (ppm)

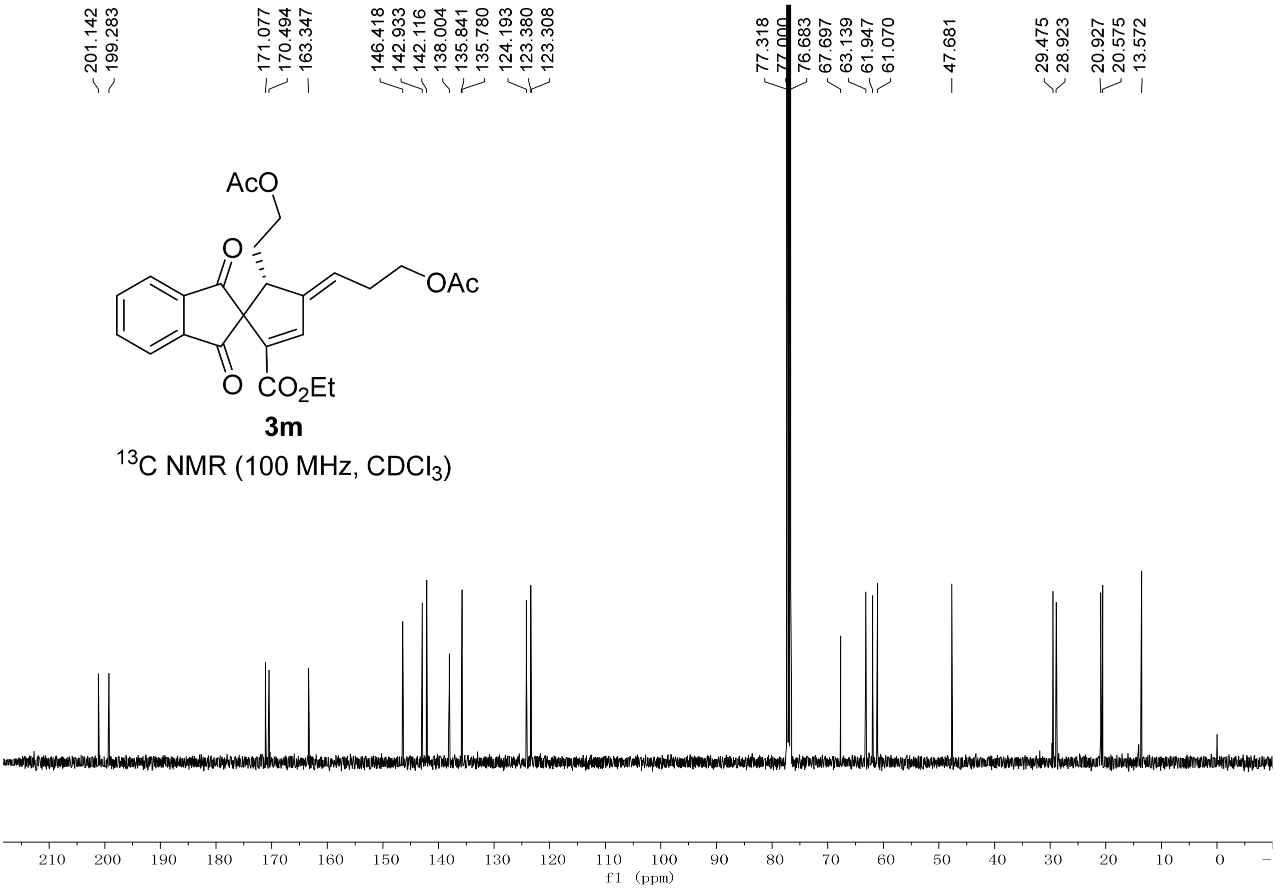
201.142 199.283 171.077 170.494 163.347 146.418 142.933 142.116 138.004 135.841 135.780 124.193 123.380 123.308 77.318 77.000 76.683 67.697 63.139 61.947 61.070 47.681 29.475 28.923 20.927 20.575 13.572
AcO
O
OAc
O
CO2Et
3m
13C NMR (100 MHz, CDCl3)
210 200 190 180 170 160 150 140 130 120 110 100 90 80 70 60 50 40 30 20 10 0
f1 (ppm)

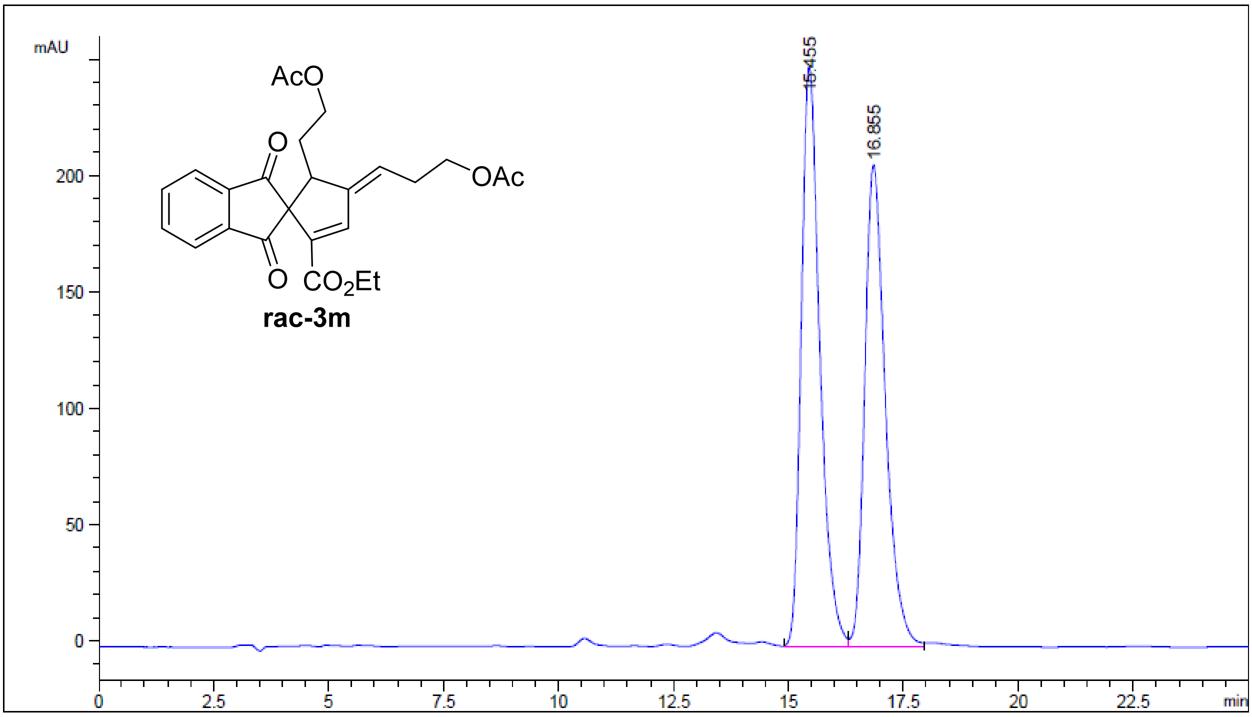

Peak #	RetTime [min]	Type	Width [min]	Area mAU *s	Height [mAU]	Area %
1	15.455	BV	0.4341	7020.06299	248.74017	52.0926
2	16.855	VBA	0.4751	6456.05469	206.65430	47.9074

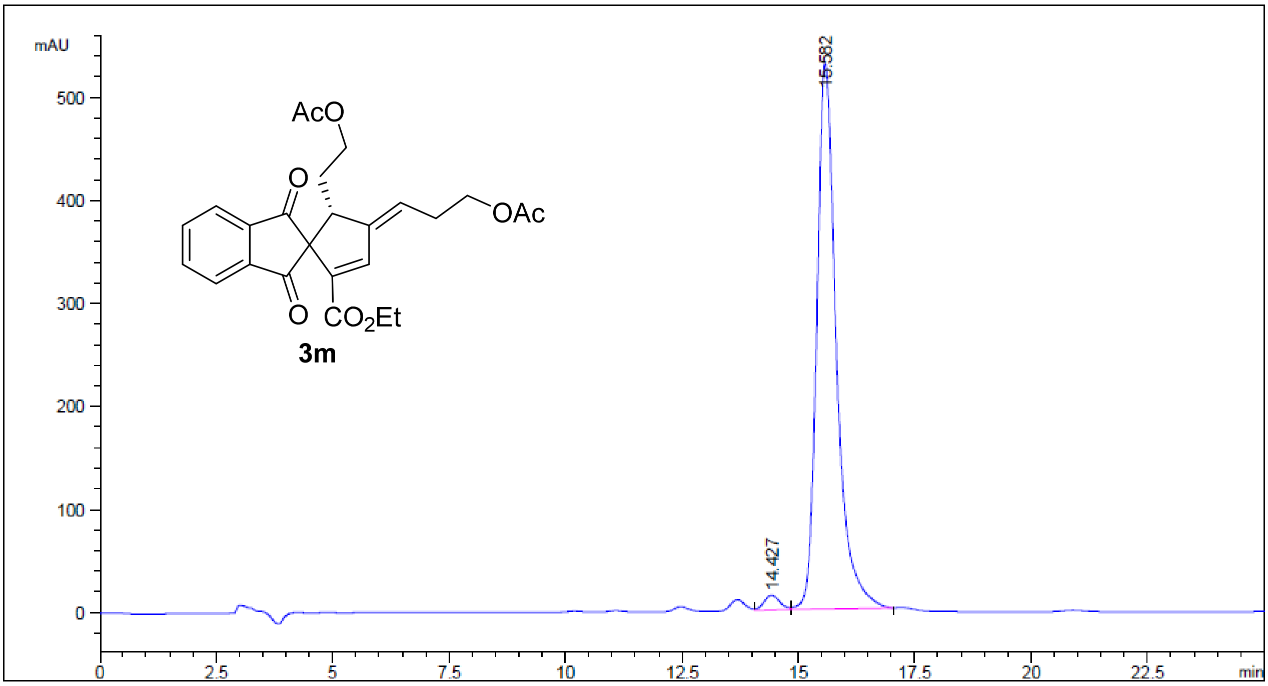

Peak #	RetTime [min]	Type	Width [min]	Area mAU *s	Height [mAU]	Area %
1	14.427	VV	0.3691	340.45947	14.39745	2.1465
2	15.582	VBA	0.4408	1.55209e4	529.74603	97.8535

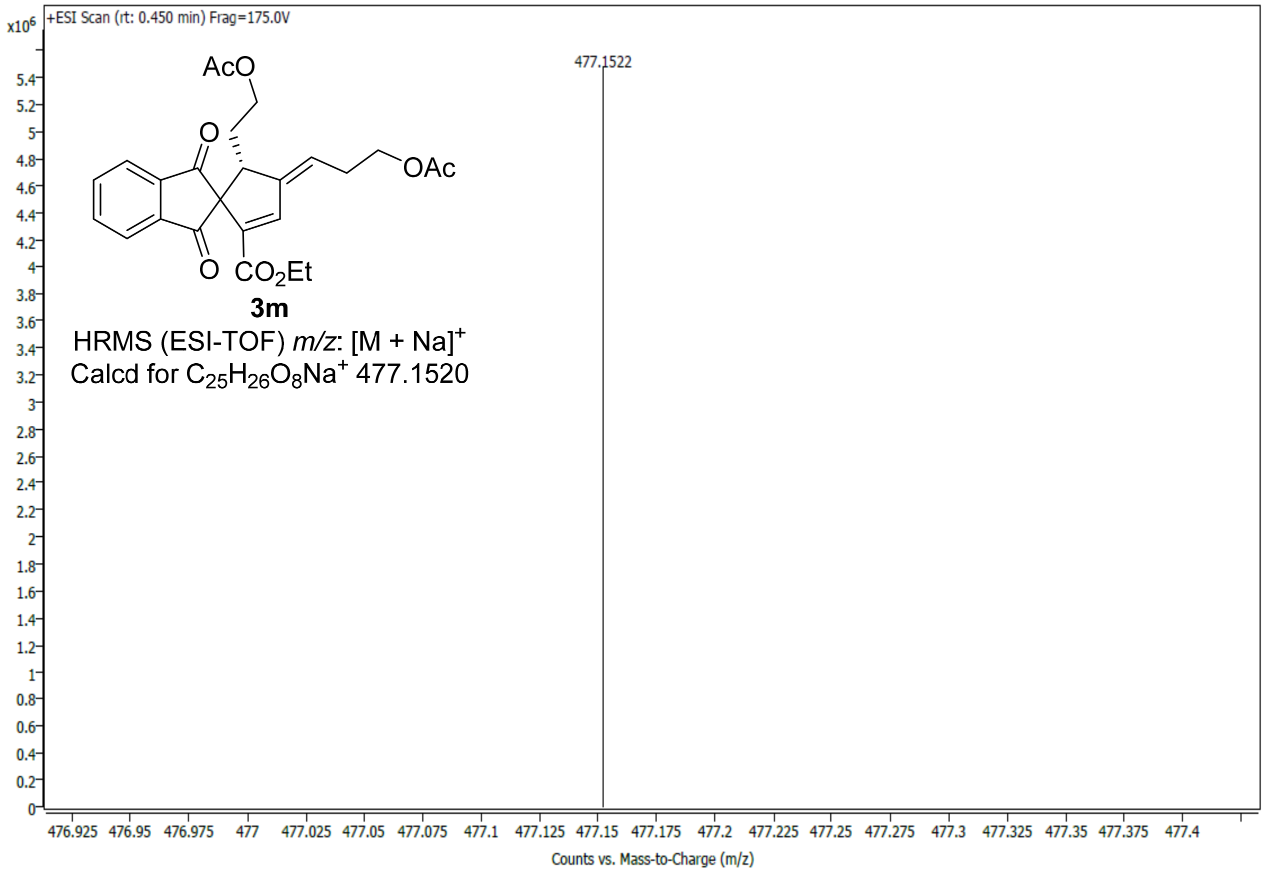
+ESI Scan (rt: 0.450 min) Frag=175.0V
x10^6
477.1522
AcO
O
OAc
O
CO2Et
3m
HRMS (ESI-TOF) m/z: [M + Na]+
Calcd for C25H26O8Na+ 477.1520
Counts vs. Mass-to-Charge (m/z)

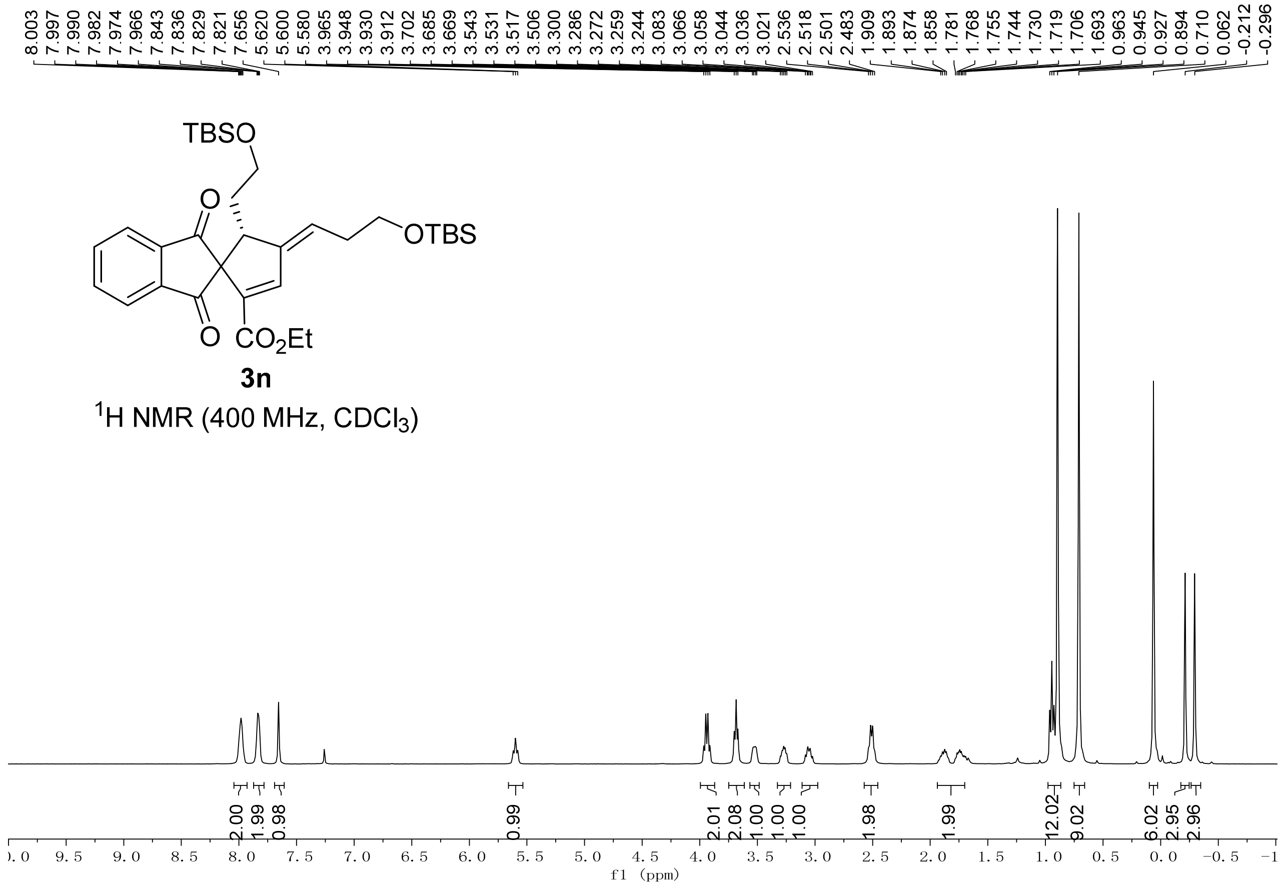
TBSO
O
OTBS
O
CO2Et
3n
1H NMR (400 MHz, CDCl3)
f1 (ppm)

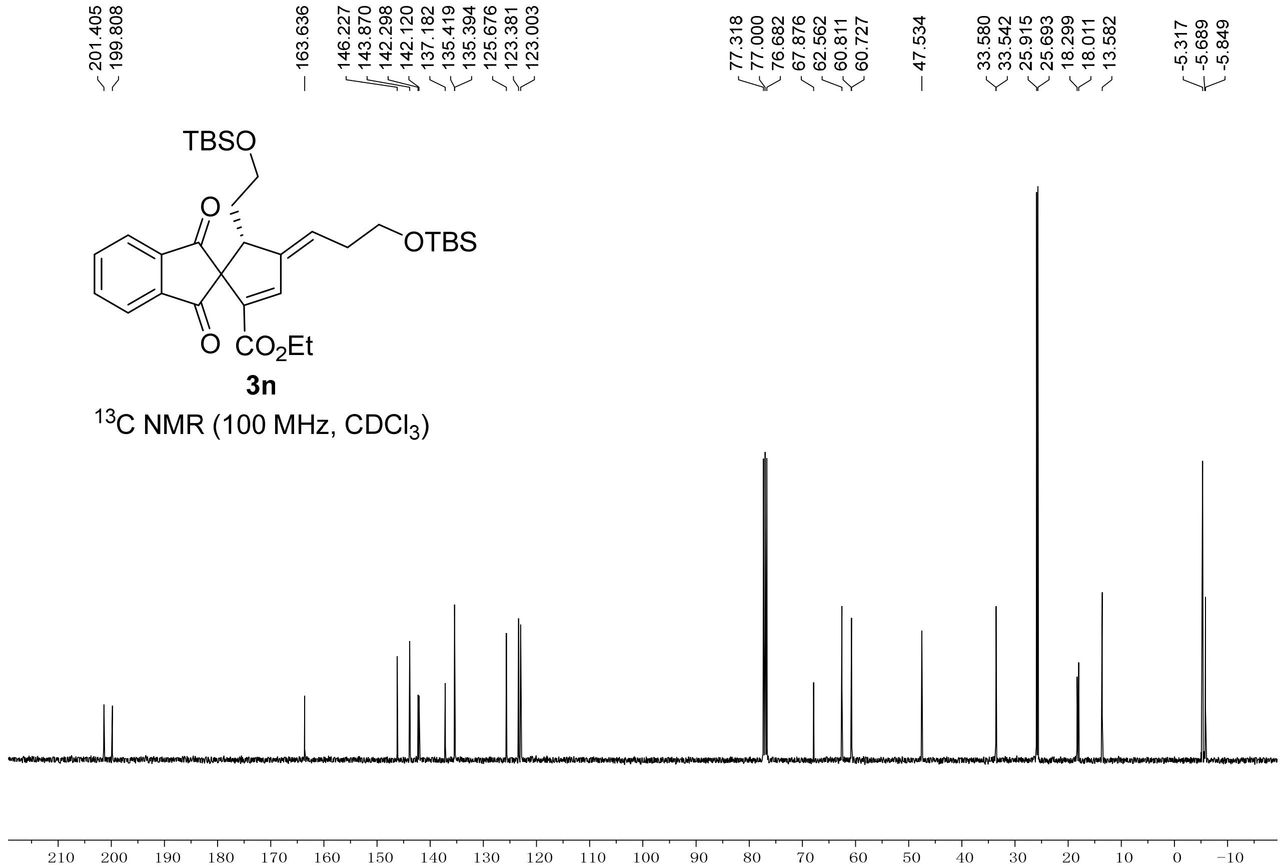
TBSO
O
OTBS
O
CO2Et
3n
13C NMR (100 MHz, CDCl3)
f1 (ppm)

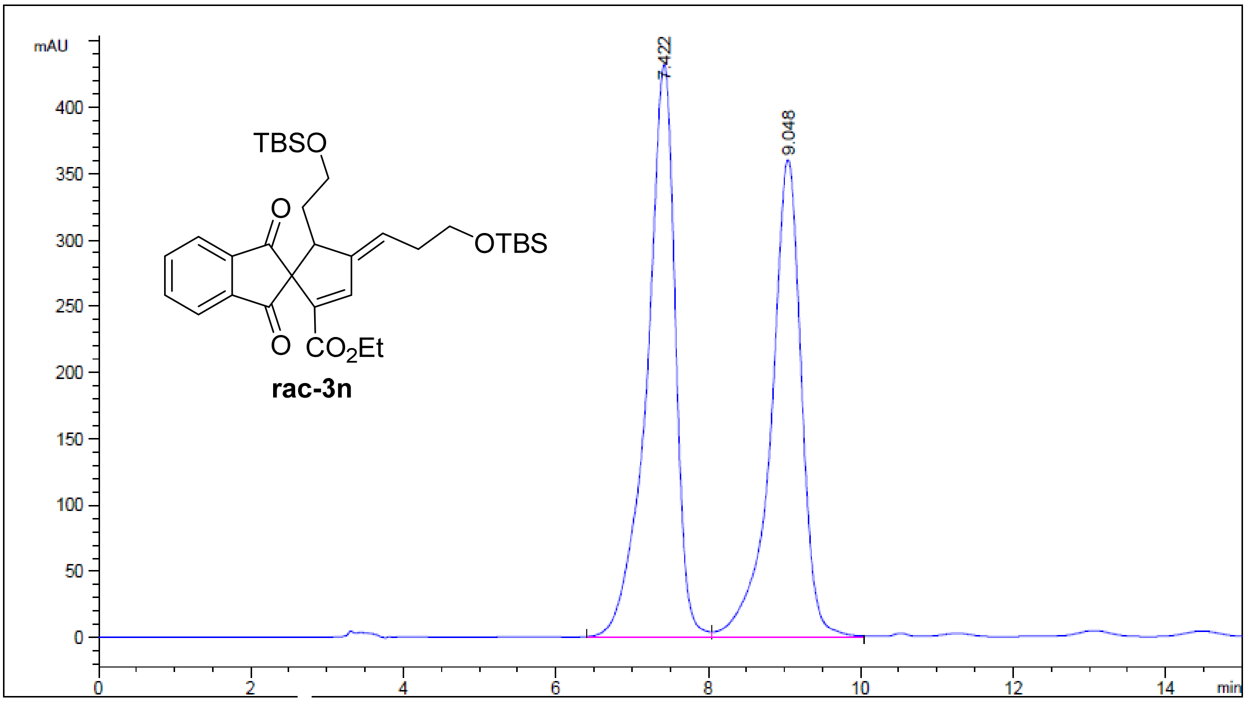

Peak #	RetTime [min]	Type	Width [min]	Area mAU *s	Height [mAU]	Area %
1	7.422	BV	0.3828	1.11465e4	431.68265	52.7775
2	9.048	VBA	0.4106	9973.35156	359.96710	47.2225

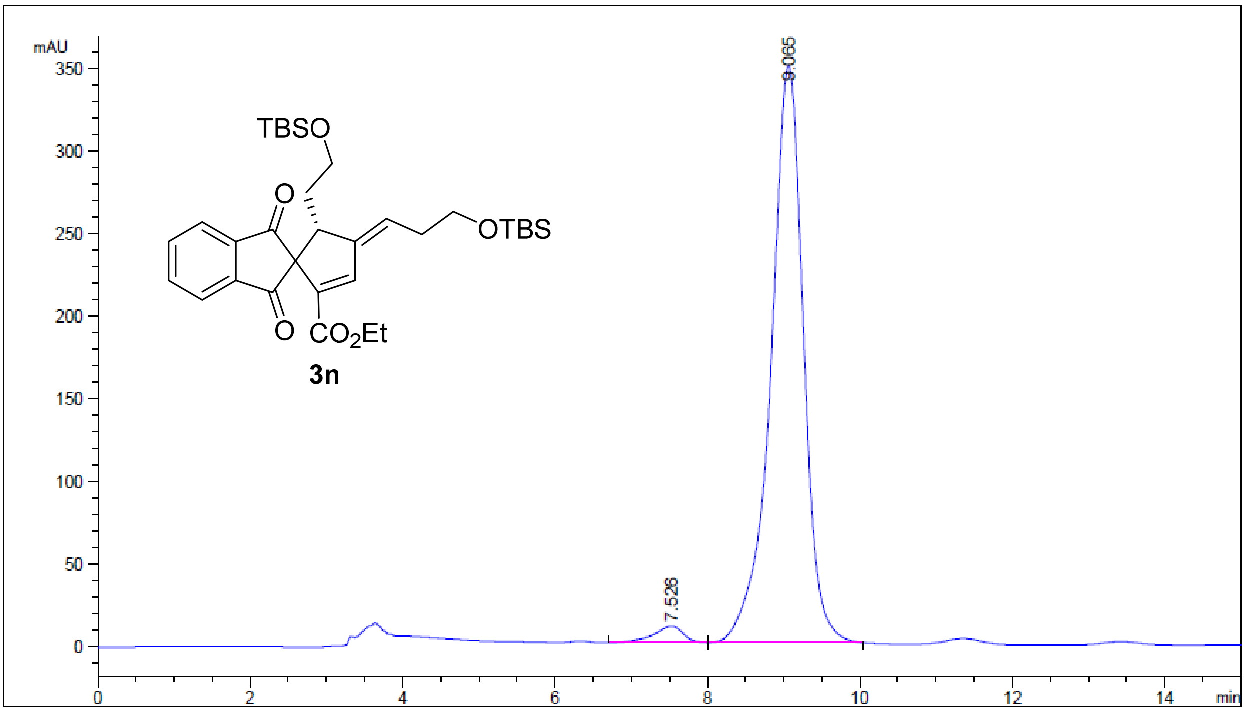

Peak #	RetTime [min]	Type	Width [min]	Area mAU *s	Height [mAU]	Area %
1	7.526	BV	0.4112	275.20209	10.09569	2.5854
2	9.065	VBA	0.4516	1.03694e4	348.87341	97.4146

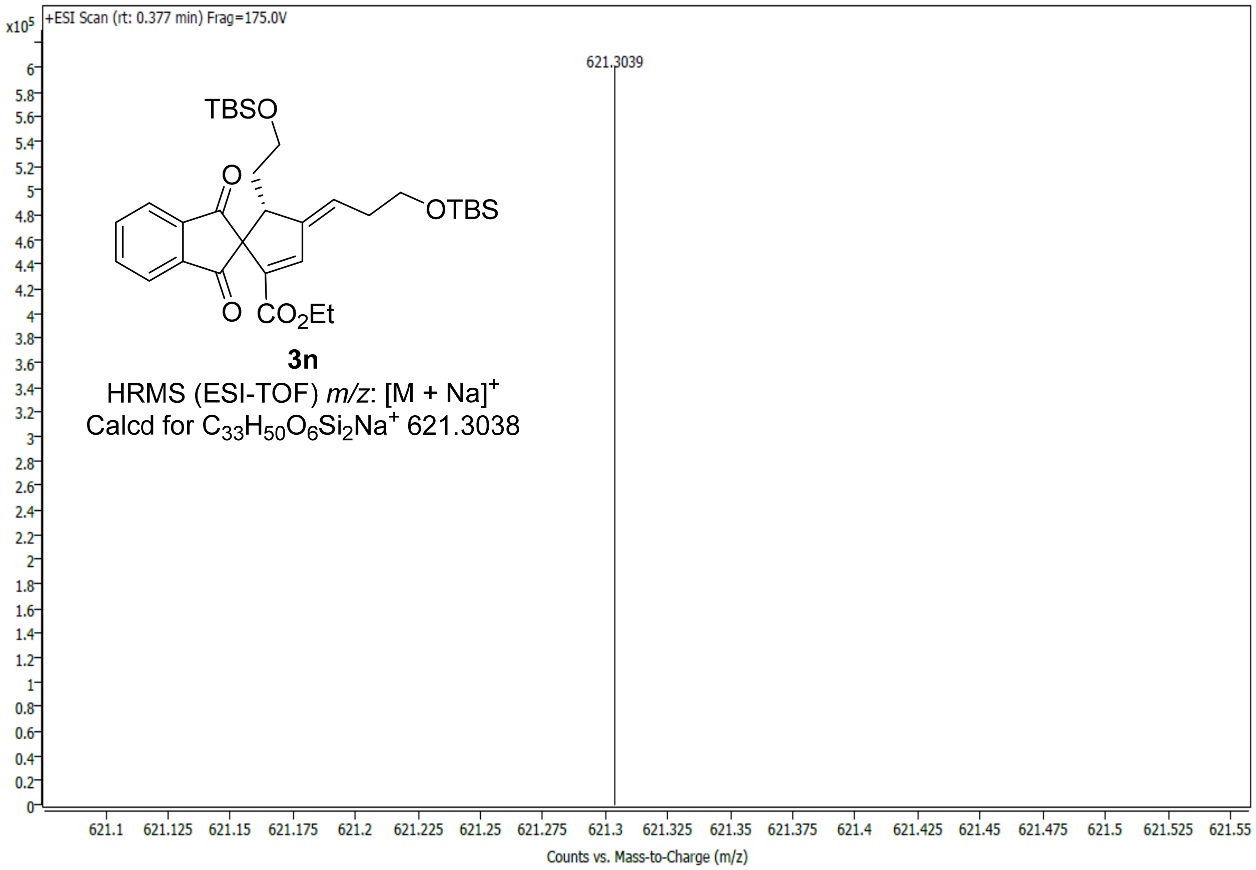

x10^5
+ESI Scan (rt: 0.377 min) Frag=175.0V
621.3039
TBSO
O
OTBS
O
CO2Et
3n
HRMS (ESI-TOF) m/z: [M + Na]+
Calcd for C33H50O6Si2Na+ 621.3038
Counts vs. Mass-to-Charge (m/z)

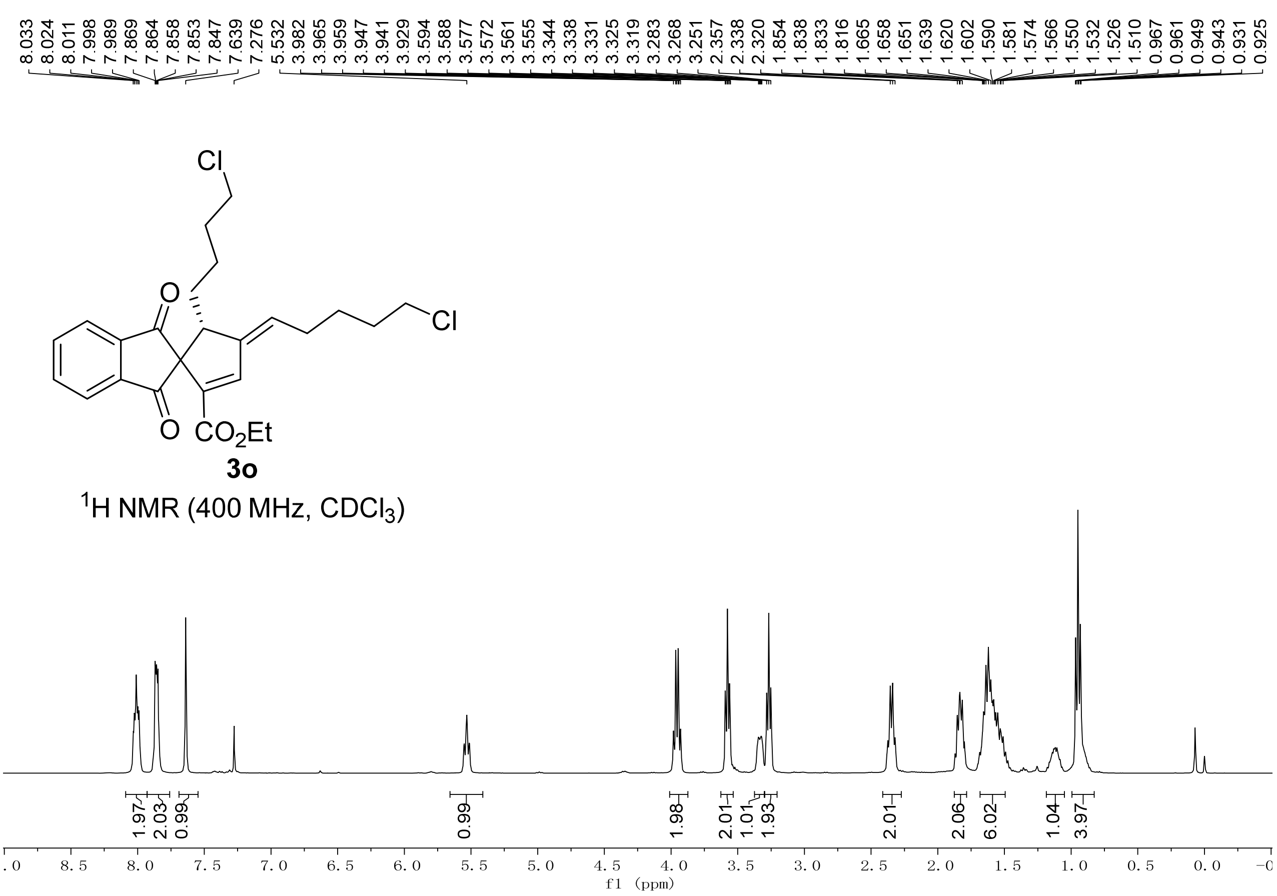
8.033 8.024 8.011 7.998 7.989 7.869 7.864 7.858 7.853 7.847 7.639 7.276 5.532 3.982 3.965 3.959 3.947 3.941 3.929 3.594 3.588 3.577 3.572 3.561 3.555 3.344 3.338 3.331 3.325 3.319 3.283 3.268 3.251 2.357 2.338 2.320 1.854 1.838 1.833 1.816 1.665 1.658 1.651 1.639 1.620 1.602 1.590 1.581 1.574 1.566 1.550 1.532 1.526 1.510 0.967 0.961 0.949 0.943 0.931 0.925
Cl
O
Cl
O
CO_2Et
3o
^1H NMR (400 MHz, $CDCl_3$)
1.97 2.03 0.99 0.99 1.98 2.01 1.01 1.93 2.01 2.06 6.02 1.04 3.97
f1 (ppm)

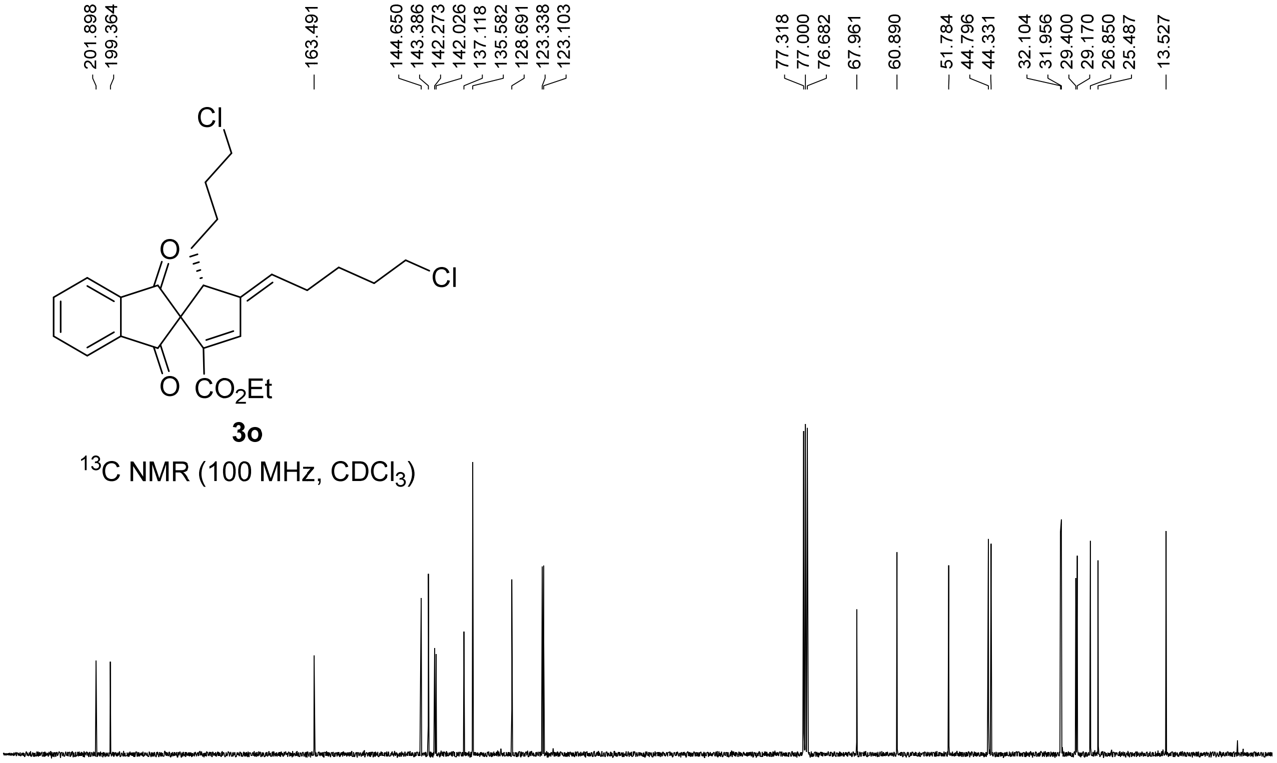
201.898 199.364 163.491 144.650 143.386 142.273 142.026 137.118 135.582 128.691 123.338 123.103 77.318 77.000 76.682 67.961 60.890 51.784 44.796 44.331 32.104 31.956 29.400 29.170 26.850 25.487 13.527
Cl
O
Cl
O
CO_2Et
3o
^{13}C NMR (100 MHz, $CDCl_3$)
f1 (ppm)

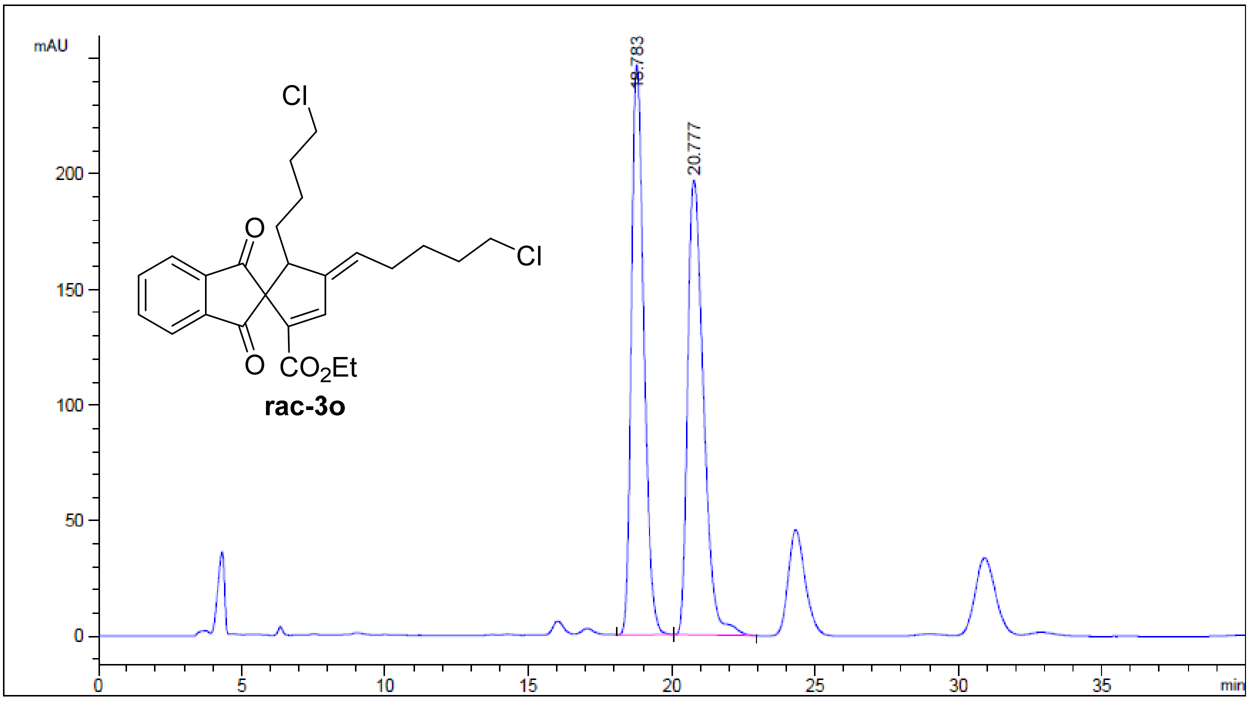

Peak #	RetTime [min]	Type	Width [min]	Area mAU *s	Height [mAU]	Area %
1	18.783	BB	0.4706	7484.61670	246.55188	49.6288
2	20.777	BBA	0.6018	7596.58838	196.76341	50.3712

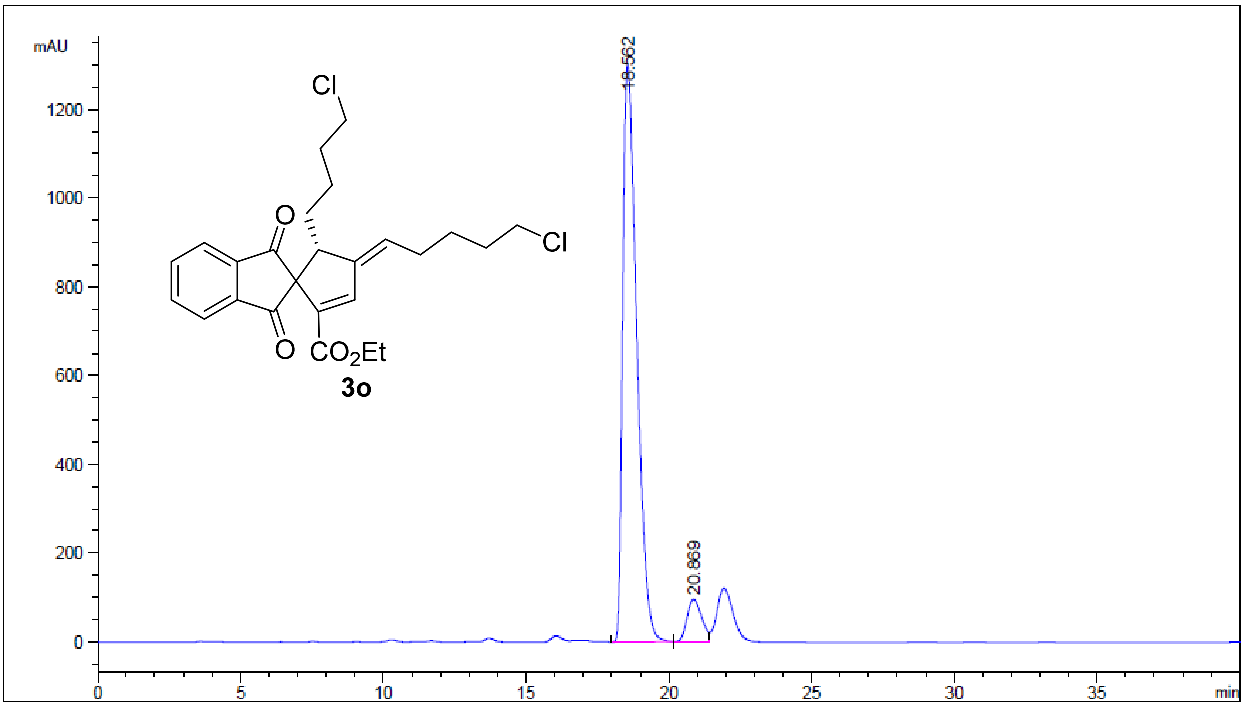

Peak #	RetTime [min]	Type	Width [min]	Area mAU *s	Height [mAU]	Area %
1	18.562	BB	0.5329	4.44110e4	1298.86353	92.9143
2	20.869	BV	0.5631	3386.82617	94.63673	7.0857

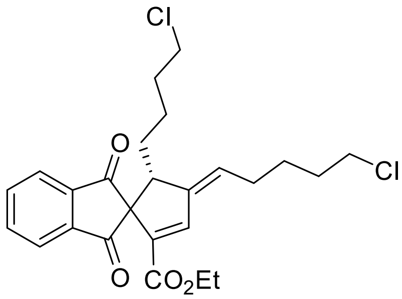

3o

HRMS (ESI-TOF) *m/z*: [M + H]$^+$
Calcd for $C_{25}H_{29}{}^{35}Cl_2O_4{}^+$ 463.1437
Calcd for $C_{25}H_{29}{}^{35}Cl^{37}ClO_4{}^+$ 465.1408

Spectrum from 20250517.wiff2 (sample 66) - 67, +TOF MS (300 - 600) from 0.019 to 0.166 min, subtr...um from 20250517.wiff2 (sample 66) - 67, +TOF MS (300 - 600) from 0.904 to 1.824 min], centroided

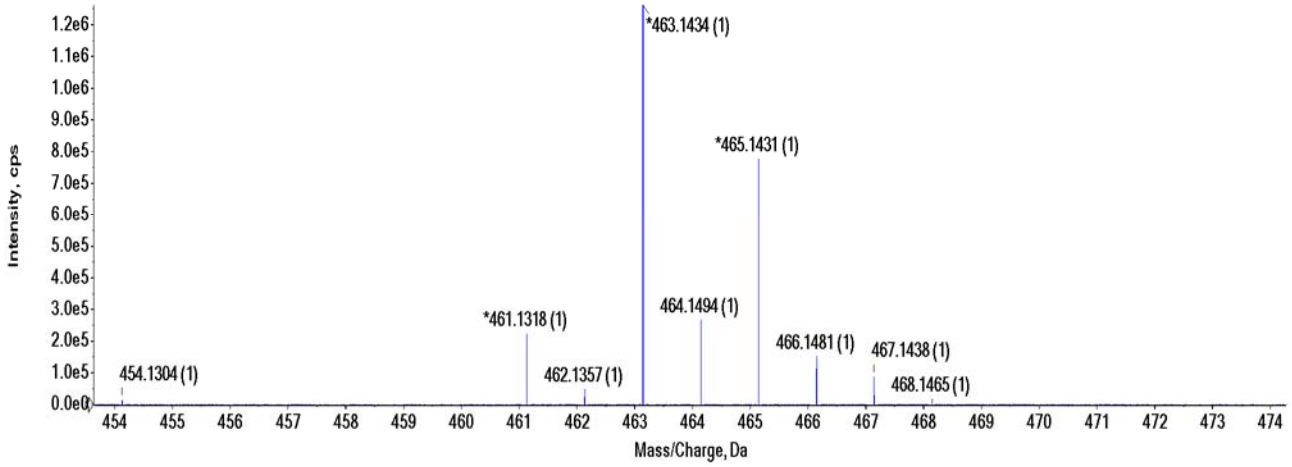

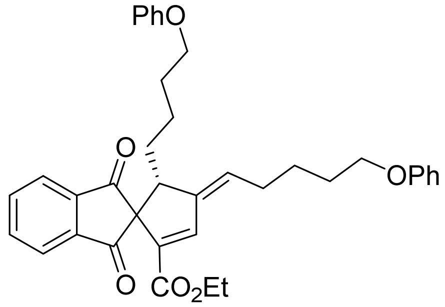

3p

^1H NMR (400 MHz, $CDCl_3$)

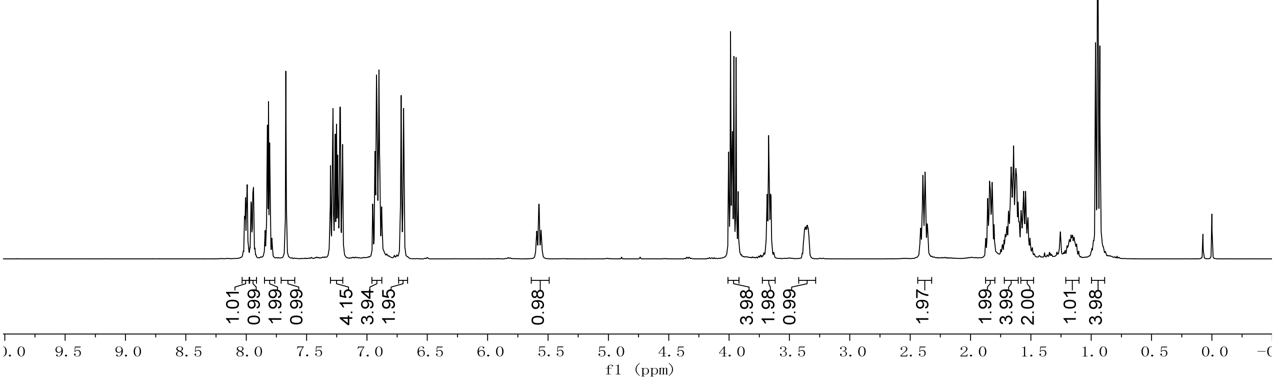

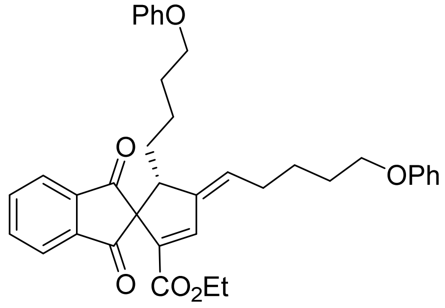

3p

^{13}C NMR (100 MHz, $CDCl_3$)

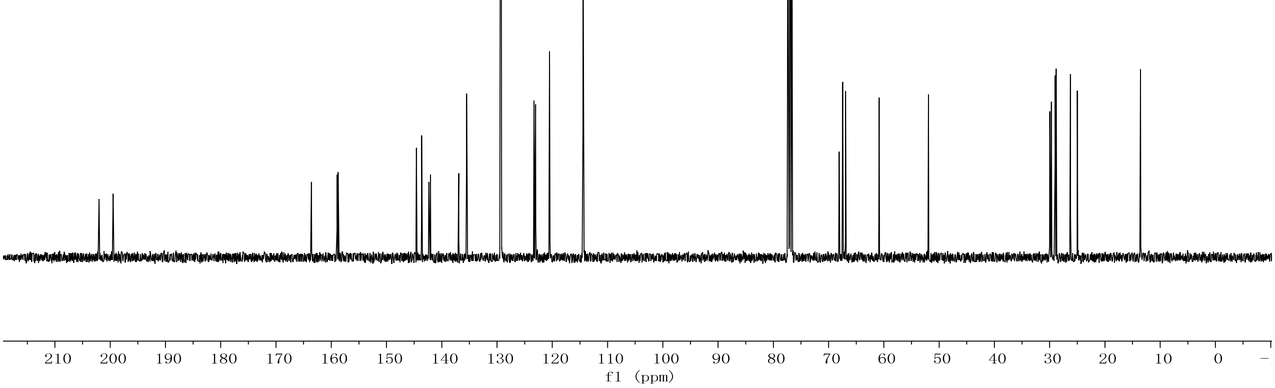

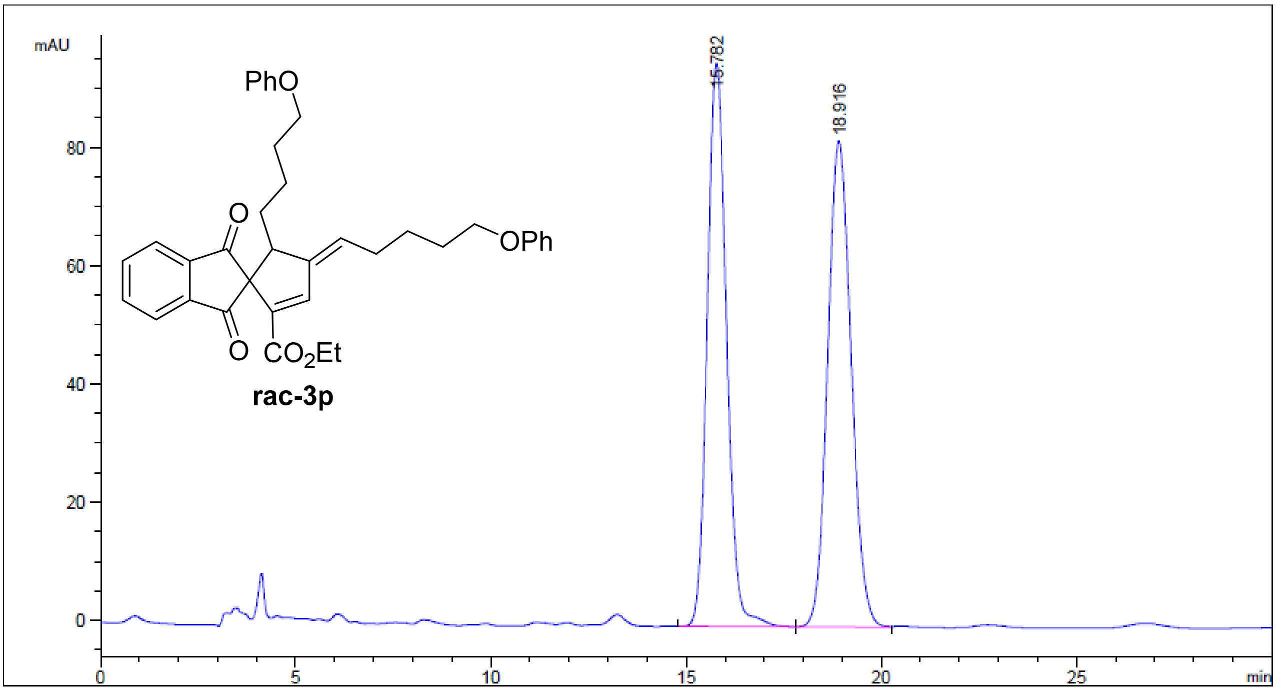

Peak #	RetTime [min]	Type	Width [min]	Area mAU *s	Height [mAU]	Area %
1	15.782	BB	0.5351	3273.24561	95.18669	48.7493
2	18.916	BV	0.6511	3441.20483	82.26089	51.2507

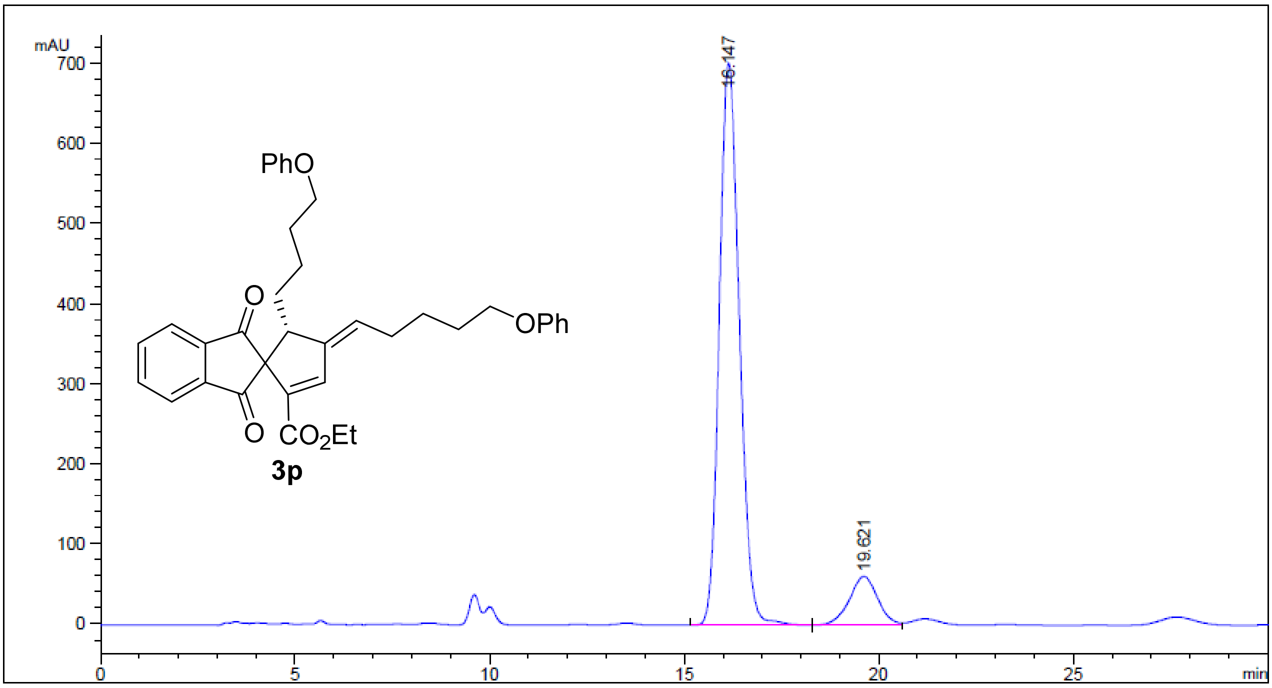

Peak #	RetTime [min]	Type	Width [min]	Area mAU *s	Height [mAU]	Area %
1	16.147	BV	0.5571	2.50594e4	700.49652	89.1005
2	19.621	VV	0.7717	3065.46997	60.49807	10.8995

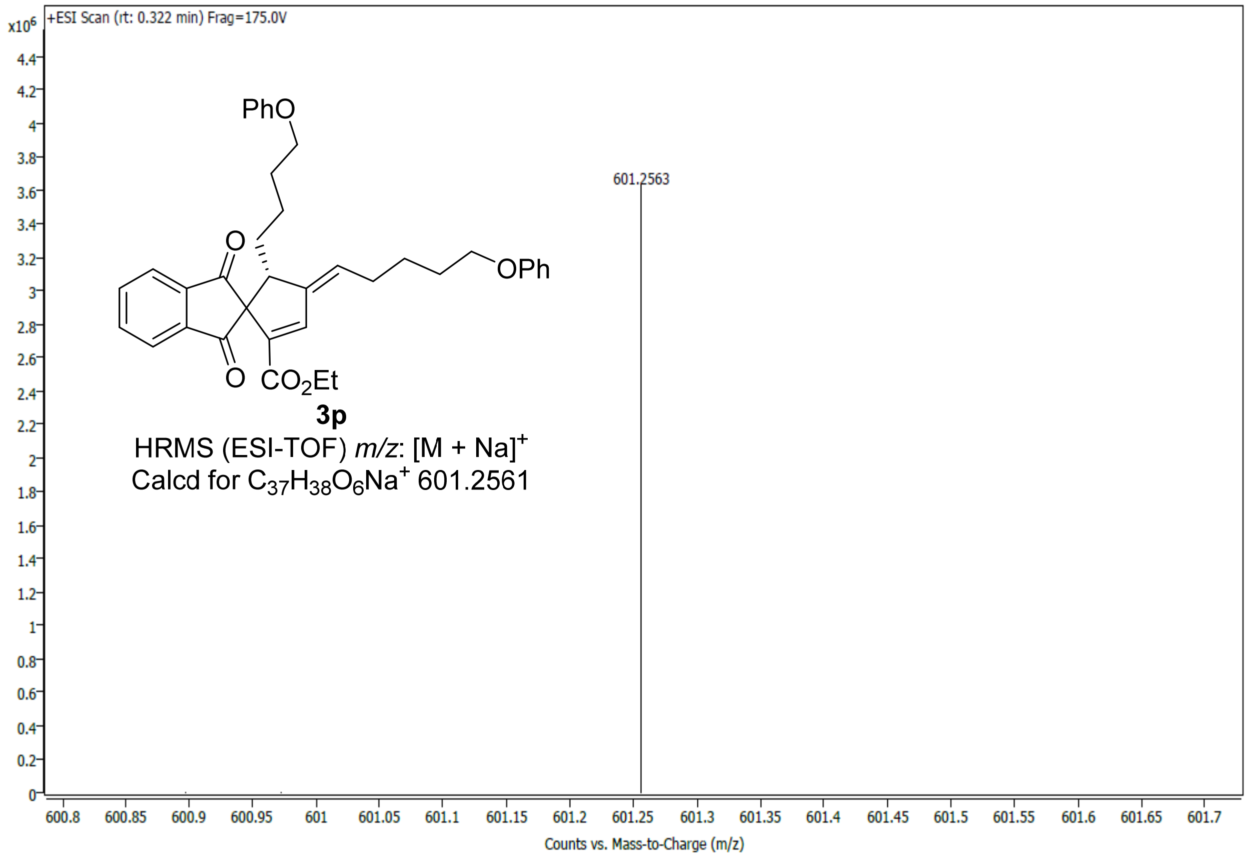
+ESI Scan (rt: 0.322 min) Frag=175.0V
x10^6
601.2563
PhO
OPh
O
O
CO2Et
3p
HRMS (ESI-TOF) m/z: [M + Na]+
Calcd for C37H38O6Na+ 601.2561
Counts vs. Mass-to-Charge (m/z)

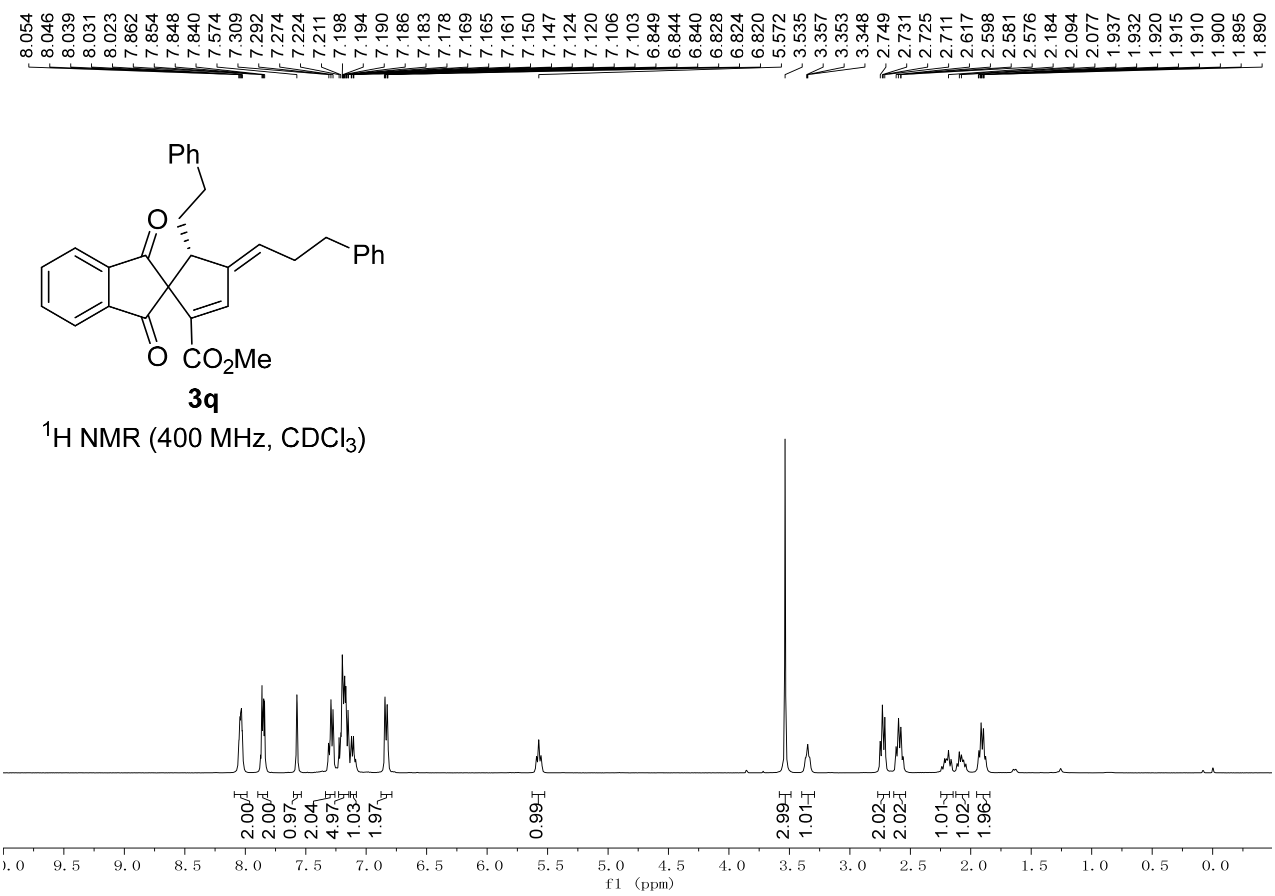

3q
^1H NMR (400 MHz, $CDCl_3$)

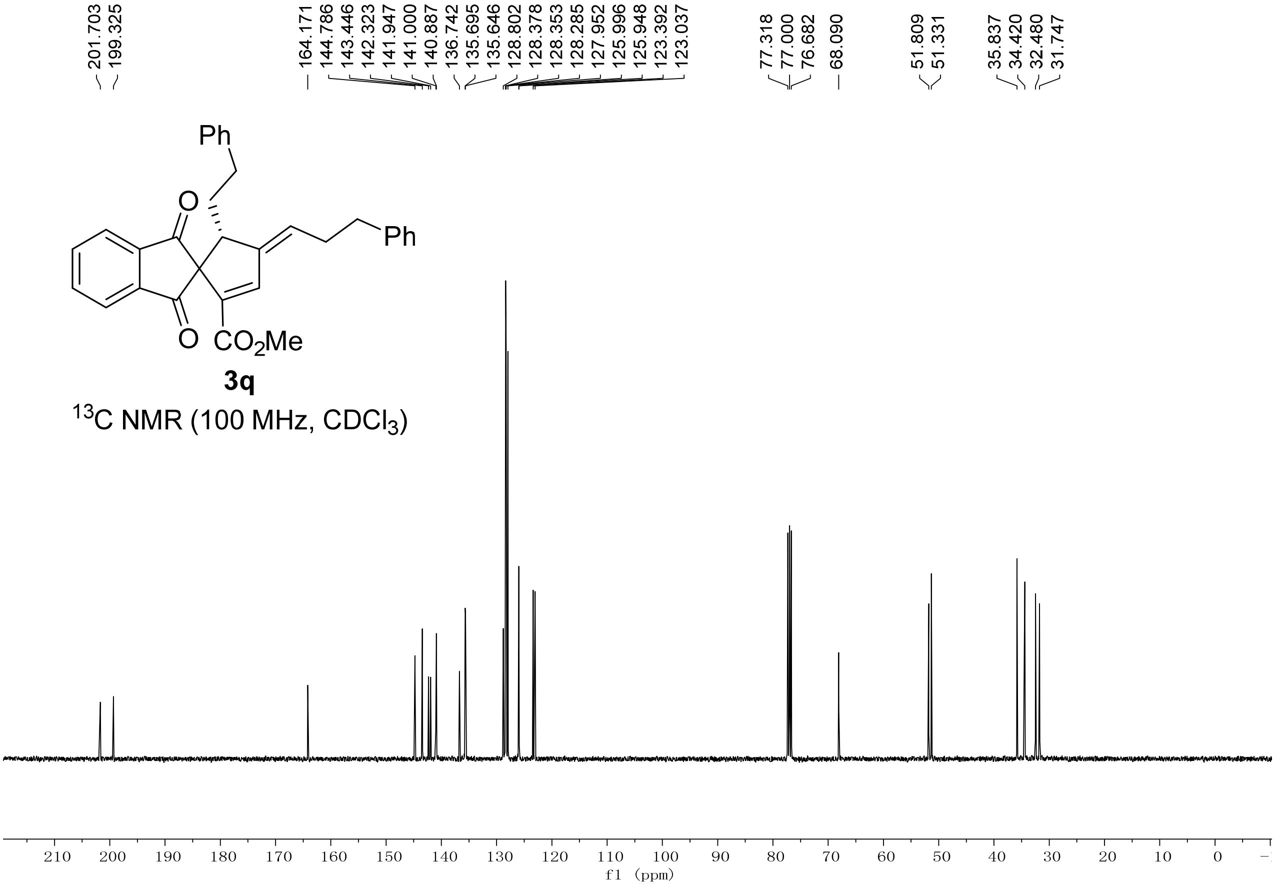

3q
^{13}C NMR (100 MHz, $CDCl_3$)

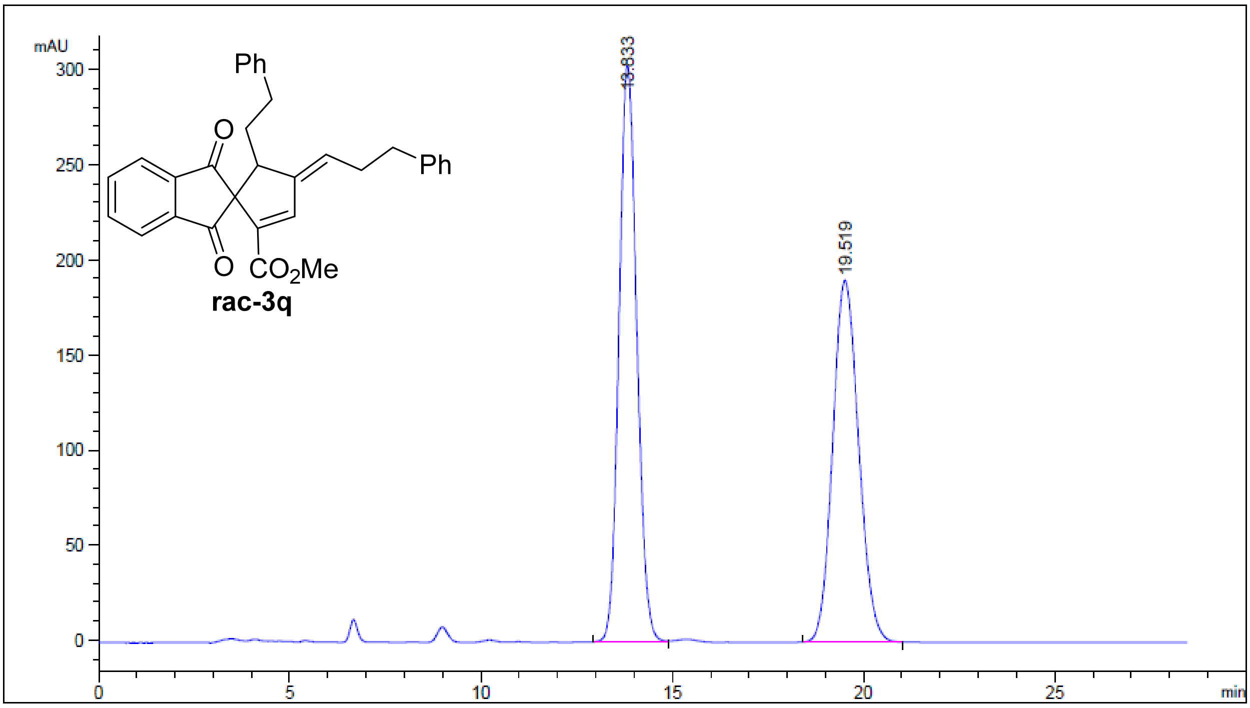

Peak #	RetTime [min]	Type	Width [min]	Area mAU *s	Height [mAU]	Area %
1	13.833	BV	0.5056	9801.62305	302.97610	52.2066
2	19.519	BBA	0.7368	8973.06836	190.17711	47.7934

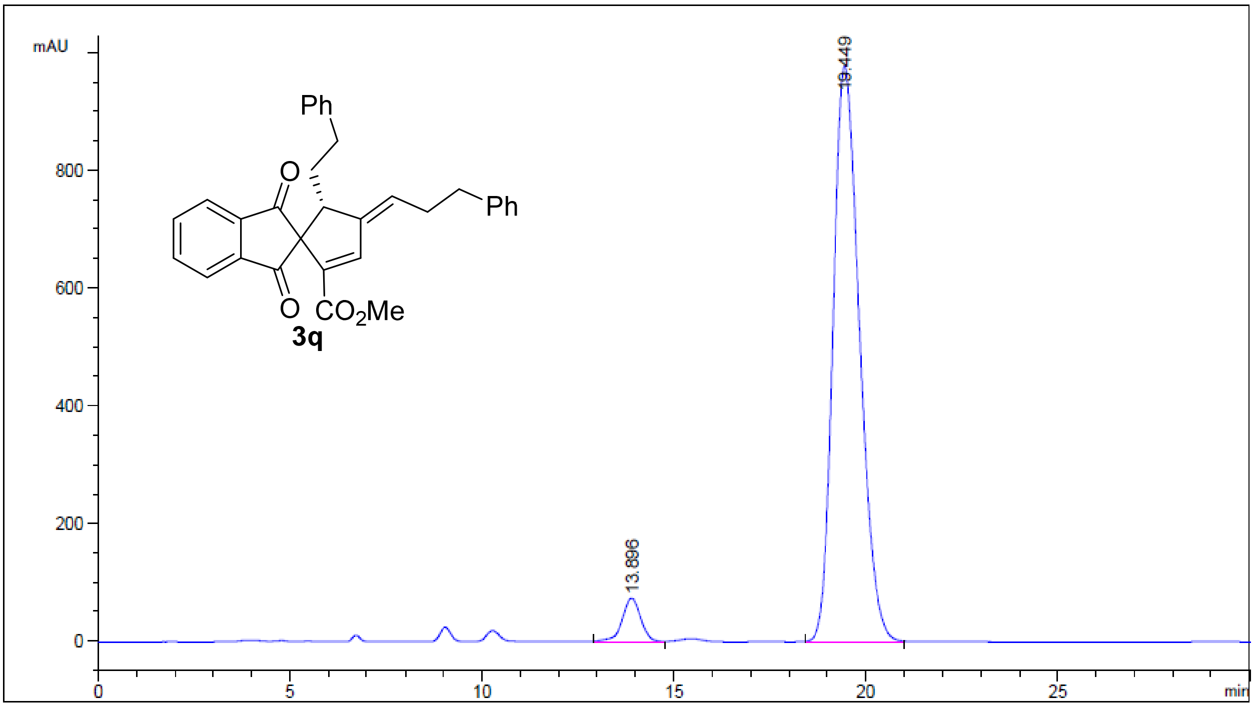

Peak #	RetTime [min]	Type	Width [min]	Area mAU *s	Height [mAU]	Area %
1	13.896	BV	0.5183	2445.00391	73.10286	4.9775
2	19.449	BBA	0.7427	4.66759e4	978.77423	95.0225

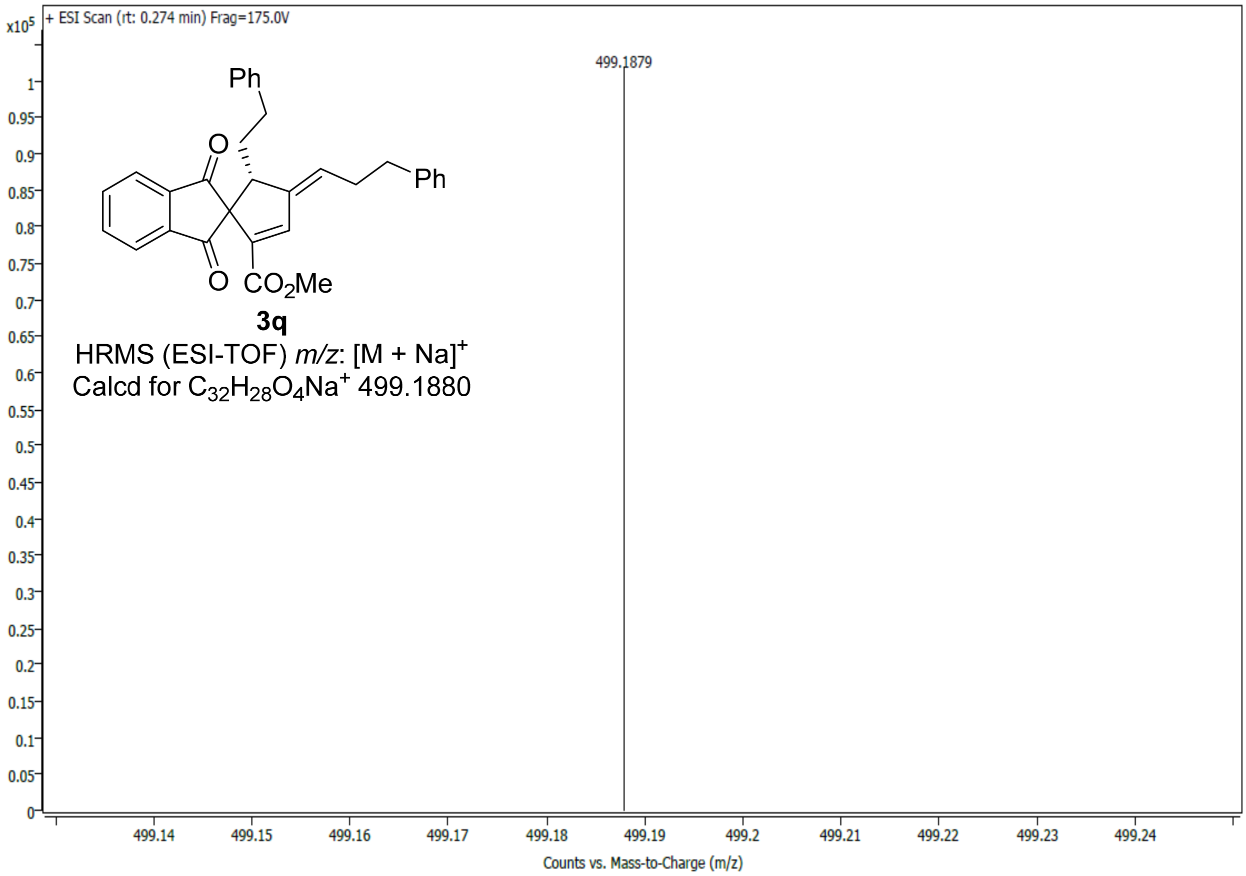
x10^5
+ ESI Scan (rt: 0.274 min) Frag=175.0V
499.1879
Ph
O
Ph
O
CO2Me
3q
HRMS (ESI-TOF) m/z: [M + Na]+
Calcd for C32H28O4Na+ 499.1880
1
0.95
0.9
0.85
0.8
0.75
0.7
0.65
0.6
0.55
0.5
0.45
0.4
0.35
0.3
0.25
0.2
0.15
0.1
0.05
0
499.14
499.15
499.16
499.17
499.18
499.19
499.2
499.21
499.22
499.23
499.24
Counts vs. Mass-to-Charge (m/z)

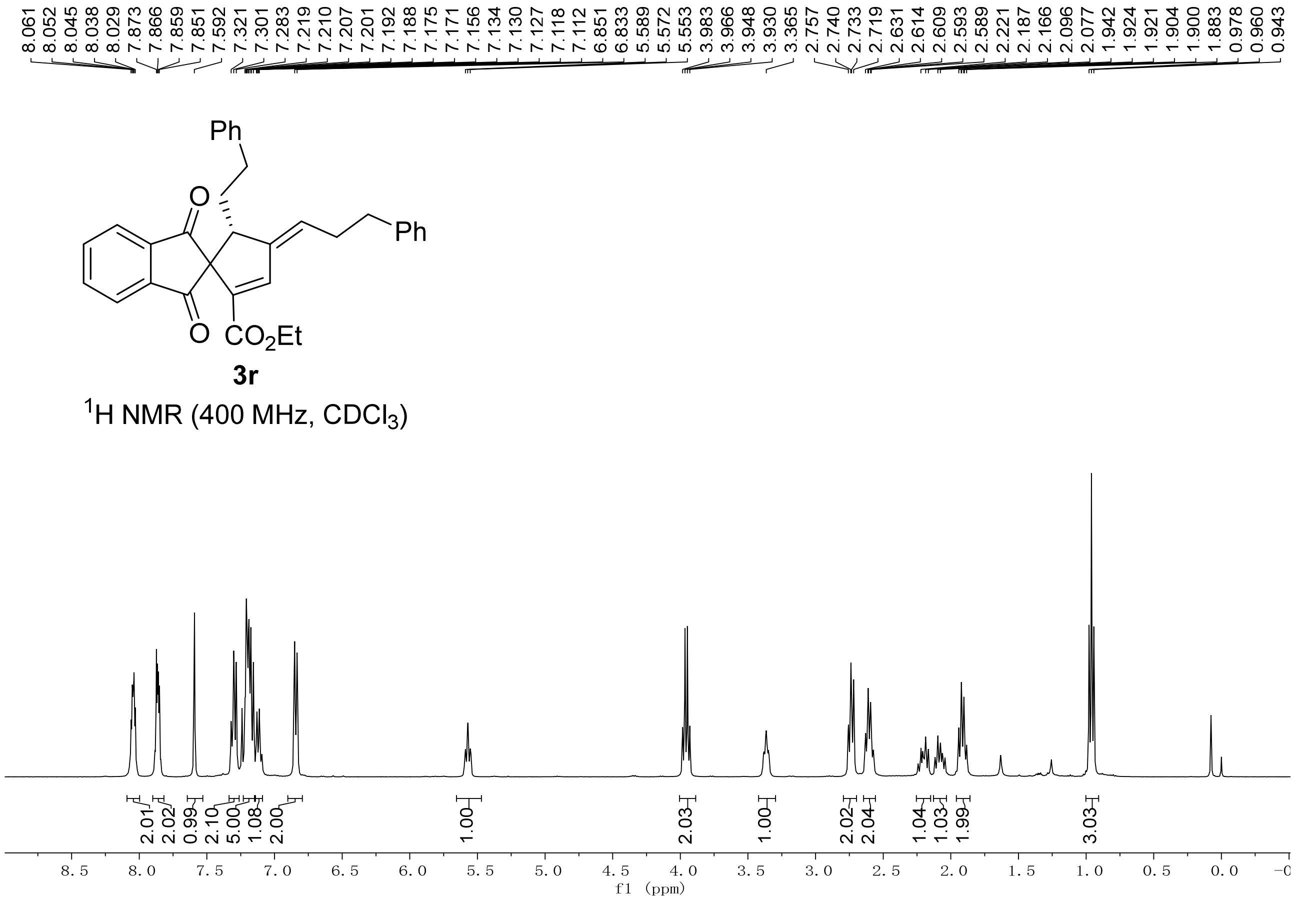

3r

^{1}H NMR (400 MHz, $CDCl_3$)

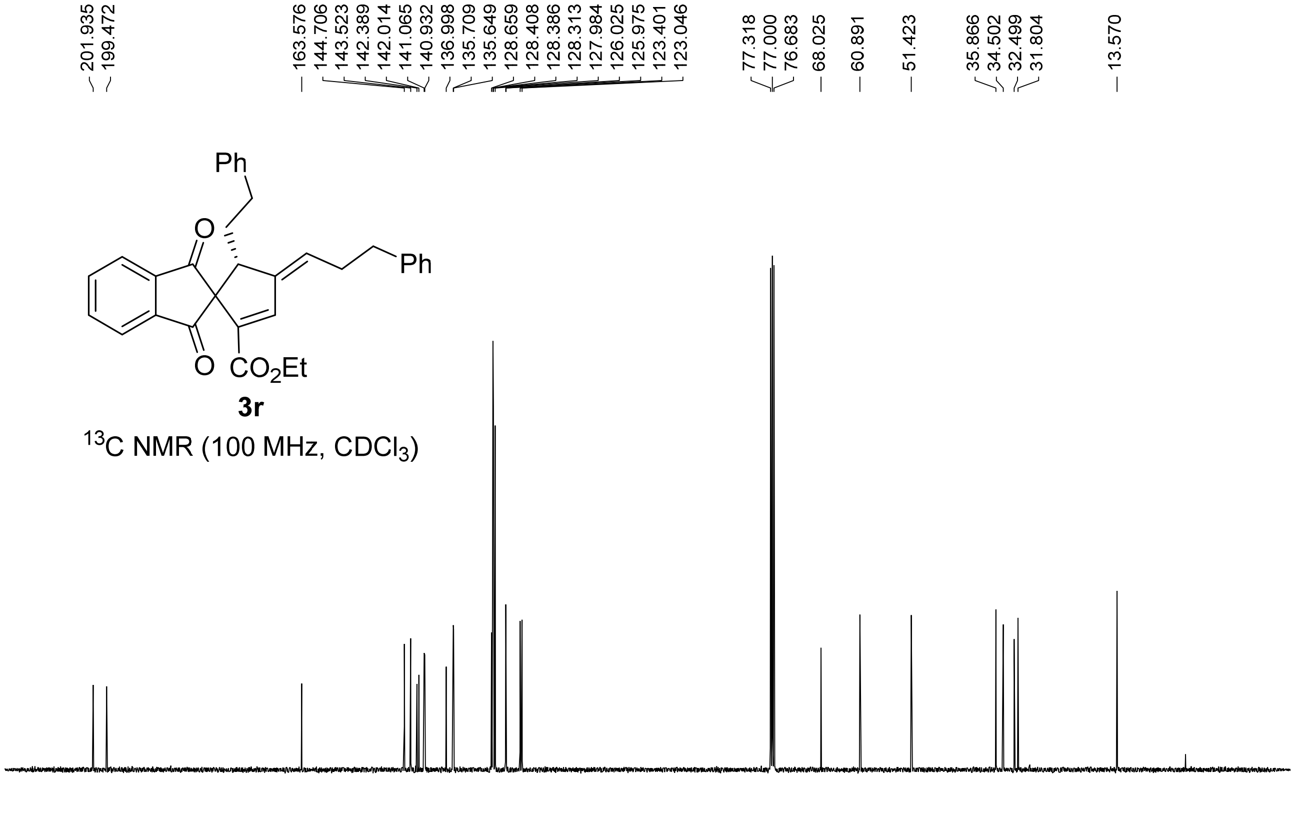

3r

^{13}C NMR (100 MHz, $CDCl_3$)

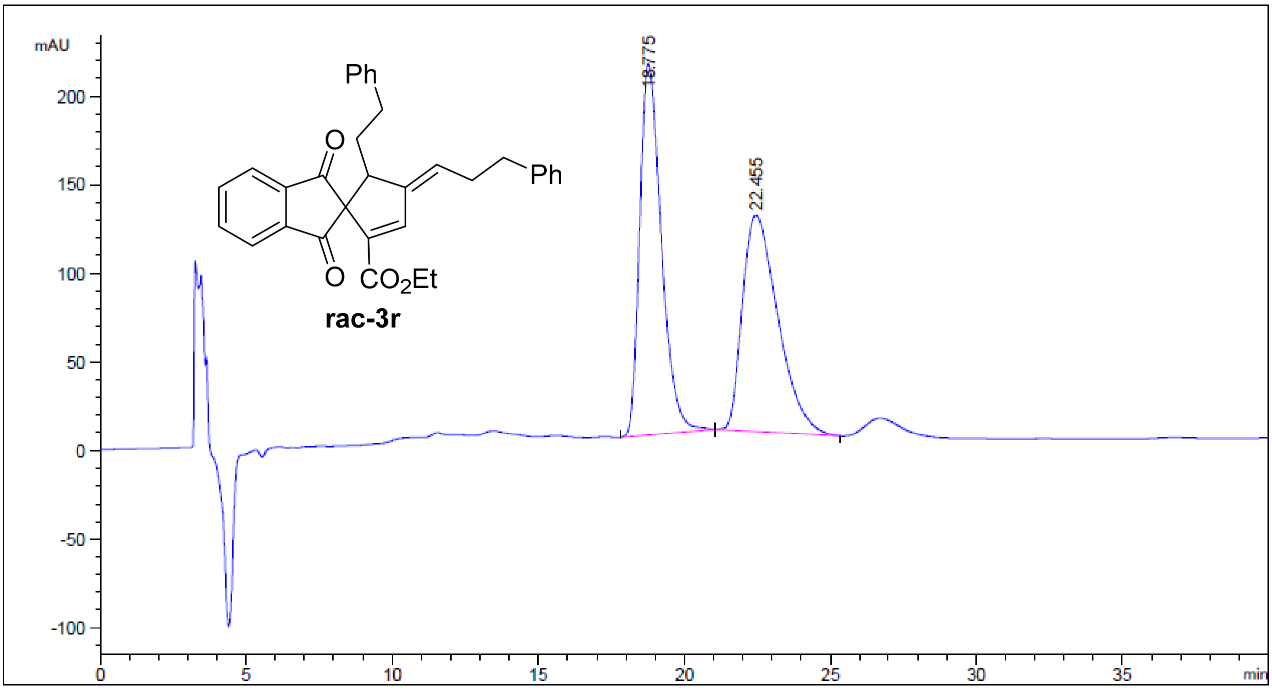

Peak #	RetTime [min]	Type	Width [min]	Area mAU *s	Height [mAU]	Area %
1	18.775	BB	0.7937	1.07691e4	209.36830	50.9505
2	22.455	BB	1.3061	1.03673e4	122.15281	49.0495

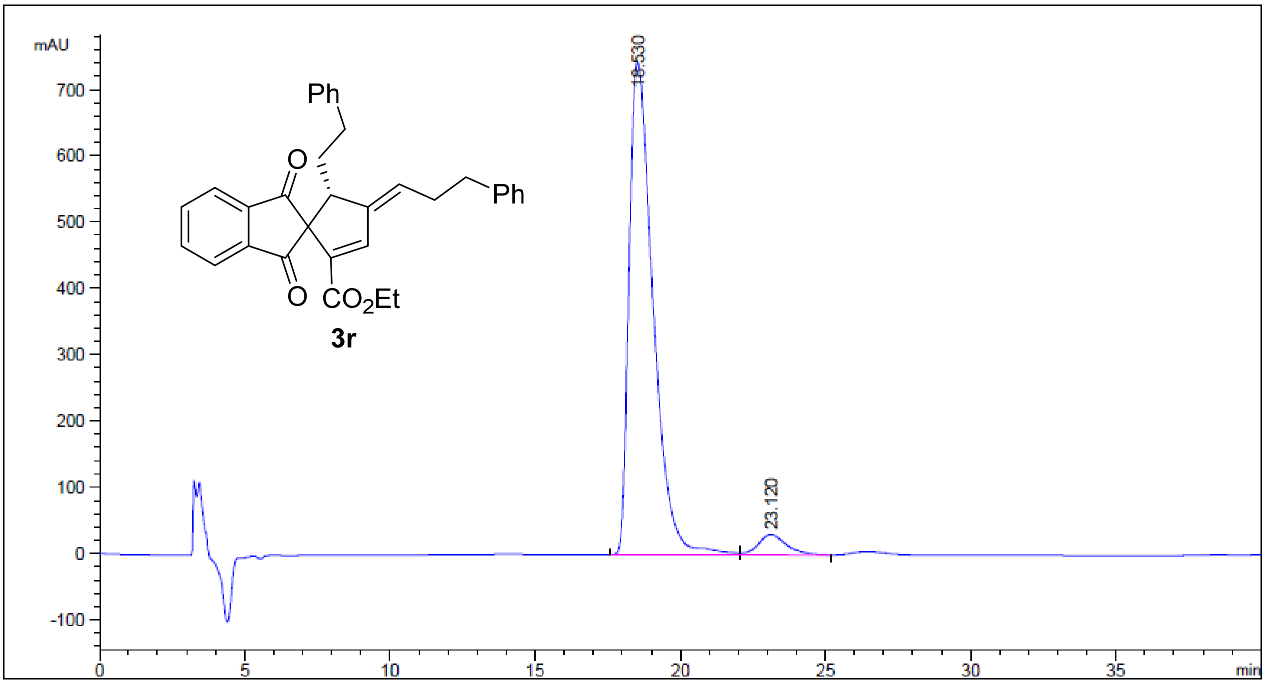

Peak #	RetTime [min]	Type	Width [min]	Area mAU *s	Height [mAU]	Area %
1	18.530	BV	0.8871	4.27164e4	742.07837	95.4120
2	23.120	VB	0.9841	2054.06958	30.71339	4.5880

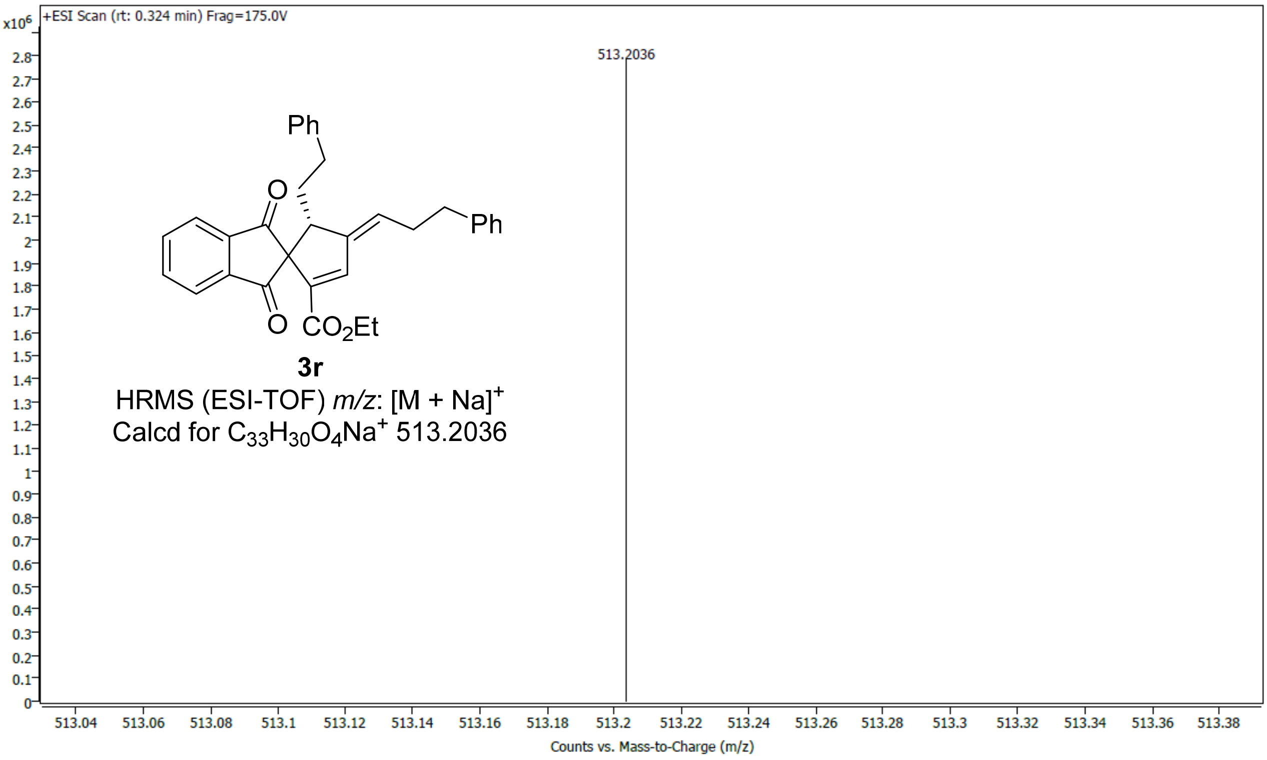

x10^6
+ESI Scan (rt: 0.324 min) Frag=175.0V
513.2036
Ph
O
Ph
O
CO2Et
3r
HRMS (ESI-TOF) m/z: [M + Na]+
Calcd for C33H30O4Na+ 513.2036
Counts vs. Mass-to-Charge (m/z)

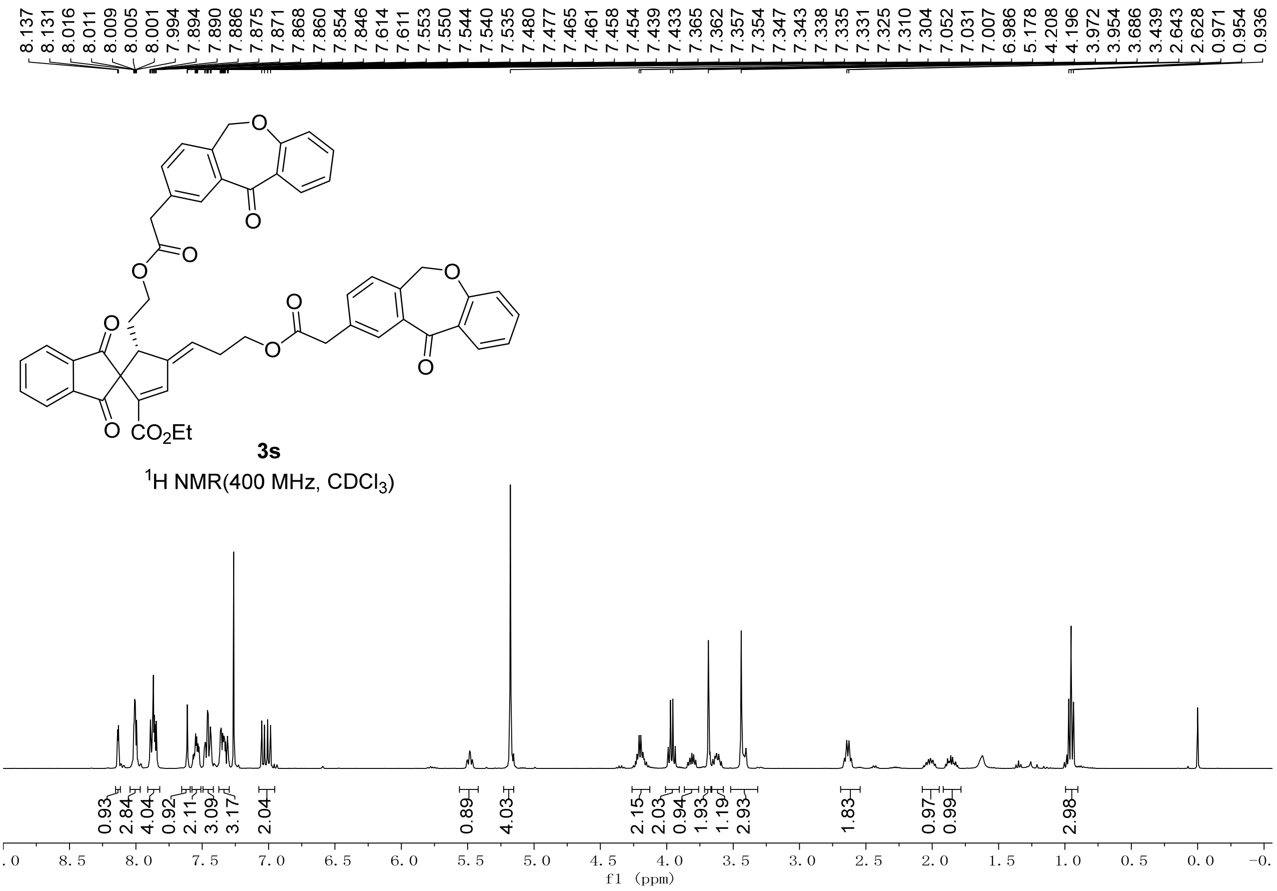
8.137 8.131 8.016 8.011 8.009 8.005 8.001 7.994 7.894 7.890 7.886 7.875 7.871 7.868 7.860 7.854 7.846 7.614 7.611 7.553 7.550 7.544 7.540 7.535 7.480 7.477 7.465 7.461 7.458 7.454 7.439 7.433 7.365 7.362 7.357 7.354 7.347 7.343 7.338 7.335 7.331 7.325 7.310 7.304 7.052 7.031 7.007 6.986 5.178 4.208 4.196 3.972 3.954 3.686 3.439 2.643 2.628 0.971 0.954 0.936
O
O
O
O
O
O
O
O
O
O
CO2Et
3s
1H NMR(400 MHz, CDCl3)
0.93 2.84 4.04 0.92 2.11 3.09 3.17 2.04 0.89 4.03 2.15 2.03 0.94 1.93 1.19 2.93 1.83 0.97 0.99 2.98
f1 (ppm)

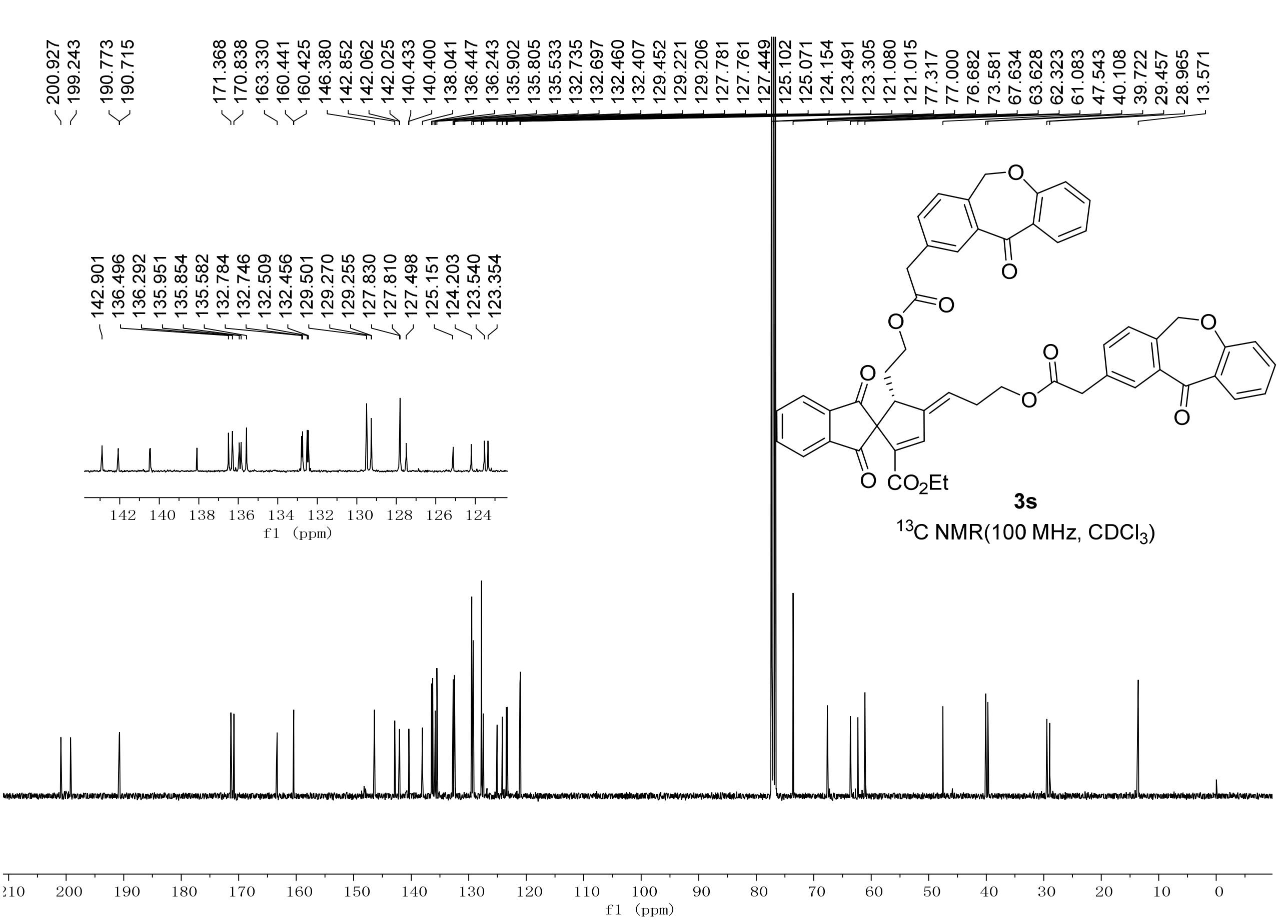
200.927 199.243 190.773 190.715 171.368 170.838 163.330 160.441 160.425 146.380 142.852 142.062 142.025 140.433 140.400 138.041 136.447 136.243 135.902 135.805 135.533 132.735 132.697 132.460 132.407 129.452 129.221 129.206 127.781 127.761 127.449 125.102 125.071 124.154 123.491 123.305 121.080 121.015 77.317 77.000 76.682 73.581 67.634 63.628 62.323 61.083 47.543 40.108 39.722 29.457 28.965 13.571
142.901 136.496 136.292 135.951 135.854 135.582 132.784 132.746 132.509 132.456 129.501 129.270 129.255 127.830 127.810 127.498 125.151 124.203 123.540 123.354
f1 (ppm)
CO2Et
3s
13C NMR(100 MHz, CDCl3)
f1 (ppm)

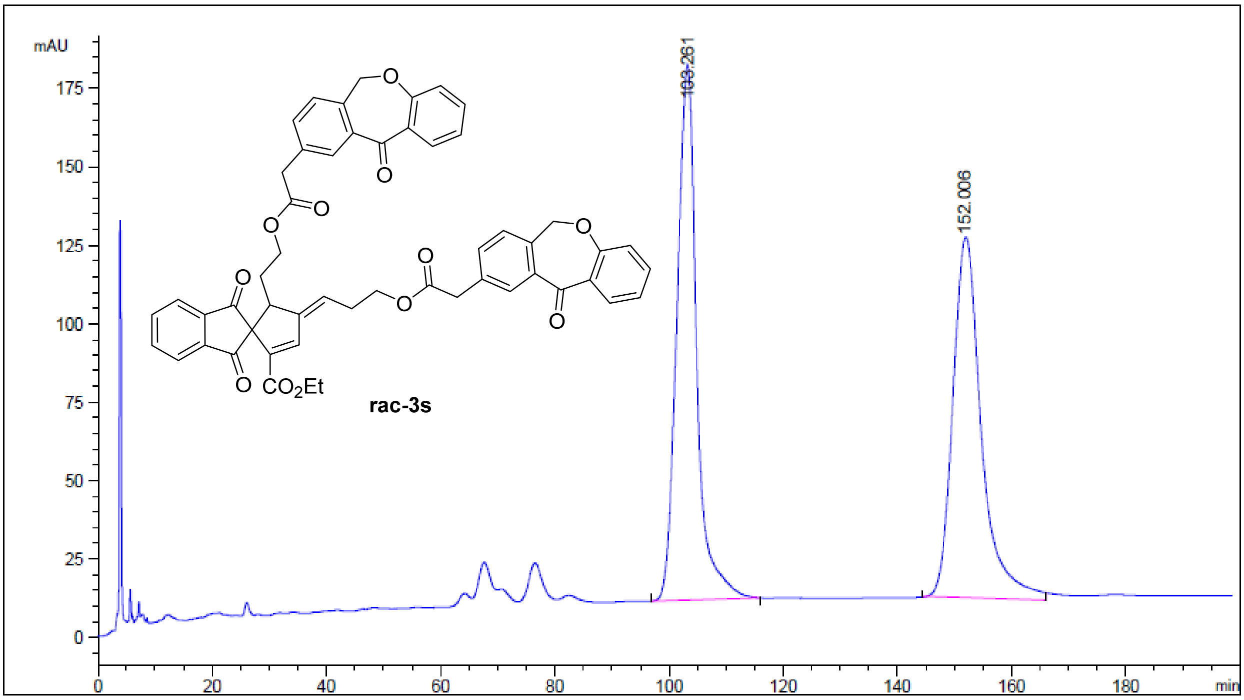

Peak #	RetTime [min]	Type	Width [min]	Area mAU *s	Height [mAU]	Area %
1	103.261	BBA	3.3732	4.10393e4	170.75708	50.6746
2	152.006	MM	5.7930	3.99467e4	114.92797	49.3254

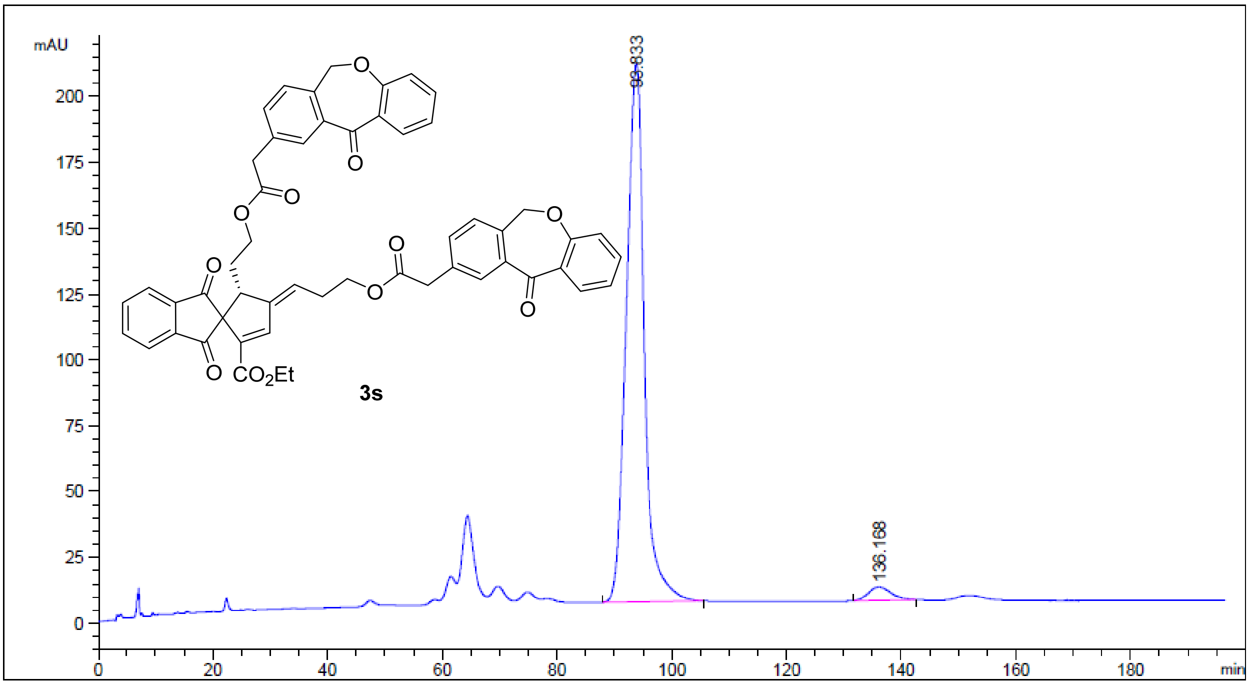

Peak #	RetTime [min]	Type	Width [min]	Area mAU *s	Height [mAU]	Area %
1	93.833	BB	3.1130	4.45147e4	204.39645	97.0808
2	136.168	BB	3.1587	1338.54980	4.95760	2.9192

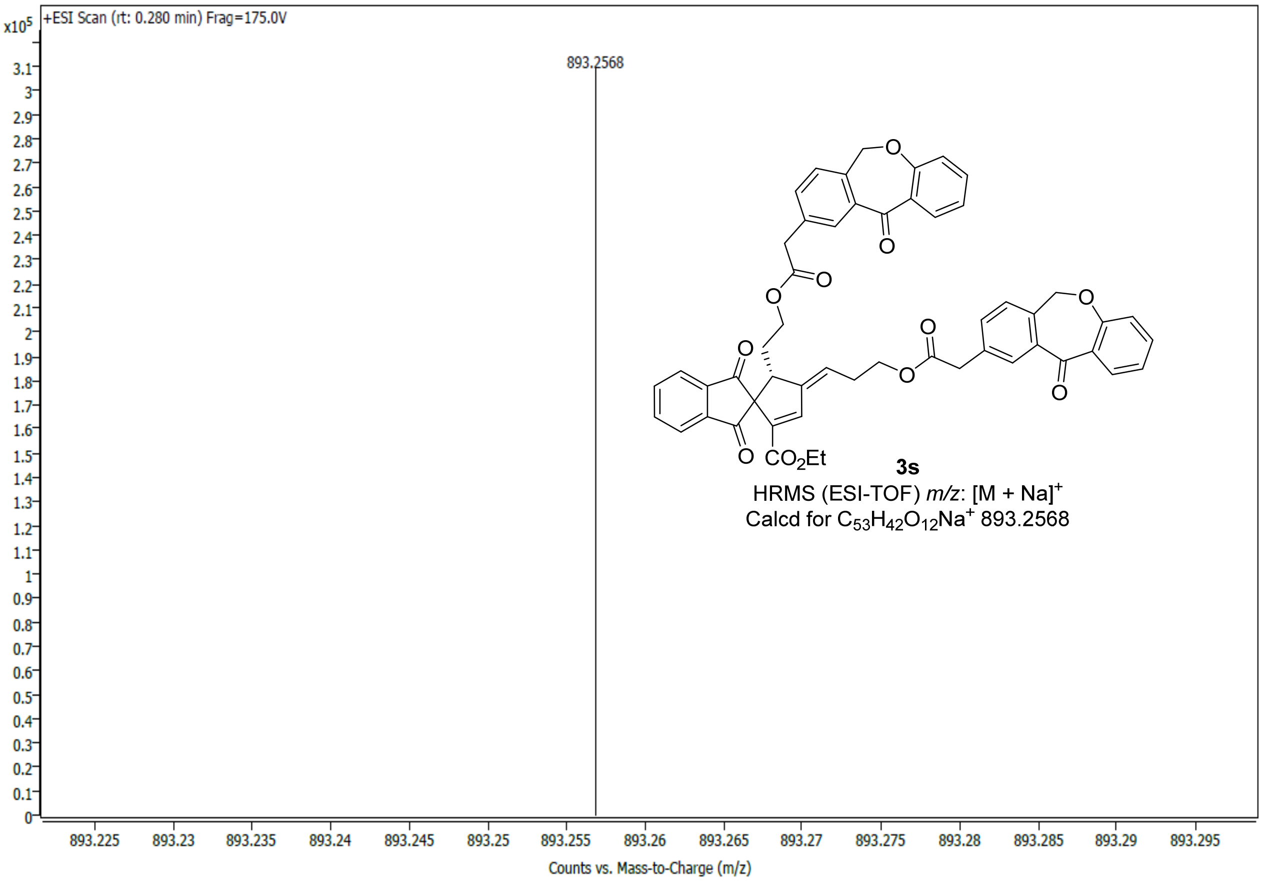

3s

HRMS (ESI-TOF) *m/z*: $[M + Na]^+$

Calcd for $C_{53}H_{42}O_{12}Na^+$ 893.2568

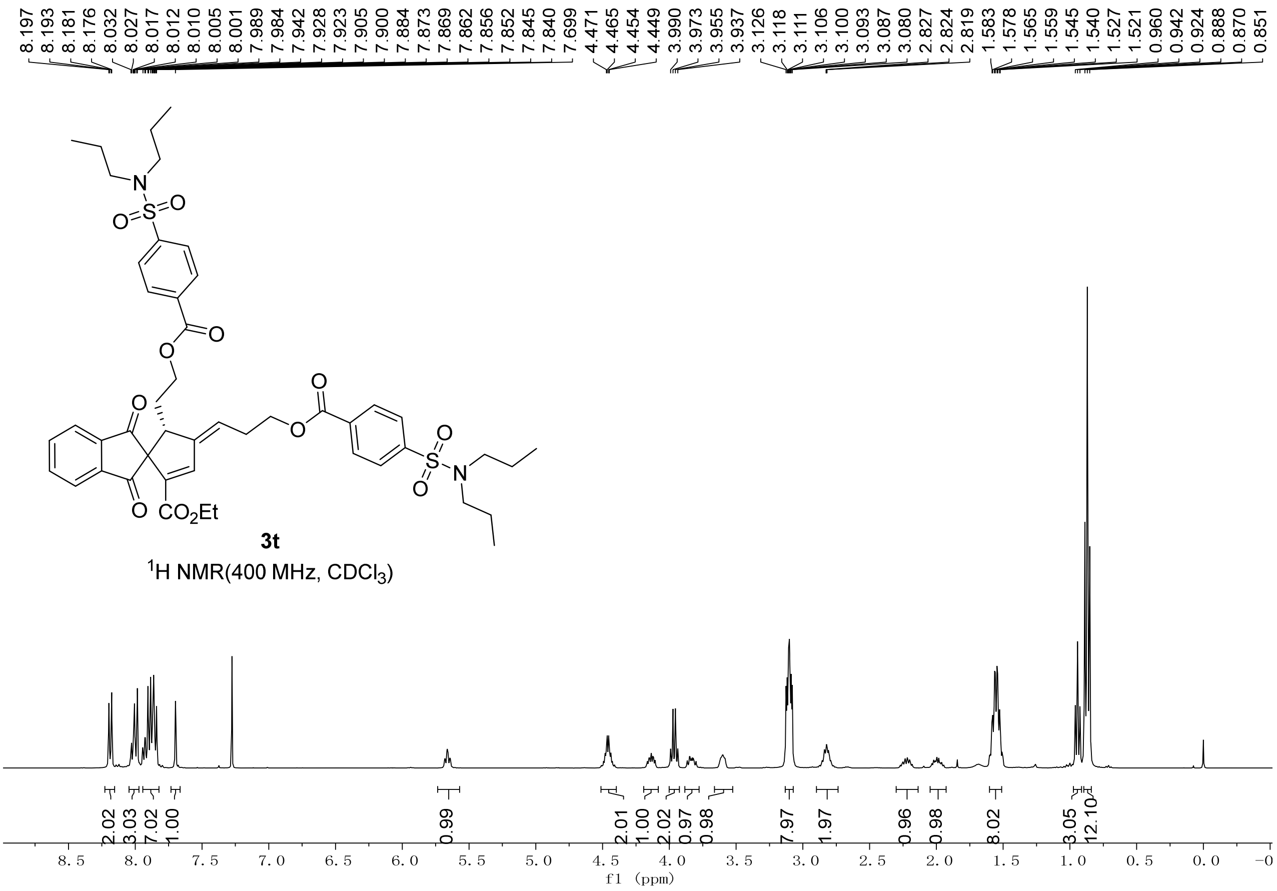
8.197 8.193 8.181 8.176 8.032 8.027 8.017 8.012 8.010 8.005 8.001 7.989 7.984 7.942 7.928 7.923 7.905 7.900 7.884 7.873 7.869 7.862 7.856 7.852 7.845 7.840 7.699 4.471 4.465 4.454 4.449 3.990 3.973 3.955 3.937 3.126 3.118 3.111 3.106 3.100 3.093 3.087 3.080 2.827 2.824 2.819 1.583 1.578 1.565 1.559 1.545 1.540 1.527 1.521 0.960 0.942 0.924 0.888 0.870 0.851
3t
^{1}H NMR(400 MHz, CDCl$_3$)
2.02 3.03 7.02 1.00 0.99 2.01 1.00 2.02 0.97 0.98 7.97 1.97 0.96 0.98 8.02 3.05 12.10
f1 (ppm)

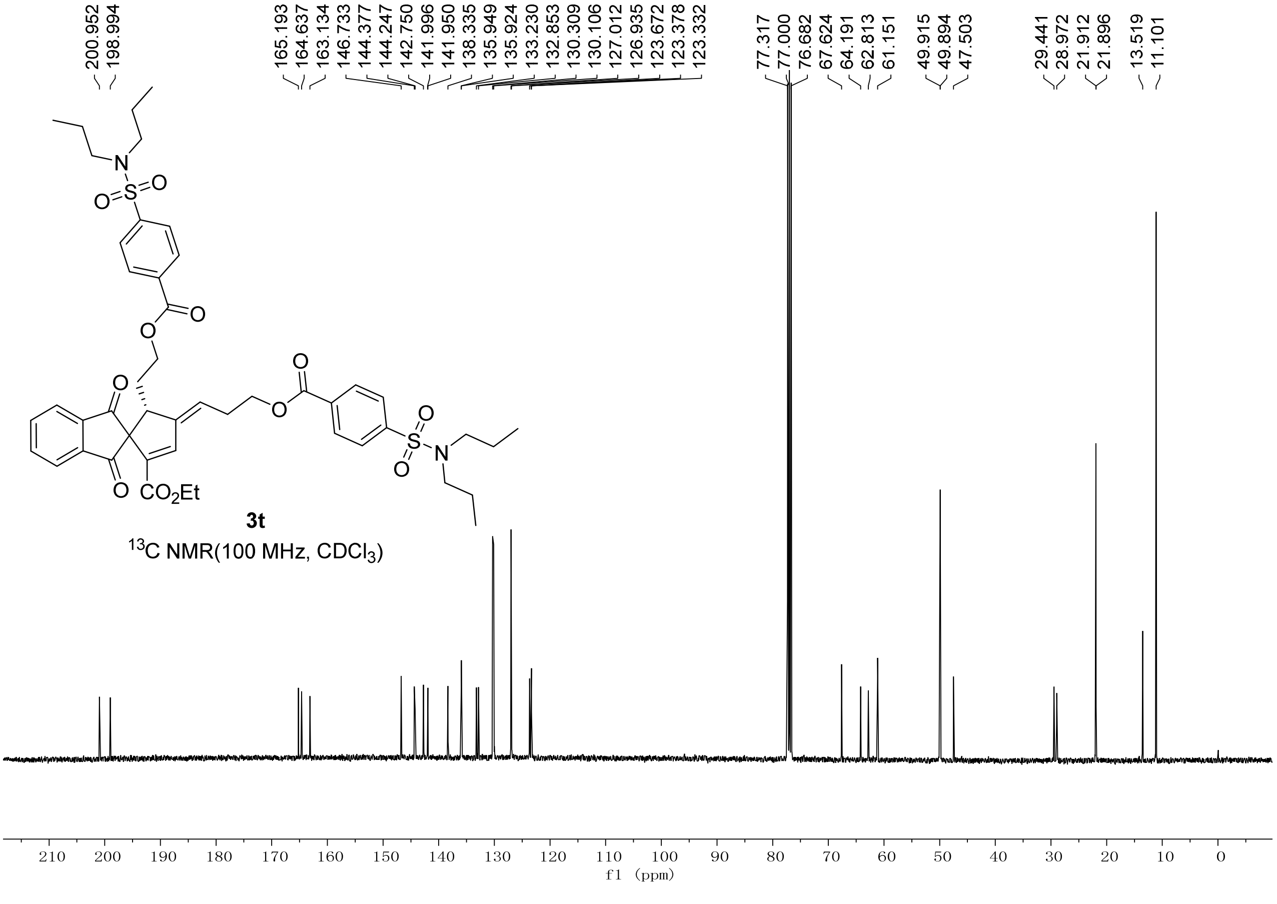
200.952 198.994 165.193 164.637 163.134 146.733 144.377 144.247 142.750 141.996 141.950 138.335 135.949 135.924 133.230 132.853 130.309 130.106 127.012 126.935 123.672 123.378 123.332 77.317 77.000 76.682 67.624 64.191 62.813 61.151 49.915 49.894 47.503 29.441 28.972 21.912 21.896 13.519 11.101
3t
^{13}C NMR(100 MHz, CDCl$_3$)
f1 (ppm)

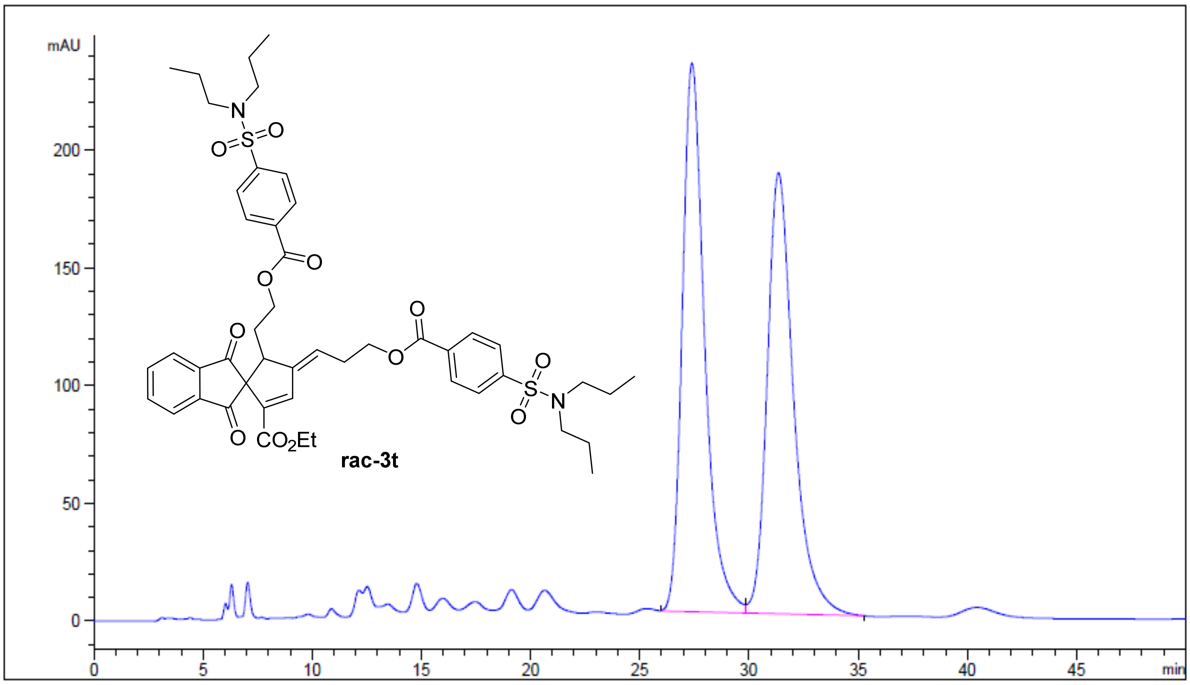

Peak #	RetTime [min]	Type	Width [min]	Area mAU *s	Area %
1	27.397	BB	1.0816	1.65737e4	51.2664
2	31.359	BB	1.2654	1.57549e4	48.7336

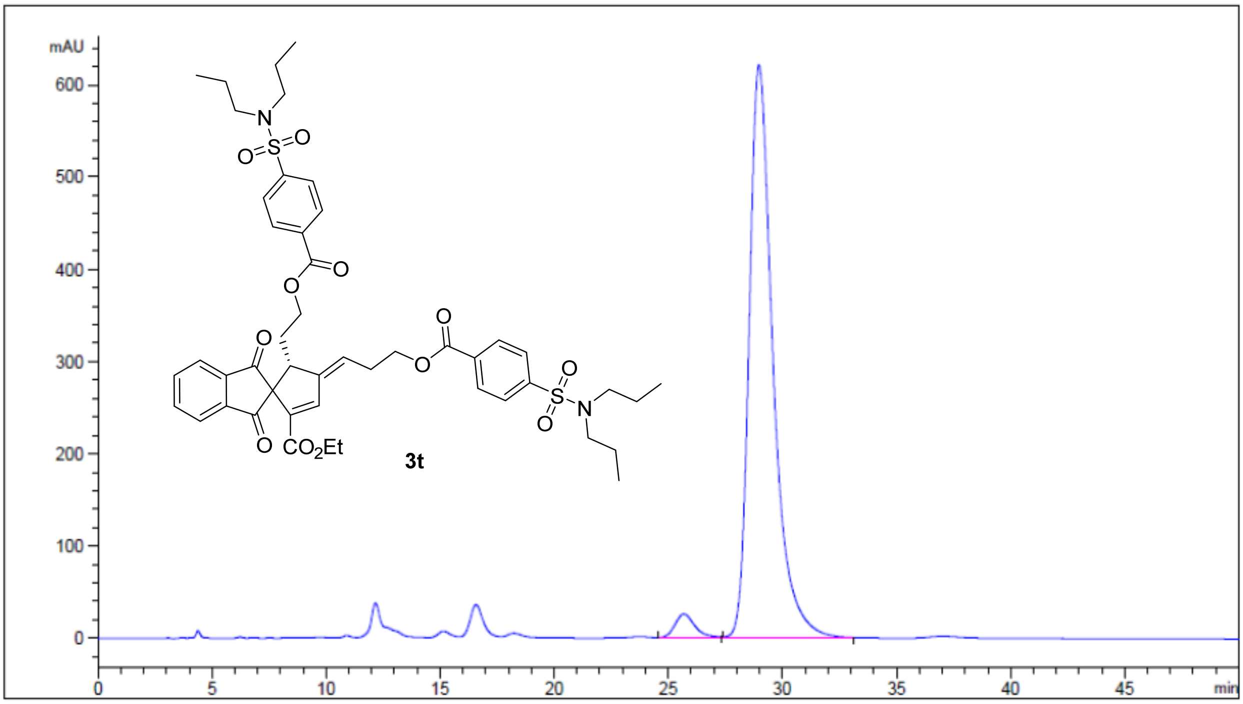

Peak #	RetTime [min]	Type	Width [min]	Area mAU *s	Area %
1	25.685	BB	0.9313	1563.01318	3.3563
2	28.980	BB	1.0923	4.50066e4	96.6437

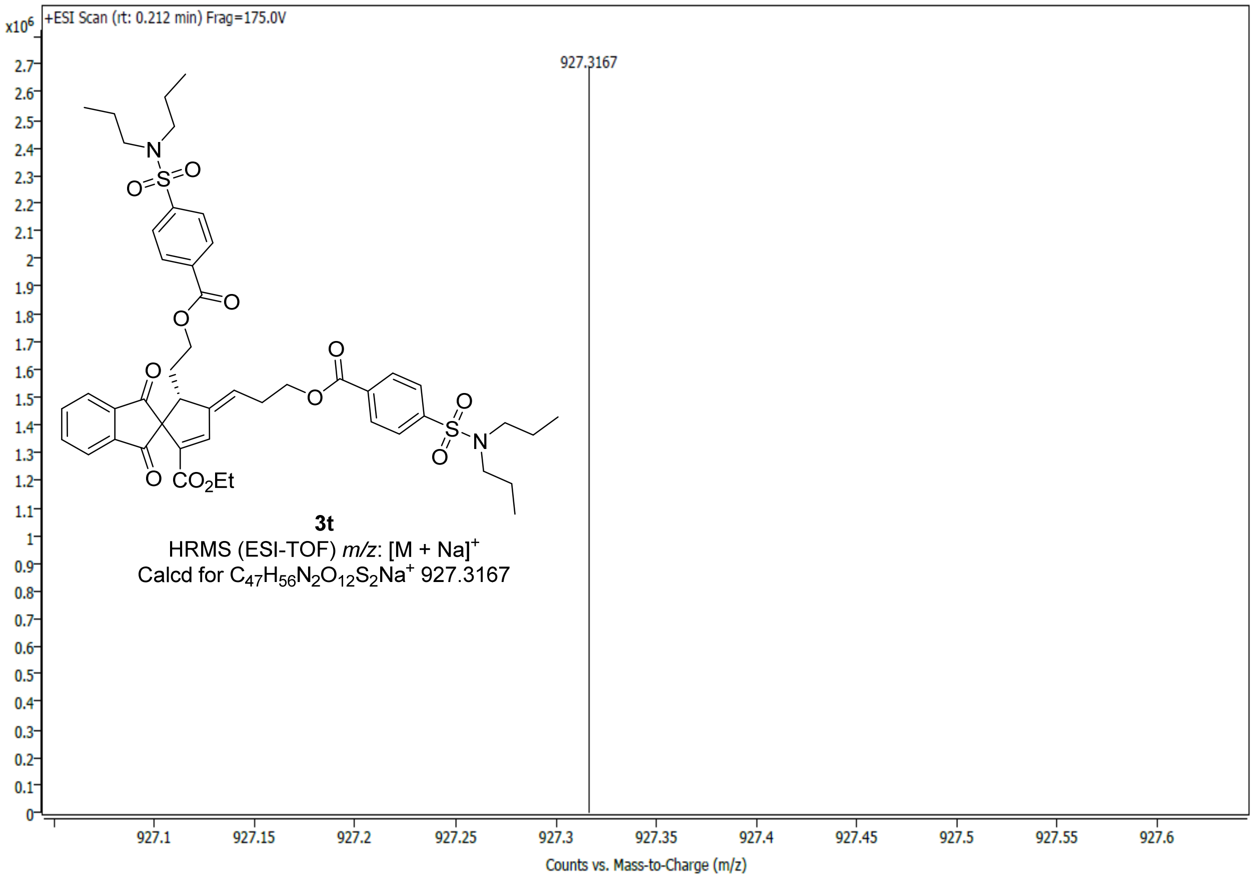

x10^6
+ESI Scan (rt: 0.212 min) Frag=175.0V
927.3167
2.7
2.6
2.5
2.4
2.3
2.2
2.1
2
1.9
1.8
1.7
1.6
1.5
1.4
1.3
1.2
1.1
1
0.9
0.8
0.7
0.6
0.5
0.4
0.3
0.2
0.1
0
927.1
927.15
927.2
927.25
927.3
927.35
927.4
927.45
927.5
927.55
927.6
Counts vs. Mass-to-Charge (m/z)
CO2Et
3t
HRMS (ESI-TOF) m/z: [M + Na]+
Calcd for C47H56N2O12S2Na+ 927.3167

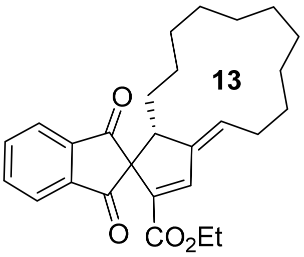

3u

^{1}H NMR (400 MHz, CDCl$_3$)

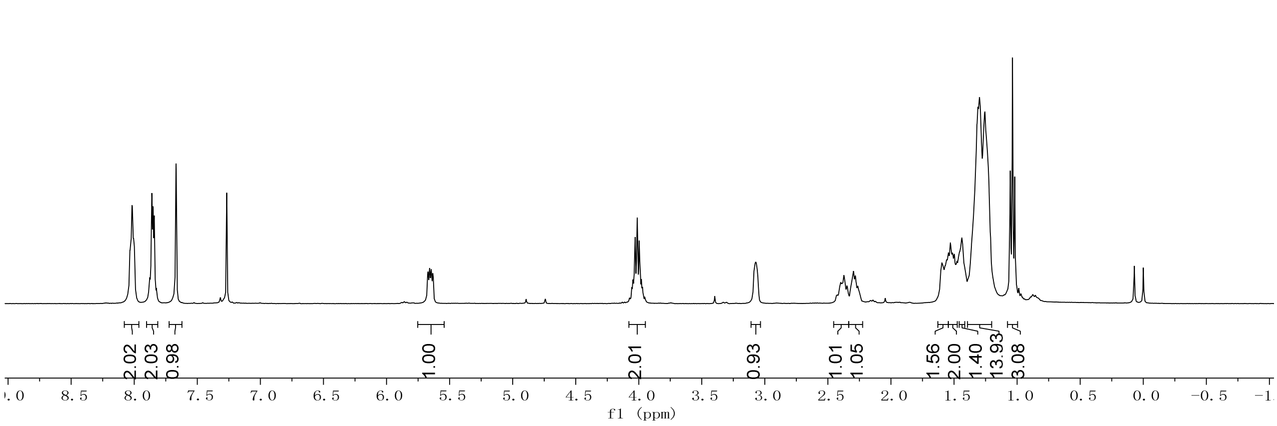

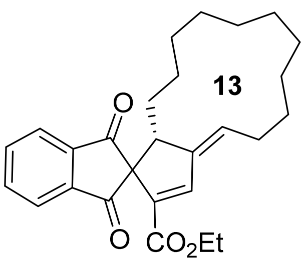

3u

^{13}C NMR (100 MHz, CDCl$_3$)

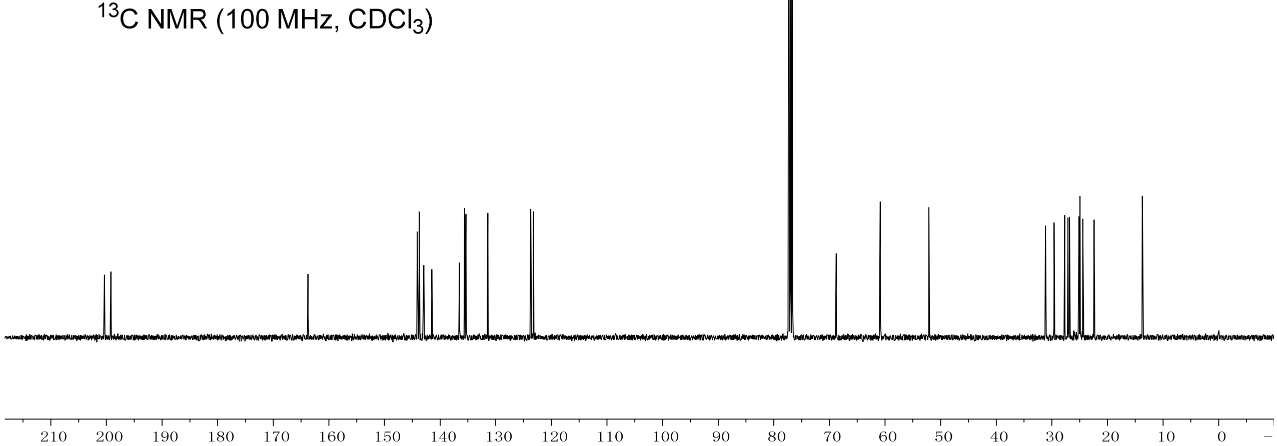

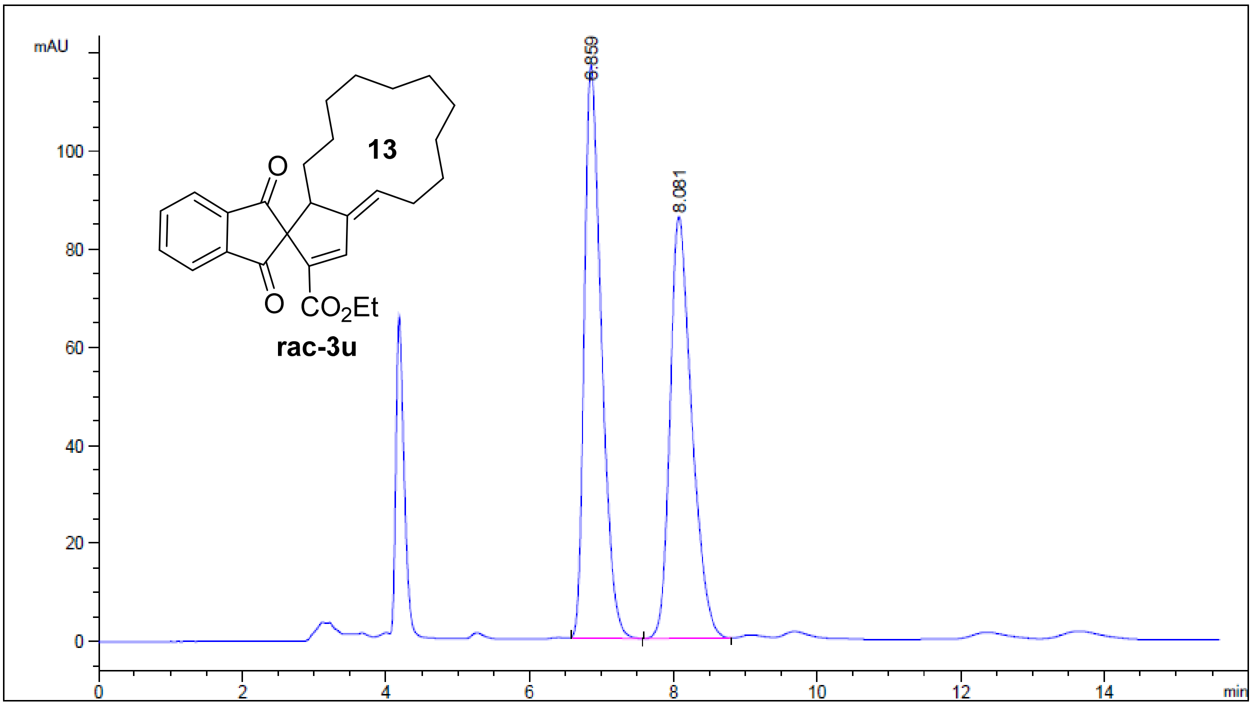

Peak #	RetTime [min]	Type	Width [min]	Area mAU *s	Height [mAU]	Area %
1	6.859	BV	0.2573	1930.27979	116.55369	51.6513
2	8.081	BB	0.3158	1806.85815	85.73760	48.3487

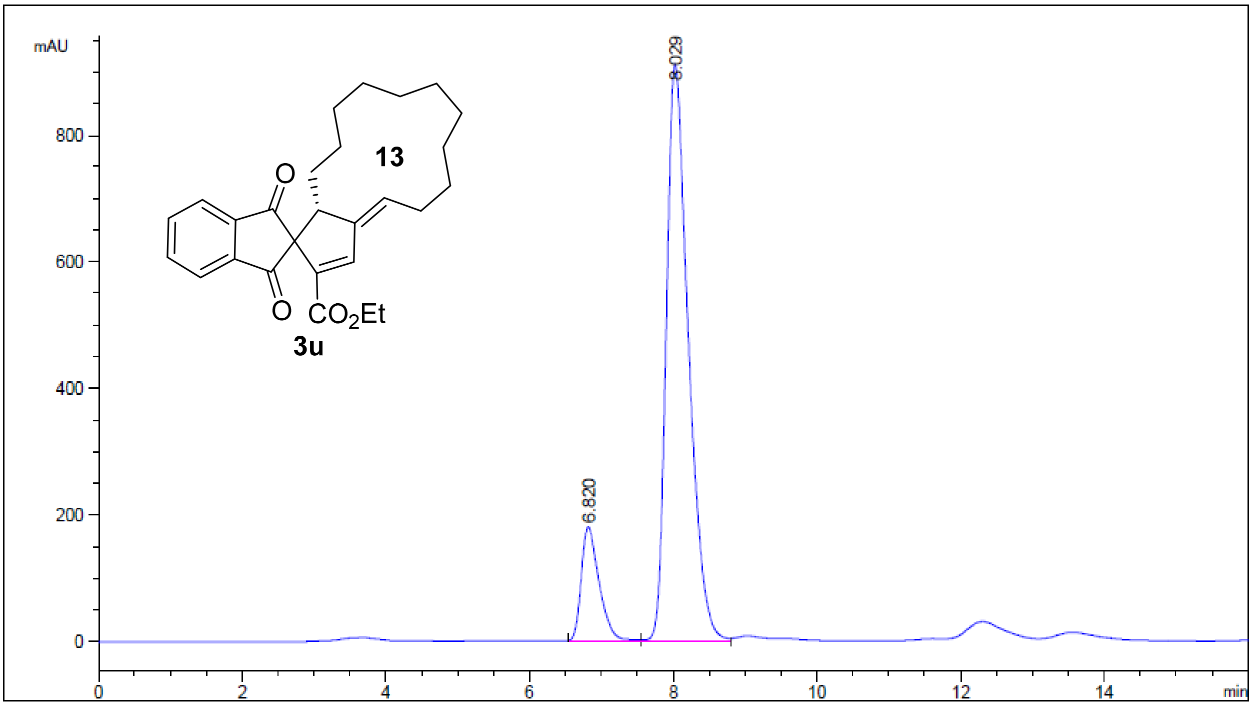

Peak #	RetTime [min]	Type	Width [min]	Area mAU *s	Height [mAU]	Area %
1	6.820	BV	0.2648	3094.39380	179.81638	13.7968
2	8.029	VV	0.3236	1.93340e4	909.76697	86.2032

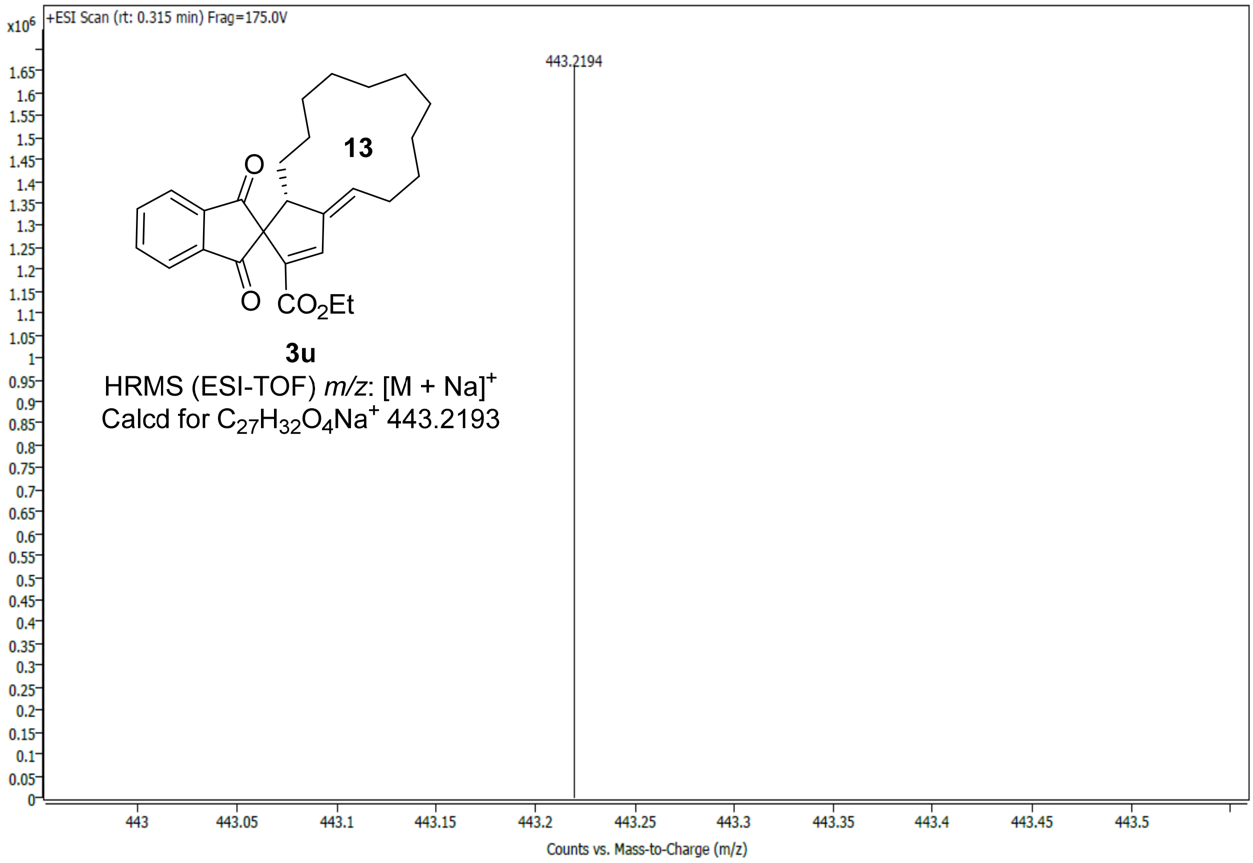

x10^6
+ESI Scan (rt: 0.315 min) Frag=175.0V
443.2194
13
O
O
CO2Et
3u
HRMS (ESI-TOF) m/z: [M + Na]+
Calcd for C27H32O4Na+ 443.2193
Counts vs. Mass-to-Charge (m/z)

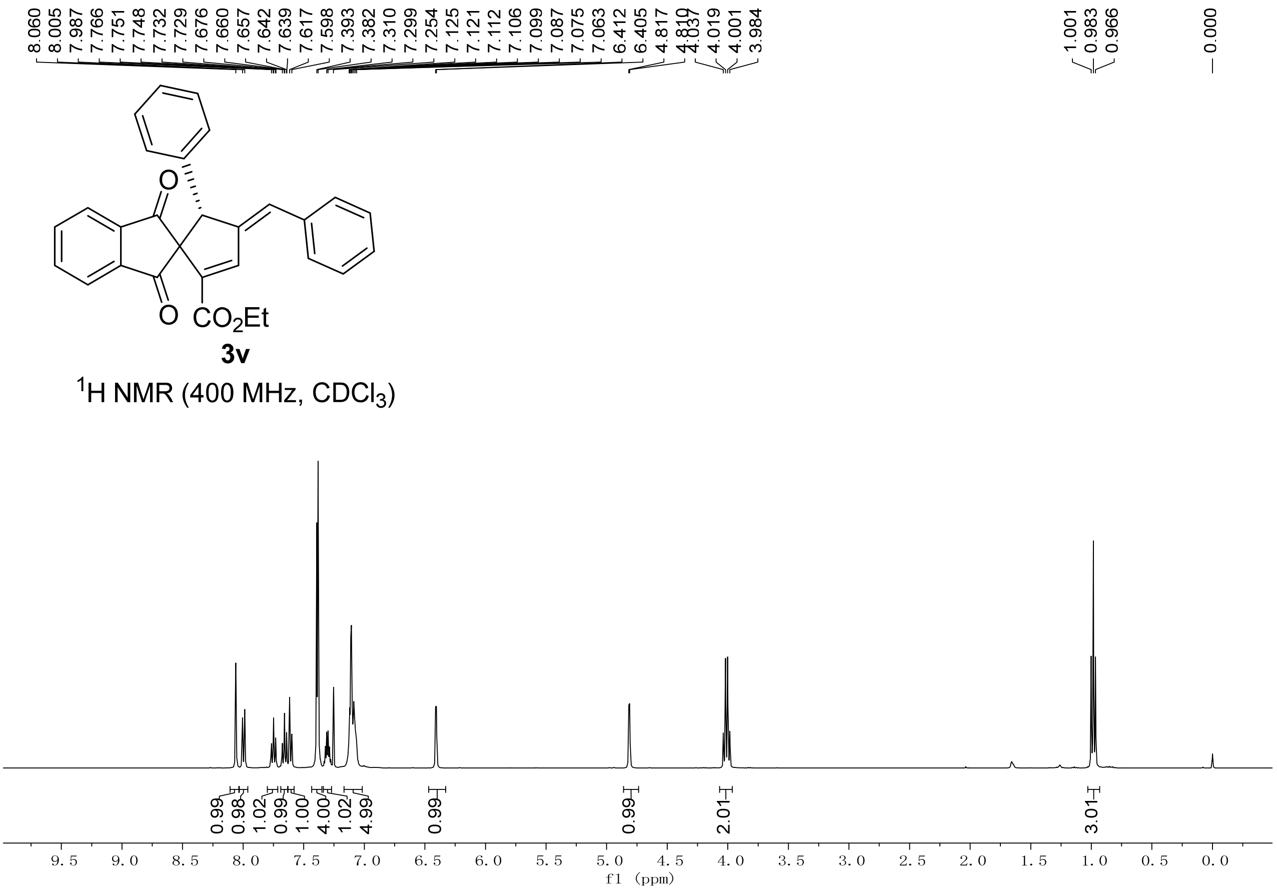
8.060 8.005 7.987 7.766 7.751 7.748 7.732 7.729 7.676 7.660 7.657 7.642 7.639 7.617 7.598 7.393 7.382 7.310 7.299 7.254 7.125 7.121 7.112 7.106 7.099 7.087 7.075 7.063 6.412 6.405 4.817 4.810 4.037 4.019 4.001 3.984
1.001 0.983 0.966
0.000
O
O
CO2Et
3v
1H NMR (400 MHz, CDCl3)
0.99 0.98 1.02 0.99 1.00 4.00 1.02 4.99 0.99 0.99 2.01 3.01
9.5 9.0 8.5 8.0 7.5 7.0 6.5 6.0 5.5 5.0 4.5 4.0 3.5 3.0 2.5 2.0 1.5 1.0 0.5 0.0
f1 (ppm)

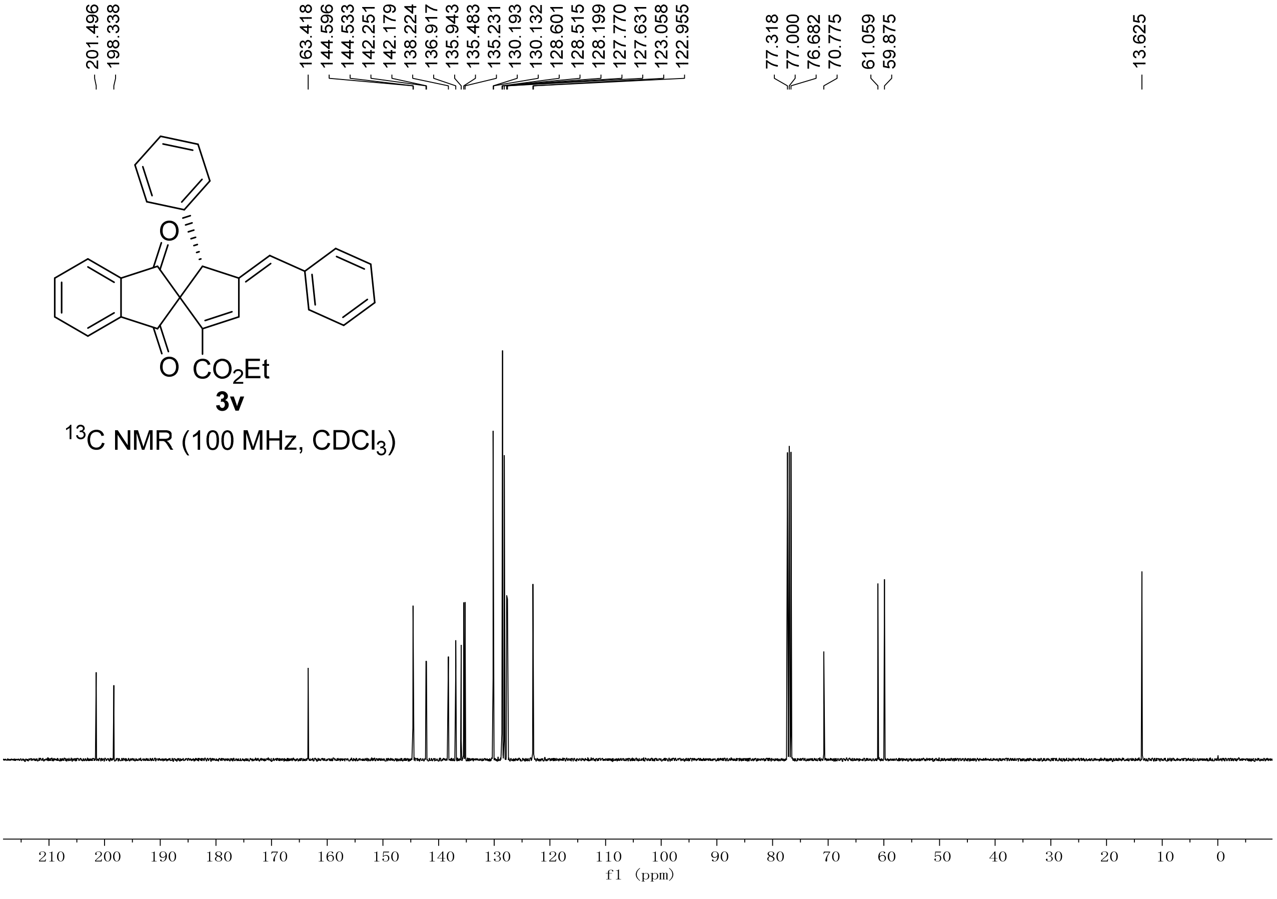
201.496 198.338
163.418 144.596 144.533 142.251 142.179 138.224 136.917 135.943 135.483 135.231 130.193 130.132 128.601 128.515 128.199 127.770 127.631 123.058 122.955
77.318 77.000 76.682 70.775
61.059 59.875
13.625
O
O
CO2Et
3v
13C NMR (100 MHz, CDCl3)
210 200 190 180 170 160 150 140 130 120 110 100 90 80 70 60 50 40 30 20 10 0
f1 (ppm)

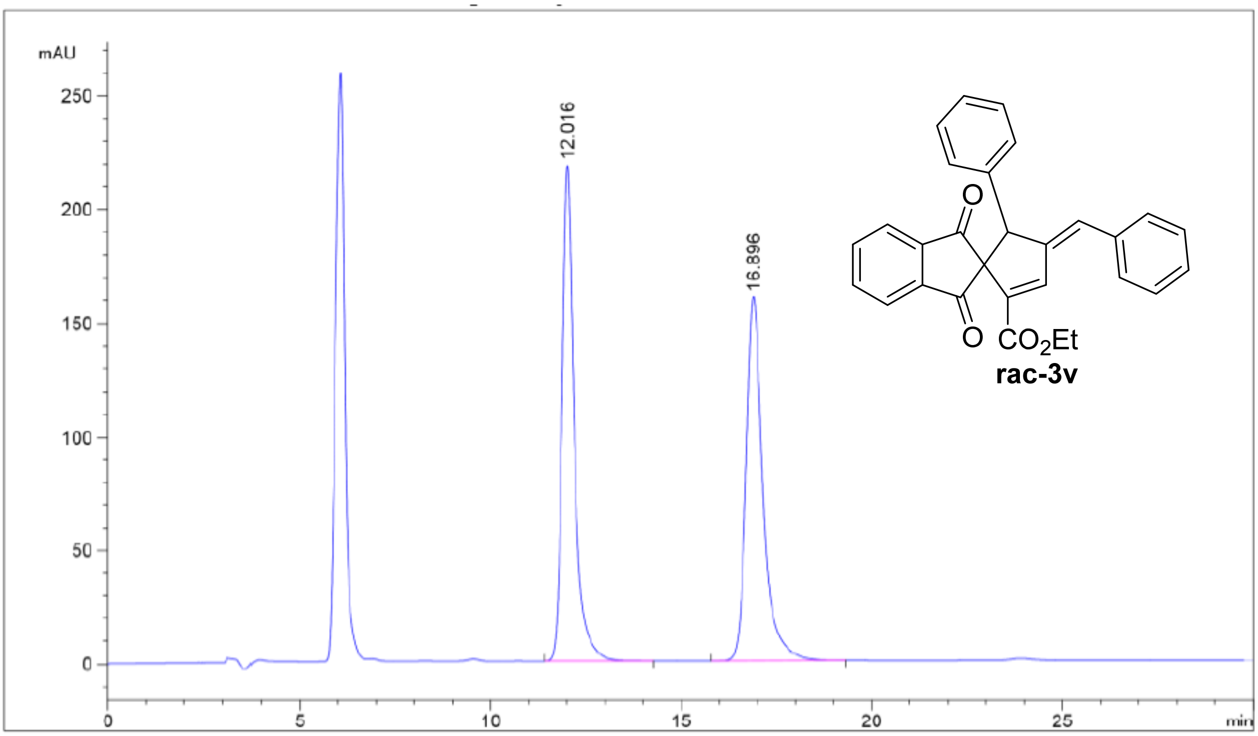

Peak #	RetTime [min]	Type	Width [min]	Area mAU *s	Height [mAU]	Area %
1	12.016	BB	0.3301	4807.11279	217.96622	49.6256
2	16.896	BB	0.4563	4879.64014	159.96458	50.3744

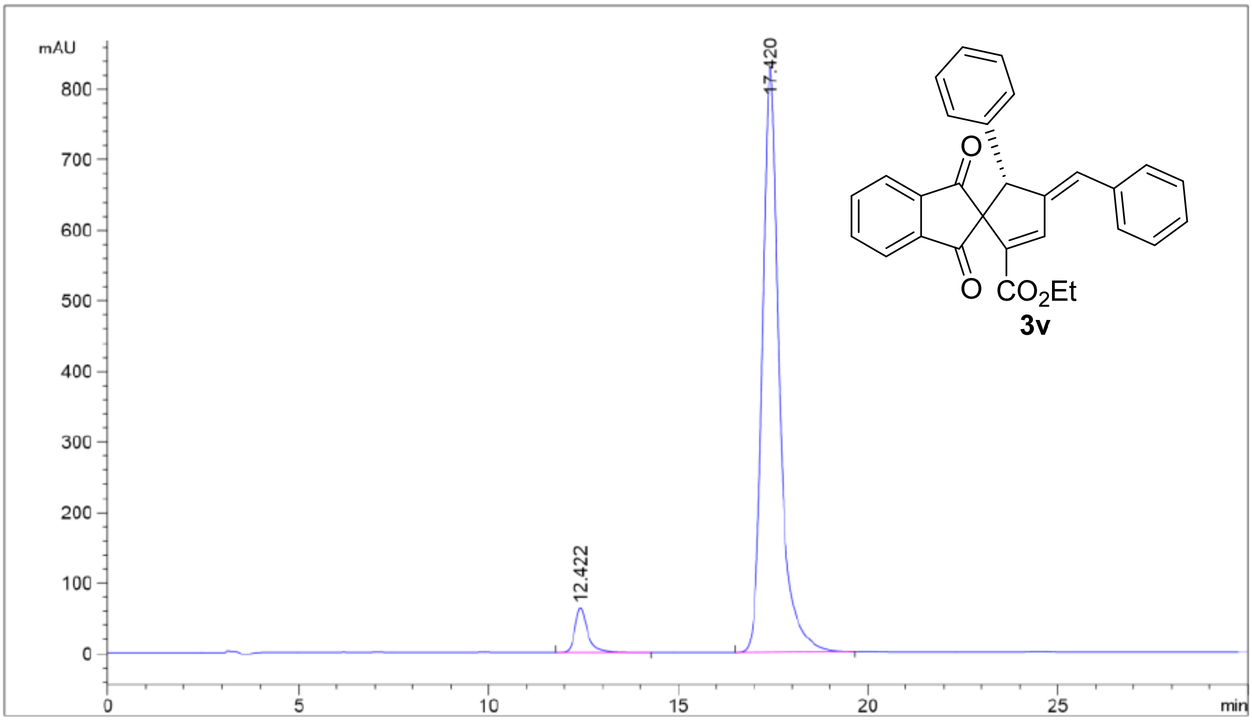

Peak #	RetTime [min]	Type	Width [min]	Area mAU *s	Height [mAU]	Area %
1	12.422	BB	0.3407	1409.38733	61.70353	5.1863
2	17.420	BB	0.4688	2.57660e4	829.13666	94.8137

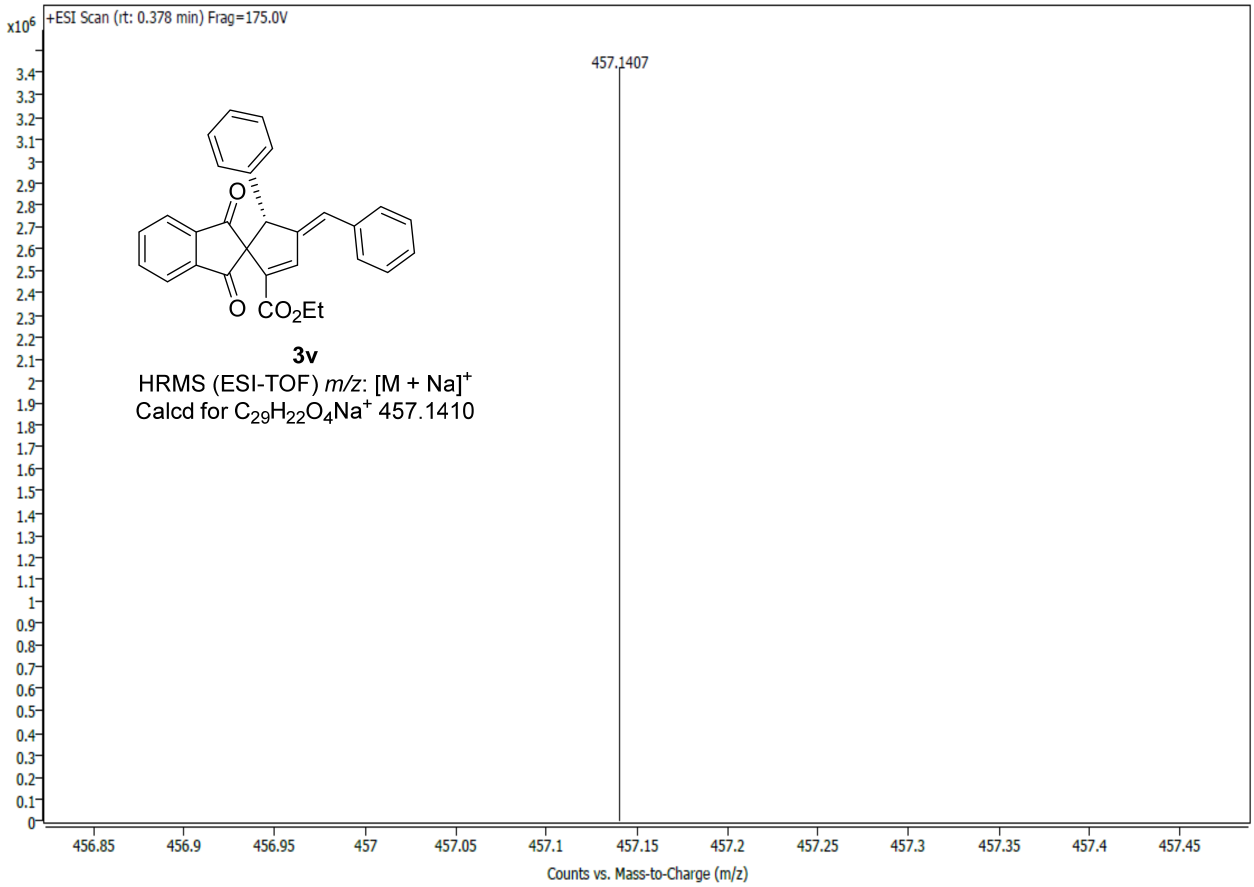
x10^6
+ESI Scan (rt: 0.378 min) Frag=175.0V
457.1407
O
O
CO2Et
3v
HRMS (ESI-TOF) m/z: [M + Na]+
Calcd for C29H22O4Na+ 457.1410
3.4
3.3
3.2
3.1
3
2.9
2.8
2.7
2.6
2.5
2.4
2.3
2.2
2.1
2
1.9
1.8
1.7
1.6
1.5
1.4
1.3
1.2
1.1
1
0.9
0.8
0.7
0.6
0.5
0.4
0.3
0.2
0.1
0
456.85
456.9
456.95
457
457.05
457.1
457.15
457.2
457.25
457.3
457.35
457.4
457.45
Counts vs. Mass-to-Charge (m/z)

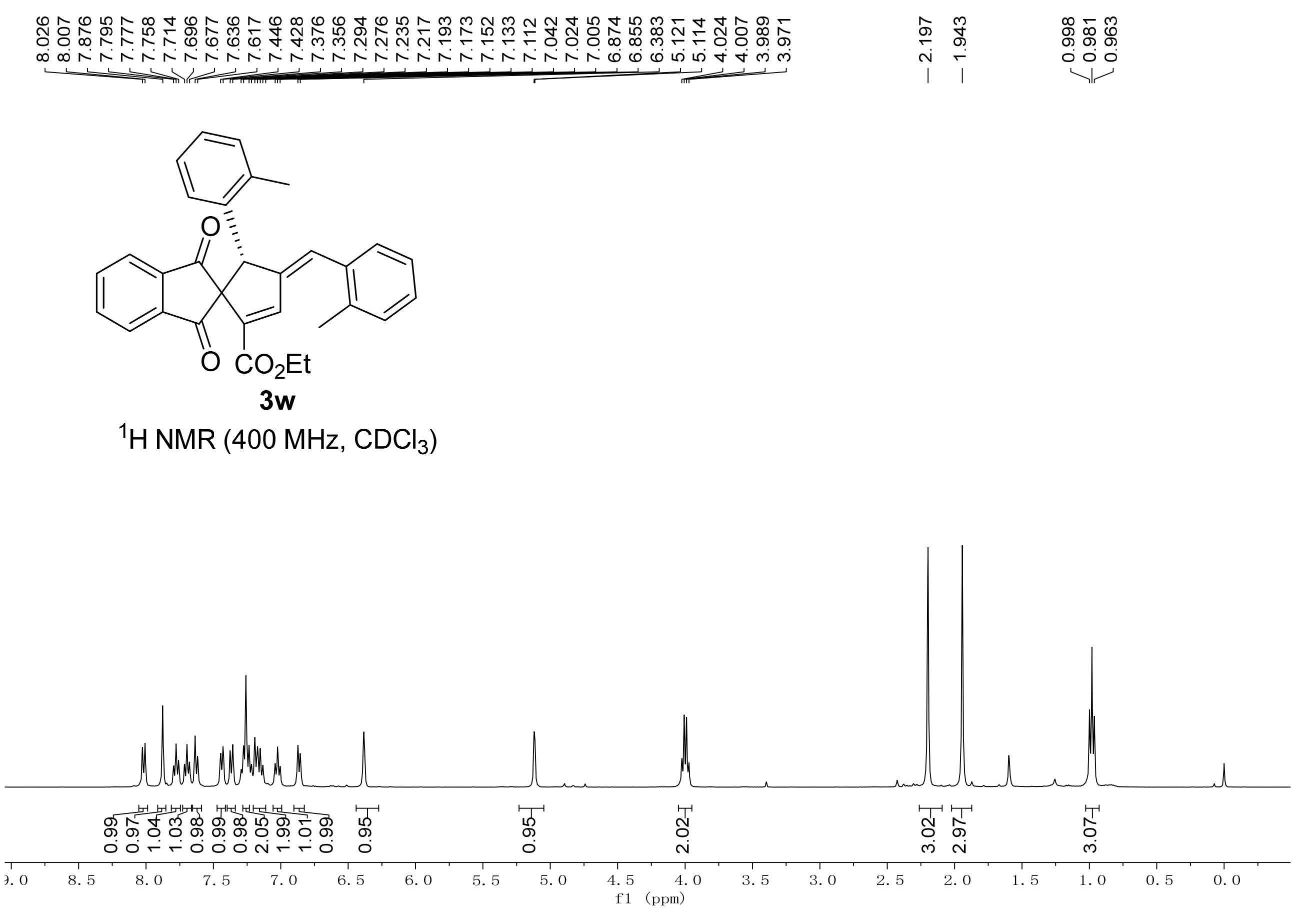
8.026 8.007 7.876 7.795 7.777 7.758 7.714 7.696 7.677 7.636 7.617 7.446 7.428 7.376 7.356 7.294 7.276 7.235 7.217 7.193 7.173 7.152 7.133 7.112 7.042 7.024 7.005 6.874 6.855 6.383 5.121 5.114 4.024 4.007 3.989 3.971
2.197
1.943
0.998 0.981 0.963
O
O
CO2Et
3w
1H NMR (400 MHz, CDCl3)
0.99 0.97 1.04 1.03 0.98 0.99 0.98 2.05 1.99 1.01 0.99 0.95 0.95 2.02 3.02 2.97 3.07
f1 (ppm)

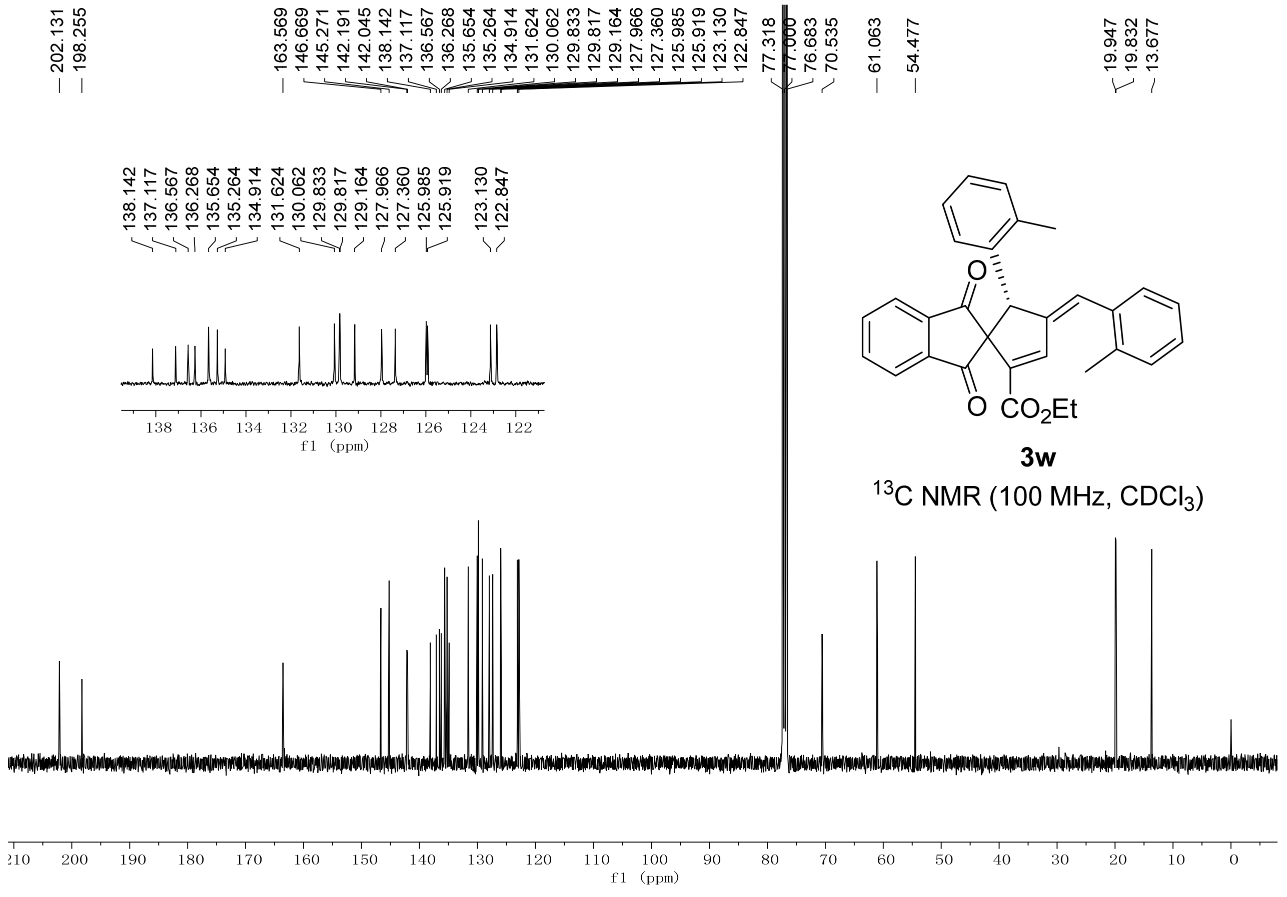
202.131 198.255
163.569 146.669 145.271 142.191 142.045 138.142 137.117 136.567 136.268 135.654 135.264 134.914 131.624 130.062 129.833 129.817 129.164 127.966 127.360 125.985 125.919 123.130 122.847
77.318 77.000 76.683 70.535
61.063
54.477
19.947 19.832 13.677
138.142 137.117 136.567 136.268 135.654 135.264 134.914 131.624 130.062 129.833 129.817 129.164 127.966 127.360 125.985 125.919 123.130 122.847
f1 (ppm)
O
O
CO2Et
3w
13C NMR (100 MHz, CDCl3)
f1 (ppm)

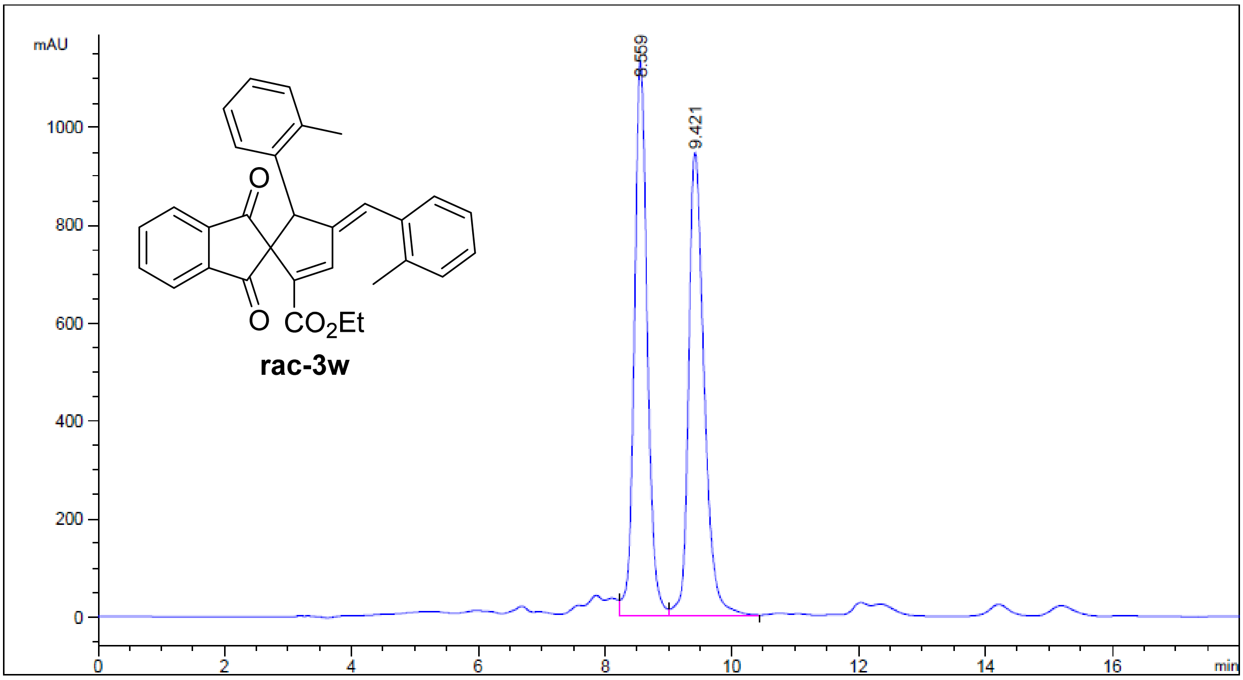

rac-3w

Peak #	RetTime [min]	Type	Width [min]	Area mAU *s	Height [mAU]	Area %
1	8.559	VV	0.2170	1.60807e4	1130.06592	49.9619
2	9.421	VV	0.2600	1.61053e4	944.85883	50.0381

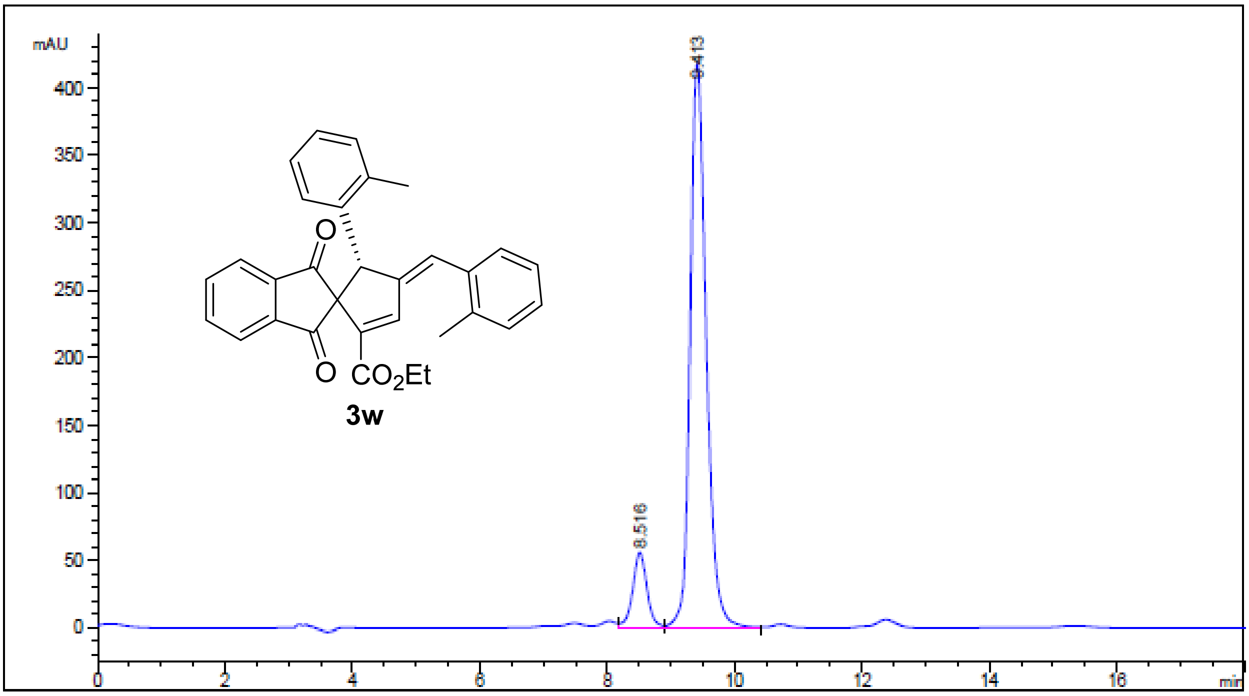

3w

Peak #	RetTime [min]	Type	Width [min]	Area mAU *s	Height [mAU]	Area %
1	8.516	VV	0.2371	873.90643	55.64470	10.7192
2	9.413	VB	0.2656	7278.80908	418.39142	89.2808

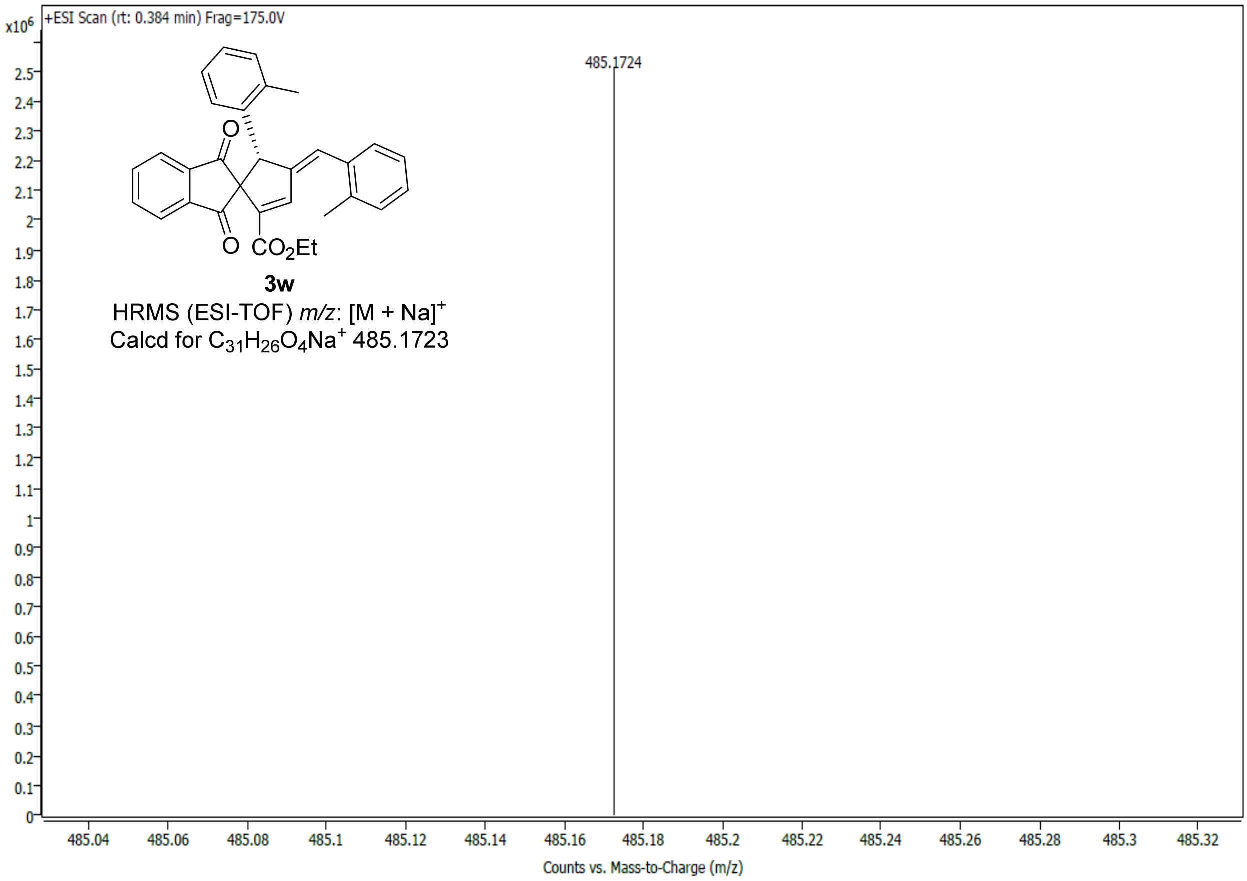

x10^6
+ESI Scan (rt: 0.384 min) Frag=175.0V
485.1724
3w
HRMS (ESI-TOF) m/z: [M + Na]+
Calcd for C31H26O4Na+ 485.1723
CO2Et
Counts vs. Mass-to-Charge (m/z)

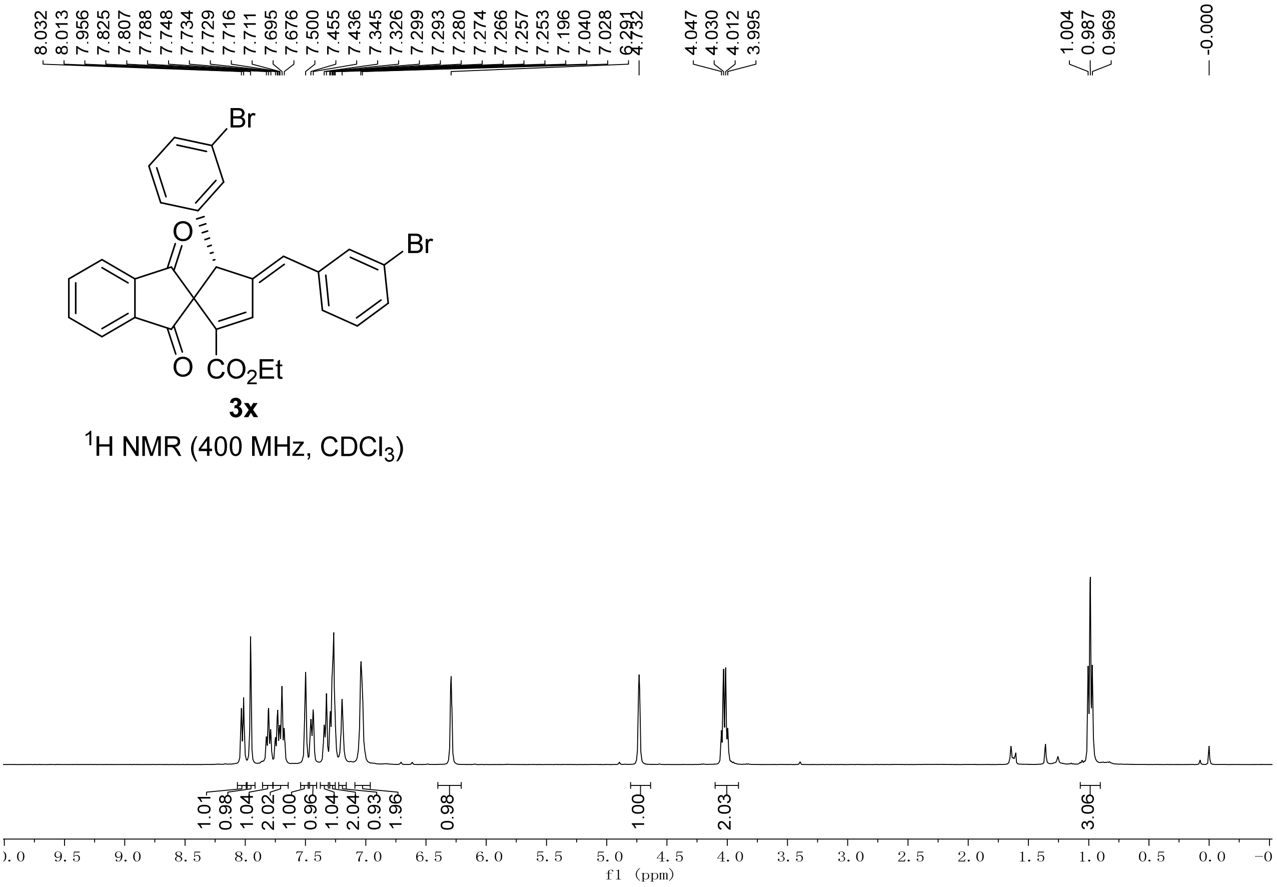
8.032 8.013 7.956 7.825 7.807 7.788 7.748 7.734 7.729 7.716 7.711 7.695 7.676 7.500 7.455 7.436 7.345 7.326 7.299 7.293 7.280 7.274 7.266 7.257 7.253 7.196 7.040 7.028 6.291 4.732
4.047 4.030 4.012 3.995
1.004 0.987 0.969
-0.000
Br
Br
O
O
CO_2Et
3x
^{1}H NMR (400 MHz, $CDCl_3$)
1.01 0.98 1.04 2.02 1.00 0.96 1.04 2.04 0.93 1.96
0.98
1.00
2.03
3.06
f1 (ppm)

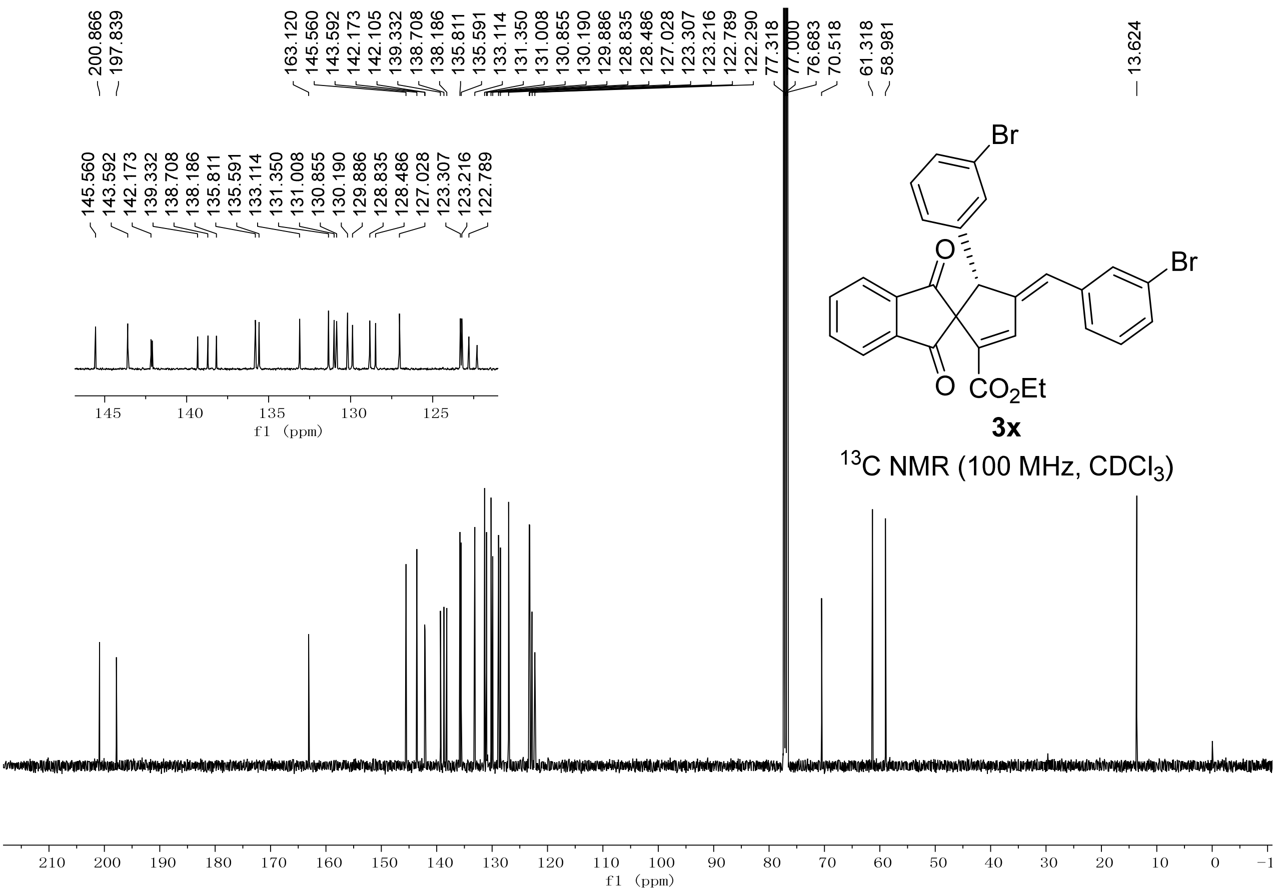
200.866 197.839
163.120 145.560 143.592 142.173 142.105 139.332 138.708 138.186 135.811 135.591 133.114 131.350 131.008 130.855 130.190 129.886 128.835 128.486 127.028 123.307 123.216 122.789 122.290 77.318 77.000 76.683 70.518
61.318 58.981
13.624
145.560 143.592 142.173 139.332 138.708 138.186 135.811 135.591 133.114 131.350 131.008 130.855 130.190 129.886 128.835 128.486 127.028 123.307 123.216 122.789
f1 (ppm)
Br
Br
O
O
CO_2Et
3x
^{13}C NMR (100 MHz, $CDCl_3$)
f1 (ppm)

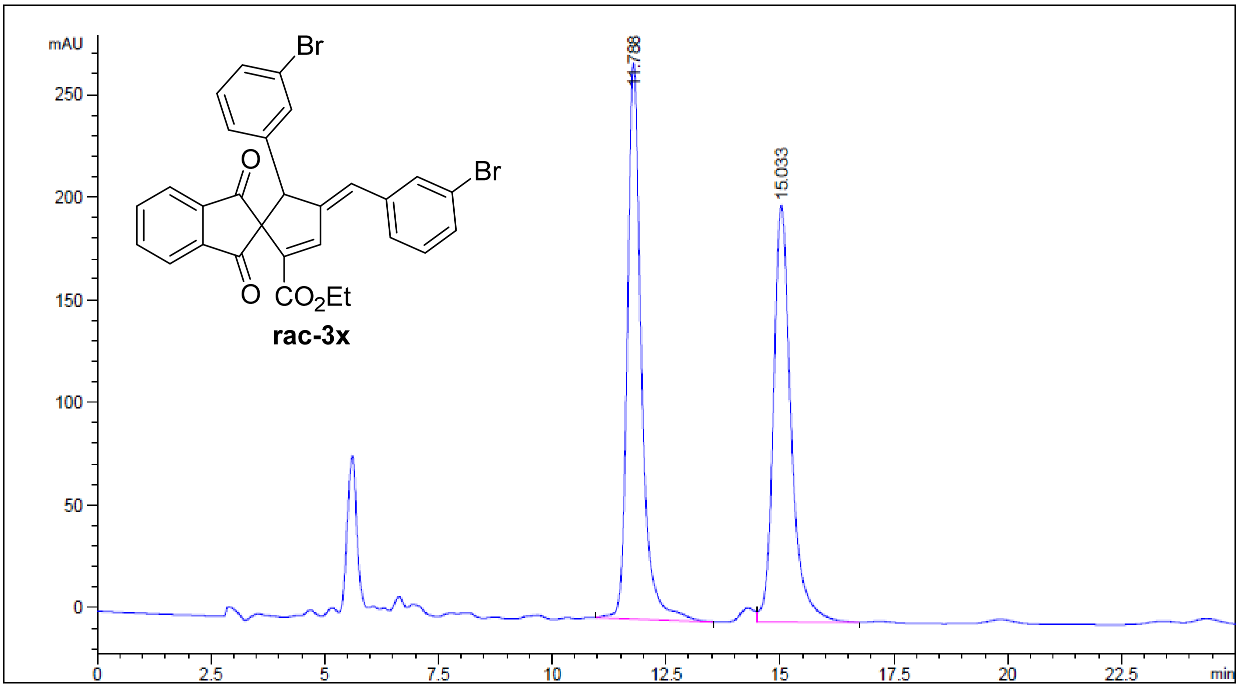

Peak #	RetTime [min]	Type	Width [min]	Area mAU *s	Area %
1	11.788	BB	0.3218	5826.33838	51.5570
2	15.033	VB	0.4036	5474.43701	48.4430

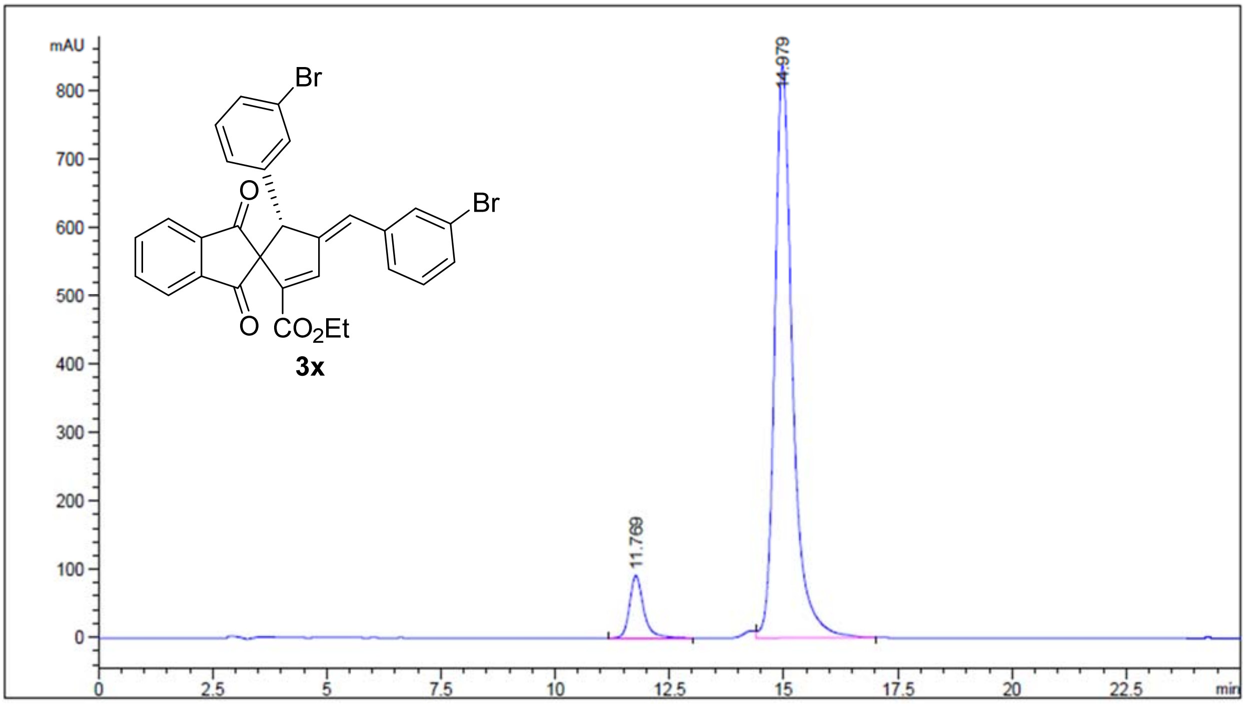

Peak #	RetTime [min]	Type	Width [min]	Area mAU *s	Area %
1	11.769	BB	0.3186	1951.93884	7.9035
2	14.979	VB	0.4077	2.27451e4	92.0965

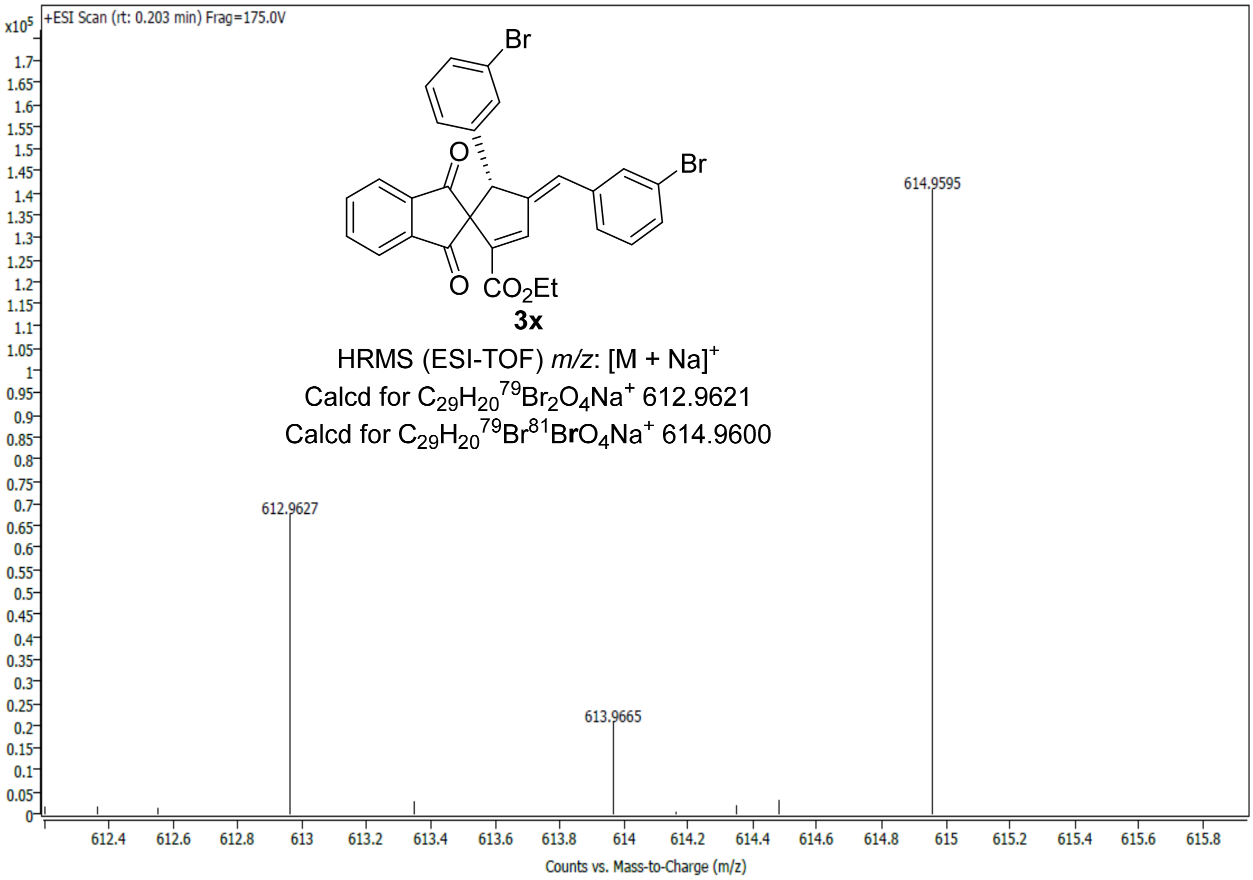
+ESI Scan (rt: 0.203 min) Frag=175.0V
x10^5
3x
HRMS (ESI-TOF) m/z: [M + Na]+
Calcd for C29H20 79Br2O4Na+ 612.9621
Calcd for C29H20 79Br81BrO4Na+ 614.9600
612.9627
613.9665
614.9595
Counts vs. Mass-to-Charge (m/z)

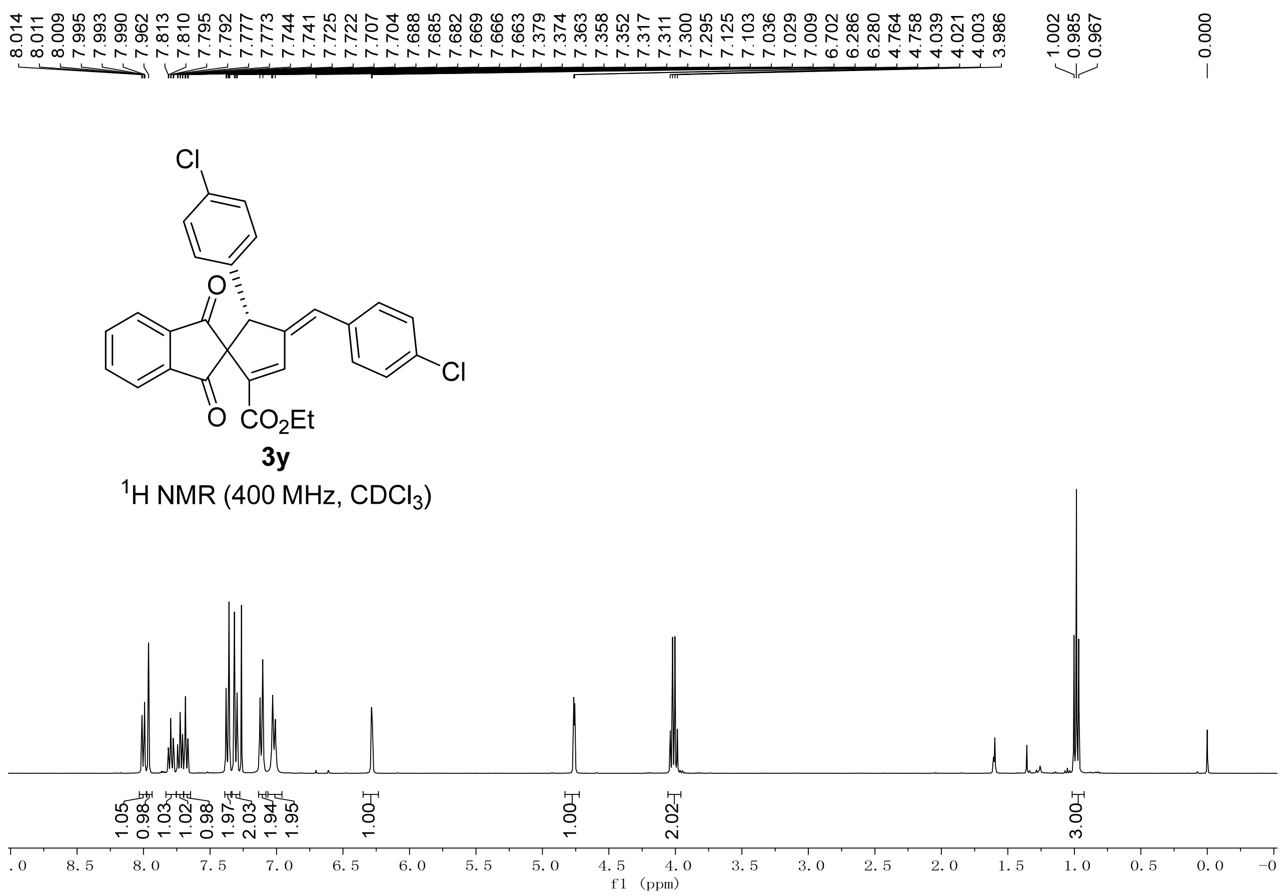
8.014 8.011 8.009 7.995 7.993 7.990 7.962 7.813 7.810 7.795 7.792 7.777 7.773 7.744 7.741 7.725 7.722 7.707 7.704 7.688 7.685 7.682 7.669 7.666 7.663 7.379 7.374 7.363 7.358 7.352 7.317 7.311 7.300 7.295 7.125 7.103 7.036 7.029 7.009 6.702 6.286 6.280 4.764 4.758 4.039 4.021 4.003 3.986
1.002 0.985 0.967
0.000
Cl
O
Cl
O
CO2Et
3y
1H NMR (400 MHz, CDCl3)
1.05 0.98 1.03 1.02 0.98 1.97 2.03 1.94 1.95
1.00
1.00
2.02
3.00
f1 (ppm)

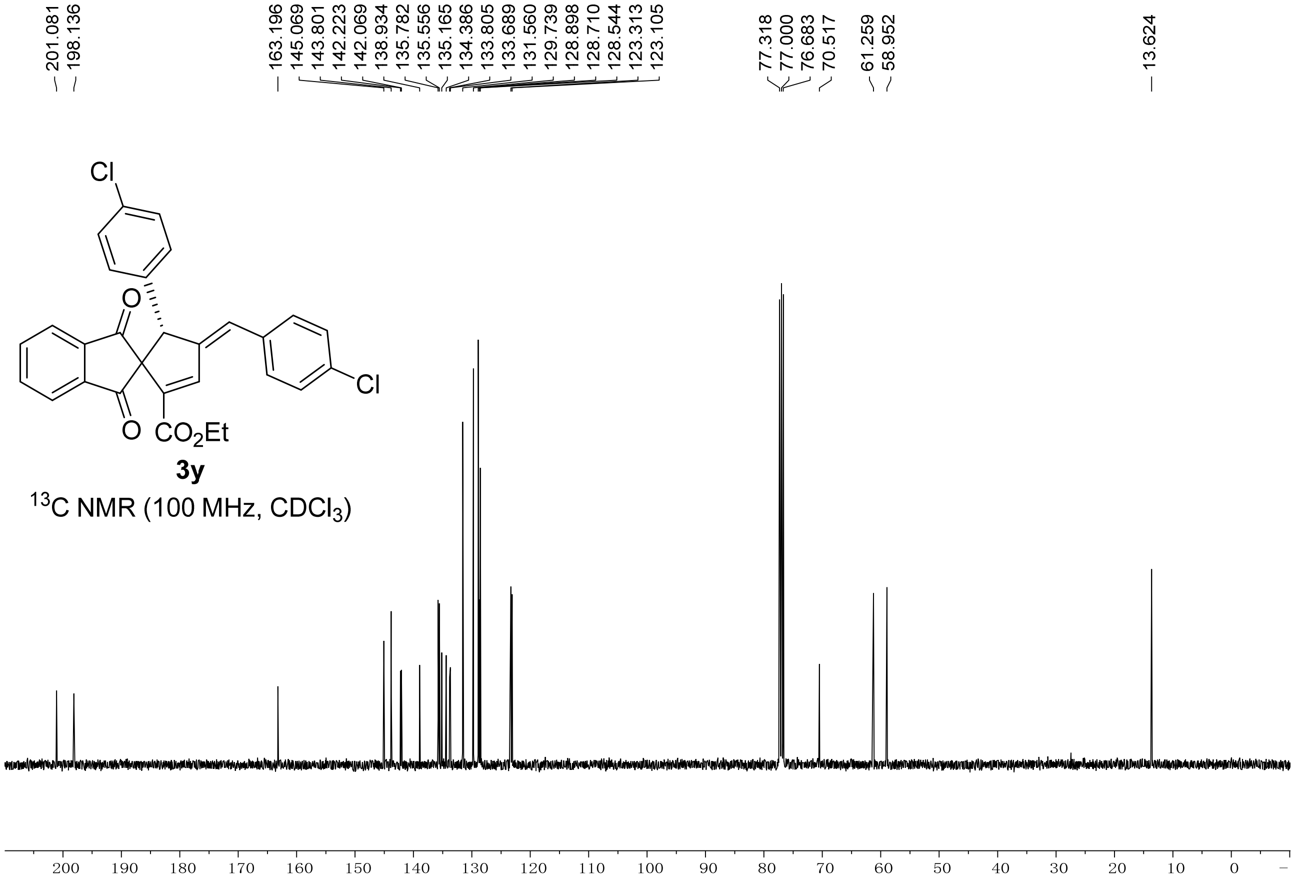
201.081 198.136
163.196 145.069 143.801 142.223 142.069 138.934 135.782 135.556 135.165 134.386 133.805 133.689 131.560 129.739 128.898 128.710 128.544 123.313 123.105
77.318 77.000 76.683 70.517
61.259 58.952
13.624
Cl
O
Cl
O
CO2Et
3y
13C NMR (100 MHz, CDCl3)
f1 (ppm)

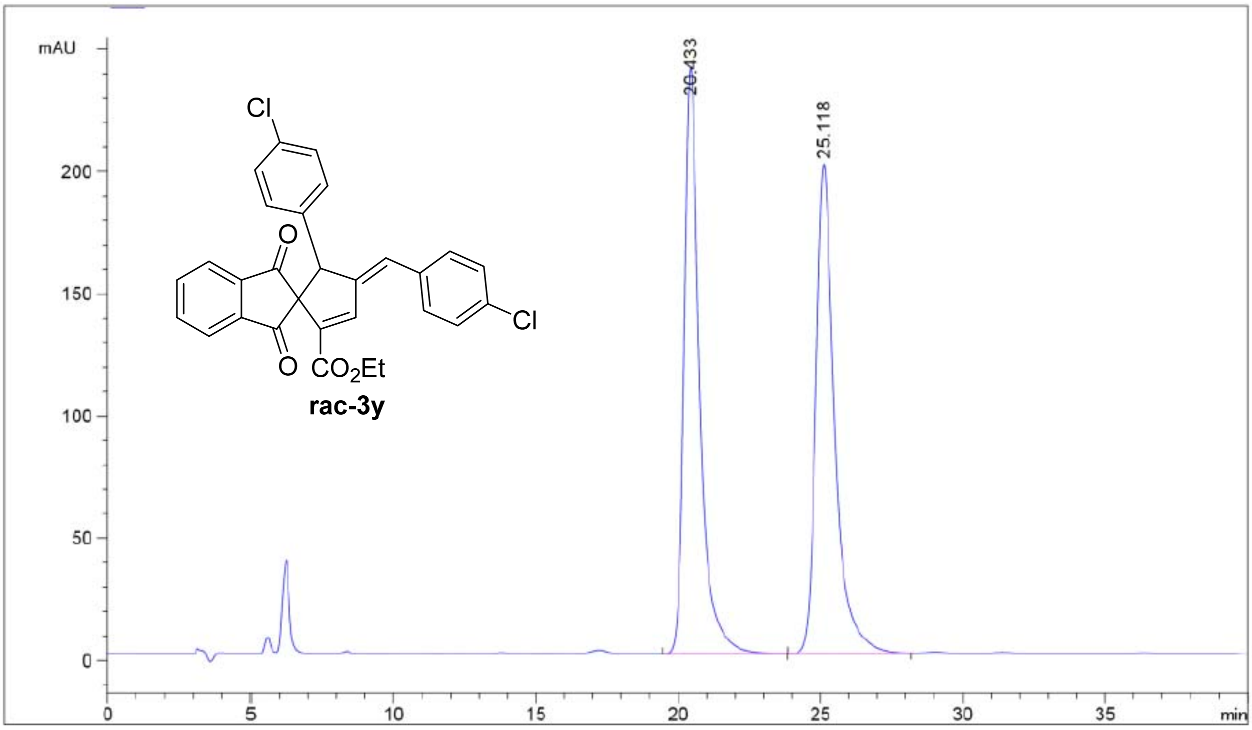

Peak #	RetTime [min]	Type	Width [min]	Area mAU *s	Height [mAU]	Area %
1	20.433	BB	0.5656	9116.73438	239.93542	49.9440
2	25.118	BBA	0.6830	9137.19434	200.00278	50.0560

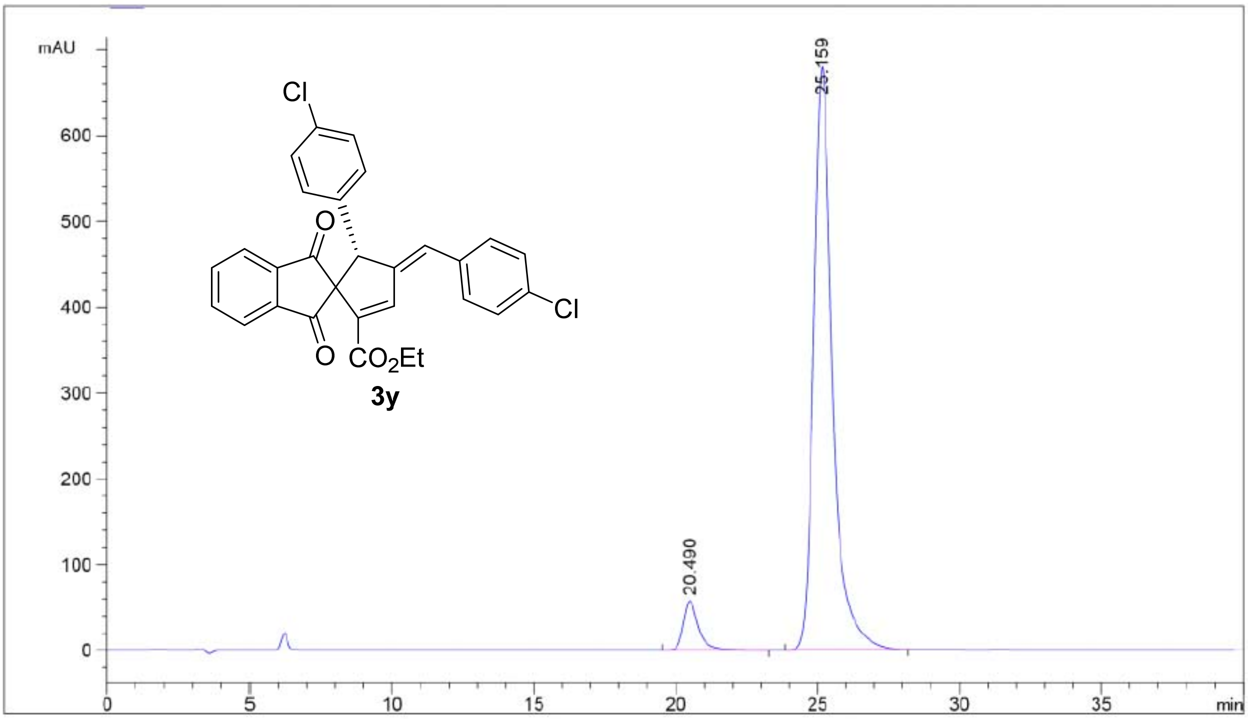

Peak #	RetTime [min]	Type	Width [min]	Area mAU *s	Height [mAU]	Area %
1	20.490	BB	0.5616	2186.34326	57.87322	6.5448
2	25.159	BBA	0.6931	3.12196e4	679.72876	93.4552

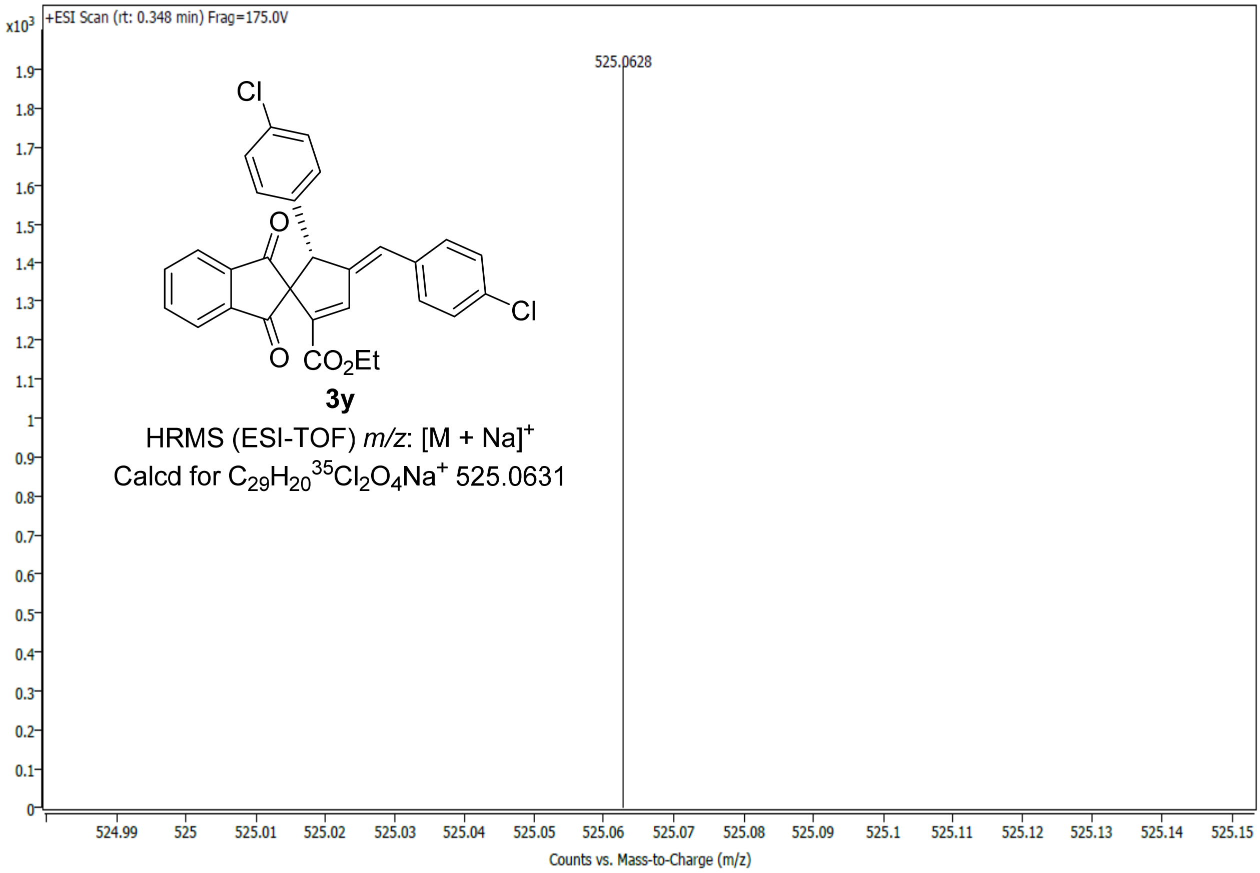
+ESI Scan (rt: 0.348 min) Frag=175.0V
x10^3
525.0628
Cl
O
O
CO2Et
Cl
3y
HRMS (ESI-TOF) m/z: [M + Na]+
Calcd for C29H20 35Cl2O4Na+ 525.0631
Counts vs. Mass-to-Charge (m/z)

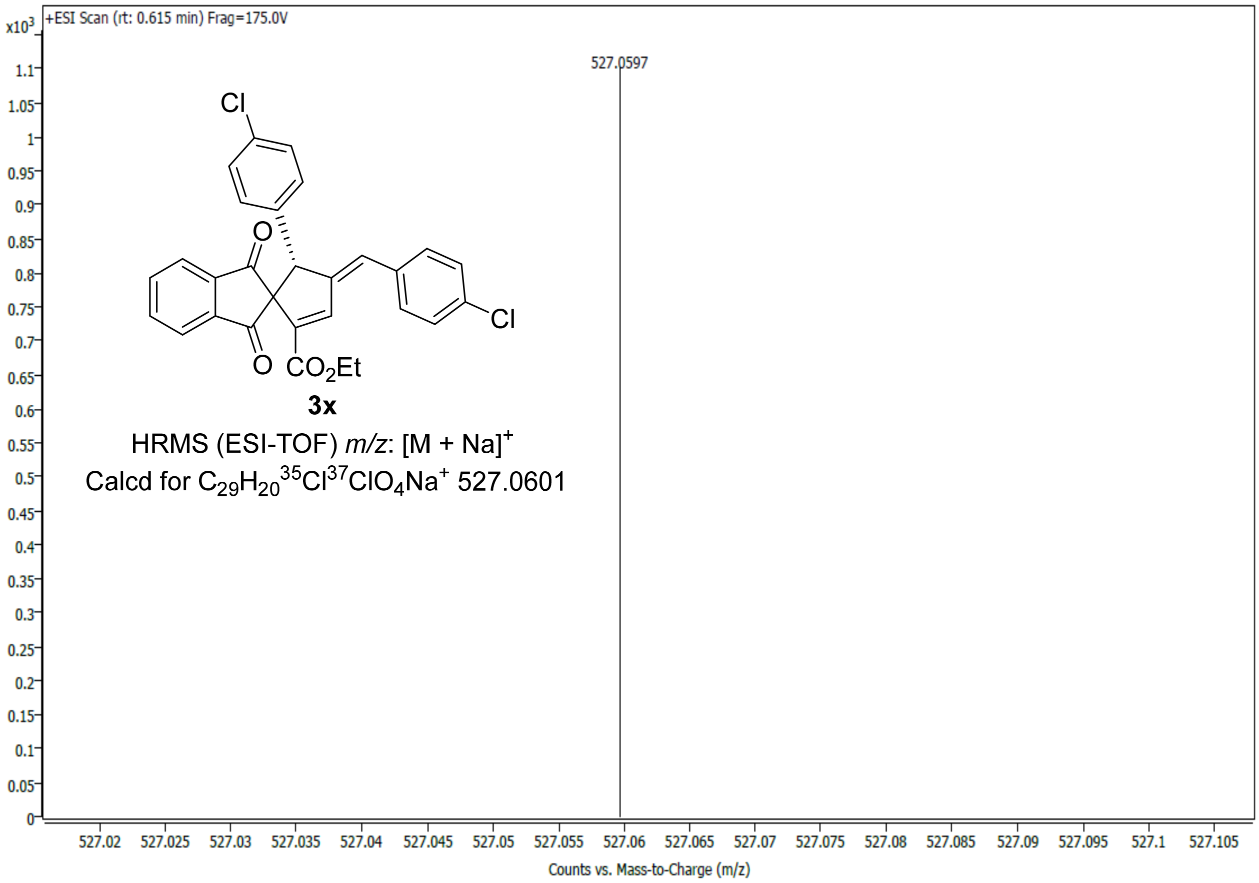
+ESI Scan (rt: 0.615 min) Frag=175.0V
x10^3
527.0597
Cl
O
O
CO2Et
Cl
3x
HRMS (ESI-TOF) m/z: [M + Na]+
Calcd for C29H20 35Cl 37ClO4Na+ 527.0601
Counts vs. Mass-to-Charge (m/z)

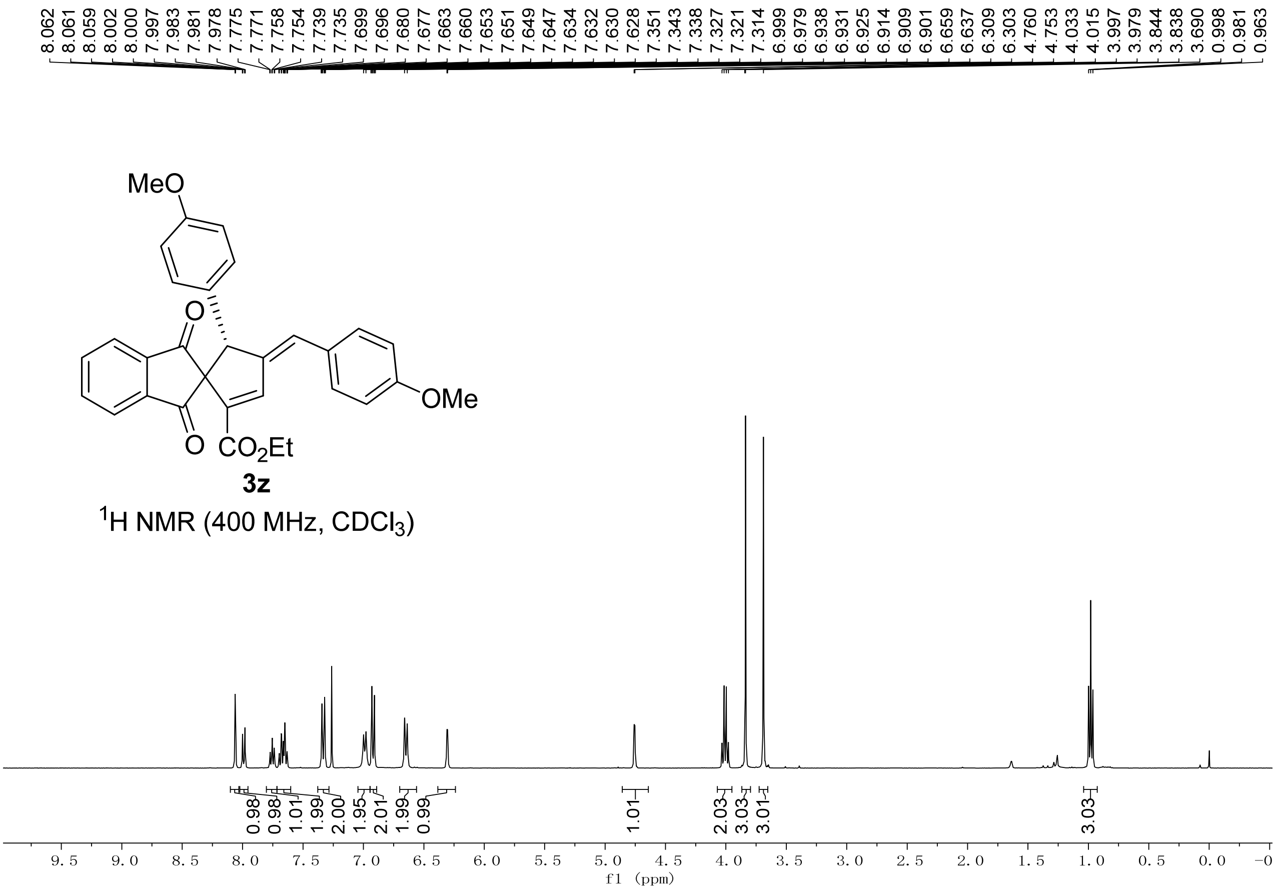
8.062
8.061
8.059
8.002
8.000
7.997
7.983
7.981
7.978
7.775
7.771
7.758
7.754
7.739
7.735
7.699
7.696
7.680
7.677
7.663
7.660
7.653
7.651
7.649
7.647
7.634
7.632
7.630
7.628
7.351
7.343
7.338
7.327
7.321
7.314
6.999
6.979
6.938
6.931
6.925
6.914
6.909
6.901
6.659
6.637
6.309
6.303
4.760
4.753
4.033
4.015
3.997
3.979
3.844
3.838
3.690
0.998
0.981
0.963
MeO
O
O
CO2Et
OMe
3z
1H NMR (400 MHz, CDCl3)
0.98
0.98
1.01
1.99
2.00
1.95
2.01
1.99
0.99
1.01
2.03
3.03
3.01
3.03
f1 (ppm)

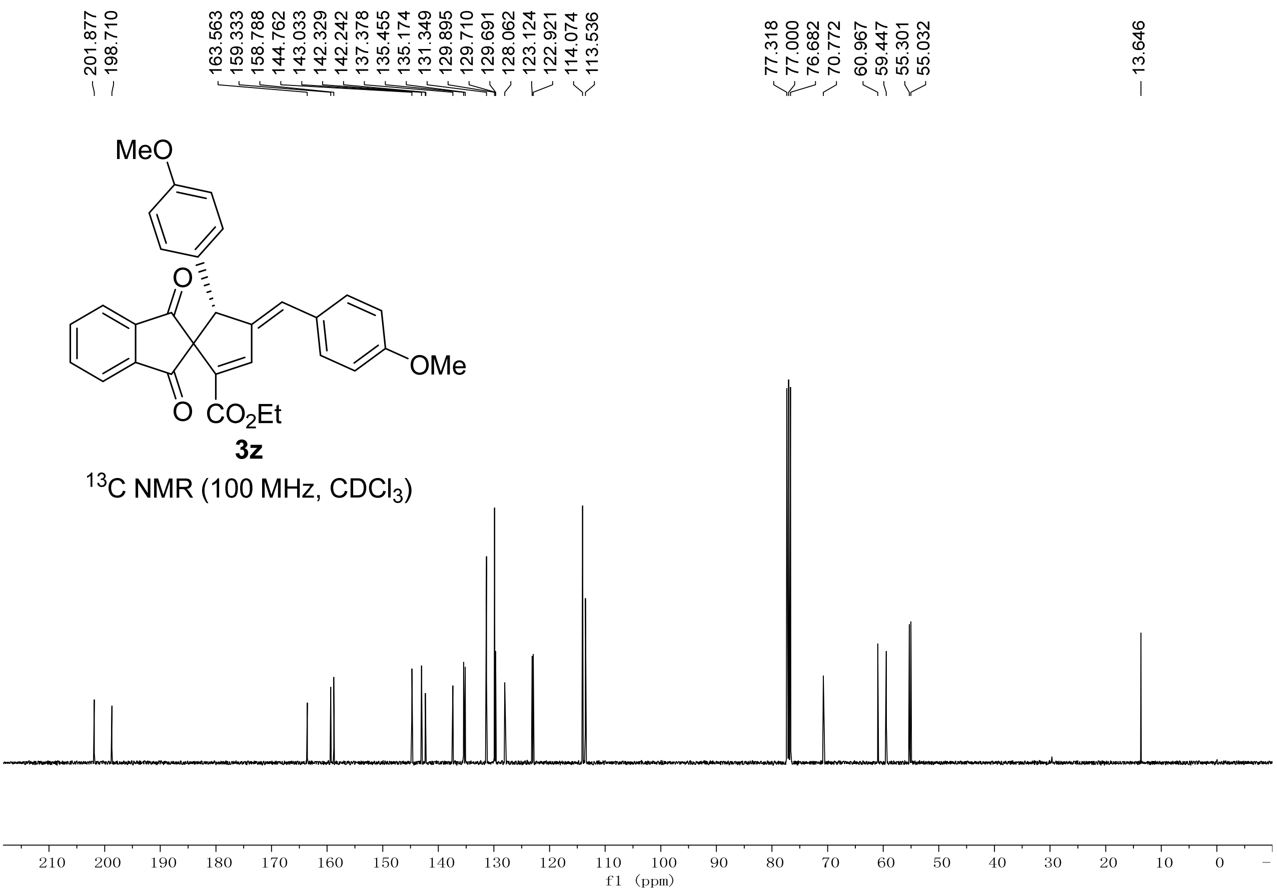
201.877
198.710
163.563
159.333
158.788
144.762
143.033
142.329
142.242
137.378
135.455
135.174
131.349
129.895
129.710
129.691
128.062
123.124
122.921
114.074
113.536
77.318
77.000
76.682
70.772
60.967
59.447
55.301
55.032
13.646
MeO
O
O
CO2Et
OMe
3z
13C NMR (100 MHz, CDCl3)
f1 (ppm)

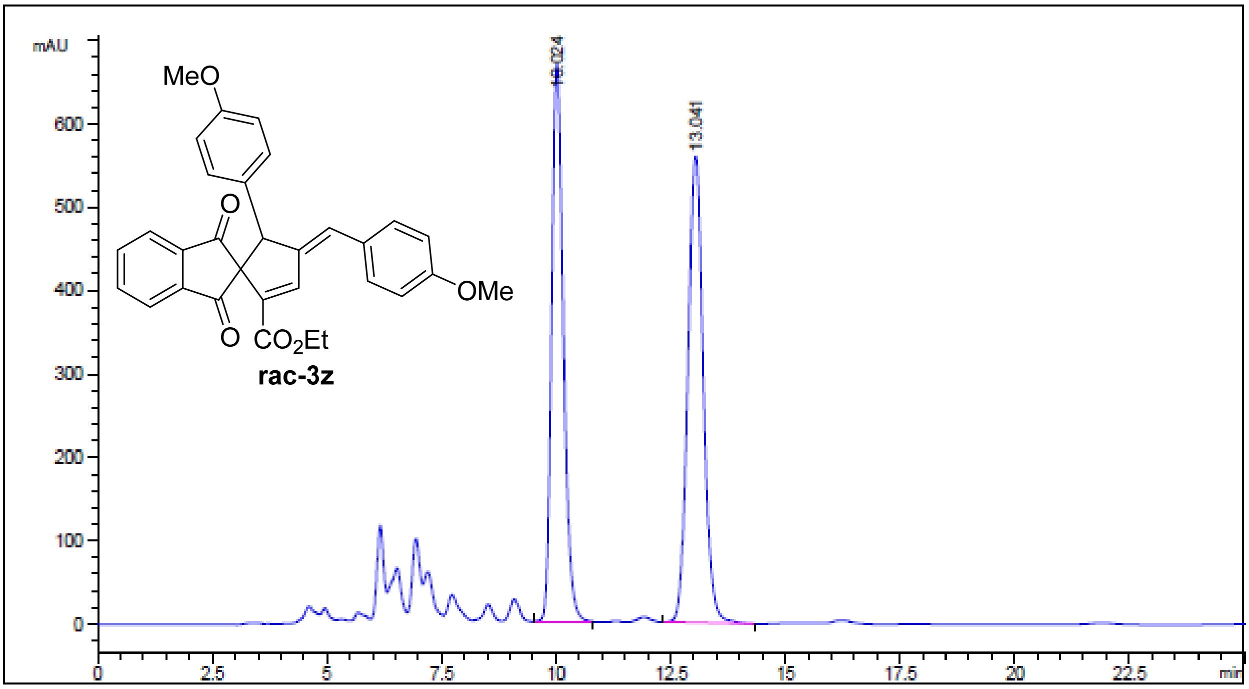

Peak #	RetTime [min]	Type	Width [min]	Area mAU *s	Height [mAU]	Area %
1	10.024	BBA	0.2707	1.17719e4	669.29779	47.7606
2	13.041	BBA	0.3529	1.28758e4	559.60303	52.2394

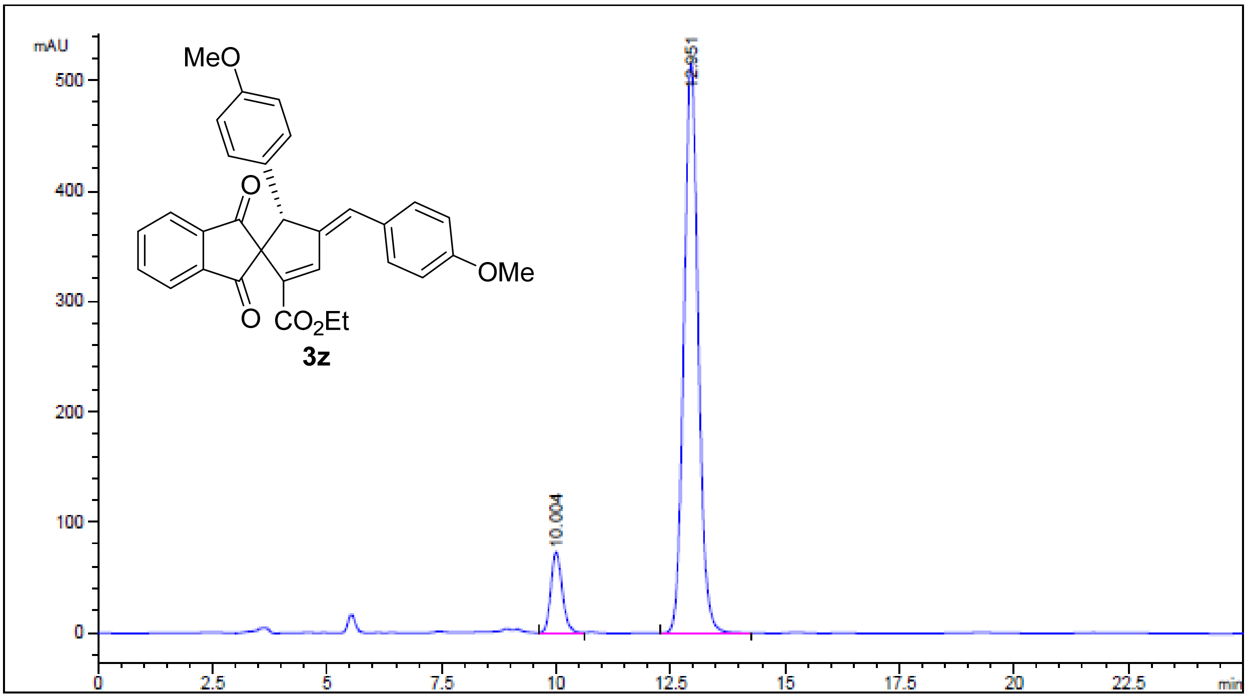

Peak #	RetTime [min]	Type	Width [min]	Area mAU *s	Height [mAU]	Area %
1	10.004	BB	0.2678	1271.04102	73.31892	10.0859
2	12.951	BB	0.3378	1.13311e4	515.83771	89.9141

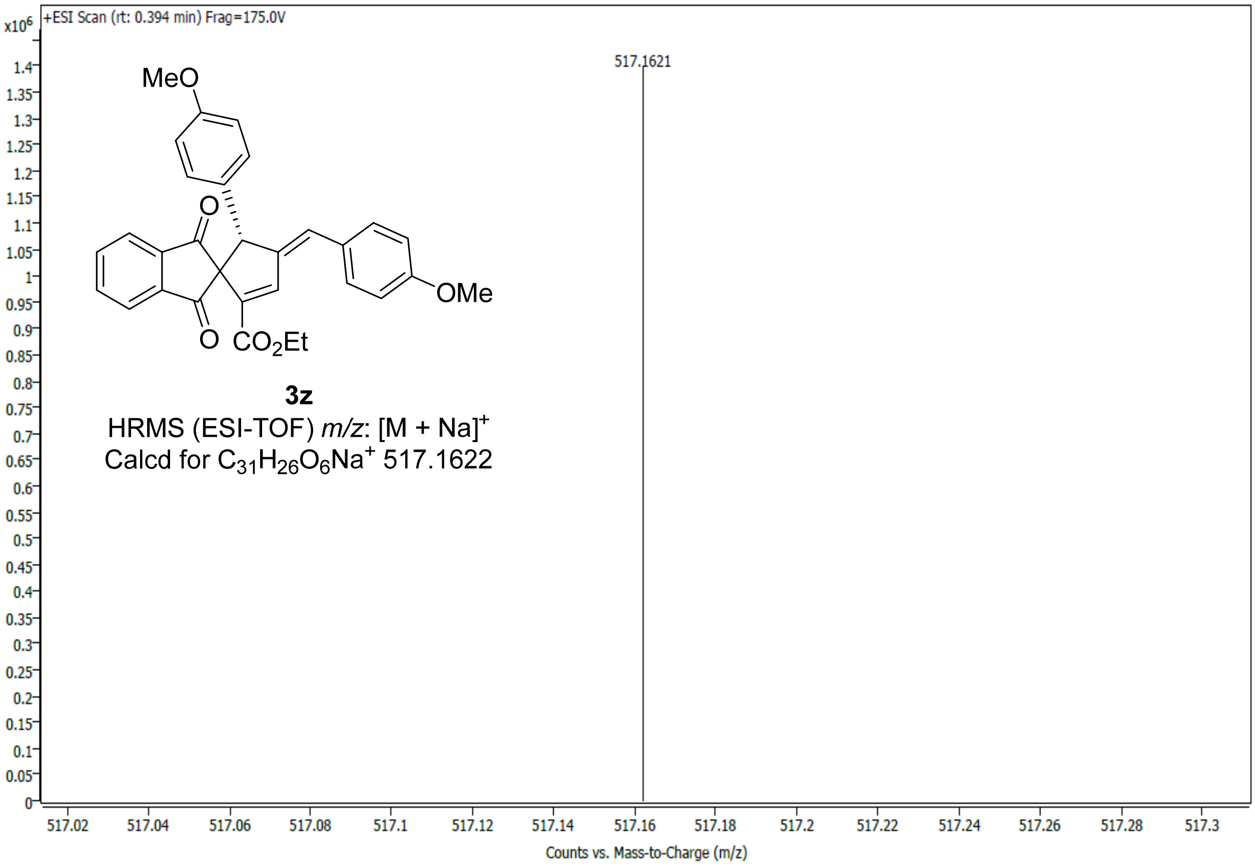

x10^6
+ESI Scan (rt: 0.394 min) Frag=175.0V
517.1621
MeO
O
O
CO2Et
OMe
3z
HRMS (ESI-TOF) m/z: [M + Na]+
Calcd for C31H26O6Na+ 517.1622
Counts vs. Mass-to-Charge (m/z)

4a

^{1}H NMR (400 MHz, $CDCl_3$)

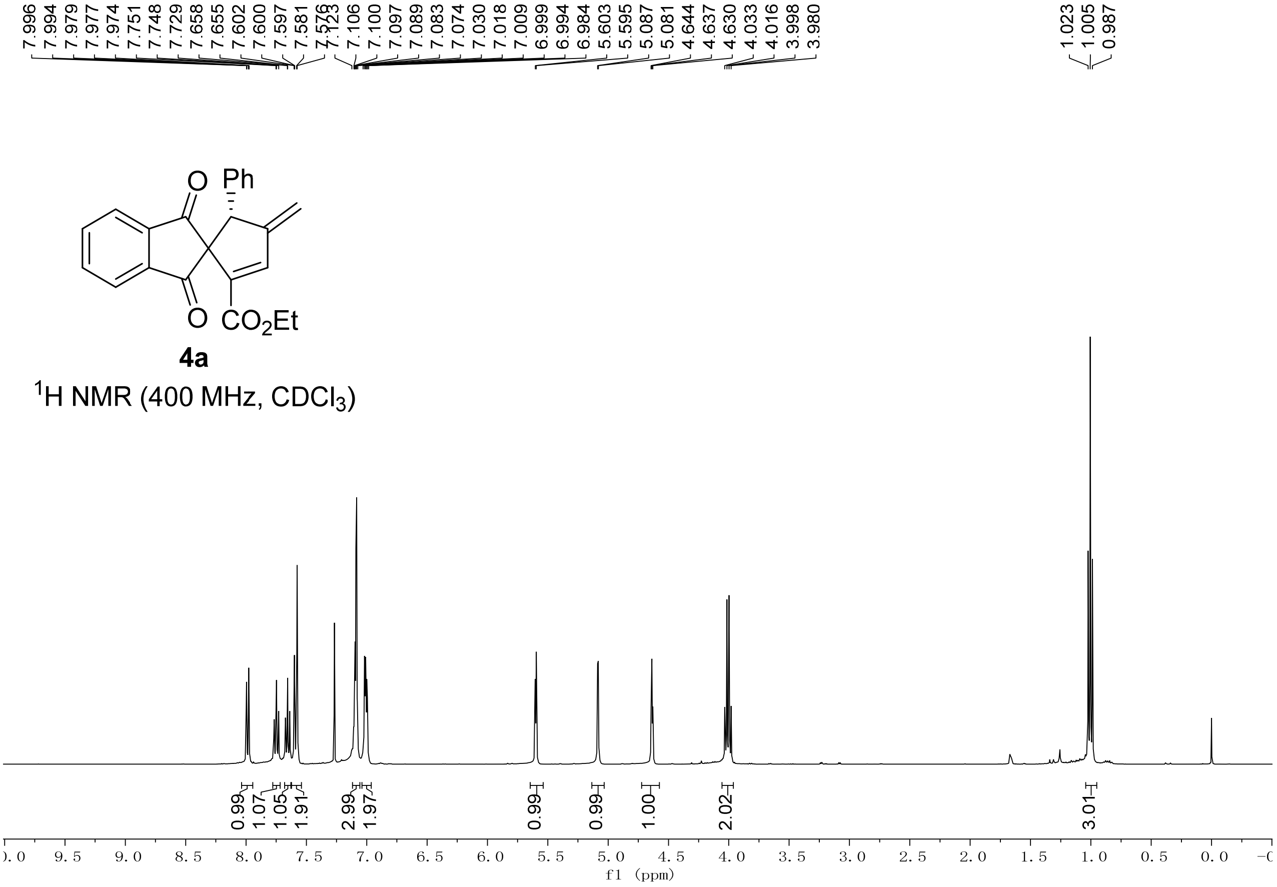

4a

^{13}C NMR (100 MHz, $CDCl_3$)

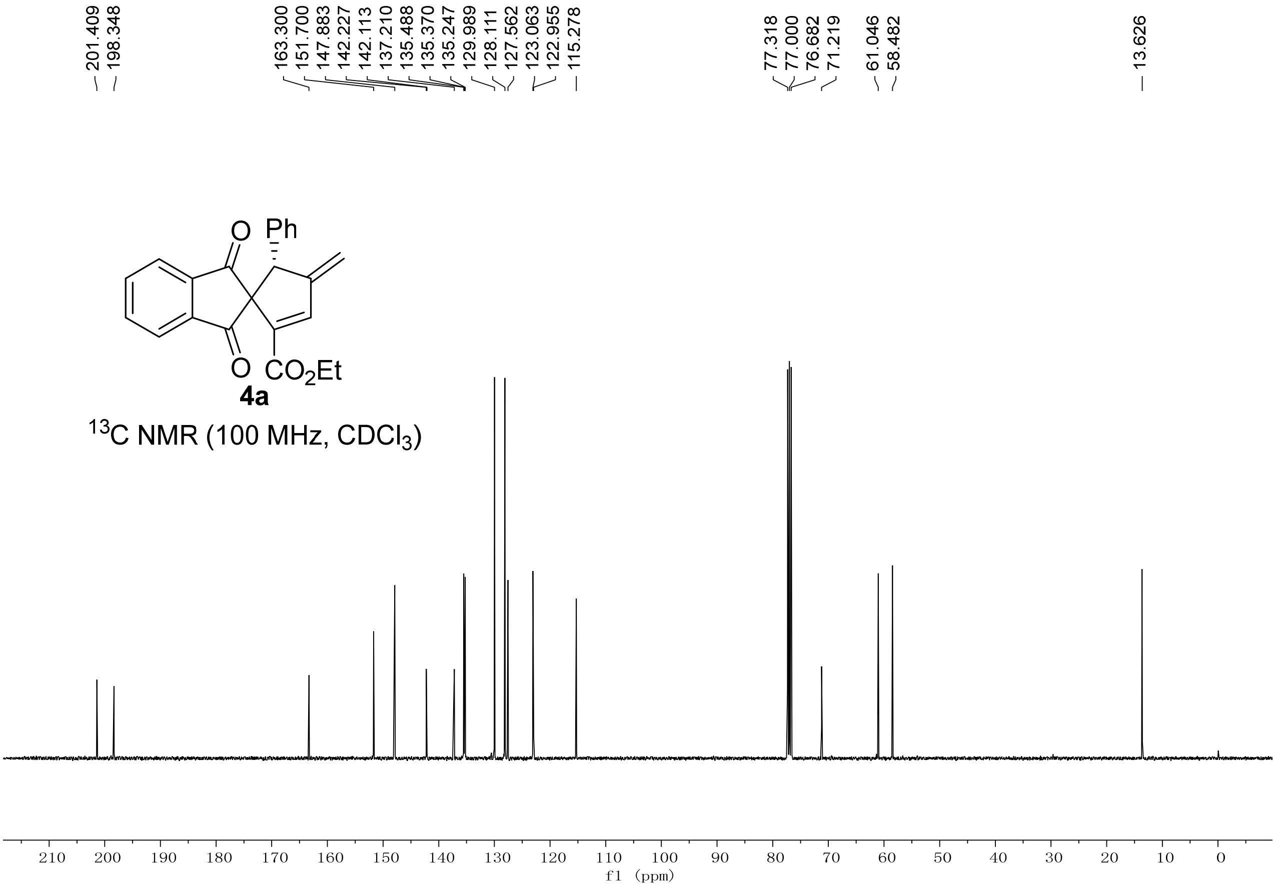

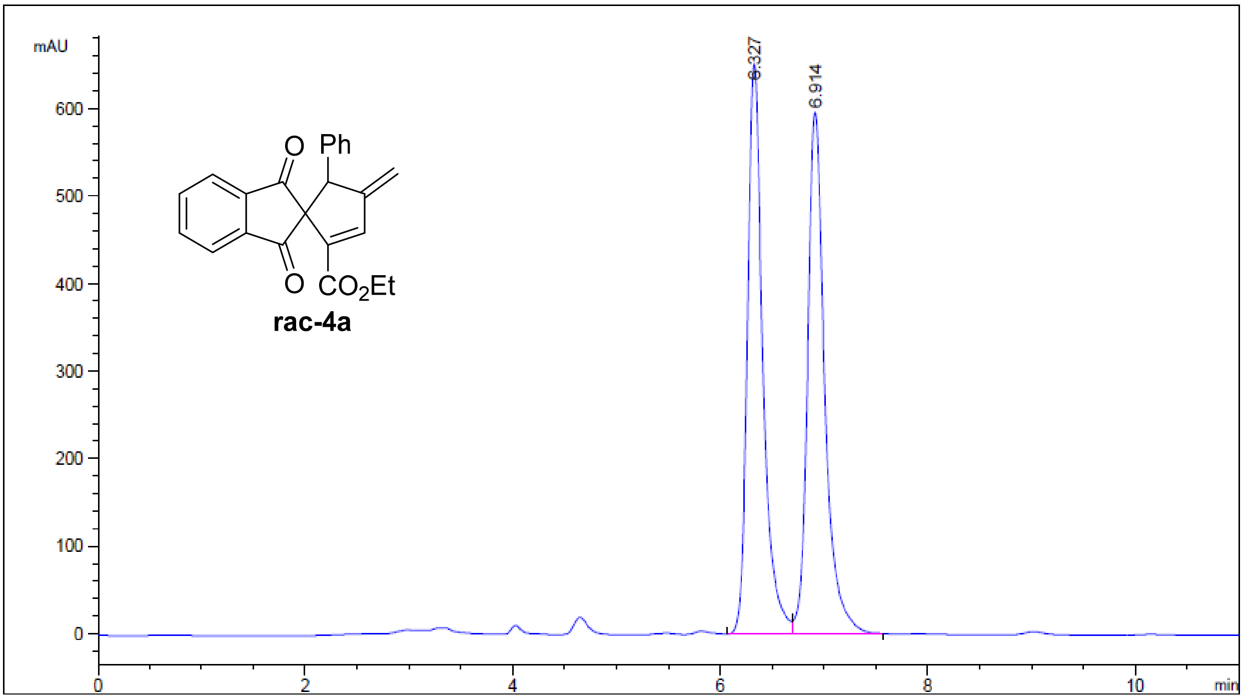

Peak #	RetTime [min]	Type	Width [min]	Area mAU *s	Height [mAU]	Area %
1	6.327	BV	0.1559	6753.32129	651.11517	49.5453
2	6.914	VBA	0.1724	6877.27197	596.25513	50.4547

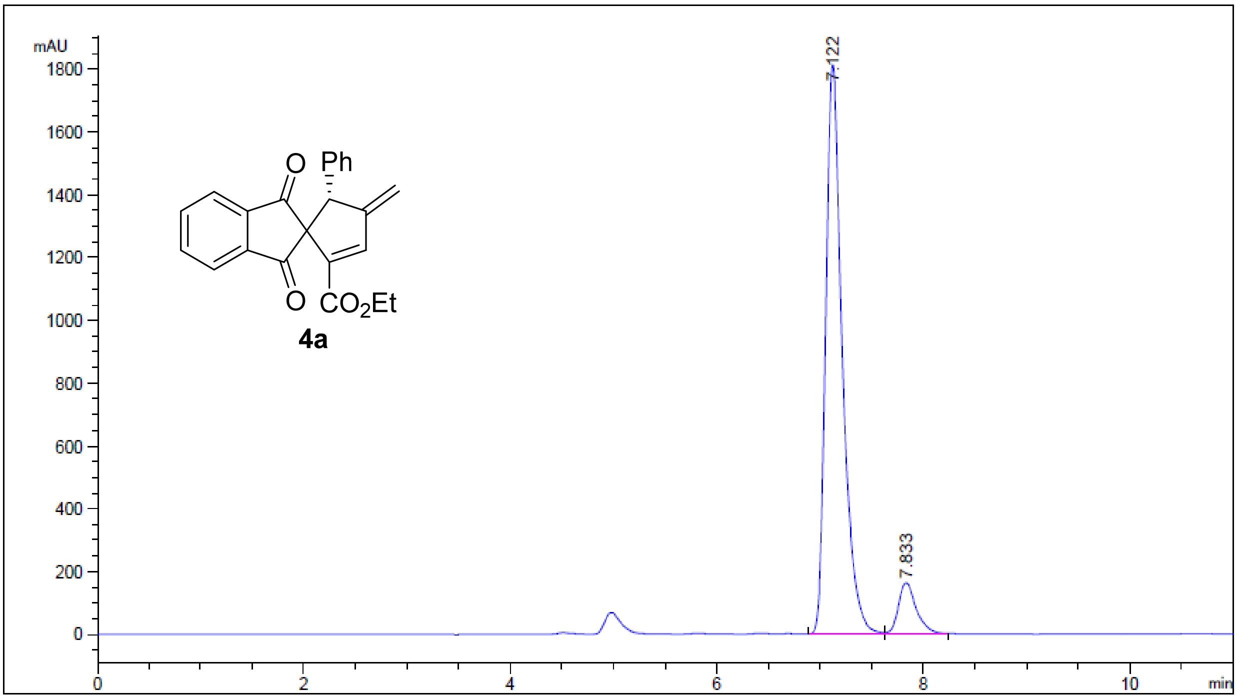

Peak #	RetTime [min]	Type	Width [min]	Area mAU *s	Height [mAU]	Area %
1	7.122	BV	0.1666	2.02430e4	1813.50012	91.0408
2	7.833	VBA	0.1827	1992.09583	163.70927	8.9592

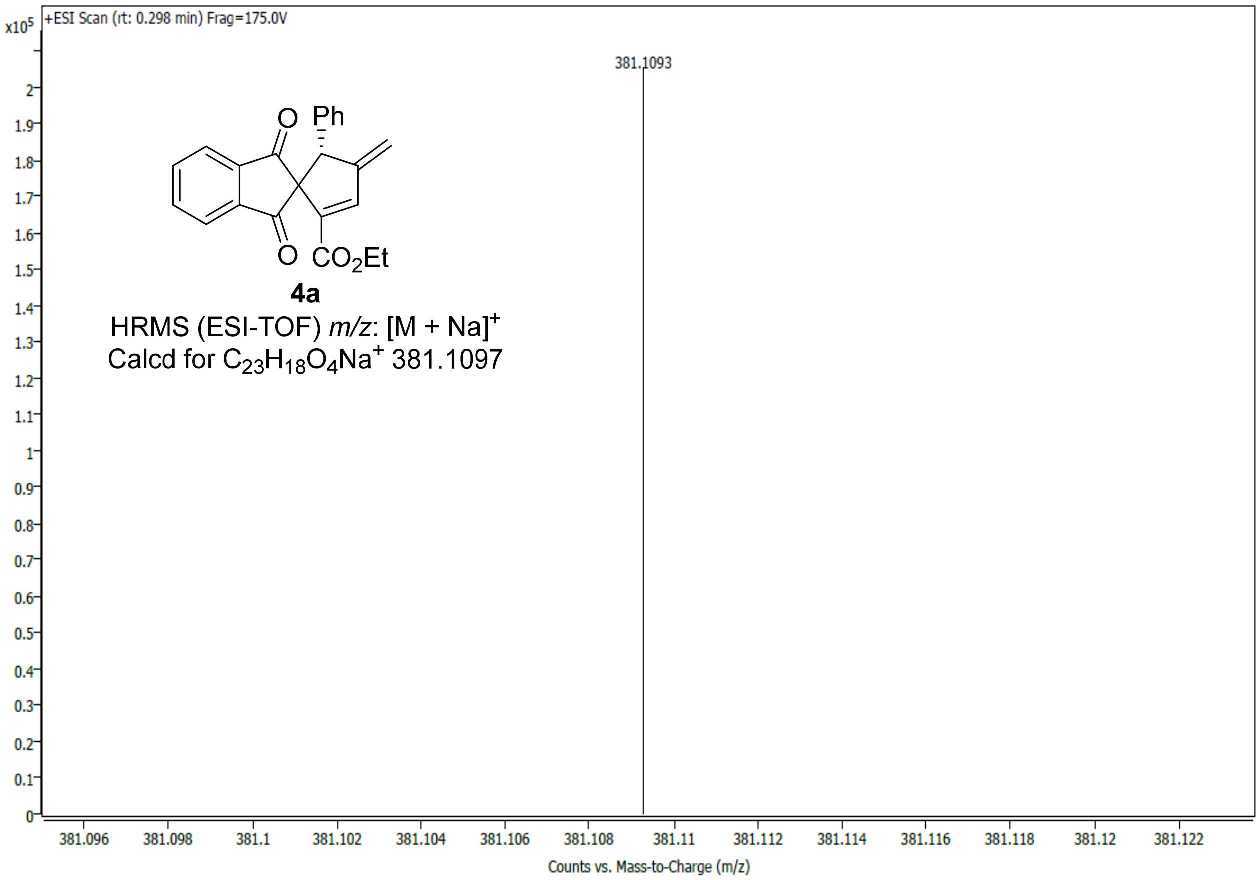

x10^5
+ESI Scan (rt: 0.298 min) Frag=175.0V
381.1093
4a
HRMS (ESI-TOF) m/z: [M + Na]+
Calcd for C23H18O4Na+ 381.1097
Counts vs. Mass-to-Charge (m/z)

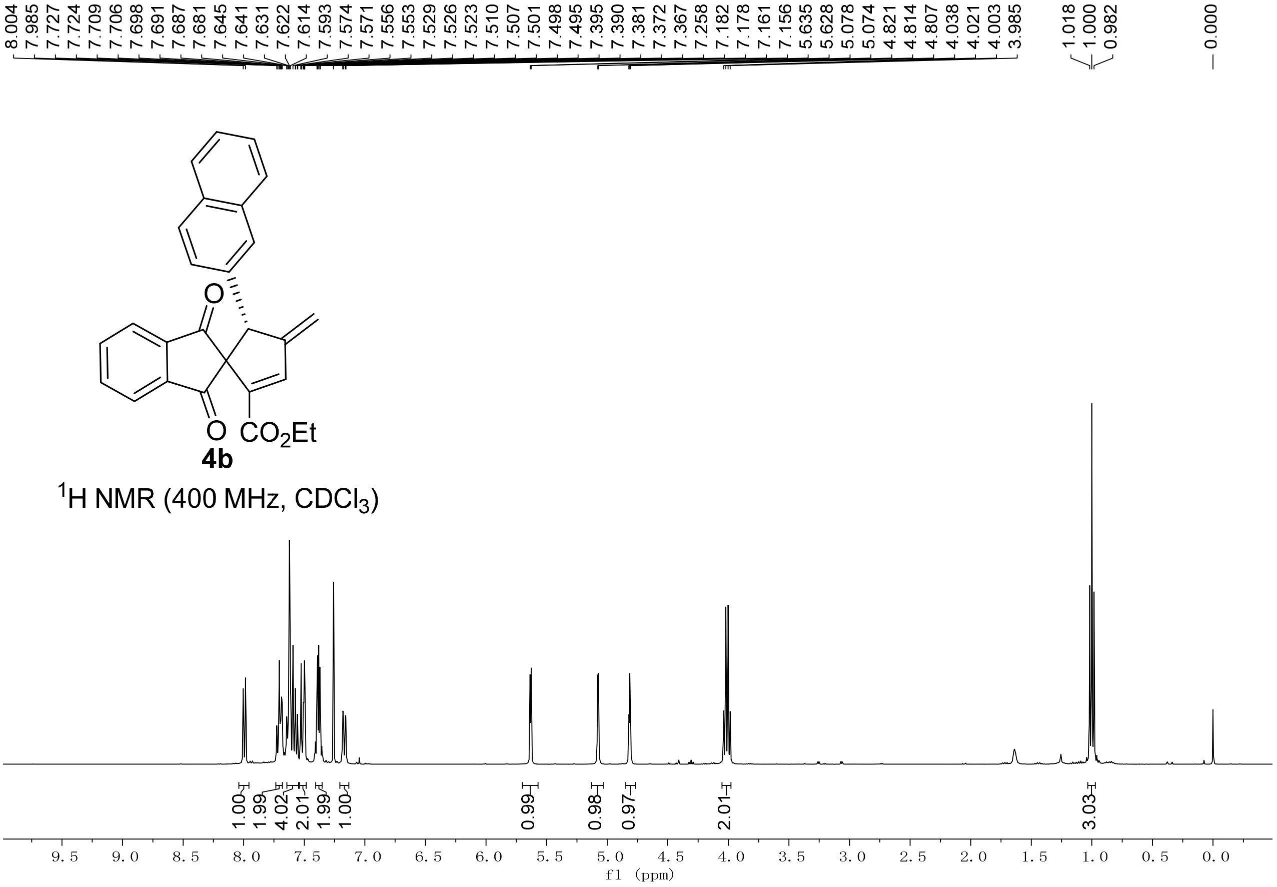
8.004 7.985 7.727 7.724 7.709 7.706 7.698 7.691 7.687 7.681 7.645 7.641 7.631 7.622 7.614 7.593 7.574 7.571 7.556 7.553 7.529 7.526 7.523 7.510 7.507 7.501 7.498 7.495 7.395 7.390 7.381 7.372 7.367 7.258 7.182 7.178 7.161 7.156 5.635 5.628 5.078 5.074 4.821 4.814 4.807 4.038 4.021 4.003 3.985
1.018 1.000 0.982
0.000
O
O
CO2Et
4b
1H NMR (400 MHz, CDCl3)
1.00 1.99 4.02 2.01 1.99 1.00 0.99 0.98 0.97 2.01 3.03
9.5 9.0 8.5 8.0 7.5 7.0 6.5 6.0 5.5 5.0 4.5 4.0 3.5 3.0 2.5 2.0 1.5 1.0 0.5 0.0
f1 (ppm)

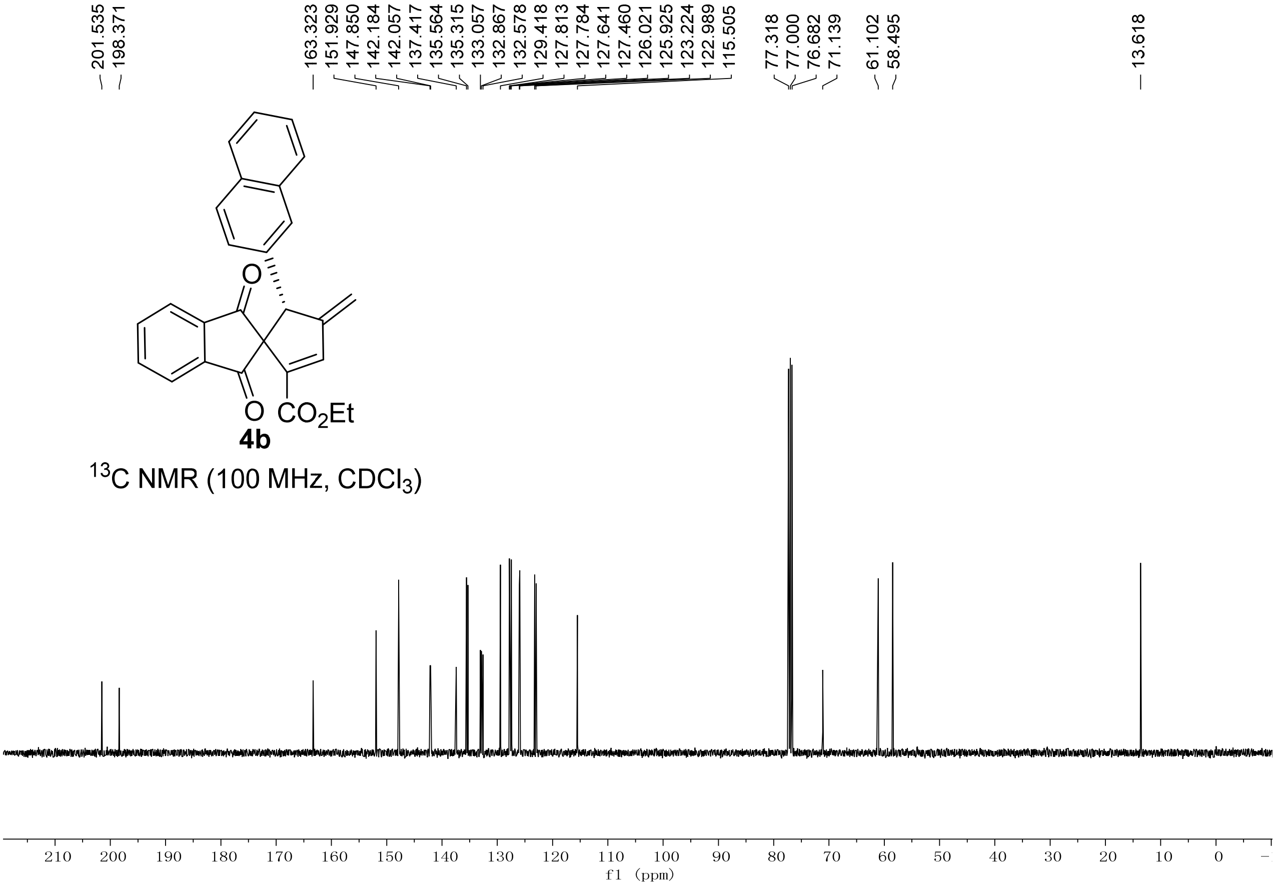
201.535 198.371
163.323 151.929 147.850 142.184 142.057 137.417 135.564 135.315 133.057 132.867 132.578 129.418 127.813 127.784 127.641 127.460 126.021 125.925 123.224 122.989 115.505
77.318 77.000 76.682 71.139
61.102 58.495
13.618
O
O
CO2Et
4b
13C NMR (100 MHz, CDCl3)
210 200 190 180 170 160 150 140 130 120 110 100 90 80 70 60 50 40 30 20 10 0
f1 (ppm)

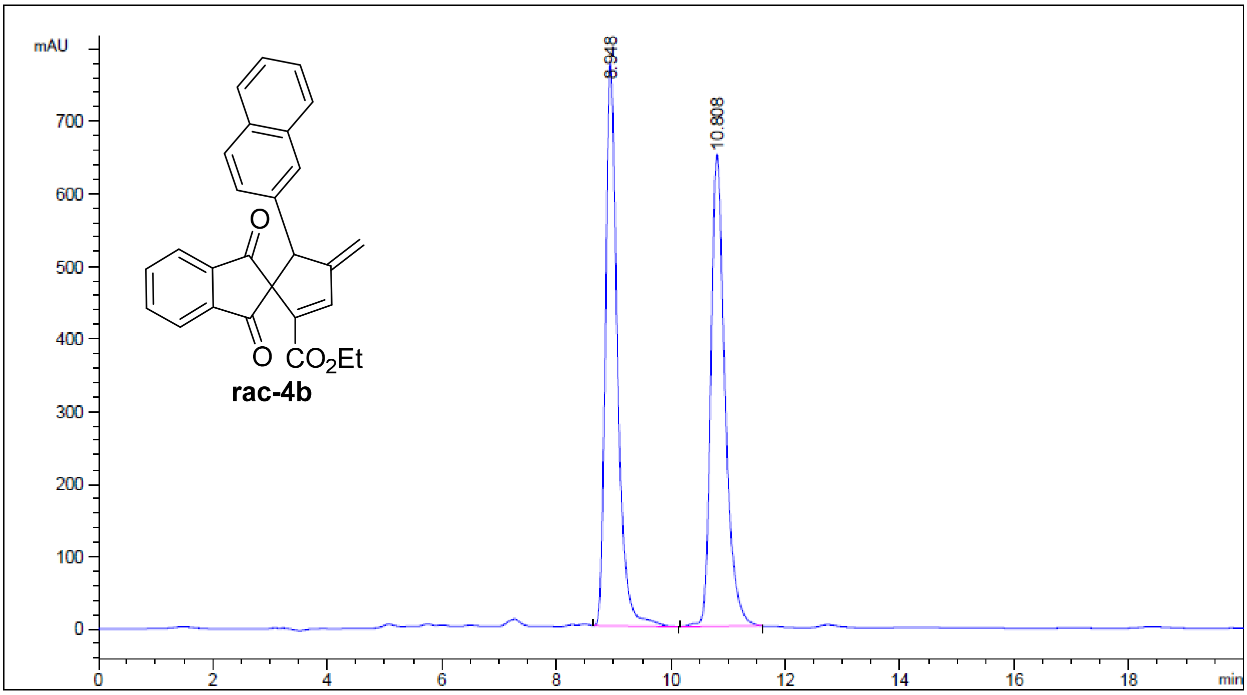

Peak #	RetTime [min]	Type	Width [min]	Area mAU *s	Height [mAU]	Area %
1	8.948	BB	0.2198	1.13052e4	774.63385	50.3223
2	10.808	BBA	0.2586	1.11604e4	649.79645	49.6777

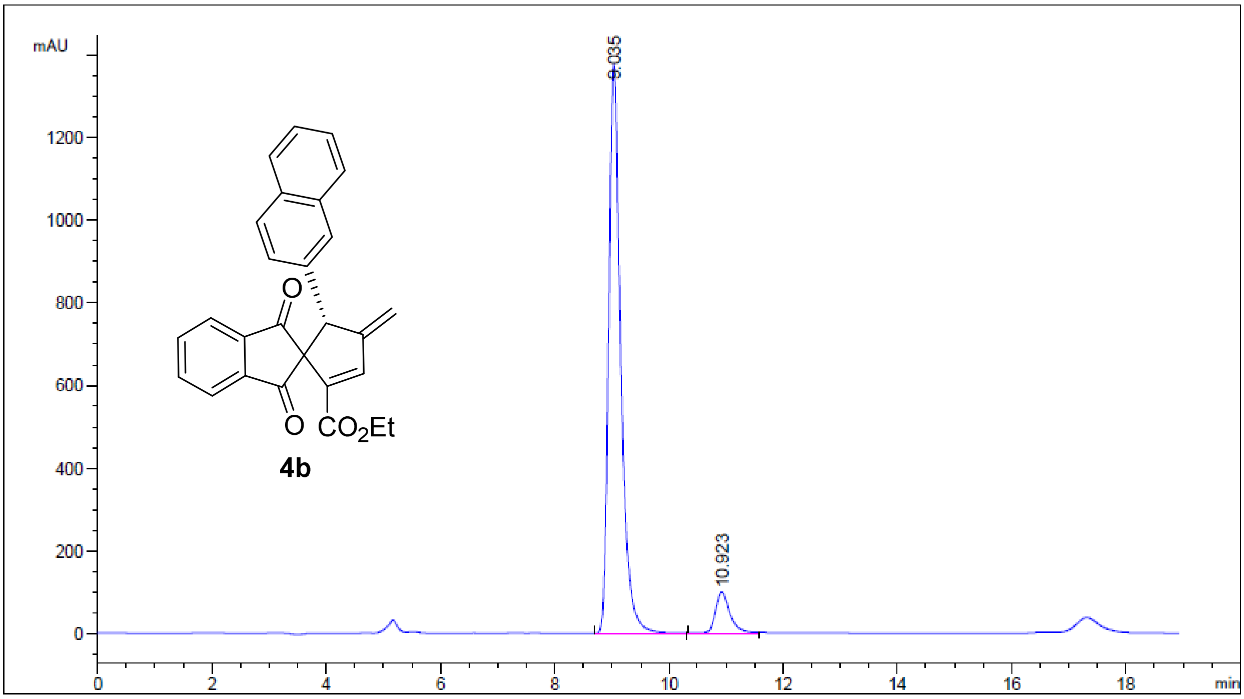

Peak #	RetTime [min]	Type	Width [min]	Area mAU *s	Height [mAU]	Area %
1	9.035	BB	0.2201	2.01459e4	1377.90808	91.9140
2	10.923	BBA	0.2679	1772.31653	99.29834	8.0860

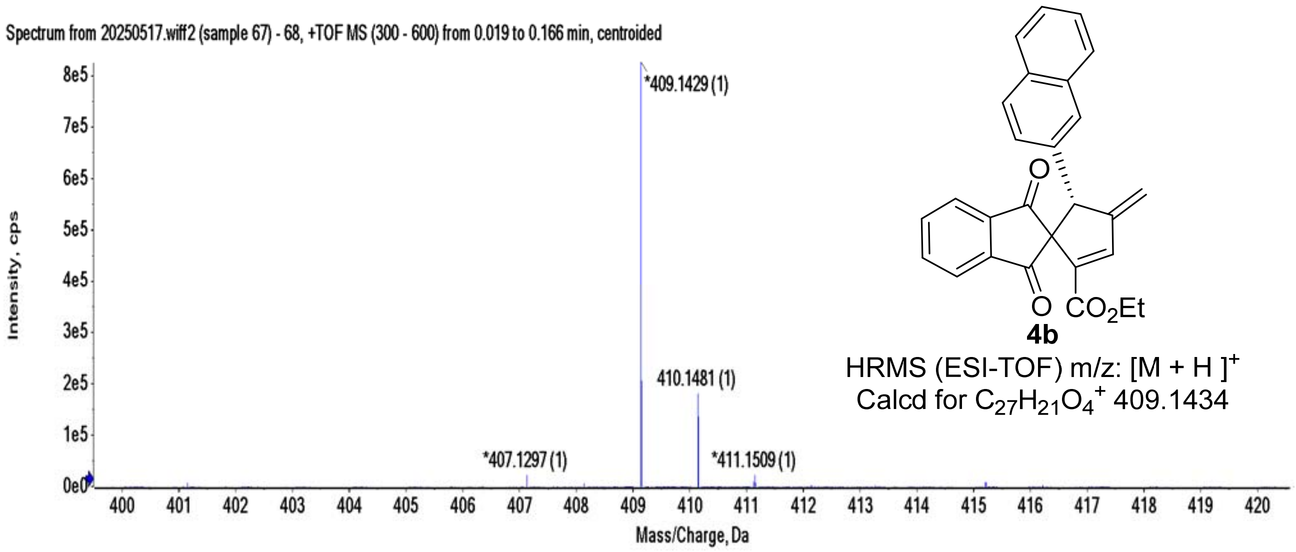

4b

HRMS (ESI-TOF) m/z: [M + H]$^+$

Calcd for $C_{27}H_{21}O_4^+$ 409.1434

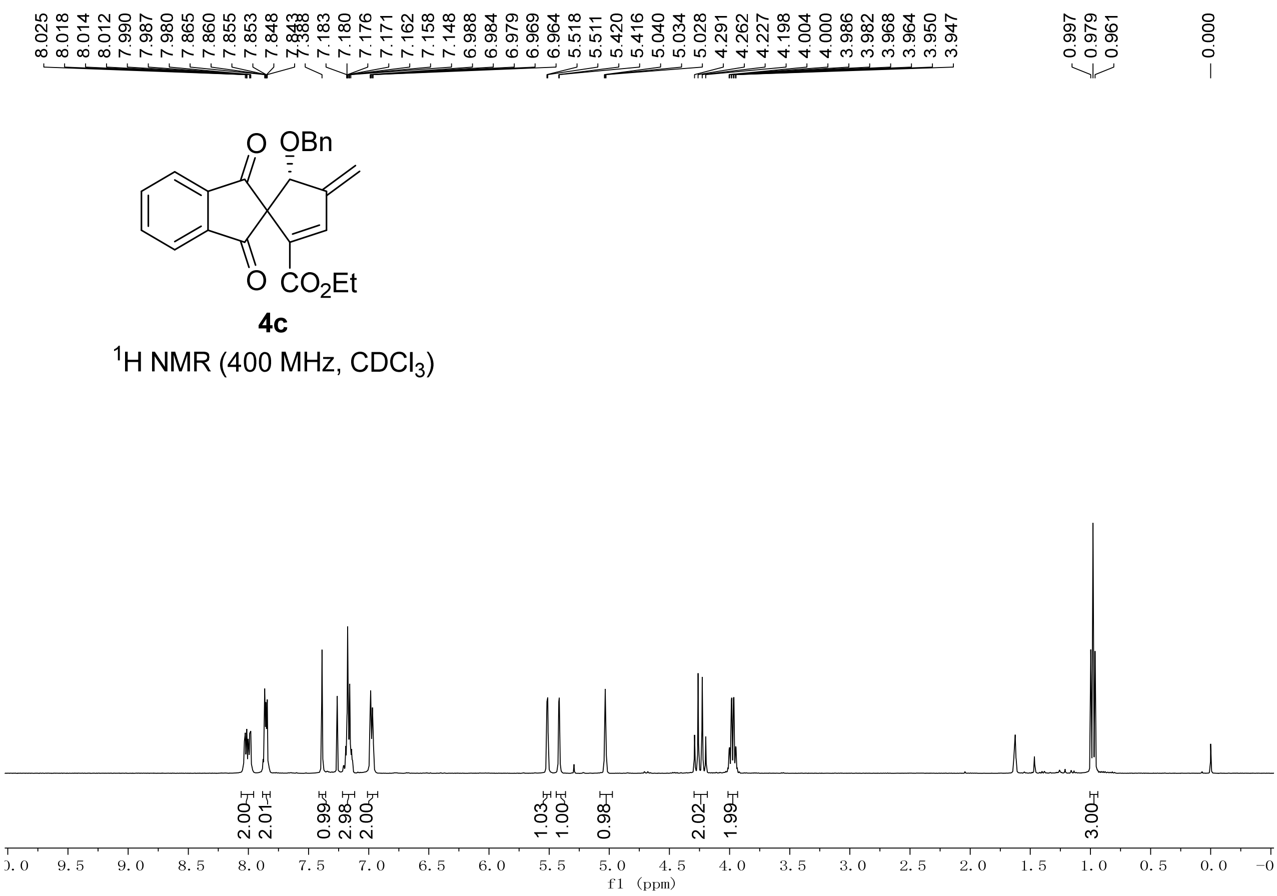
O OBn
O CO2Et
4c
1H NMR (400 MHz, CDCl3)
f1 (ppm)

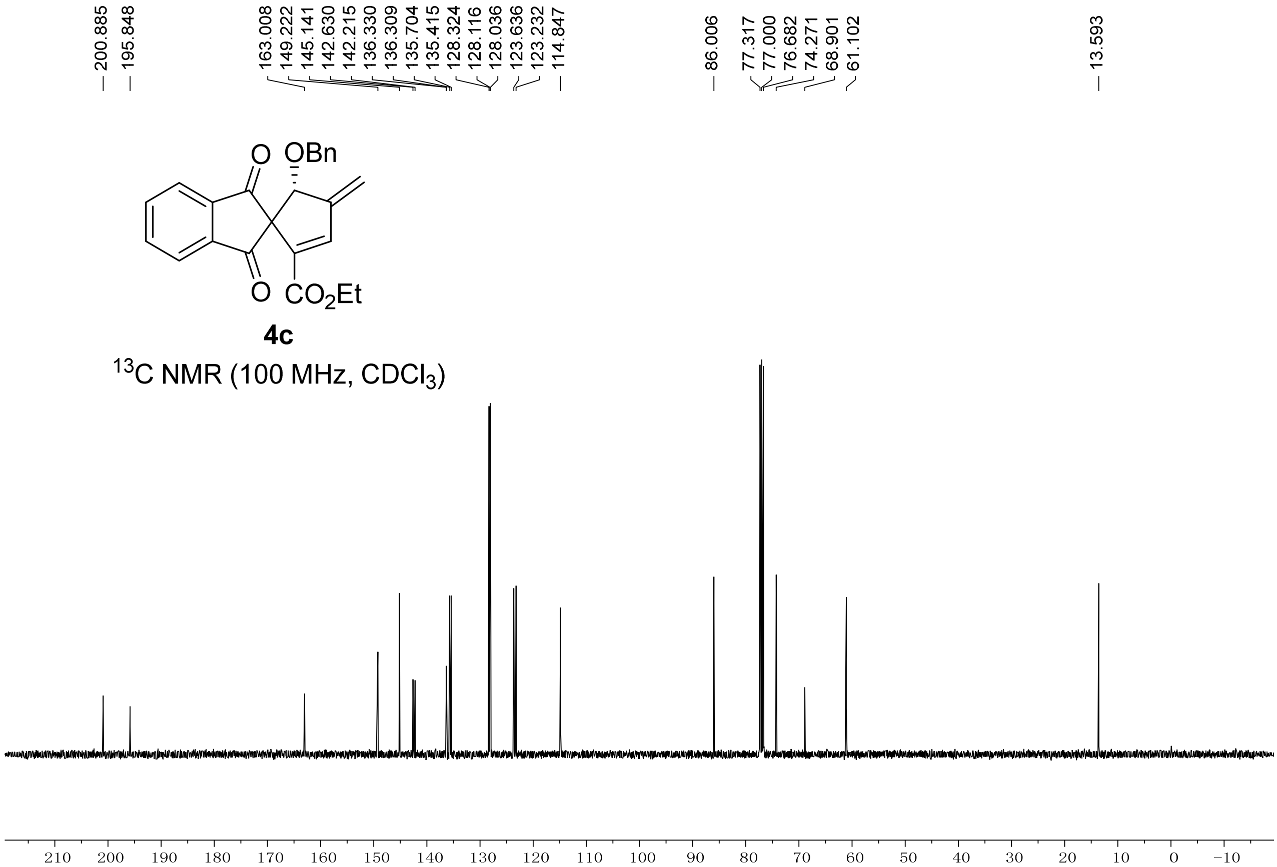
O OBn
O CO2Et
4c
13C NMR (100 MHz, CDCl3)
f1 (ppm)

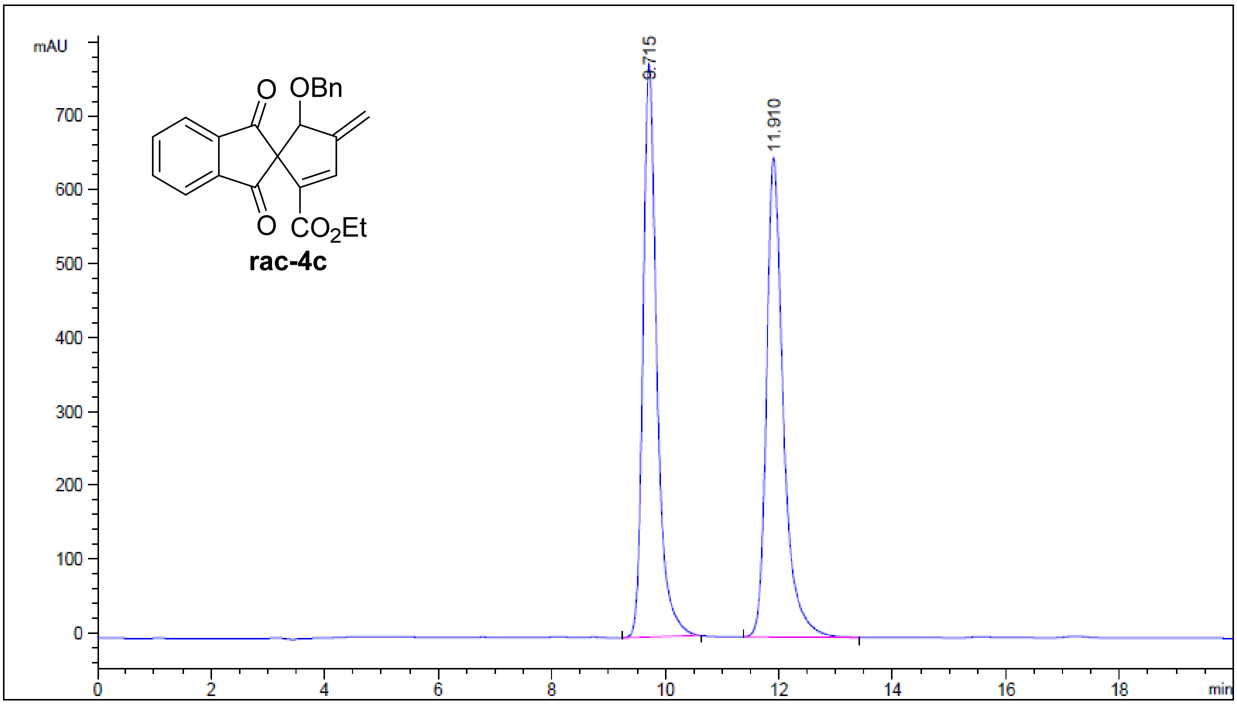

Peak #	RetTime [min]	Type	Width [min]	Area mAU *s	Height [mAU]	Area %
1	9.715	BBA	0.2566	1.31689e4	774.42975	49.4823
2	11.910	BBA	0.3117	1.34445e4	648.74408	50.5177

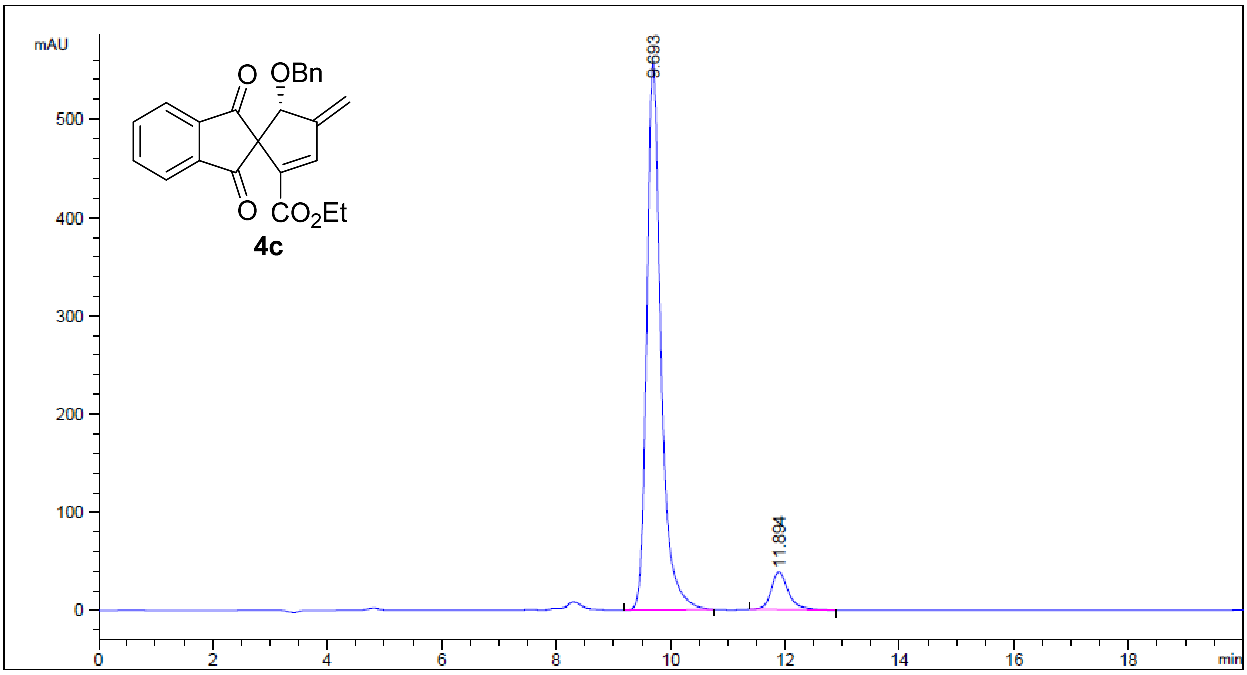

Peak #	RetTime [min]	Type	Width [min]	Area mAU *s	Height [mAU]	Area %
1	9.693	BBA	0.2605	9649.27930	556.66052	92.4675
2	11.894	BBA	0.3095	786.03949	38.26927	7.5325

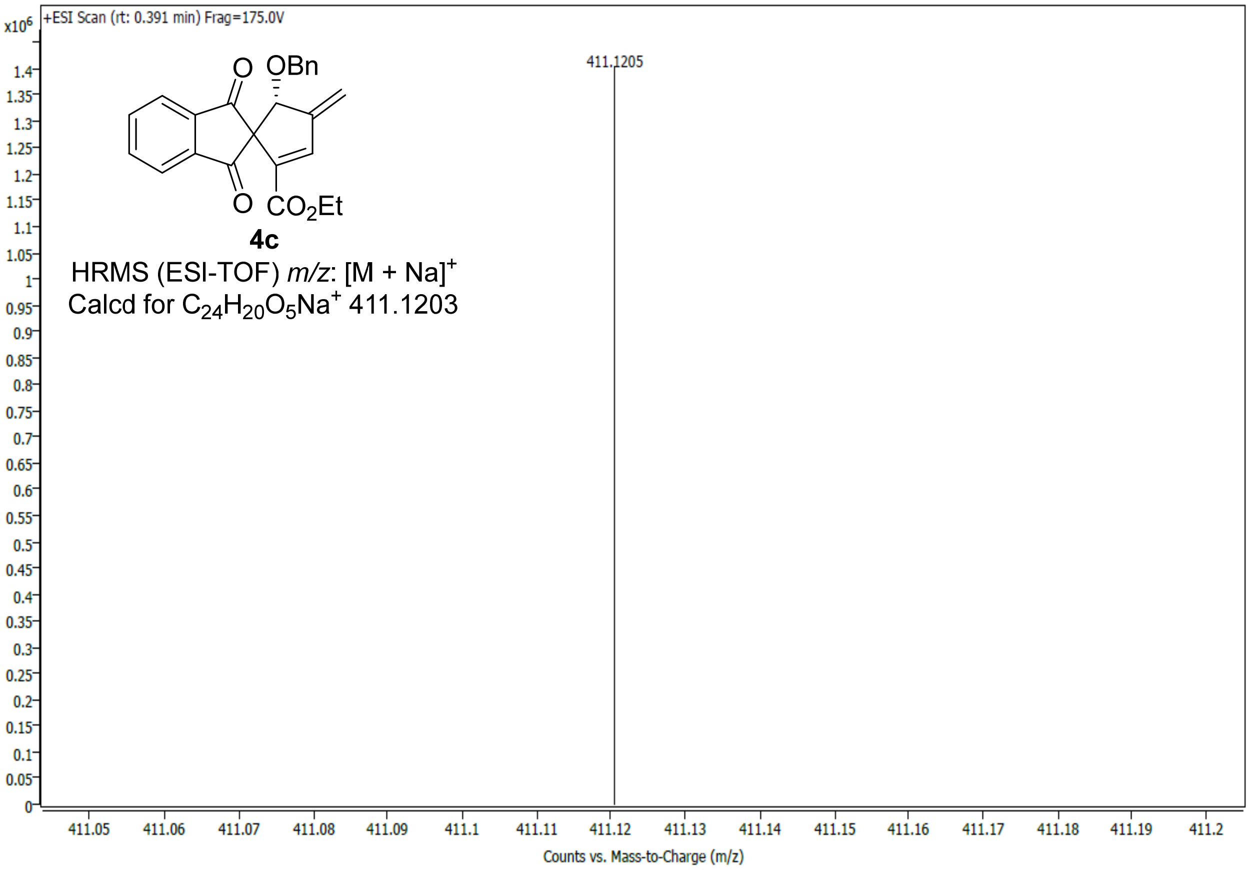
+ESI Scan (rt: 0.391 min) Frag=175.0V
x10^6
411.1205
O
OBn
O
CO2Et
4c
HRMS (ESI-TOF) m/z: [M + Na]+
Calcd for C24H20O5Na+ 411.1203
1.4
1.35
1.3
1.25
1.2
1.15
1.1
1.05
1
0.95
0.9
0.85
0.8
0.75
0.7
0.65
0.6
0.55
0.5
0.45
0.4
0.35
0.3
0.25
0.2
0.15
0.1
0.05
0
411.05
411.06
411.07
411.08
411.09
411.1
411.11
411.12
411.13
411.14
411.15
411.16
411.17
411.18
411.19
411.2
Counts vs. Mass-to-Charge (m/z)

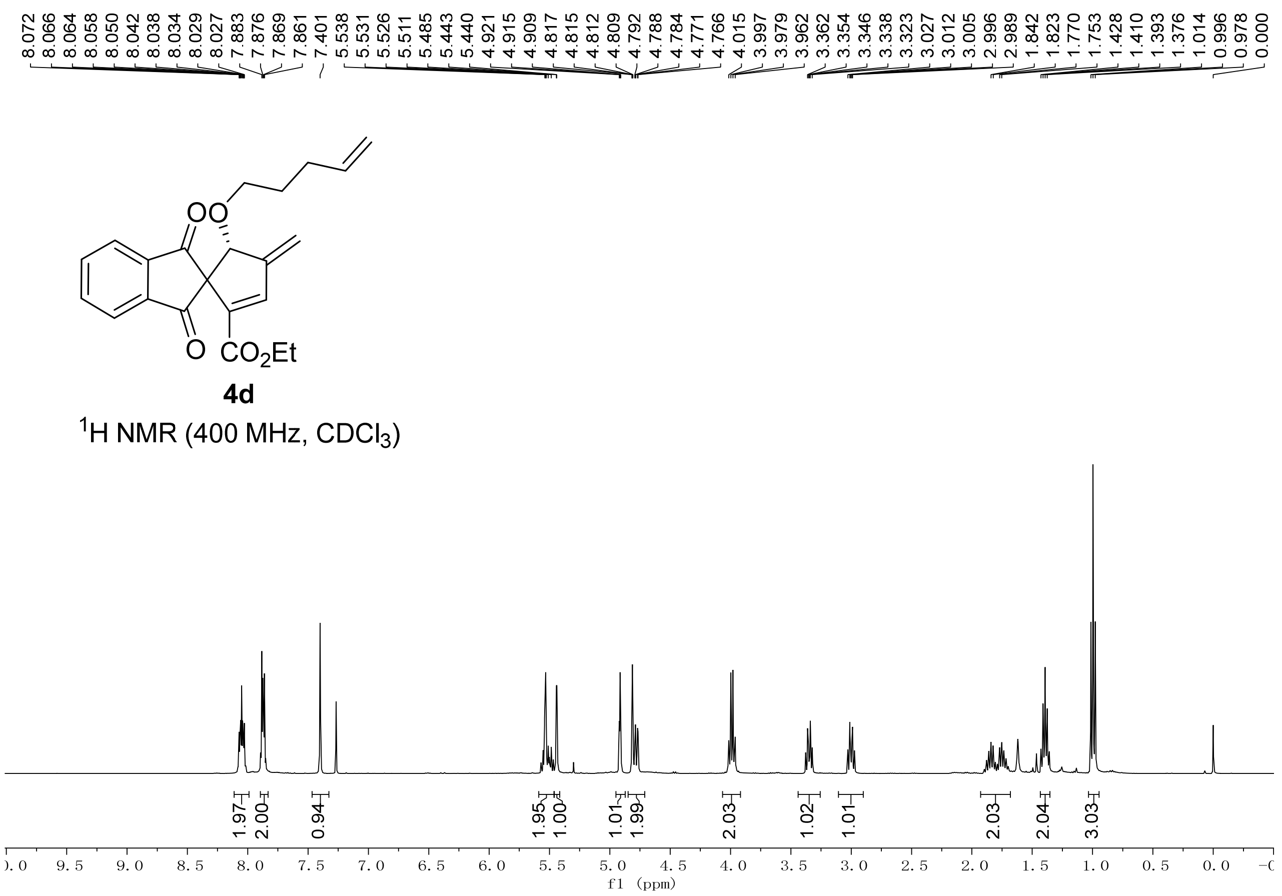
8.072 8.066 8.064 8.058 8.050 8.042 8.038 8.034 8.029 8.027 7.883 7.876 7.869 7.861 7.401 5.538 5.531 5.526 5.511 5.485 5.443 5.440 4.921 4.915 4.909 4.817 4.815 4.812 4.809 4.792 4.788 4.784 4.771 4.766 4.015 3.997 3.979 3.962 3.362 3.354 3.346 3.338 3.323 3.027 3.012 3.005 2.996 2.989 1.842 1.823 1.770 1.753 1.428 1.410 1.393 1.376 1.014 0.996 0.978 0.000
O
O
O
CO2Et
4d
1H NMR (400 MHz, CDCl3)
1.97 2.00 0.94 1.95 1.00 1.01 1.99 2.03 1.02 1.01 2.03 2.04 3.03
f1 (ppm)

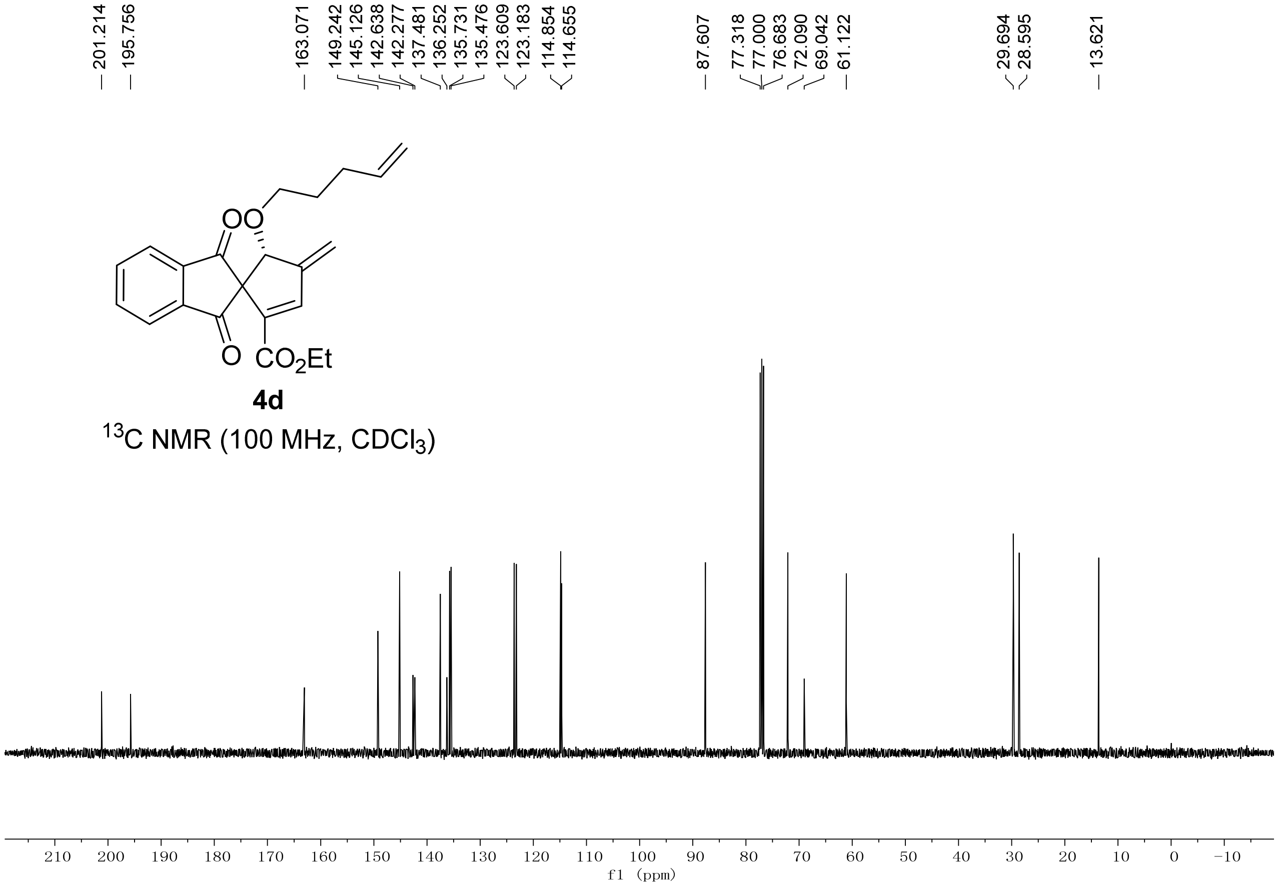
201.214 195.756 163.071 149.242 145.126 142.638 142.277 137.481 136.252 135.731 135.476 123.609 123.183 114.854 114.655 87.607 77.318 77.000 76.683 72.090 69.042 61.122 29.694 28.595 13.621
O
O
O
CO2Et
4d
13C NMR (100 MHz, CDCl3)
f1 (ppm)

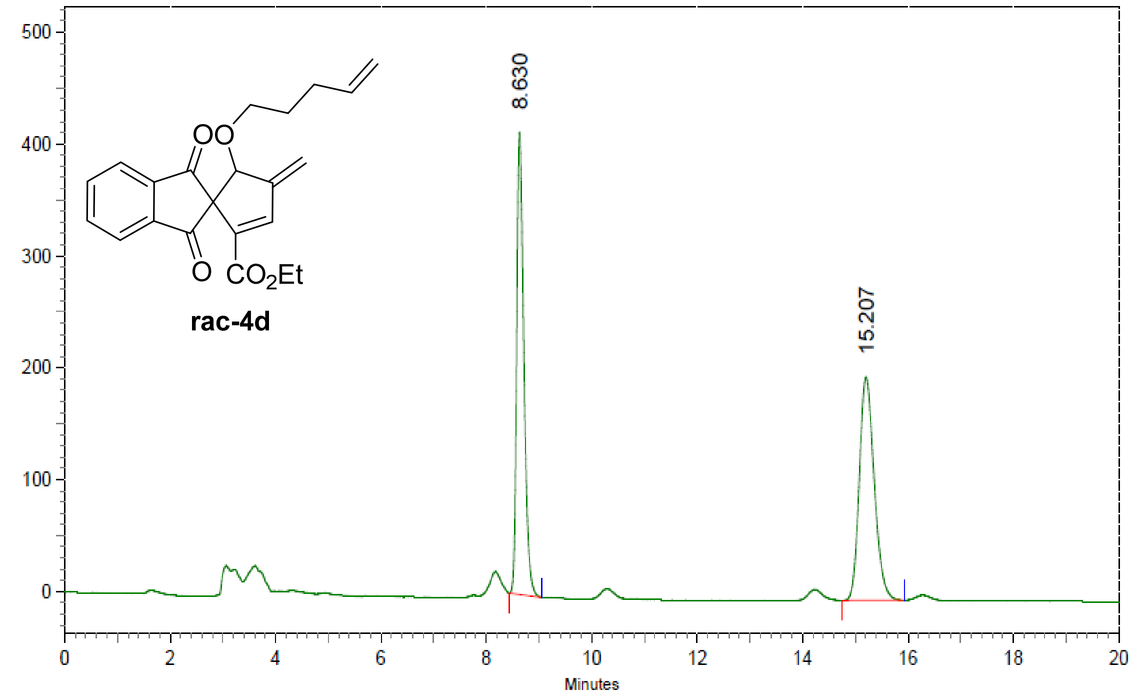

Peak No.	Ret Time	Width	Height	Area	Area [%]
1	8.630	0.600	6932391	65276854	49.8499
2	15.207	1.163	3348311	65669883	50.1501

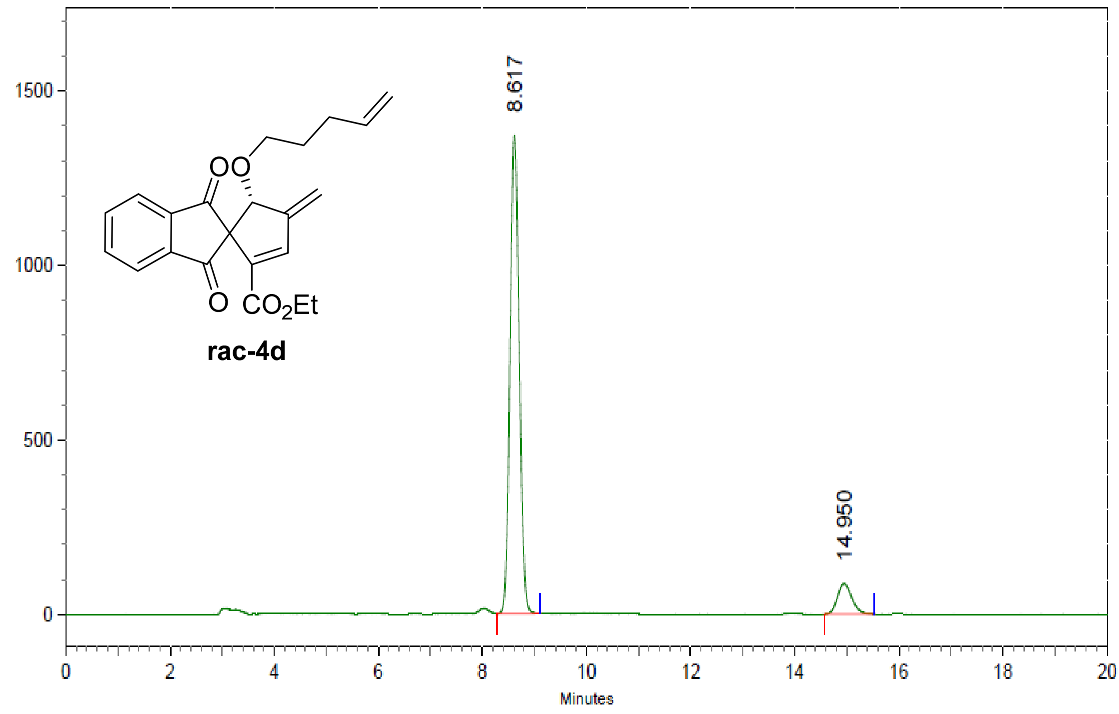

Peak No.	Ret Time	Width	Height	Area	Area [%]
1	8.617	0.827	22987753	285310827	90.9493
2	14.950	0.973	1472419	28392361	9.0507

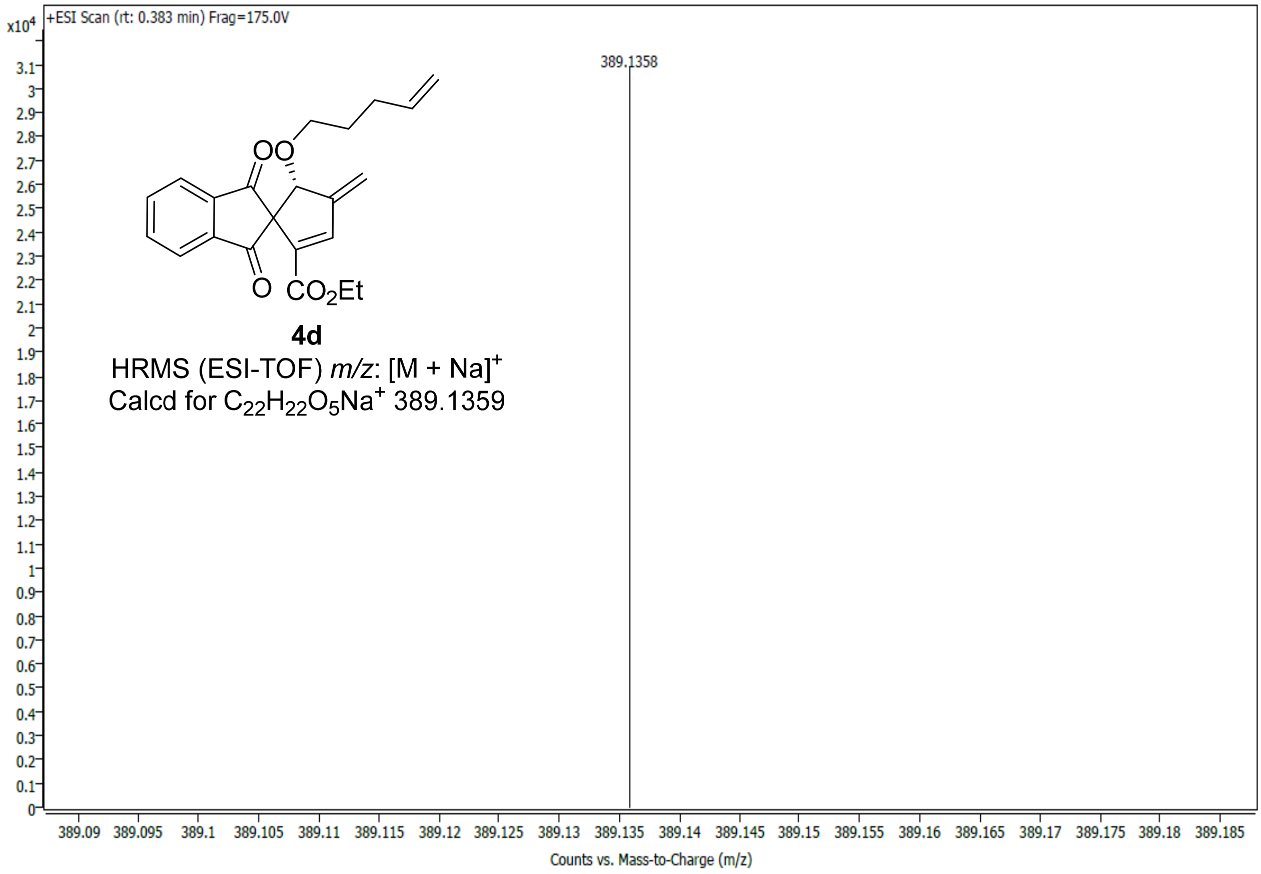
+ESI Scan (rt: 0.383 min) Frag=175.0V
x10^4
389.1358
CO2Et
4d
HRMS (ESI-TOF) m/z: [M + Na]+
Calcd for C22H22O5Na+ 389.1359
Counts vs. Mass-to-Charge (m/z)

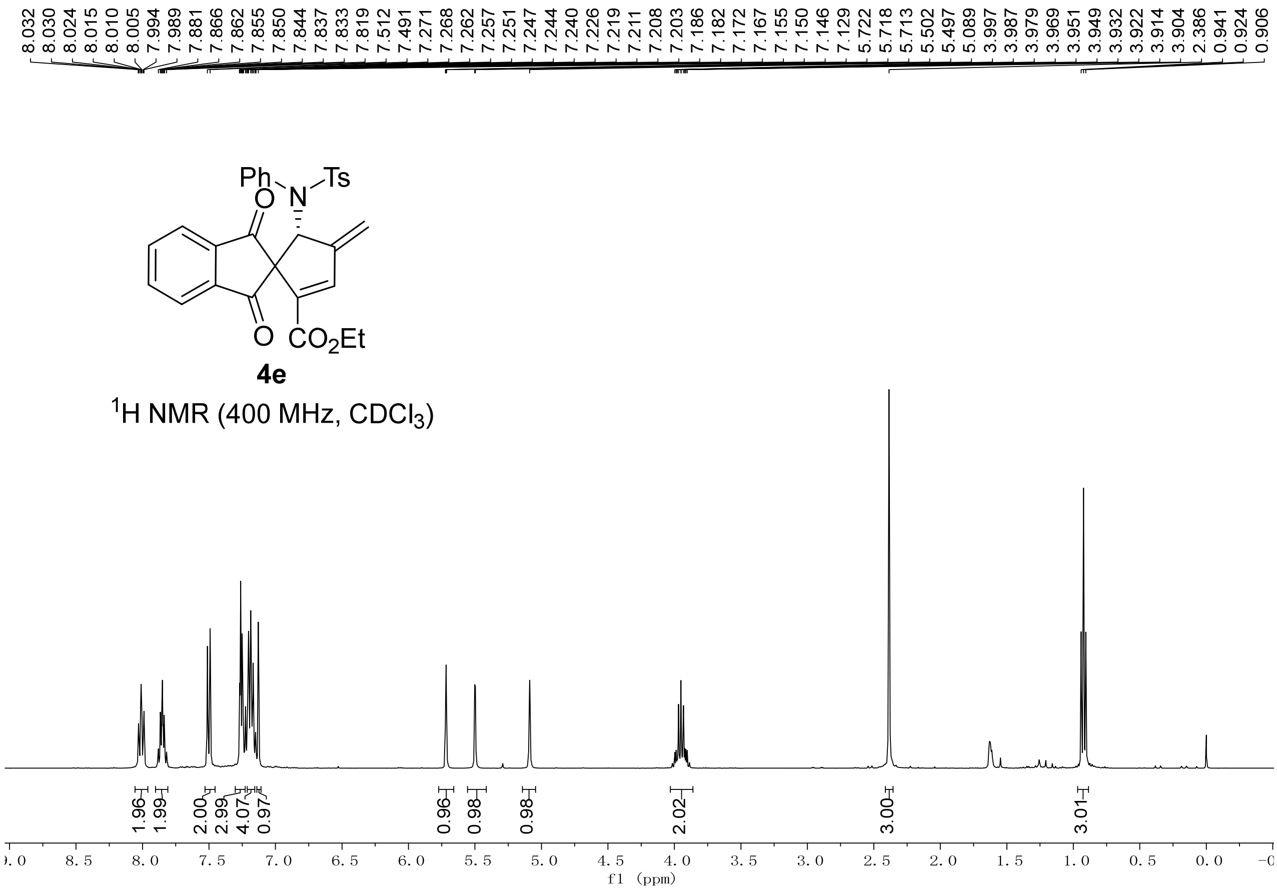

4e

^{1}H NMR (400 MHz, $CDCl_3$)

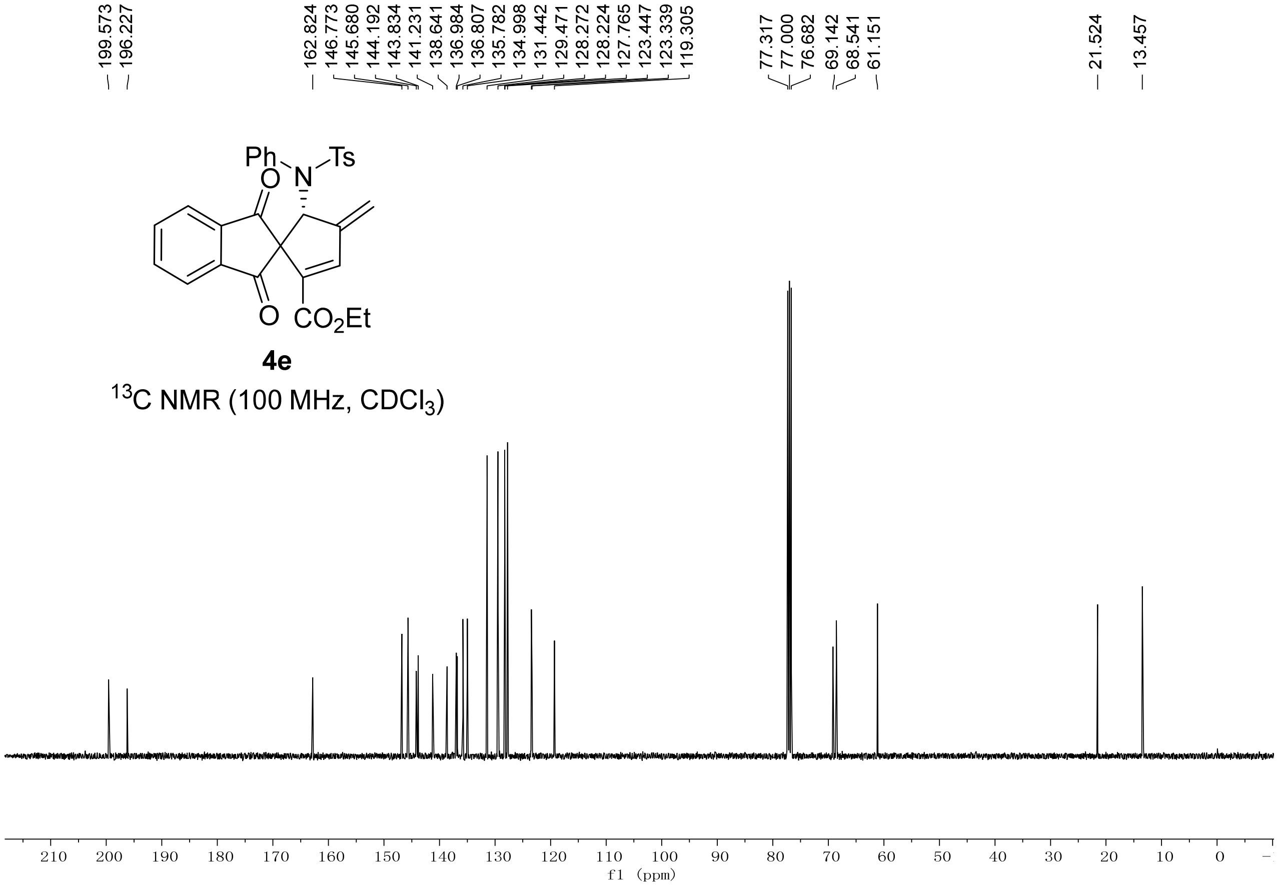

4e

^{13}C NMR (100 MHz, $CDCl_3$)

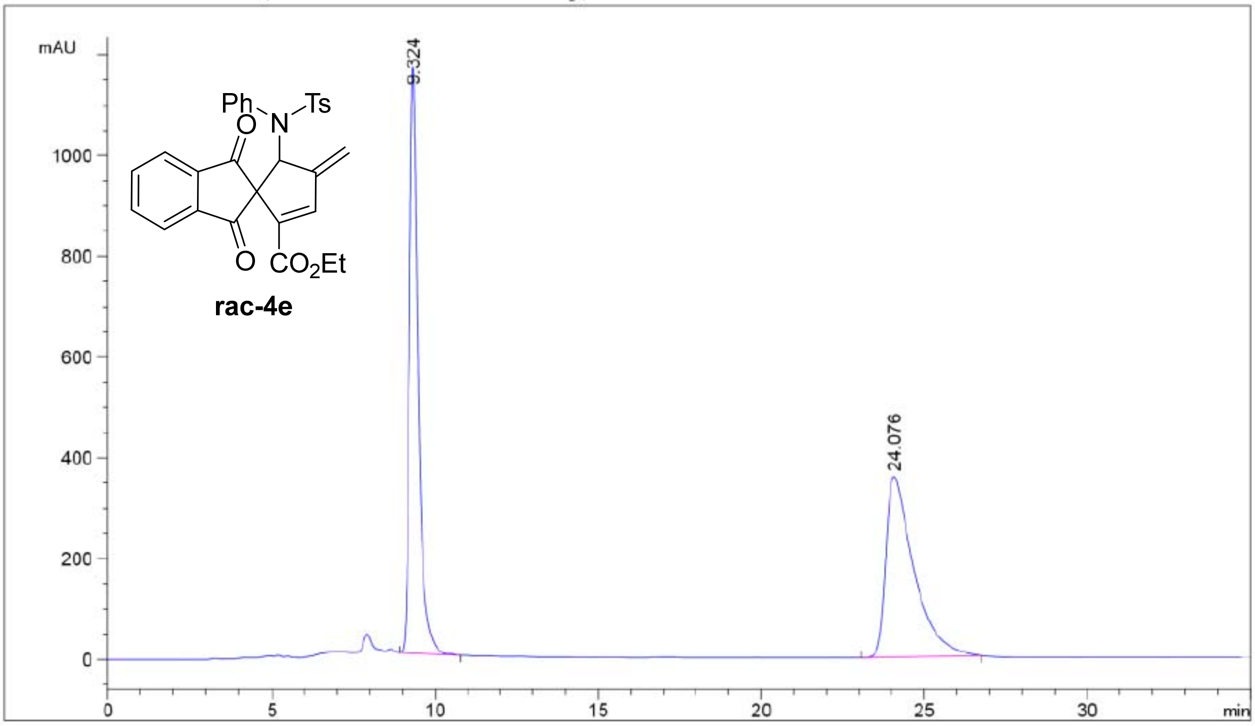

Peak #	RetTime [min]	Type	Width [min]	Area mAU *s	Height [mAU]	Area %
1	9.324	BBA	0.2799	2.16697e4	1163.68530	50.3707
2	24.076	BBA	0.8713	2.13508e4	358.36749	49.6293

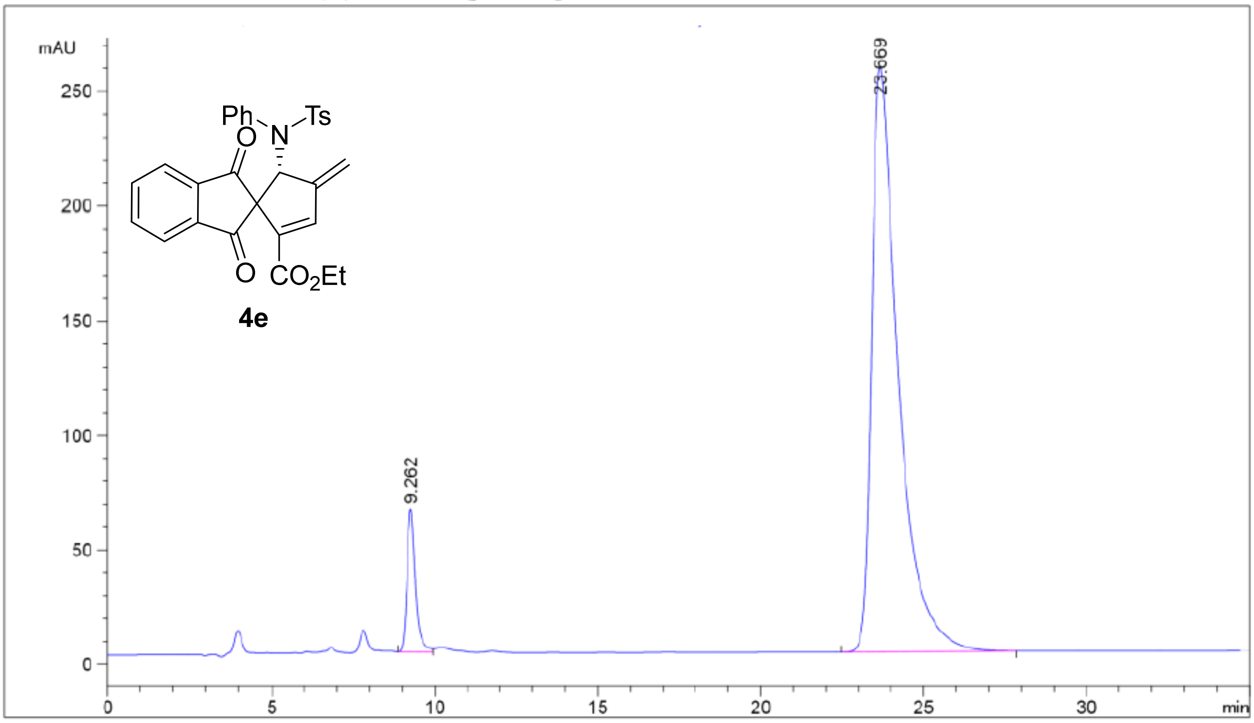

Peak #	RetTime [min]	Type	Width [min]	Area mAU *s	Height [mAU]	Area %
1	9.262	BV	0.2731	1124.11572	62.29654	7.2036
2	23.669	BB	0.8330	1.44808e4	254.96054	92.7964

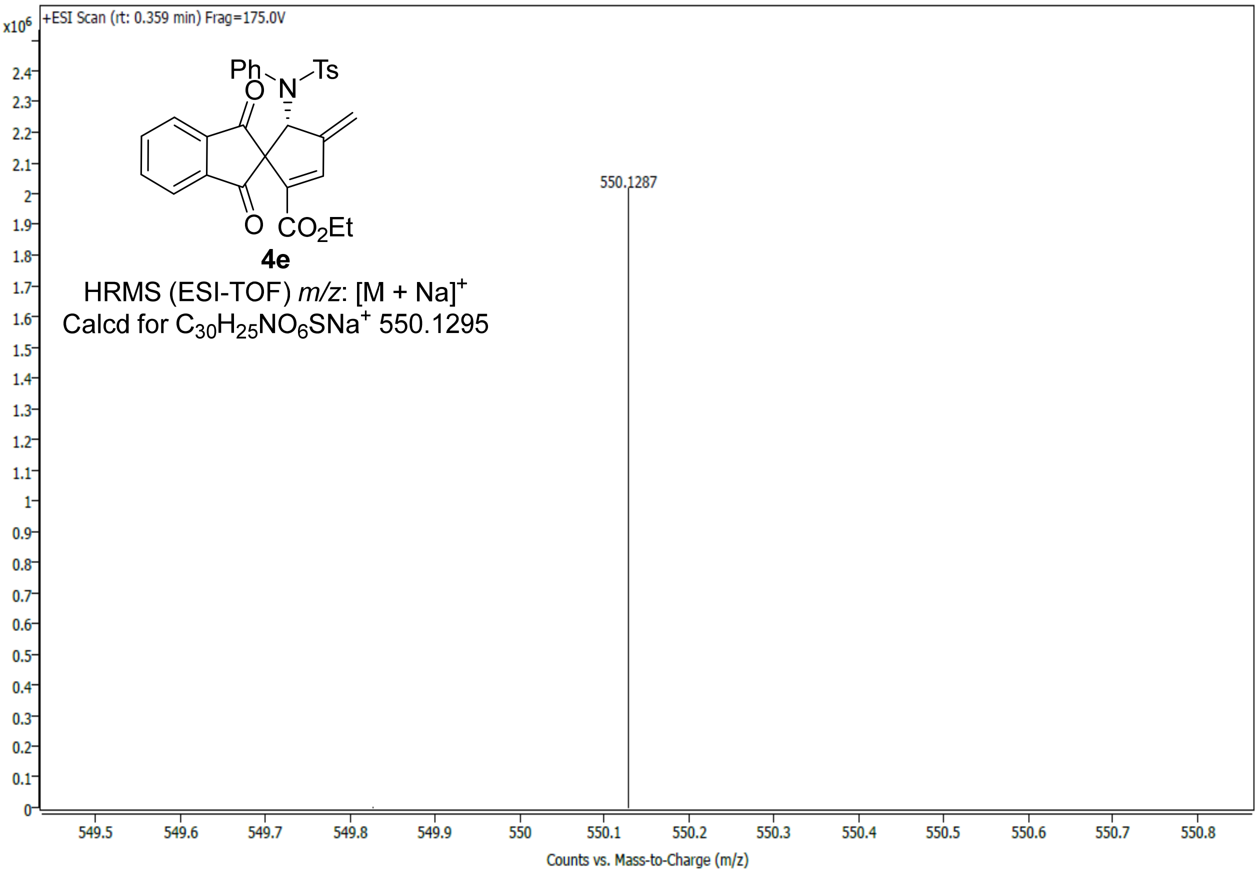

+ESI Scan (rt: 0.359 min) Frag=175.0V
x10^6
550.1287
4e
HRMS (ESI-TOF) m/z: [M + Na]+
Calcd for C30H25NO6SNa+ 550.1295
Counts vs. Mass-to-Charge (m/z)

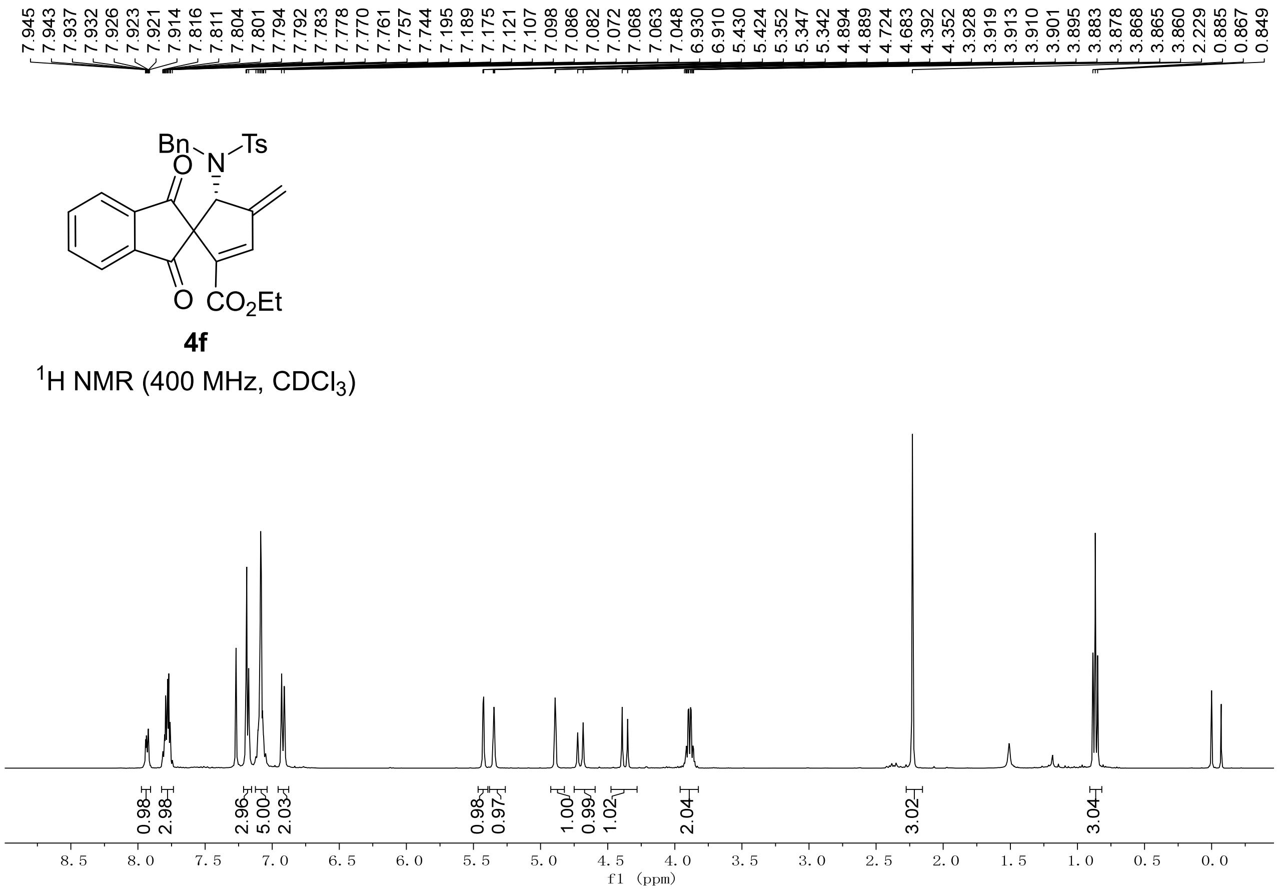

4f

^{1}H NMR (400 MHz, $CDCl_3$)

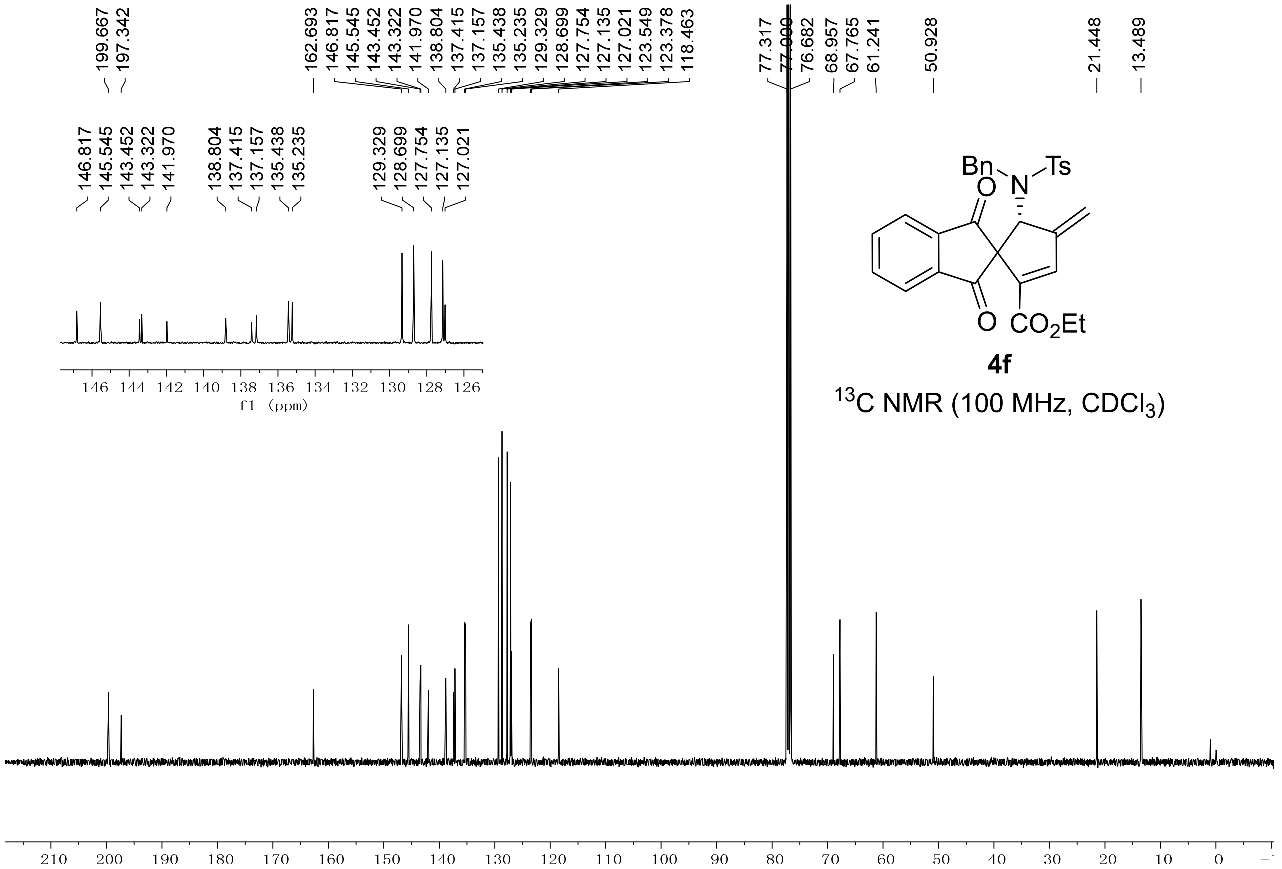

4f

^{13}C NMR (100 MHz, $CDCl_3$)

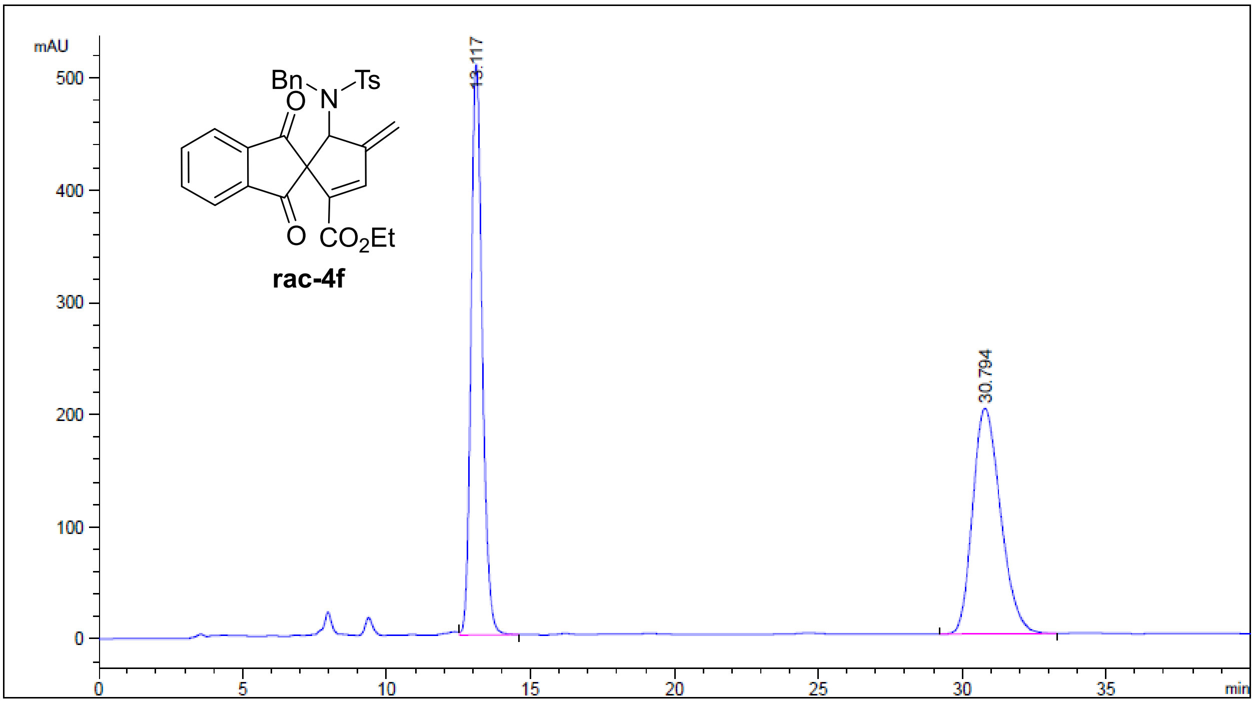

Peak #	RetTime [min]	Type	Width [min]	Area mAU *s	Height [mAU]	Area %
1	13.117	VB	0.4157	1.37878e4	507.99231	50.2938
2	30.794	BB	1.0463	1.36268e4	201.23254	49.7062

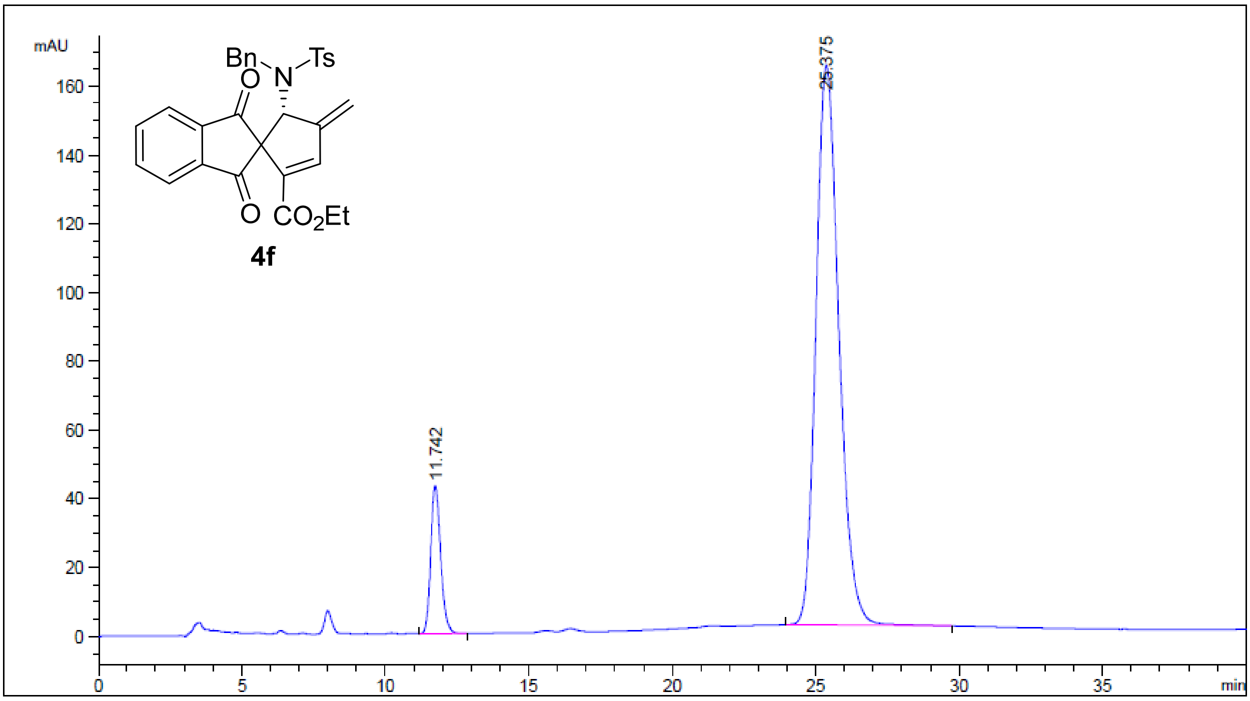

Peak #	RetTime [min]	Type	Width [min]	Area mAU *s	Height [mAU]	Area %
1	11.742	BB	0.3687	1030.98132	42.97864	10.2101
2	25.375	BB	0.8622	9066.66699	162.82646	89.7899

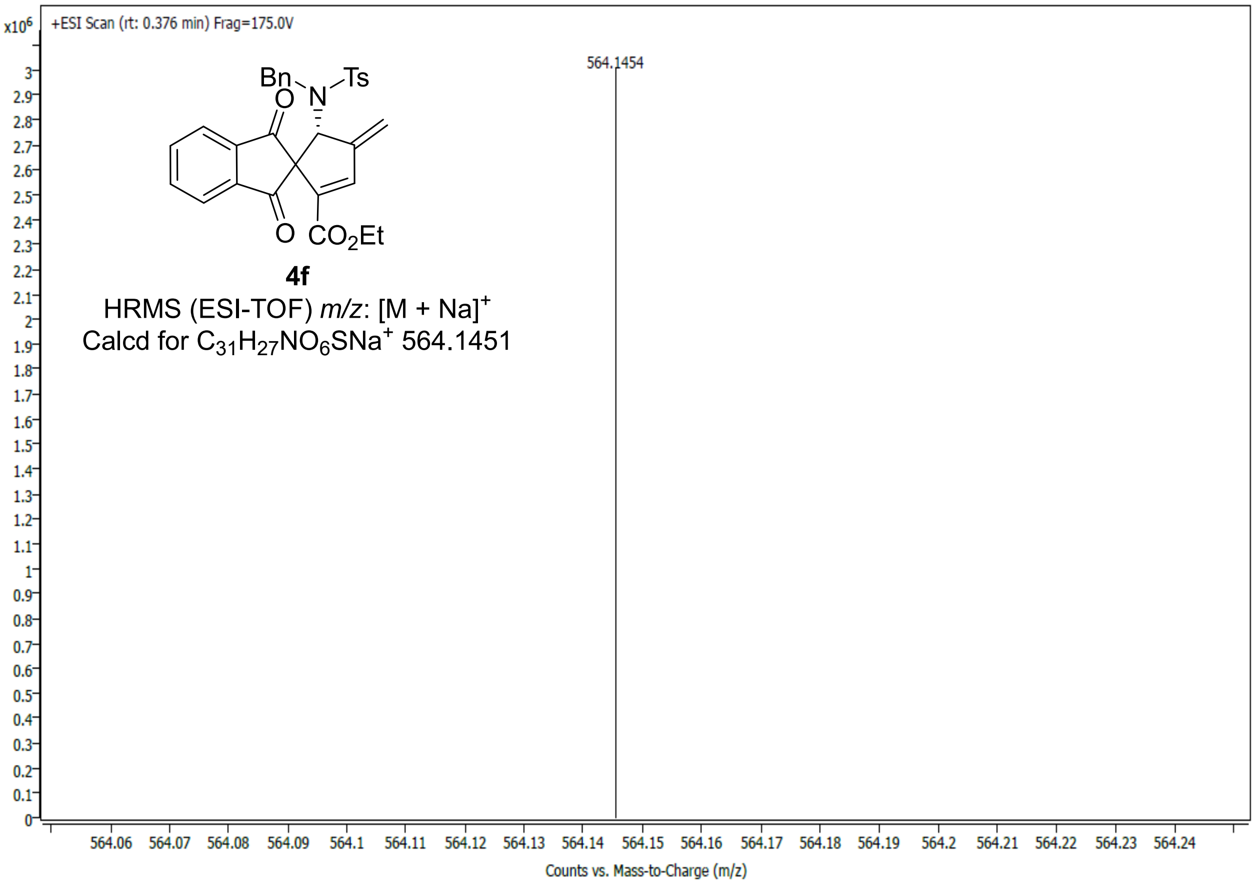
x10^6
+ESI Scan (rt: 0.376 min) Frag=175.0V
564.1454
Bn
O
N
Ts
O
O
CO2Et
4f
HRMS (ESI-TOF) m/z: [M + Na]+
Calcd for C31H27NO6SNa+ 564.1451
3
2.9
2.8
2.7
2.6
2.5
2.4
2.3
2.2
2.1
2
1.9
1.8
1.7
1.6
1.5
1.4
1.3
1.2
1.1
1
0.9
0.8
0.7
0.6
0.5
0.4
0.3
0.2
0.1
0
564.06
564.07
564.08
564.09
564.1
564.11
564.12
564.13
564.14
564.15
564.16
564.17
564.18
564.19
564.2
564.21
564.22
564.23
564.24
Counts vs. Mass-to-Charge (m/z)

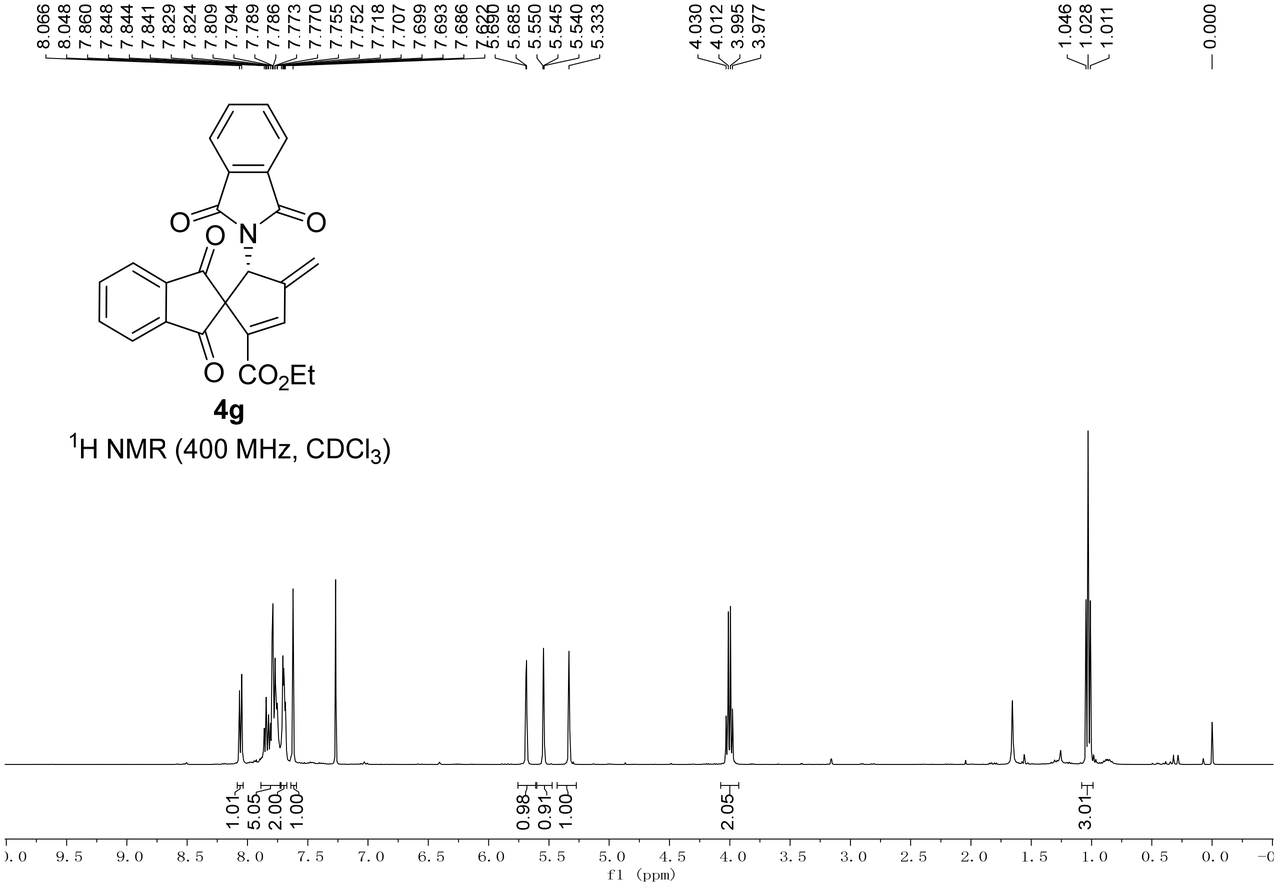
8.066
8.048
7.860
7.848
7.844
7.841
7.829
7.824
7.809
7.794
7.789
7.786
7.773
7.770
7.755
7.752
7.718
7.707
7.699
7.693
7.686
5.685
5.550
5.545
5.540
5.333
4.030
4.012
3.995
3.977
1.046
1.028
1.011
0.000
4g
^{1}H NMR (400 MHz, CDCl$_3$)
1.01
5.05
2.00
1.00
0.98
0.91
1.00
2.05
3.01
f1 (ppm)

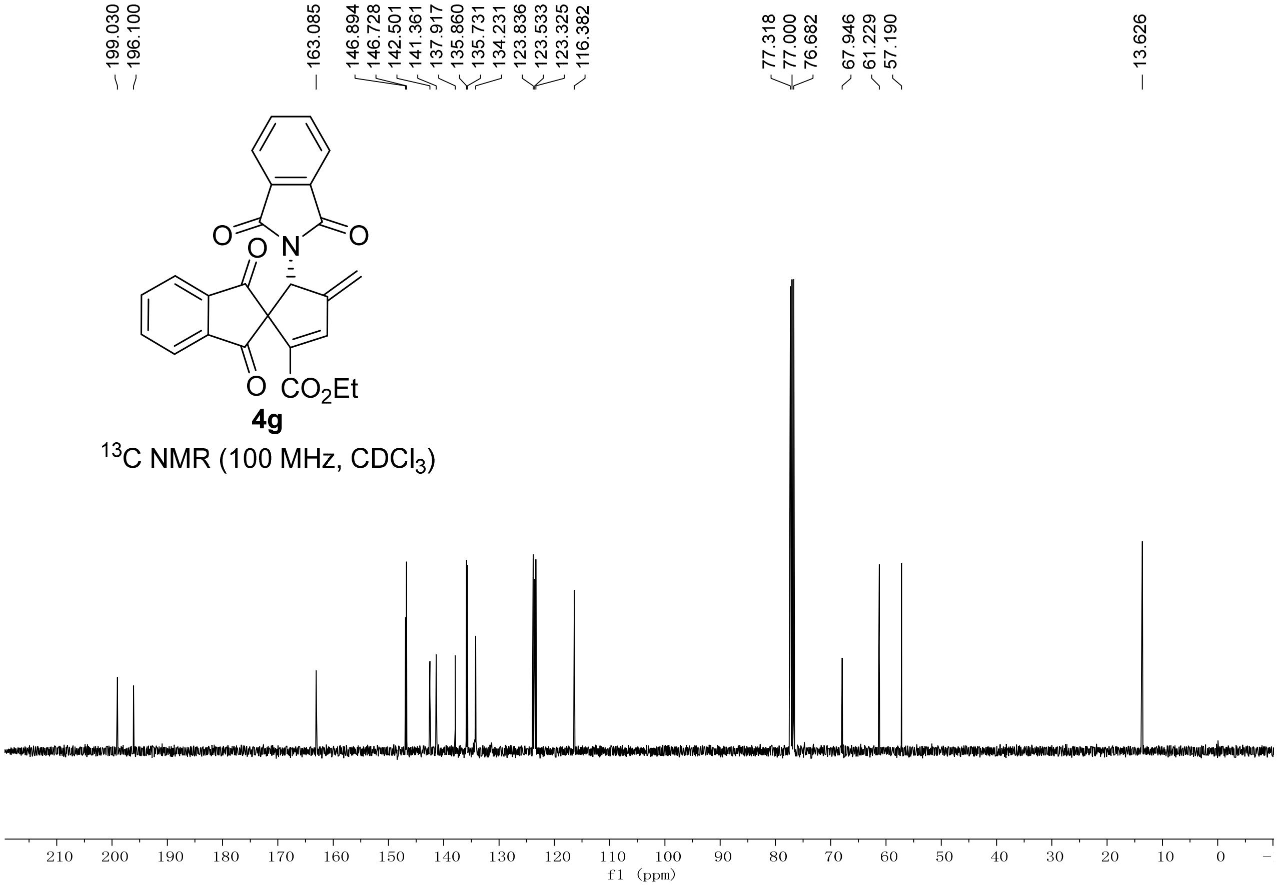
199.030
196.100
163.085
146.894
146.728
142.501
141.361
137.917
135.860
135.731
134.231
123.836
123.533
123.325
116.382
77.318
77.000
76.682
67.946
61.229
57.190
13.626
4g
^{13}C NMR (100 MHz, CDCl$_3$)
f1 (ppm)

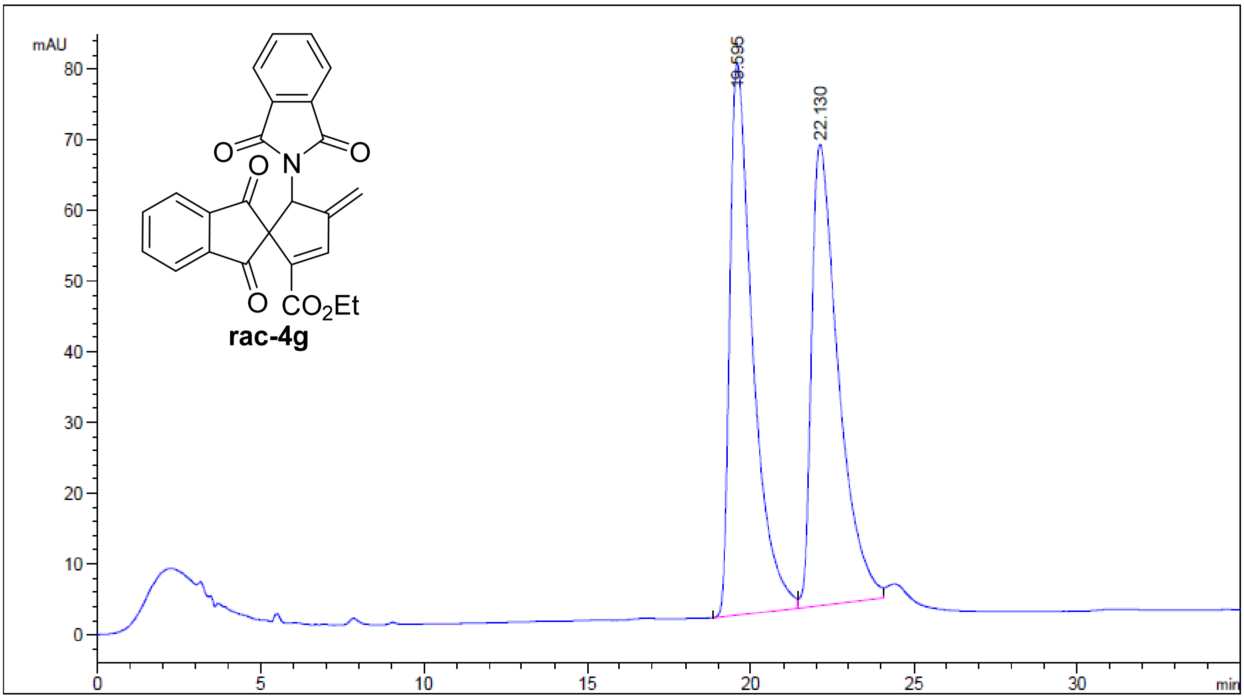

Peak #	RetTime [min]	Type	Width [min]	Area mAU *s	Height [mAU]	Area %
1	19.595	BV	0.7279	3810.81445	77.90732	50.5917
2	22.130	VV	0.8473	3721.68213	65.12380	49.4083

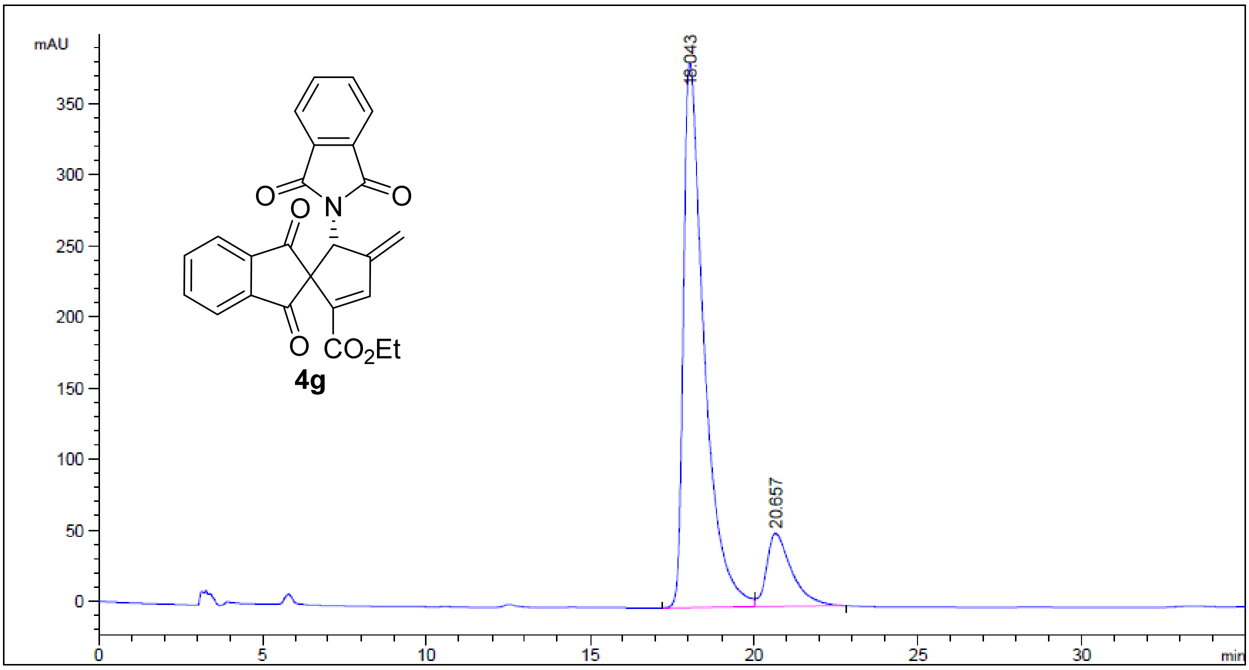

Peak #	RetTime [min]	Type	Width [min]	Area mAU *s	Height [mAU]	Area %
1	18.043	BV	0.6464	1.68587e4	383.83035	85.9881
2	20.657	VBA	0.7845	2747.15991	51.57176	14.0119

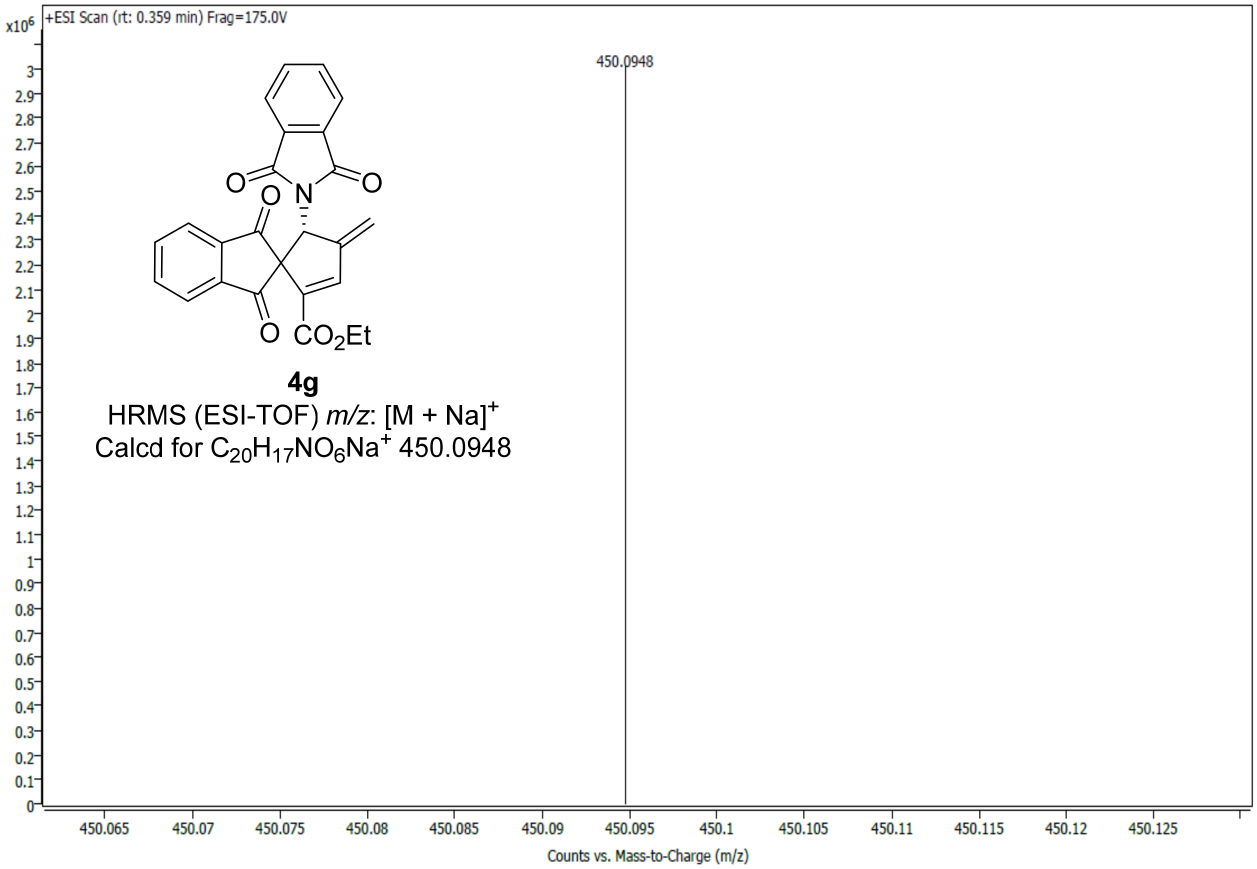
+ESI Scan (rt: 0.359 min) Frag=175.0V
x10^6
450.0948
4g
HRMS (ESI-TOF) m/z: [M + Na]+
Calcd for C20H17NO6Na+ 450.0948
Counts vs. Mass-to-Charge (m/z)

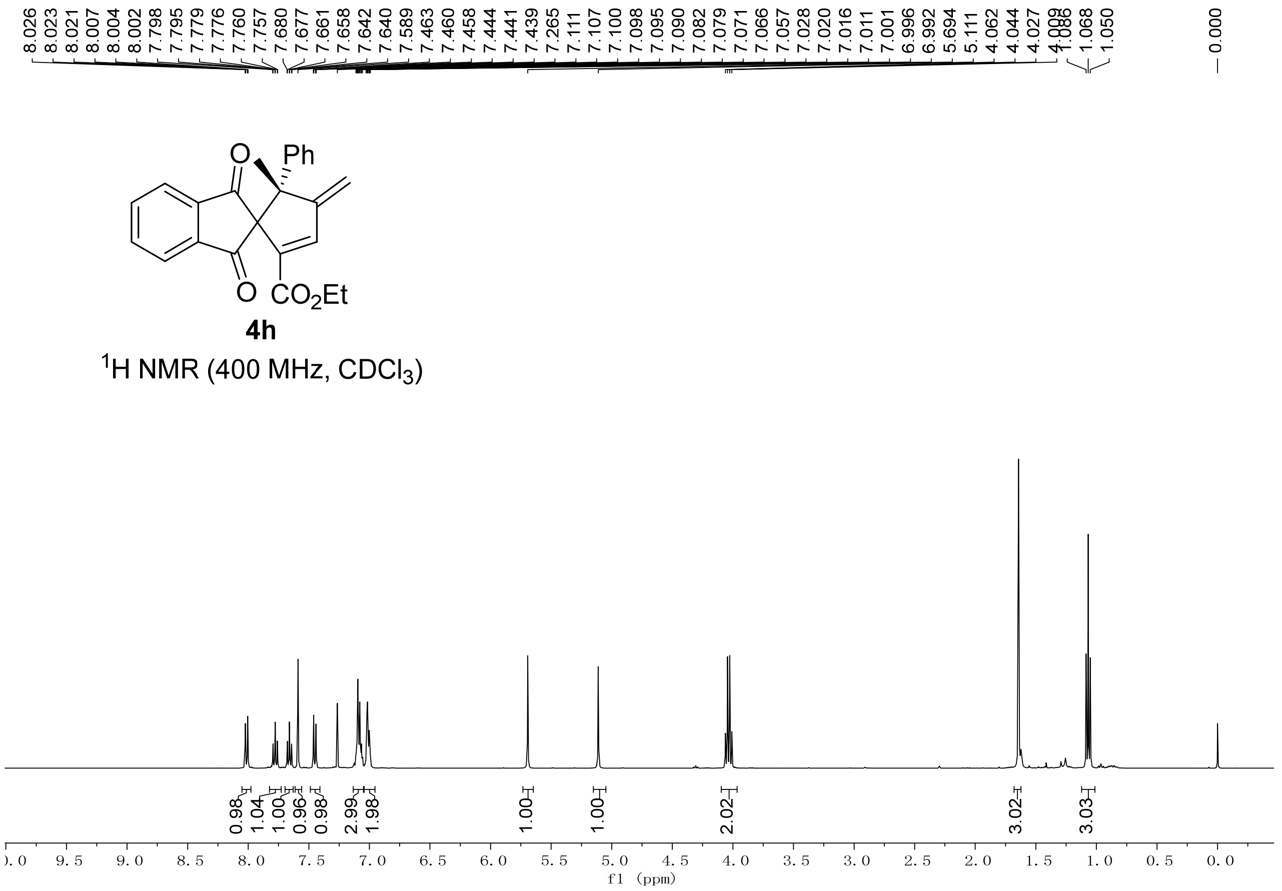
8.026 8.023 8.021 8.007 8.004 8.002 7.798 7.795 7.779 7.776 7.760 7.757 7.680 7.677 7.661 7.658 7.642 7.640 7.589 7.463 7.460 7.458 7.444 7.441 7.439 7.265 7.111 7.107 7.100 7.098 7.095 7.090 7.082 7.079 7.071 7.066 7.057 7.028 7.020 7.016 7.011 7.001 6.996 6.992 5.694 5.111 4.062 4.044 4.027 4.009 1.086 1.068 1.050 0.000
O
Ph
O
CO_2Et
4h
^1H NMR (400 MHz, $CDCl_3$)
0.98 1.04 1.00 0.96 0.98 2.99 1.98 1.00 1.00 2.02 3.02 3.03
f1 (ppm)

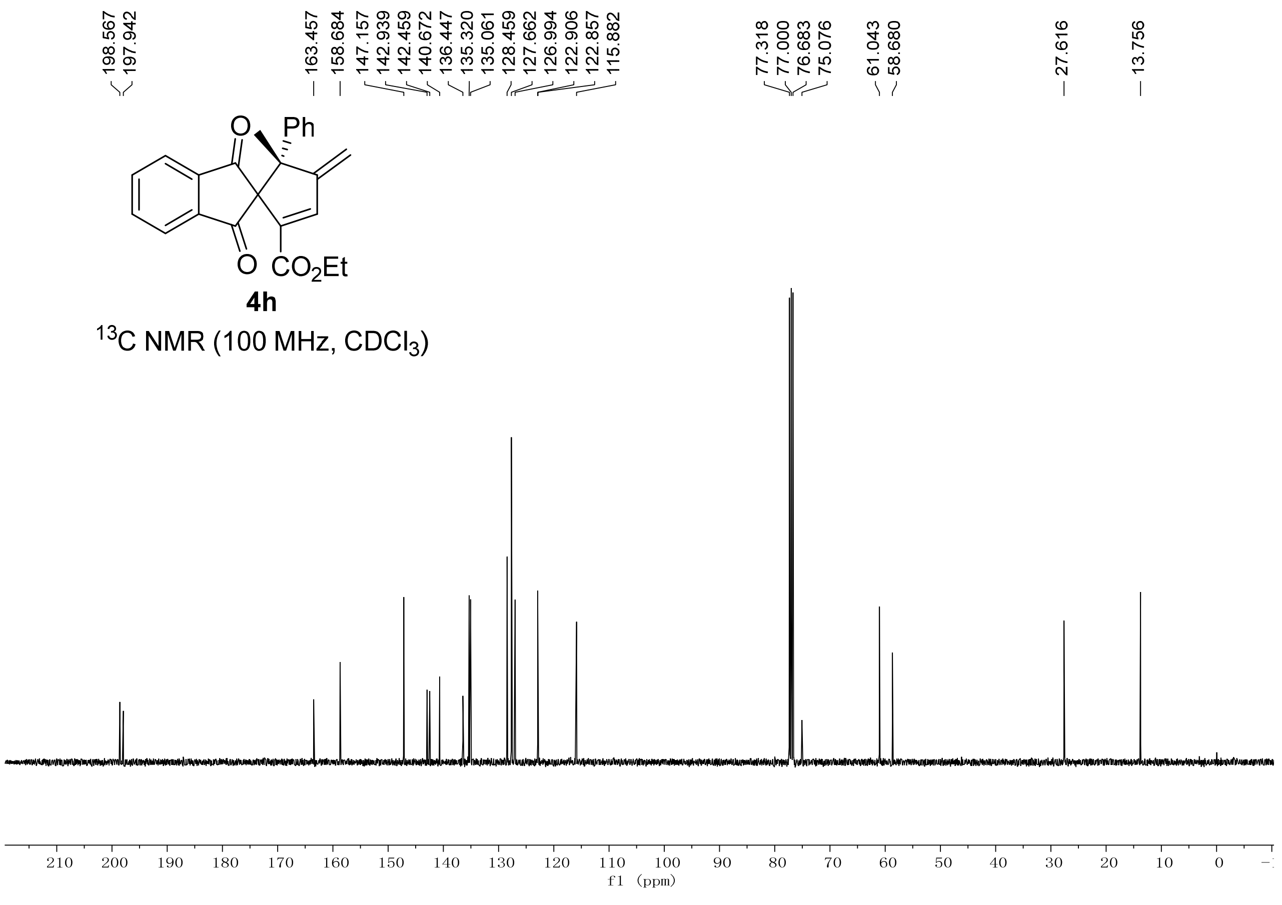
198.567 197.942 163.457 158.684 147.157 142.939 142.459 140.672 136.447 135.320 135.061 128.459 127.662 126.994 122.906 122.857 115.882 77.318 77.000 76.683 75.076 61.043 58.680 27.616 13.756
O
Ph
O
CO_2Et
4h
^{13}C NMR (100 MHz, $CDCl_3$)
f1 (ppm)

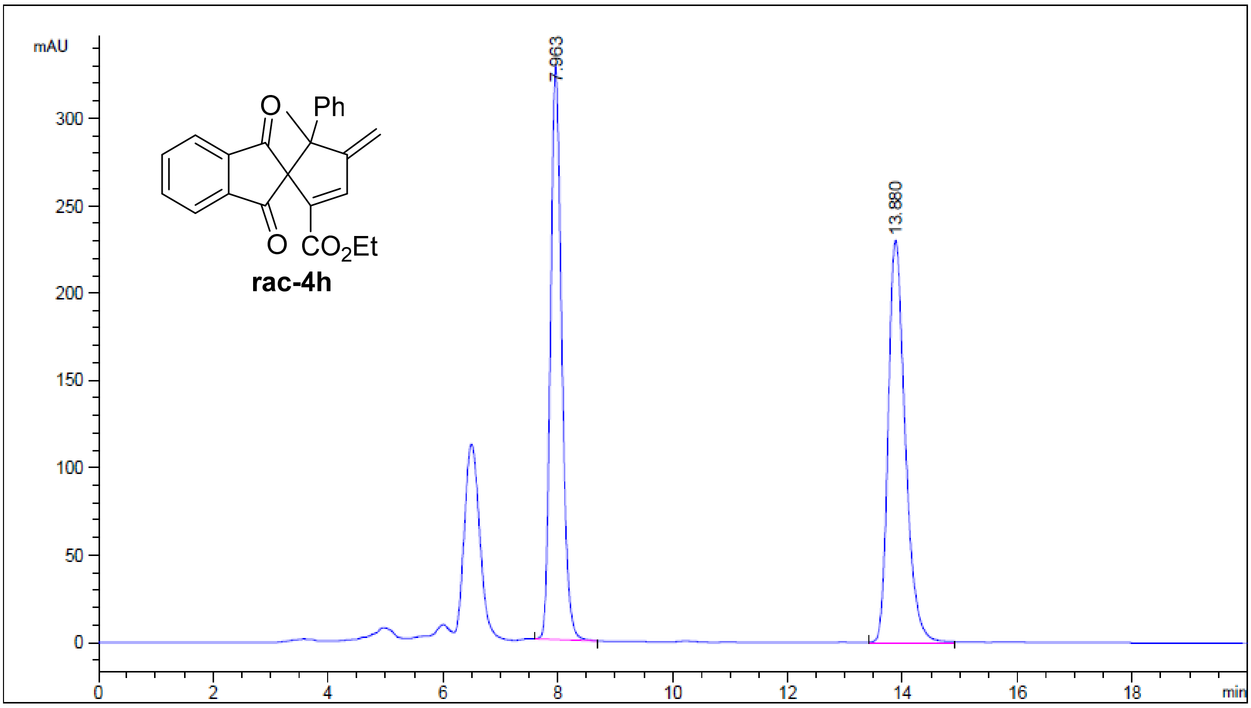

Peak #	RetTime [min]	Type	Width [min]	Area mAU *s	Height [mAU]	Area %
1	7.963	BBA	0.2086	4440.48779	328.65985	48.3884
2	13.880	BBA	0.3127	4736.26807	230.35765	51.6116

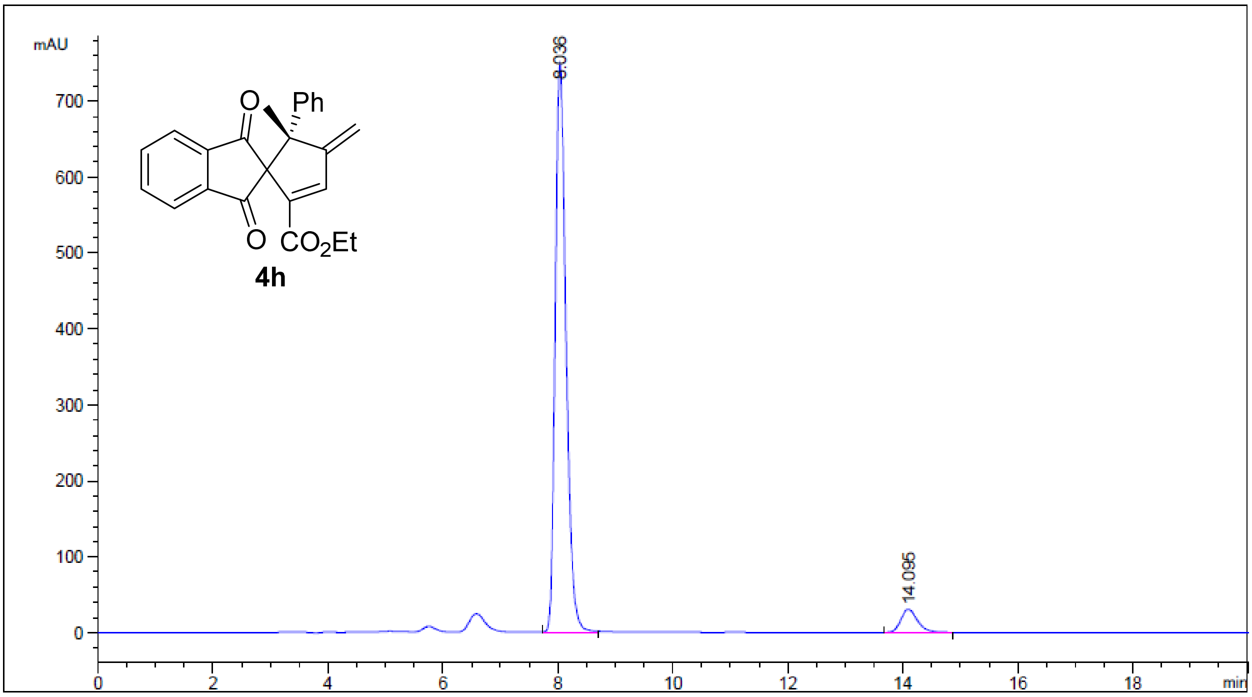

Peak #	RetTime [min]	Type	Width [min]	Area mAU *s	Height [mAU]	Area %
1	8.036	BBA	0.2002	9765.50586	748.78931	93.9668
2	14.095	BB	0.3139	626.99811	30.52639	6.0332

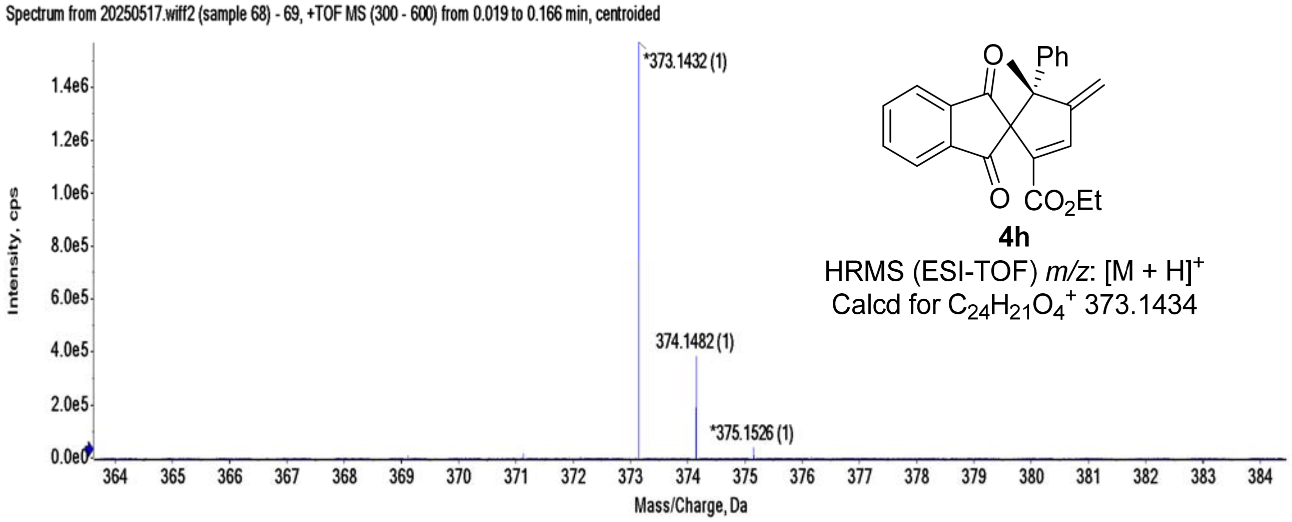

4h

HRMS (ESI-TOF) *m/z*: $[M + H]^+$

Calcd for $C_{24}H_{21}O_4^+$ 373.1434

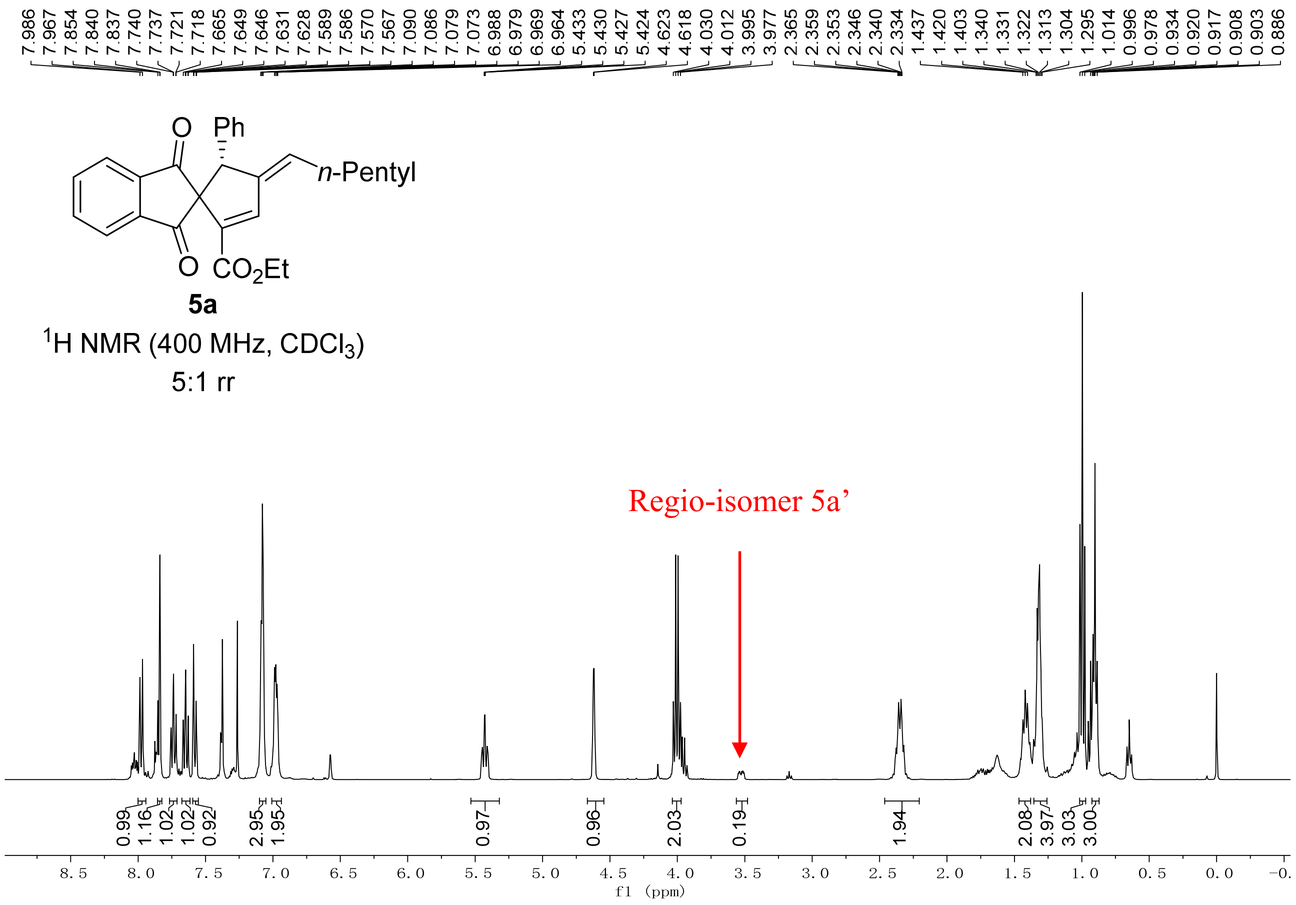

Ph
n-Pentyl
O
O
CO2Et
5a
1H NMR (400 MHz, CDCl3)
5:1 rr
Regio-isomer 5a'
f1 (ppm)

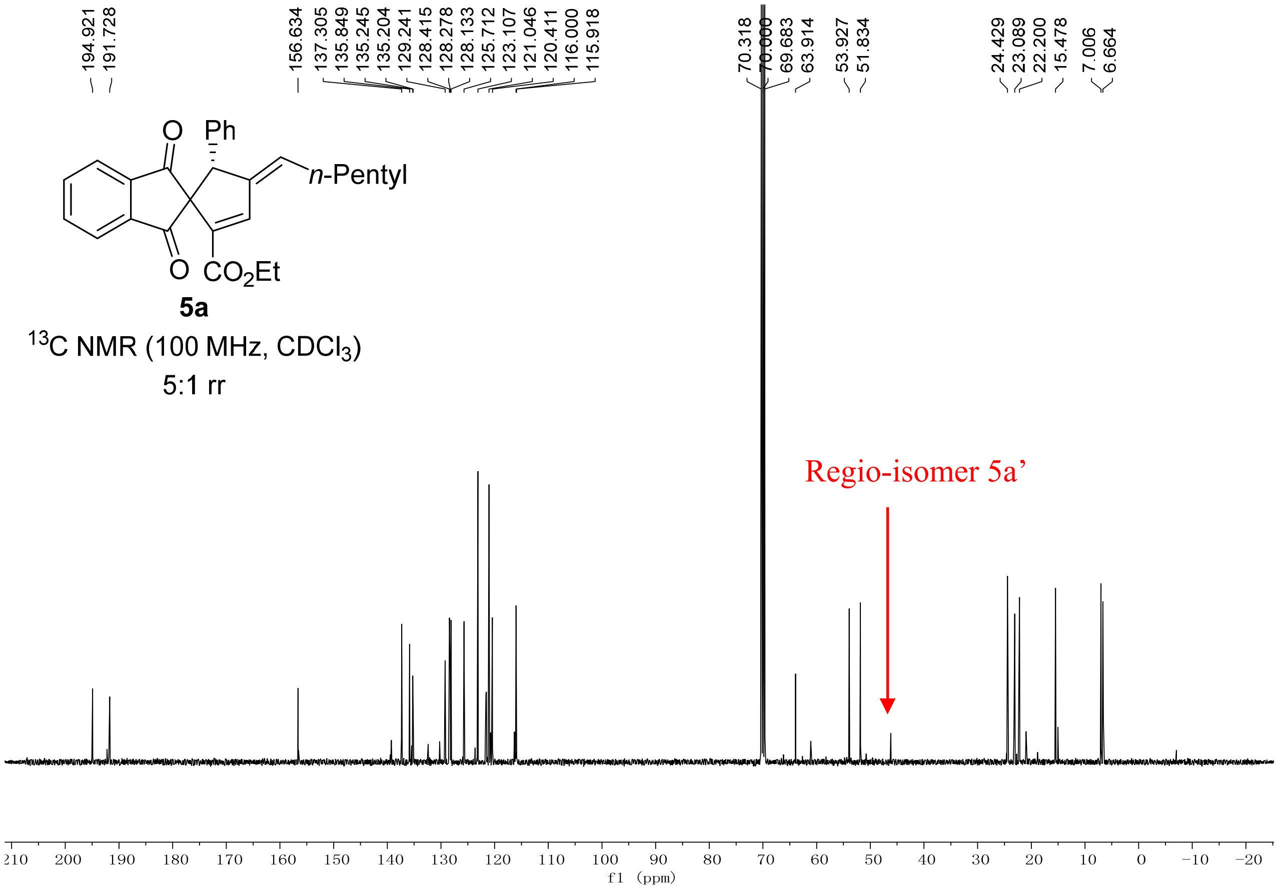

Ph
n-Pentyl
O
O
CO2Et
5a
13C NMR (100 MHz, CDCl3)
5:1 rr
Regio-isomer 5a'
f1 (ppm)

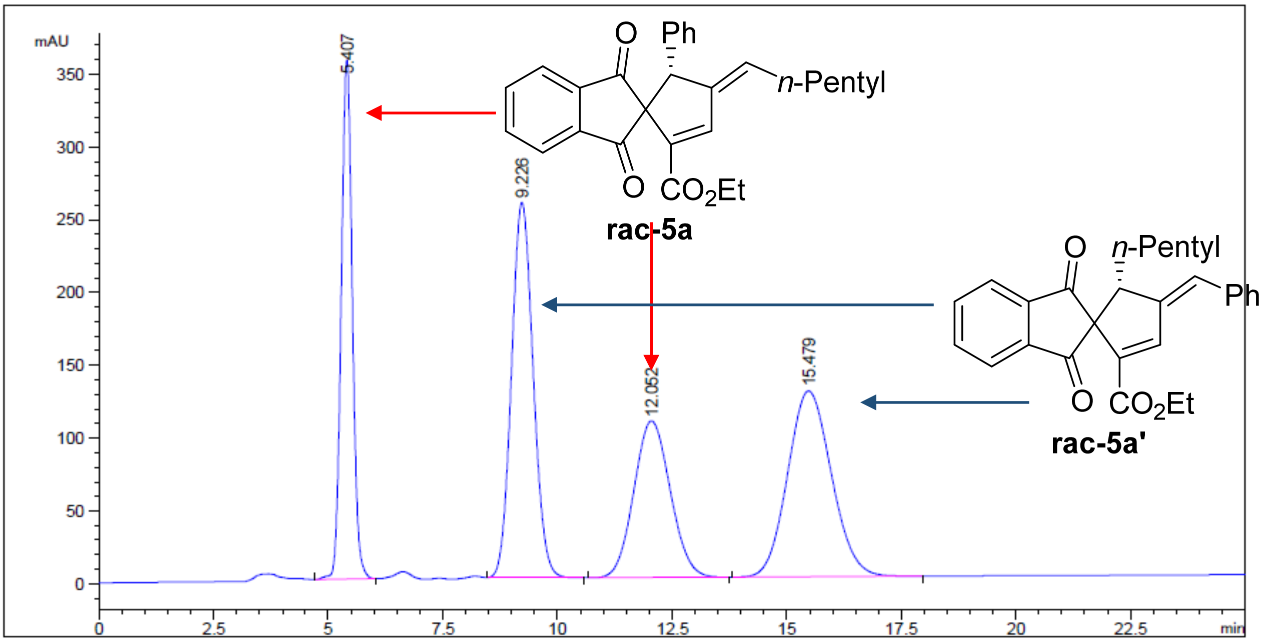

Peak #	RetTime [min]	Type	Width [min]	Area mAU *s	Height [mAU]	Area %
1	5.407	BB	0.2605	6014.07031	357.14621	20.8150
2	9.226	BB	0.5101	8468.12695	257.68747	29.3086
3	12.052	BB	0.8627	5939.95801	107.55418	20.5585
4	15.479	BB	1.0394	8470.80664	127.60880	29.3179

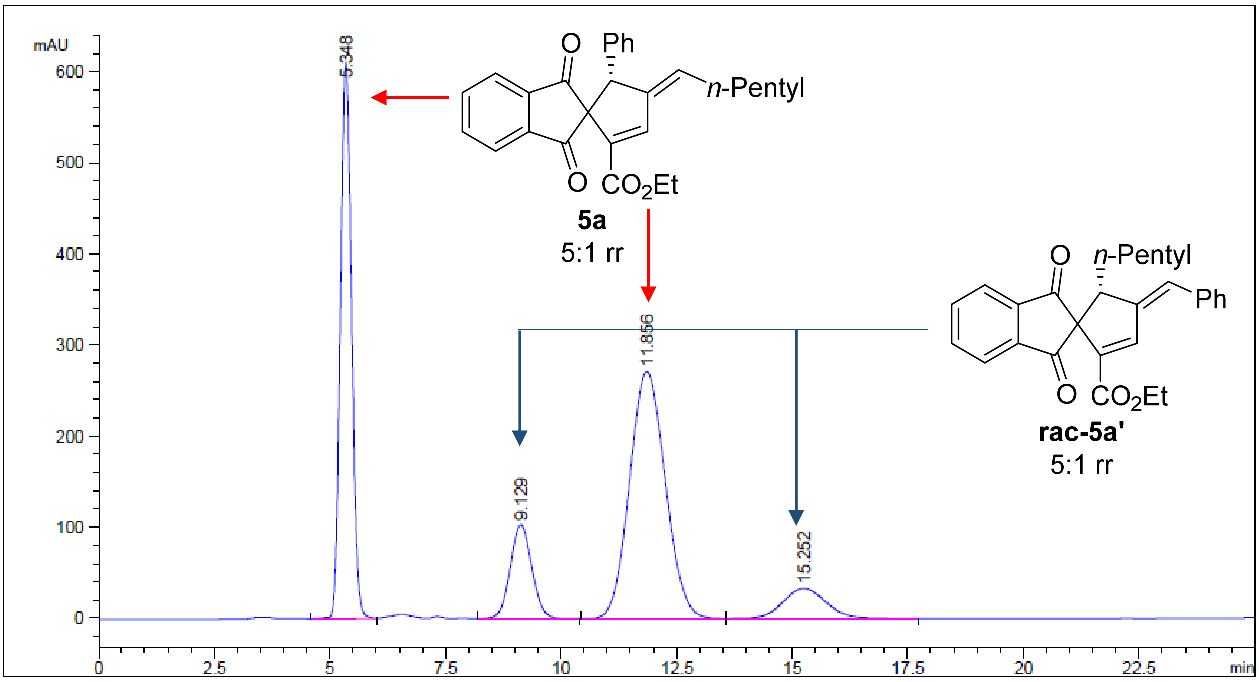

Peak #	RetTime [min]	Type	Width [min]	Area mAU *s	Height [mAU]	Area %
1	5.348	BV	0.2667	1.02985e4	610.35651	33.9332
2	9.129	BB	0.5035	3340.07788	103.00732	11.0055
3	11.856	BB	0.8417	1.45692e4	271.43509	48.0052
4	15.252	BB	1.0007	2141.47095	33.17212	7.0561

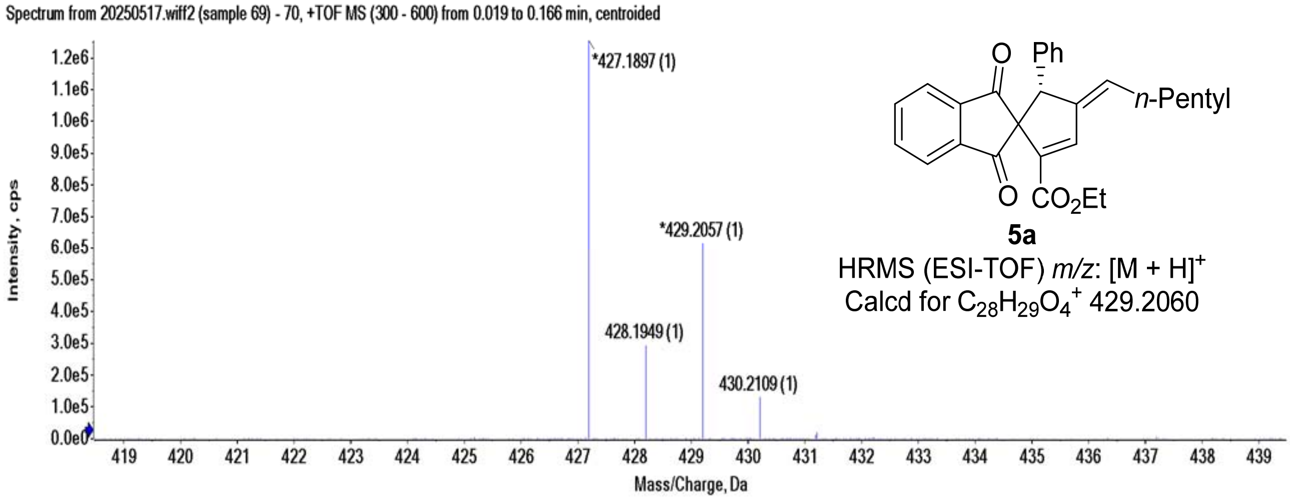

5a

HRMS (ESI-TOF) *m/z*: $[M + H]^+$

Calcd for $C_{28}H_{29}O_4^+$ 429.2060

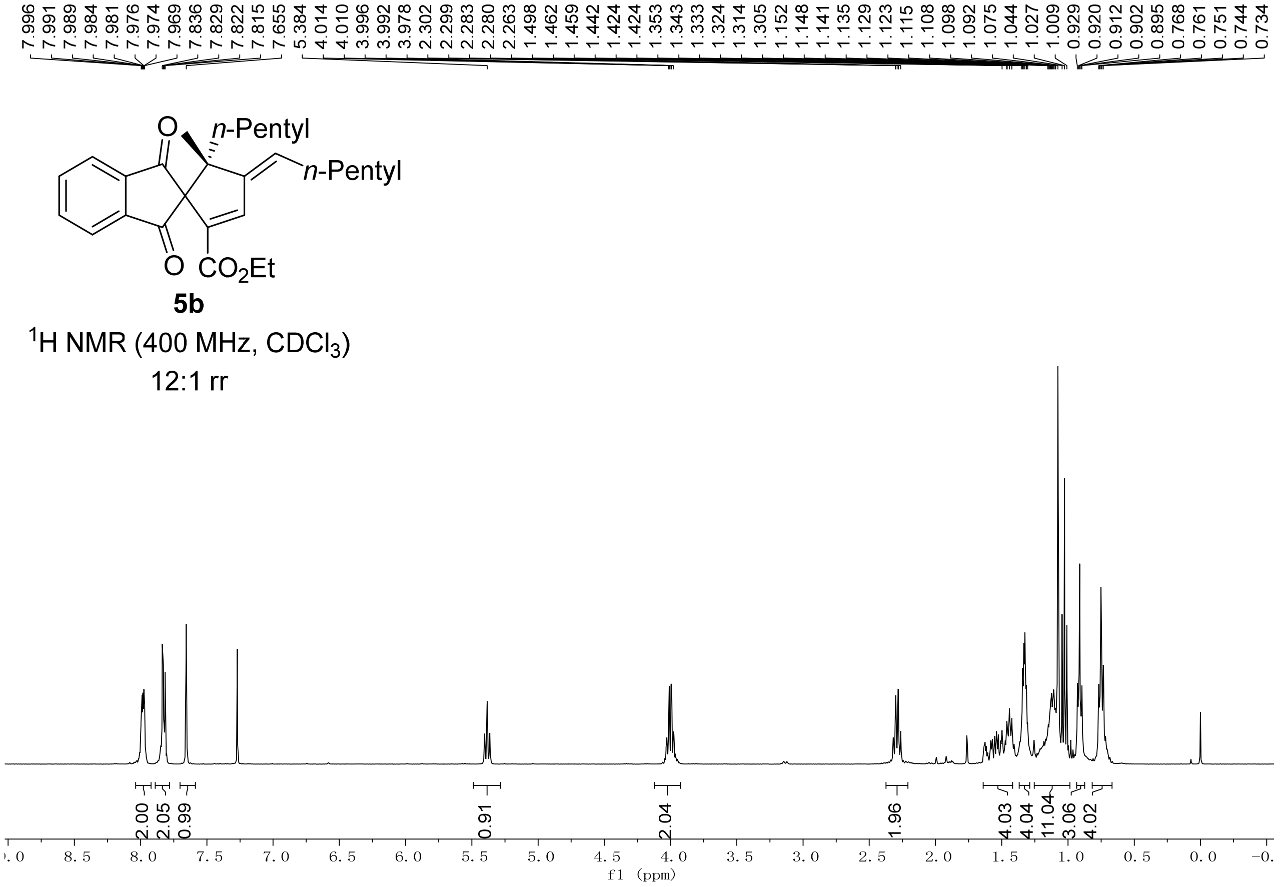
7.996 7.991 7.989 7.984 7.981 7.976 7.974 7.969 7.836 7.829 7.822 7.815 7.655 5.384 4.014 4.010 3.996 3.992 3.978 2.302 2.299 2.283 2.280 2.263 1.498 1.462 1.459 1.442 1.424 1.424 1.353 1.343 1.333 1.324 1.314 1.305 1.152 1.148 1.141 1.135 1.129 1.123 1.115 1.108 1.098 1.092 1.075 1.044 1.027 1.009 0.929 0.920 0.912 0.902 0.895 0.768 0.761 0.751 0.744 0.734
O
O
n-Pentyl
n-Pentyl
CO2Et
5b
1H NMR (400 MHz, CDCl3)
12:1 rr
2.00 2.05 0.99 0.91 2.04 1.96 4.03 4.04 11.04 3.06 4.02
f1 (ppm)

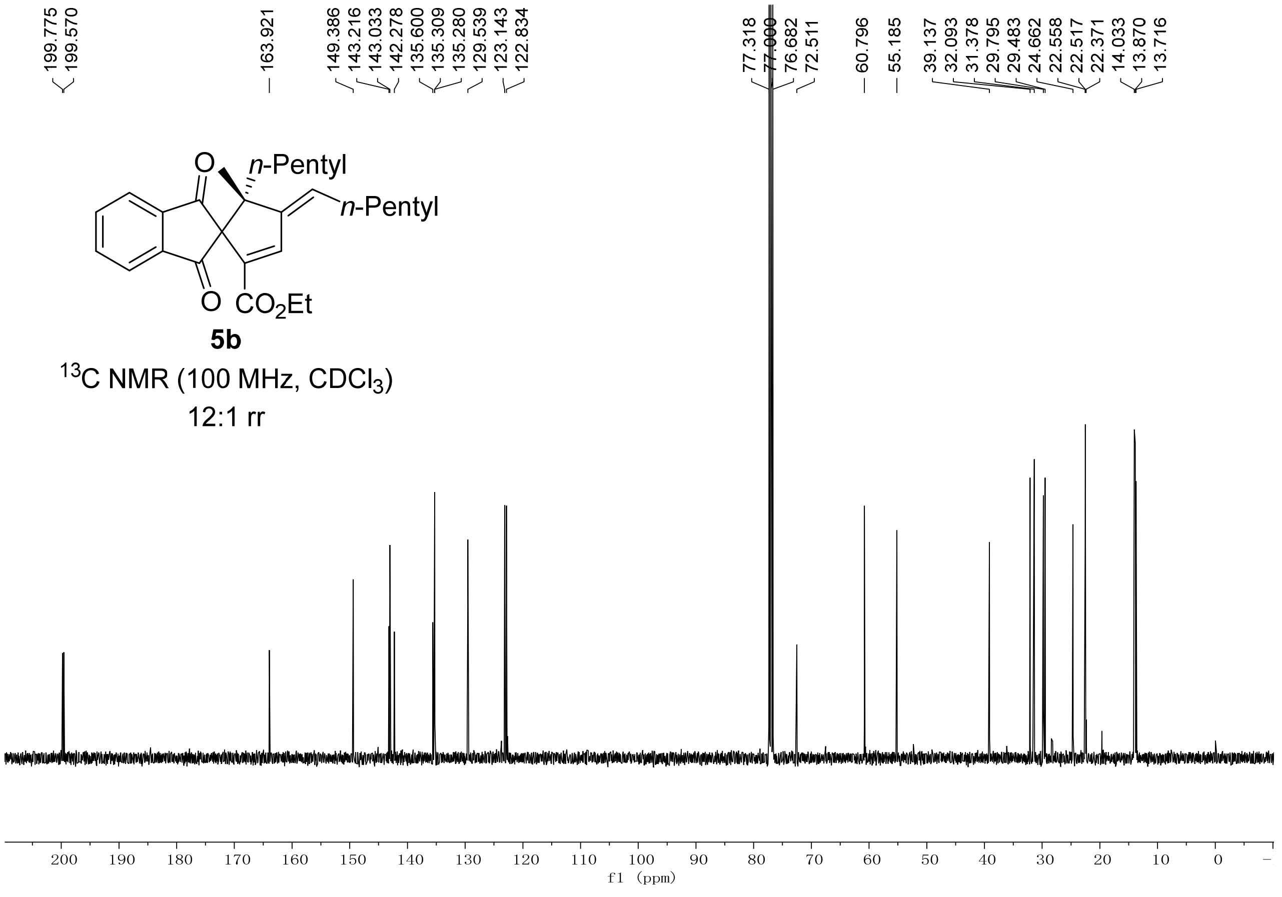
199.775 199.570 163.921 149.386 143.216 143.033 142.278 135.600 135.309 135.280 129.539 123.143 122.834 77.318 77.000 76.682 72.511 60.796 55.185 39.137 32.093 31.378 29.795 29.483 24.662 22.558 22.517 22.371 14.033 13.870 13.716
O
O
n-Pentyl
n-Pentyl
CO2Et
5b
13C NMR (100 MHz, CDCl3)
12:1 rr
f1 (ppm)

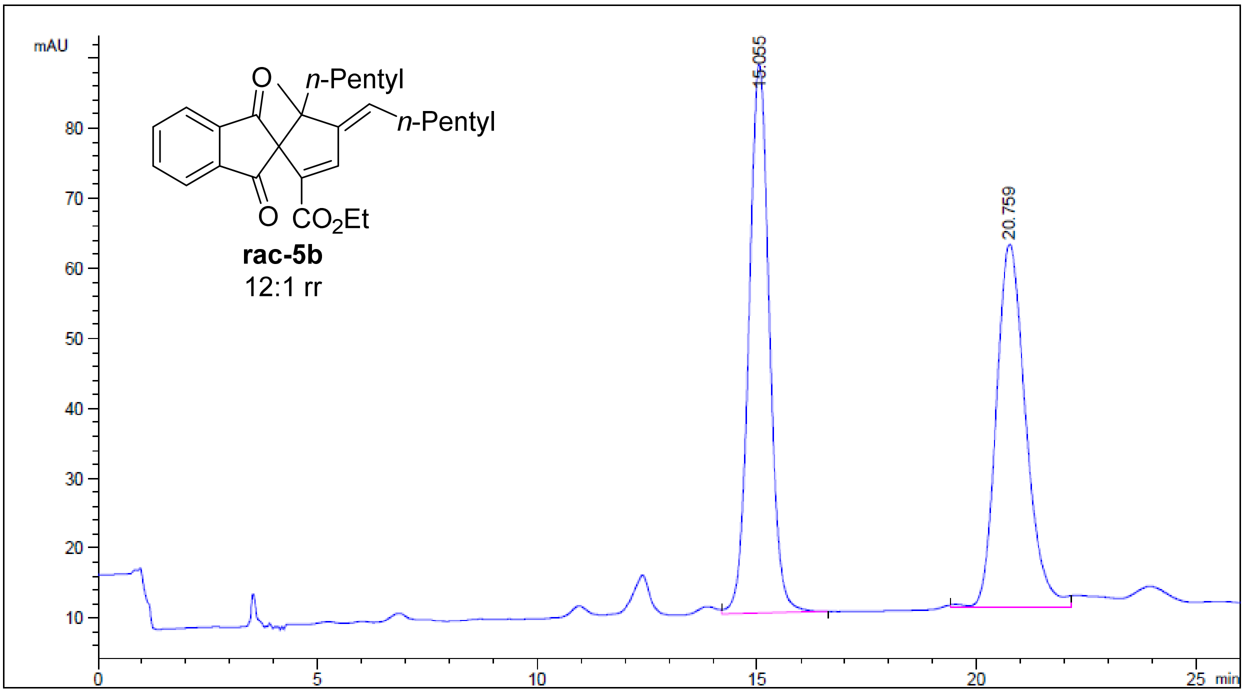

Peak #	RetTime [min]	Type	Width [min]	Area mAU *s	Height [mAU]	Area %
1	15.055	VB	0.4998	2539.45508	78.47913	50.9908
2	20.759	MM	0.7829	2440.77173	51.96175	49.0092

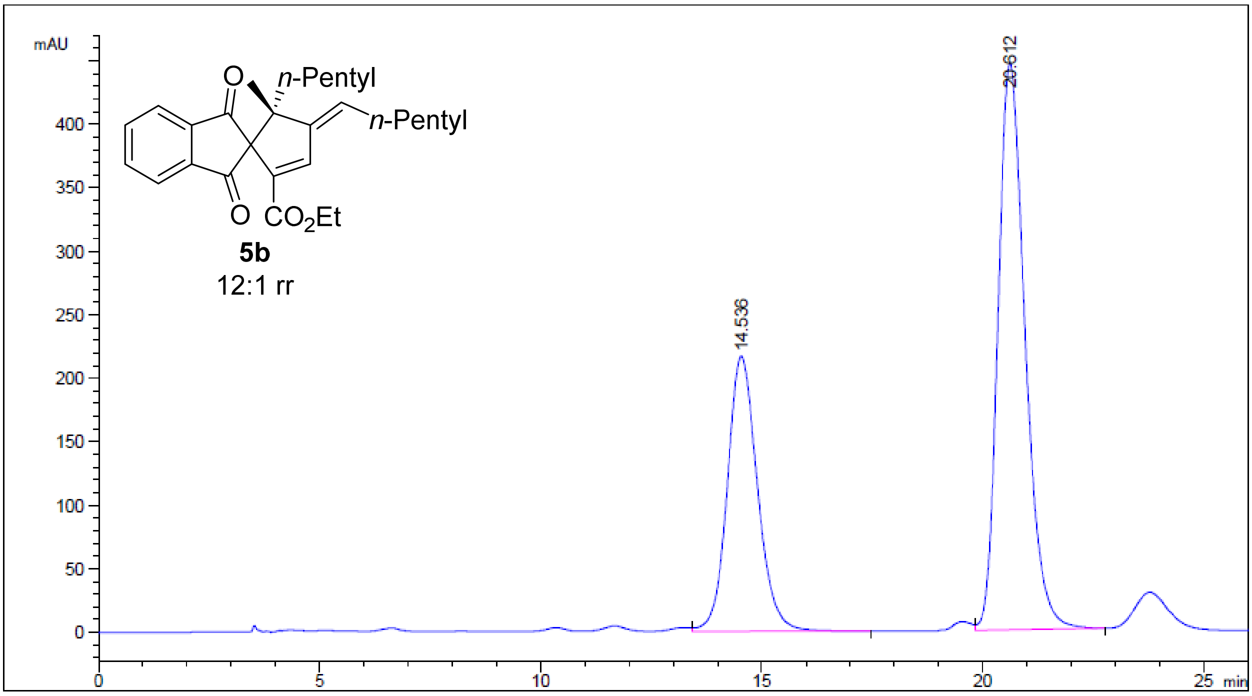

Peak #	RetTime [min]	Type	Width [min]	Area mAU *s	Height [mAU]	Area %
1	14.536	VB	0.7258	1.02487e4	216.39742	35.0718
2	20.612	VB	0.6567	1.89733e4	445.79547	64.9282

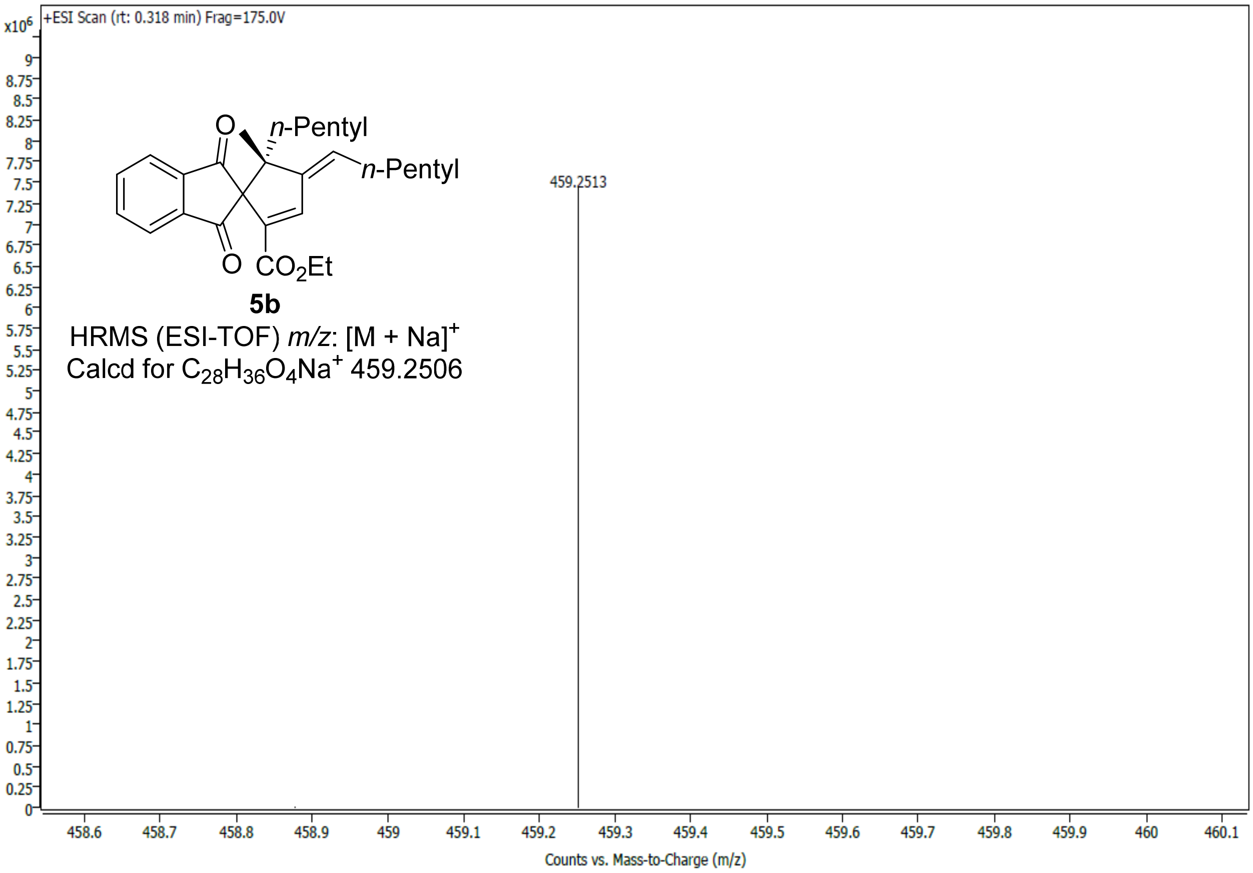

x10^6
+ESI Scan (rt: 0.318 min) Frag=175.0V
n-Pentyl
n-Pentyl
O
O
CO2Et
5b
HRMS (ESI-TOF) m/z: [M + Na]+
Calcd for C28H36O4Na+ 459.2506
459.2513
Counts vs. Mass-to-Charge (m/z)

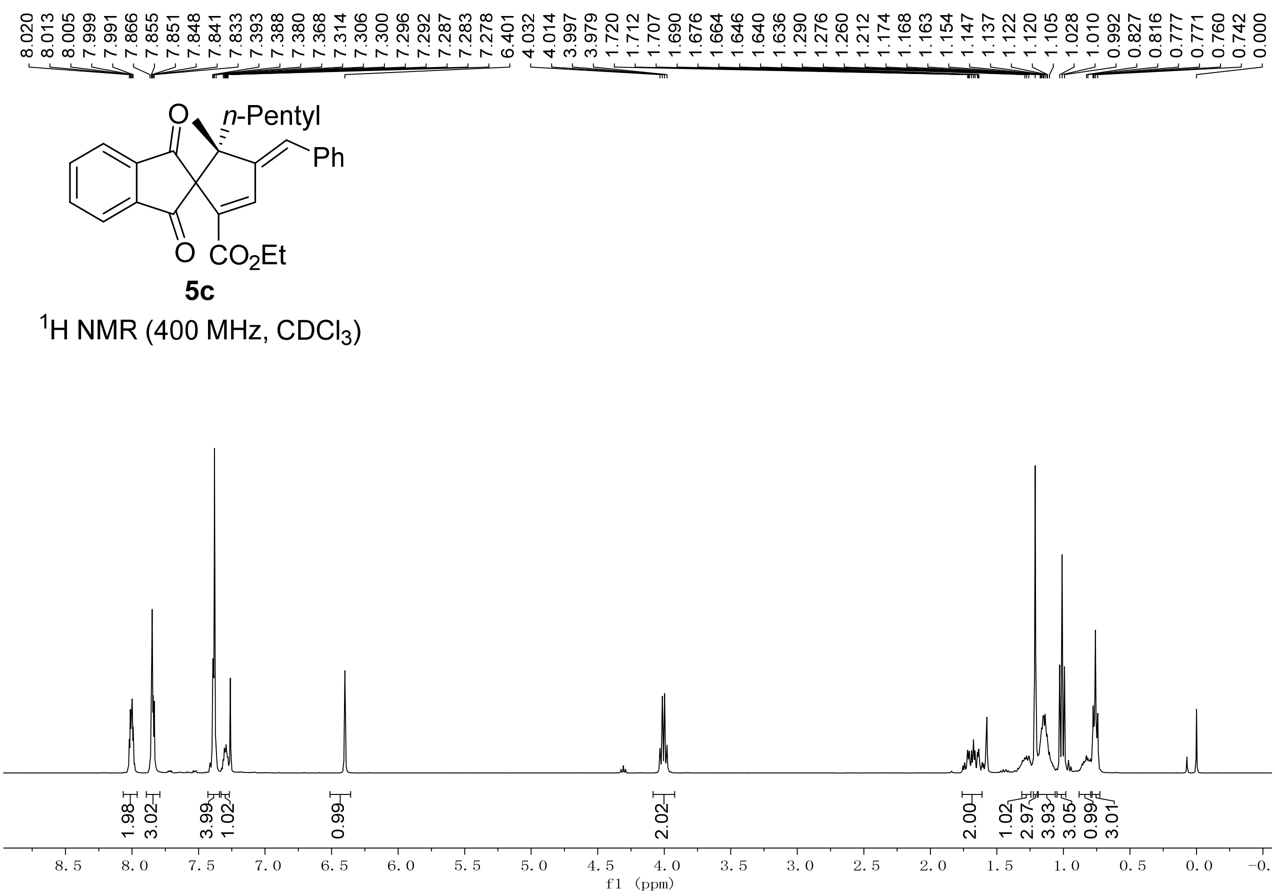

5c

^{1}H NMR (400 MHz, $CDCl_3$)

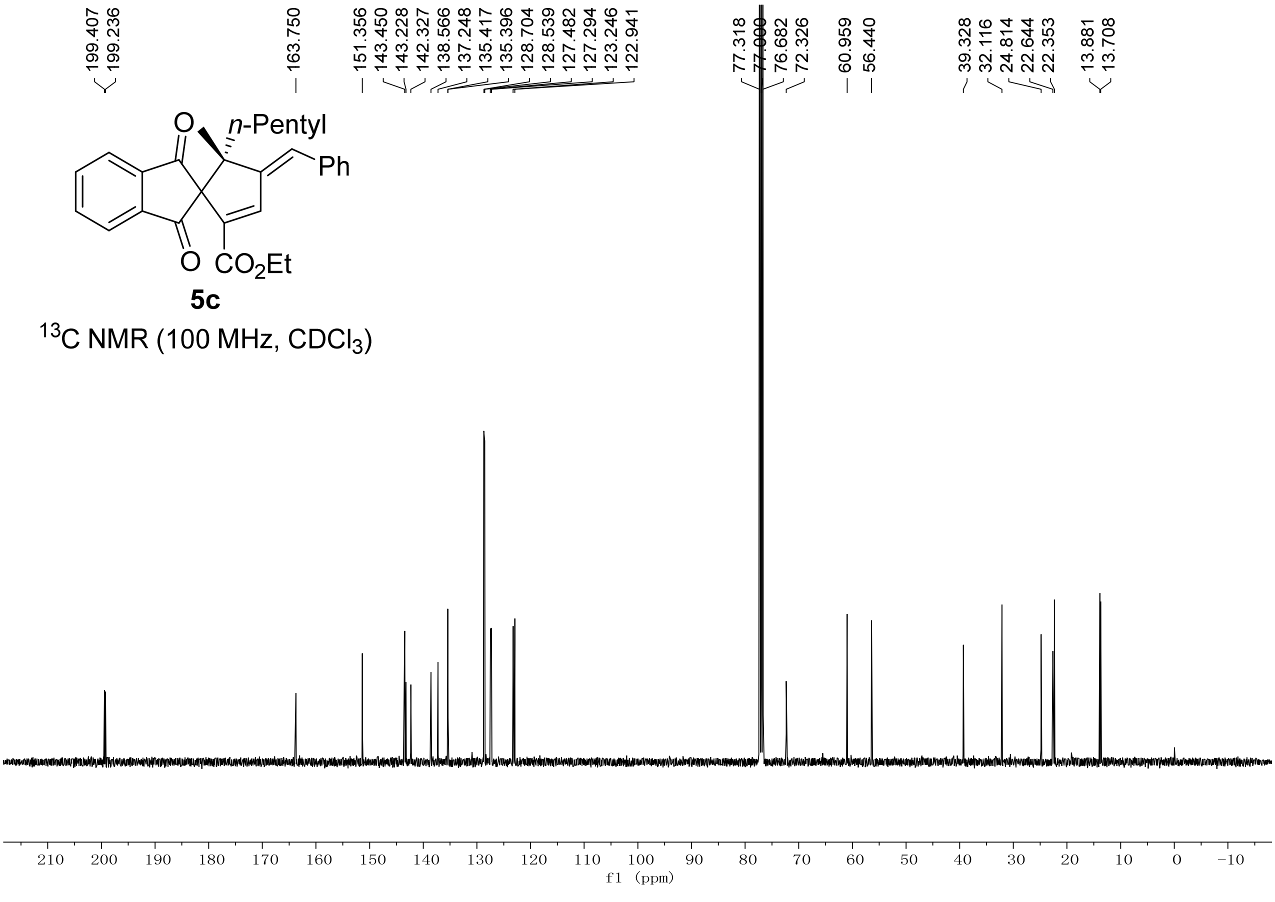

5c

^{13}C NMR (100 MHz, $CDCl_3$)

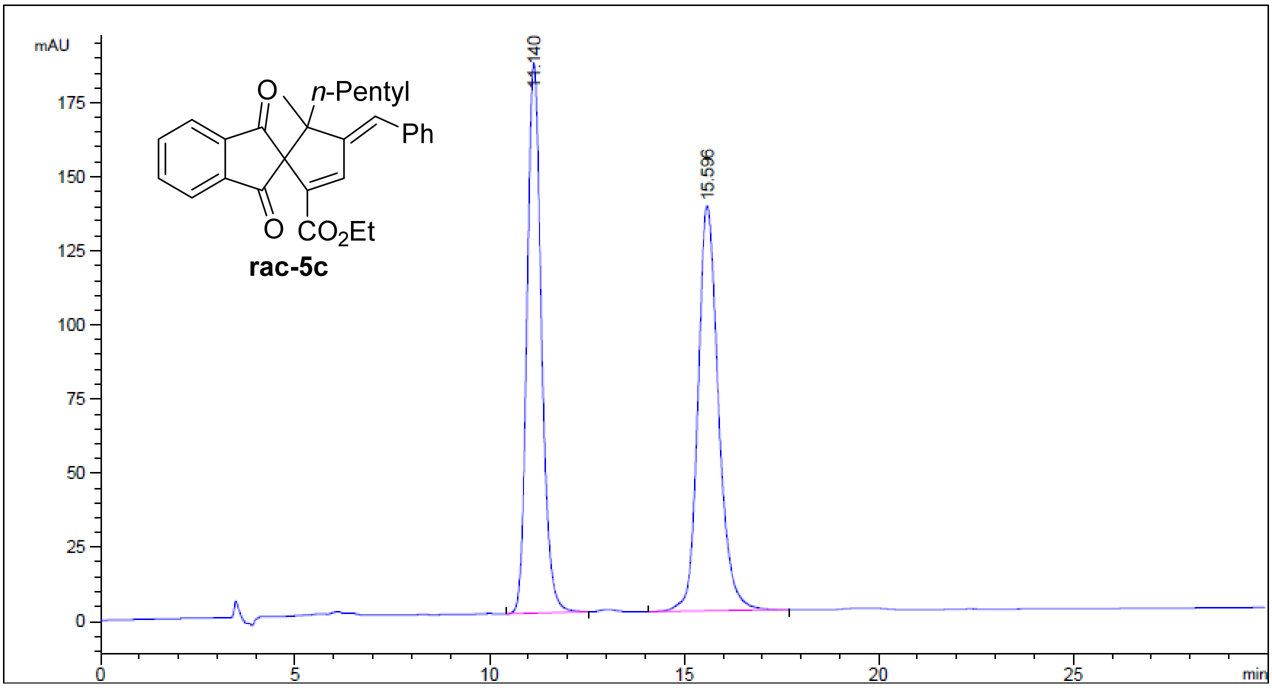

Peak #	RetTime [min]	Type	Width [min]	Area mAU *s	Height [mAU]	Area %
1	11.140	BB	0.3982	4803.31104	185.46581	48.8396
2	15.596	BB	0.5603	5031.55127	136.73647	51.1604

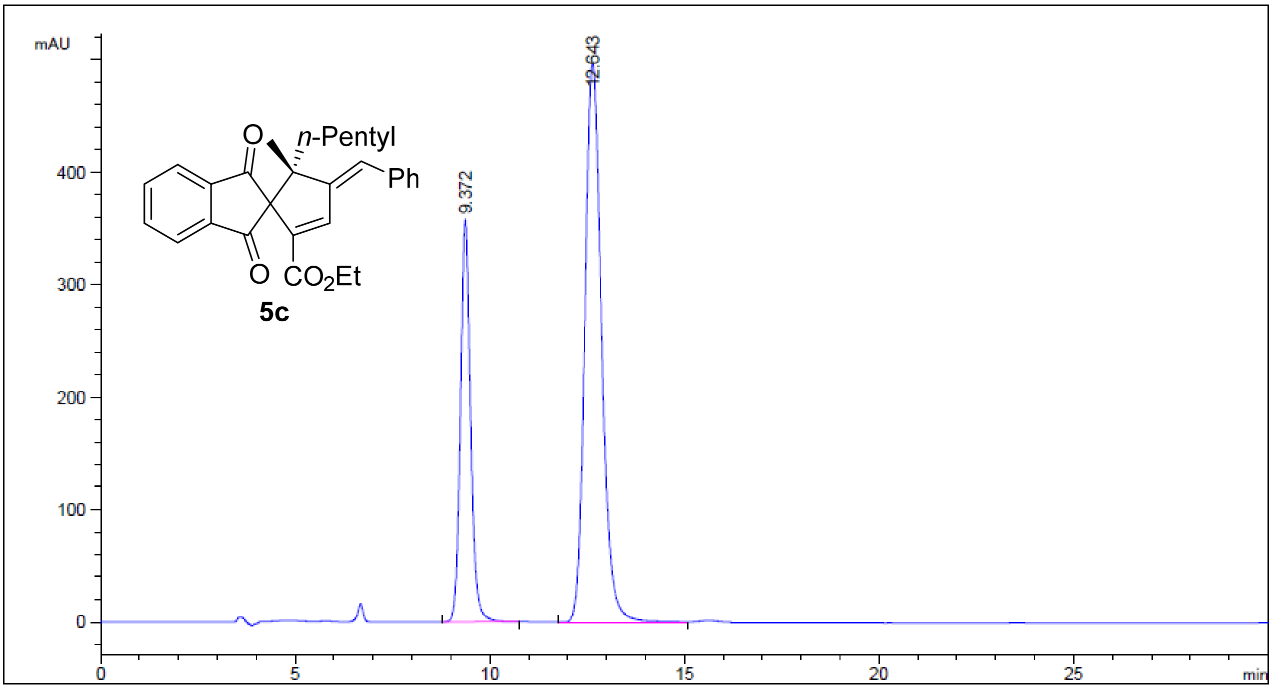

Peak #	RetTime [min]	Type	Width [min]	Area mAU *s	Height [mAU]	Area %
1	9.372	BB	0.2784	6539.89160	358.37848	30.6346
2	12.643	BB	0.4582	1.48082e4	497.11041	69.3654

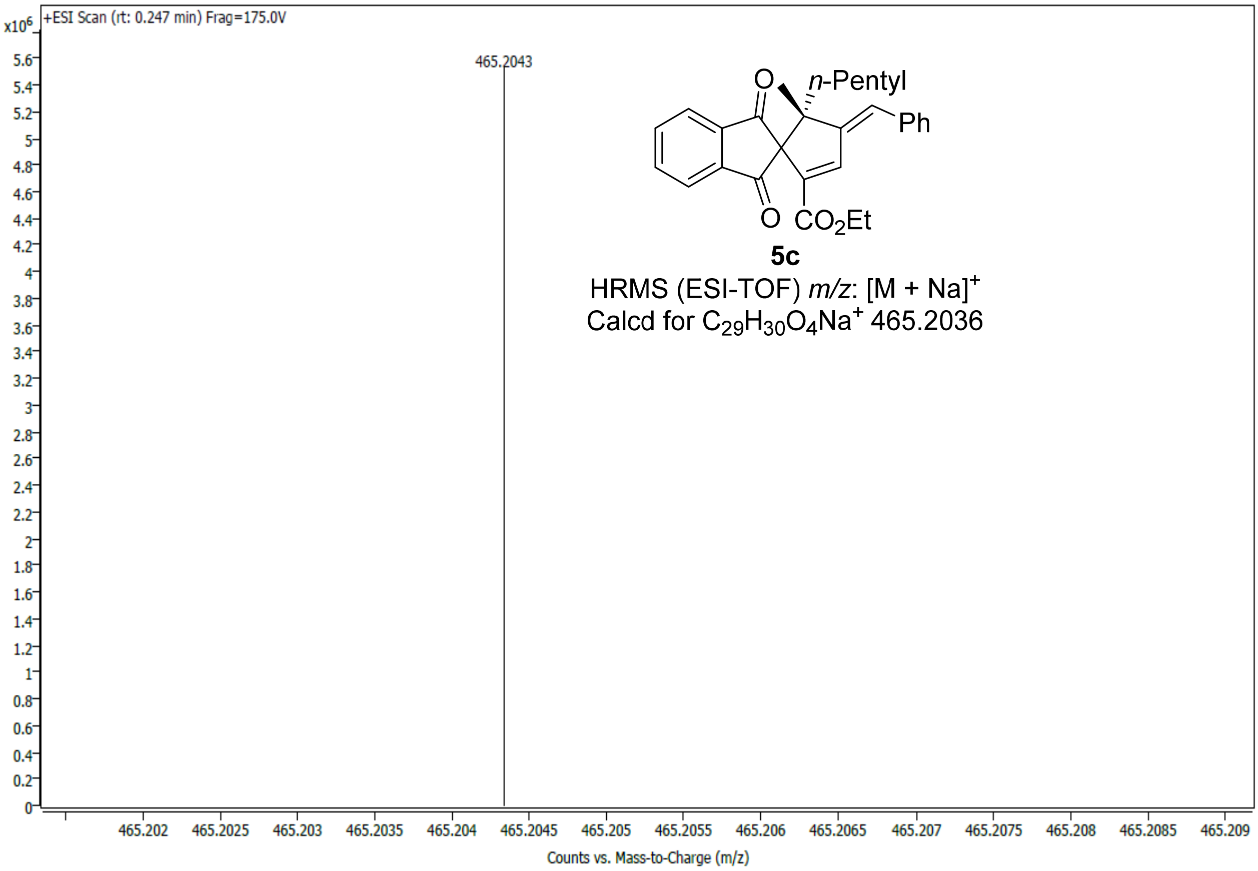
+ESI Scan (rt: 0.247 min) Frag=175.0V
x10^6
465.2043
n-Pentyl
Ph
O
O
CO2Et
5c
HRMS (ESI-TOF) m/z: [M + Na]+
Calcd for C29H30O4Na+ 465.2036
Counts vs. Mass-to-Charge (m/z)

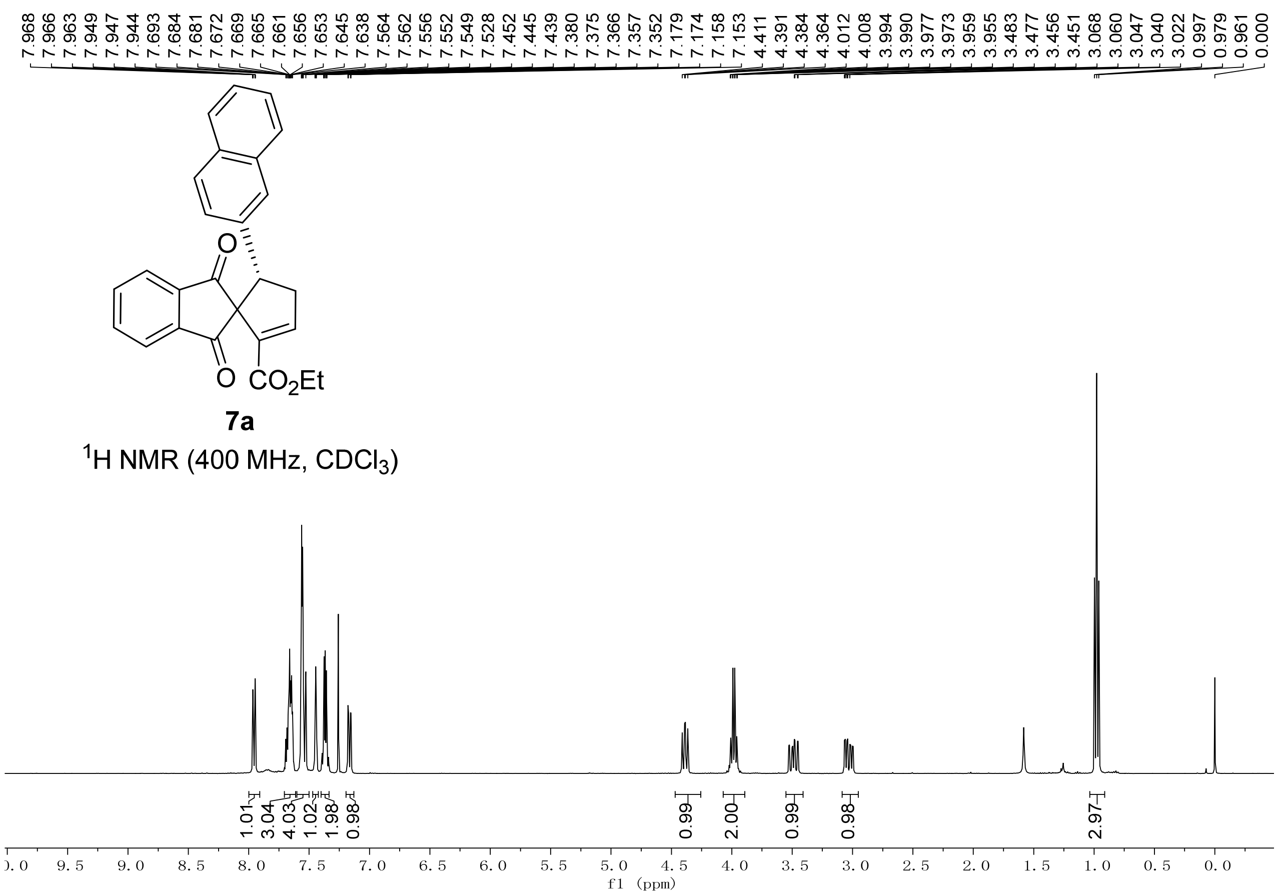
7.968
7.966
7.963
7.949
7.947
7.944
7.693
7.684
7.681
7.672
7.669
7.665
7.661
7.656
7.653
7.645
7.638
7.564
7.562
7.556
7.552
7.549
7.528
7.452
7.445
7.439
7.380
7.375
7.366
7.357
7.352
7.179
7.174
7.158
7.153
4.411
4.391
4.384
4.364
4.012
4.008
3.994
3.990
3.977
3.973
3.959
3.955
3.483
3.477
3.456
3.451
3.068
3.060
3.047
3.040
3.022
0.997
0.979
0.961
0.000
O
O
CO2Et
7a
1H NMR (400 MHz, CDCl3)
1.01
3.04
4.03
1.02
1.98
0.98
0.99
2.00
0.99
0.98
2.97
f1 (ppm)

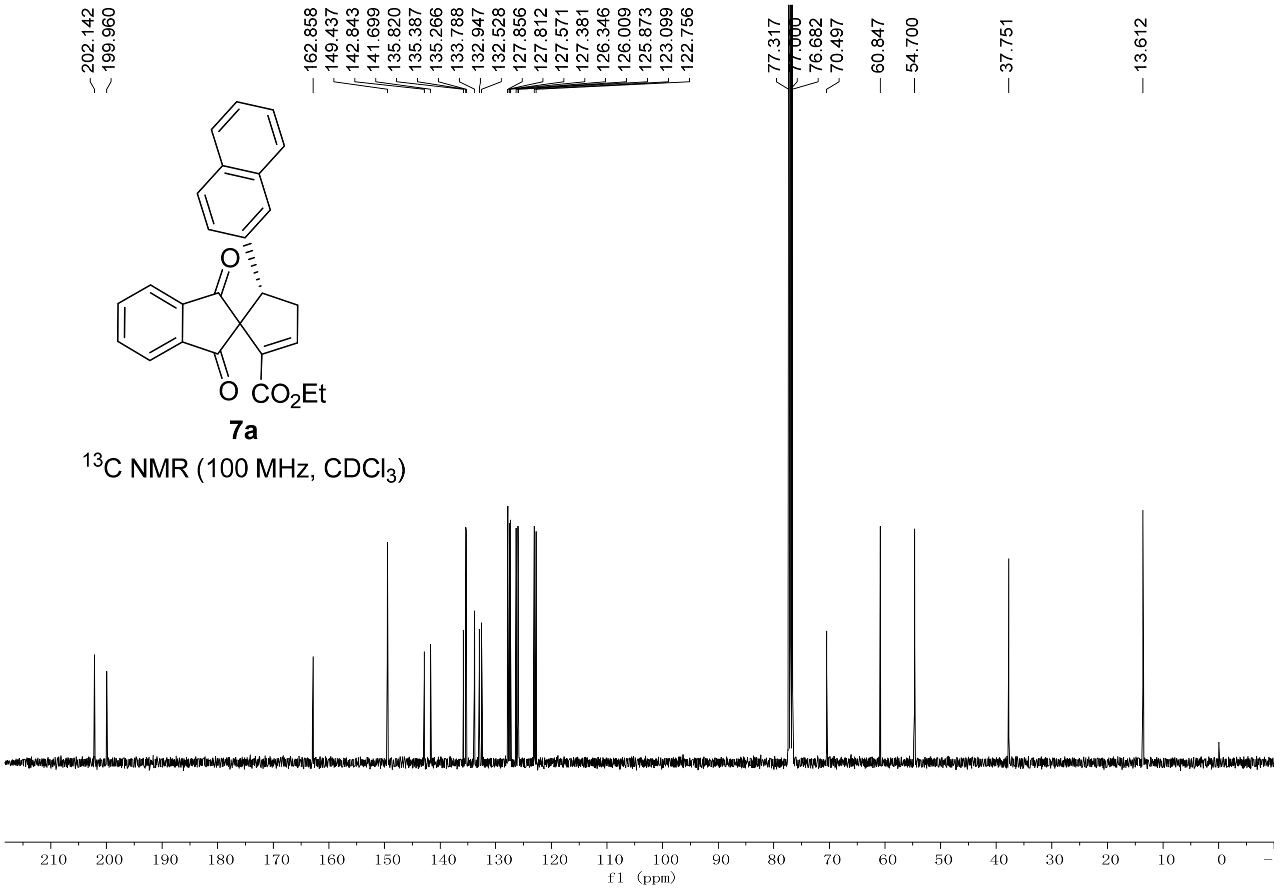
202.142
199.960
162.858
149.437
142.843
141.699
135.820
135.387
135.266
133.788
132.947
132.528
127.856
127.812
127.571
127.381
126.346
126.009
125.873
123.099
122.756
77.317
76.682
70.497
60.847
54.700
37.751
13.612
O
O
CO2Et
7a
13C NMR (100 MHz, CDCl3)
f1 (ppm)

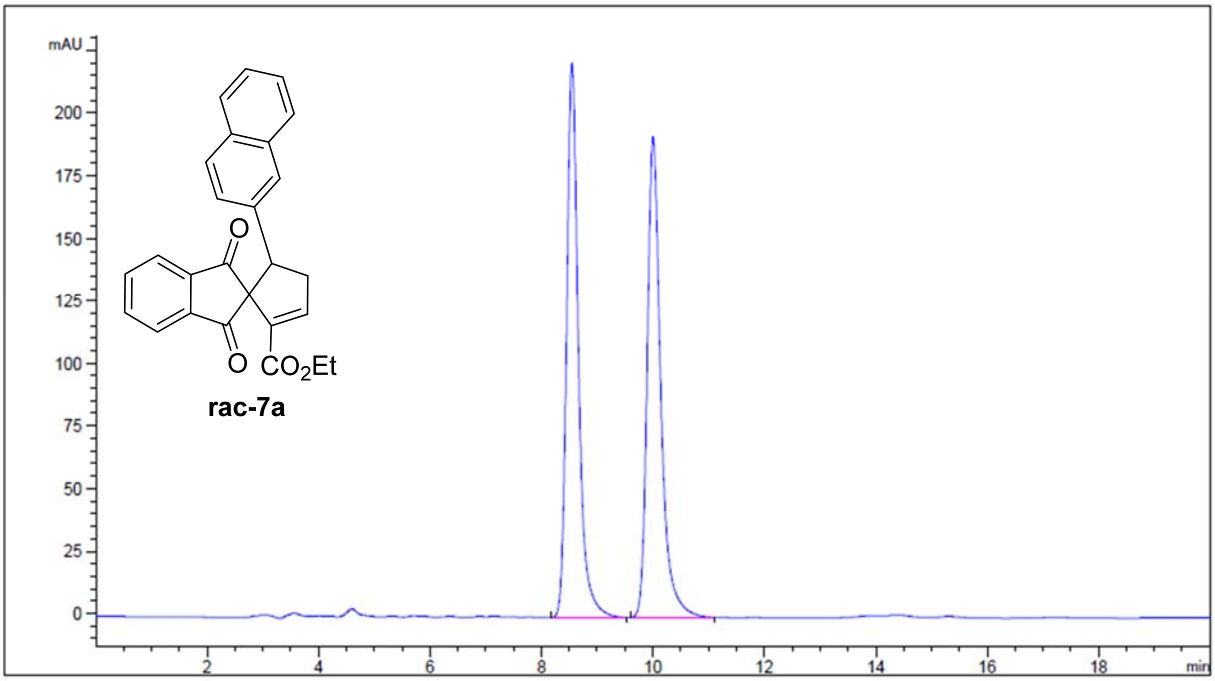

Peak #	RetTime [min]	Type	Width [min]	Area mAU *s	Area %
1	8.549	BB	0.2228	3264.54565	50.0526
2	10.004	BB	0.2546	3257.67847	49.9474

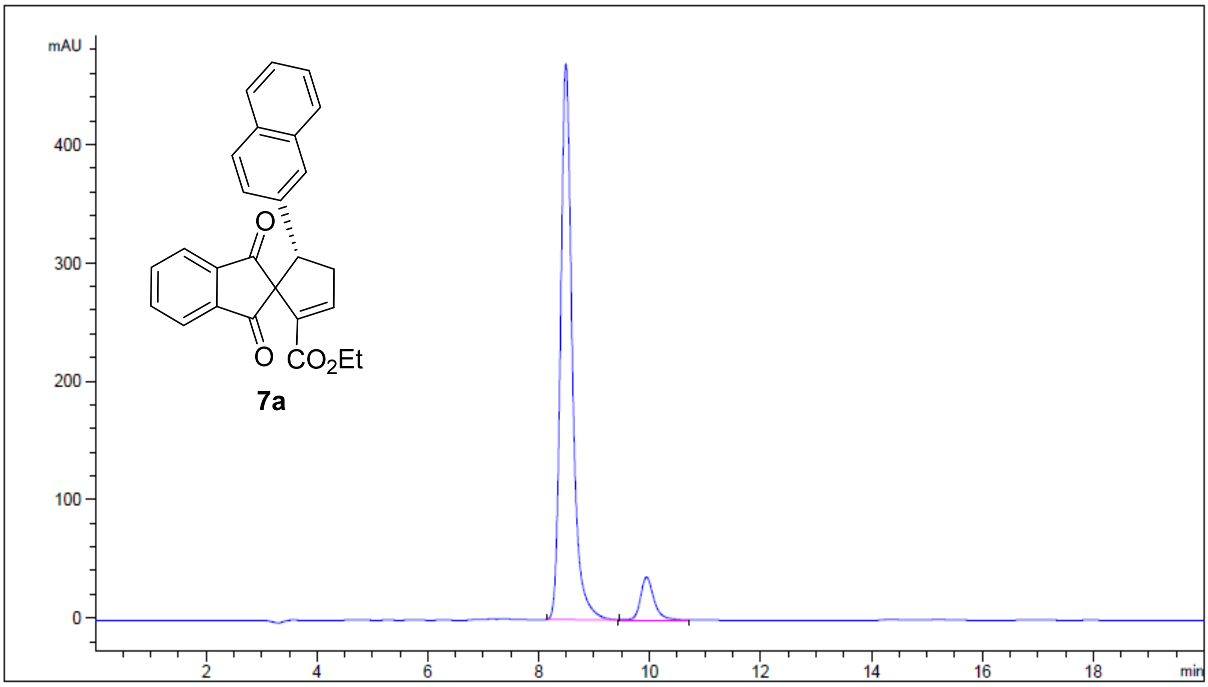

Peak #	RetTime [min]	Type	Width [min]	Area mAU *s	Area %
1	8.492	BB	0.2209	6874.68750	91.9032
2	9.946	BB	0.2516	605.66669	8.0968

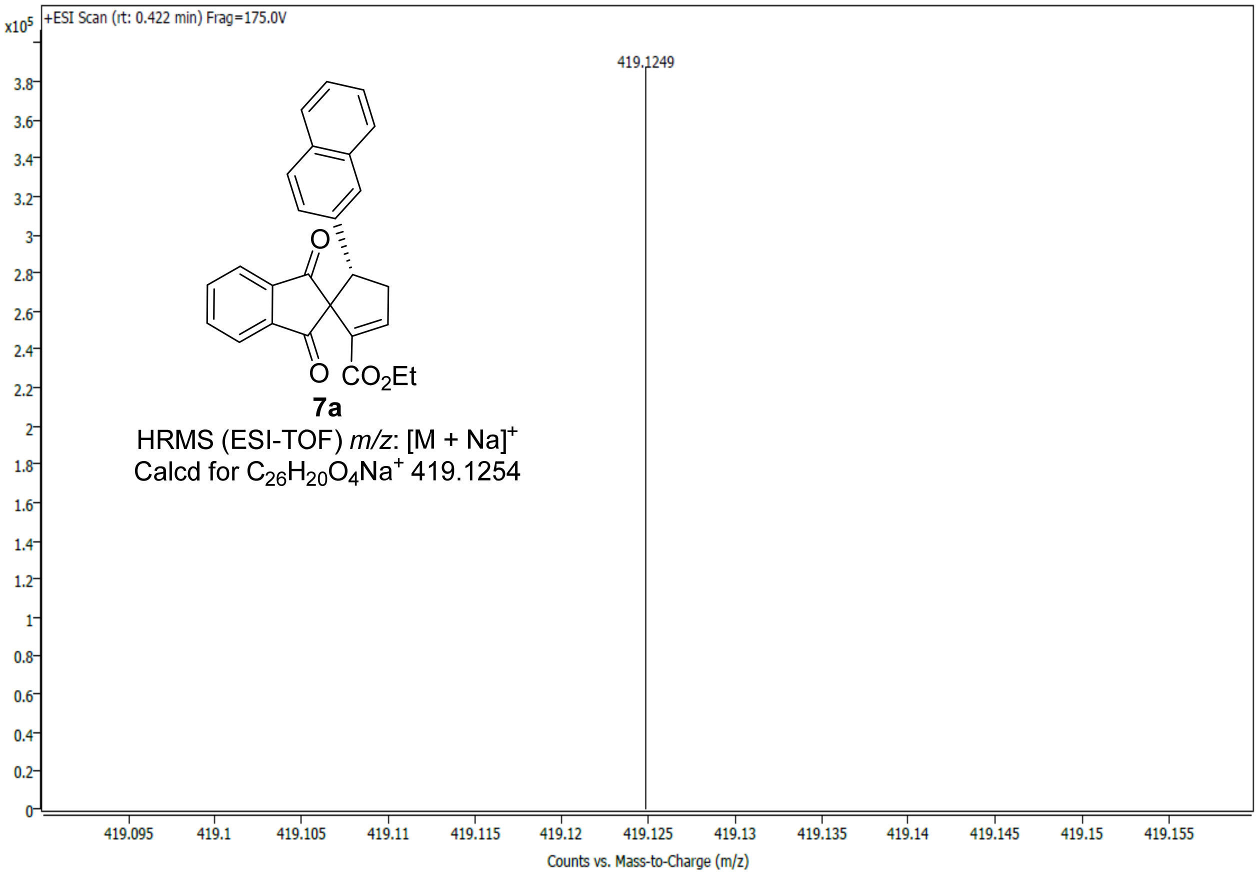
x10^5
+ESI Scan (rt: 0.422 min) Frag=175.0V
419.1249
7a
HRMS (ESI-TOF) m/z: [M + Na]+
Calcd for C26H20O4Na+ 419.1254
CO2Et
O
O
3.8
3.6
3.4
3.2
3
2.8
2.6
2.4
2.2
2
1.8
1.6
1.4
1.2
1
0.8
0.6
0.4
0.2
0
419.095
419.1
419.105
419.11
419.115
419.12
419.125
419.13
419.135
419.14
419.145
419.15
419.155
Counts vs. Mass-to-Charge (m/z)

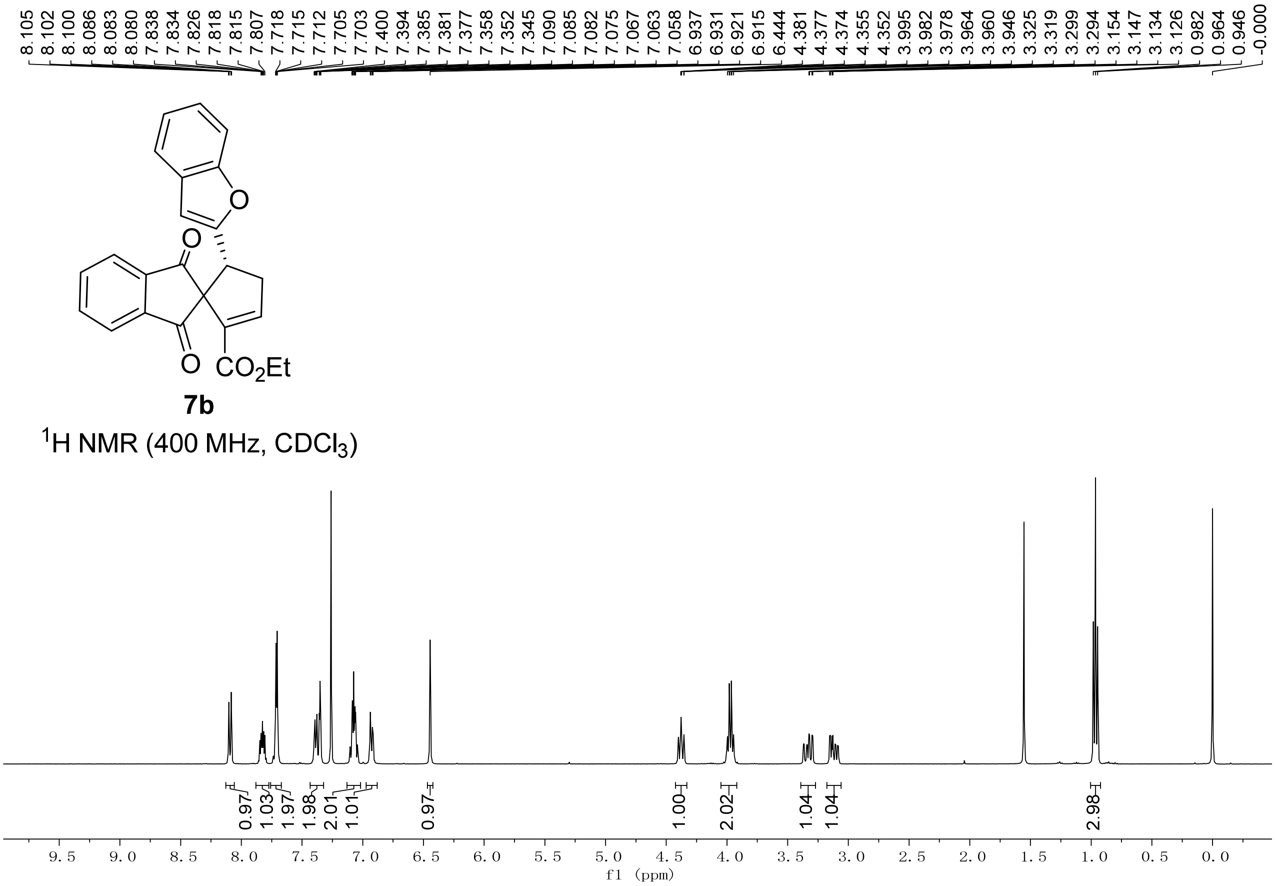

7b

^{1}H NMR (400 MHz, $CDCl_3$)

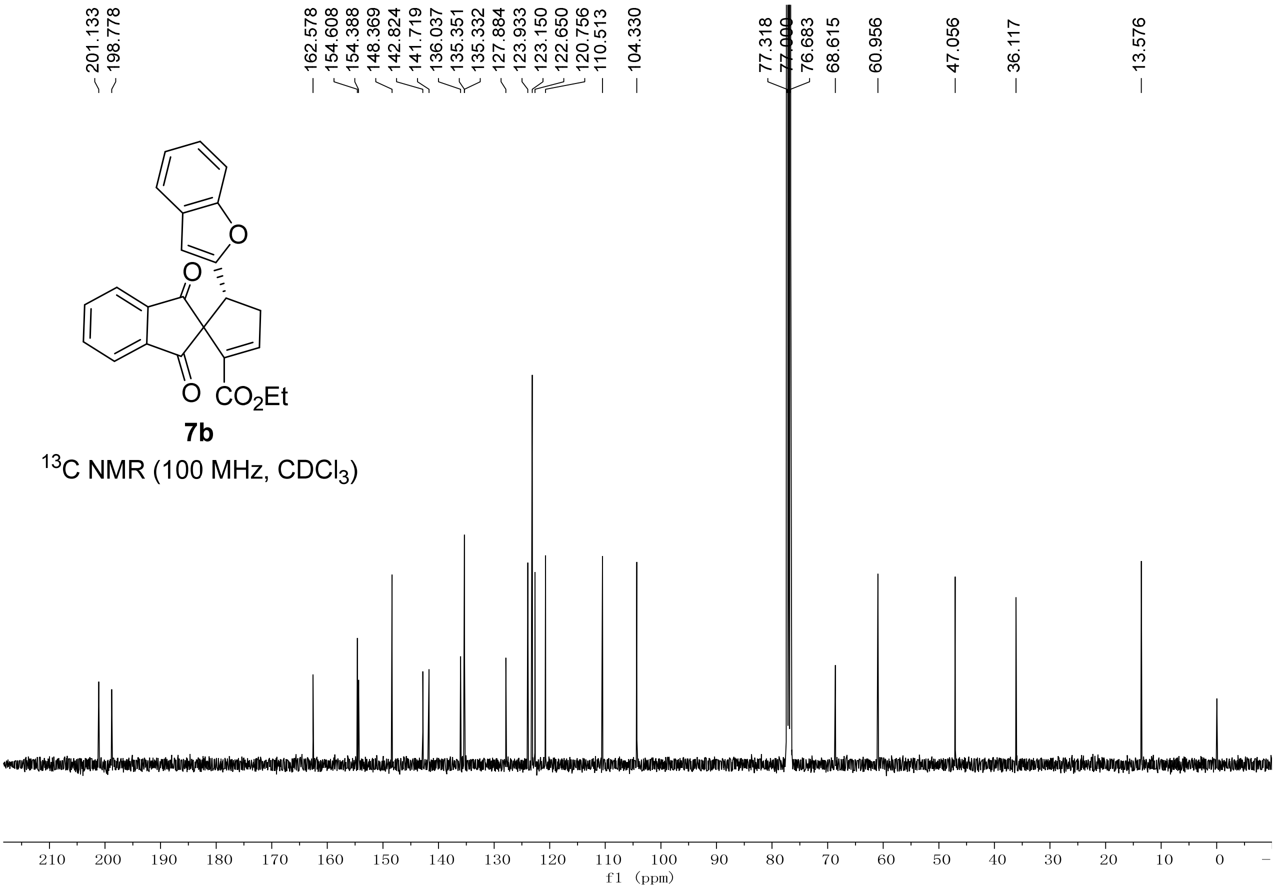

7b

^{13}C NMR (100 MHz, $CDCl_3$)

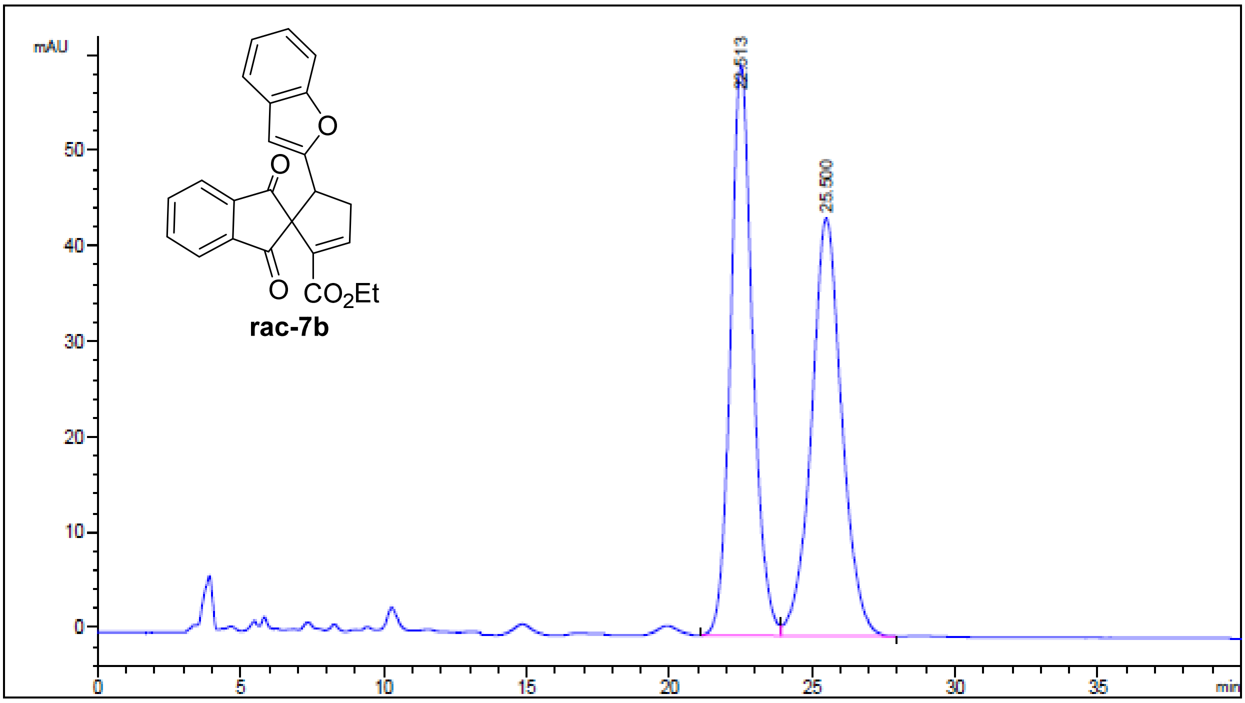

Peak #	RetTime [min]	Type	Width [min]	Area mAU *s	Height [mAU]	Area %
1	22.513	BV	0.8091	3216.15088	59.69376	49.8652
2	25.500	VB	1.0956	3233.53613	43.72915	50.1348

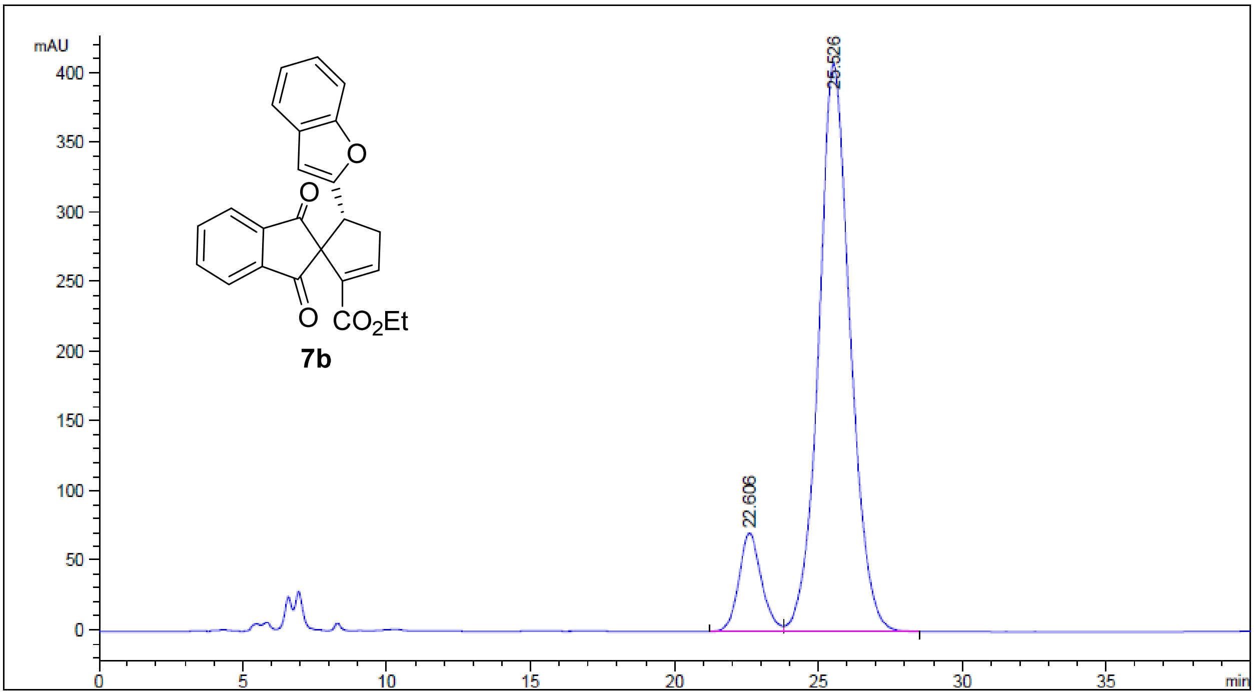

Peak #	RetTime [min]	Type	Width [min]	Area mAU *s	Height [mAU]	Area %
1	22.606	BV	0.8151	3839.58276	70.59535	11.0049
2	25.526	VB	1.1460	3.10501e4	407.12482	88.9951

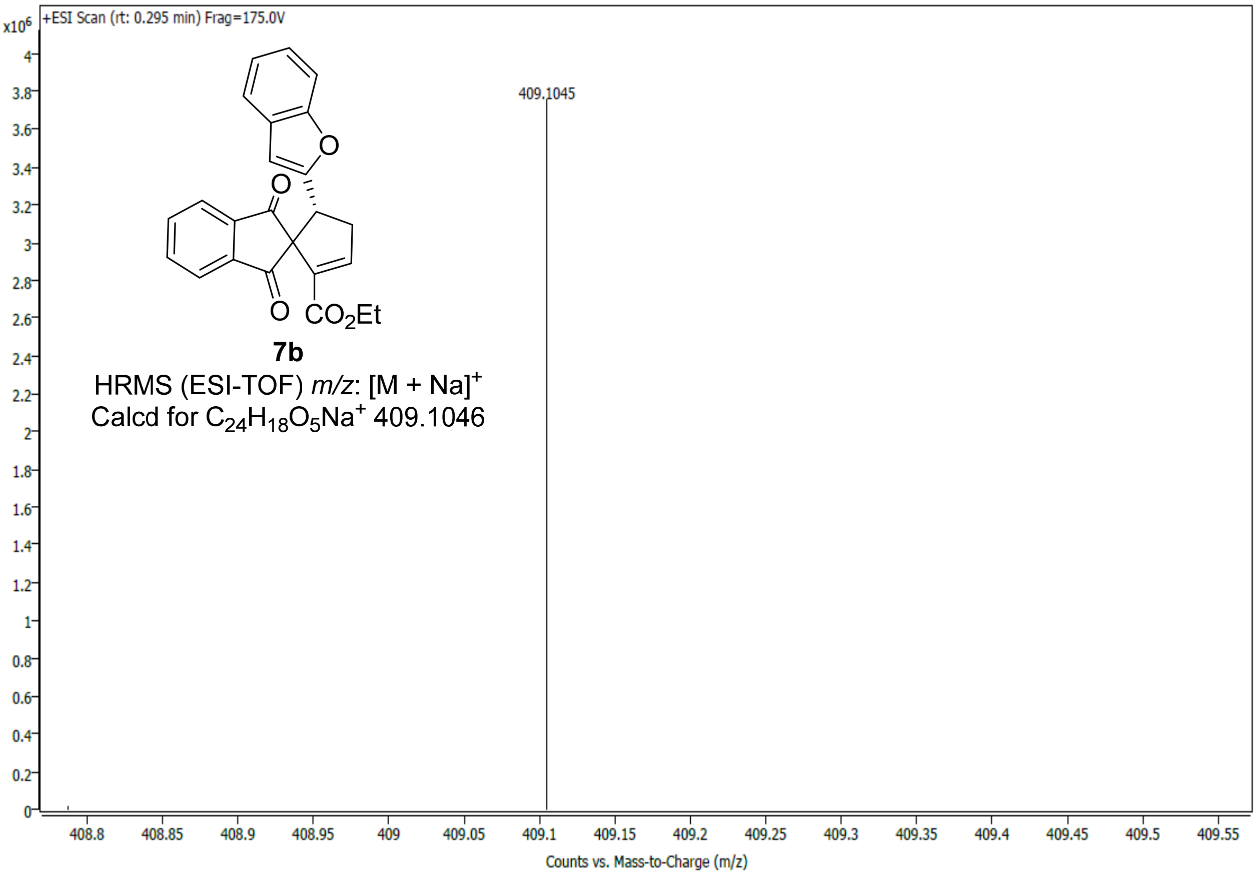
+ESI Scan (rt: 0.295 min) Frag=175.0V
x10^6
409.1045
7b
HRMS (ESI-TOF) m/z: [M + Na]+
Calcd for C24H18O5Na+ 409.1046
Counts vs. Mass-to-Charge (m/z)

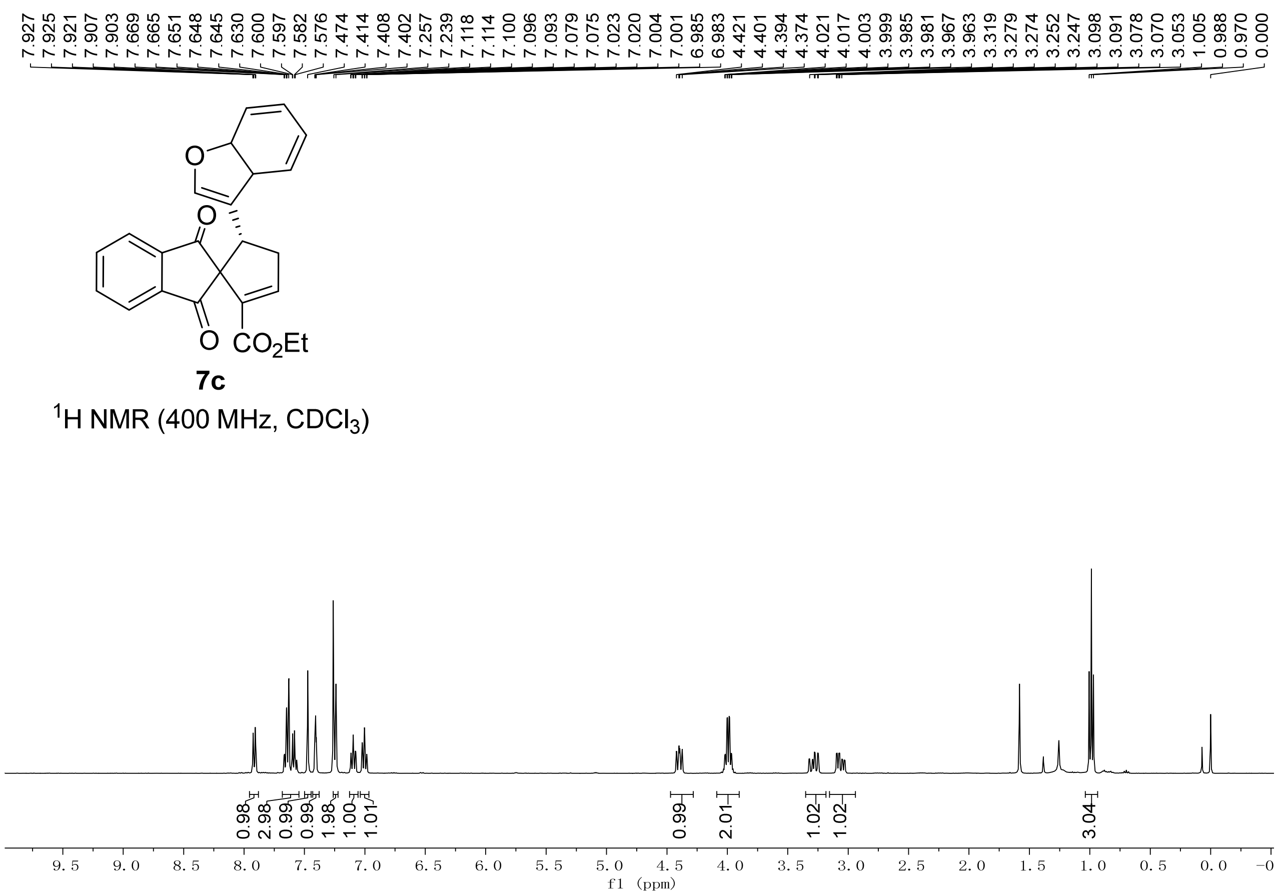
7c
^{1}H NMR (400 MHz, CDCl$_3$)
f1 (ppm)

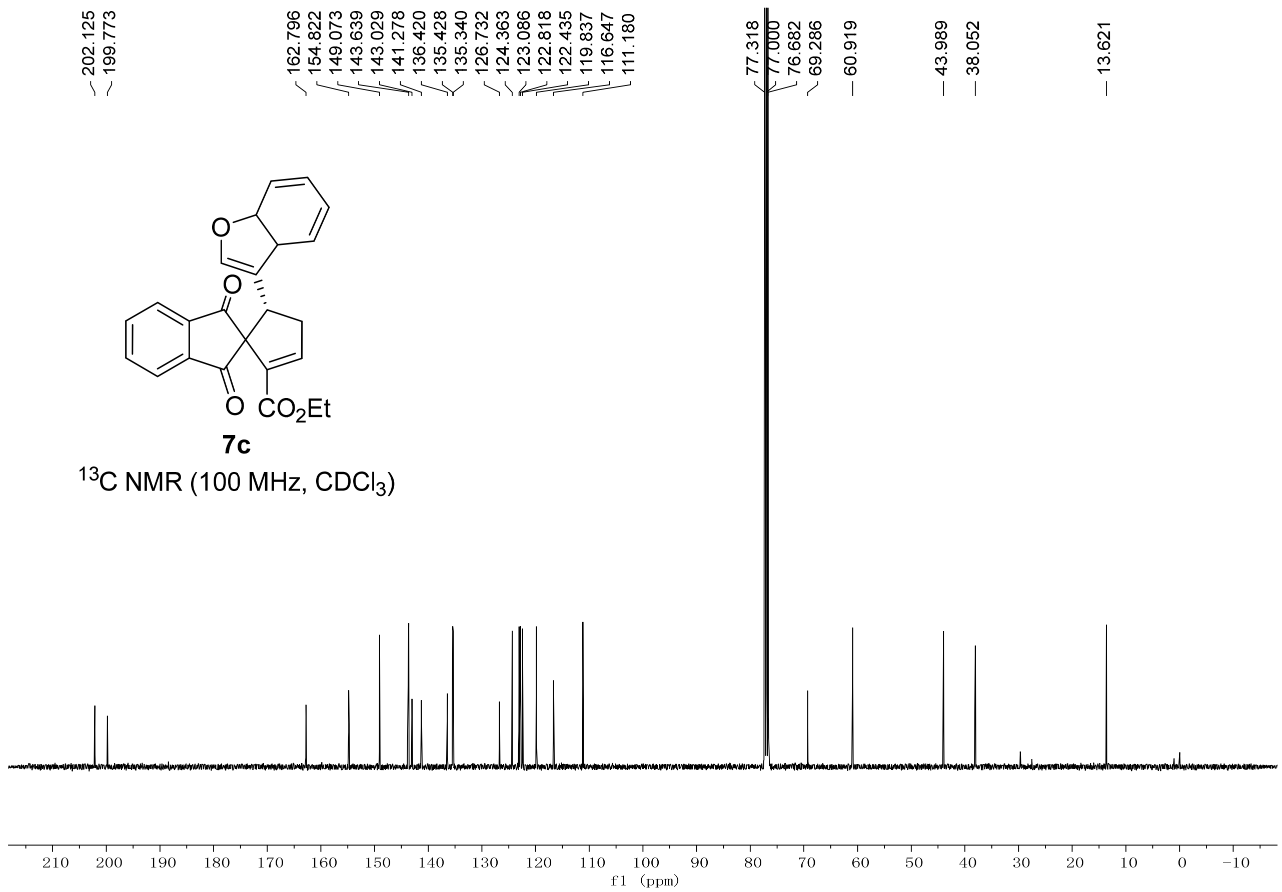
7c
^{13}C NMR (100 MHz, CDCl$_3$)
f1 (ppm)

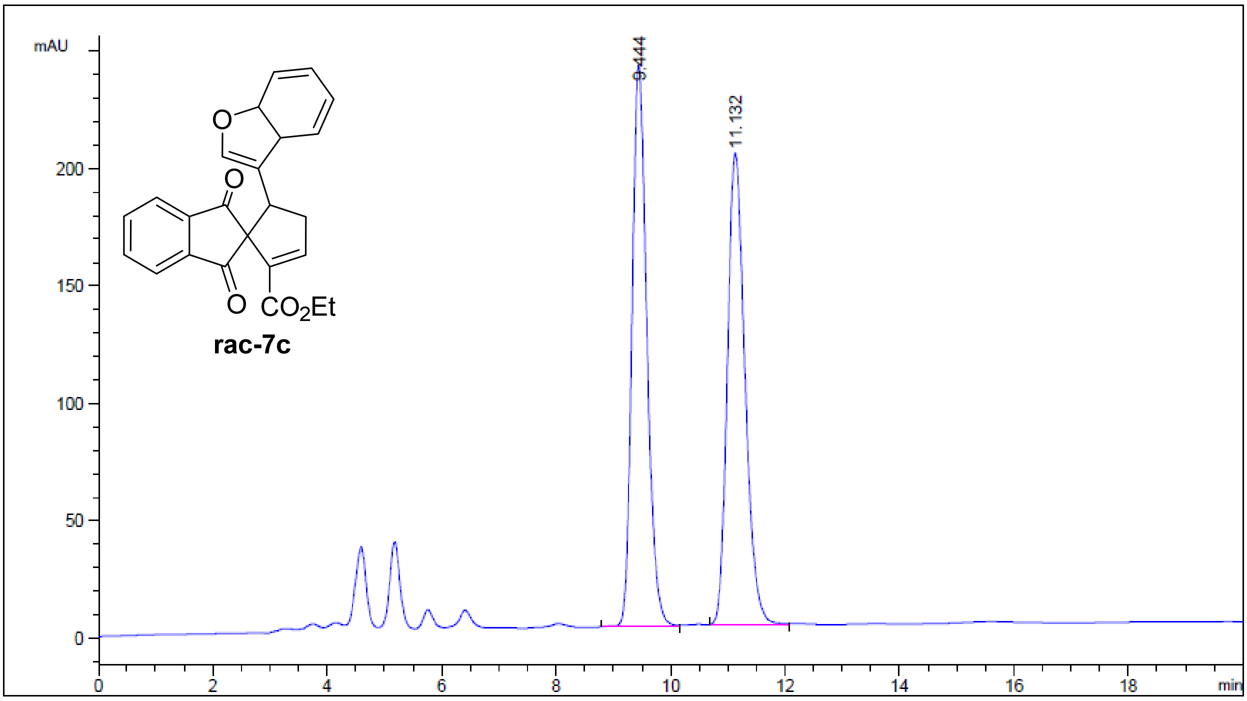

Peak #	RetTime [min]	Type	Width [min]	Area mAU *s	Height [mAU]	Area %
1	9.444	BV	0.2751	4328.84668	239.28012	50.1797
2	11.132	BBA	0.3254	4297.83447	200.85231	49.8203

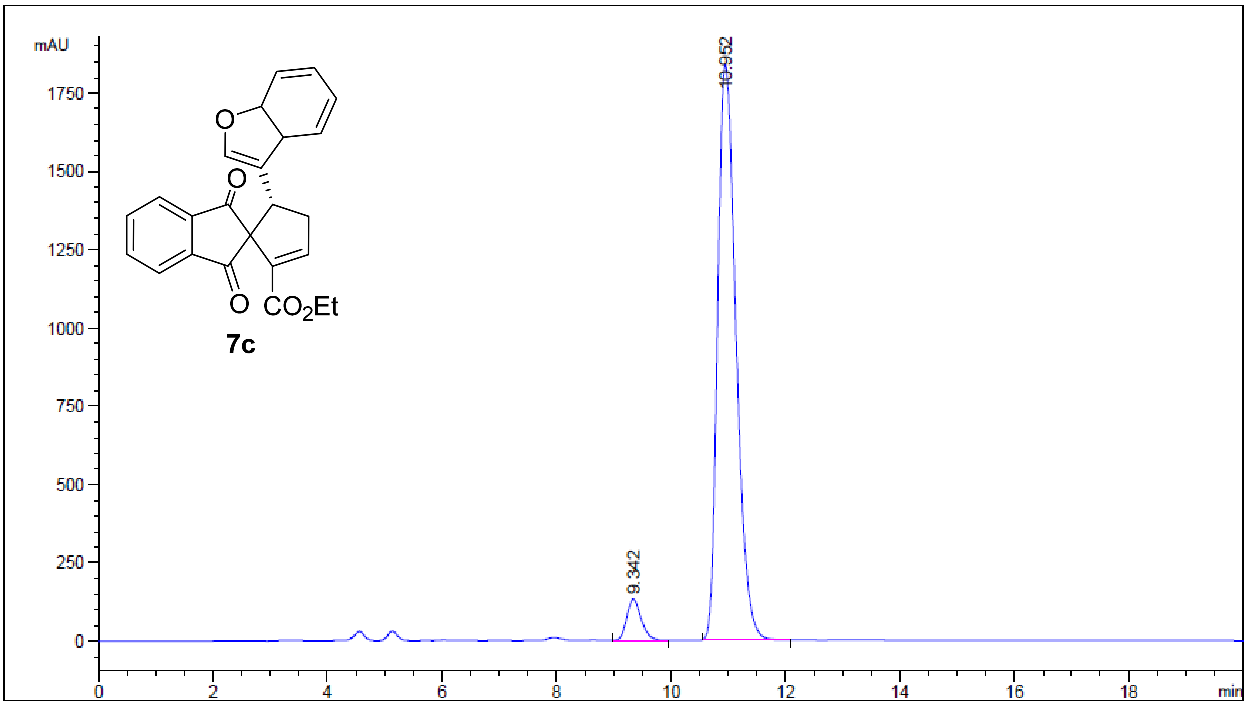

Peak #	RetTime [min]	Type	Width [min]	Area mAU *s	Height [mAU]	Area %
1	9.342	BV	0.2704	2312.82324	130.74126	5.3647
2	10.952	BBA	0.3467	4.07988e4	1835.54053	94.6353

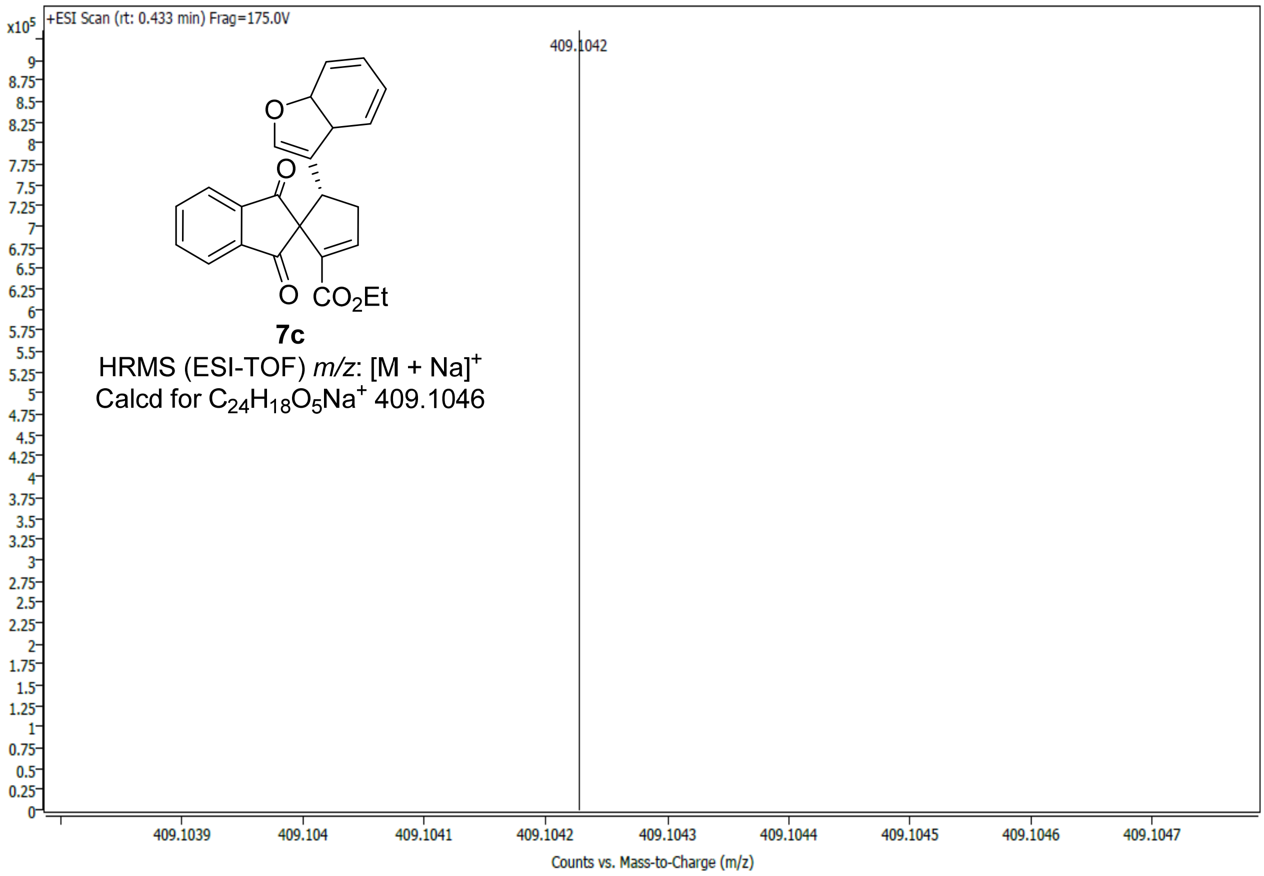
+ESI Scan (rt: 0.433 min) Frag=175.0V
x10^5
409.1042
7c
HRMS (ESI-TOF) m/z: [M + Na]+
Calcd for C24H18O5Na+ 409.1046
CO2Et
Counts vs. Mass-to-Charge (m/z)

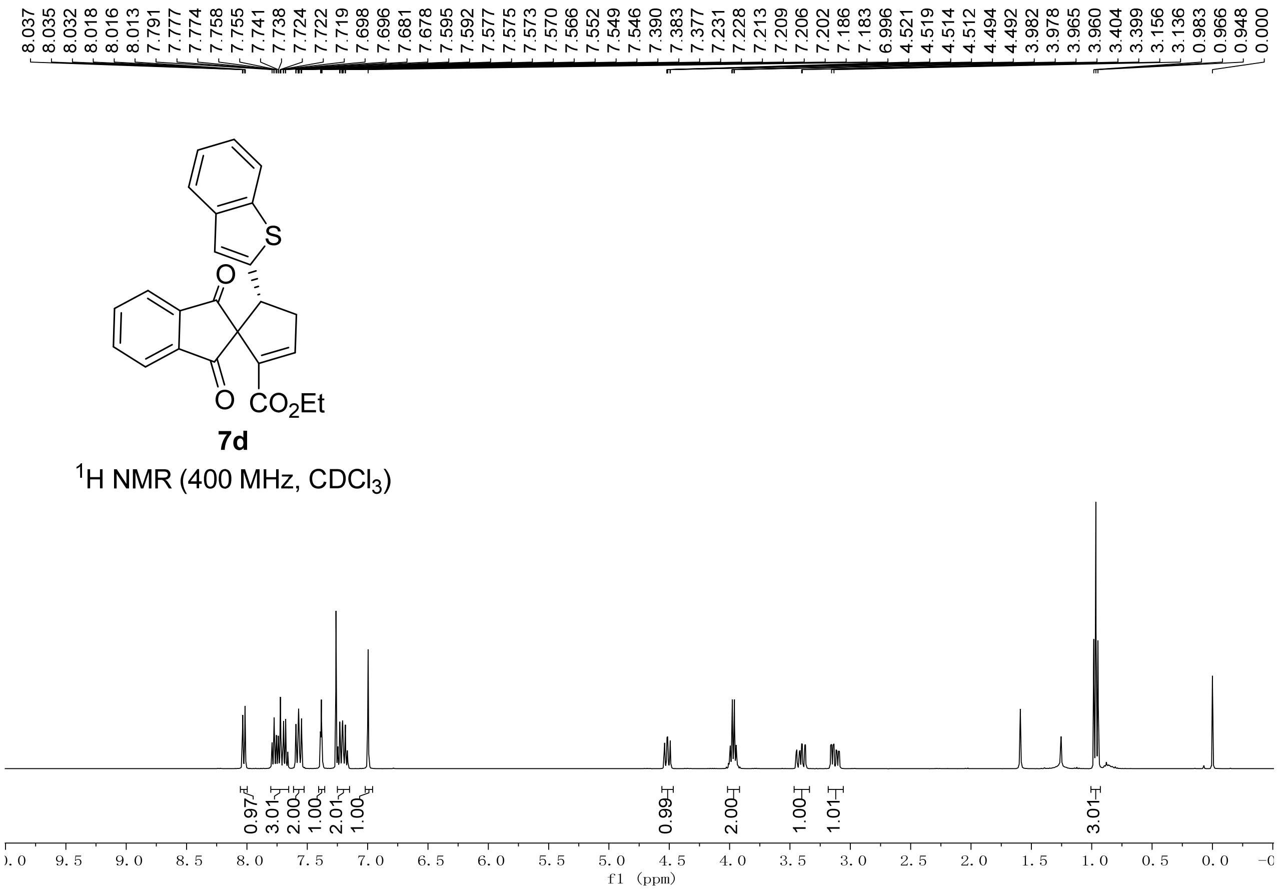
8.037
8.035
8.032
8.018
8.016
8.013
7.791
7.777
7.774
7.758
7.755
7.741
7.738
7.724
7.722
7.719
7.698
7.696
7.681
7.678
7.595
7.592
7.577
7.575
7.573
7.570
7.566
7.552
7.549
7.546
7.390
7.383
7.377
7.231
7.228
7.213
7.209
7.206
7.202
7.186
7.183
6.996
4.521
4.519
4.514
4.512
4.494
4.492
3.982
3.978
3.965
3.960
3.404
3.399
3.156
3.136
0.983
0.966
0.948
0.000
O
S
O
CO2Et
7d
1H NMR (400 MHz, CDCl3)
0.97
3.01
2.00
1.00
2.01
1.00
0.99
2.00
1.00
1.01
3.01
f1 (ppm)

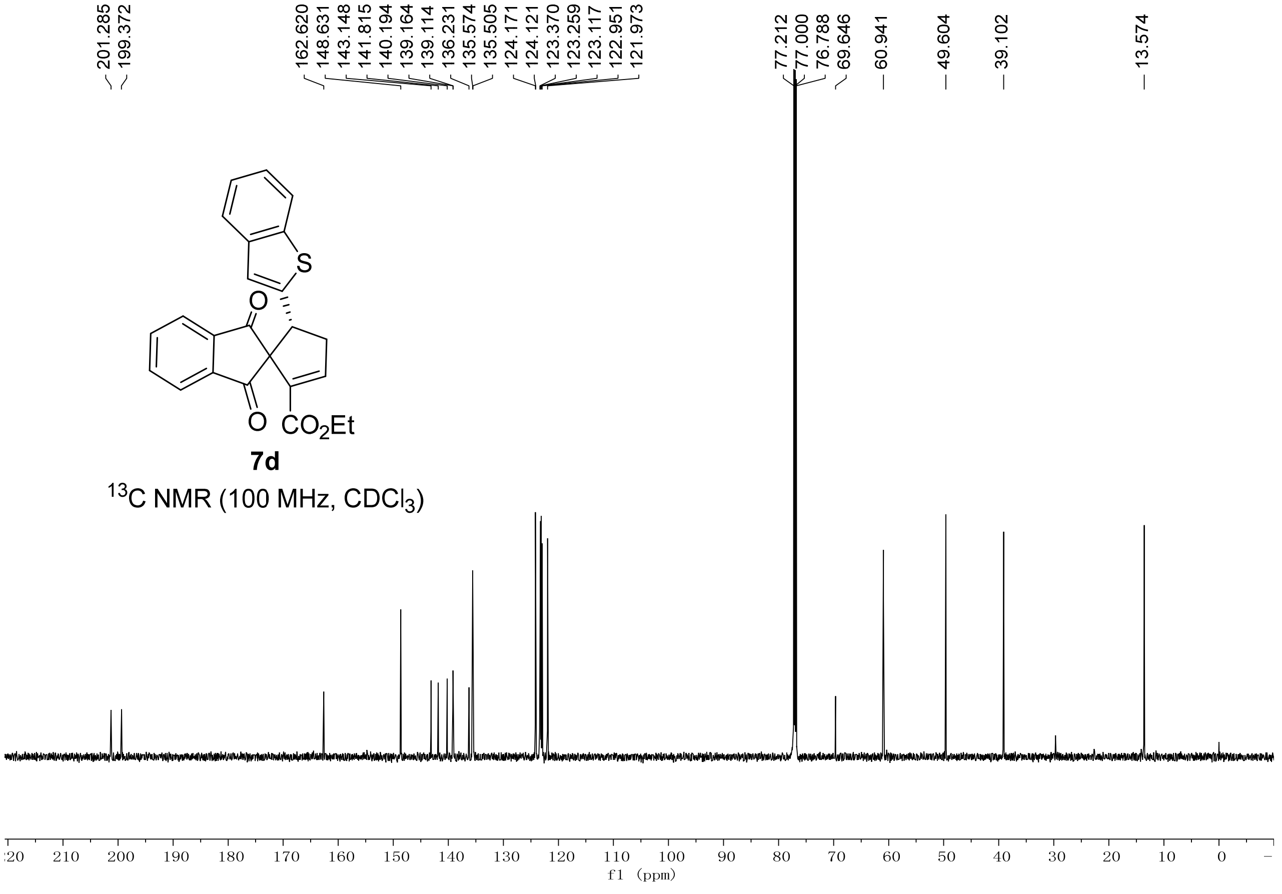
201.285
199.372
162.620
148.631
143.148
141.815
140.194
139.164
139.114
136.231
135.574
135.505
124.171
124.121
123.370
123.259
123.117
122.951
121.973
77.212
77.000
76.788
69.646
60.941
49.604
39.102
13.574
O
S
O
CO2Et
7d
13C NMR (100 MHz, CDCl3)
f1 (ppm)

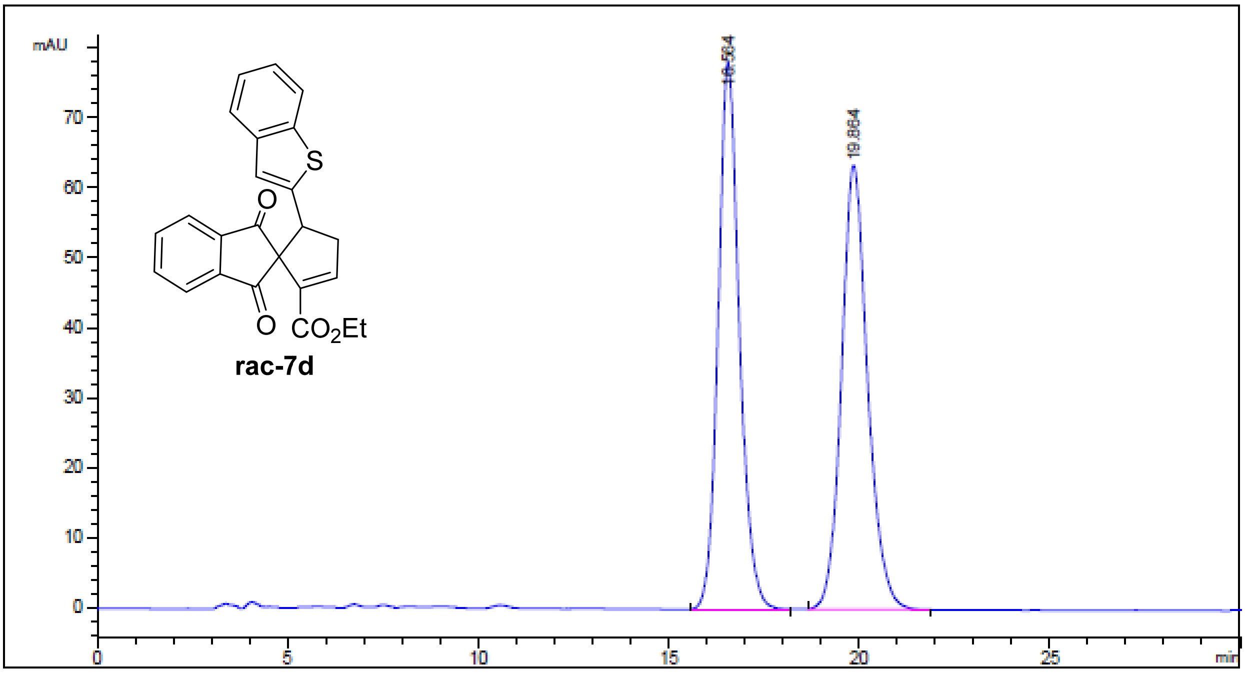

Peak #	RetTime [min]	Type	Width [min]	Area mAU *s	Height [mAU]	Area %
1	16.564	BB	0.5808	3030.08594	78.08745	49.9853
2	19.864	BB	0.7207	3031.86963	63.42781	50.0147

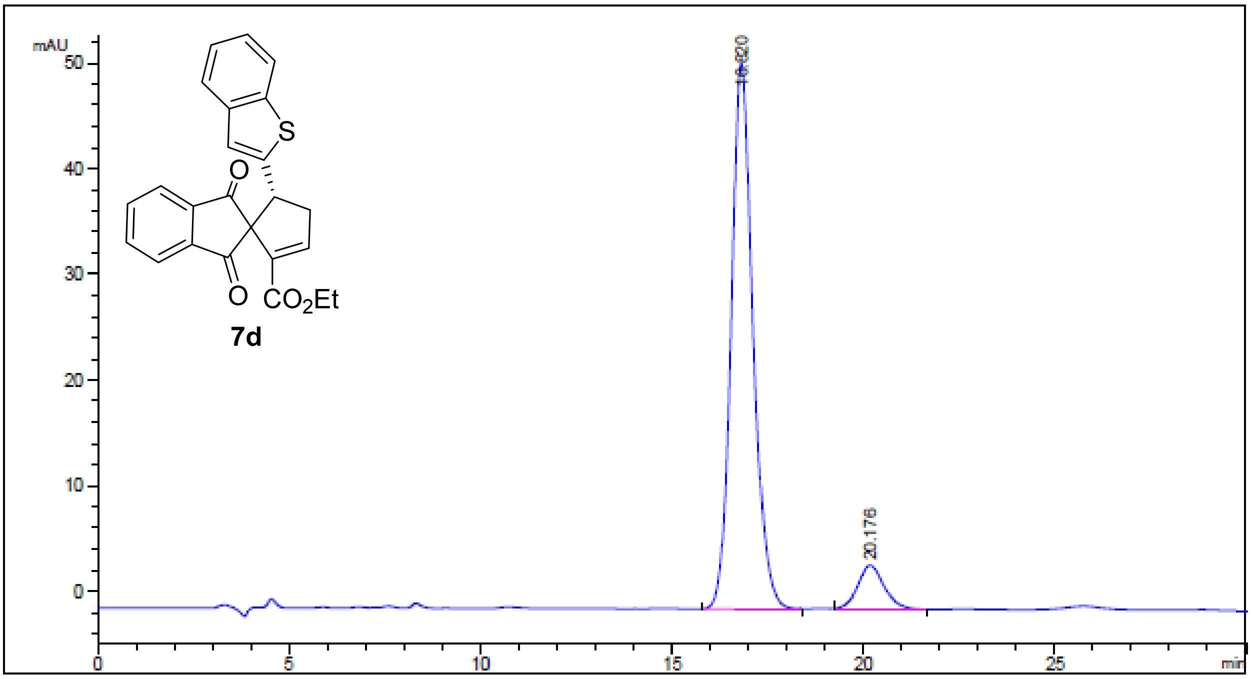

Peak #	RetTime [min]	Type	Width [min]	Area mAU *s	Height [mAU]	Area %
1	16.820	BB	0.5950	2042.55835	51.69043	91.0236
2	20.176	BB	0.7217	201.42900	4.16336	8.9764

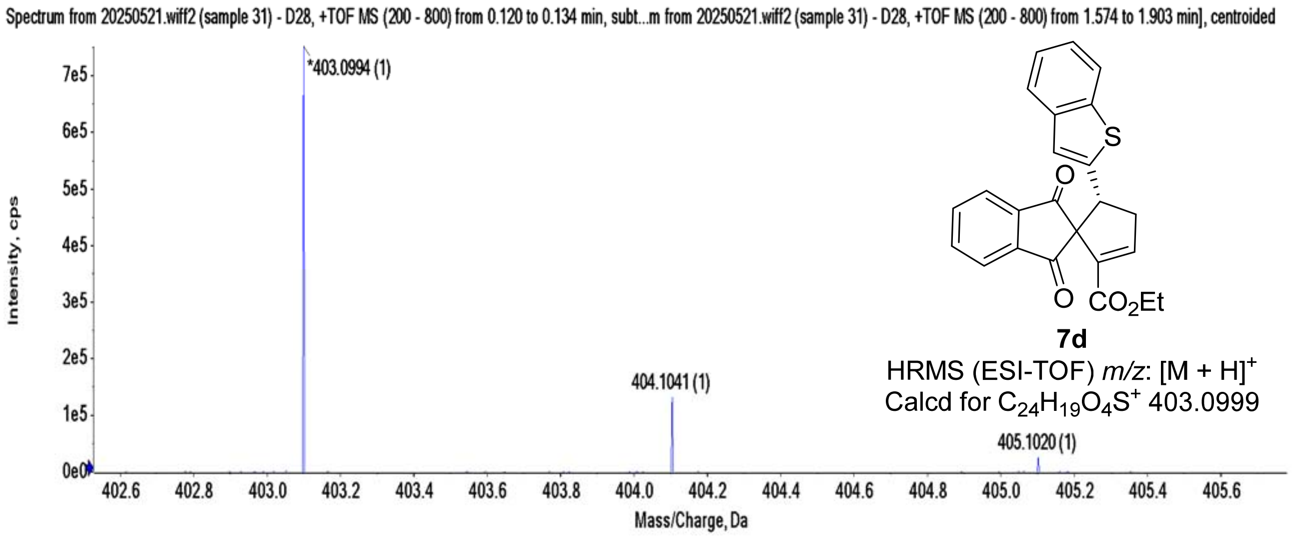

7d

HRMS (ESI-TOF) *m/z*: $[M + H]^+$

Calcd for $C_{24}H_{19}O_4S^+$ 403.0999

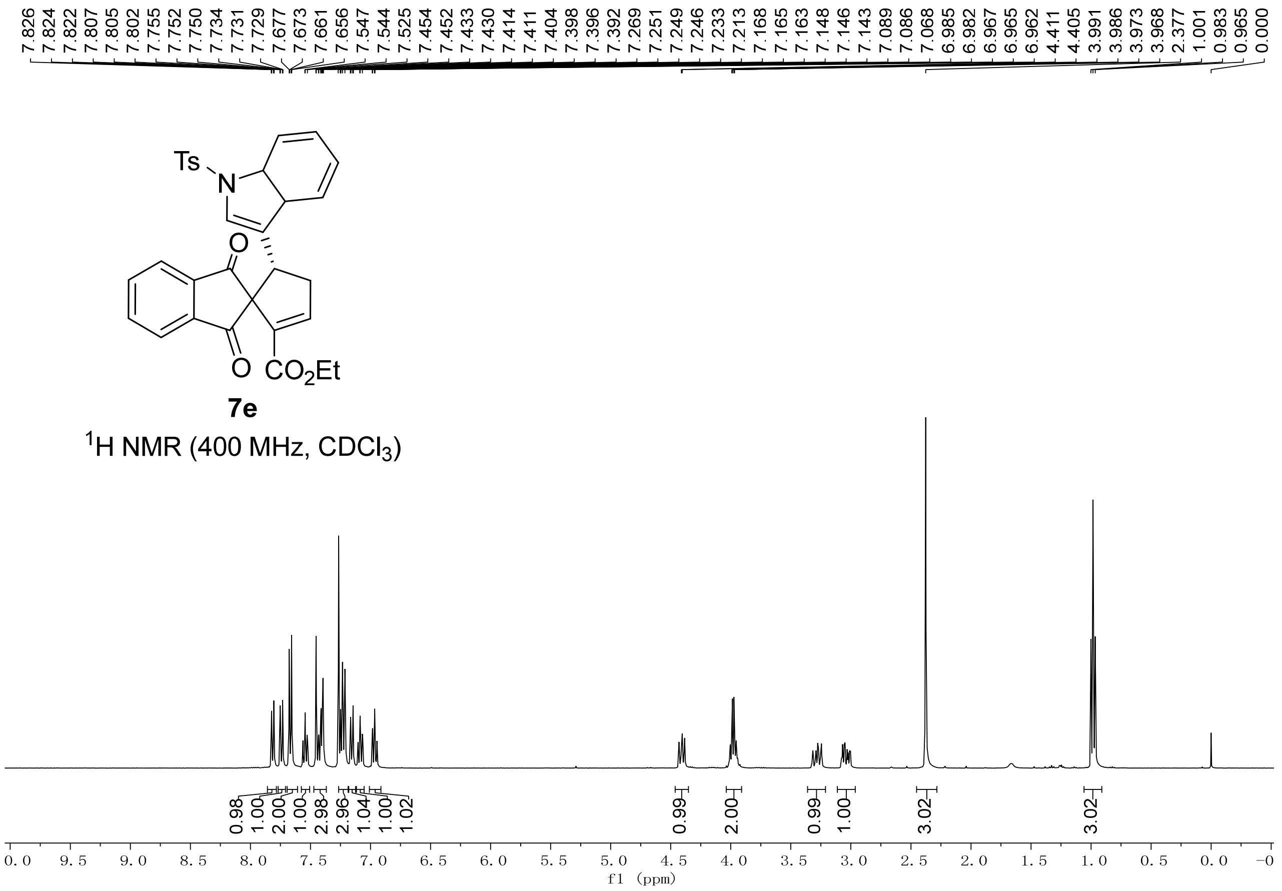
7.826
7.824
7.822
7.807
7.805
7.802
7.755
7.752
7.750
7.734
7.731
7.729
7.677
7.673
7.661
7.656
7.547
7.544
7.525
7.454
7.452
7.433
7.430
7.414
7.411
7.404
7.398
7.396
7.392
7.269
7.251
7.249
7.246
7.233
7.213
7.168
7.165
7.163
7.148
7.146
7.143
7.089
7.086
7.068
6.985
6.982
6.967
6.965
6.962
4.411
4.405
3.991
3.986
3.973
3.968
2.377
1.001
0.983
0.965
0.000
Ts
N
O
O
CO2Et
7e
1H NMR (400 MHz, CDCl3)
0.98
1.00
2.00
1.00
2.98
2.96
1.04
1.00
1.02
0.99
2.00
0.99
1.00
3.02
3.02
f1 (ppm)

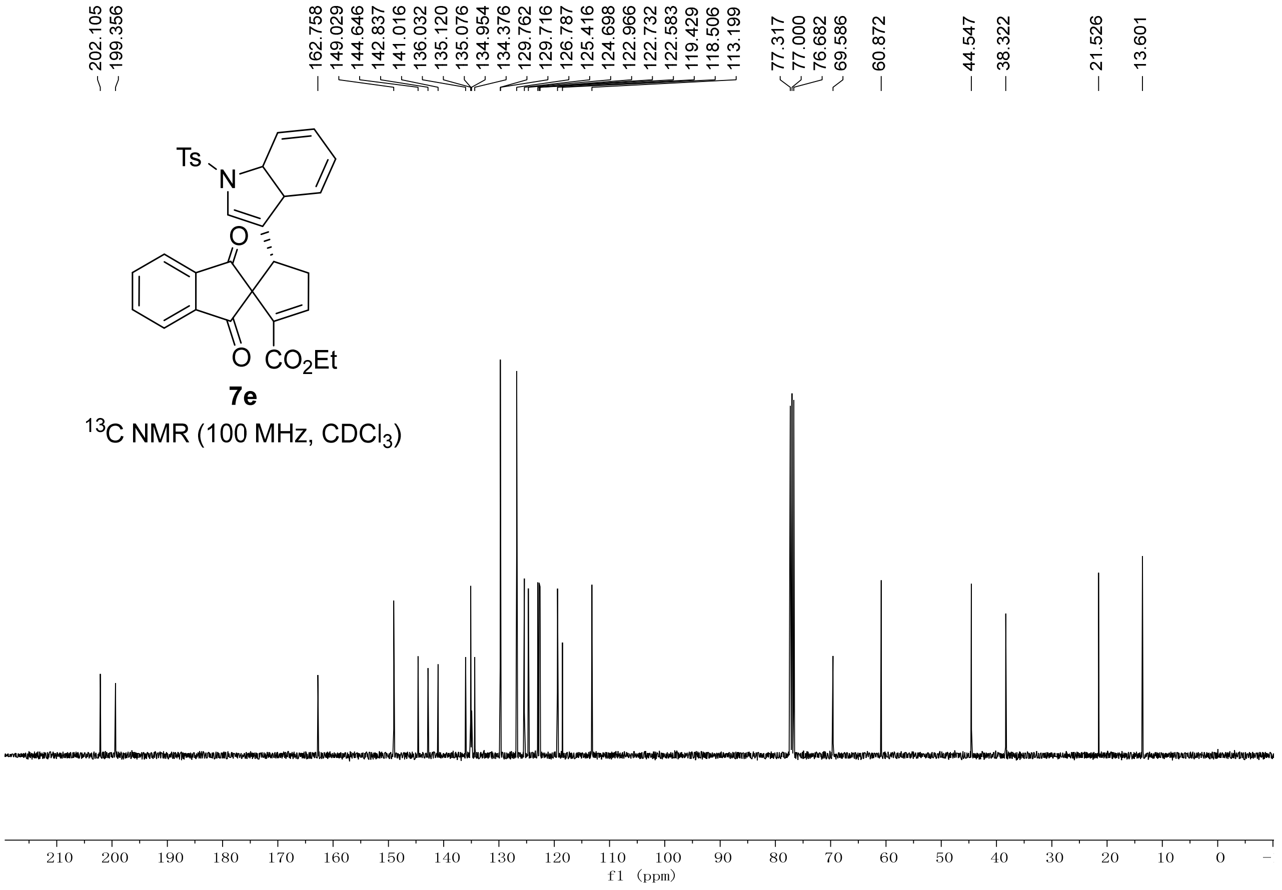
202.105
199.356
162.758
149.029
144.646
142.837
141.016
136.032
135.120
135.076
134.954
134.376
129.762
129.716
126.787
125.416
124.698
122.966
122.732
122.583
119.429
118.506
113.199
77.317
77.000
76.682
69.586
60.872
44.547
38.322
21.526
13.601
Ts
N
O
O
CO2Et
7e
13C NMR (100 MHz, CDCl3)
f1 (ppm)

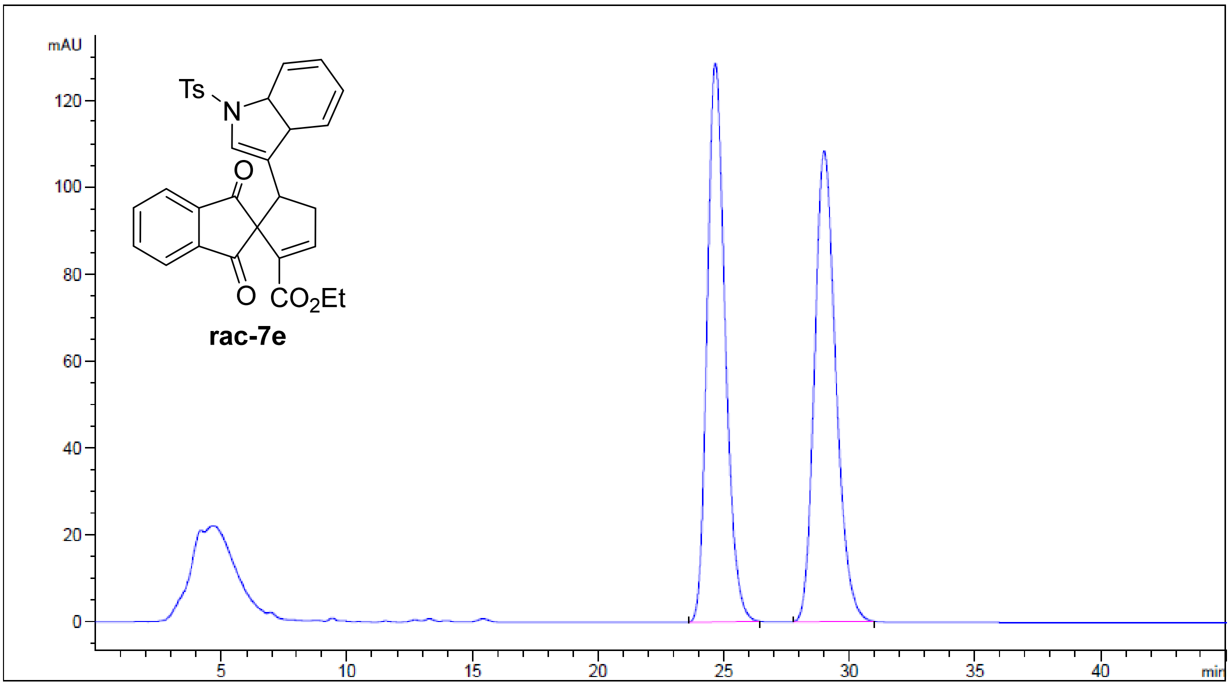

Peak #	RetTime [min]	Type	Width [min]	Area mAU *s	Area %
1	24.669	BB	0.7658	6411.53271	50.0282
2	29.004	BB	0.9069	6404.29688	49.9718

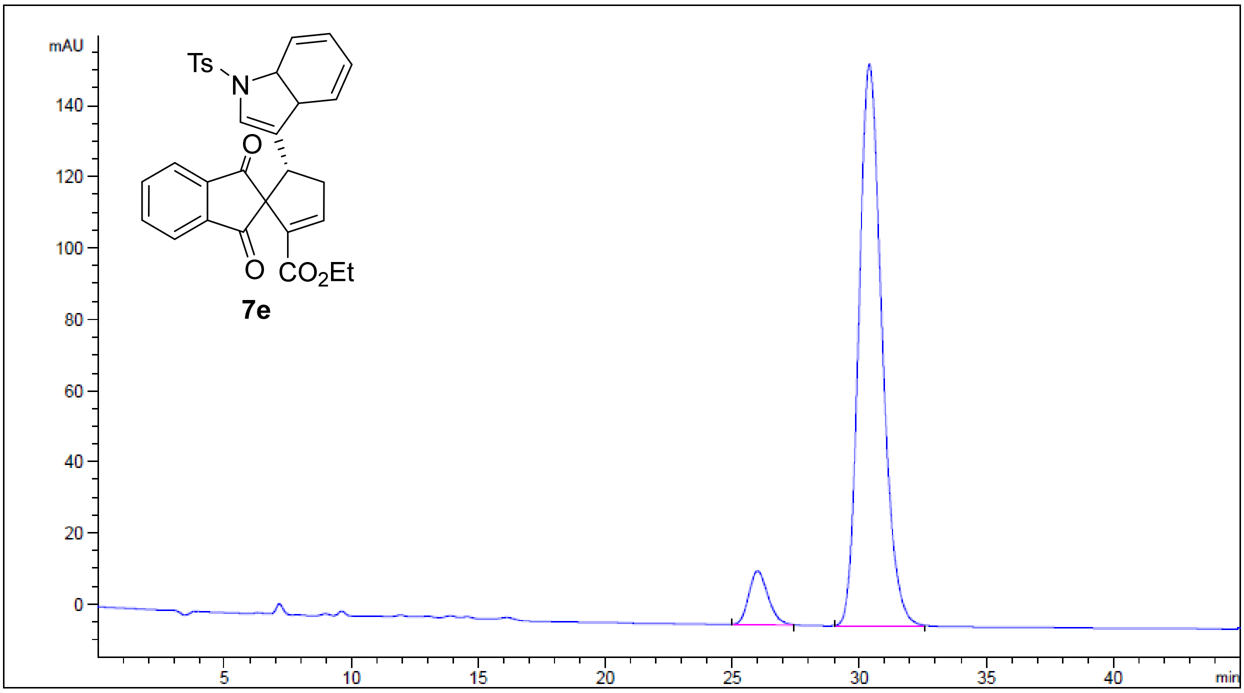

Peak #	RetTime [min]	Type	Width [min]	Area mAU *s	Area %
1	25.995	BB	0.8138	801.19525	7.4384
2	30.398	BB	0.9735	9969.92480	92.5616

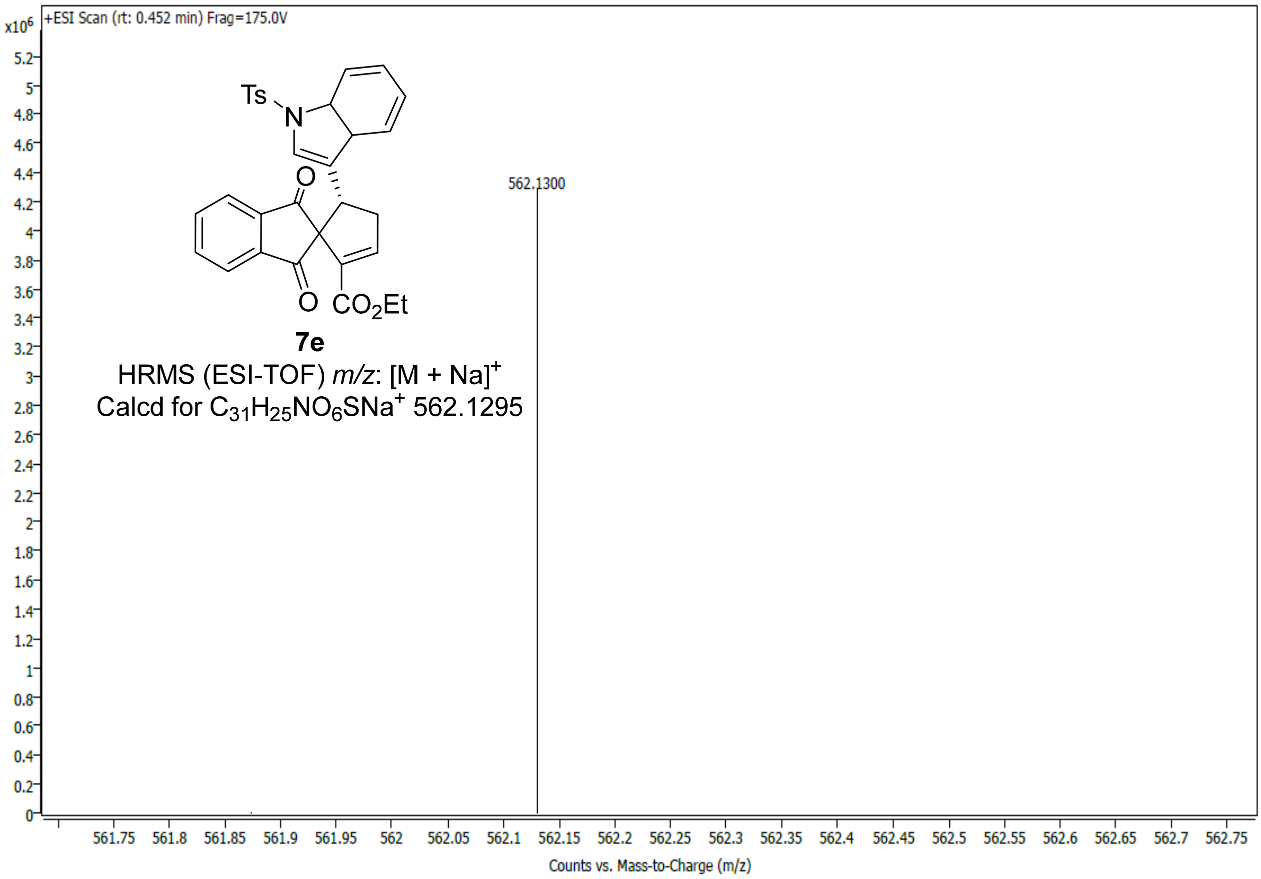
+ESI Scan (rt: 0.452 min) Frag=175.0V
x10^6
Ts
N
O
O
CO2Et
7e
HRMS (ESI-TOF) m/z: [M + Na]+
Calcd for C31H25NO6SNa+ 562.1295
562.1300
Counts vs. Mass-to-Charge (m/z)

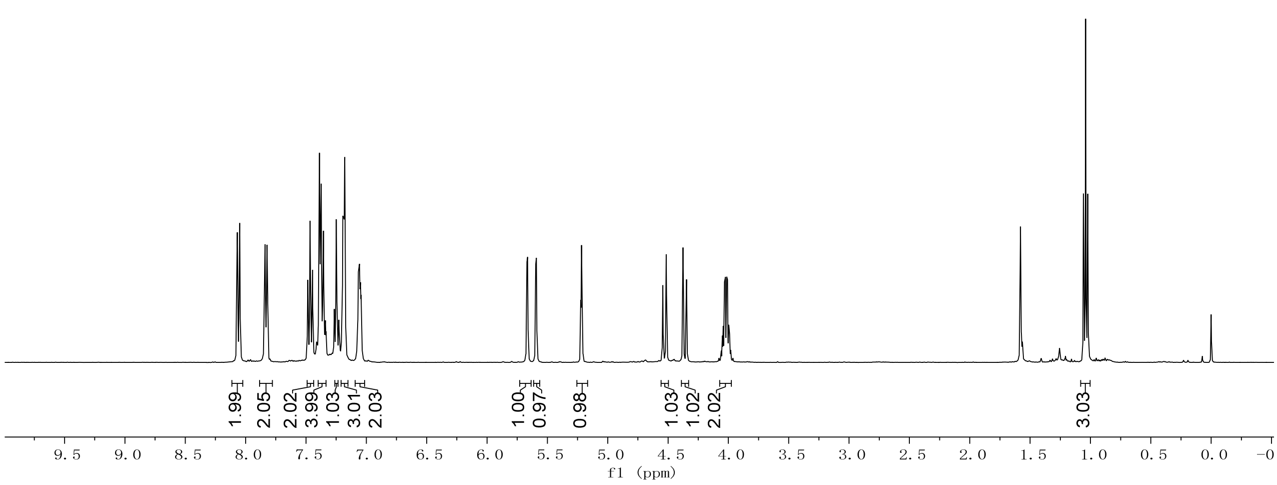

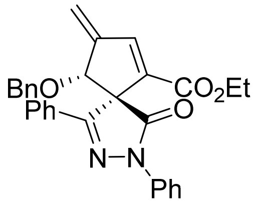

9a

^{1}H NMR (400 MHz, $CDCl_3$)

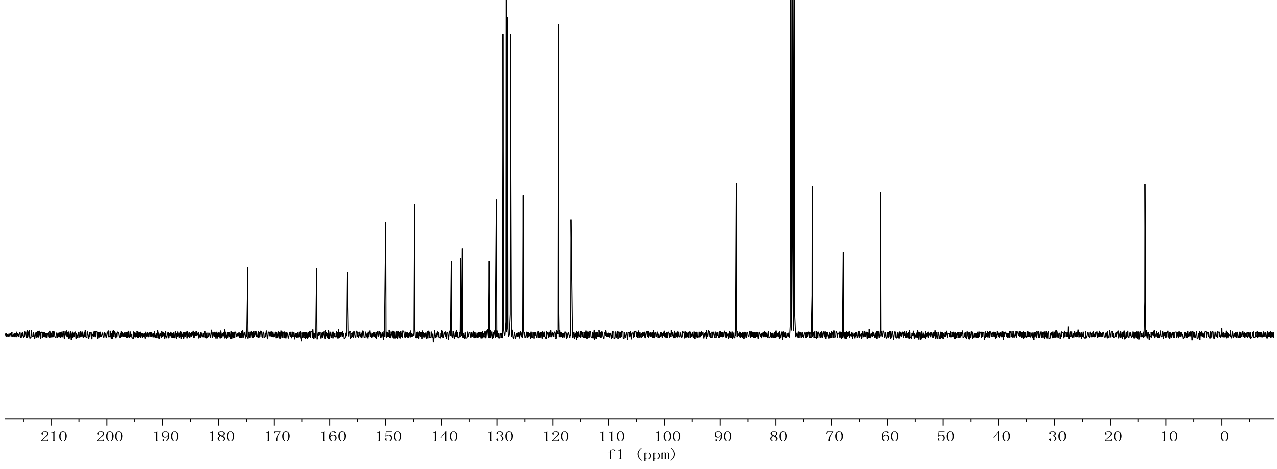

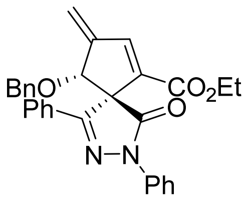

9a

^{13}C NMR (100 MHz, $CDCl_3$)

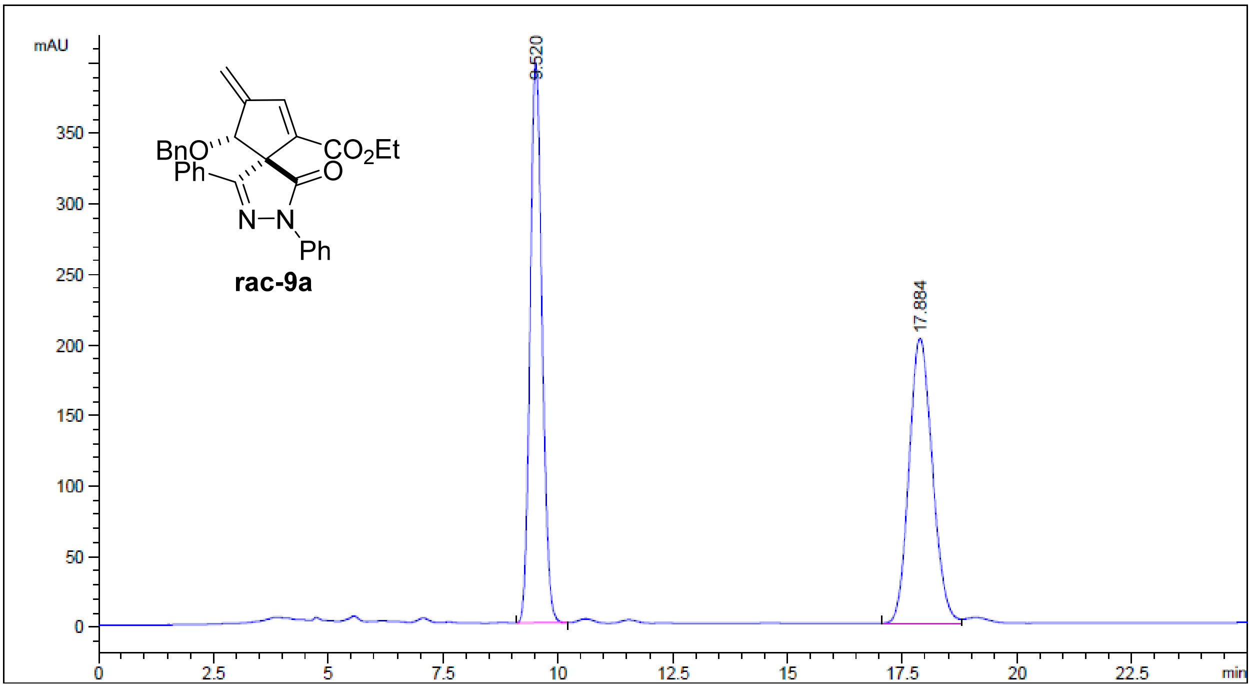

Peak #	RetTime [min]	Type	Width [min]	Area mAU *s	Height [mAU]	Area %
1	9.520	BV	0.2879	7202.03418	395.99042	50.1671
2	17.884	BV	0.5471	7154.05859	202.01733	49.8329

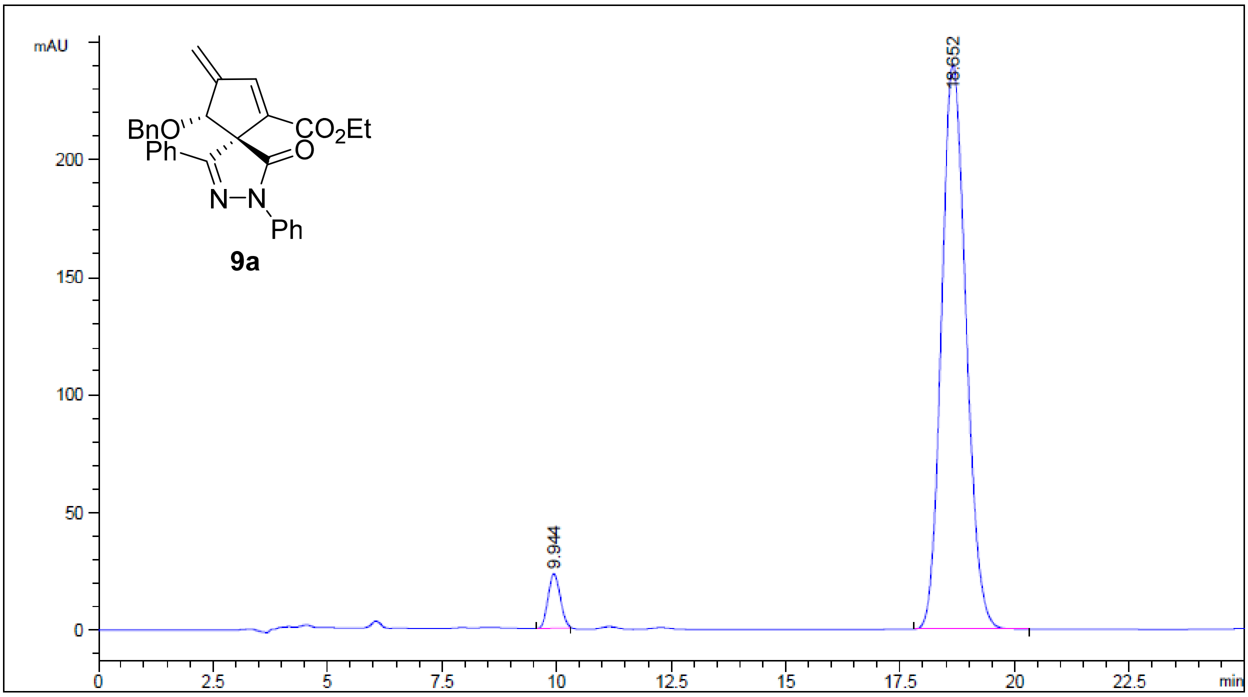

Peak #	RetTime [min]	Type	Width [min]	Area mAU *s	Height [mAU]	Area %
1	9.944	BBA	0.2929	432.10681	23.21045	4.6109
2	18.652	BB	0.5801	8939.29883	240.04291	95.3891

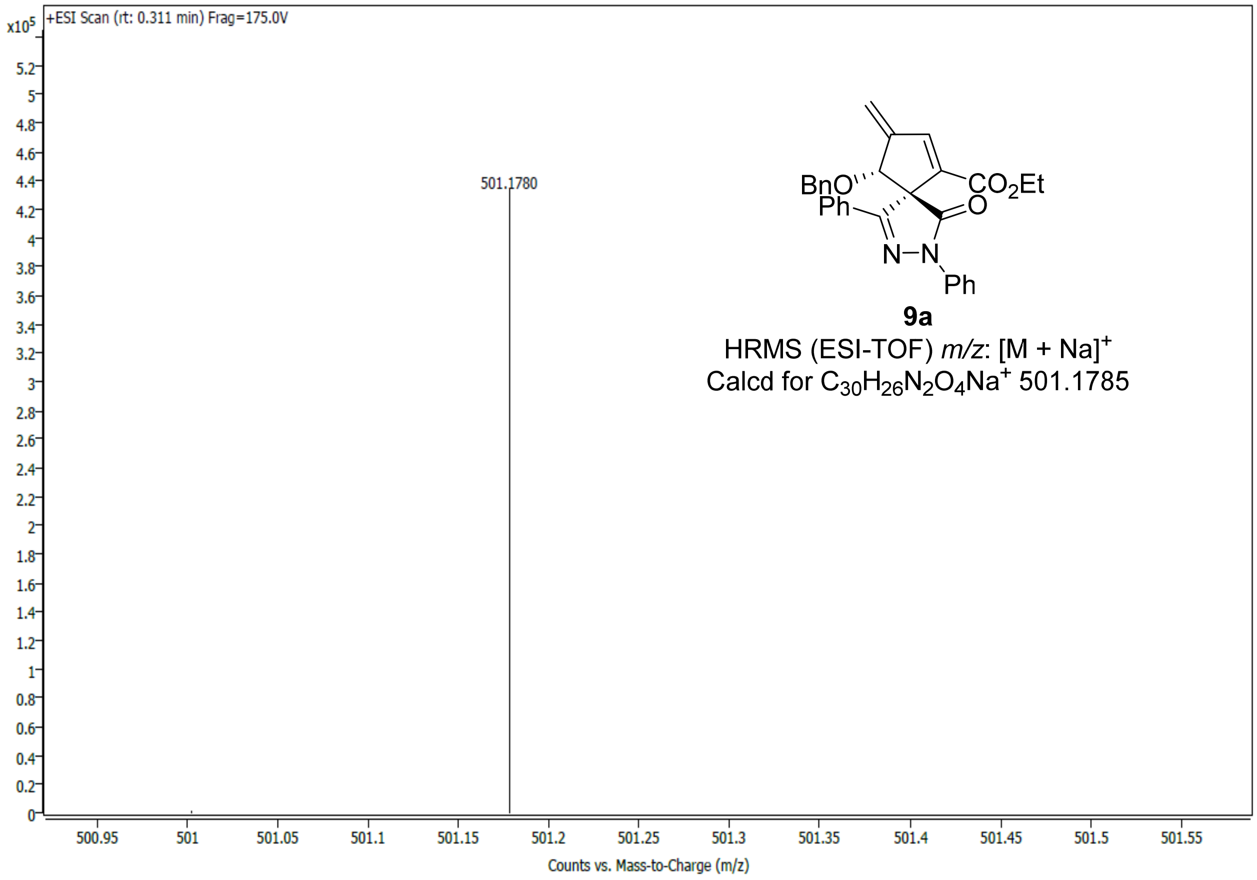
+ESI Scan (rt: 0.311 min) Frag=175.0V
x10^5
501.1780
9a
HRMS (ESI-TOF) m/z: [M + Na]+
Calcd for C30H26N2O4Na+ 501.1785
Counts vs. Mass-to-Charge (m/z)

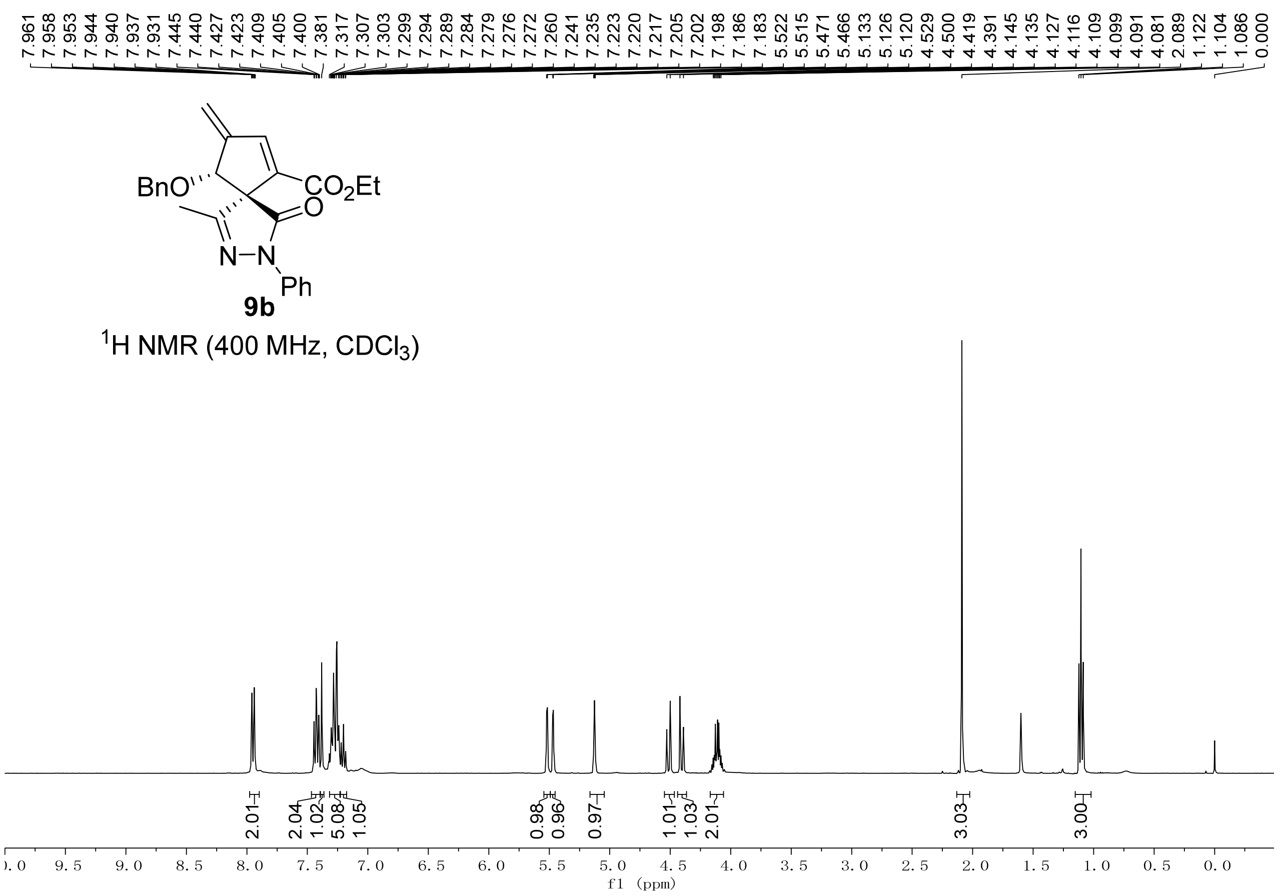
7.961 7.958 7.953 7.944 7.940 7.937 7.931 7.445 7.440 7.427 7.423 7.409 7.405 7.400 7.381 7.317 7.307 7.303 7.299 7.294 7.289 7.284 7.279 7.276 7.272 7.260 7.241 7.235 7.223 7.220 7.217 7.205 7.202 7.198 7.186 7.183 5.522 5.515 5.471 5.466 5.133 5.126 5.120 4.529 4.500 4.419 4.391 4.145 4.135 4.127 4.116 4.109 4.099 4.091 4.081 2.089 1.122 1.104 1.086 0.000
BnO
CO2Et
O
N−N
Ph
9b
1H NMR (400 MHz, CDCl3)
2.01 2.04 1.02 5.08 1.05 0.98 0.96 0.97 1.01 1.03 2.01 3.03 3.00
f1 (ppm)

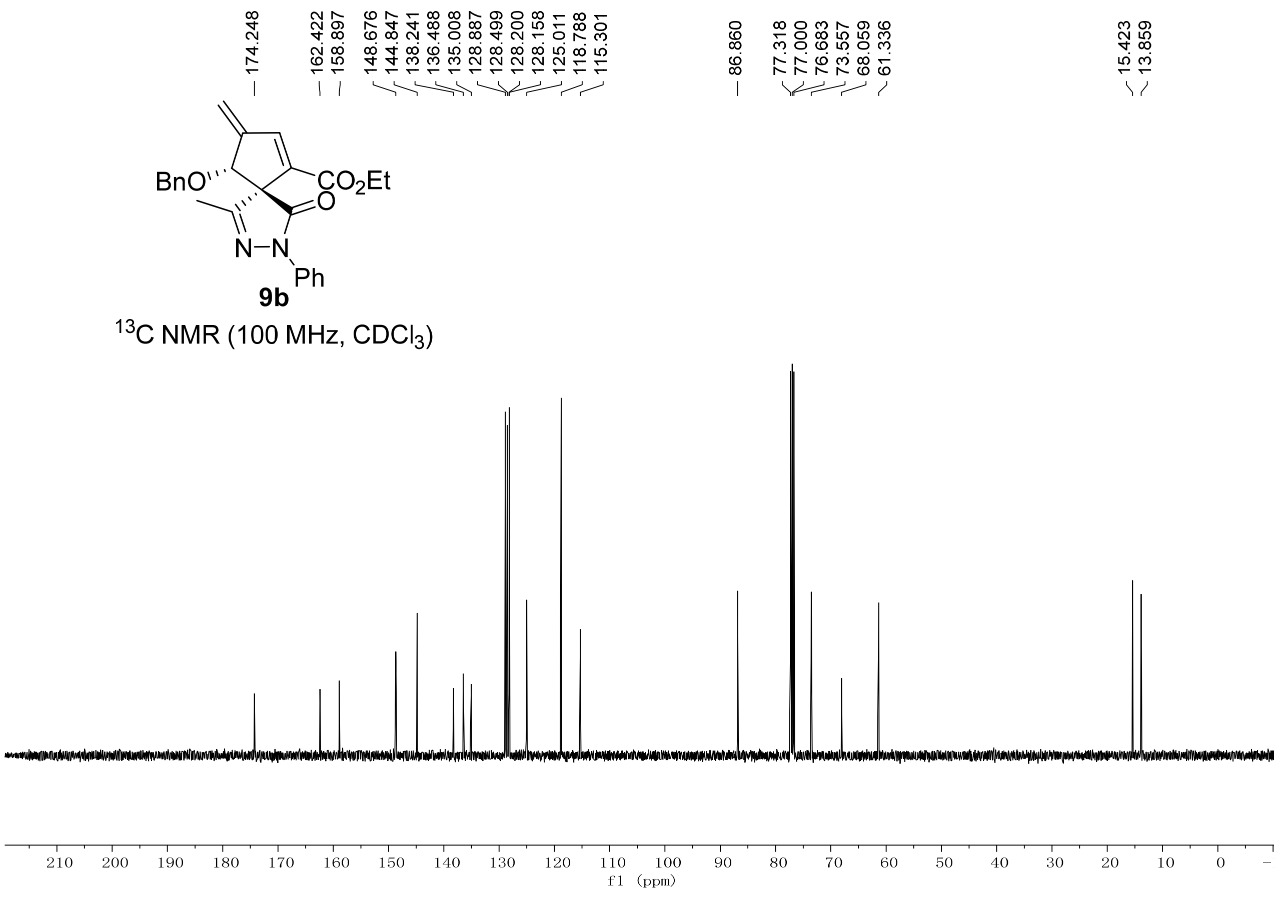
174.248 162.422 158.897 148.676 144.847 138.241 136.488 135.008 128.887 128.499 128.200 128.158 125.011 118.788 115.301 86.860 77.318 77.000 76.683 73.557 68.059 61.336 15.423 13.859
BnO
CO2Et
O
N−N
Ph
9b
13C NMR (100 MHz, CDCl3)
f1 (ppm)

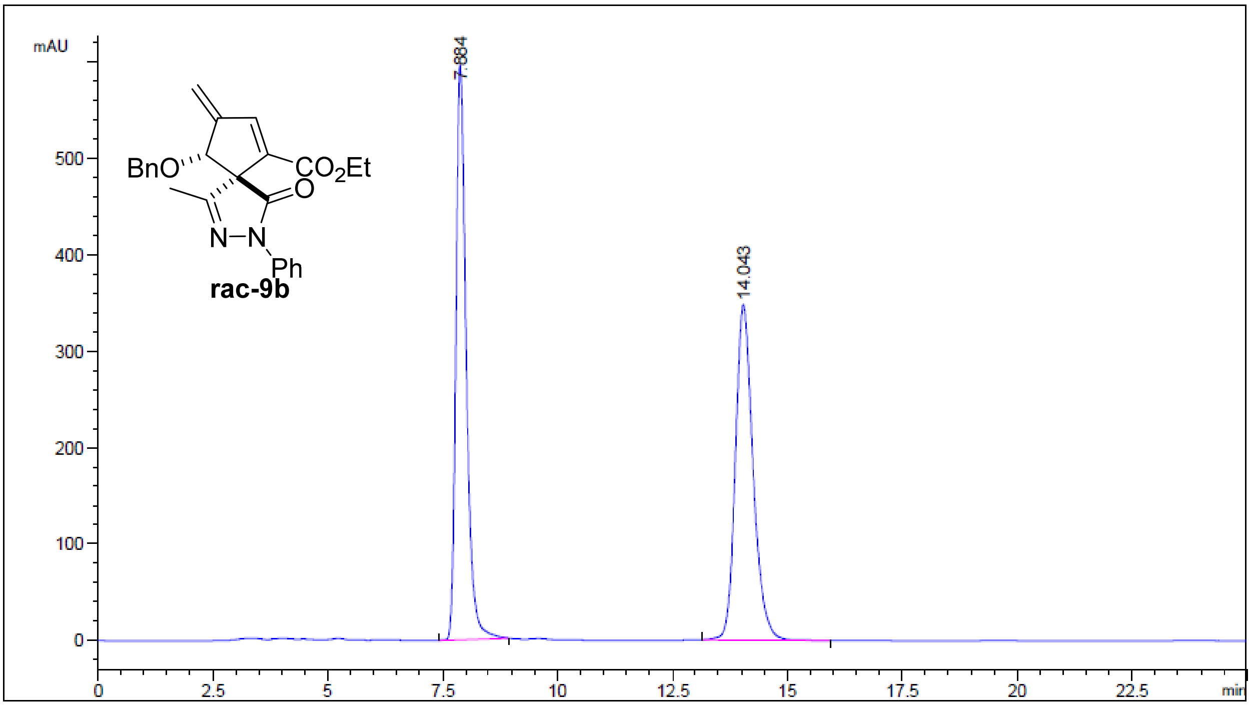

Peak #	RetTime [min]	Type	Width [min]	Area mAU *s	Height [mAU]	Area %
1	7.884	BBA	0.2369	9273.08691	595.97931	49.8770
2	14.043	BBA	0.4056	9318.80762	347.94821	50.1230

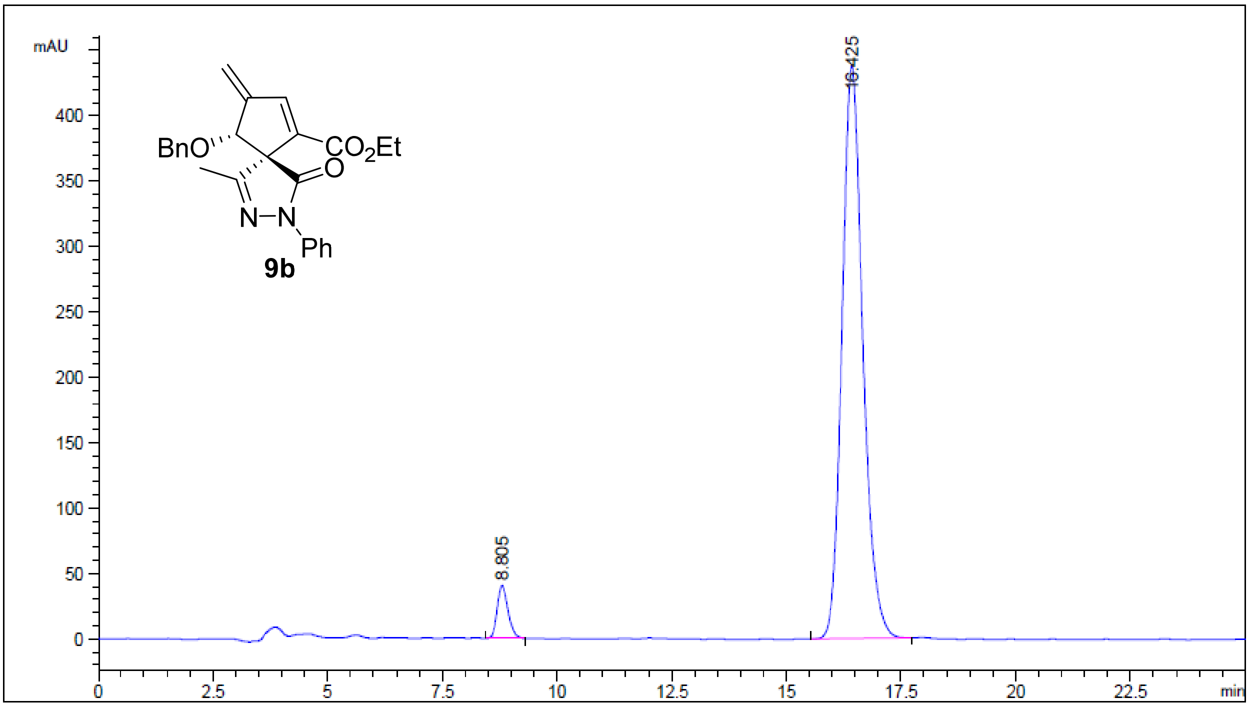

Peak #	RetTime [min]	Type	Width [min]	Area mAU *s	Height [mAU]	Area %
1	8.805	BB	0.2510	656.87189	40.35639	4.5465
2	16.425	BBA	0.4776	1.37911e4	438.42847	95.4535

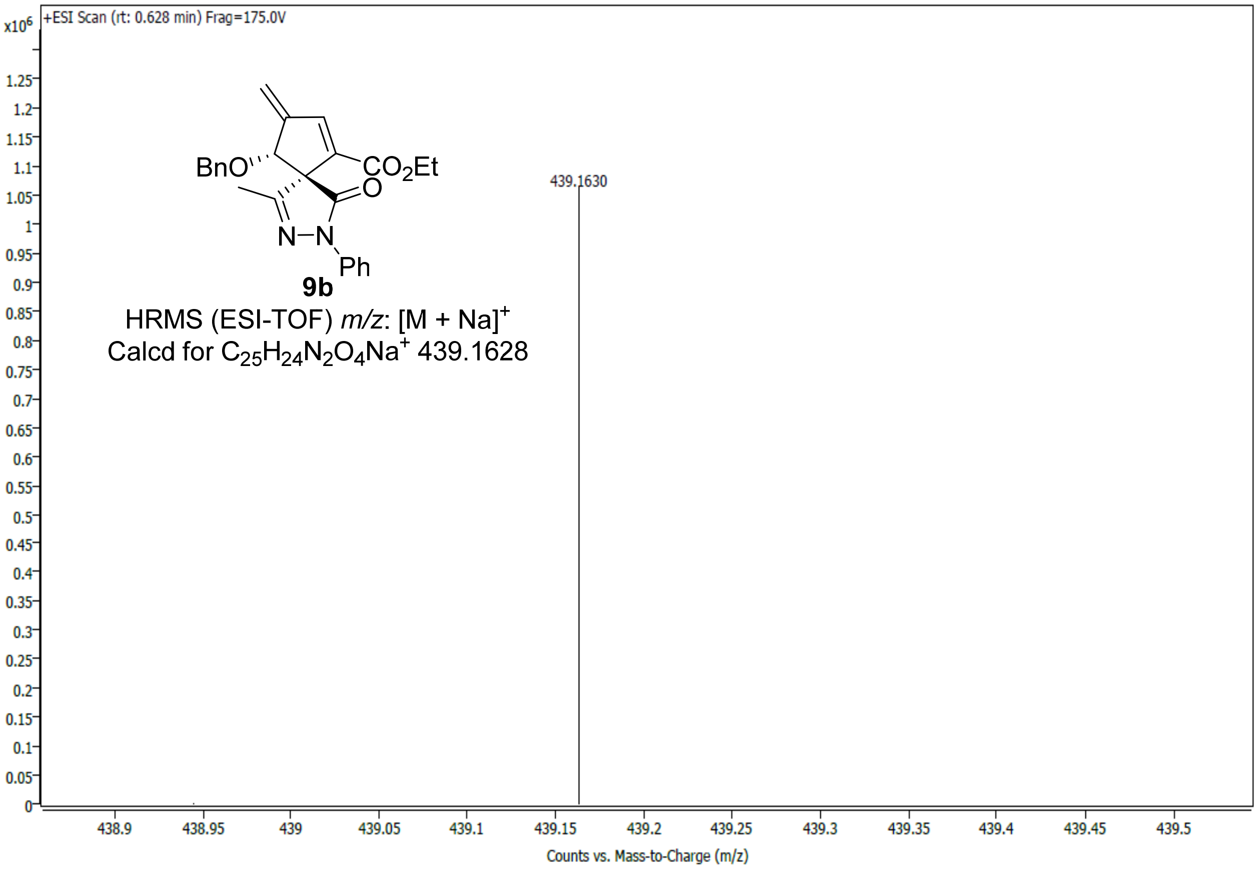
x10^6
+ESI Scan (rt: 0.628 min) Frag=175.0V
BnO
CO2Et
O
N–N
Ph
9b
HRMS (ESI-TOF) m/z: [M + Na]+
Calcd for C25H24N2O4Na+ 439.1628
439.1630
Counts vs. Mass-to-Charge (m/z)

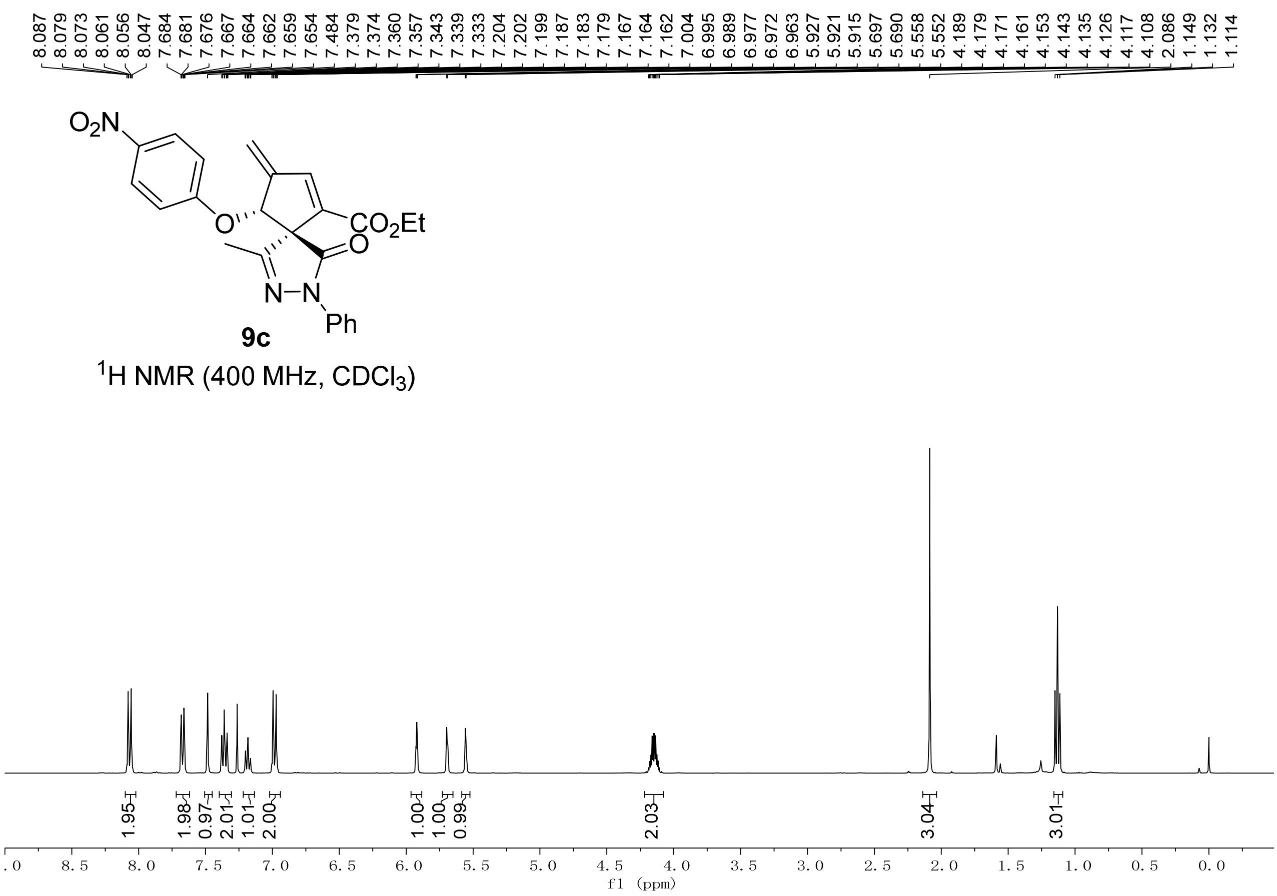

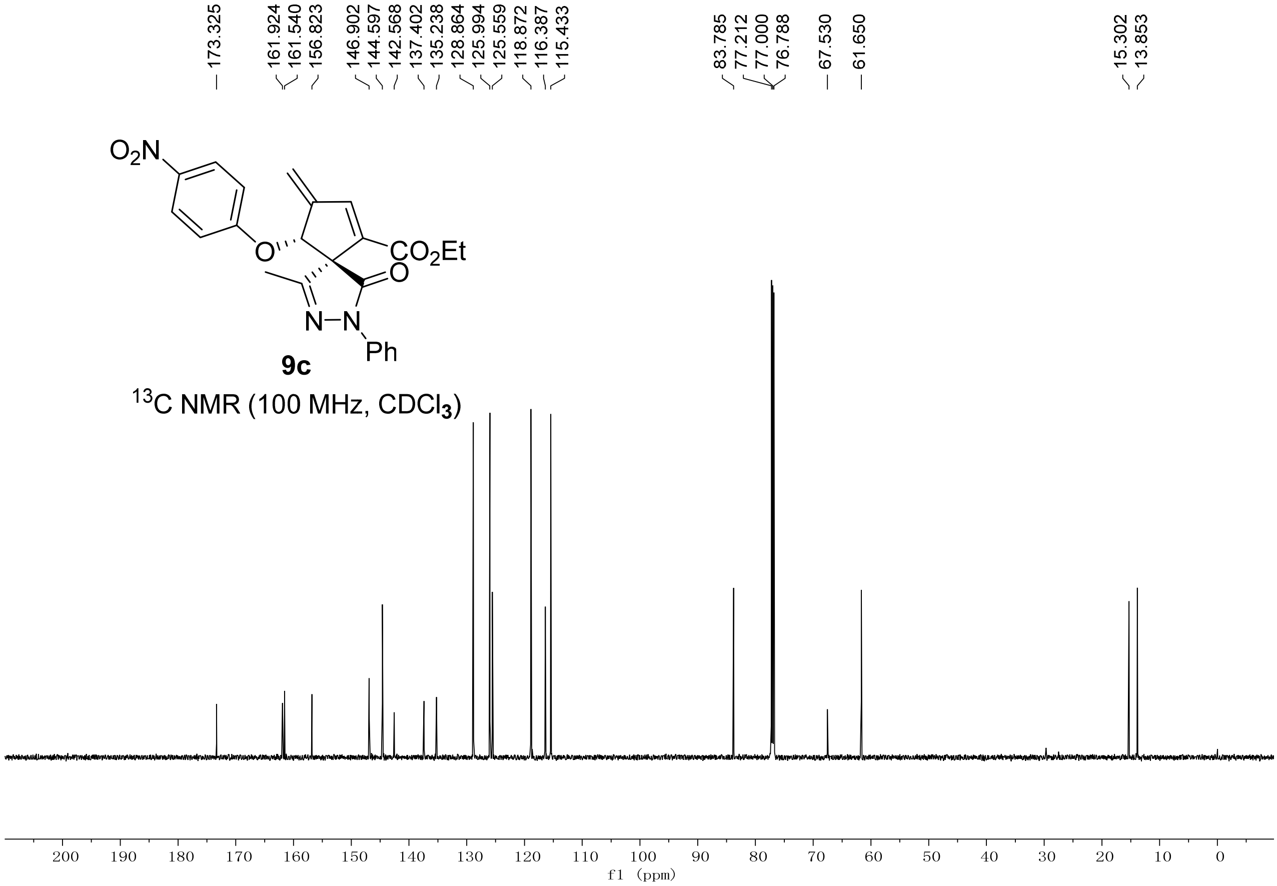

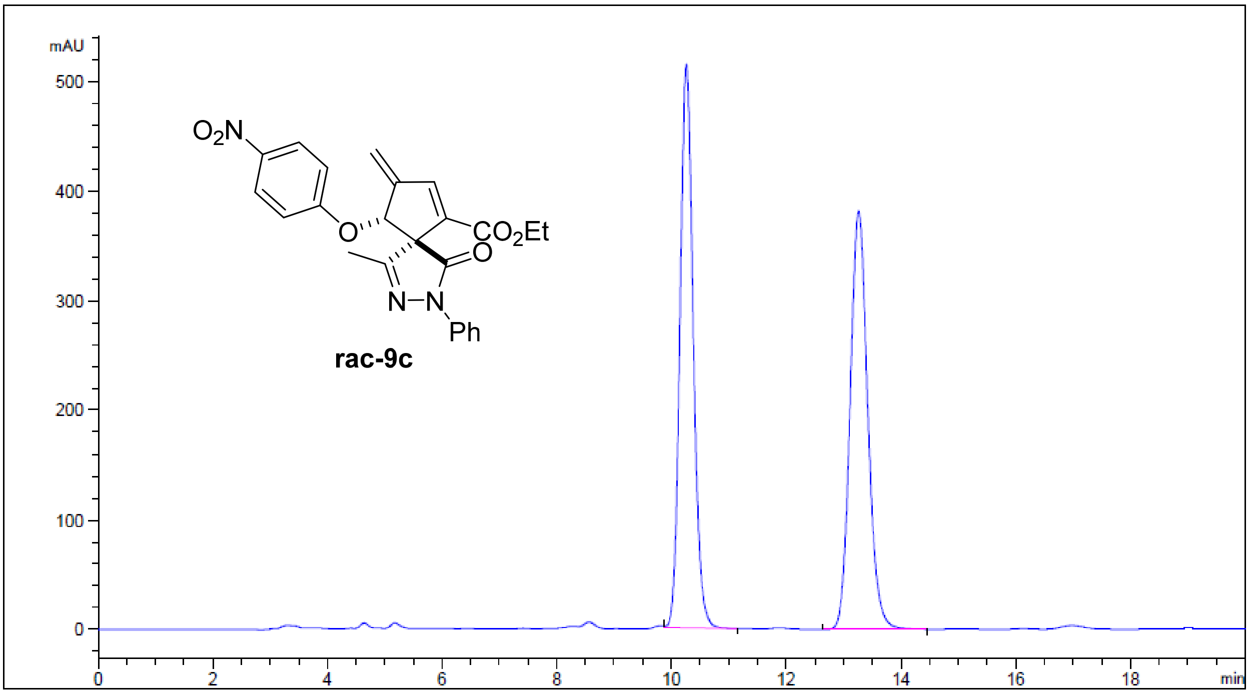

Peak #	RetTime [min]	Type	Width [min]	Area mAU *s	Area %
1	10.260	VB	0.2474	8256.88477	50.2067
2	13.262	VB	0.3313	8188.90137	49.7933

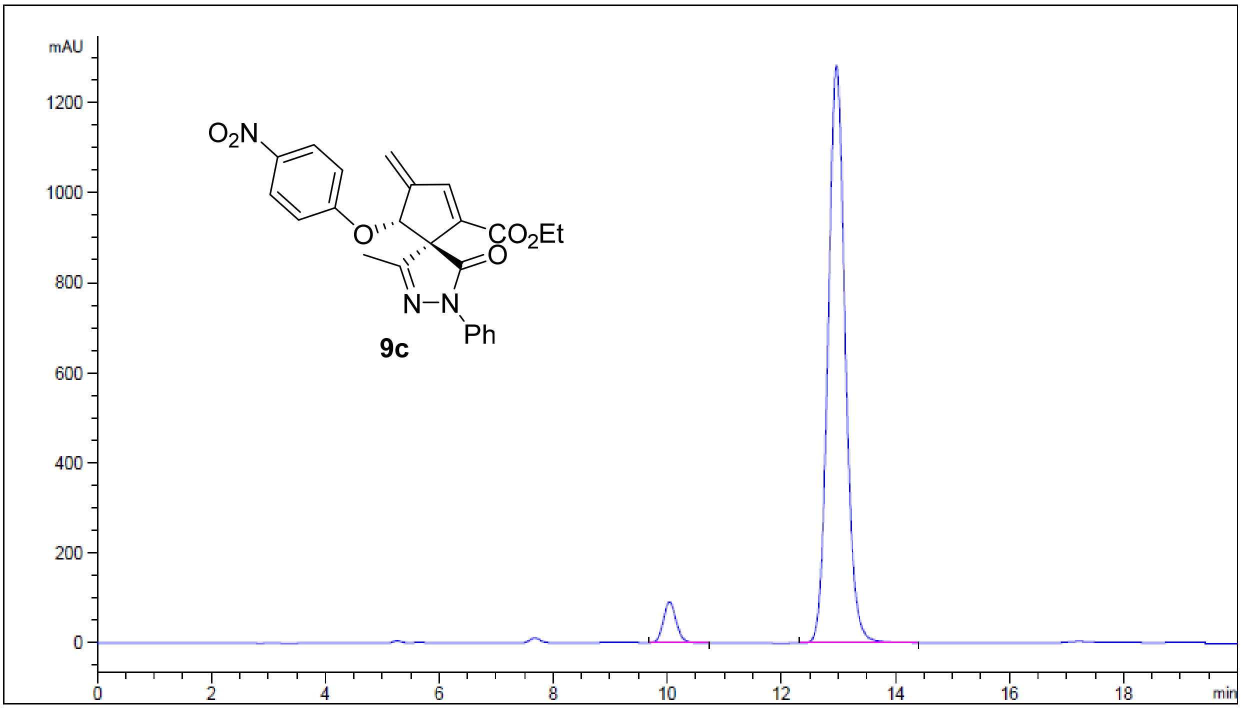

Peak #	RetTime [min]	Type	Width [min]	Area mAU *s	Area %
1	10.038	BB	0.2398	1427.34180	5.0204
2	12.968	BBA	0.3234	2.70037e4	94.9796

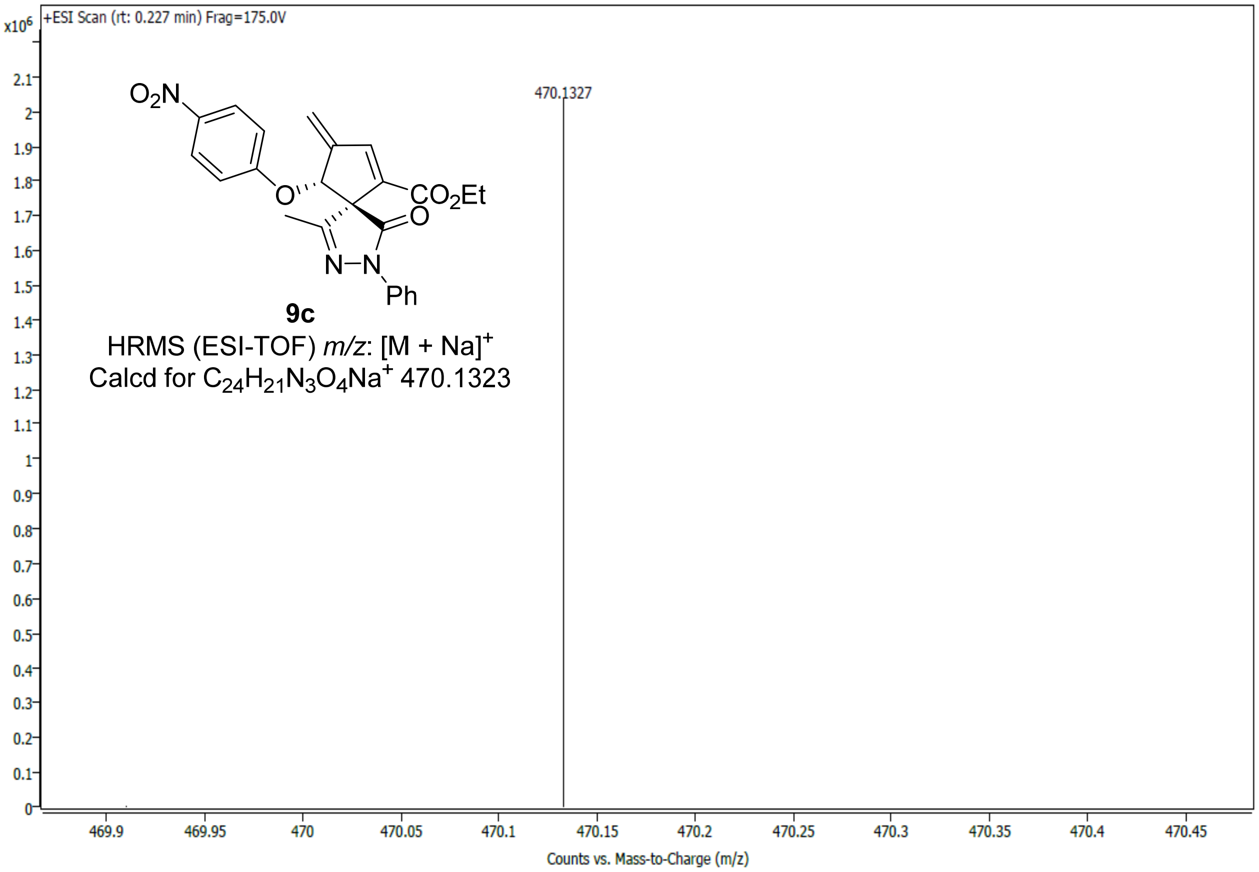
+ESI Scan (rt: 0.227 min) Frag=175.0V
x10^6
470.1327
O2N
CO2Et
O
N–N
Ph
9c
HRMS (ESI-TOF) m/z: [M + Na]+
Calcd for C24H21N3O4Na+ 470.1323
Counts vs. Mass-to-Charge (m/z)

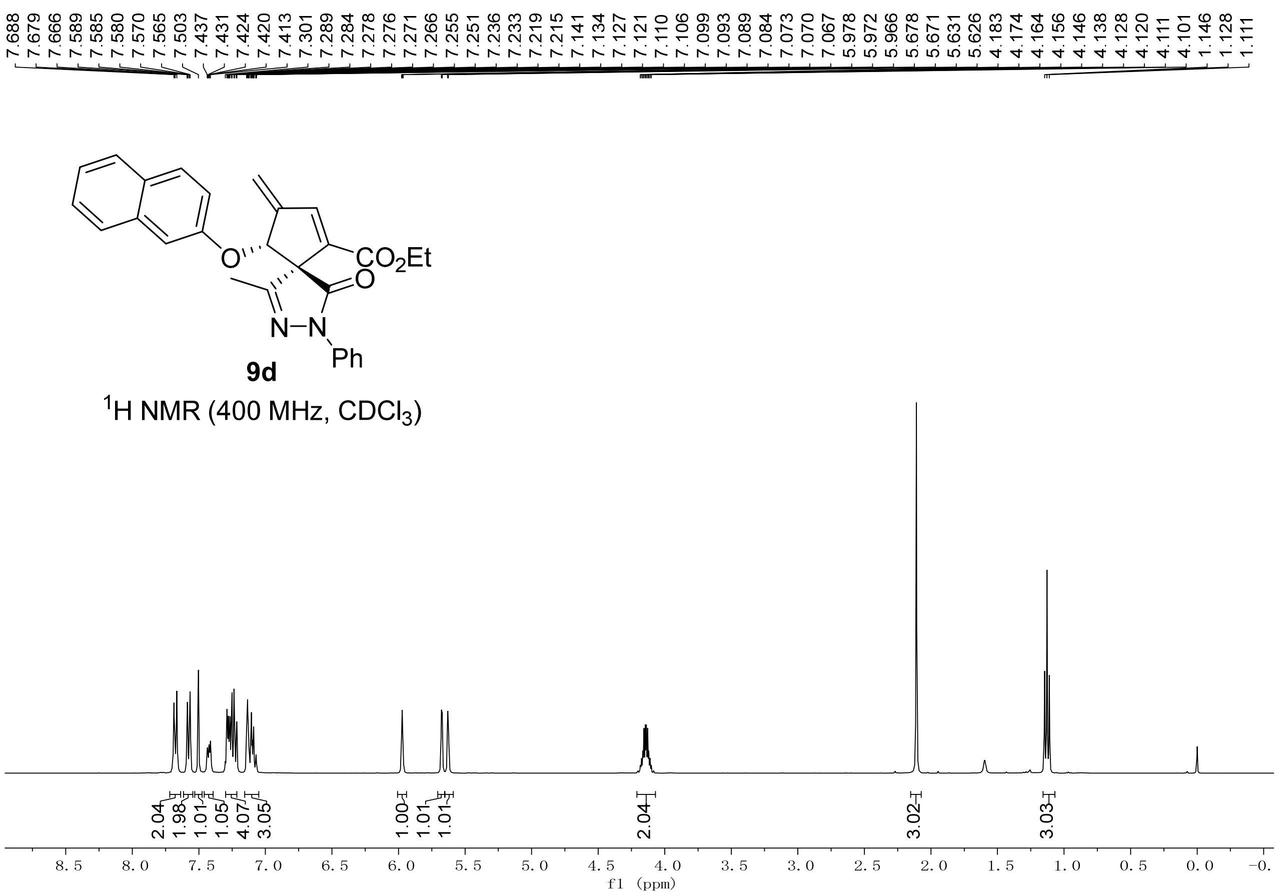
7.688 7.679 7.666 7.589 7.585 7.580 7.570 7.565 7.503 7.437 7.431 7.424 7.420 7.413 7.301 7.289 7.284 7.278 7.276 7.271 7.266 7.255 7.251 7.236 7.233 7.219 7.215 7.141 7.134 7.127 7.121 7.110 7.106 7.099 7.093 7.089 7.084 7.073 7.070 7.067 5.978 5.972 5.966 5.678 5.671 5.631 5.626 4.183 4.174 4.164 4.156 4.146 4.138 4.128 4.120 4.111 4.101 1.146 1.128 1.111
CO2Et
O
N–N
Ph
9d
1H NMR (400 MHz, CDCl3)
2.04 1.98 1.01 1.05 4.07 3.05 1.00 1.01 1.01 2.04 3.02 3.03
8.5 8.0 7.5 7.0 6.5 6.0 5.5 5.0 4.5 4.0 3.5 3.0 2.5 2.0 1.5 1.0 0.5 0.0 -0.
f1 (ppm)

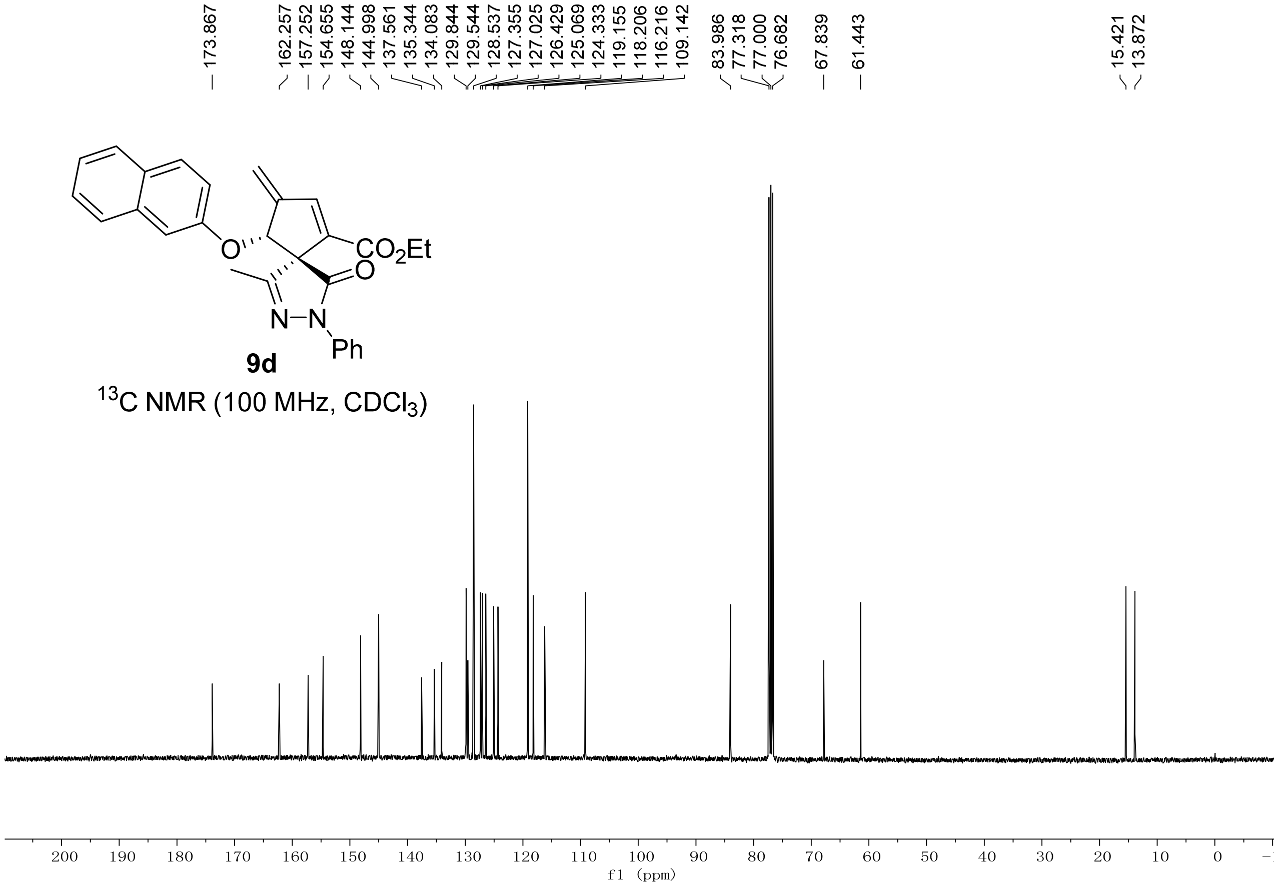
173.867 162.257 157.252 154.655 148.144 144.998 137.561 135.344 134.083 129.844 129.544 128.537 127.355 127.025 126.429 125.069 124.333 119.155 118.206 116.216 109.142 83.986 77.318 77.000 76.682 67.839 61.443 15.421 13.872
CO2Et
O
N–N
Ph
9d
13C NMR (100 MHz, CDCl3)
200 190 180 170 160 150 140 130 120 110 100 90 80 70 60 50 40 30 20 10 0
f1 (ppm)

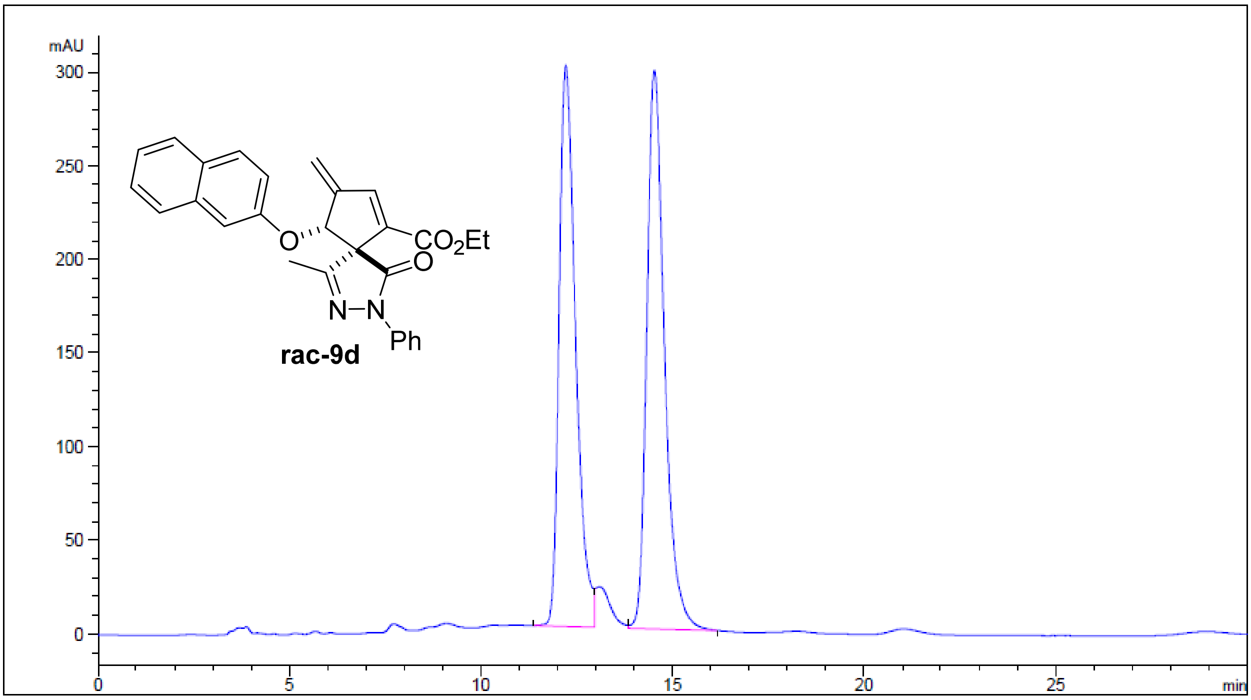

Peak #	RetTime [min]	Type	Width [min]	Area mAU *s	Area %
1	12.213	BV	0.4589	9000.74512	48.4905
2	14.527	VBA	0.4859	9561.12598	51.5095

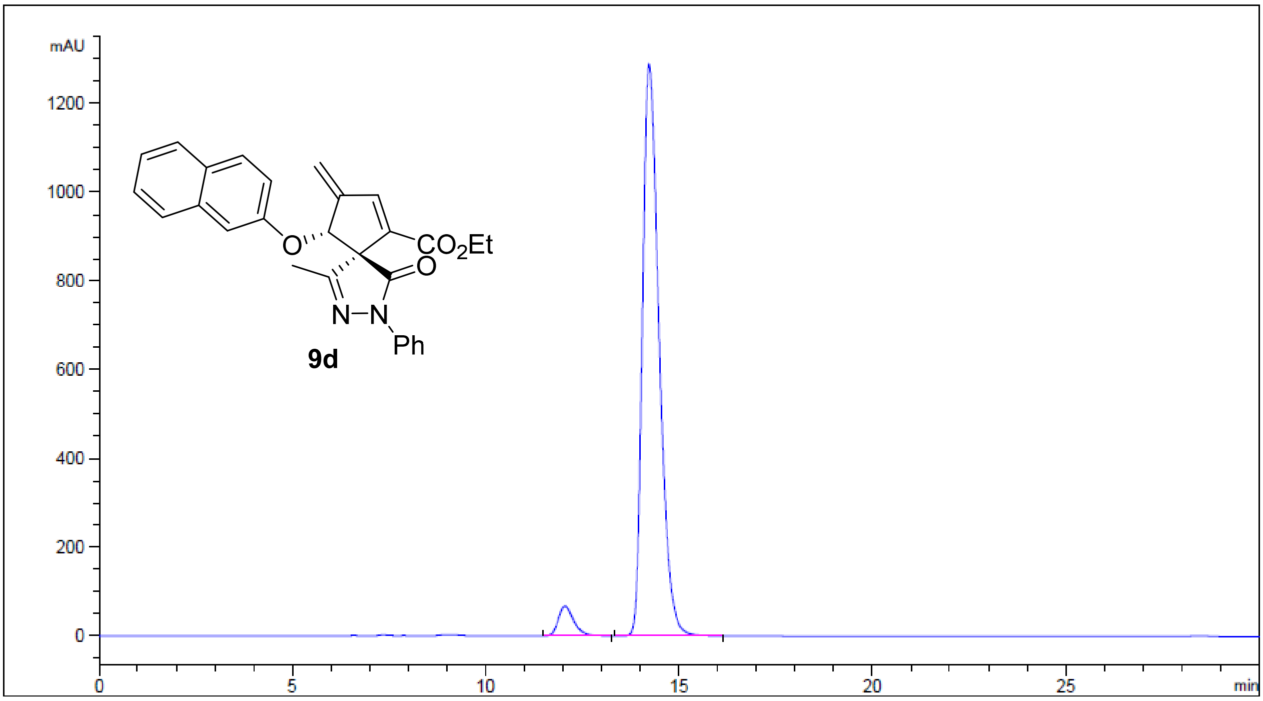

Peak #	RetTime [min]	Type	Width [min]	Area mAU *s	Area %
1	12.052	BB	0.4298	1898.24573	4.6887
2	14.231	BB	0.4625	3.85875e4	95.3113

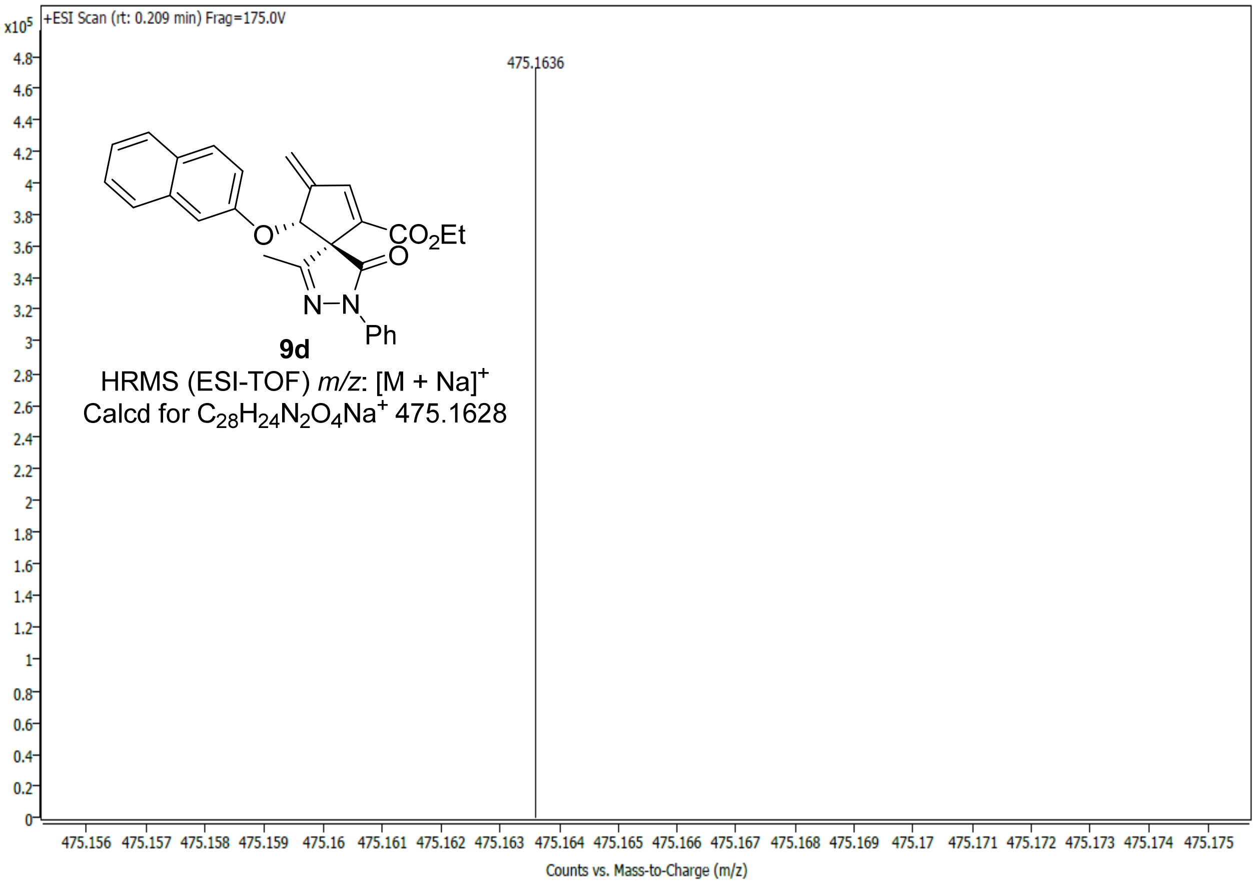
+ESI Scan (rt: 0.209 min) Frag=175.0V
x10^5
475.1636
9d
HRMS (ESI-TOF) m/z: [M + Na]+
Calcd for C28H24N2O4Na+ 475.1628
Counts vs. Mass-to-Charge (m/z)

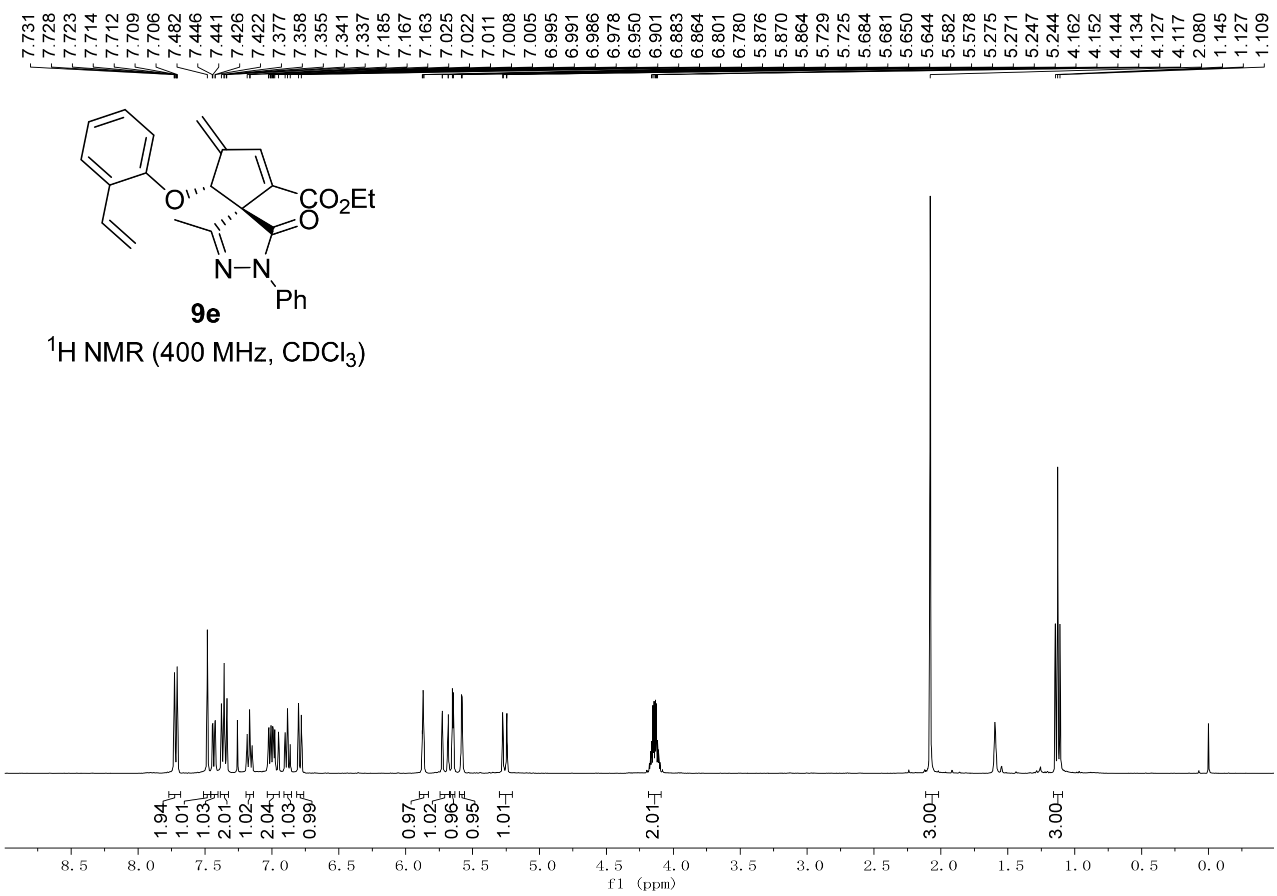
7.731
7.728
7.723
7.714
7.712
7.709
7.706
7.482
7.446
7.441
7.426
7.422
7.377
7.358
7.355
7.341
7.337
7.185
7.167
7.163
7.025
7.022
7.011
7.008
7.005
6.995
6.991
6.986
6.978
6.950
6.901
6.883
6.864
6.801
6.780
5.876
5.870
5.864
5.729
5.725
5.684
5.681
5.650
5.644
5.582
5.578
5.275
5.271
5.247
5.244
4.162
4.152
4.144
4.134
4.127
4.117
2.080
1.145
1.127
1.109
O
CO2Et
O
N–N
Ph
9e
1H NMR (400 MHz, CDCl3)
1.94
1.01
1.03
2.01
1.02
2.04
1.03
0.99
0.97
1.02
0.96
0.95
1.01
2.01
3.00
3.00
8.5
8.0
7.5
7.0
6.5
6.0
5.5
5.0
4.5
4.0
3.5
3.0
2.5
2.0
1.5
1.0
0.5
0.0
f1 (ppm)

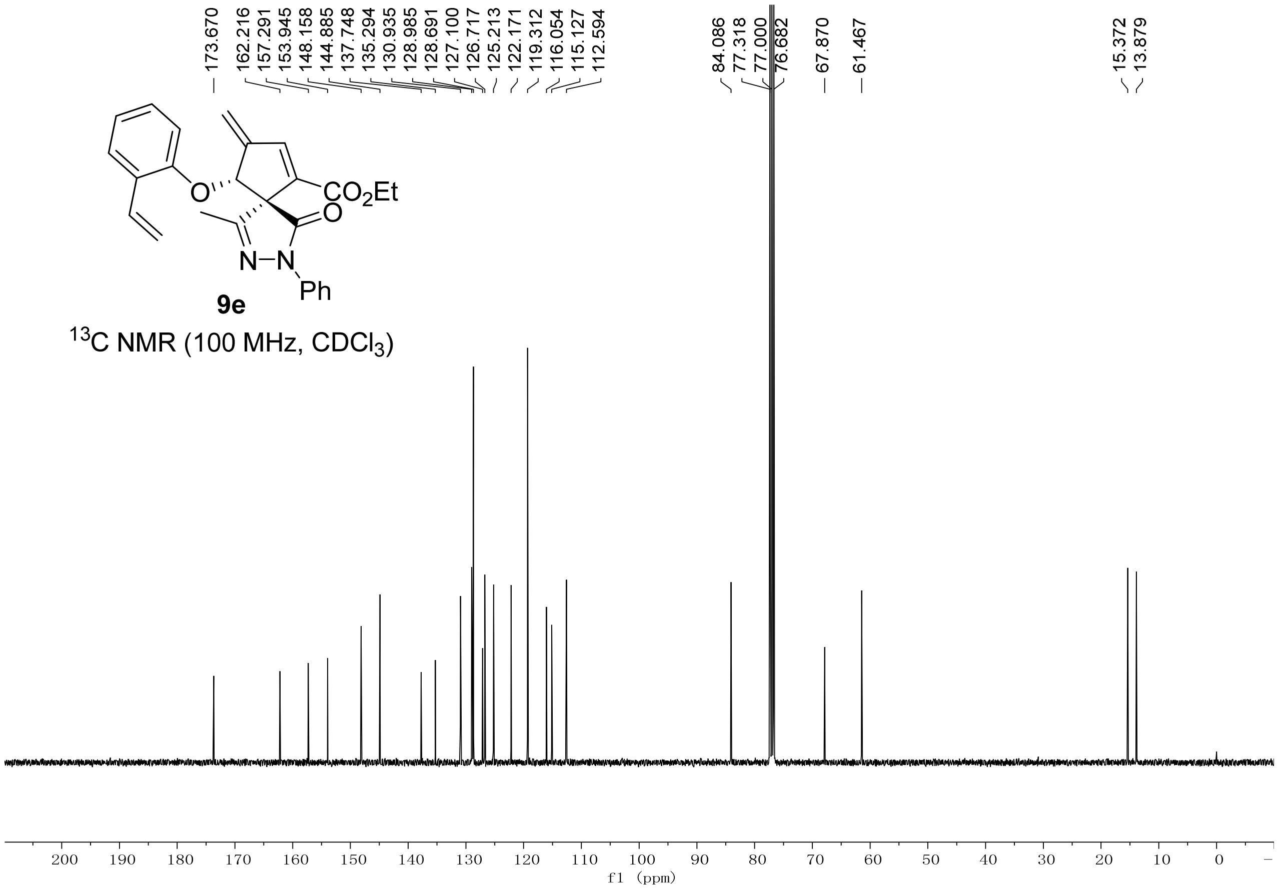
173.670
162.216
157.291
153.945
148.158
144.885
137.748
135.294
130.935
128.985
128.691
127.100
126.717
125.213
122.171
119.312
116.054
115.127
112.594
84.086
77.318
77.000
76.682
67.870
61.467
15.372
13.879
O
CO2Et
O
N–N
Ph
9e
13C NMR (100 MHz, CDCl3)
200
190
180
170
160
150
140
130
120
110
100
90
80
70
60
50
40
30
20
10
0
f1 (ppm)

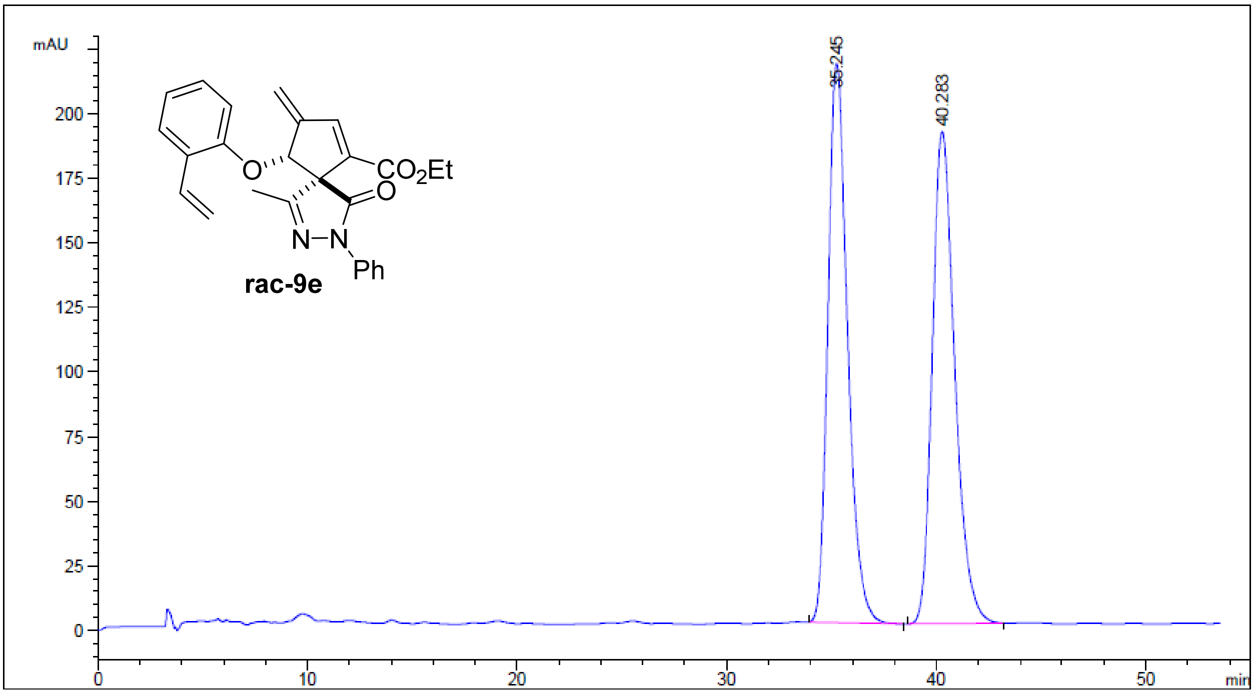

Peak #	RetTime [min]	Type	Width [min]	Area mAU *s	Height [mAU]	Area %
1	35.245	BB	0.9958	1.40469e4	216.48453	49.7985
2	40.283	BBA	1.1357	1.41606e4	190.38248	50.2015

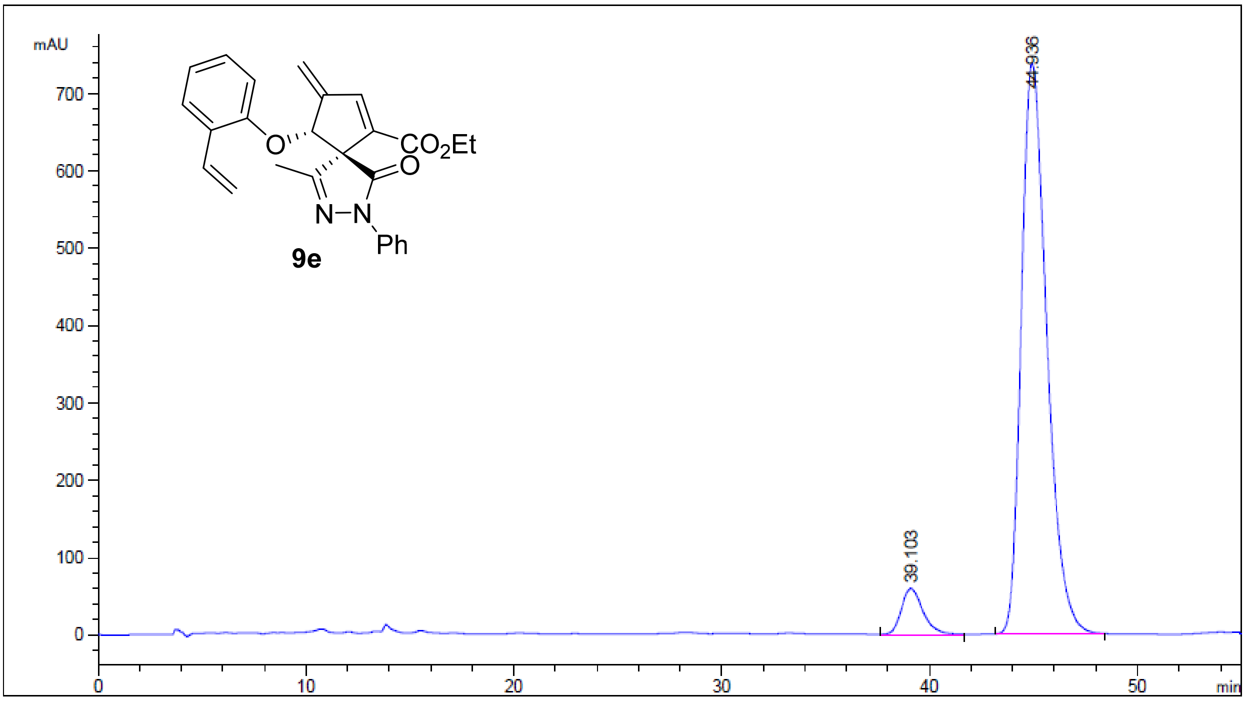

Peak #	RetTime [min]	Type	Width [min]	Area mAU *s	Height [mAU]	Area %
1	39.103	BBA	1.0909	4275.92480	59.76605	6.3520
2	44.936	BBA	1.3116	6.30403e4	737.59875	93.6480

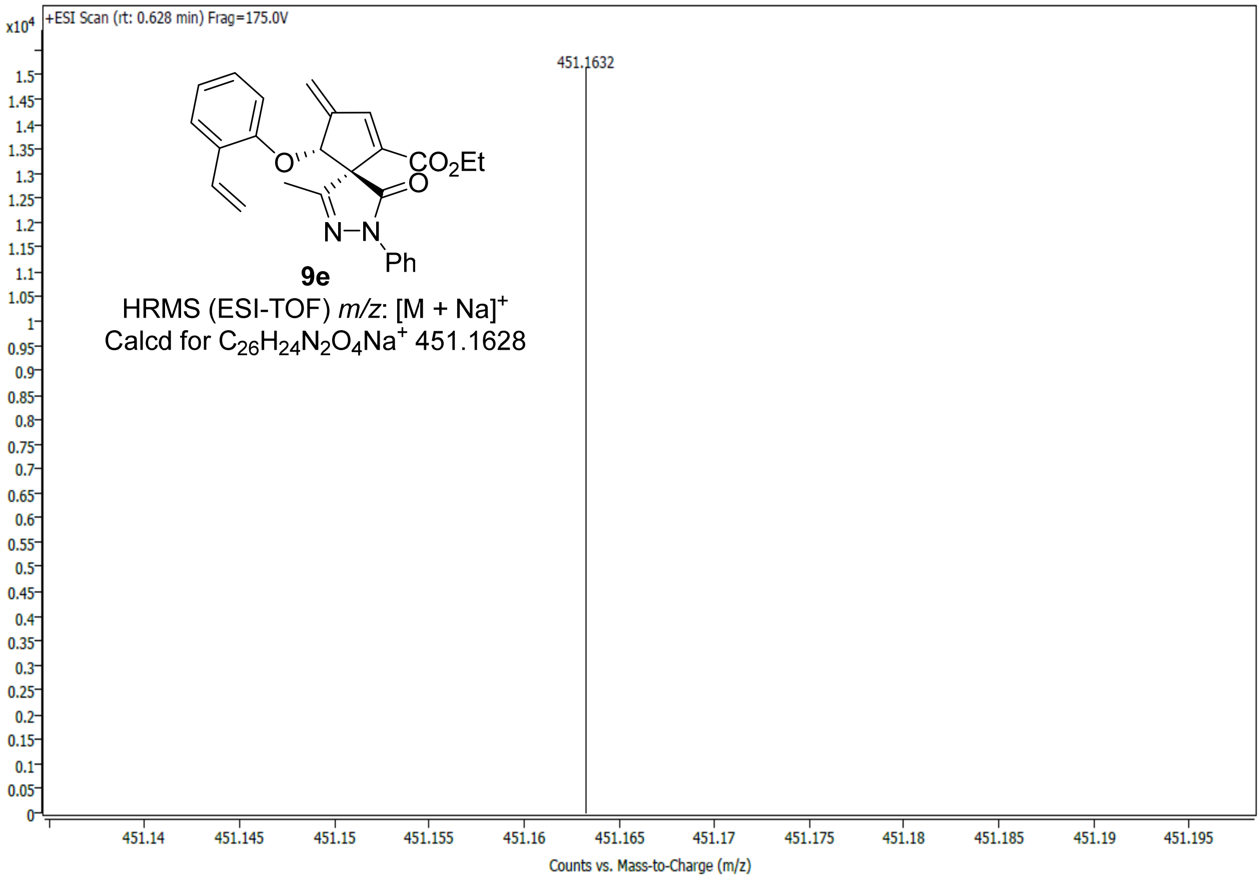
+ESI Scan (rt: 0.628 min) Frag=175.0V
x10^4
451.1632
9e
HRMS (ESI-TOF) m/z: [M + Na]+
Calcd for C26H24N2O4Na+ 451.1628
CO2Et
O
N–N
Ph
Counts vs. Mass-to-Charge (m/z)

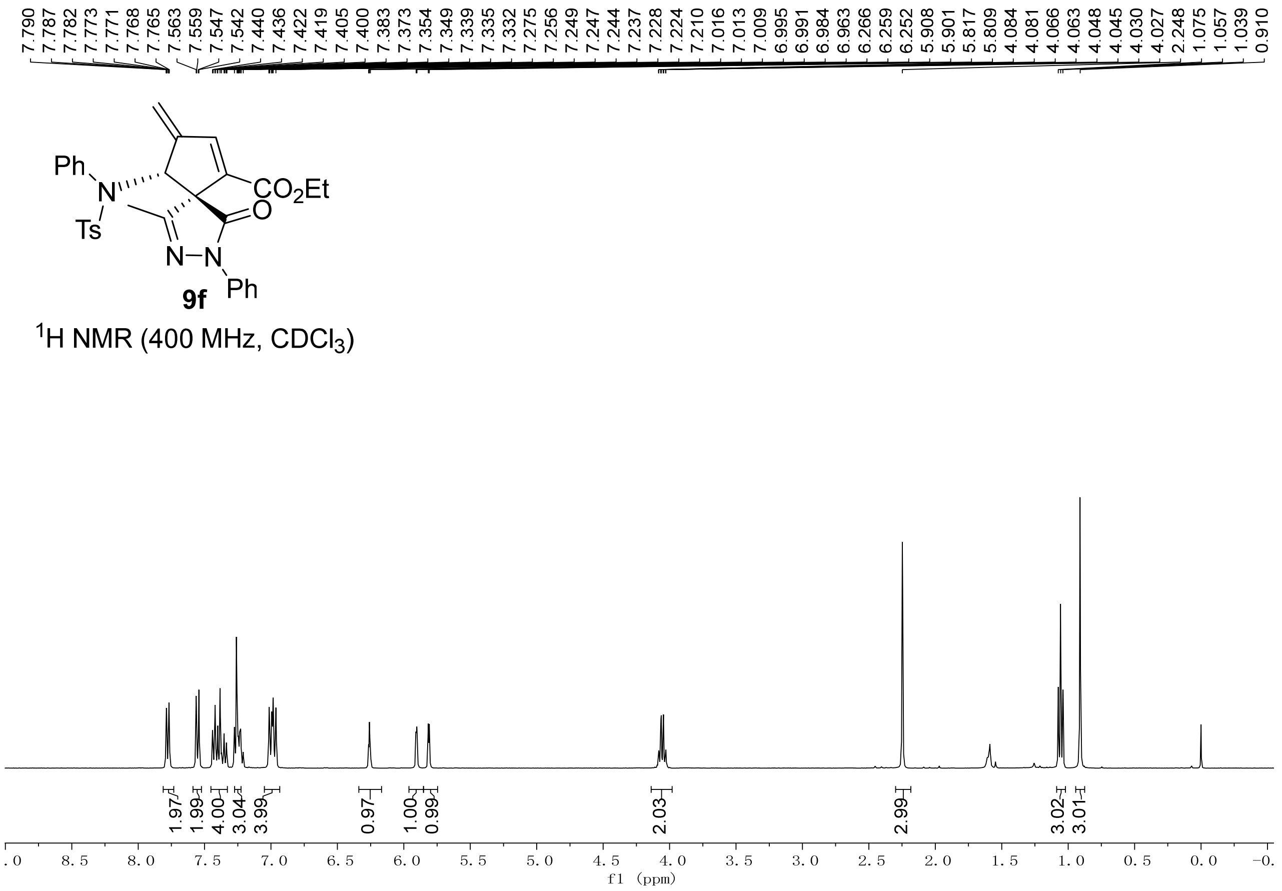
7.790 7.787 7.782 7.773 7.771 7.768 7.765 7.563 7.559 7.547 7.542 7.440 7.436 7.422 7.419 7.405 7.400 7.383 7.373 7.354 7.349 7.339 7.335 7.332 7.275 7.256 7.249 7.247 7.244 7.237 7.228 7.224 7.210 7.016 7.013 7.009 6.995 6.991 6.984 6.963 6.266 6.259 6.252 5.908 5.901 5.817 5.809 4.084 4.081 4.066 4.063 4.048 4.045 4.030 4.027 2.248 1.075 1.057 1.039 0.910
Ph
N
Ts
CO2Et
O
N–N
Ph
9f
1H NMR (400 MHz, CDCl3)
1.97 1.99 4.00 3.04 3.99 0.97 1.00 0.99 2.03 2.99 3.02 3.01
f1 (ppm)

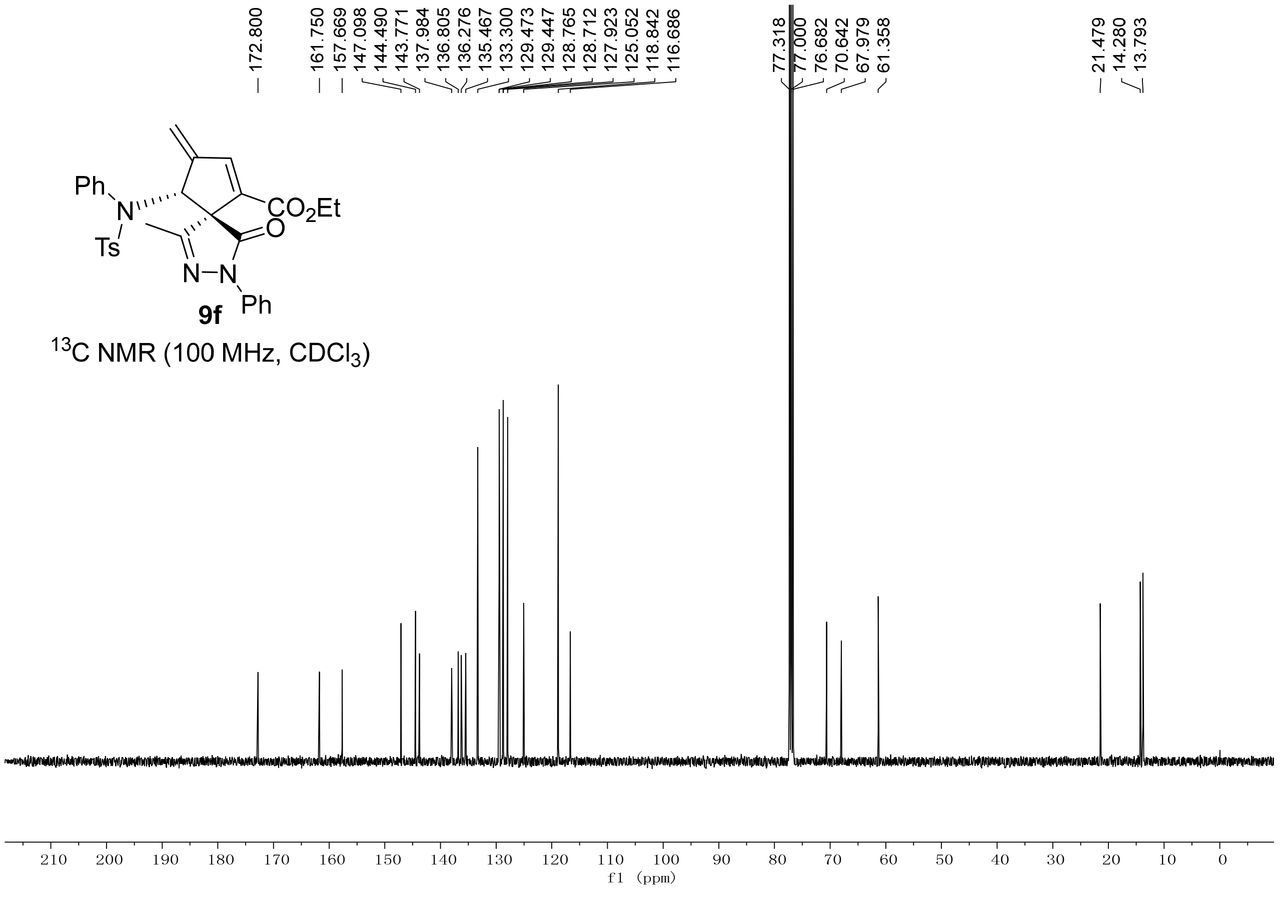
172.800 161.750 157.669 147.098 144.490 143.771 137.984 136.805 136.276 135.467 133.300 129.473 129.447 128.765 128.712 127.923 125.052 118.842 116.686 77.318 77.000 76.682 70.642 67.979 61.358 21.479 14.280 13.793
Ph
N
Ts
CO2Et
O
N–N
Ph
9f
13C NMR (100 MHz, CDCl3)
f1 (ppm)

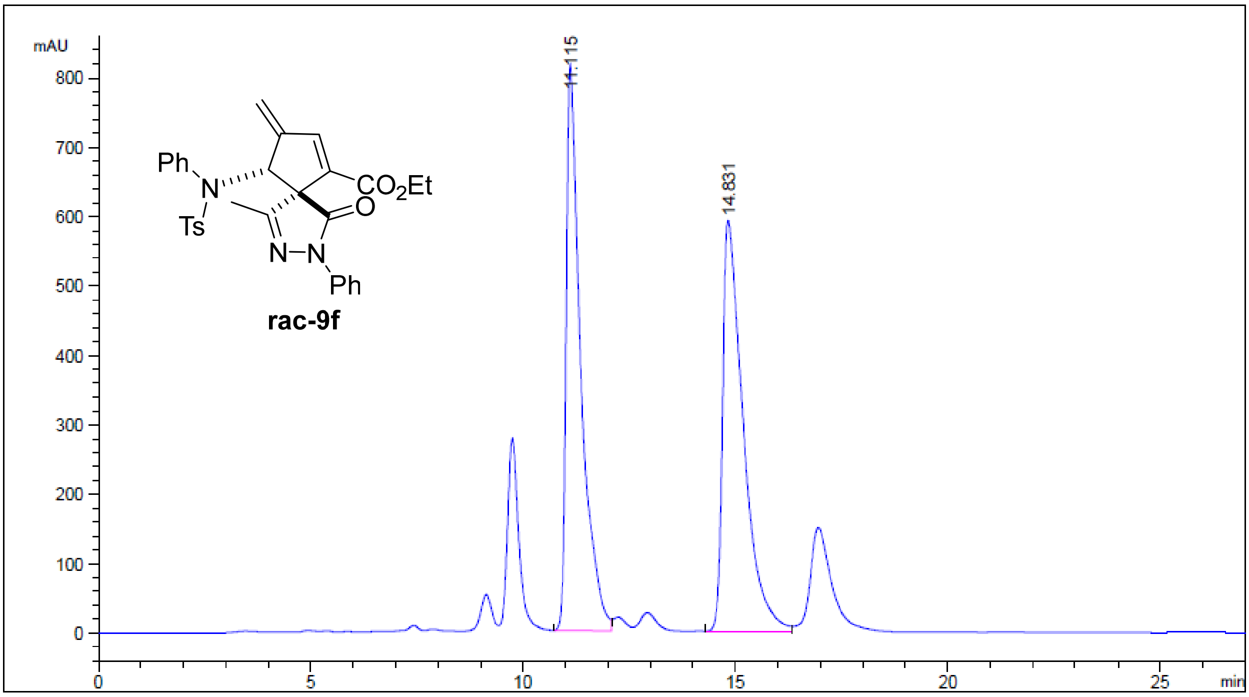

Peak #	RetTime [min]	Type	Width [min]	Area mAU *s	Height [mAU]	Area %
1	11.115	BV	0.3462	1.96222e4	815.72772	49.3888
2	14.831	VV	0.5256	2.01079e4	592.32727	50.6112

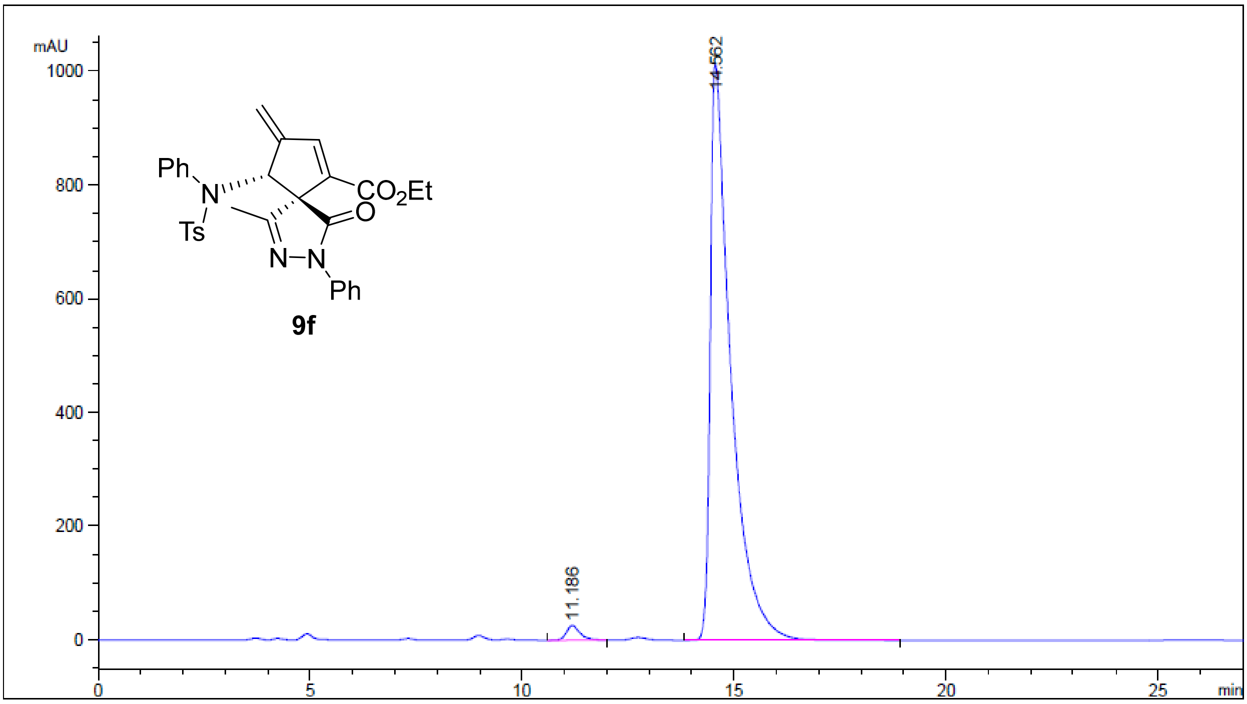

Peak #	RetTime [min]	Type	Width [min]	Area mAU *s	Height [mAU]	Area %
1	11.186	BB	0.3276	562.70184	25.61827	1.6211
2	14.562	BB	0.4781	3.41493e4	1012.15070	98.3789

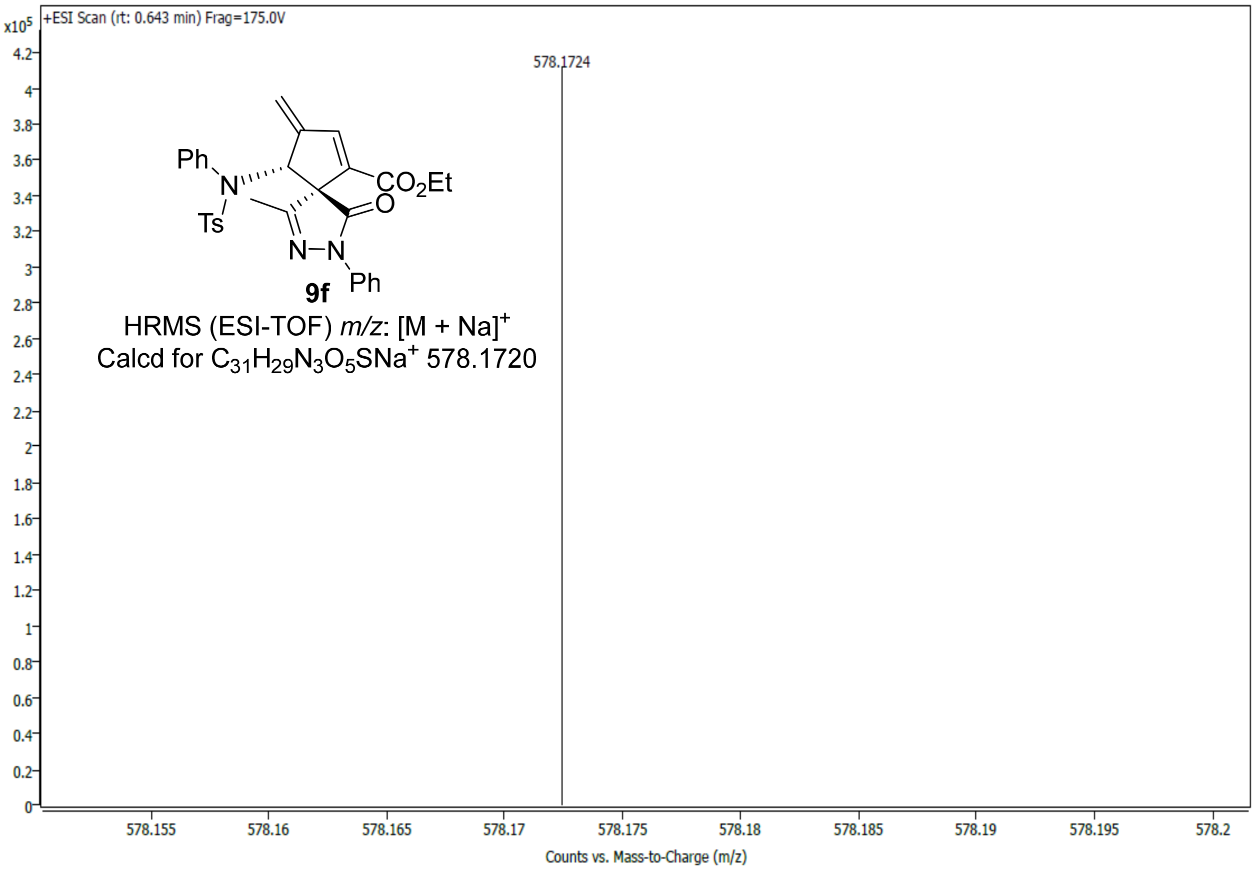

x10^5
+ESI Scan (rt: 0.643 min) Frag=175.0V
578.1724
Ph
N
Ts
CO2Et
O
N–N
Ph
9f
HRMS (ESI-TOF) m/z: [M + Na]+
Calcd for C31H29N3O5SNa+ 578.1720
4.2
4
3.8
3.6
3.4
3.2
3
2.8
2.6
2.4
2.2
2
1.8
1.6
1.4
1.2
1
0.8
0.6
0.4
0.2
0
578.155
578.16
578.165
578.17
578.175
578.18
578.185
578.19
578.195
578.2
Counts vs. Mass-to-Charge (m/z)

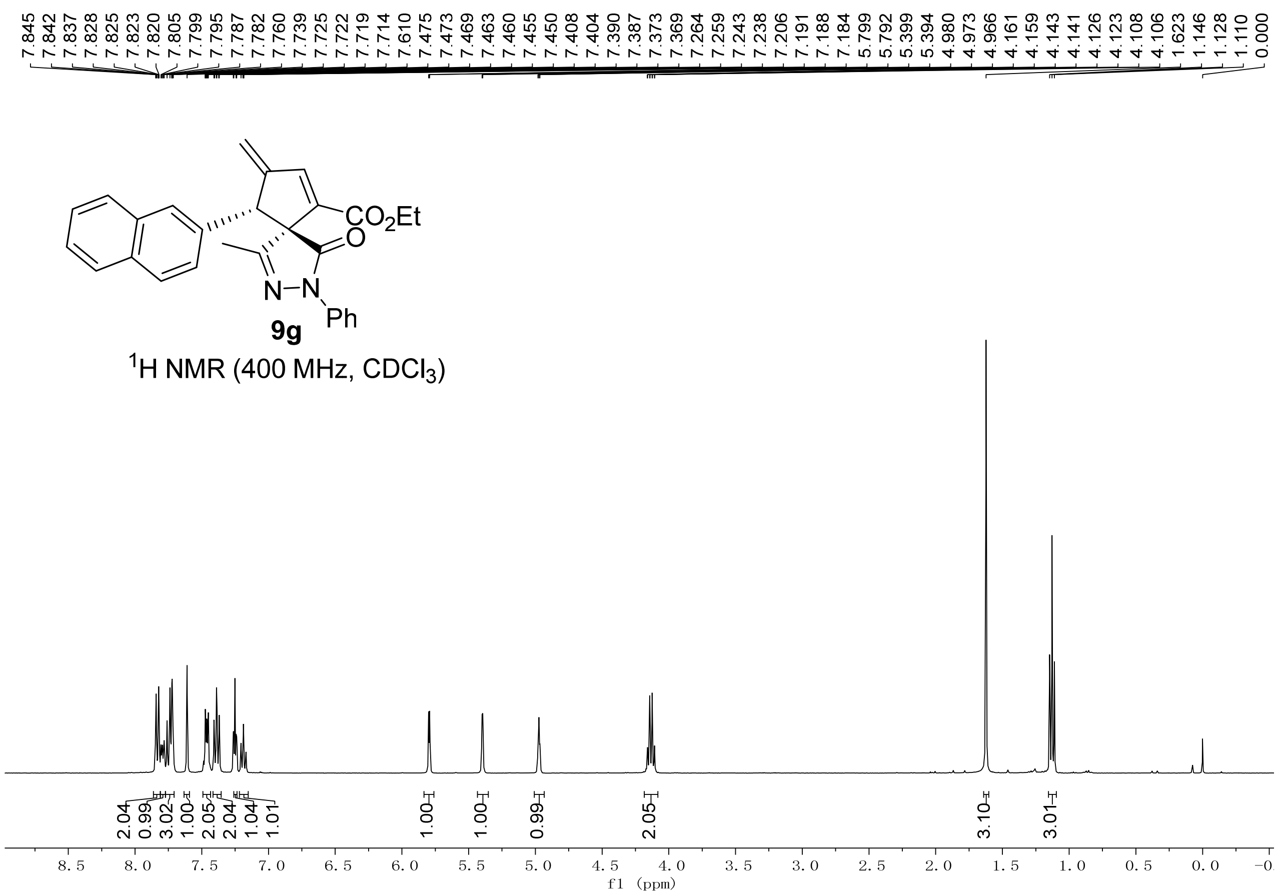
7.845 7.842 7.837 7.828 7.825 7.823 7.820 7.805 7.799 7.795 7.787 7.782 7.760 7.739 7.725 7.722 7.719 7.714 7.610 7.475 7.473 7.469 7.463 7.460 7.455 7.450 7.408 7.404 7.390 7.387 7.373 7.369 7.264 7.259 7.243 7.238 7.206 7.191 7.188 7.184 5.799 5.792 5.399 5.394 4.980 4.973 4.966 4.161 4.159 4.143 4.141 4.126 4.123 4.108 4.106 1.623 1.146 1.128 1.110 0.000
CO2Et
O
N–N
Ph
9g
1H NMR (400 MHz, CDCl3)
2.04 0.99 3.02 1.00 2.05 2.04 1.04 1.01 1.00 1.00 0.99 2.05 3.10 3.01
f1 (ppm)

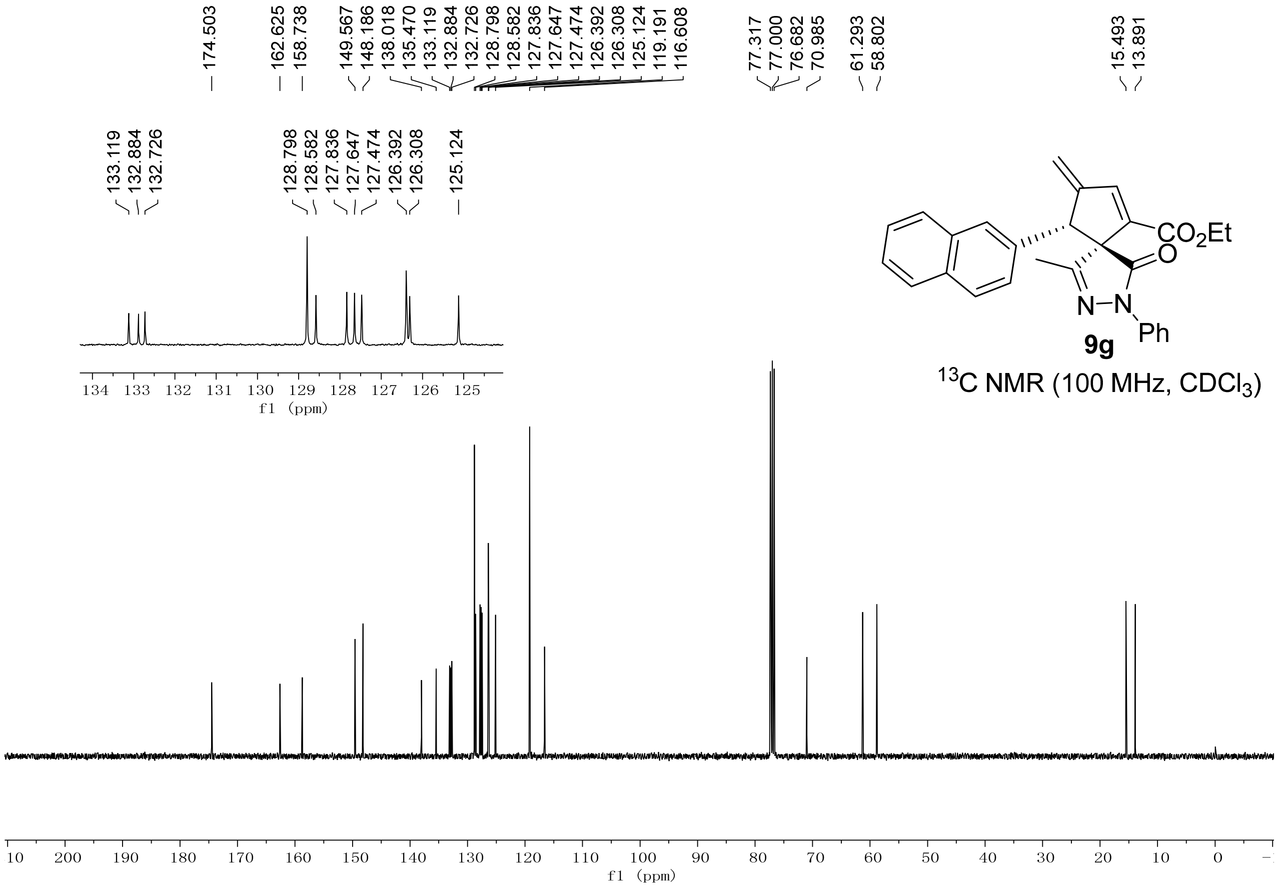
174.503 162.625 158.738 149.567 148.186 138.018 135.470 133.119 132.884 132.726 128.798 128.582 127.836 127.647 127.474 126.392 126.308 125.124 119.191 116.608 77.317 77.000 76.682 70.985 61.293 58.802 15.493 13.891
133.119 132.884 132.726 128.798 128.582 127.836 127.647 127.474 126.392 126.308 125.124
f1 (ppm)
CO2Et
O
N–N
Ph
9g
13C NMR (100 MHz, CDCl3)
f1 (ppm)

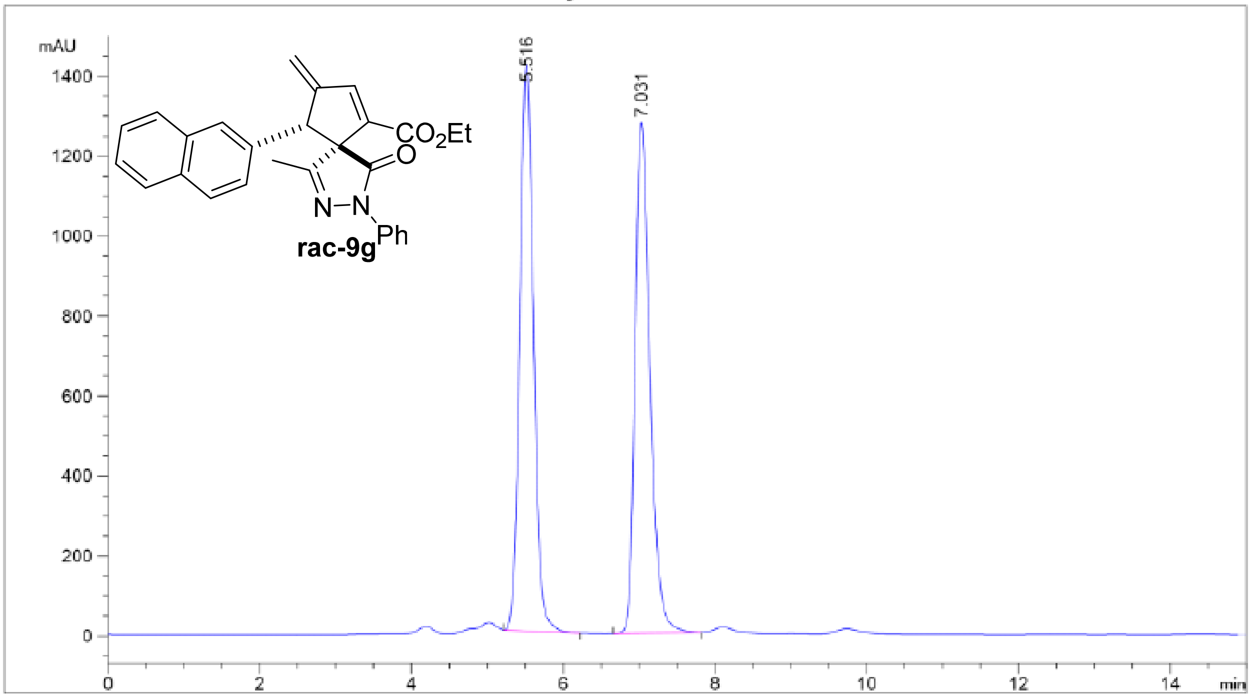

Peak #	RetTime [min]	Type	Width [min]	Area mAU *s	Height [mAU]	Area %
1	5.516	BB	0.1939	1.77066e4	1416.78748	50.4079
2	7.031	BB	0.2100	1.74200e4	1277.60022	49.5921

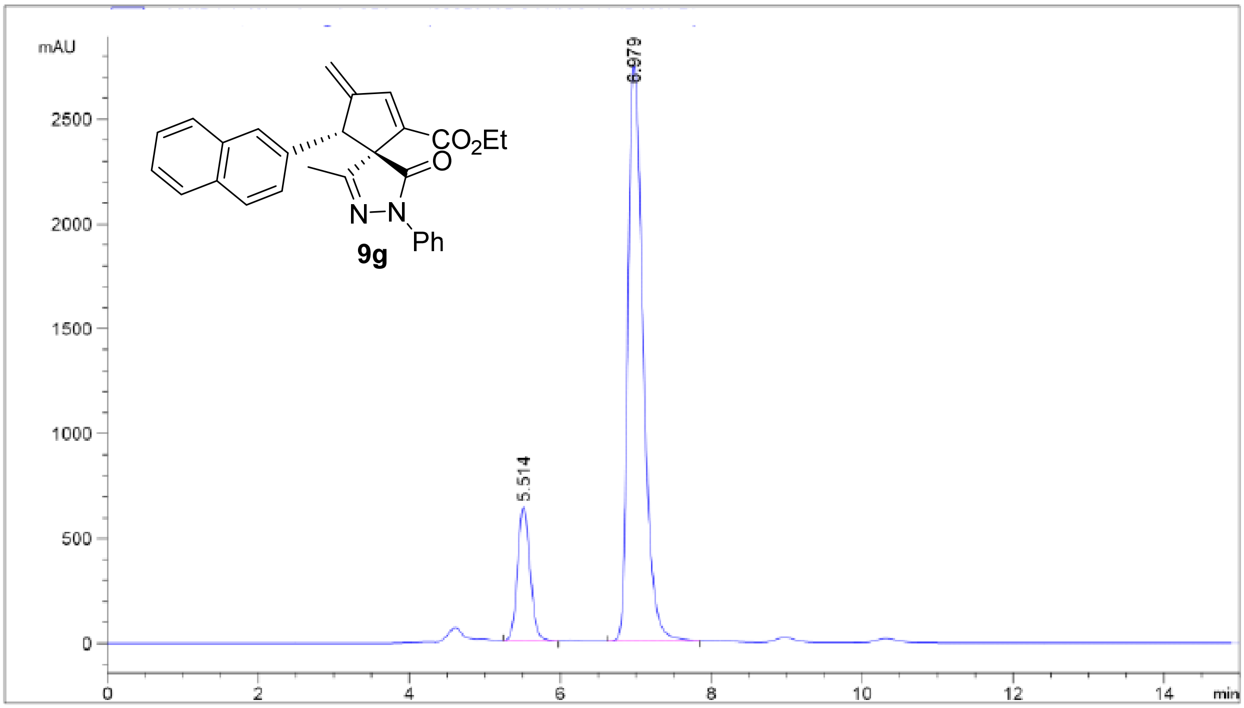

Peak #	RetTime [min]	Type	Width [min]	Area mAU *s	Height [mAU]	Area %
1	5.514	BV	0.1794	7435.77783	639.08752	16.2743
2	6.979	BBA	0.2136	3.82546e4	2744.51904	83.7257

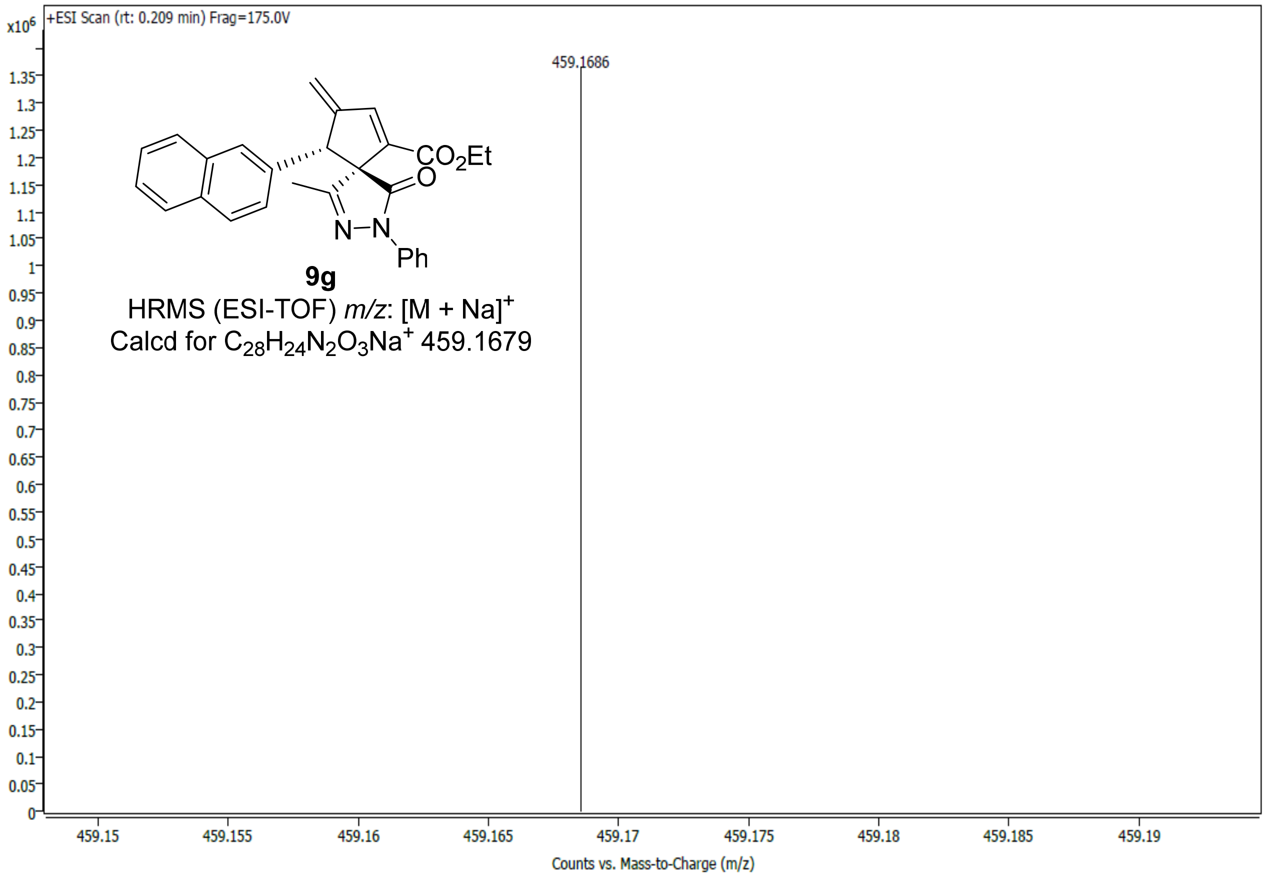

x10^6
+ESI Scan (rt: 0.209 min) Frag=175.0V
459.1686
CO2Et
O
N
N
Ph
9g
HRMS (ESI-TOF) m/z: [M + Na]+
Calcd for C28H24N2O3Na+ 459.1679
1.35
1.3
1.25
1.2
1.15
1.1
1.05
1
0.95
0.9
0.85
0.8
0.75
0.7
0.65
0.6
0.55
0.5
0.45
0.4
0.35
0.3
0.25
0.2
0.15
0.1
0.05
0
459.15
459.155
459.16
459.165
459.17
459.175
459.18
459.185
459.19
Counts vs. Mass-to-Charge (m/z)

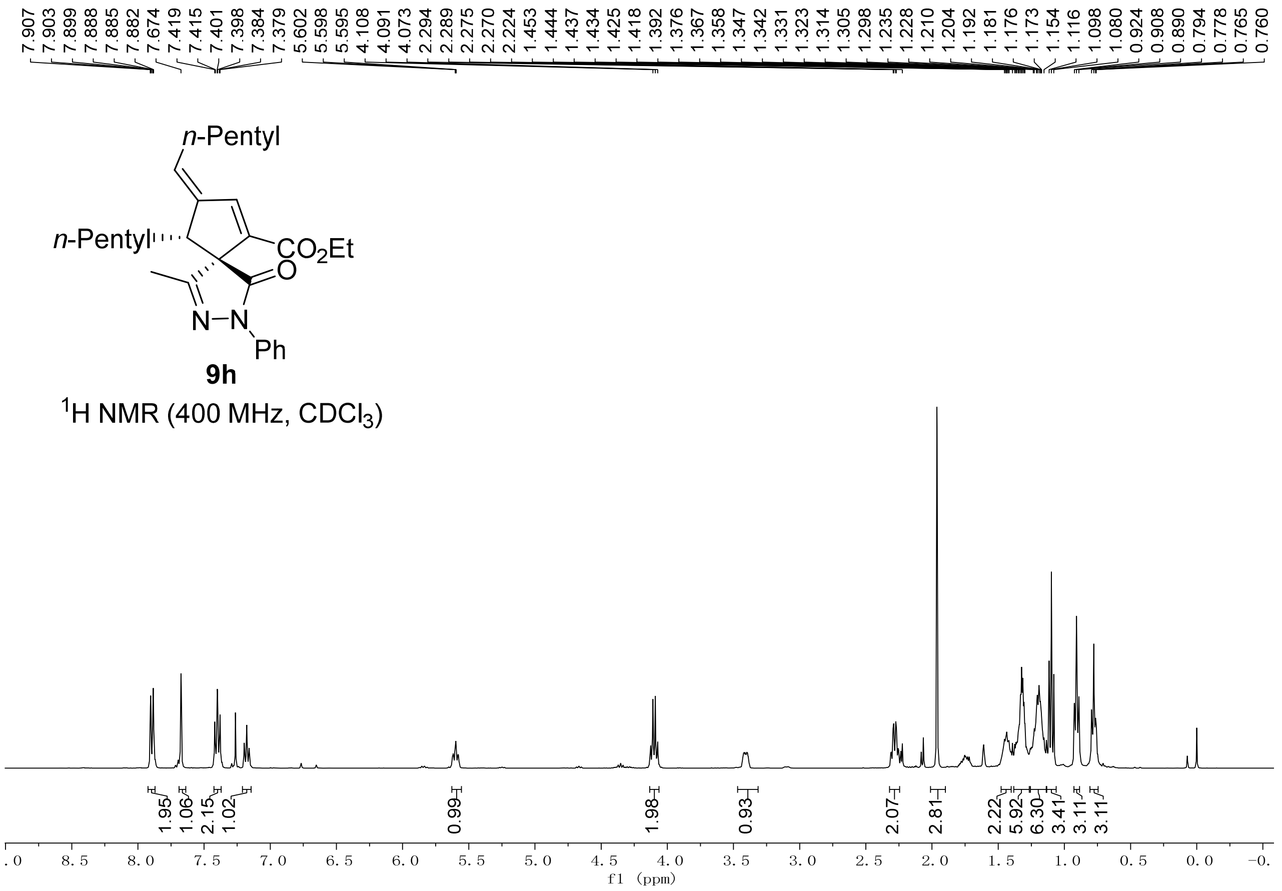
7.907 7.903 7.899 7.888 7.885 7.882 7.674 7.419 7.415 7.401 7.398 7.384 7.379 5.602 5.598 5.595 4.108 4.091 4.073 2.294 2.289 2.275 2.270 2.224 1.453 1.444 1.437 1.434 1.425 1.418 1.392 1.376 1.367 1.358 1.347 1.342 1.331 1.323 1.314 1.305 1.298 1.235 1.228 1.210 1.204 1.192 1.181 1.176 1.173 1.154 1.116 1.098 1.080 0.924 0.908 0.890 0.794 0.778 0.765 0.760
n-Pentyl
n-Pentyl
CO2Et
O
N–N
Ph
9h
1H NMR (400 MHz, CDCl3)
1.95 1.06 2.15 1.02 0.99 1.98 0.93 2.07 2.81 2.22 5.92 6.30 3.41 3.11 3.11
f1 (ppm)

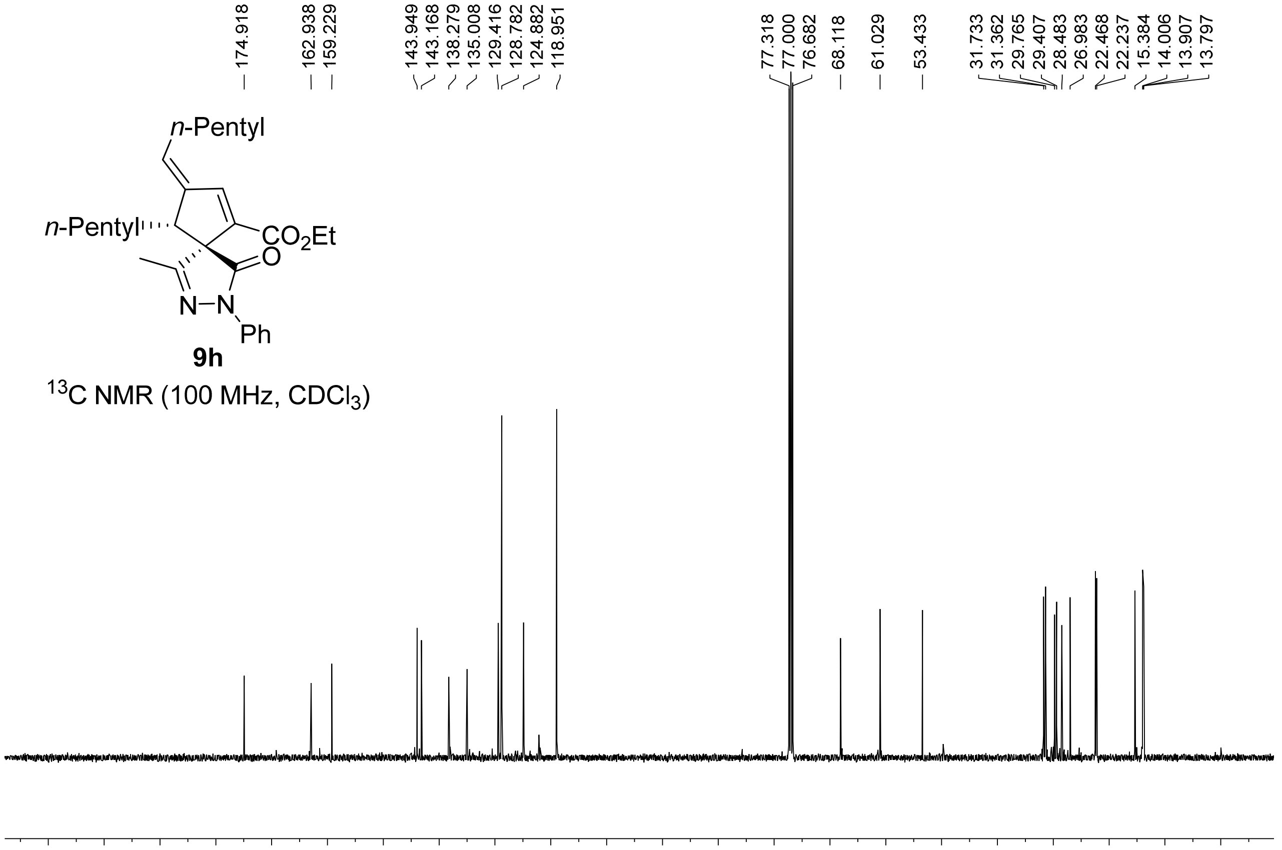
174.918 162.938 159.229 143.949 143.168 138.279 135.008 129.416 128.782 124.882 118.951 77.318 77.000 76.682 68.118 61.029 53.433 31.733 31.362 29.765 29.407 28.483 26.983 22.468 22.237 15.384 14.006 13.907 13.797
n-Pentyl
n-Pentyl
CO2Et
O
N–N
Ph
9h
13C NMR (100 MHz, CDCl3)
f1 (ppm)

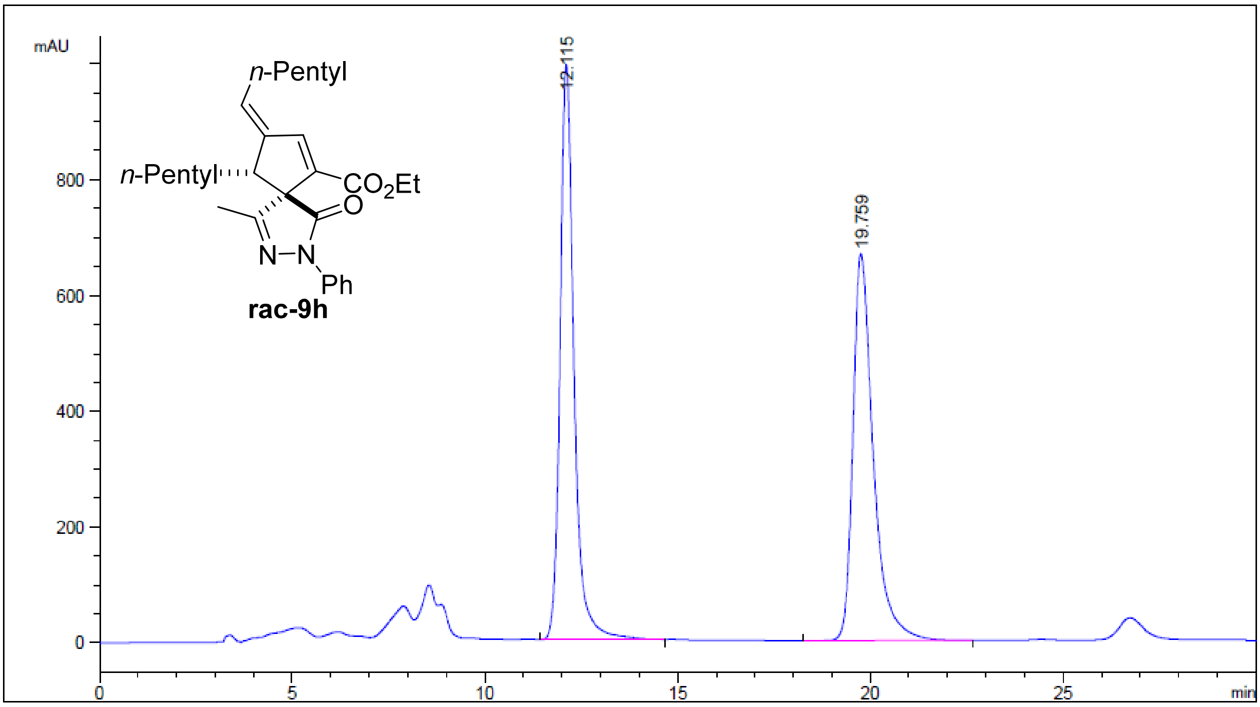

Peak #	RetTime [min]	Type	Width [min]	Area mAU *s	Height [mAU]	Area %
1	12.115	BB	0.3763	2.49511e4	992.59515	50.4168
2	19.759	BB	0.5494	2.45386e4	667.99701	49.5832

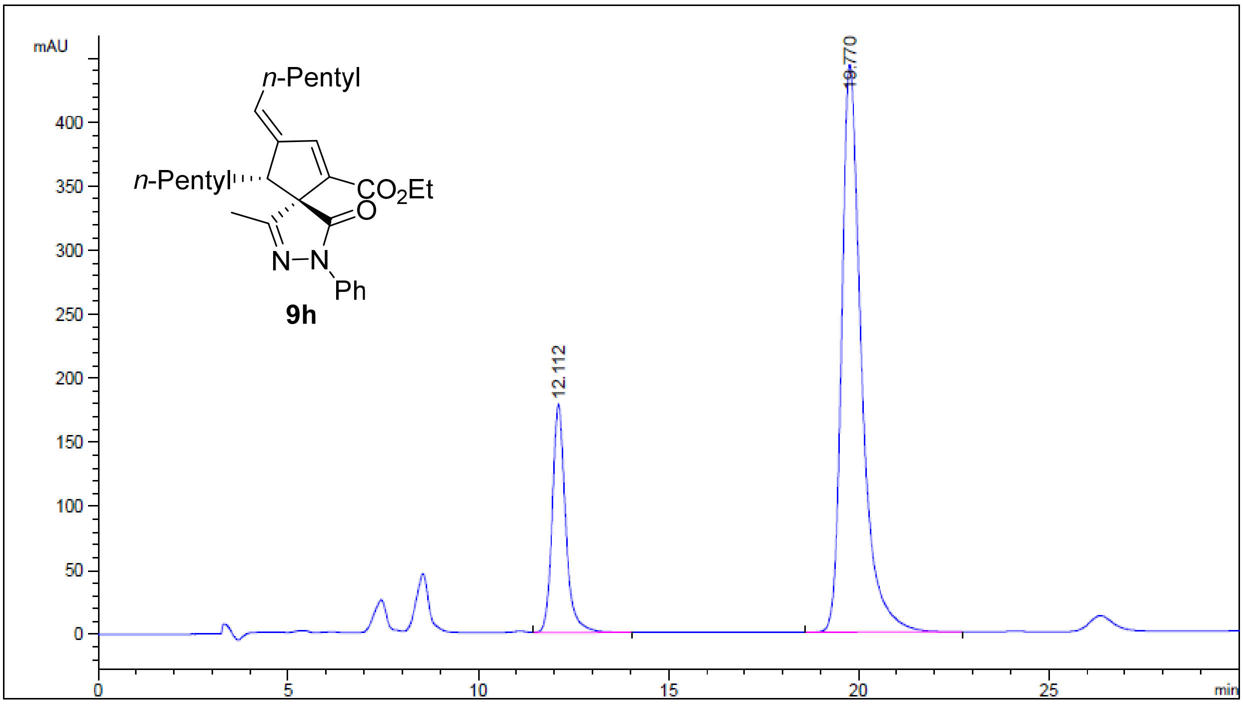

Peak #	RetTime [min]	Type	Width [min]	Area mAU *s	Height [mAU]	Area %
1	12.112	VB	0.3675	4340.00488	177.97380	21.2563
2	19.770	BB	0.5426	1.60775e4	443.18069	78.7437

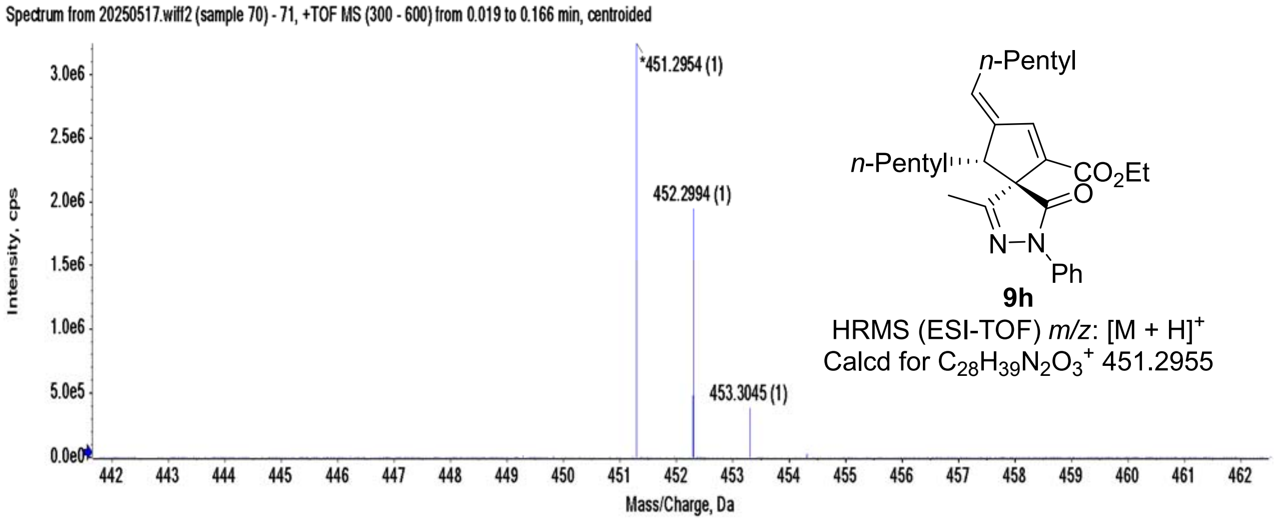

9h

HRMS (ESI-TOF) *m/z*: $[M + H]^+$
Calcd for $C_{28}H_{39}N_2O_3^+$ 451.2955

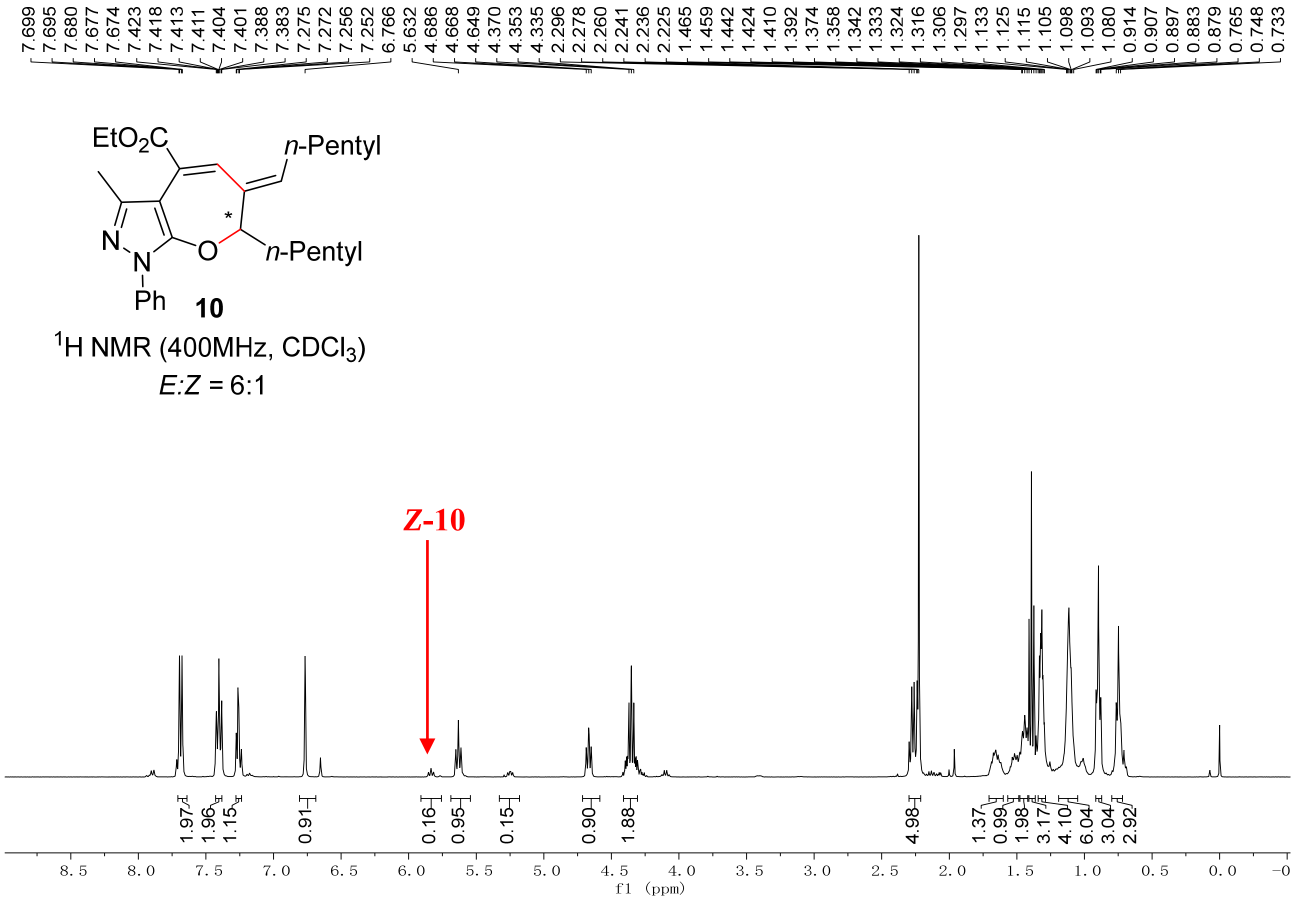
EtO2C
n-Pentyl
n-Pentyl
N
N
O
Ph
10
1H NMR (400MHz, CDCl3)
E:Z = 6:1
Z-10
f1 (ppm)

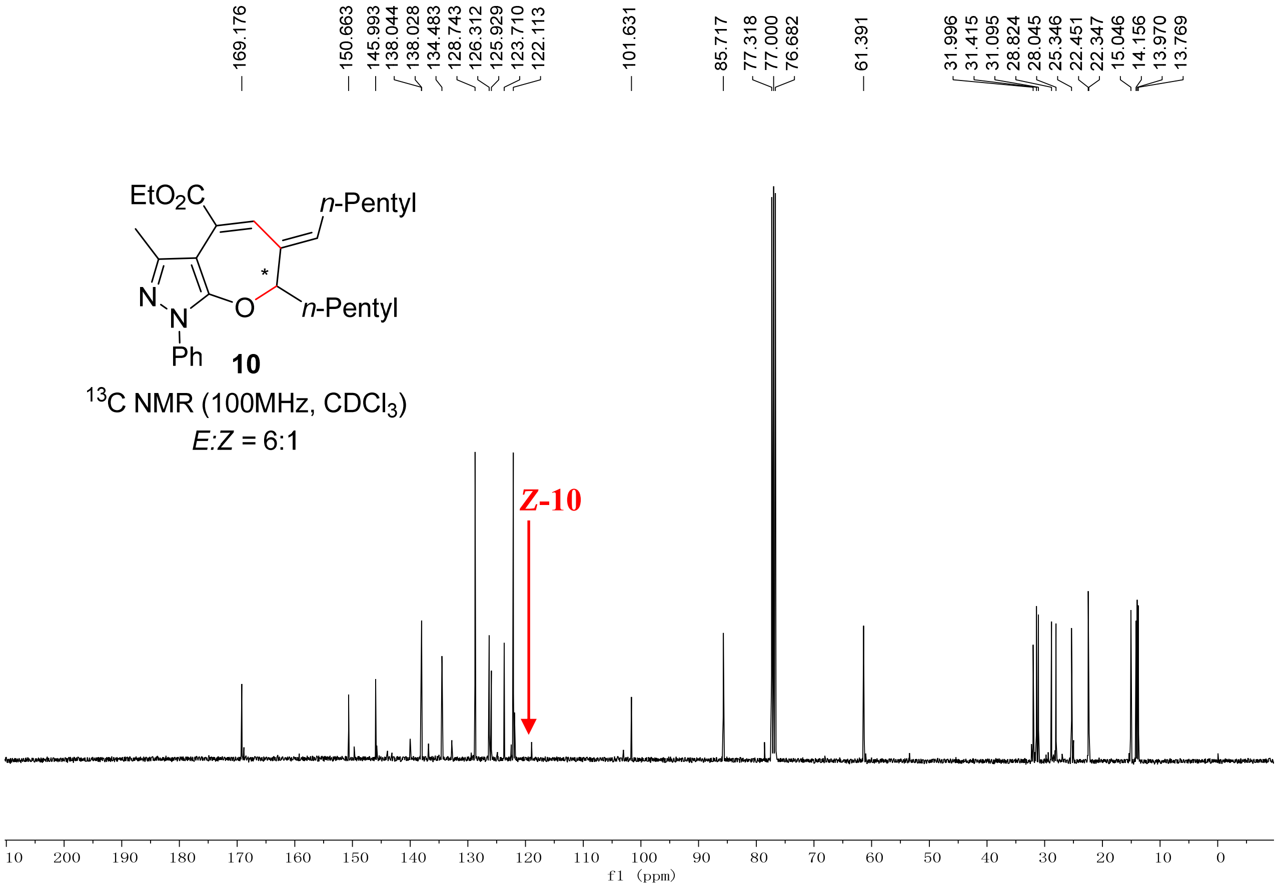
EtO2C
n-Pentyl
n-Pentyl
N
N
O
Ph
10
13C NMR (100MHz, CDCl3)
E:Z = 6:1
Z-10
f1 (ppm)

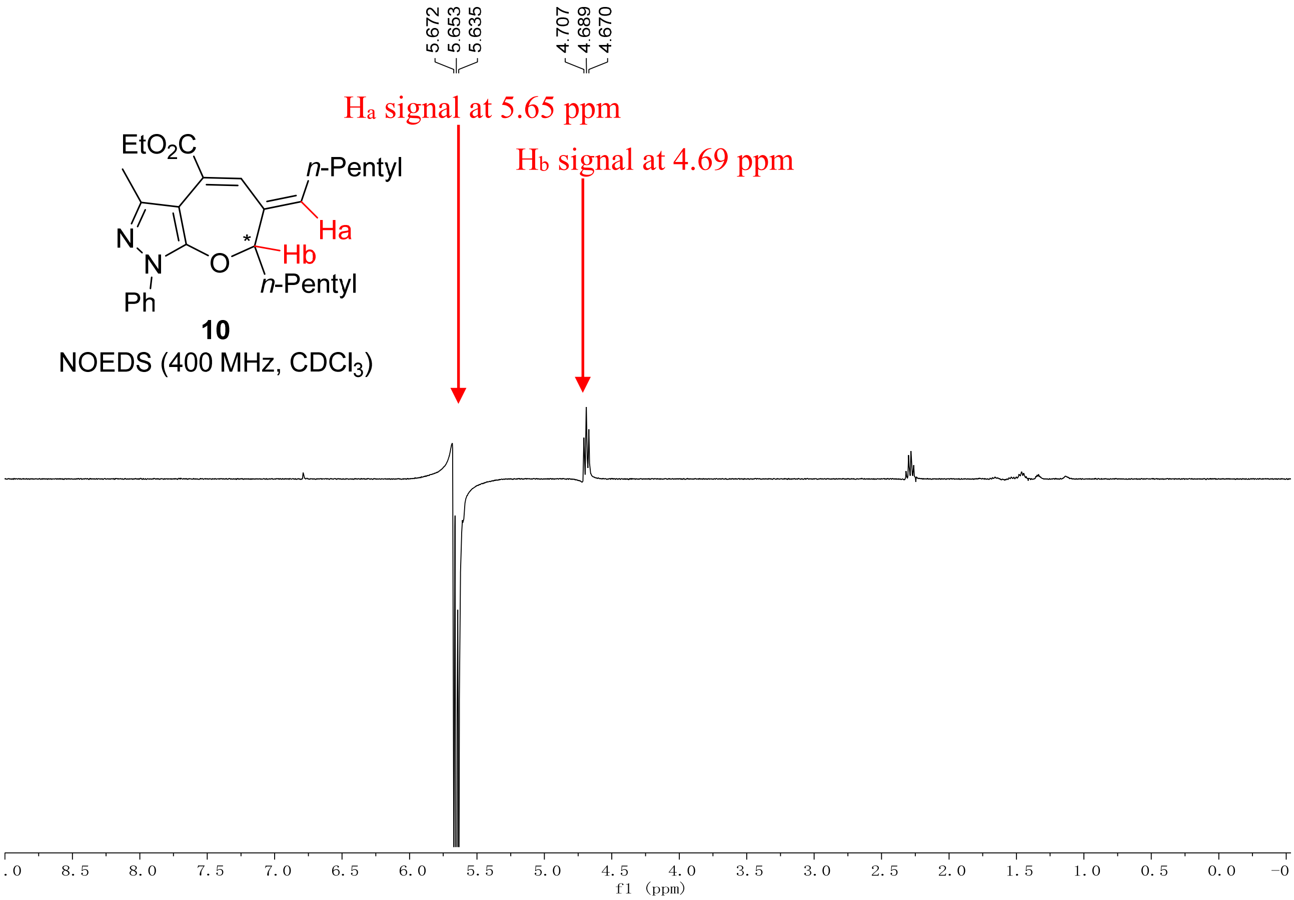
5.672
5.653
5.635
4.707
4.689
4.670
Ha signal at 5.65 ppm
Hb signal at 4.69 ppm
EtO2C
n-Pentyl
Ha
Hb
n-Pentyl
N
N
O
Ph
10
NOEDS (400 MHz, CDCl3)
f1 (ppm)

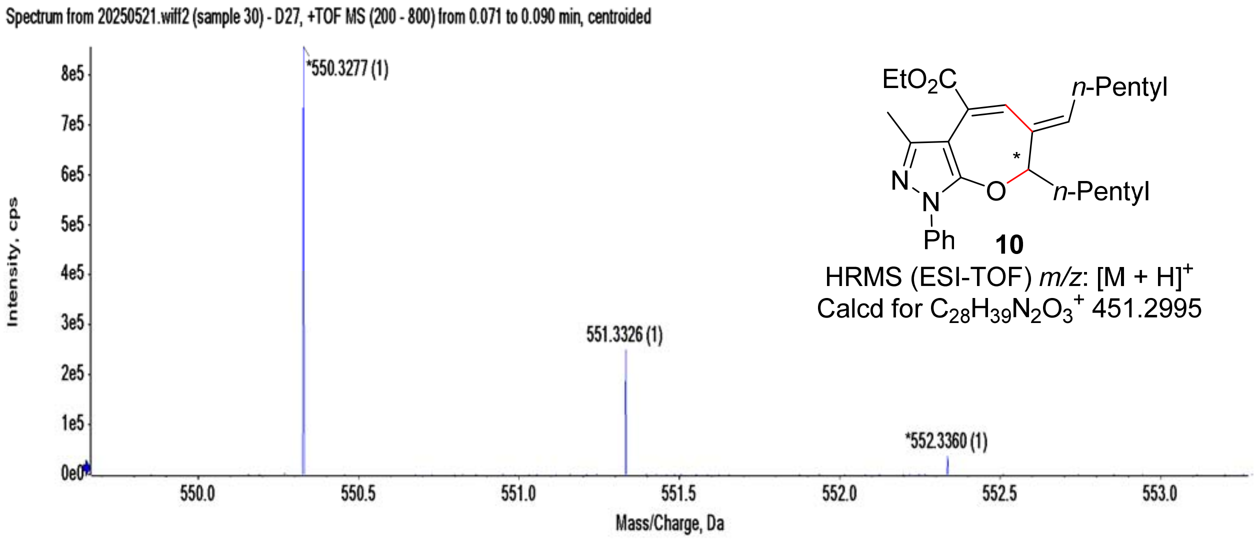
Spectrum from 20250521.wiff2 (sample 30) - D27, +TOF MS (200 - 800) from 0.071 to 0.090 min, centroided
*550.3277 (1)
551.3326 (1)
*552.3360 (1)
Intensity, cps
Mass/Charge, Da
EtO2C
n-Pentyl
n-Pentyl
Ph
10
HRMS (ESI-TOF) m/z: [M + H]+
Calcd for C28H39N2O3+ 451.2995

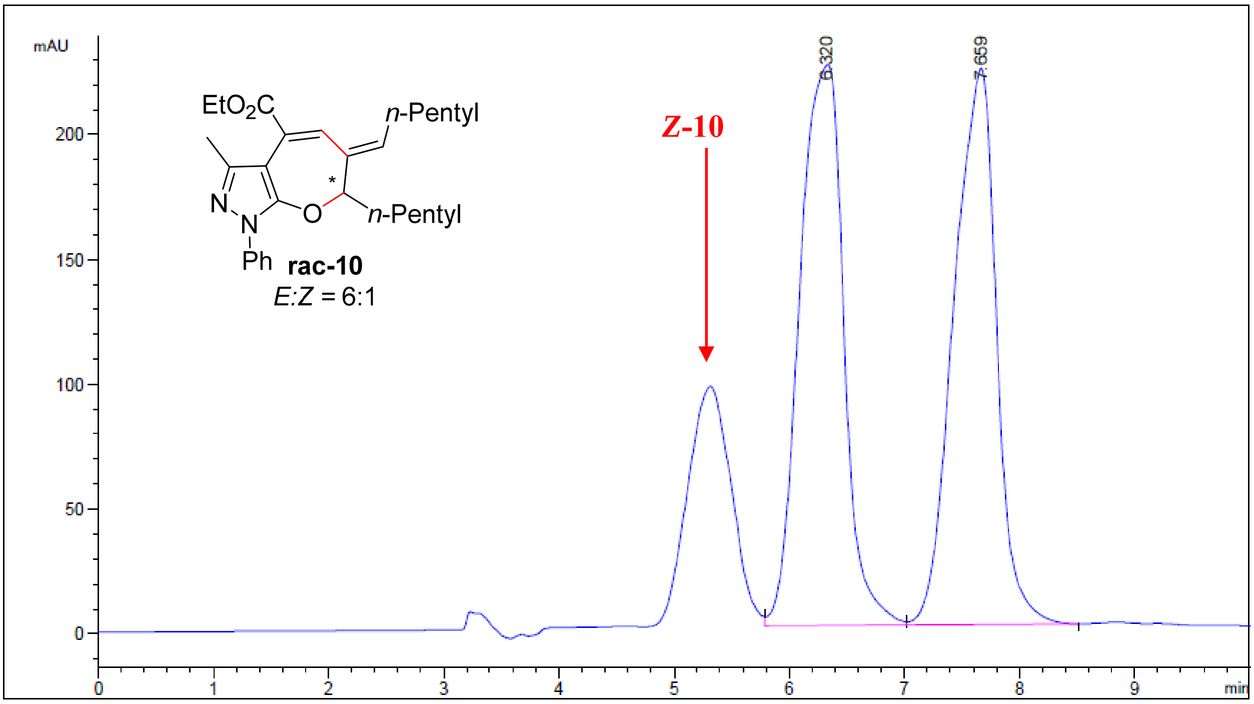

Peak #	RetTime [min]	Type	Width [min]	Area mAU *s	Height [mAU]	Area %
1	6.320	VV	0.4282	5984.33154	223.98788	51.1077
2	7.659	VB	0.4173	5724.92041	222.05066	48.8923

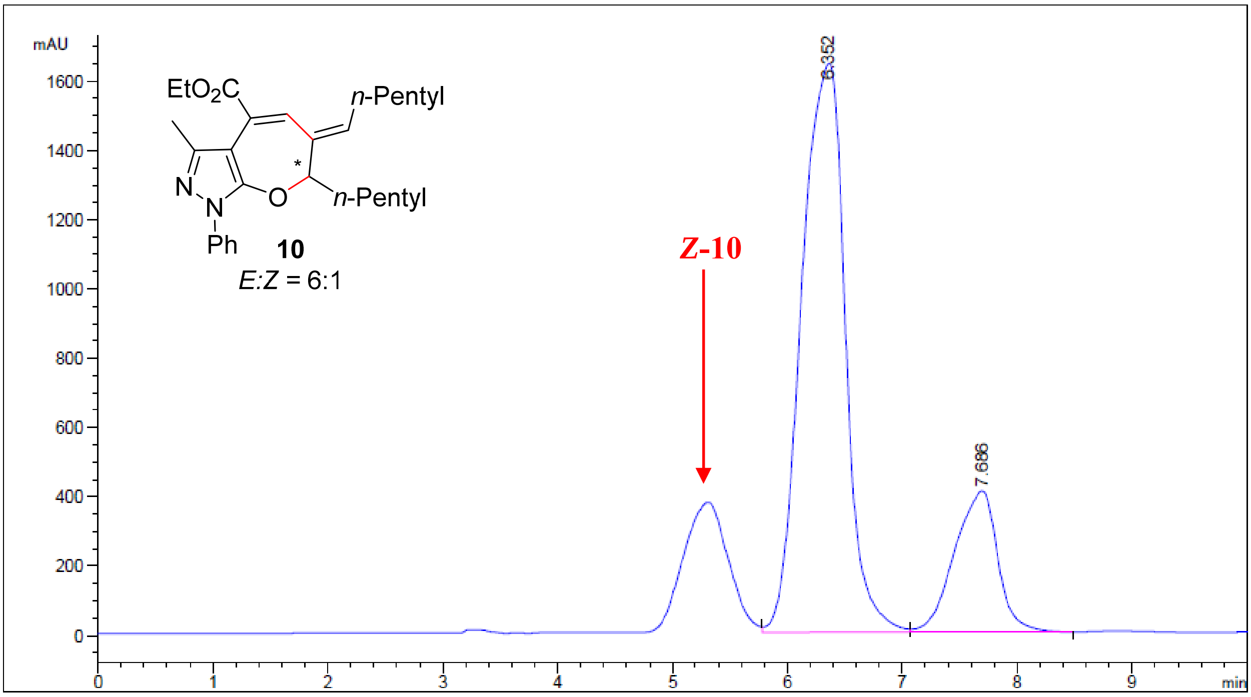

Peak #	RetTime [min]	Type	Width [min]	Area mAU *s	Height [mAU]	Area %
1	6.352	VV	0.4305	4.33028e4	1639.46289	80.3376
2	7.686	VB	0.4264	1.05982e4	406.57404	19.6624

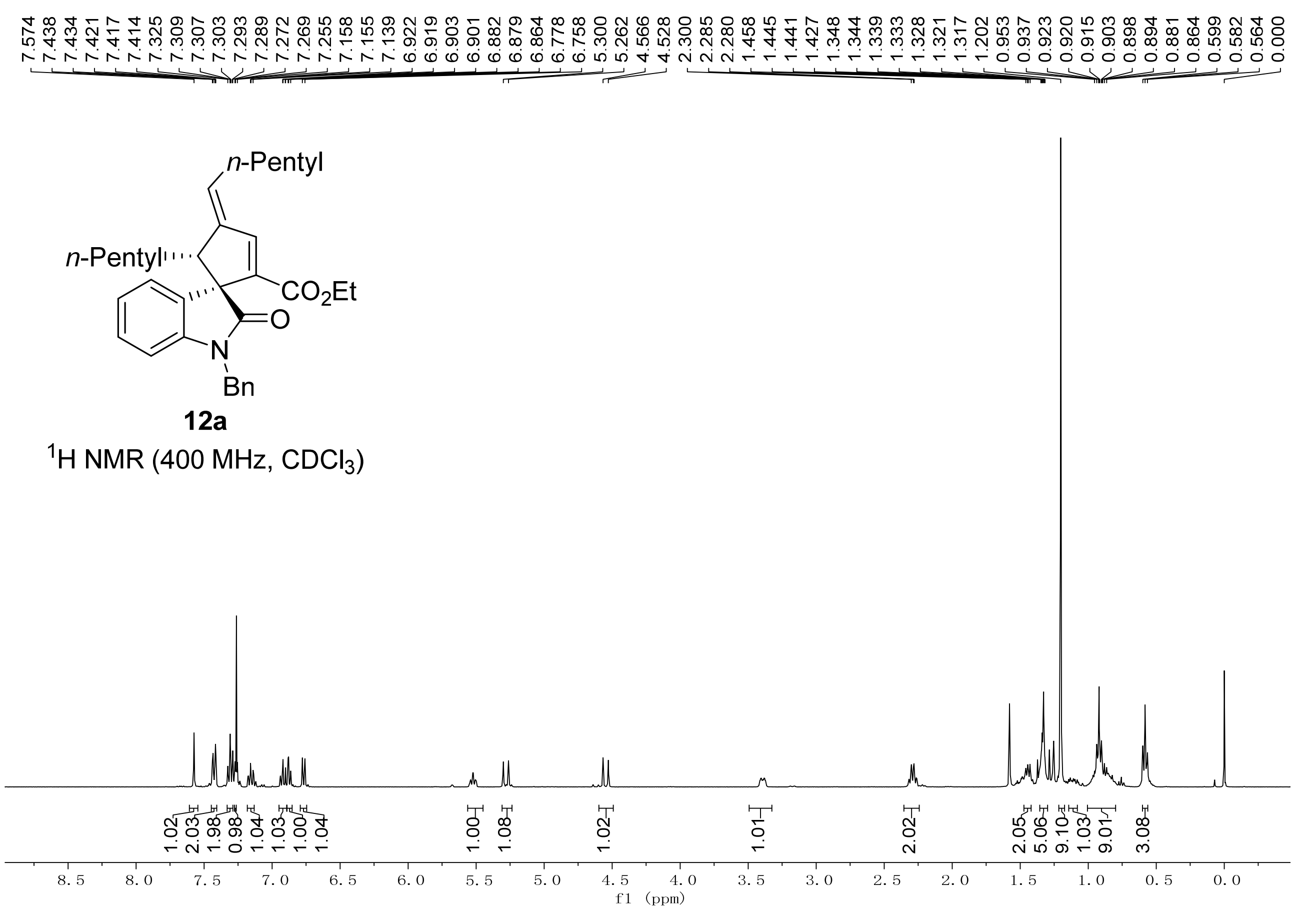

12a

^{1}H NMR (400 MHz, $CDCl_3$)

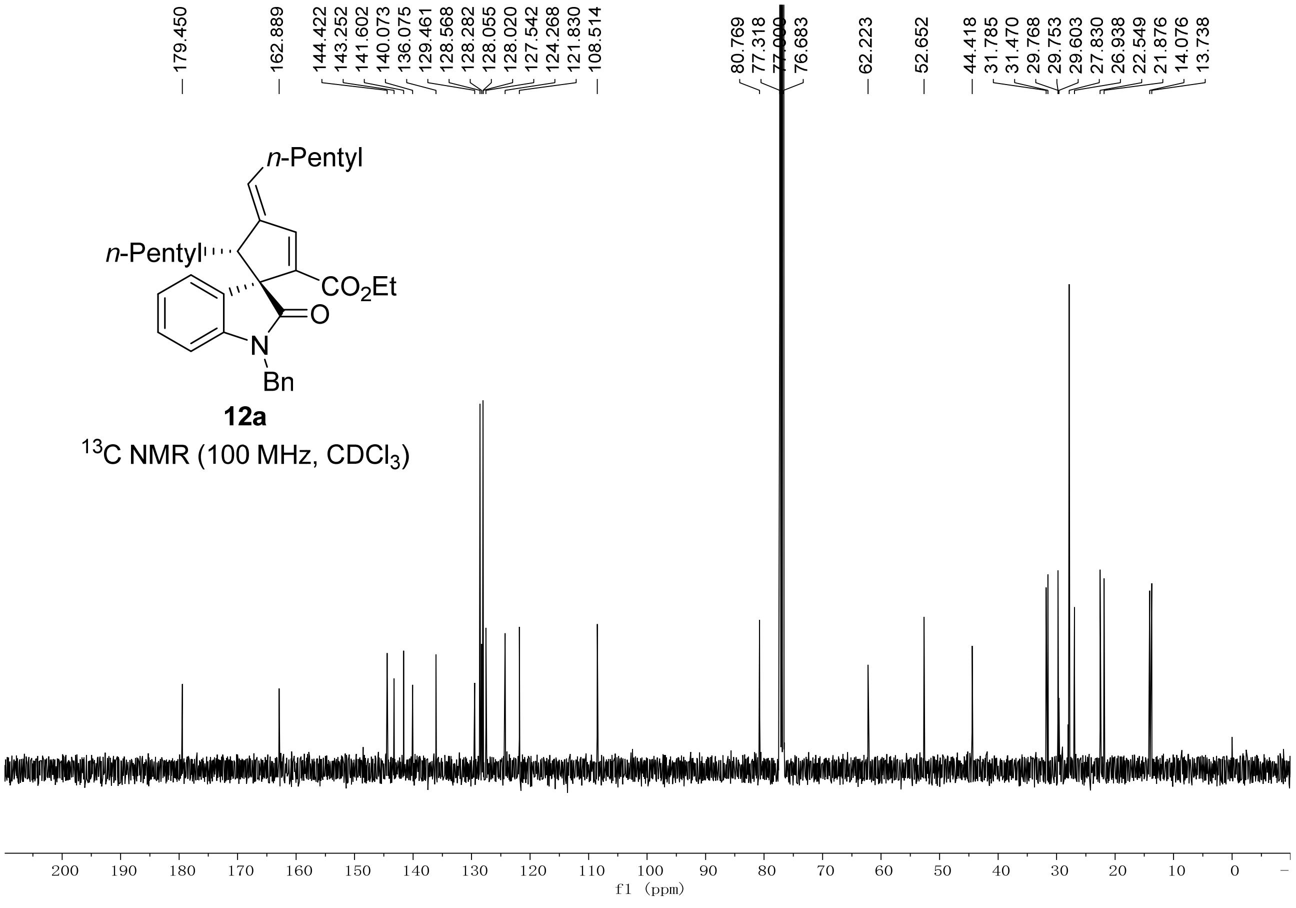

12a

^{13}C NMR (100 MHz, $CDCl_3$)

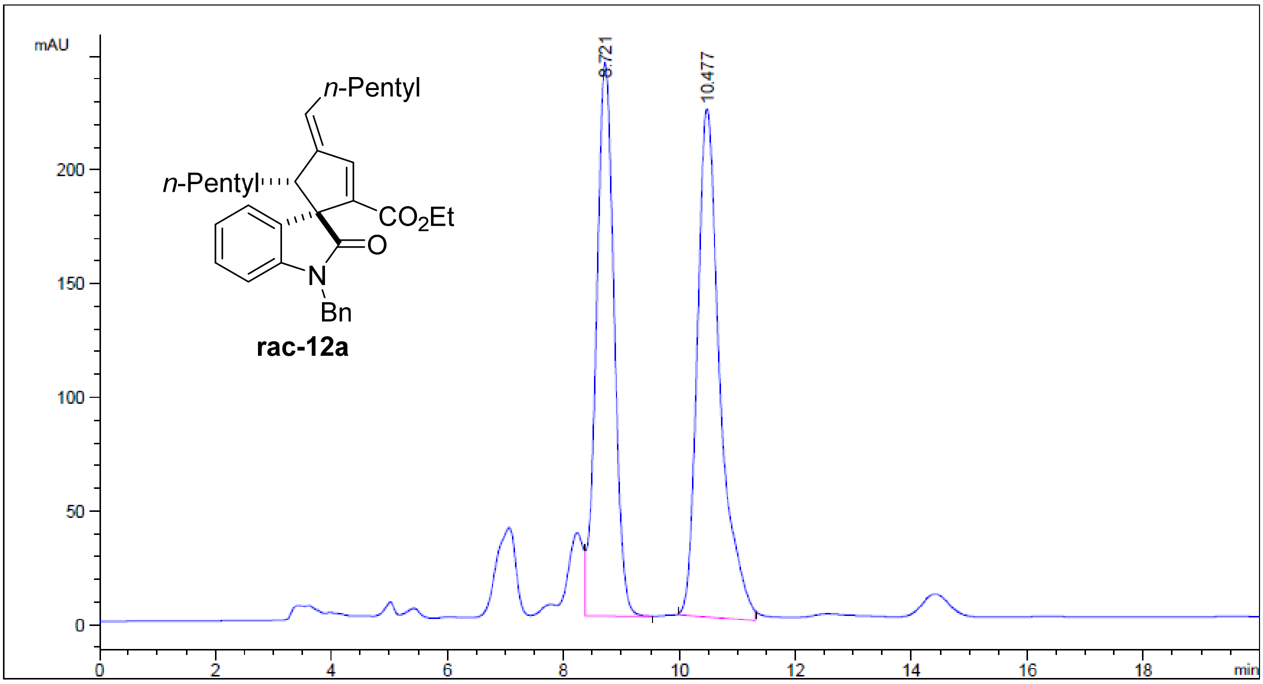

Peak #	RetTime [min]	Type	Width [min]	Area mAU *s	Height [mAU]	Area %
1	8.721	VB	0.3284	5150.94092	243.39377	45.9231
2	10.477	MM	0.4524	6065.51709	223.45348	54.0769

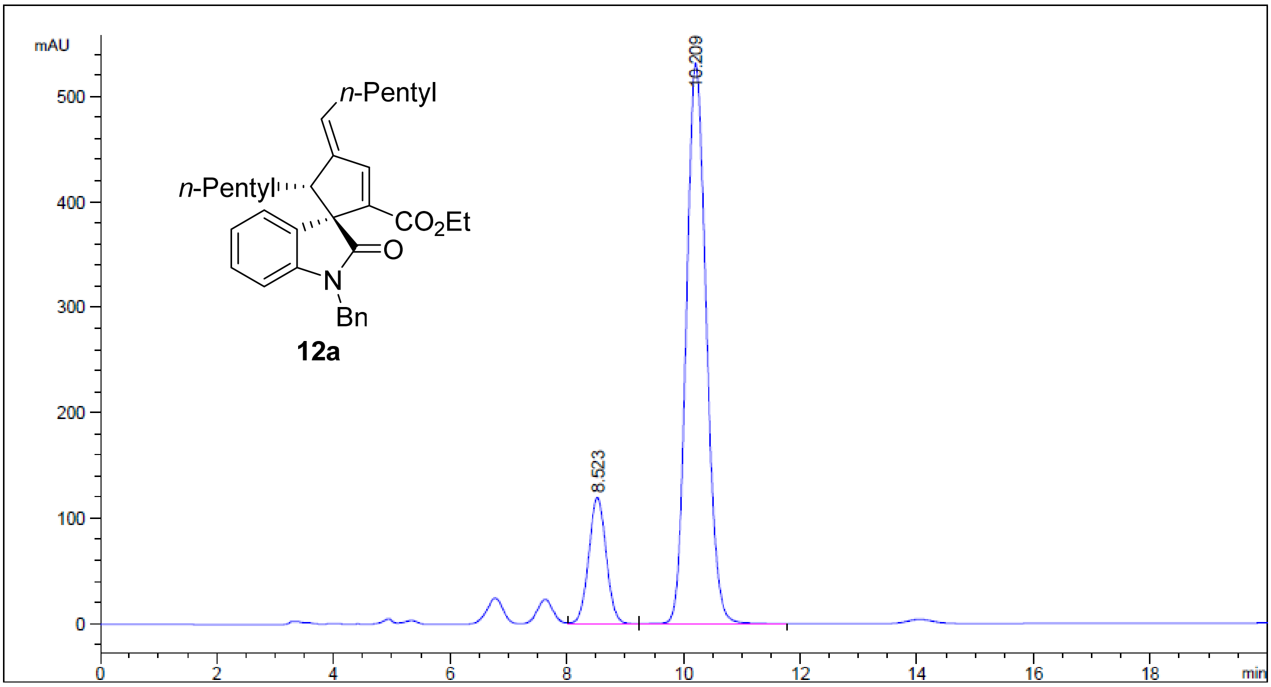

Peak #	RetTime [min]	Type	Width [min]	Area mAU *s	Height [mAU]	Area %
1	8.523	VB	0.3247	2484.88062	119.23824	16.4971
2	10.209	BB	0.3696	1.25777e4	530.84167	83.5029

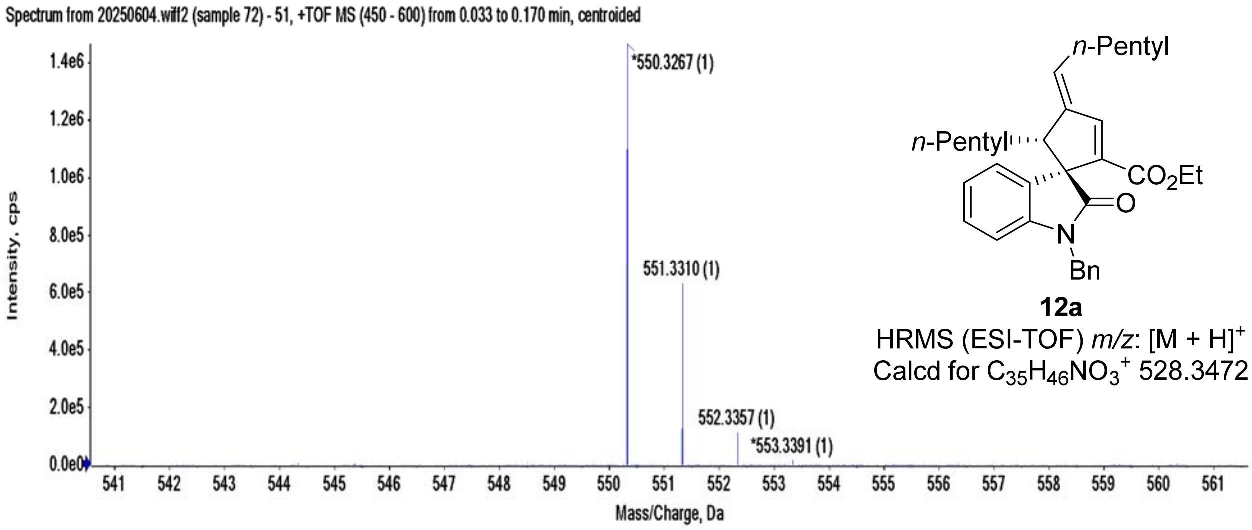

12a

HRMS (ESI-TOF) *m/z*: $[M + H]^+$

Calcd for $C_{35}H_{46}NO_3^+$ 528.3472

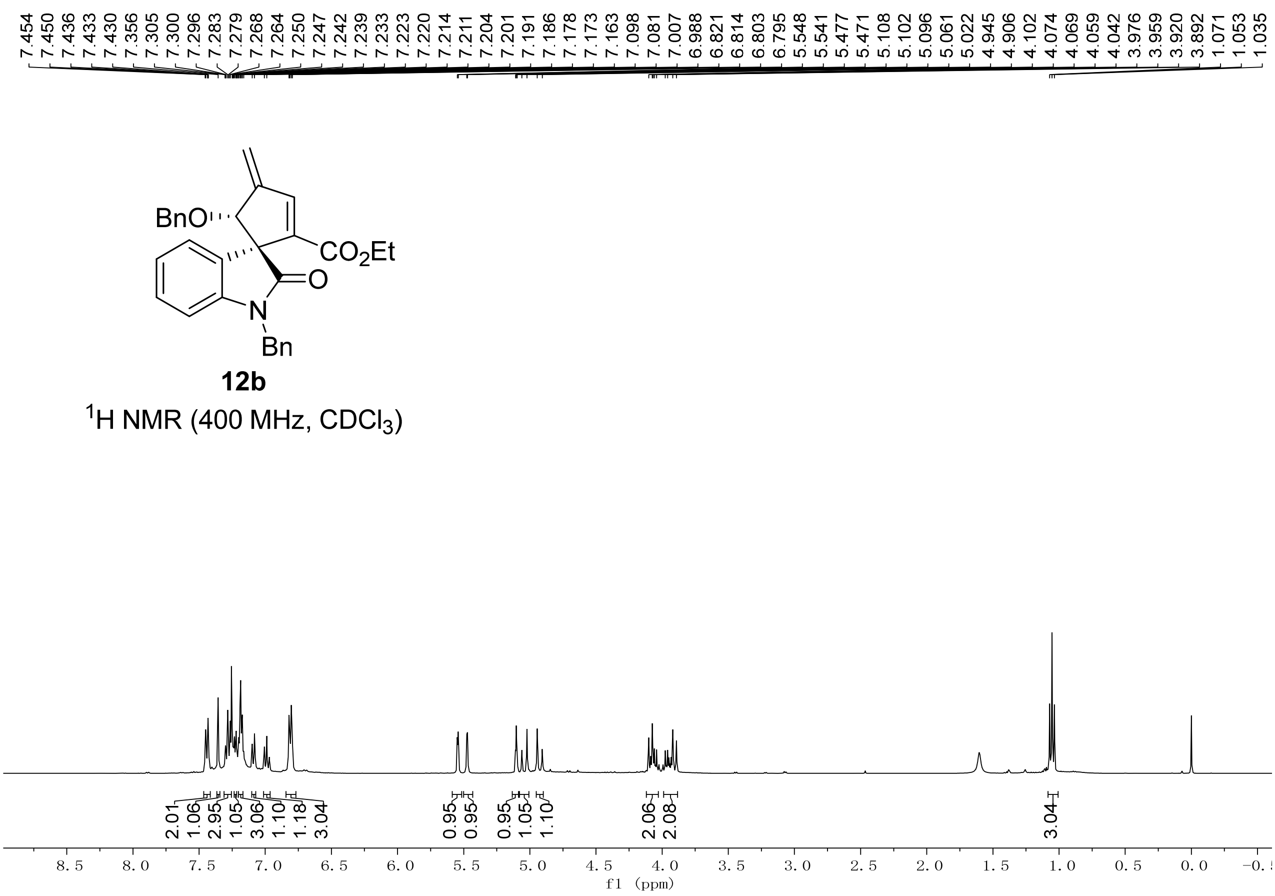

12b

^{1}H NMR (400 MHz, $CDCl_3$)

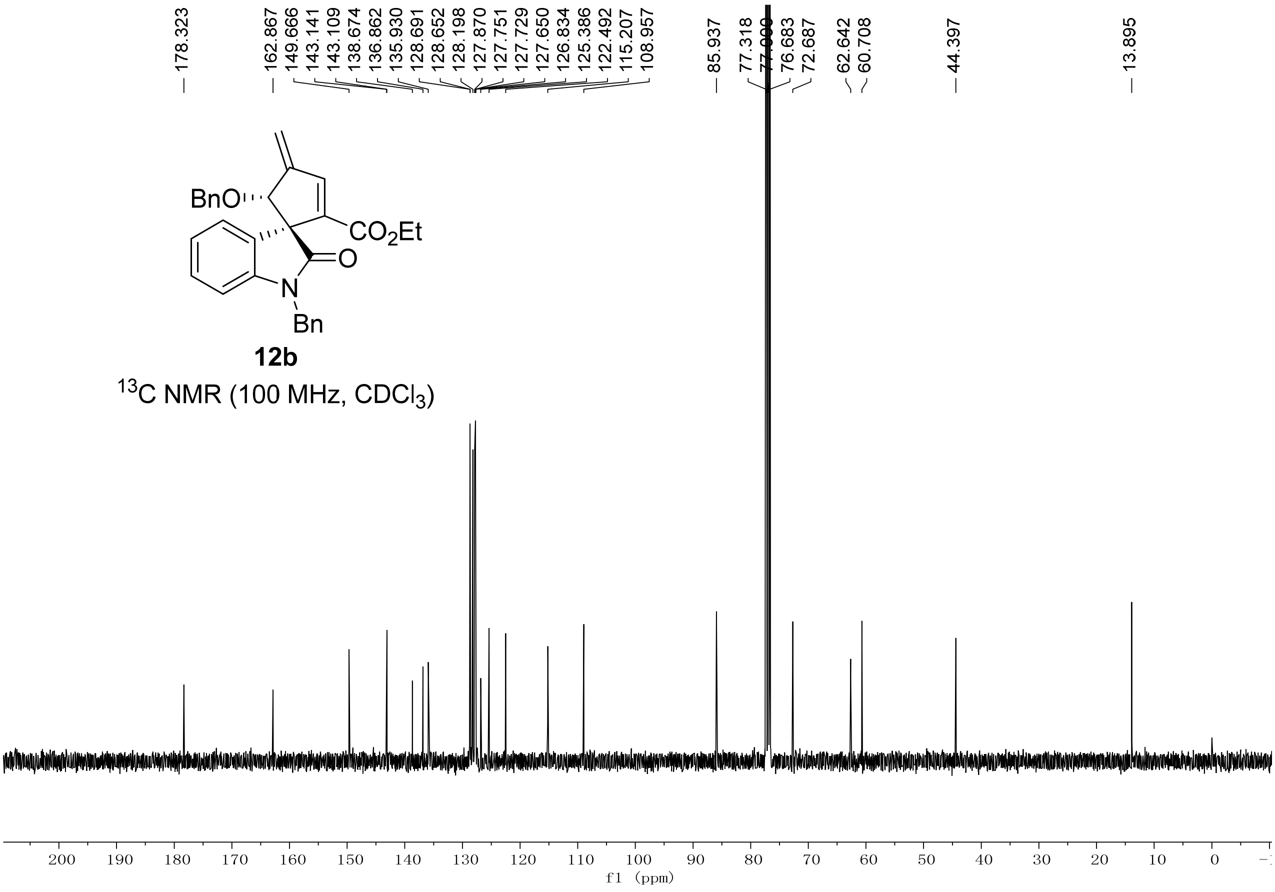

12b

^{13}C NMR (100 MHz, $CDCl_3$)

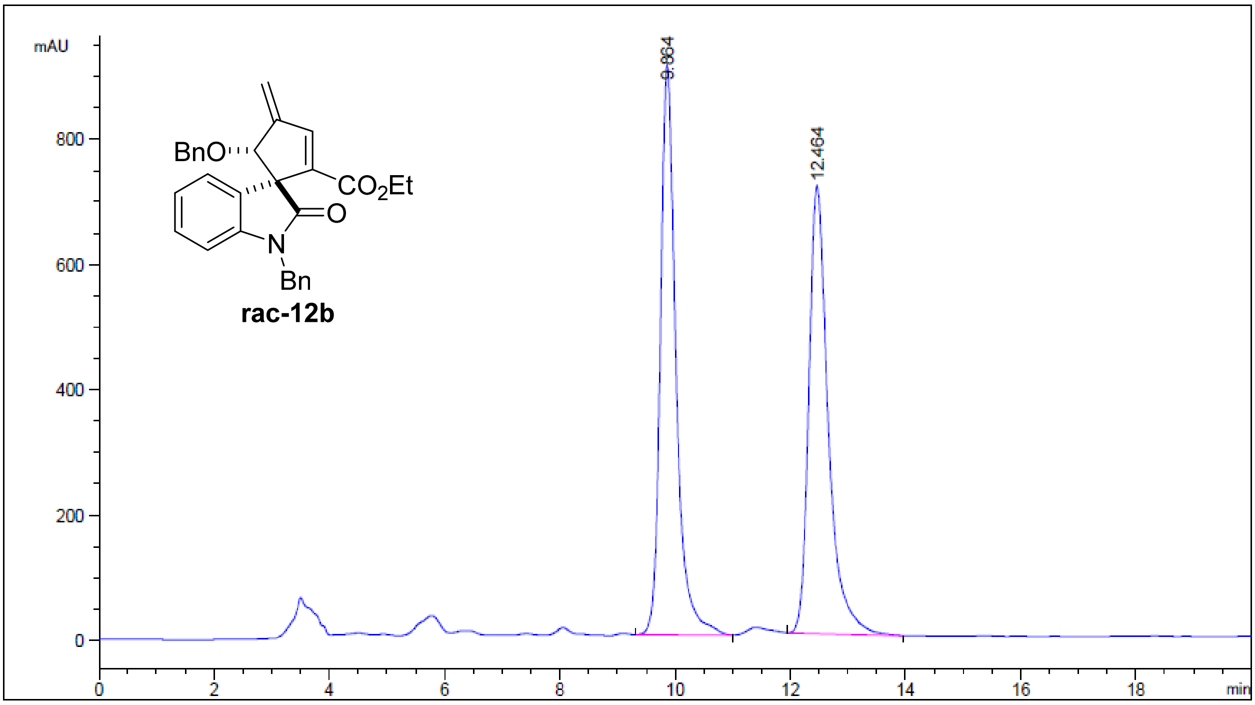

Peak #	RetTime [min]	Type	Width [min]	Area mAU *s	Height [mAU]	Area %
1	9.864	BB	0.2796	1.70315e4	909.57074	50.5095
2	12.464	BBA	0.3511	1.66878e4	714.63739	49.4905

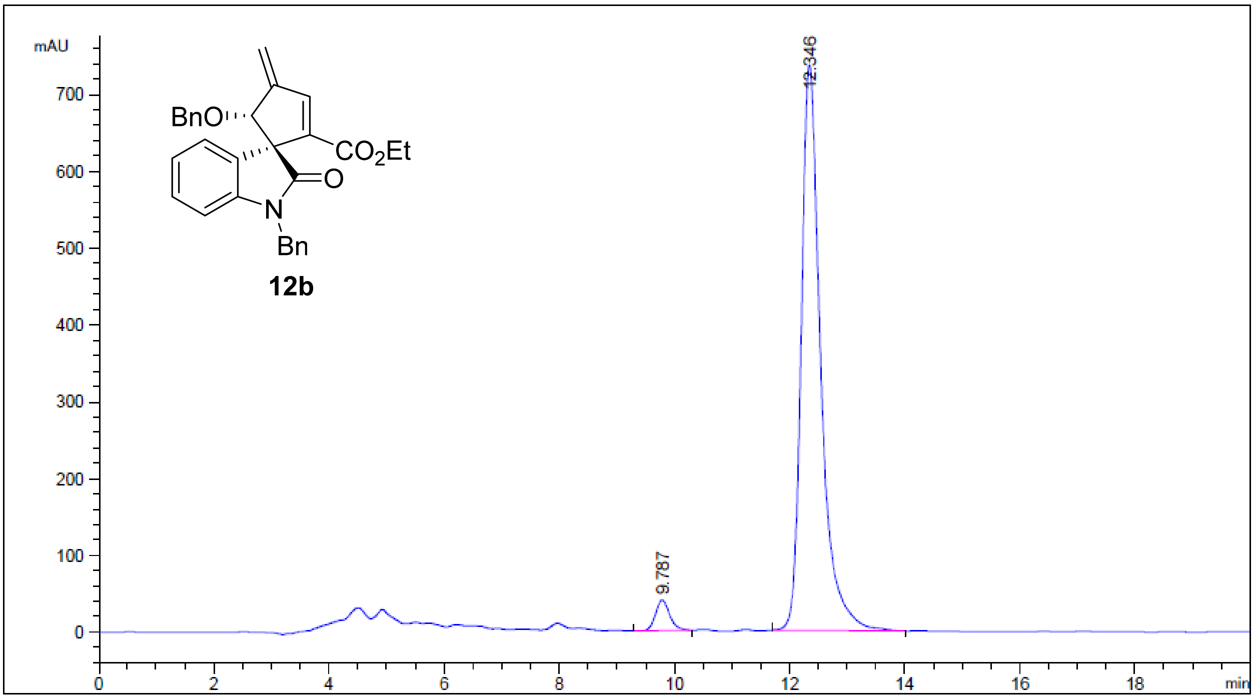

Peak #	RetTime [min]	Type	Width [min]	Area mAU *s	Height [mAU]	Area %
1	9.787	BB	0.2585	671.15222	39.66810	3.7422
2	12.346	BBA	0.3554	1.72636e4	735.39209	96.2578

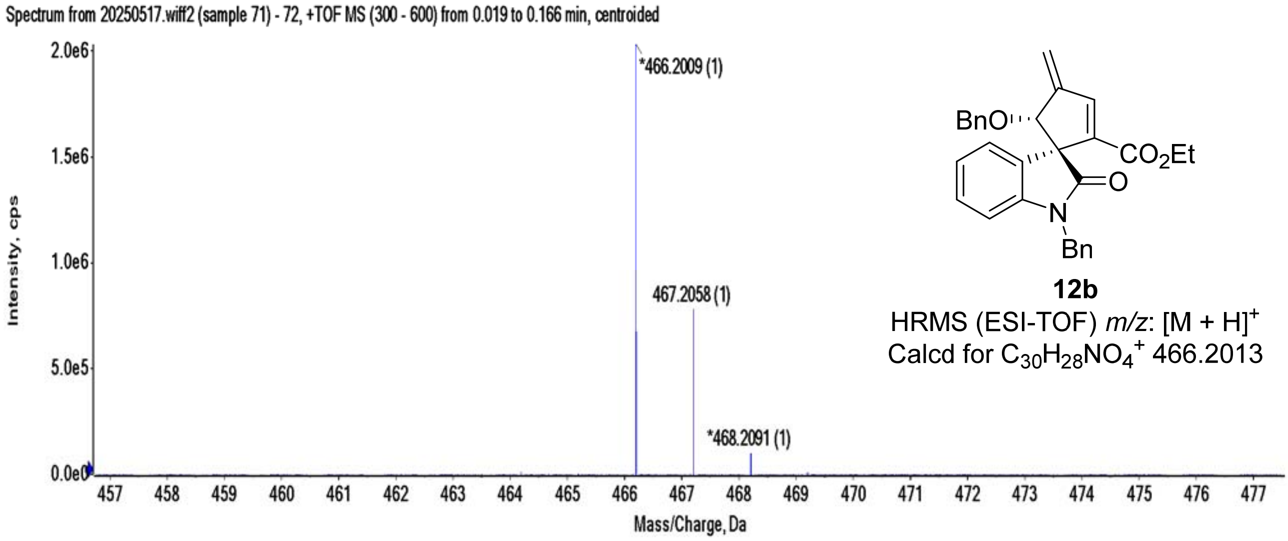

12b

HRMS (ESI-TOF) *m/z*: $[M + H]^+$

Calcd for $C_{30}H_{28}NO_4^+$ 466.2013

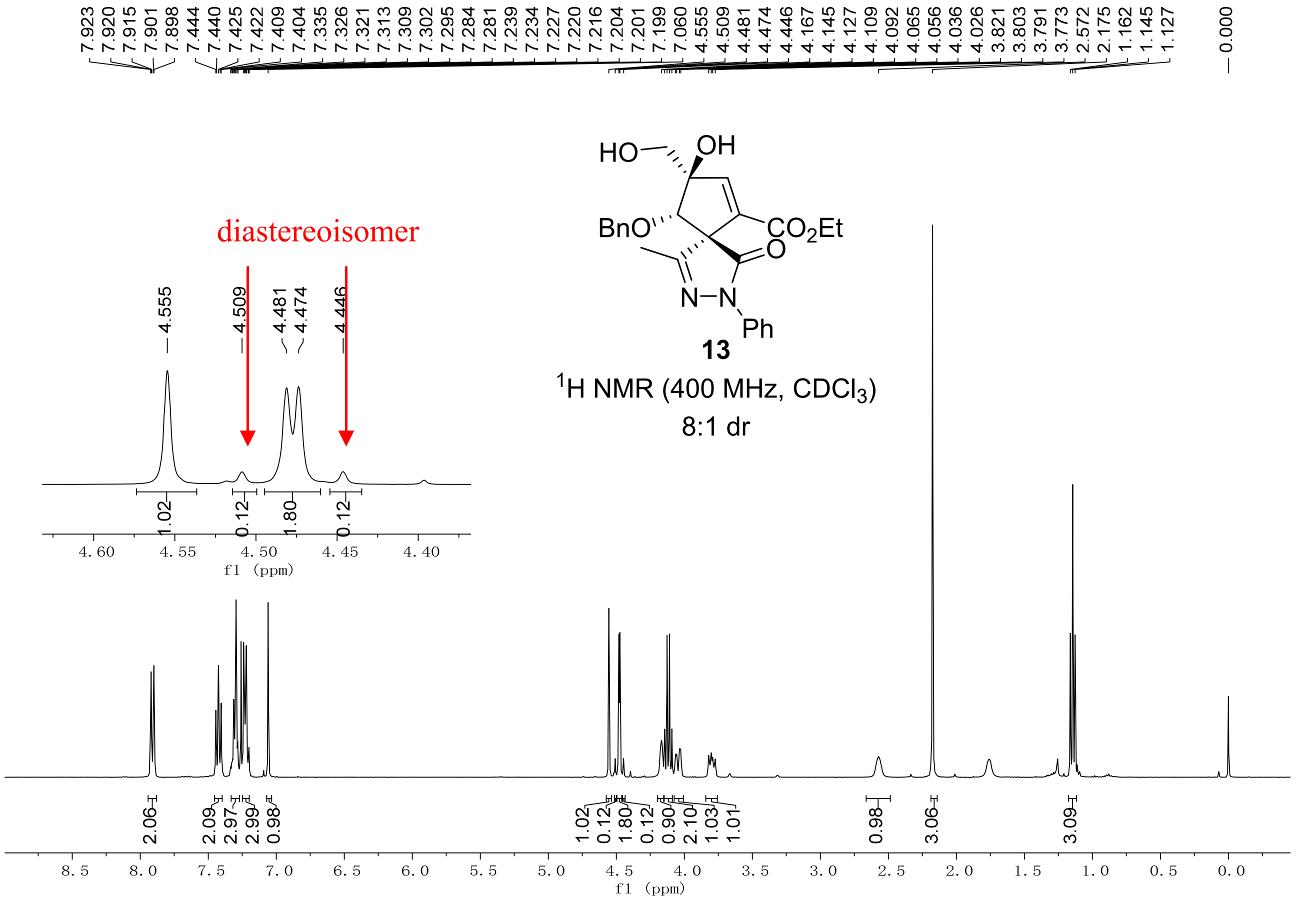

diastereoisomer
13
^{1}H NMR (400 MHz, $CDCl_3$)
8:1 dr

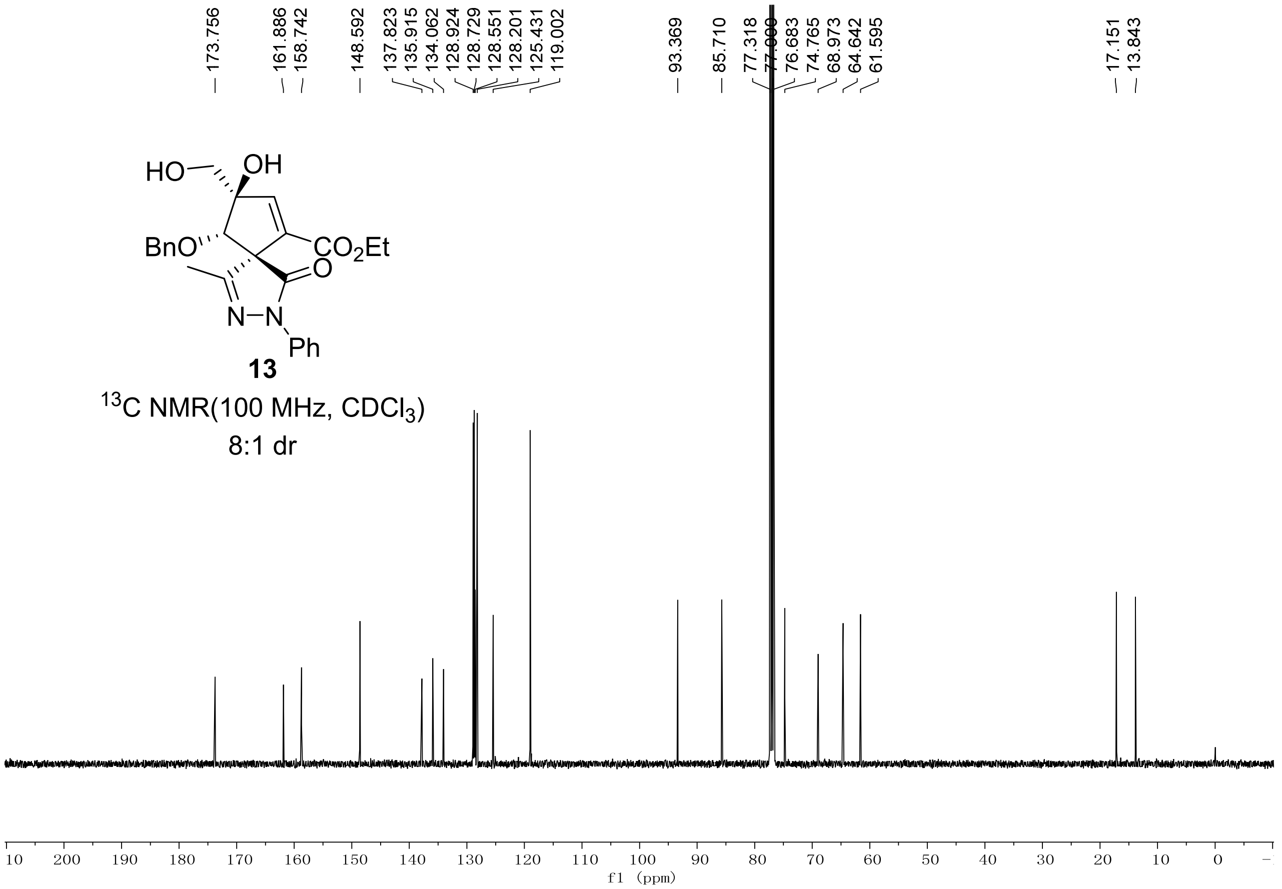

13
^{13}C NMR(100 MHz, $CDCl_3$)
8:1 dr

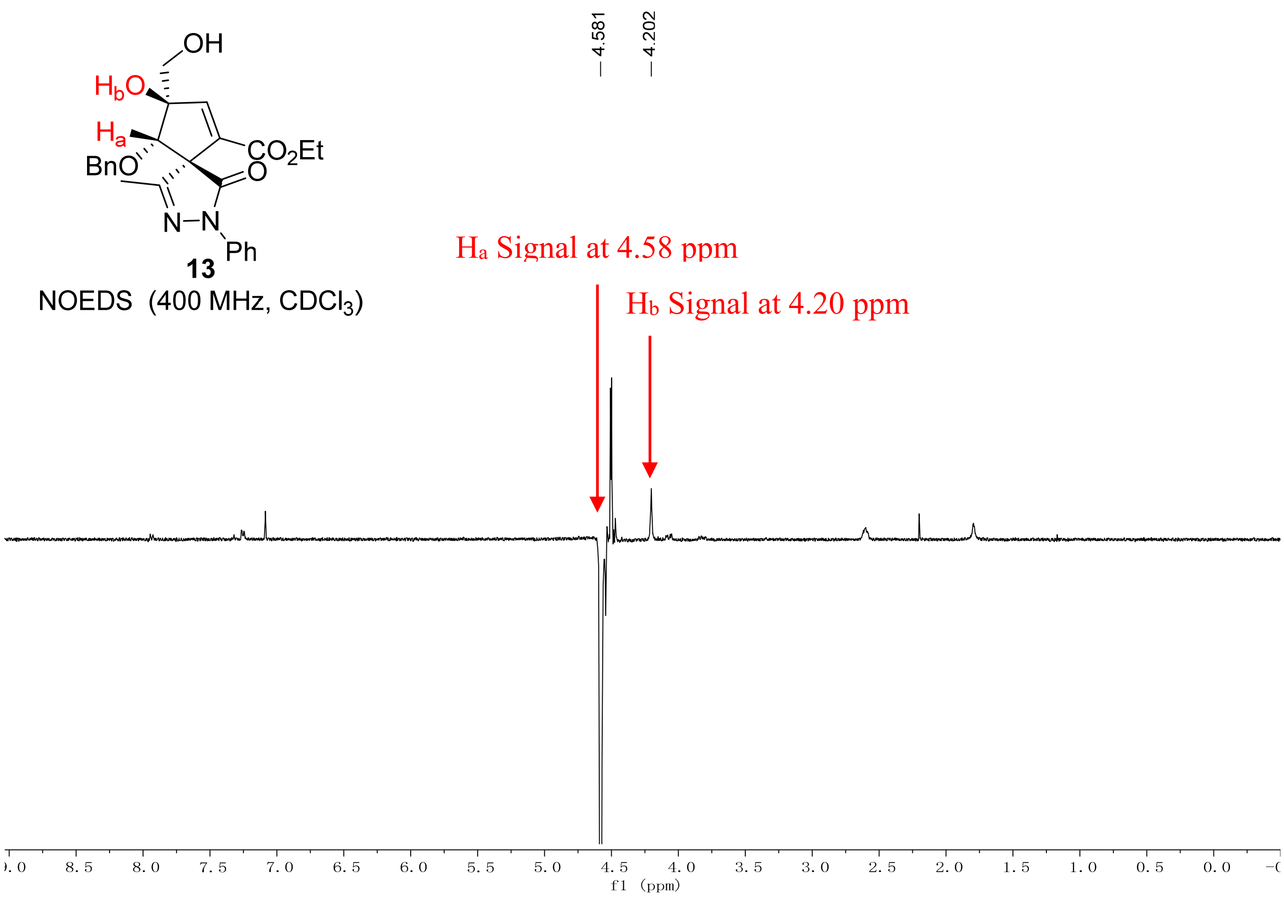

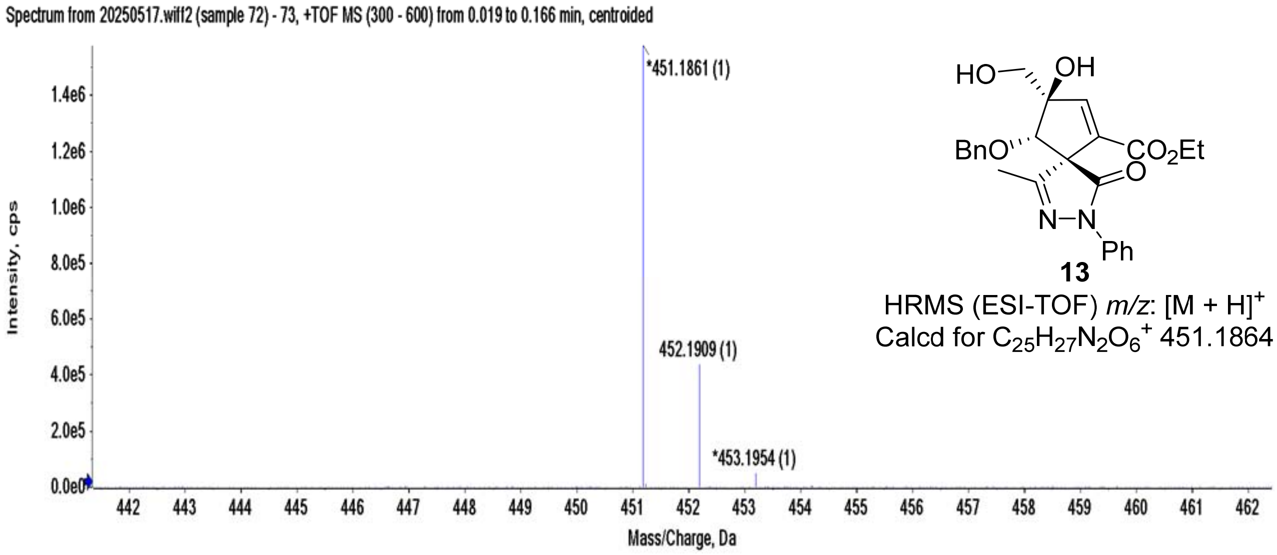

13

HRMS (ESI-TOF) *m/z*: $[M + H]^+$
Calcd for $C_{25}H_{27}N_2O_6^+$ 451.1864

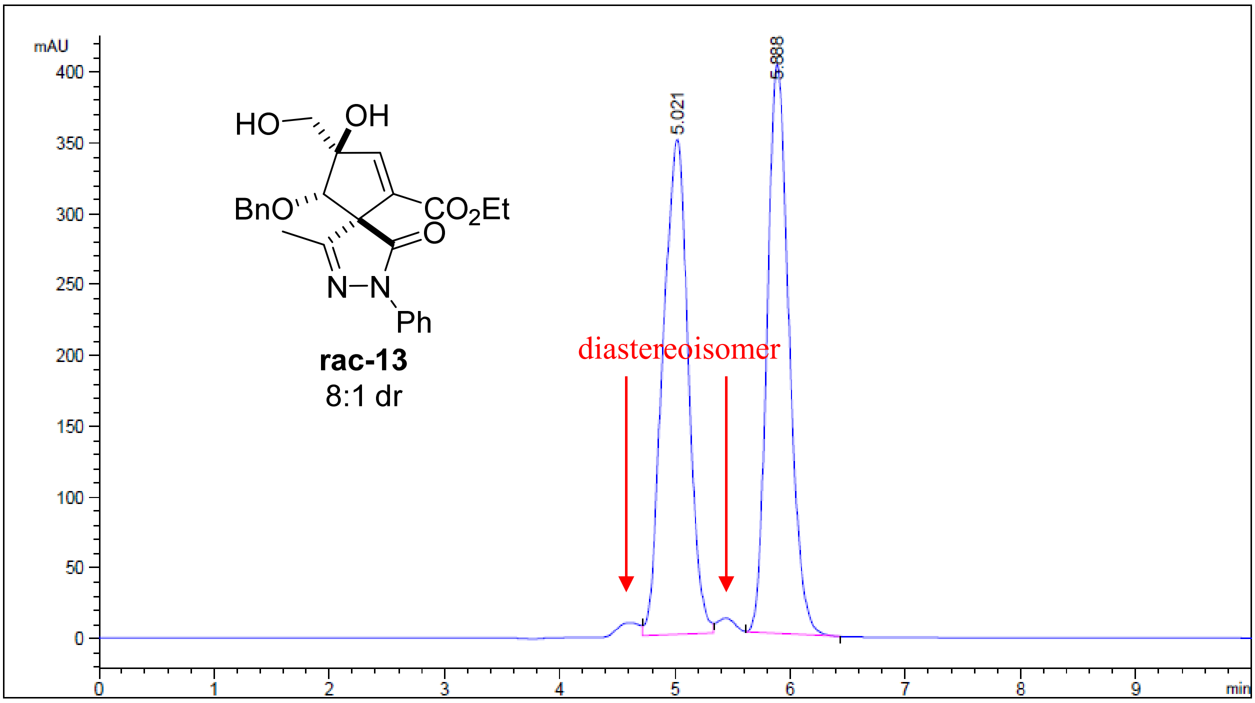

Peak #	RetTime [min]	Type	Width [min]	Area mAU *s	Height [mAU]	Area %
1	5.021	VV	0.2529	5358.29883	349.95923	50.2746
2	5.888	BB	0.2018	5299.76709	402.15475	49.7254

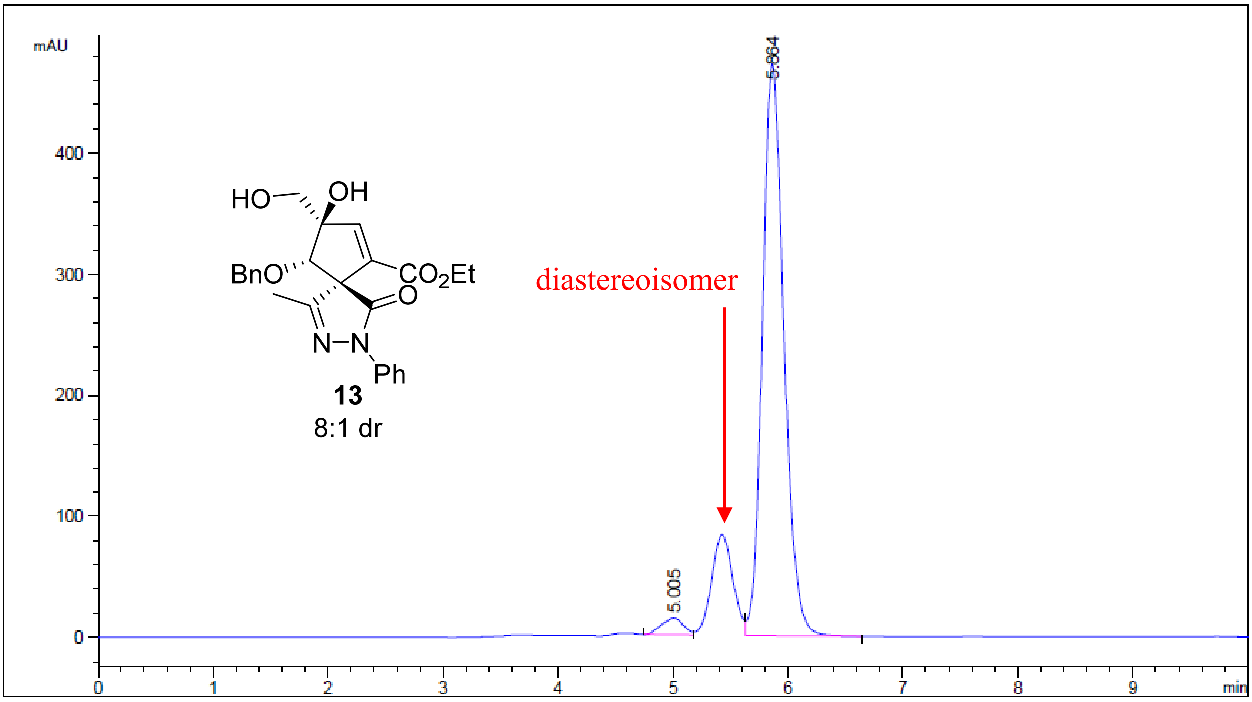

Peak #	RetTime [min]	Type	Width [min]	Area mAU *s	Height [mAU]	Area %
1	5.005	BV	0.2036	190.88573	14.18565	3.0186
2	5.864	VB	0.1995	6132.81006	472.27249	96.9814

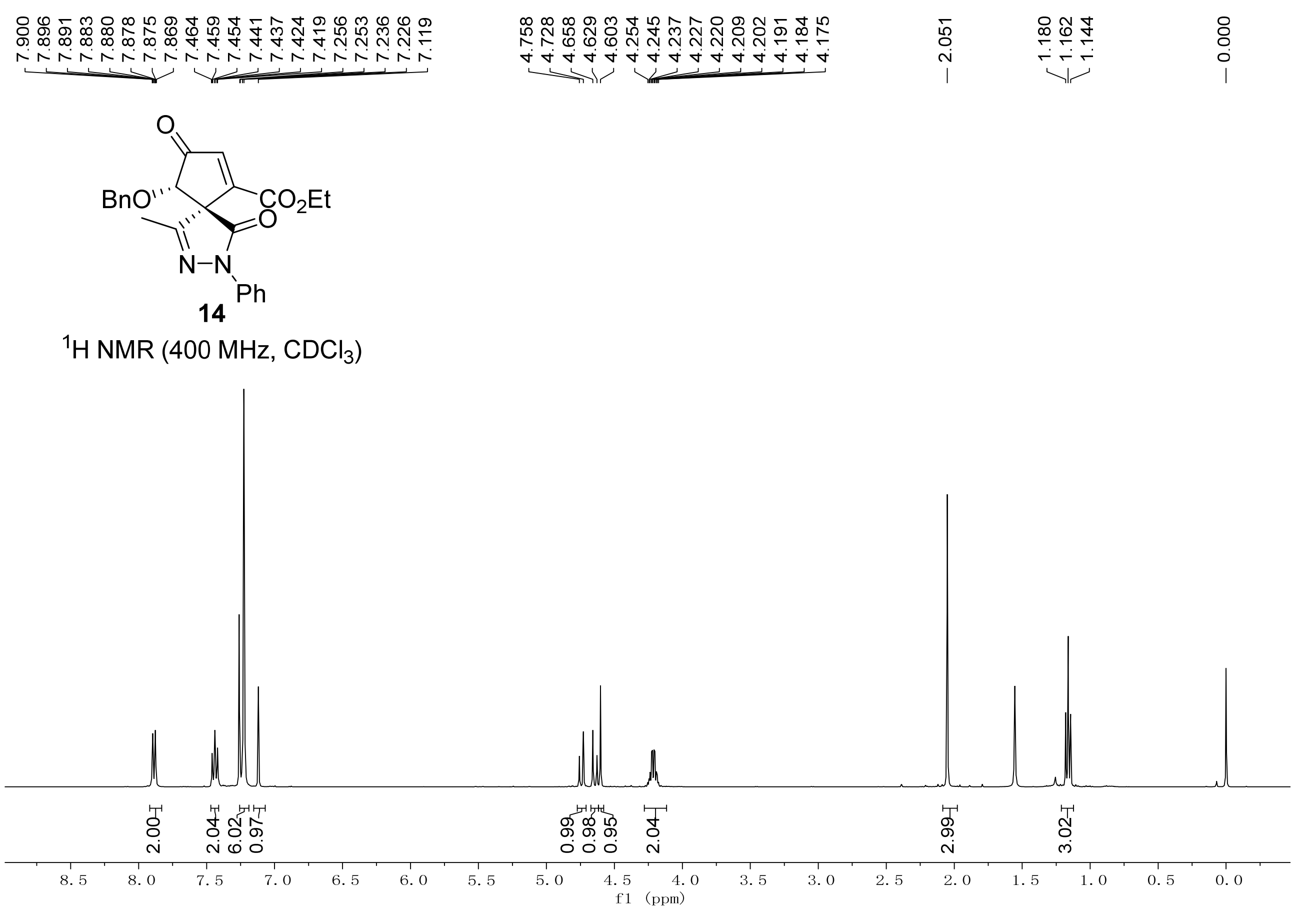

14

^{1}H NMR (400 MHz, $CDCl_3$)

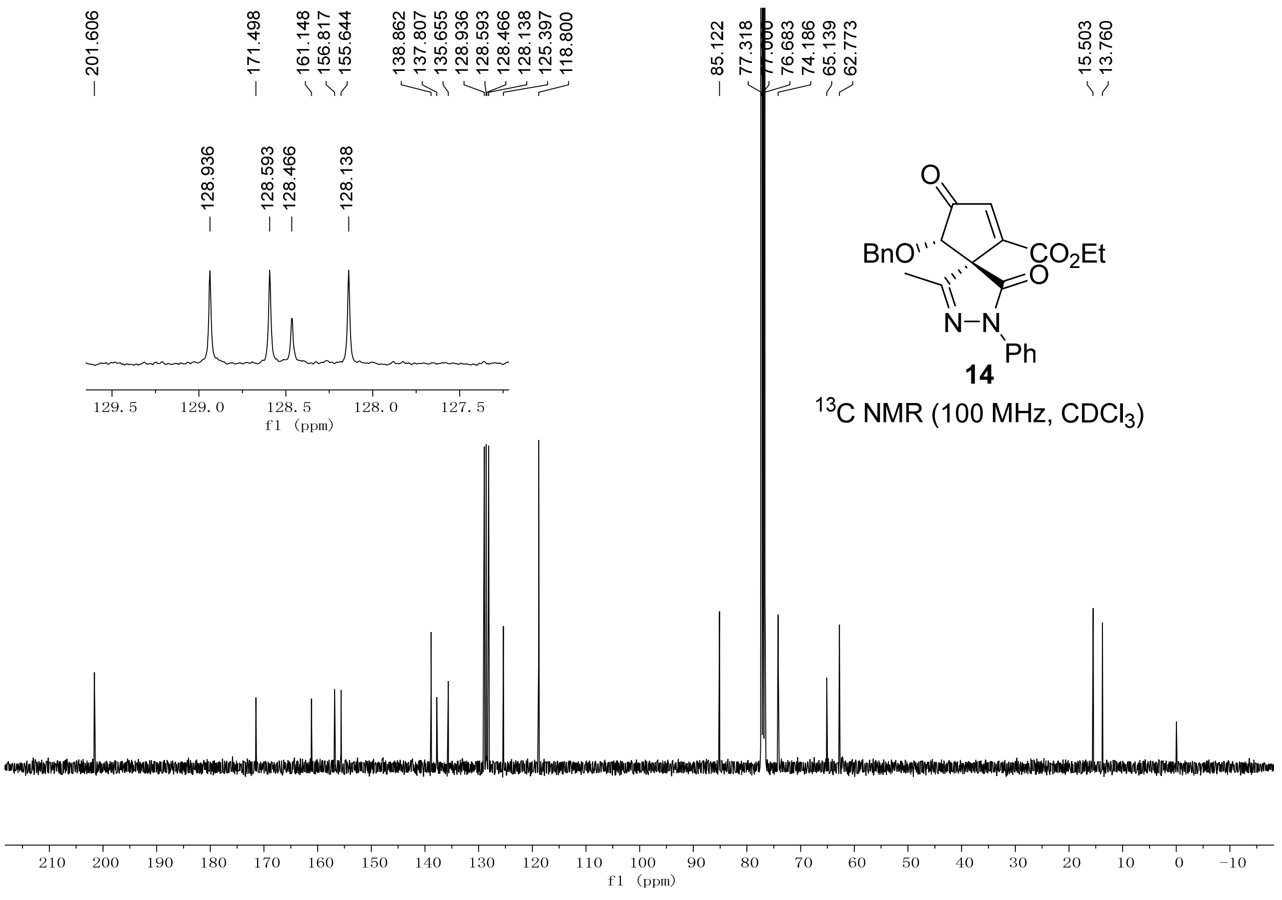

14

^{13}C NMR (100 MHz, $CDCl_3$)

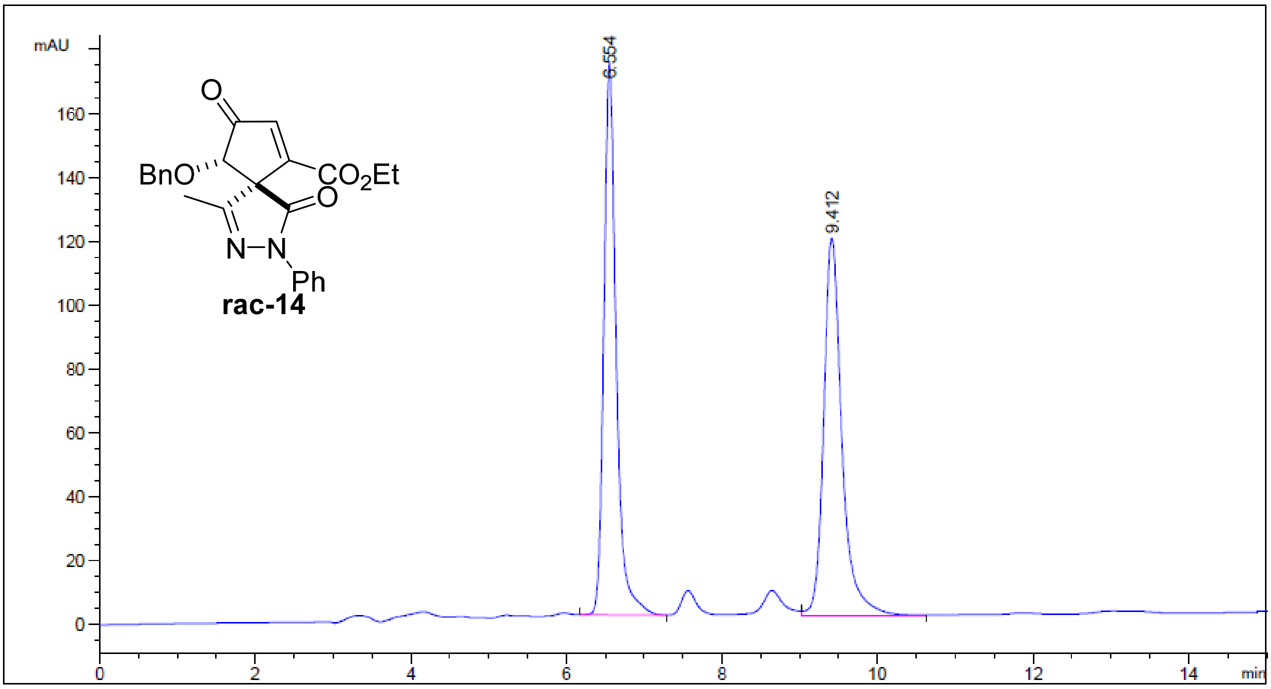

Peak #	RetTime [min]	Type	Width [min]	Area mAU *s	Height [mAU]	Area %
1	6.554	BB	0.1686	1914.03088	172.66345	50.0134
2	9.412	VB	0.2430	1913.00598	117.99076	49.9866

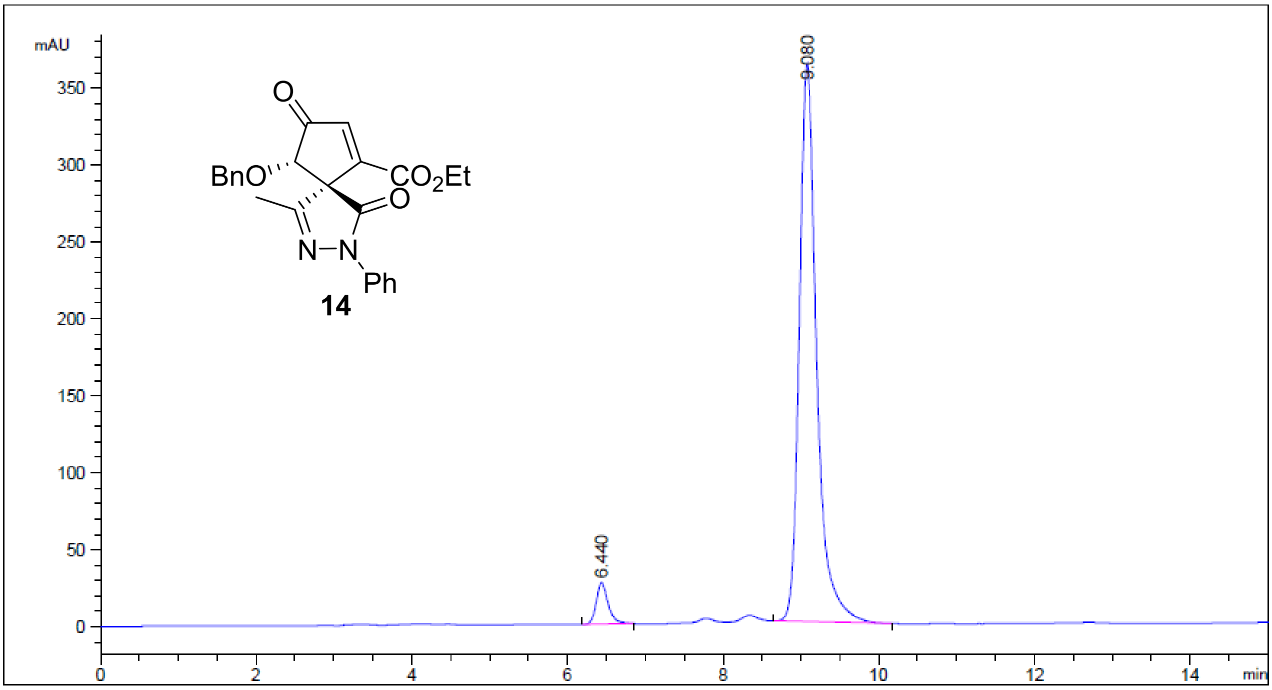

Peak #	RetTime [min]	Type	Width [min]	Area mAU *s	Height [mAU]	Area %
1	6.440	BBA	0.1609	285.36685	27.04737	4.8450
2	9.080	BB	0.2328	5604.60986	362.55560	95.1550

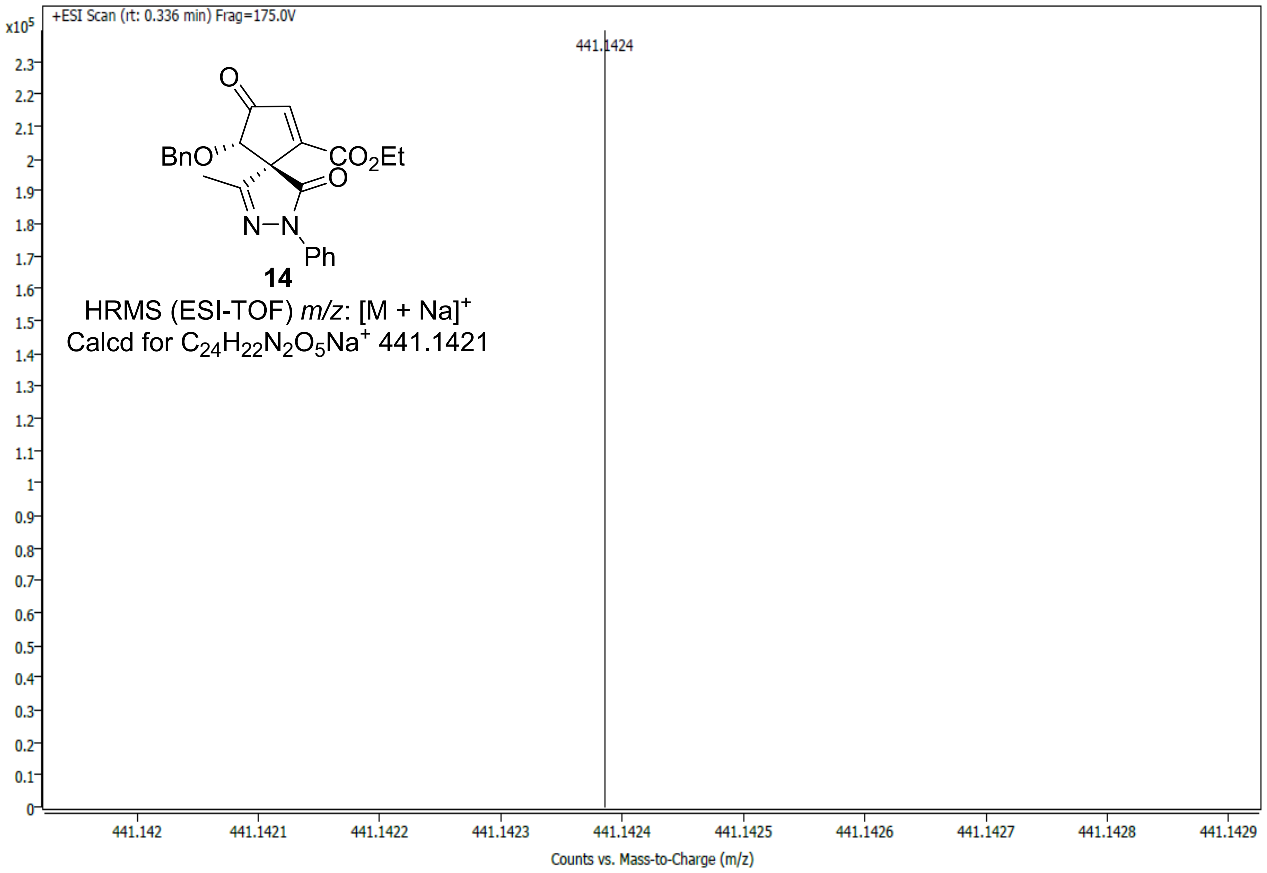
+ESI Scan (rt: 0.336 min) Frag=175.0V
x10^5
441.1424
BnO
CO2Et
O
N
N
Ph
14
HRMS (ESI-TOF) m/z: [M + Na]+
Calcd for C24H22N2O5Na+ 441.1421
Counts vs. Mass-to-Charge (m/z)

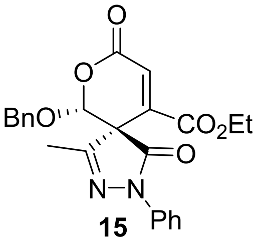

15

^{1}H NMR(400 MHz, $CDCl_3$)

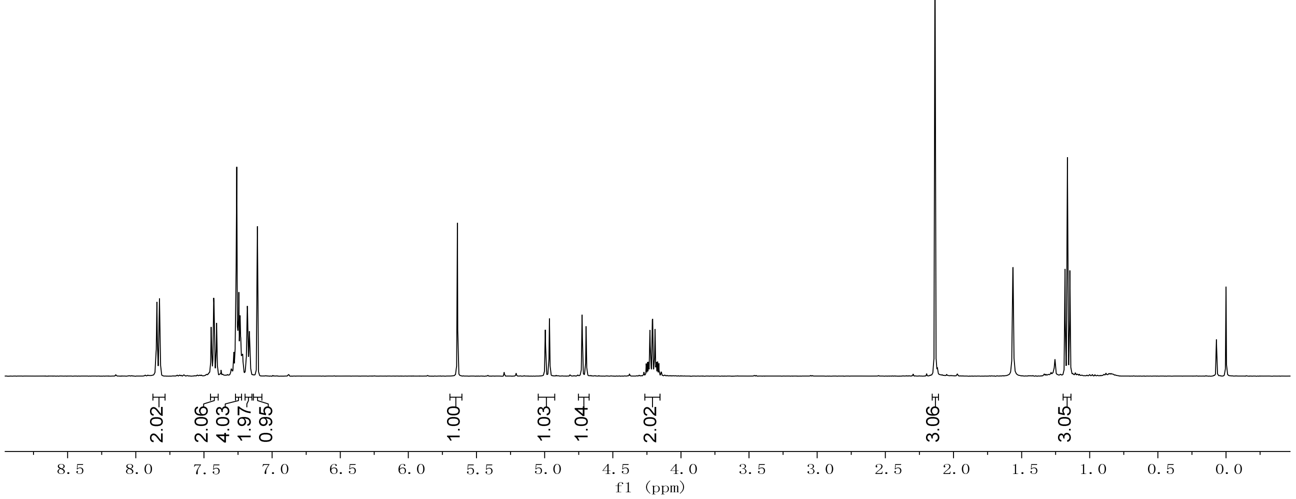

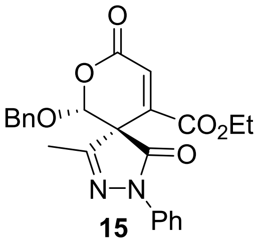

15

^{13}C NMR(100 MHz, $CDCl_3$)

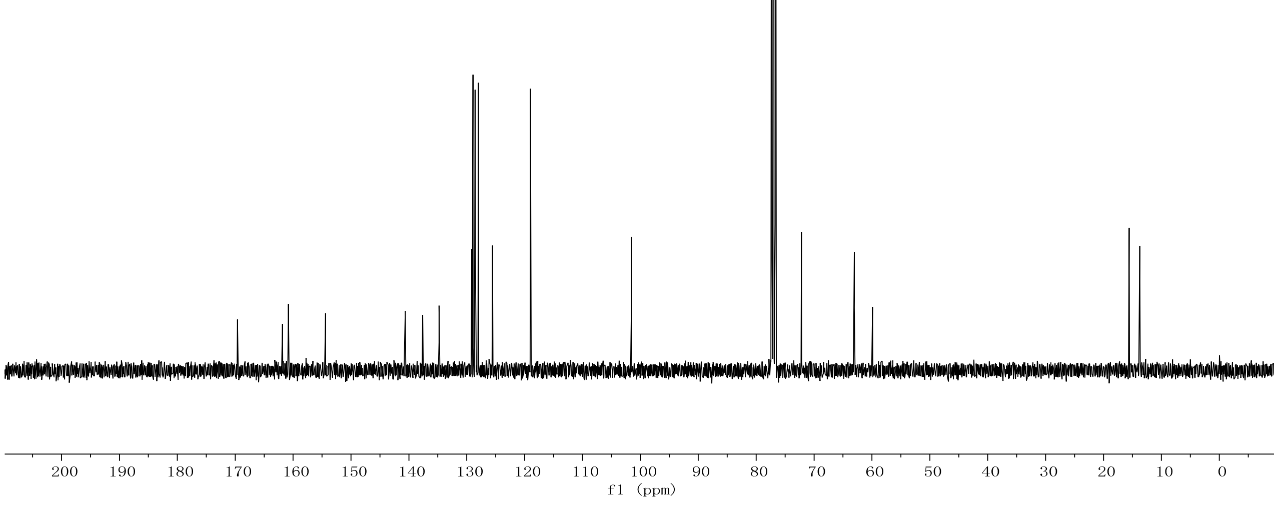

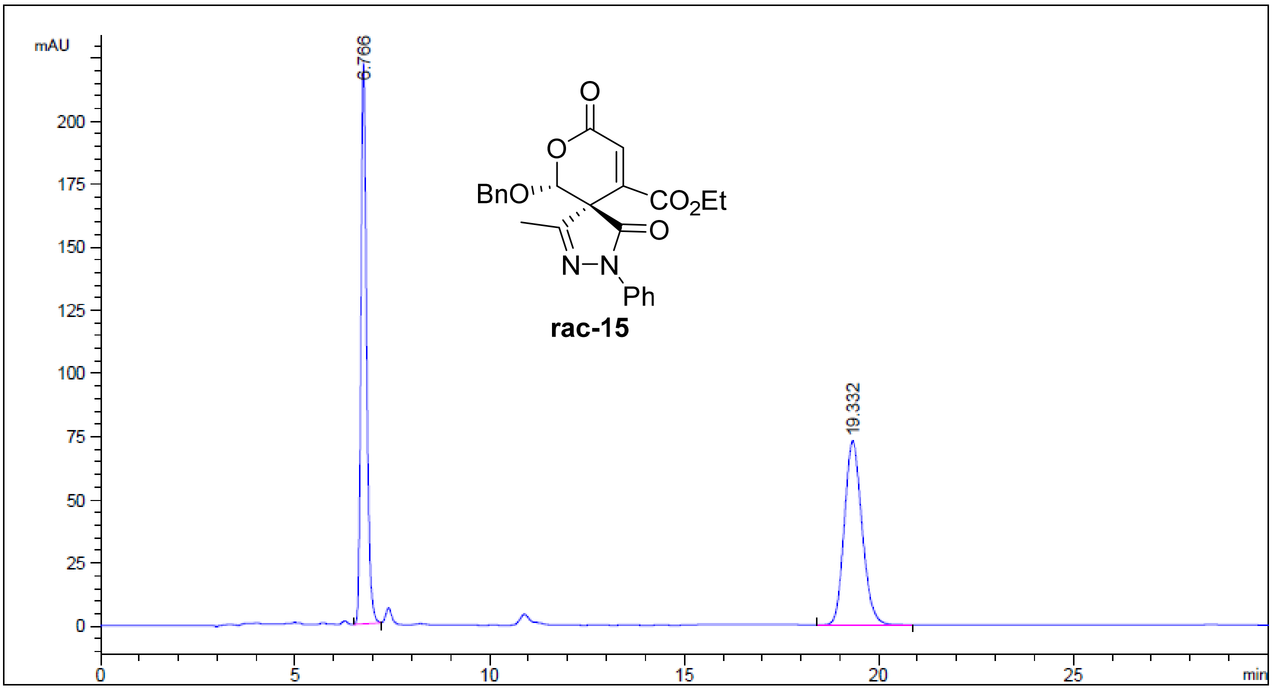

Peak #	RetTime [min]	Type	Width [min]	Area mAU *s	Height [mAU]	Area %
1	6.766	BB	0.1578	2278.64526	221.64510	49.6248
2	19.332	BB	0.4898	2313.09839	72.84966	50.3752

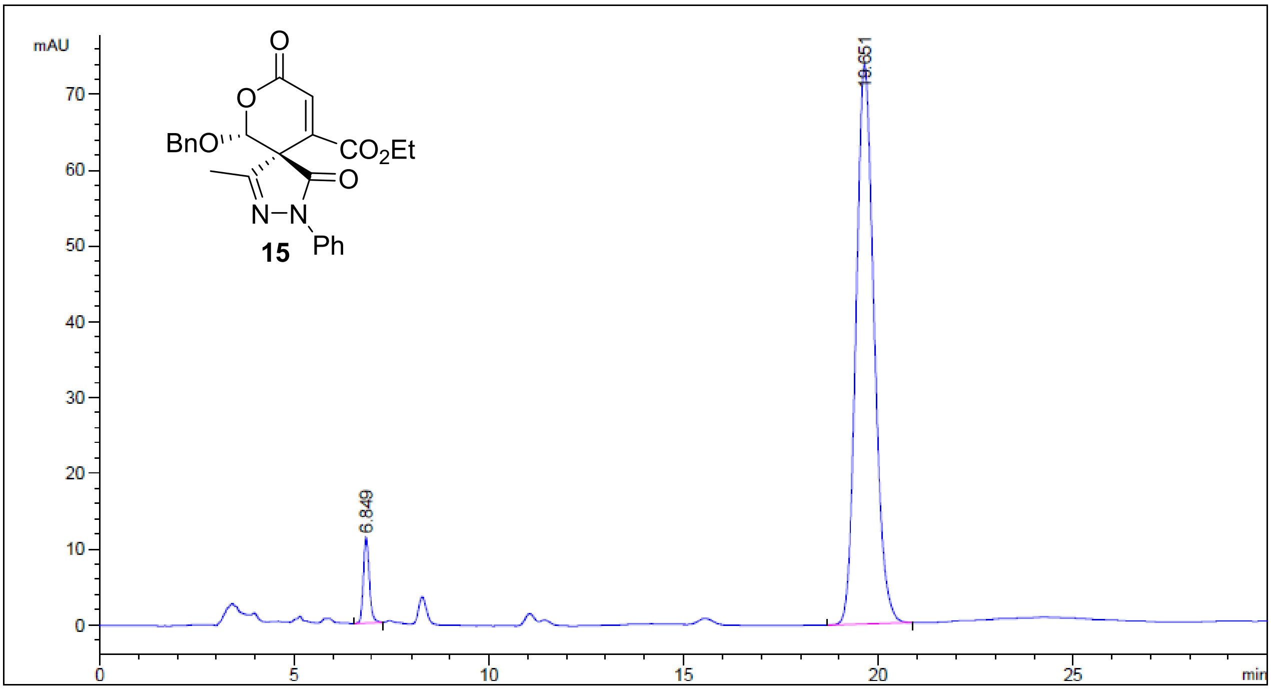

Peak #	RetTime [min]	Type	Width [min]	Area mAU *s	Height [mAU]	Area %
1	6.849	BB	0.1609	119.10837	11.29684	4.7597
2	19.651	BB	0.4982	2383.34473	73.96580	95.2403

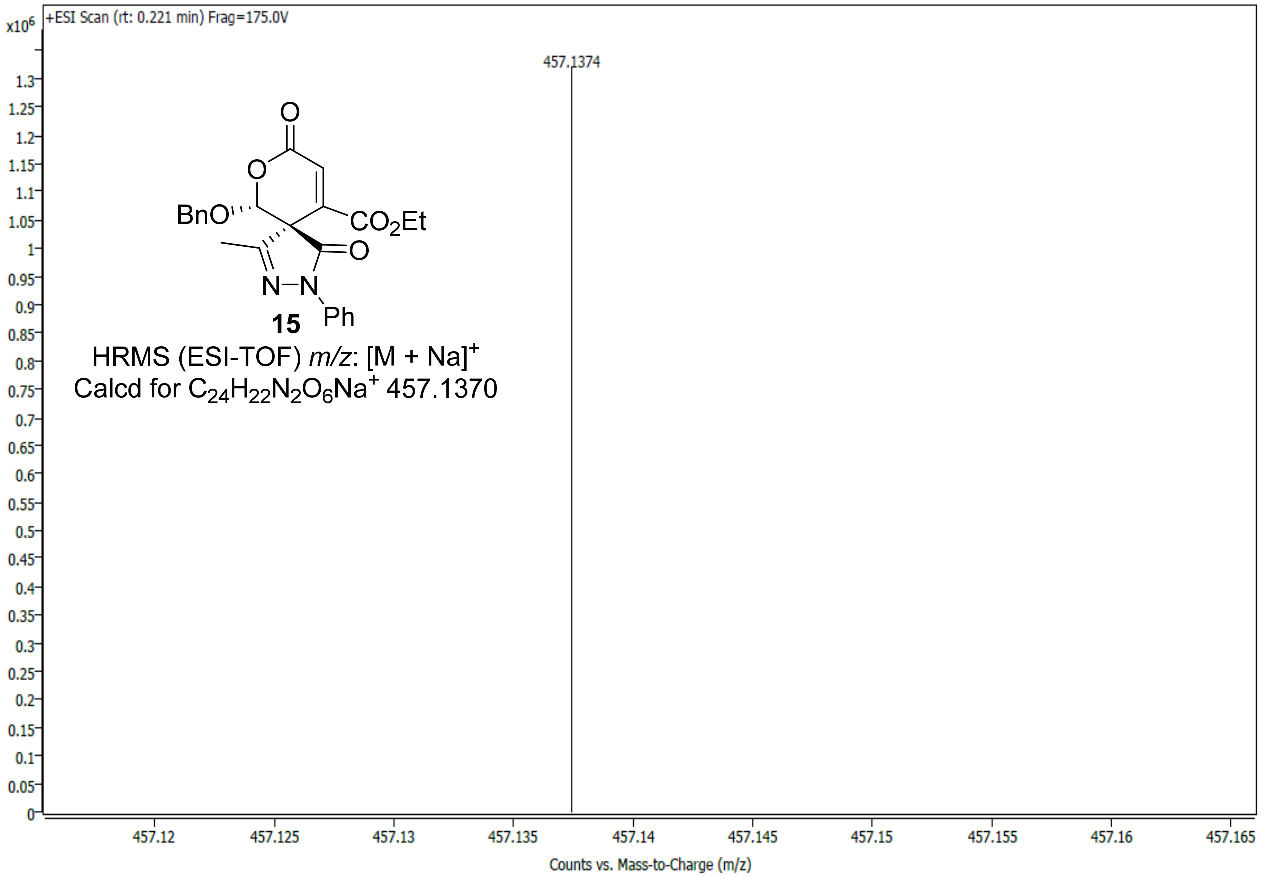

+ESI Scan (rt: 0.221 min) Frag=175.0V
x10^6
457.1374
BnO
CO2Et
N–N
Ph
15
HRMS (ESI-TOF) m/z: [M + Na]+
Calcd for C24H22N2O6Na+ 457.1370
Counts vs. Mass-to-Charge (m/z)

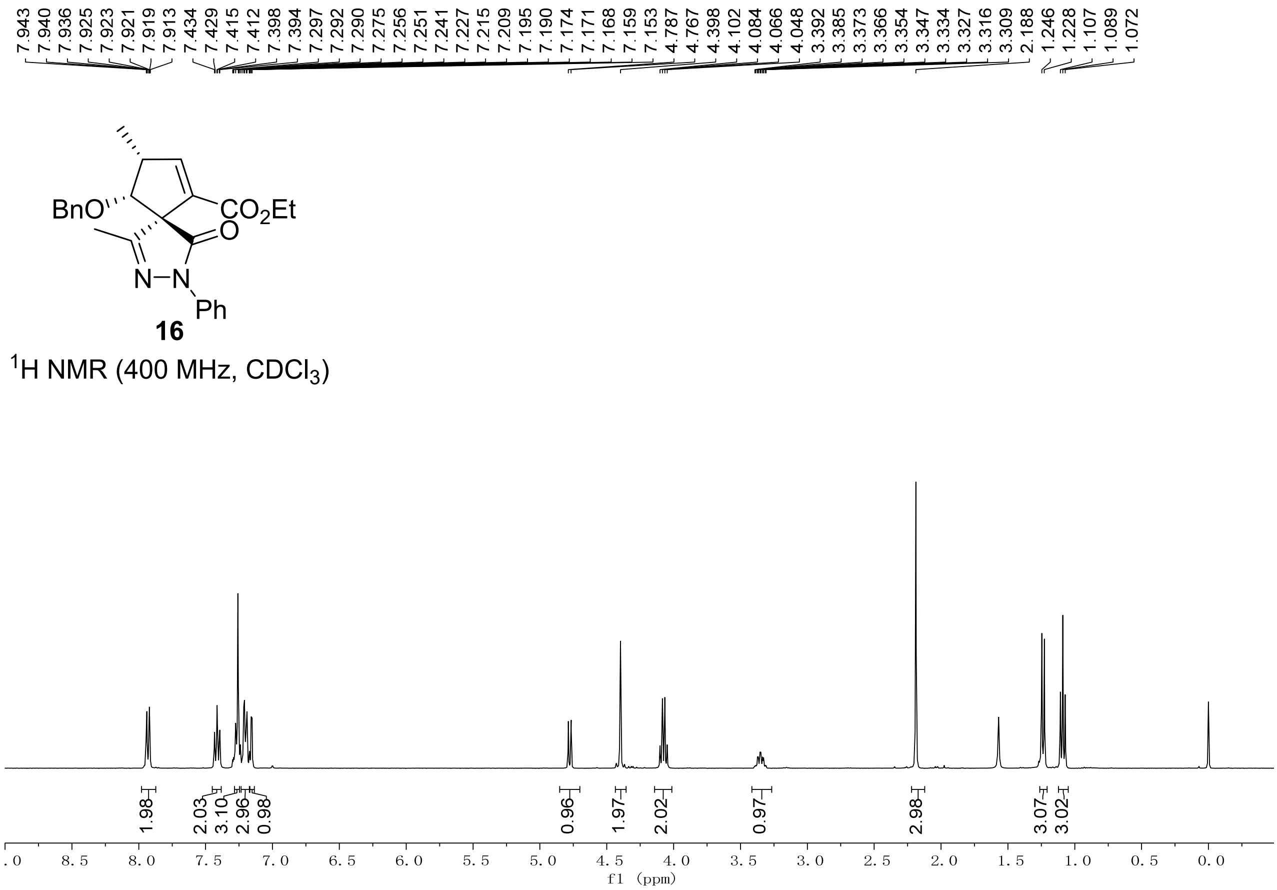

16

^{1}H NMR (400 MHz, $CDCl_3$)

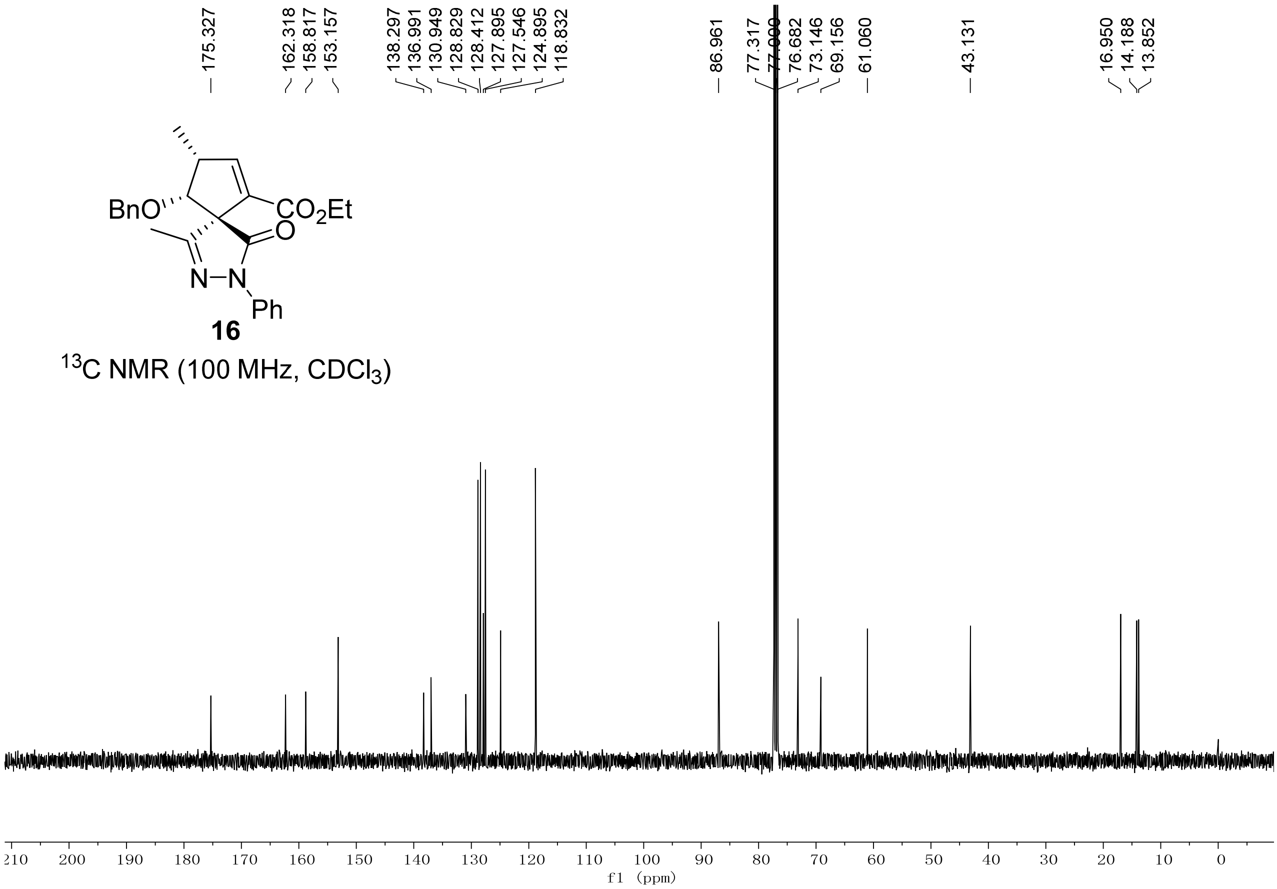

16

^{13}C NMR (100 MHz, $CDCl_3$)

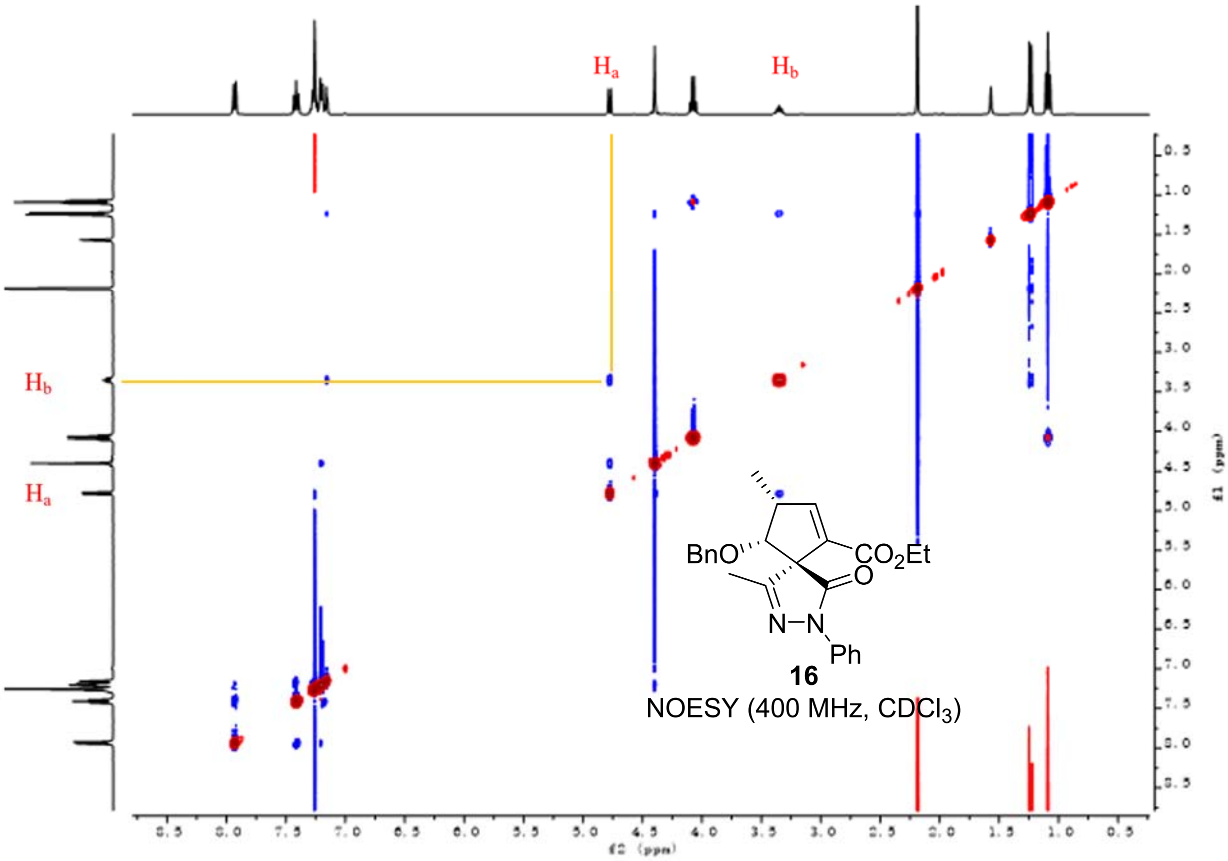

Ha
Hb
BnO
CO2Et
O
N–N
Ph
16
NOESY (400 MHz, CDCl3)
f2 (ppm)
f1 (ppm)

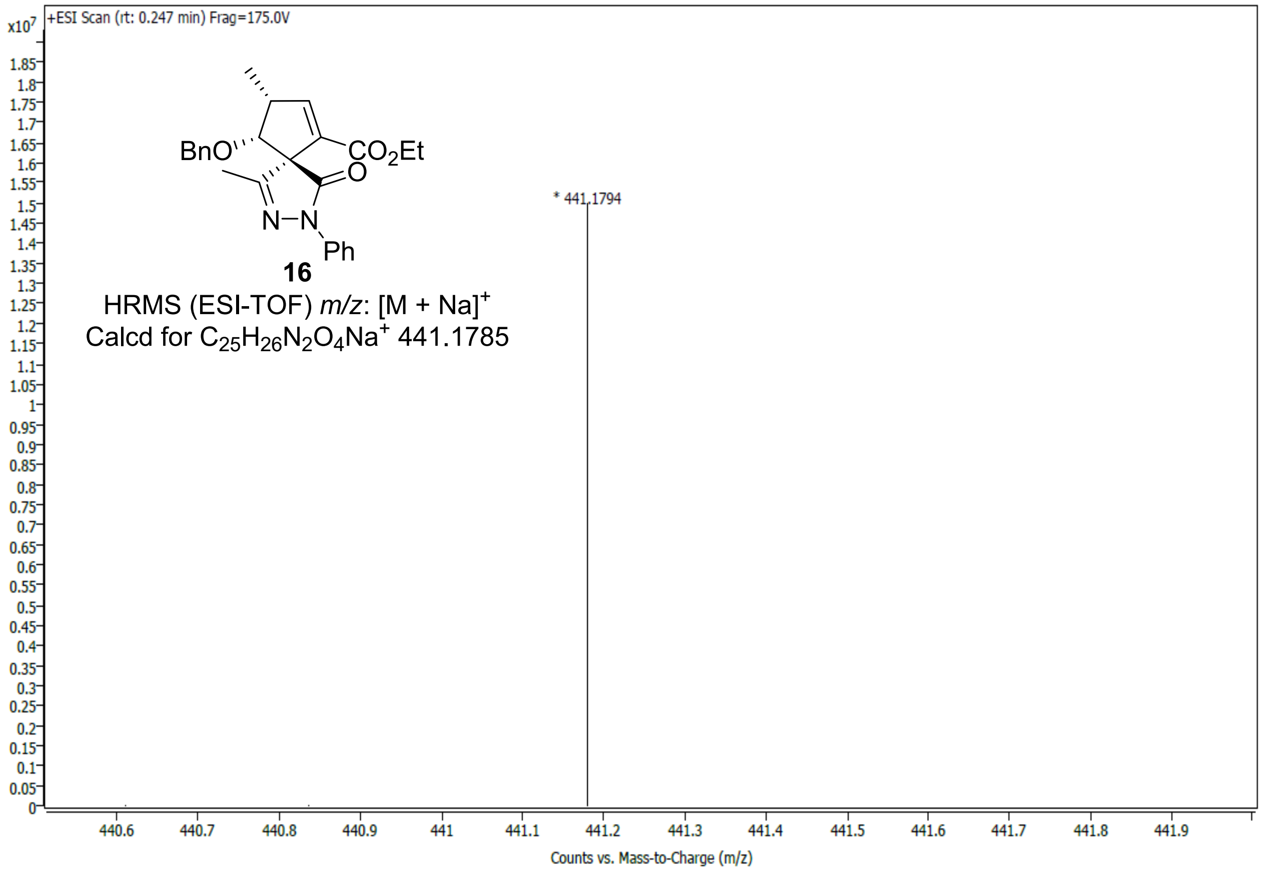

x10^7
+ESI Scan (rt: 0.247 min) Frag=175.0V
BnO
CO2Et
O
N–N
Ph
16
HRMS (ESI-TOF) m/z: [M + Na]+
Calcd for C25H26N2O4Na+ 441.1785
* 441.1794
Counts vs. Mass-to-Charge (m/z)

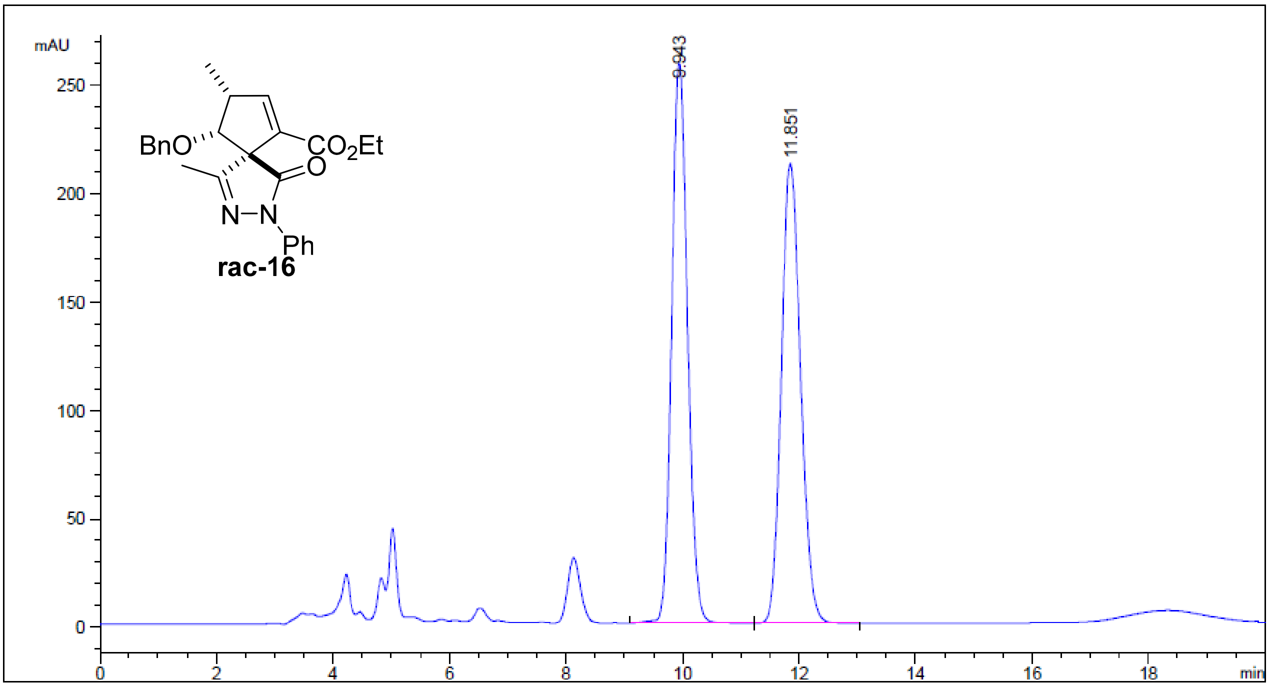

Peak #	RetTime [min]	Type	Width [min]	Area mAU *s	Height [mAU]	Area %
1	9.943	BB	0.2956	4902.07520	258.36795	50.1001
2	11.851	BB	0.3590	4882.49365	212.00949	49.8999

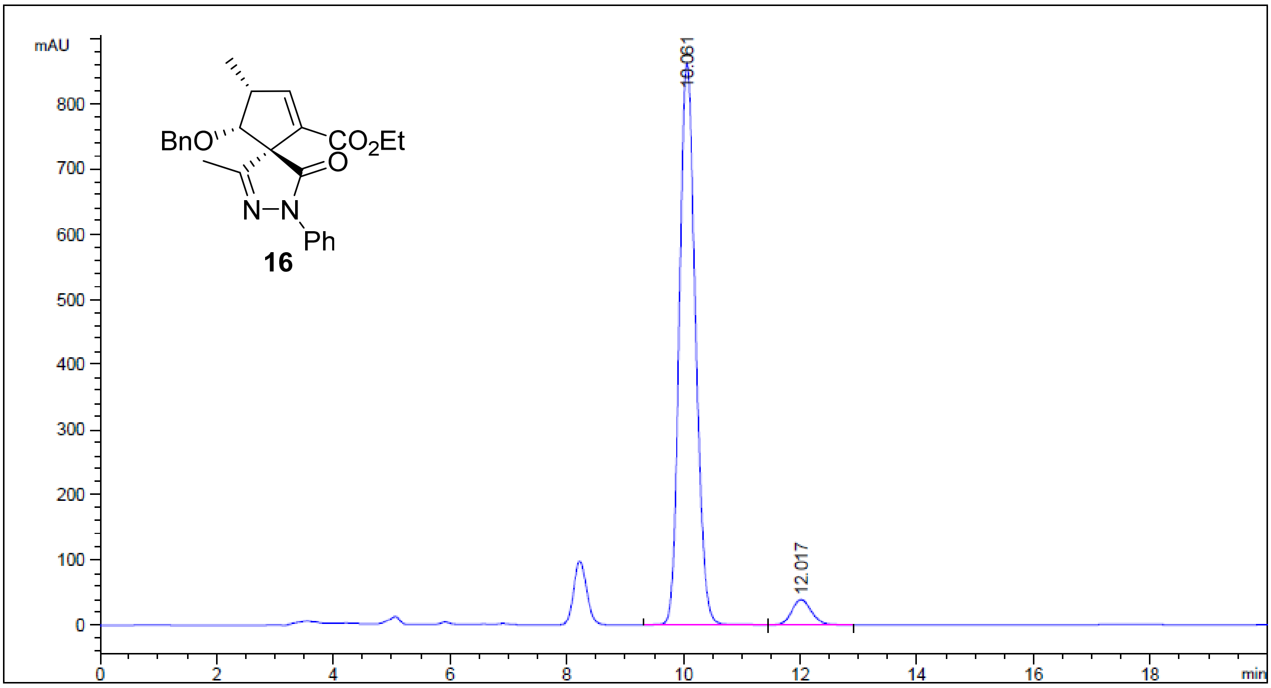

Peak #	RetTime [min]	Type	Width [min]	Area mAU *s	Height [mAU]	Area %
1	10.061	BB	0.3004	1.65607e4	859.92310	94.8147
2	12.017	BB	0.3666	905.67859	38.64970	5.1853

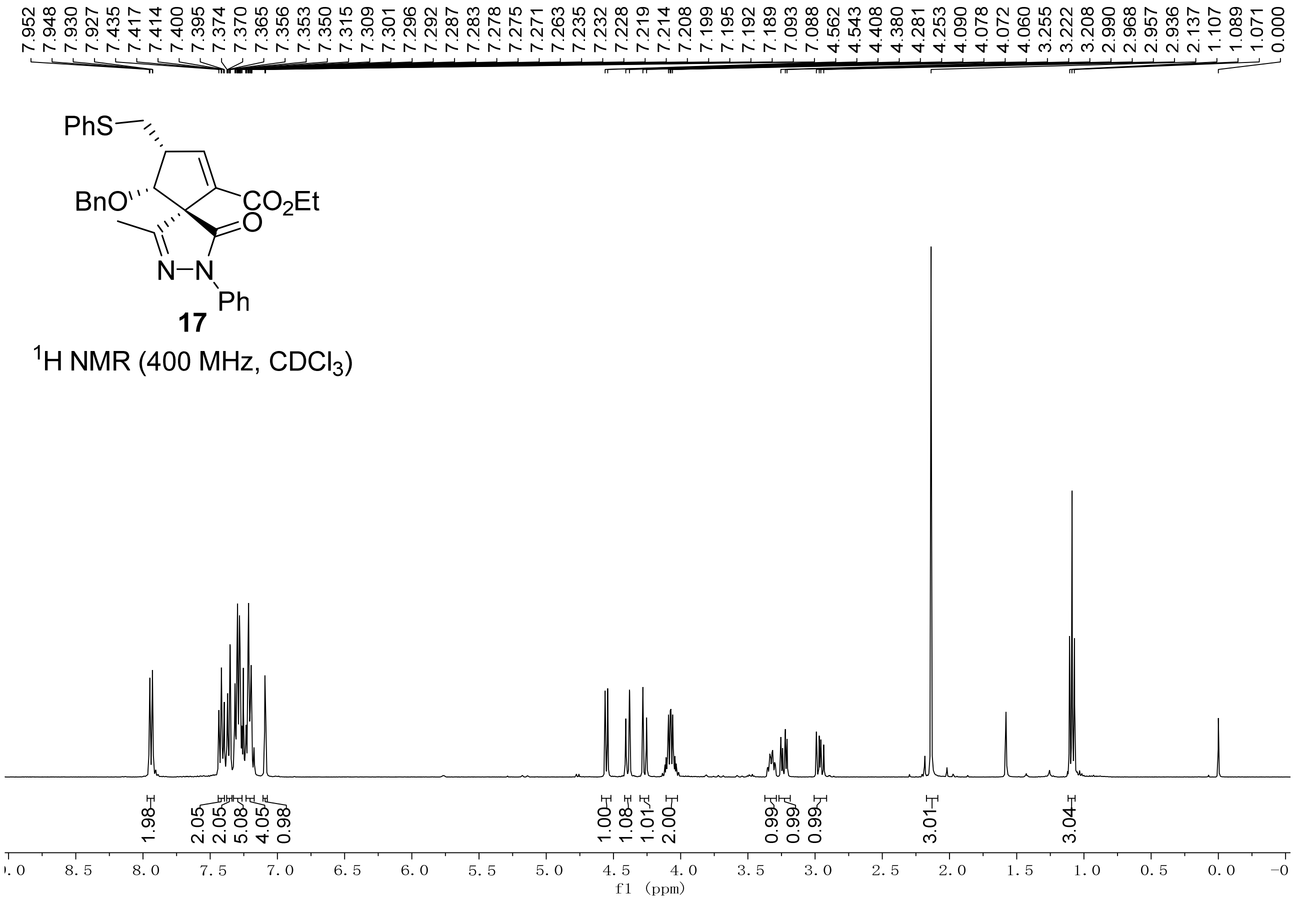
7.952 7.948 7.930 7.927 7.435 7.417 7.414 7.400 7.395 7.374 7.370 7.365 7.356 7.353 7.350 7.315 7.309 7.301 7.296 7.292 7.287 7.283 7.278 7.275 7.271 7.263 7.235 7.232 7.228 7.219 7.214 7.208 7.199 7.195 7.192 7.189 7.093 7.088 4.562 4.543 4.408 4.380 4.281 4.253 4.090 4.078 4.072 4.060 3.255 3.222 3.208 2.990 2.968 2.957 2.936 2.137 1.107 1.089 1.071 0.000
PhS
BnO
CO2Et
O
N–N
Ph
17
1H NMR (400 MHz, CDCl3)
1.98 2.05 2.05 5.08 4.05 0.98 1.00 1.08 1.01 2.00 0.99 0.99 0.99 3.01 3.04
f1 (ppm)

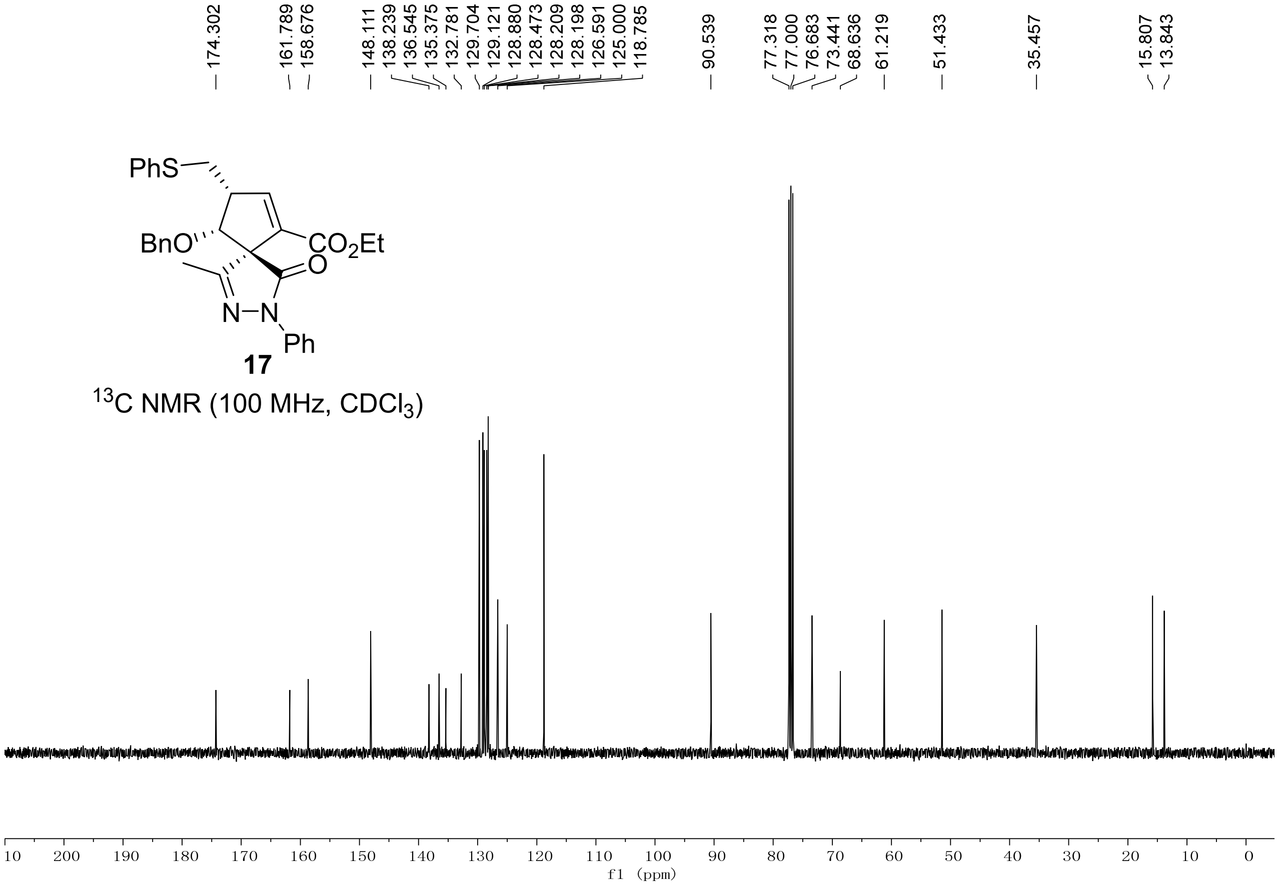
174.302 161.789 158.676 148.111 138.239 136.545 135.375 132.781 129.704 129.121 128.880 128.473 128.209 128.198 126.591 125.000 118.785 90.539 77.318 77.000 76.683 73.441 68.636 61.219 51.433 35.457 15.807 13.843
PhS
BnO
CO2Et
O
N–N
Ph
17
13C NMR (100 MHz, CDCl3)
f1 (ppm)

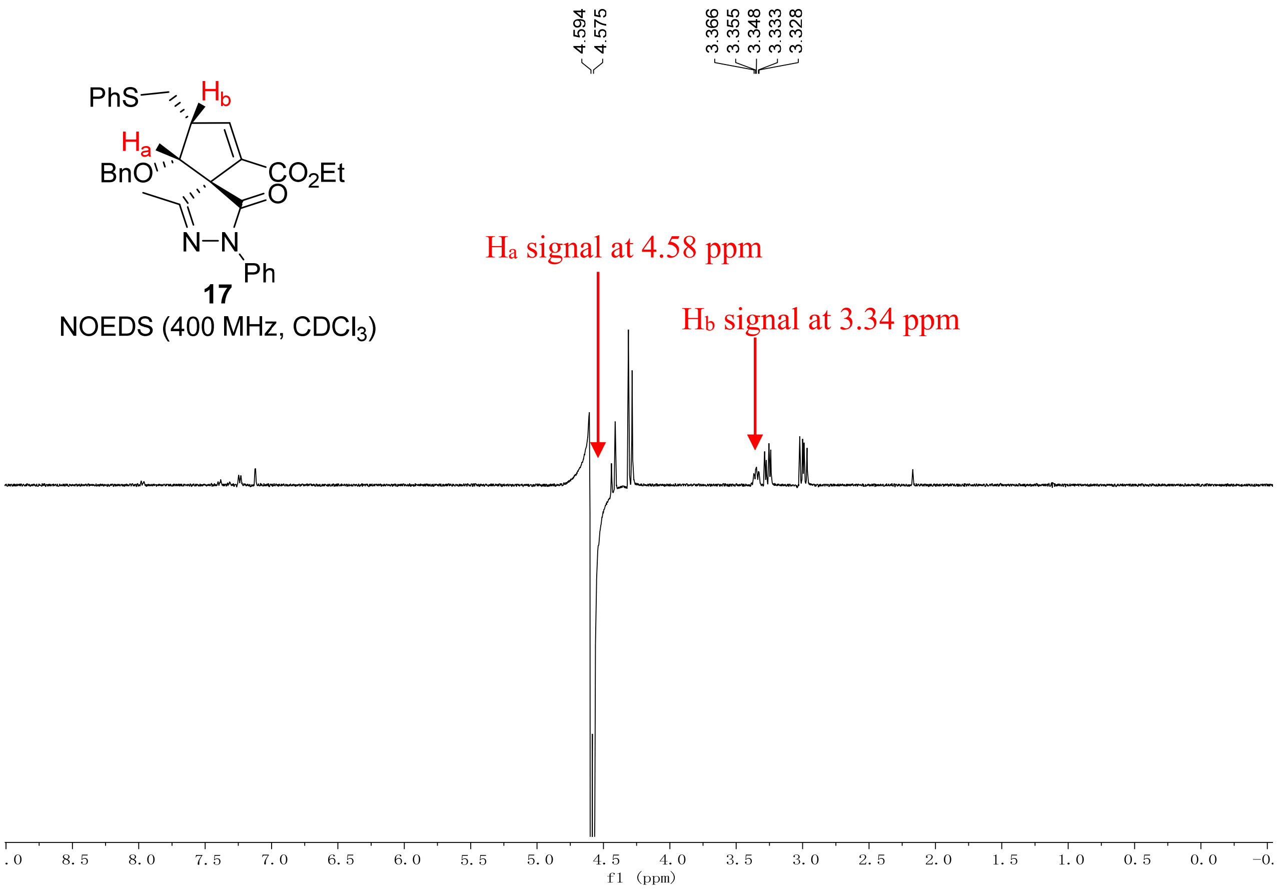

4.594
4.575
3.366
3.355
3.348
3.333
3.328
PhS
Hb
Ha
BnO
CO2Et
O
N–N
Ph
17
NOEDS (400 MHz, CDCl3)
Ha signal at 4.58 ppm
Hb signal at 3.34 ppm
f1 (ppm)

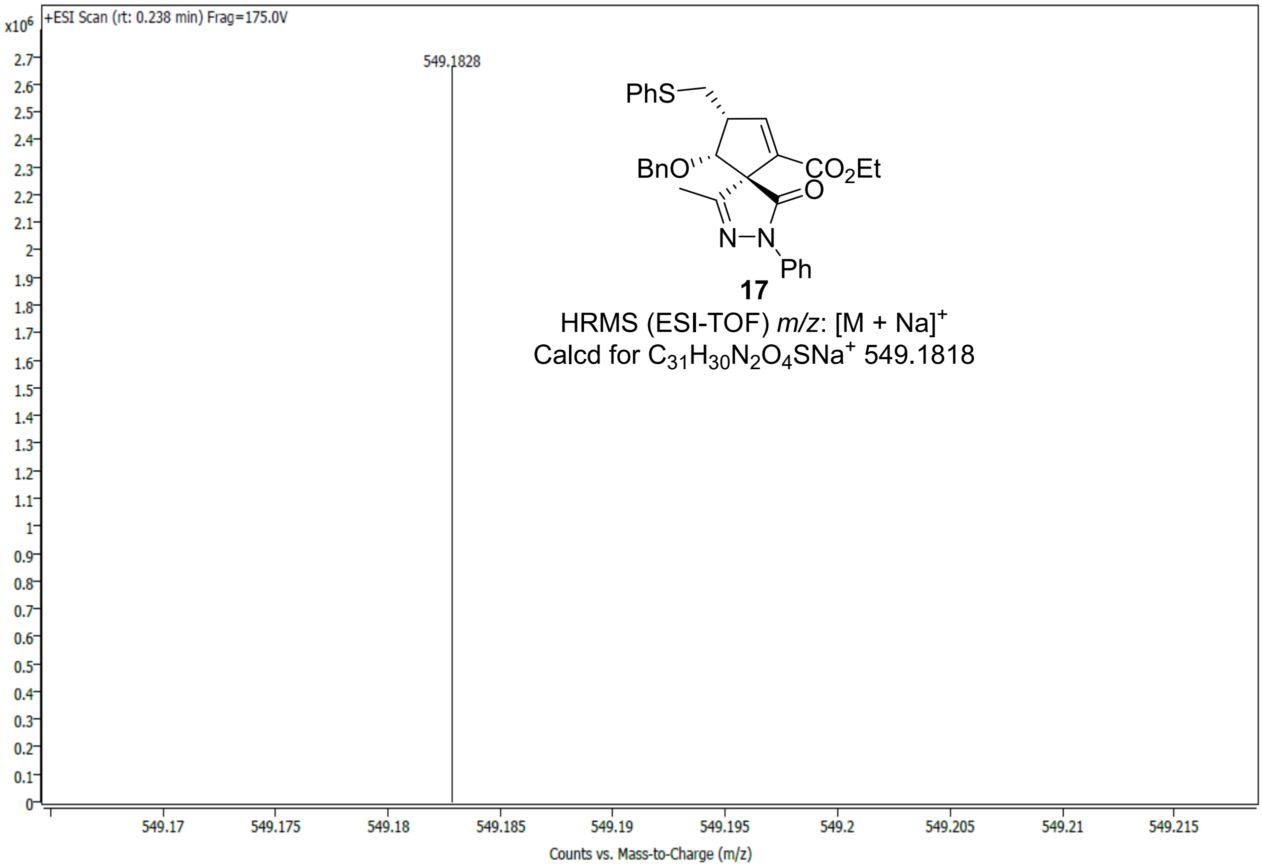

x10^6
+ESI Scan (rt: 0.238 min) Frag=175.0V
549.1828
PhS
BnO
CO2Et
O
N–N
Ph
17
HRMS (ESI-TOF) m/z: [M + Na]+
Calcd for C31H30N2O4SNa+ 549.1818
Counts vs. Mass-to-Charge (m/z)

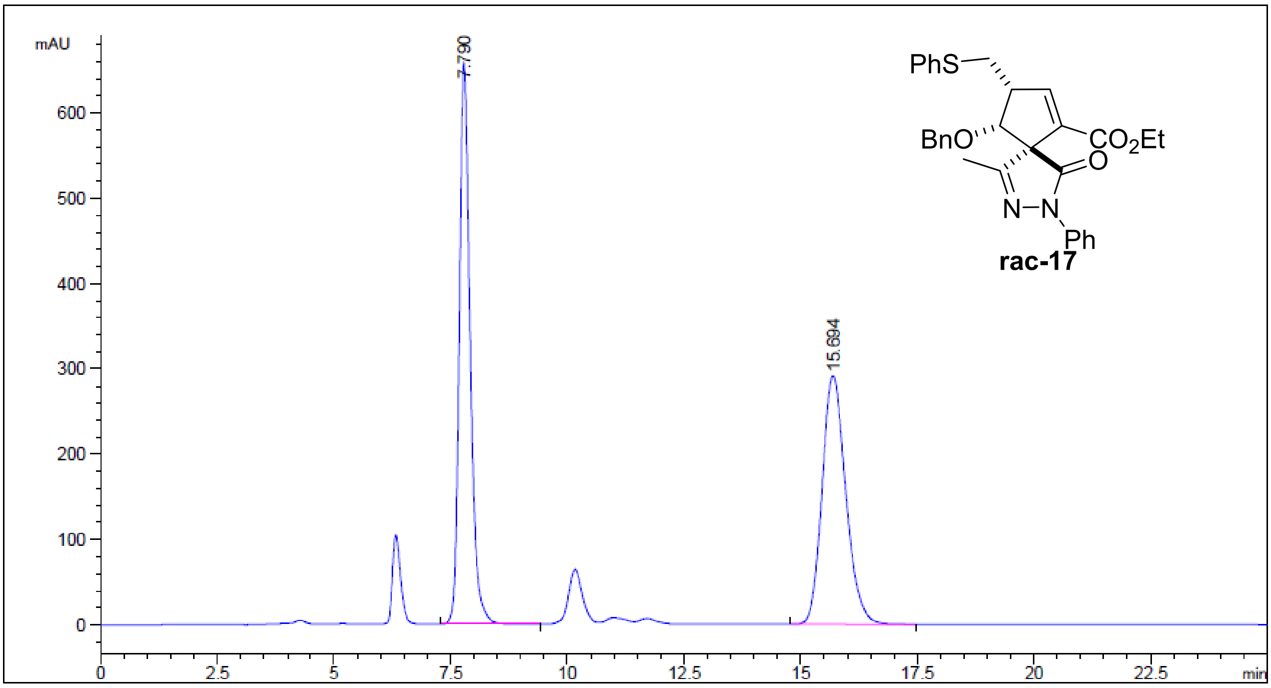

Peak #	RetTime [min]	Type	Width [min]	Area mAU *s	Height [mAU]	Area %
1	7.790	BB	0.2457	1.06463e4	657.32910	50.8508
2	15.694	BB	0.5438	1.02901e4	290.83429	49.1492

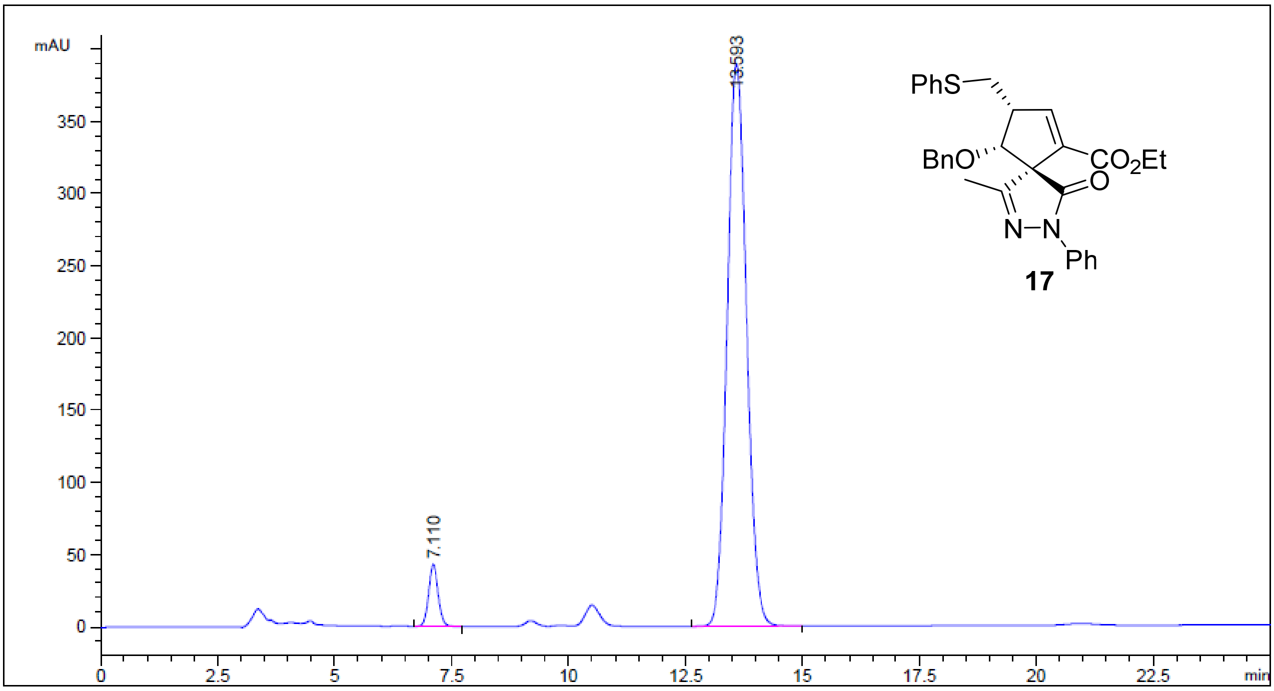

Peak #	RetTime [min]	Type	Width [min]	Area mAU *s	Height [mAU]	Area %
1	7.110	BB	0.2181	604.39679	42.94206	5.1114
2	13.593	BB	0.4498	1.12200e4	389.27594	94.8886

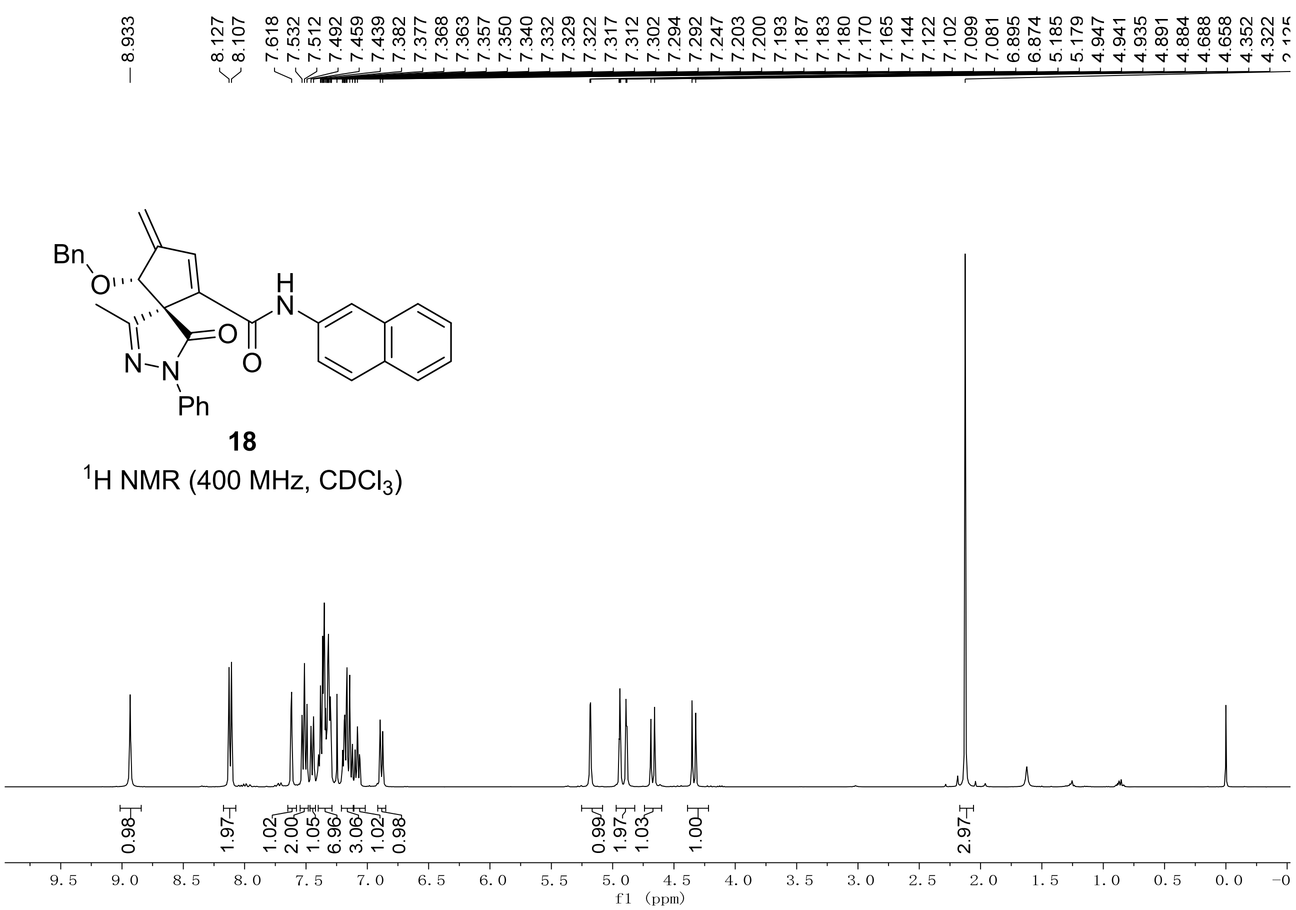

18

^{1}H NMR (400 MHz, CDCl$_3$)

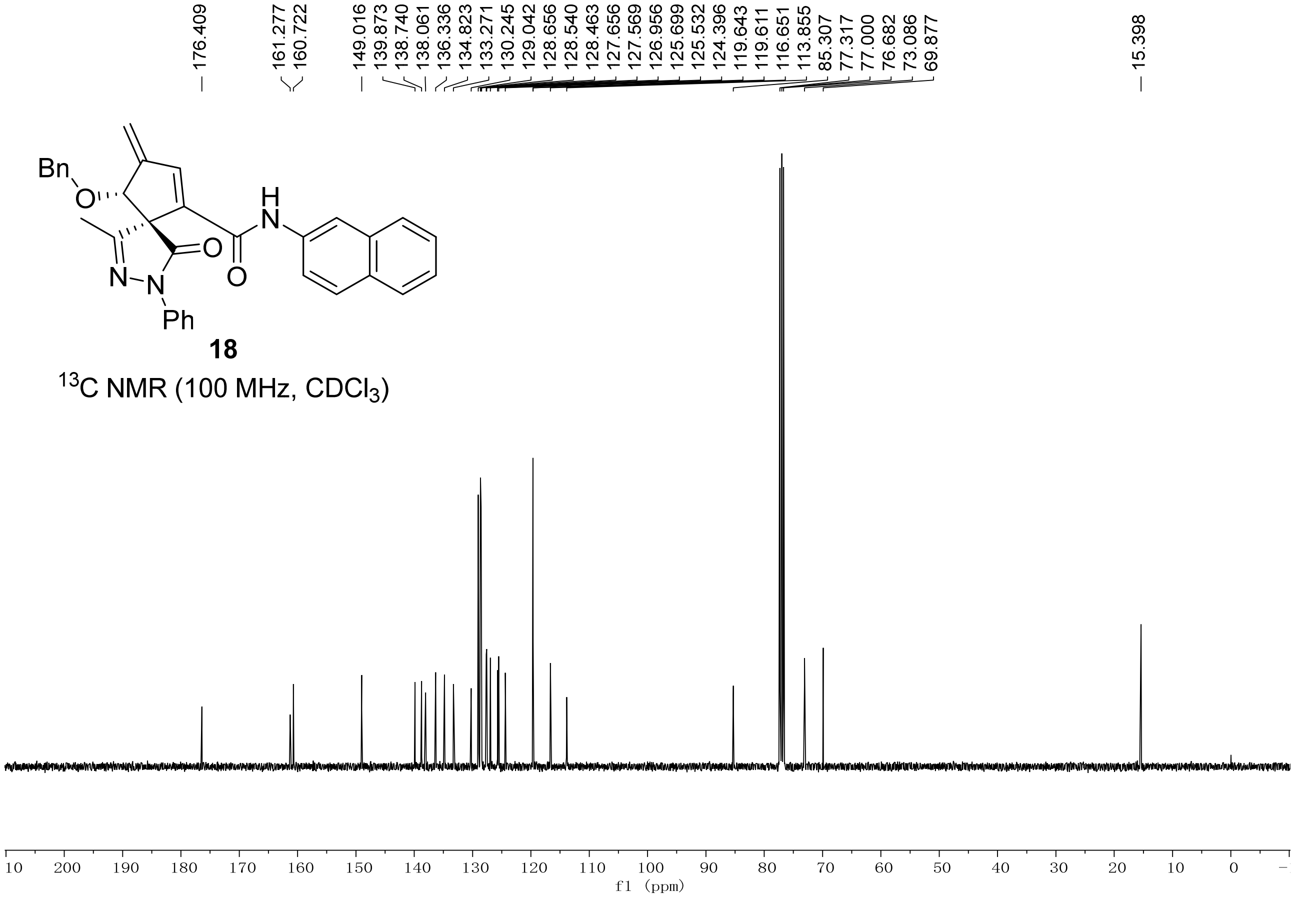

18

^{13}C NMR (100 MHz, CDCl$_3$)

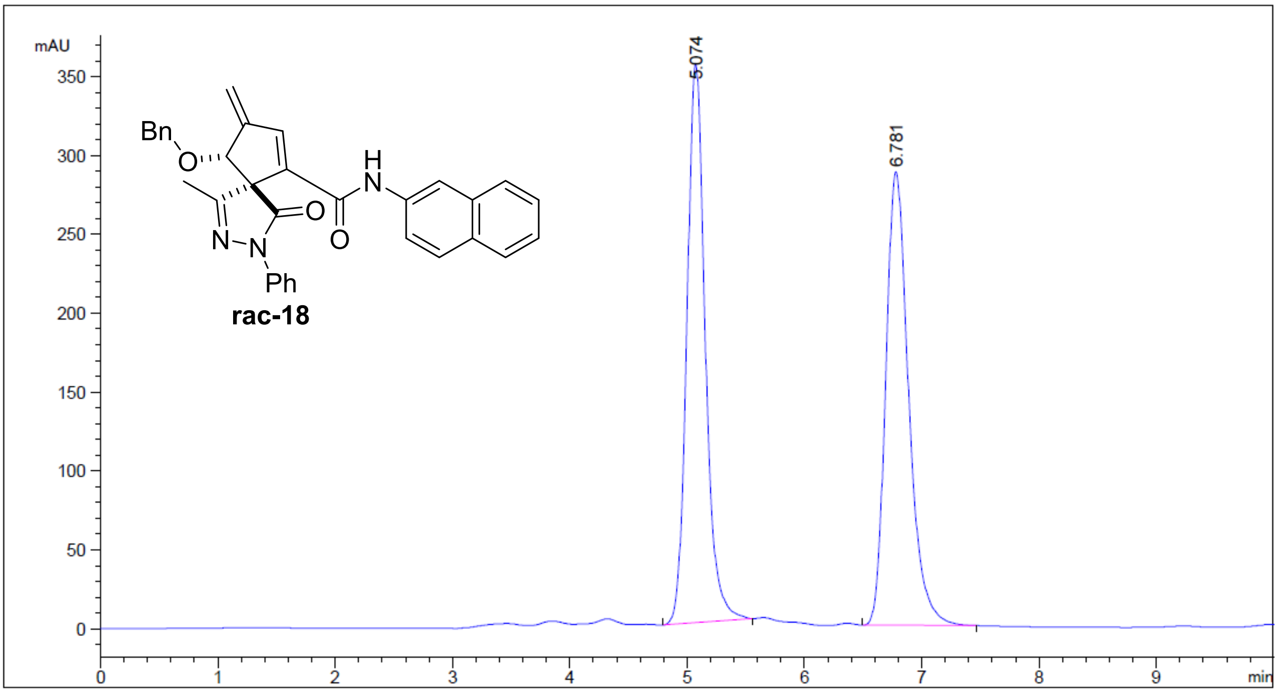

Peak #	RetTime [min]	Type	Width [min]	Area mAU *s	Height [mAU]	Area %
1	5.074	BBA	0.1677	3853.82642	354.15689	49.7426
2	6.781	BBA	0.2074	3893.71606	287.67770	50.2574

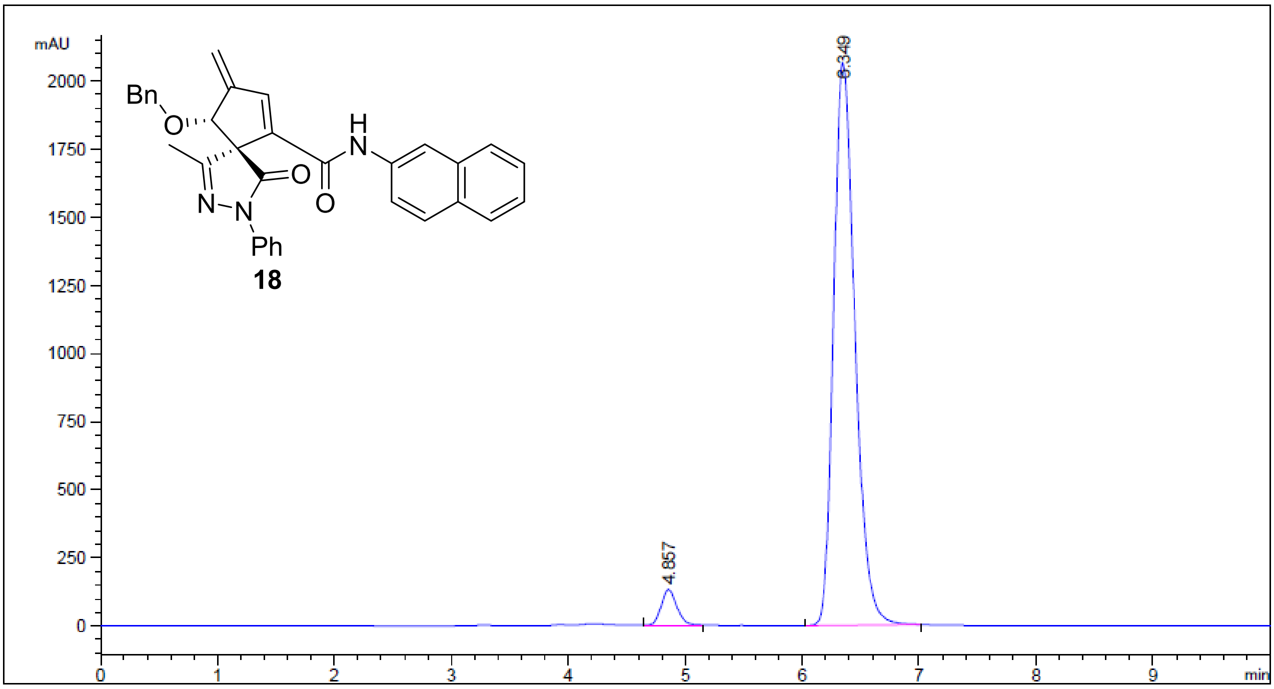

Peak #	RetTime [min]	Type	Width [min]	Area mAU *s	Height [mAU]	Area %
1	4.857	BBA	0.1448	1194.57825	130.27629	4.3555
2	6.349	BBA	0.1980	2.62325e4	2060.50391	95.6445

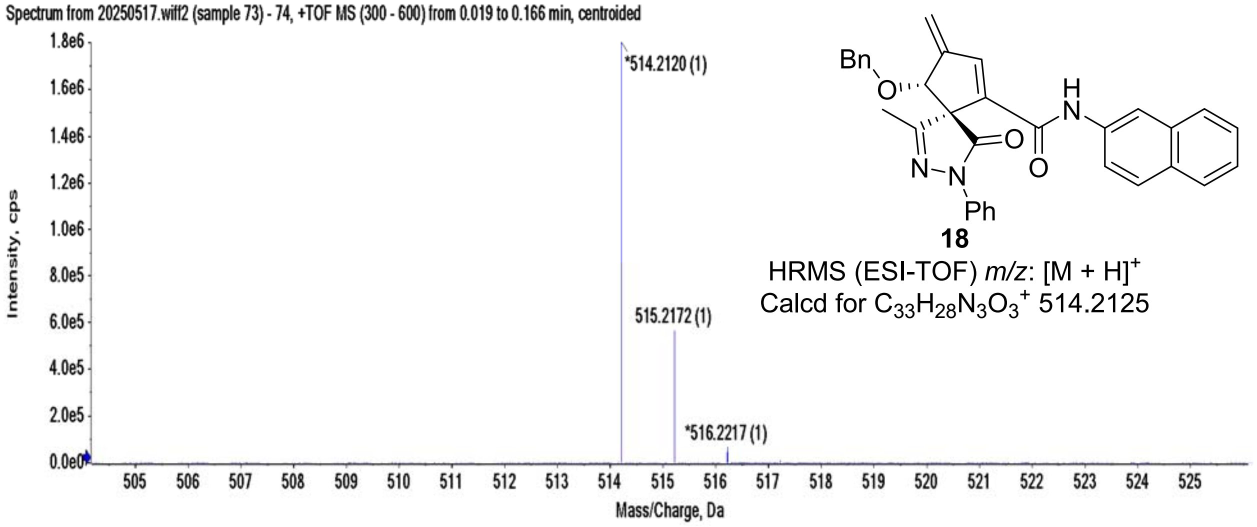

18

HRMS (ESI-TOF) *m/z*: $[M + H]^+$

Calcd for $C_{33}H_{28}N_3O_3^+$ 514.2125

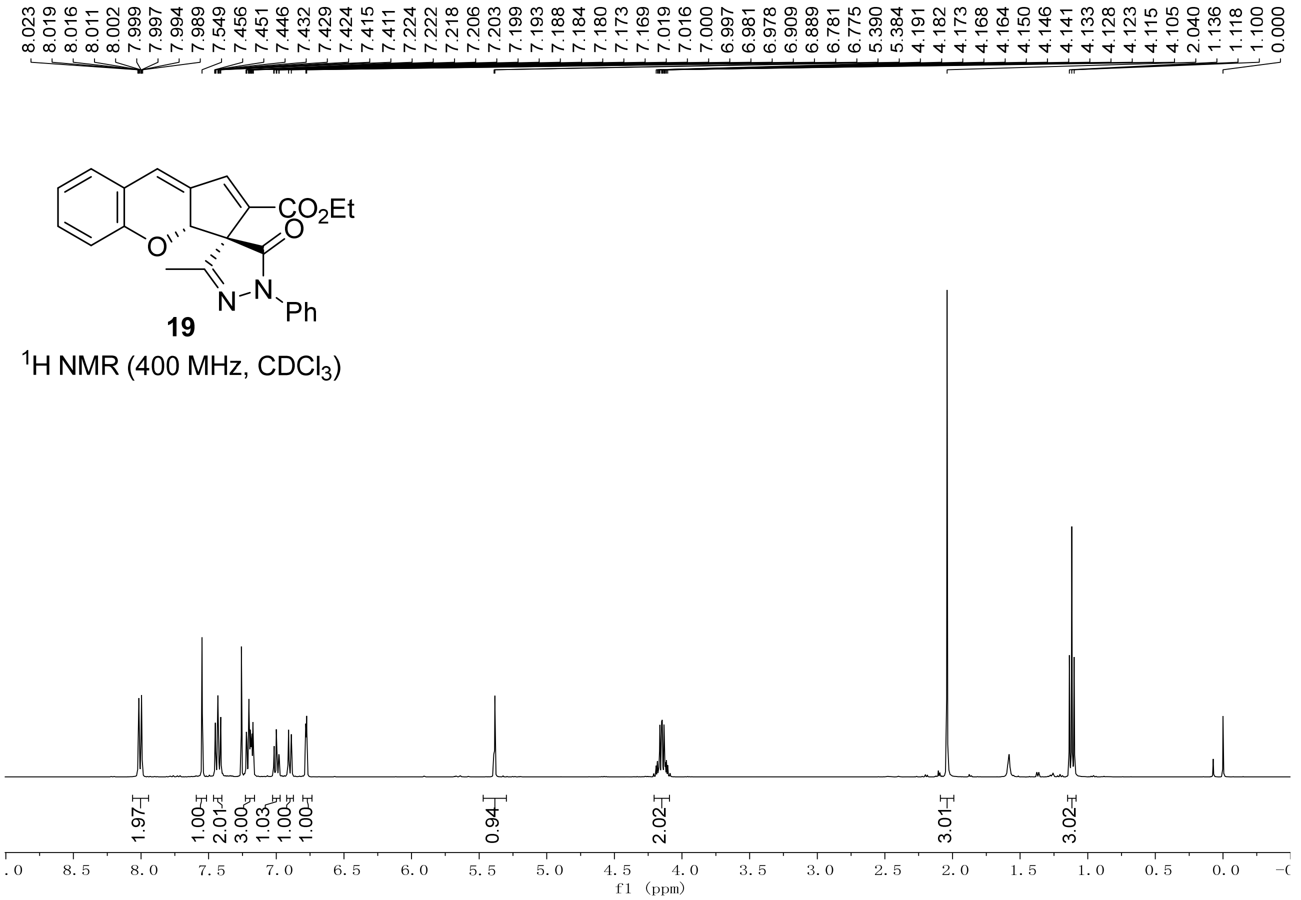
8.023 8.019 8.016 8.011 8.002 7.999 7.997 7.994 7.989 7.549 7.456 7.451 7.446 7.432 7.429 7.424 7.415 7.411 7.224 7.222 7.218 7.206 7.203 7.199 7.193 7.188 7.184 7.180 7.173 7.169 7.019 7.016 7.000 6.997 6.981 6.978 6.909 6.889 6.781 6.775 5.390 5.384 4.191 4.182 4.173 4.168 4.164 4.150 4.146 4.141 4.133 4.128 4.123 4.115 4.105 2.040 1.136 1.118 1.100 0.000
CO2Et
O
O
N
N
Ph
19
1H NMR (400 MHz, CDCl3)
1.97
1.00
2.01
3.00
1.03
1.00
1.00
0.94
2.02
3.01
3.02
f1 (ppm)

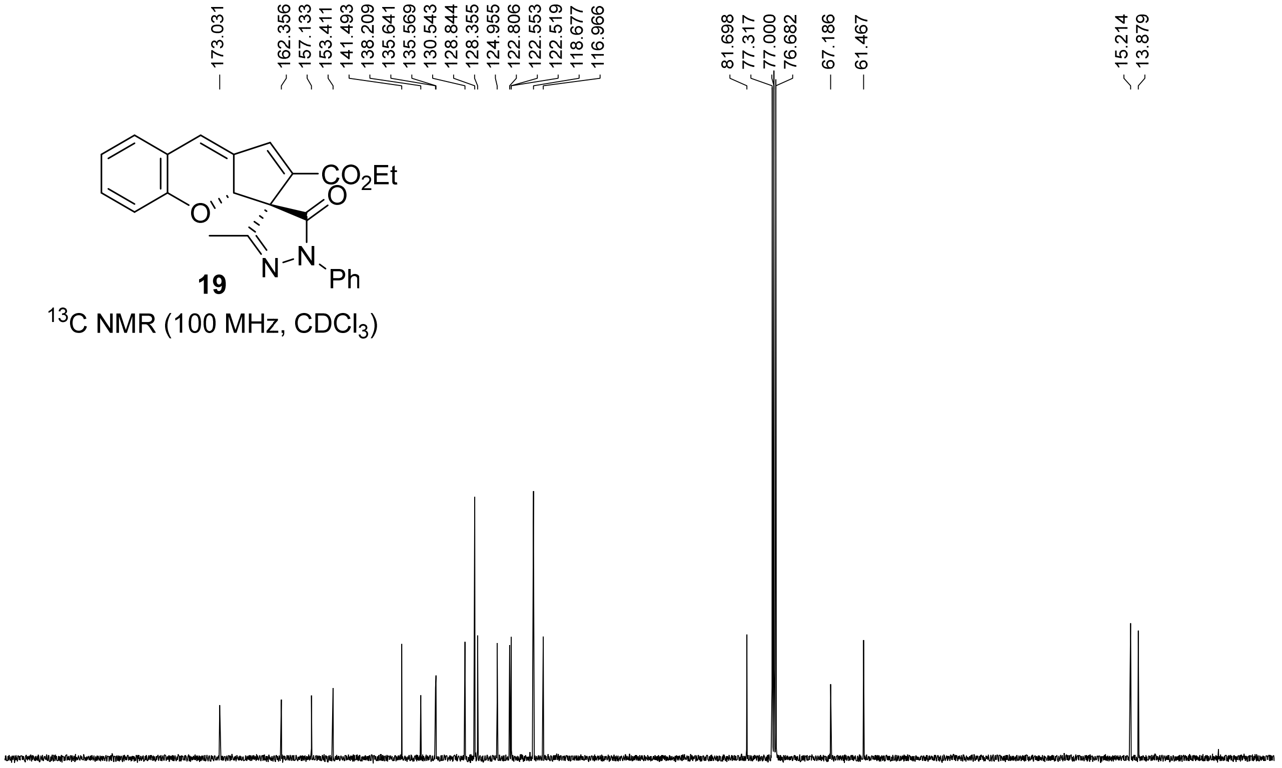
173.031
162.356 157.133 153.411 141.493 138.209 135.641 135.569 130.543 128.844 128.355 124.955 122.806 122.553 122.519 118.677 116.966
81.698 77.317 77.000 76.682
67.186
61.467
15.214 13.879
CO2Et
O
O
N
N
Ph
19
13C NMR (100 MHz, CDCl3)
f1 (ppm)

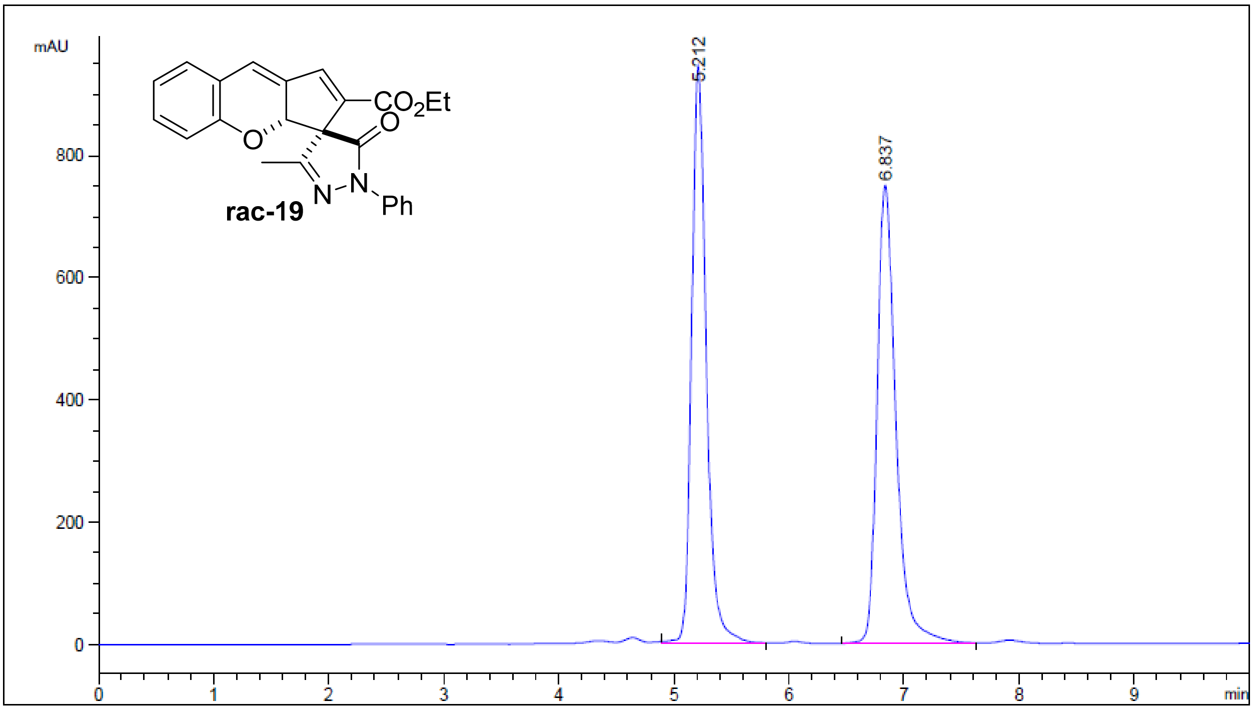

Peak #	RetTime [min]	Type	Width [min]	Area mAU *s	Height [mAU]	Area %
1	5.212	BBA	0.1339	8364.51465	944.15839	49.4238
2	6.837	BBA	0.1725	8559.54492	749.68304	50.5762

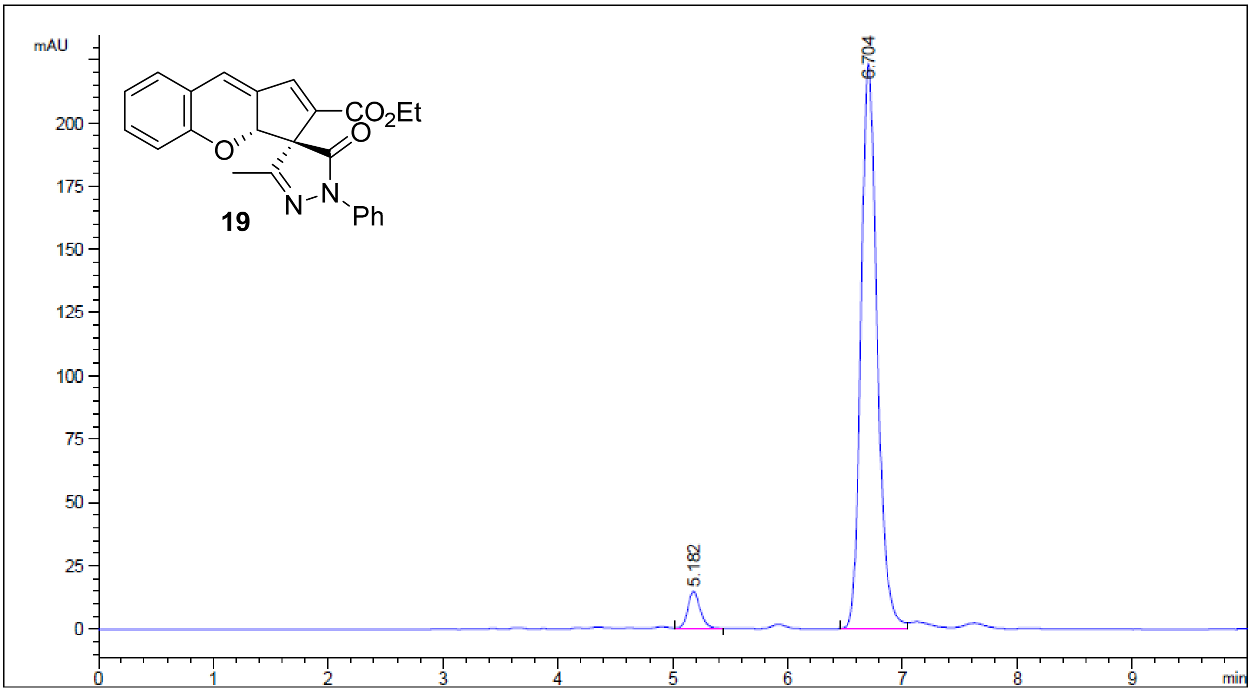

Peak #	RetTime [min]	Type	Width [min]	Area mAU *s	Height [mAU]	Area %
1	5.182	VB	0.1129	108.23647	14.56946	4.7055
2	6.704	BV	0.1510	2191.96777	223.22267	95.2945

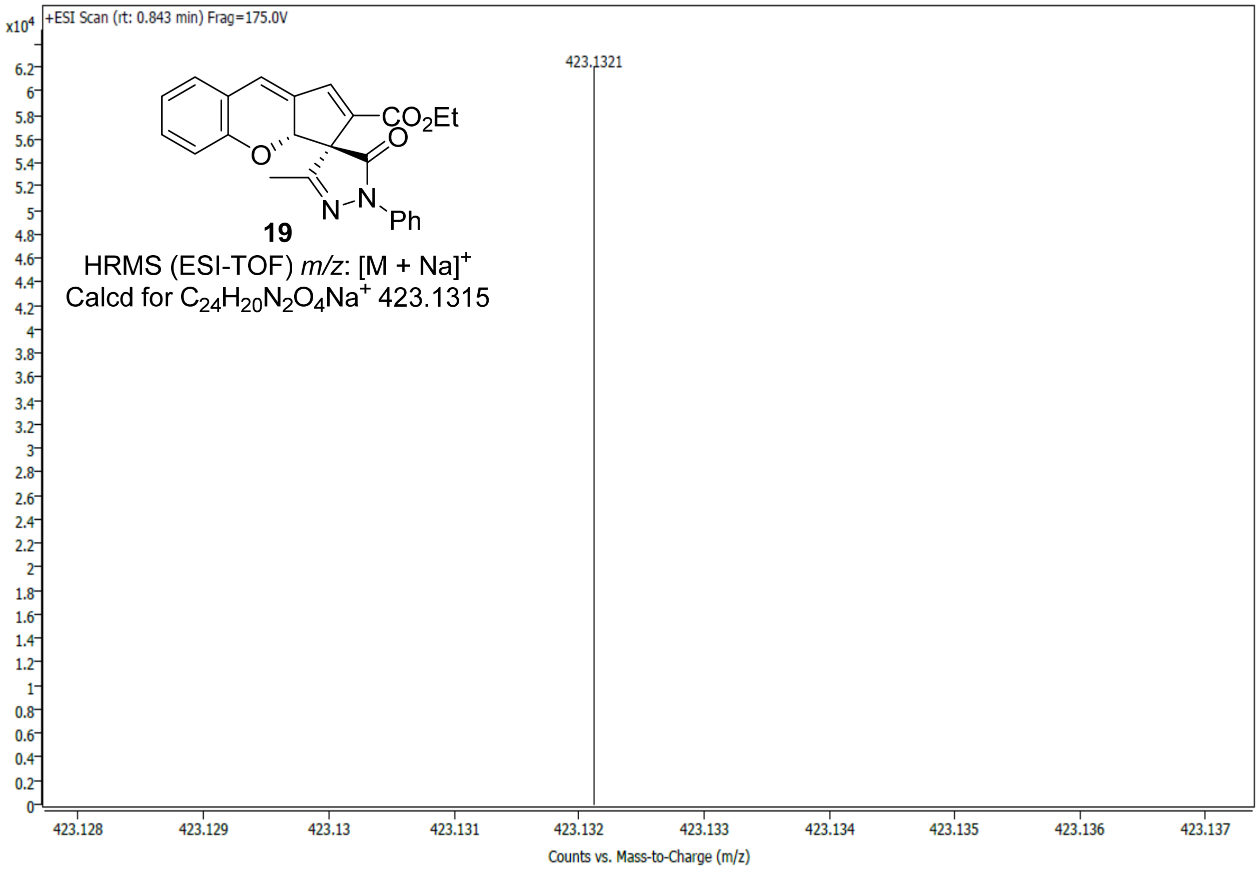
+ESI Scan (rt: 0.843 min) Frag=175.0V
x10^4
423.1321
CO2Et
O
N
Ph
19
HRMS (ESI-TOF) m/z: [M + Na]+
Calcd for C24H20N2O4Na+ 423.1315
Counts vs. Mass-to-Charge (m/z)

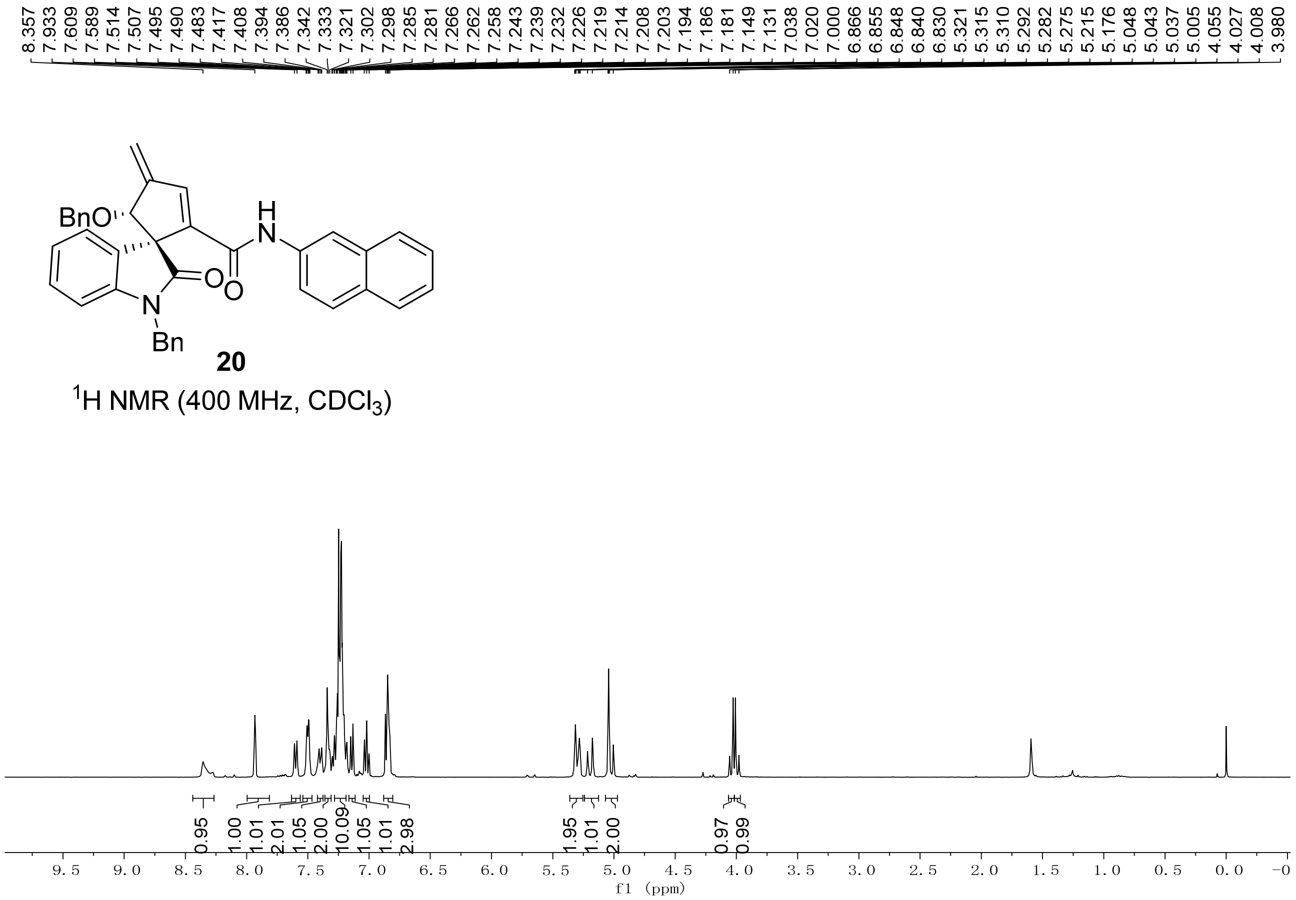

20
^{1}H NMR (400 MHz, $CDCl_3$)

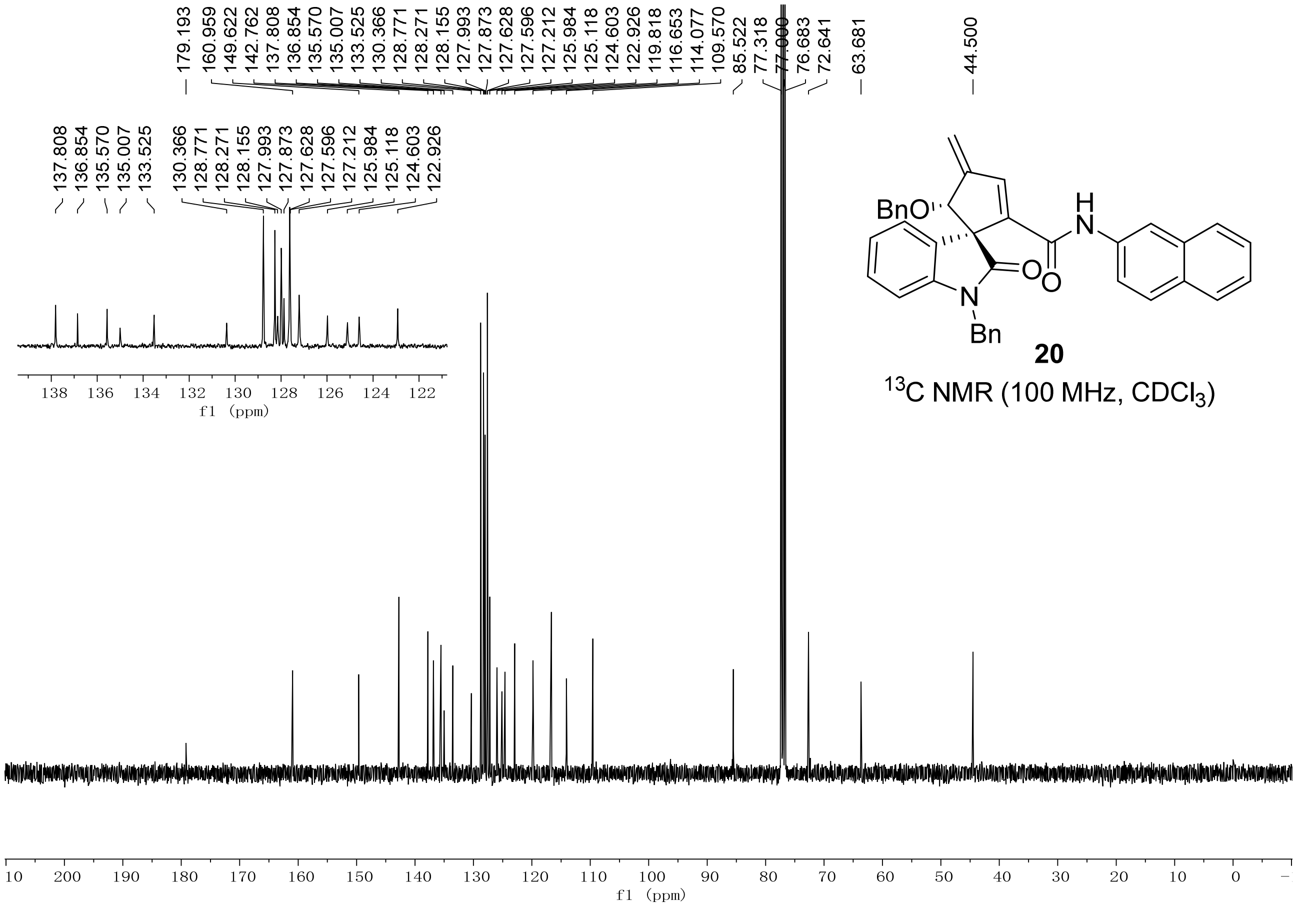

20
^{13}C NMR (100 MHz, $CDCl_3$)

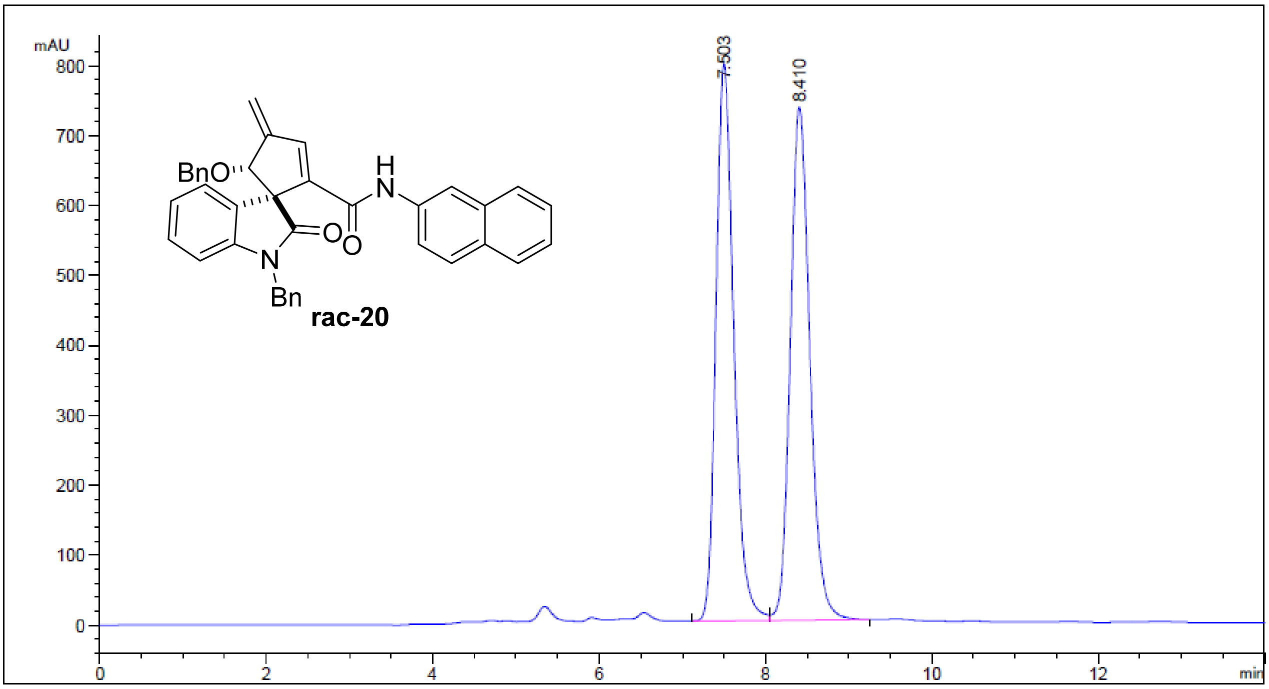

Peak #	RetTime [min]	Type	Width [min]	Area mAU *s	Height [mAU]	Area %
1	7.503	BV	0.2292	1.18608e4	795.87494	49.7425
2	8.410	VBA	0.2517	1.19836e4	733.66876	50.2575

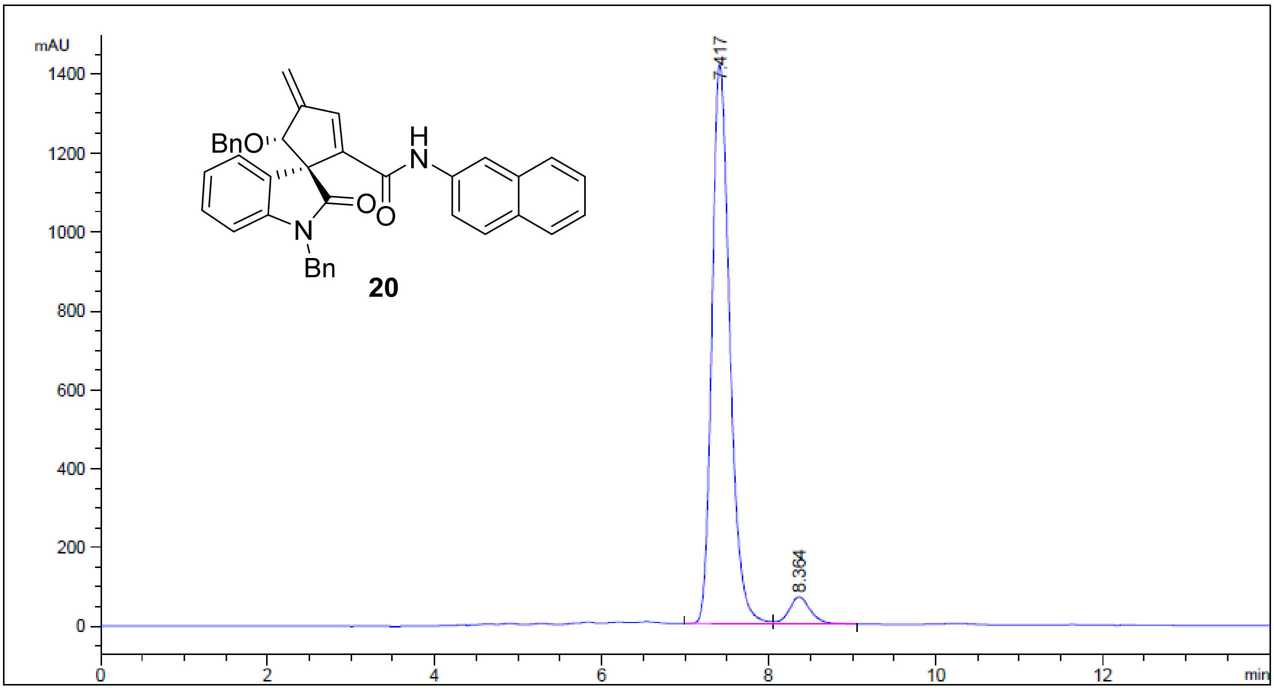

Peak #	RetTime [min]	Type	Width [min]	Area mAU *s	Height [mAU]	Area %
1	7.417	BV	0.2289	2.11011e4	1418.47095	94.4466
2	8.364	VB	0.2753	1240.73230	68.51608	5.5534

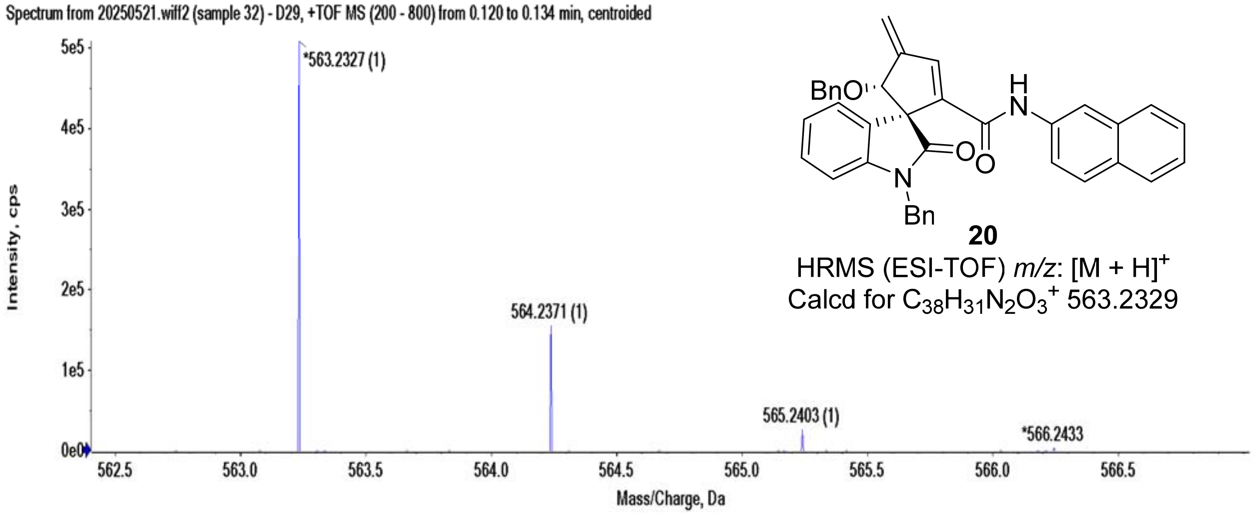

20

HRMS (ESI-TOF) *m/z*: $[M + H]^+$

Calcd for $C_{38}H_{31}N_2O_3^+$ 563.2329

11. DFT computational calculation data

11.1 FMO analyses for Pd0-allene complex

2a'

Zero-point correction= 0.113254 (Hartree/Particle)

Thermal correction to Energy= 0.119729

Thermal correction to Enthalpy= 0.120673

Thermal correction to Gibbs Free Energy= 0.083776

Electronic Energy = -116.660961

C	1.24875500	0.41623500	0.39298800
H	1.55001500	1.09112200	1.19671900
C	0.00001600	0.42299700	-0.00004100
C	-1.24873900	0.41617800	-0.39299000
H	-1.55005100	1.09105000	-1.19673000
C	-2.32934500	-0.46450300	0.18770400
H	-3.14545400	0.14020500	0.60420600
H	-1.93333100	-1.10291900	0.98225600
C	2.32933900	-0.46452500	-0.18767900
H	1.93323200	-1.10301600	-0.98212300
H	2.76841600	-1.10738800	0.58638300
H	3.14544700	0.14009300	-0.60429700
H	-2.76843000	-1.10743400	-0.58630600

INT6

Zero-point correction= 0.487893 (Hartree/Particle)

Thermal correction to Energy= 0.516222

Thermal correction to Enthalpy= 0.517167

Thermal correction to Gibbs Free Energy= 0.429197

Electronic Energy = -1131.651883

C	3.60603900	0.46237100	1.01735600
C	5.05445800	-1.37405200	0.12615100
C	3.14187000	-1.91353700	1.68183300
C	-3.61231900	0.09770300	-1.22591000
C	-3.68399200	0.86440200	1.16899600
C	-5.21850500	-0.96632400	0.40764500
C	0.62708400	-1.93813800	-0.53760800
C	-0.79309900	-1.99945400	-0.18112400
O	1.25547800	-3.01739000	-1.03250400
C	2.61436800	-2.56405700	-1.31383300
C	2.69289800	-1.13659700	-0.69807700
N	1.28548100	-0.85381100	-0.35789000
N	-1.37398300	-0.98109200	0.33633100
C	-2.76011700	-1.39507300	0.64178600
C	-2.86884900	-2.78066100	-0.04936900
O	-1.49198100	-3.13071300	-0.38327500
C	3.62079900	-0.99943400	0.54243300
C	-3.82110300	-0.34594200	0.23032000
H	2.59454300	0.79572900	1.26460900
H	4.24172400	0.57593100	1.90332300
H	3.99339400	1.13046200	0.23977100
H	5.14909300	-2.42781800	-0.16094600
H	5.74198000	-1.20233300	0.96147500

H	5.39497500	-0.75991100	-0.71650900
H	3.82762900	-1.84160800	2.53295200
H	2.14644600	-1.61634600	2.02670800
H	3.10176300	-2.96781900	1.38161800
H	-3.69202100	-0.74160600	-1.92803600
H	-4.37516900	0.83209000	-1.50674300
H	-2.62835700	0.55950900	-1.35108800
H	-3.88795200	0.57649000	2.20806000
H	-4.40119900	1.64479800	0.89042600
H	-2.67570600	1.28445300	1.11891800
H	-5.98982600	-0.21453400	0.20807600
H	-5.39321400	-1.80267100	-0.27981500
H	-5.36618400	-1.32982900	1.43233800
H	2.74213200	-2.56942600	-2.39924700
H	3.29409700	-3.28759200	-0.86404800
H	3.01667900	-0.40649500	-1.44659300
H	-2.82325300	-1.51625500	1.73187600
H	-3.26558500	-3.56772900	0.59212700
H	-3.43102000	-2.75366700	-0.98679900
Pd	-0.01539000	0.92281600	0.13856400
C	-0.67320200	2.92671700	0.27254000
H	-0.89991000	3.17393900	1.31402800
C	0.67989800	2.78185800	-0.08324500
C	1.80325500	3.39930800	-0.43566900
H	2.68130700	2.81017700	-0.70006100
C	1.96594800	4.89900600	-0.48765200
H	2.76187400	5.24100500	0.18949700
H	1.03714800	5.40235300	-0.20092400
C	-1.70780500	3.42209000	-0.72616800
H	-1.43651600	3.13150000	-1.74581200
H	-1.77964800	4.52015000	-0.69821100
H	-2.70659900	3.02511400	-0.51435000
H	2.24109300	5.24042500	-1.49581400

11.2. Data of all the Optimized Compounds and Transition State

11.2.1 Absolute Calculation Energies, Enthalpies, and Free Energies

Geometry	E(elec-B3LYP-D3)[1]	G(corr-B3LYP-D3)[2]	H(corr-B3LYP-D3)[3]	E(solv,B3LYP-D3)[4]	IF[5]
**1b**	-1262.713669	0.307532	0.390406	-1263.119304	-
**2a'**	-195.304139	0.083776	0.120673	-195.372928	-
**3b'**	-1035.831969	0.278601	0.352039	-1036.159201	-
**INT0**	-2198.698149	0.653135	0.786768	-2199.339068	-

**INT1**	-1776.828033	0.527122	0.63619	-1777.374362	-
**INT2**	-1972.162639	0.632158	0.758831	-1972.765508	-
**INT2'**	-1972.16593	0.633768	0.759265	-1972.765502	-
**INT3**	-1972.201025	0.635887	0.761683	-1972.808654	-
**INT4**	-1971.755704	0.624654	0.747863	-1972.335173	-
**INT5**	-1971.795578	0.624359	0.748722	-1972.358957	-
**INT6**	-1131.263381	0.429197	0.517167	-1131.575222	-
**TS1**	-2198.668065	0.654727	0.785125	-2199.31588	-187.06
**TS2**	-1972.126314	0.629907	0.756922	-1972.731058	-393.96
**TS2'**	-1972.123908	0.629099	0.757009	-1972.724193	-362.01
**TS3**	-1971.745281	0.621125	0.746451	-1972.323328	-230.29
***ent*-TS3**	-1971.741972	0.623492	0.746549	-1972.322114	-193.53
**TS3'**	-1971.725685	0.621084	0.745957	-1972.310597	-315.96
**TS3-RE**	-1971.723269	0.623622	0.745682	-1972.290265	-251.1
**TS3-inner-*syn***	-1971.726688	0.622318	0.744934	-1972.293870	-338.19
**TS3-inner-*anti***	-1971.729365	0.622059	0.745549	-1972.298745	-192.51
**TS4**	-2393.982689	0.763429	0.908844	-2394.685	-466.45
***t*-BuO$^-$**	-233.051226	0.091707	0.127872	-233.245666	-
***t*-BuOH**	-233.684853	0.107429	0.144008	-233.779426	-
**OBoc$^-$**	-421.703966	0.105365	0.148078	-421.944193	-

[1]The electronic energy calculated by B3LYP-D3 in gas phase. [2]The thermal correction to Gibbs free energy calculated by B3LYP-D3 in gas phase. [3]The thermal correction to enthalpy calculated by B3LYP-D3 in gas phase. [4]The electronic energy calculated by B3LYP-D3 in toluene solvent. [5]The B3LYP-D3 calculated imaginary frequencies for the transition states.

11.2.2 Geometries for All the Optimized Compounds and Transition State

1b

C	5.44503400	-0.09951900	-1.06403700

C	5.42998200	-1.24662100	-0.25038200
C	4.24403400	-1.70505800	0.32210000
C	3.07742300	-0.98435100	0.06927100
C	3.09249800	0.15807200	-0.74053800
C	4.27492300	0.61404500	-1.32143800
C	1.69994200	-1.26300700	0.54125800
C	0.81480600	-0.05079200	0.13100300
C	1.72523500	0.71747600	-0.88379400
O	1.37844900	1.60752000	-1.62585500
O	1.30952500	-2.26183000	1.10371700
C	0.56503200	0.89050700	1.30335400
C	1.33372400	0.88849400	2.39753900
O	-0.31442600	-0.52287100	-0.59468200
O	-2.35507000	-1.16111400	-0.81195300
C	-3.70833300	-1.55462700	-0.37500000
C	-4.41227100	-1.82909600	-1.70269400
C	-4.37341000	-0.39315700	0.36648700
C	-3.62871300	-2.82230500	0.47673900
C	-1.43603300	-0.83598900	0.09549200
O	-1.55330100	-0.80316100	1.29938900
C	-0.47733000	1.96992600	1.20921300
O	-0.71048600	2.76718400	2.09418100
O	-1.11254700	1.95024800	0.02983400
C	-2.60870200	2.75878500	-1.61431600
C	-2.14570400	2.93169400	-0.18077000
H	6.38381500	0.22927800	-1.49996900
H	6.35749900	-1.78256100	-0.07164700
H	4.21462000	-2.59269100	0.94590000
H	4.26866300	1.49591200	-1.95384600
H	1.16409300	1.63809300	3.16230100
H	2.11505900	0.15716900	2.56996300
H	-3.90286100	-2.62779400	-2.25037100
H	-5.44716400	-2.13515000	-1.51958800
H	-4.41906000	-0.93083200	-2.32806400
H	-3.85228100	-0.17053800	1.29836300
H	-5.41105400	-0.65703400	0.59725900
H	-4.38268300	0.50368200	-0.26179700
H	-4.64148500	-3.17885800	0.69331300
H	-3.09703300	-3.61013400	-0.06695400
H	-3.11324400	-2.63244800	1.41908300
H	-3.41499100	3.46468000	-1.84026500
H	-1.77832500	2.93435900	-2.30461800
H	-2.97313900	1.73964500	-1.77793300
H	-2.94693300	2.75382400	0.54432000
H	-1.73347700	3.92674000	0.01057900

2a'

C	1.24875500	0.41623500	0.39298800
H	1.55001500	1.09112200	1.19671900
C	0.00001600	0.42299700	-0.00004100
C	-1.24873900	0.41617800	-0.39299000
H	-1.55005100	1.09105000	-1.19673000
C	-2.32934500	-0.46450300	0.18770400

H	-3.14545400	0.14020500	0.60420600
H	-1.93333100	-1.10291900	0.98225600
C	2.32933900	-0.46452500	-0.18767900
H	1.93323200	-1.10301600	-0.98212300
H	2.76841600	-1.10738800	0.58638300
H	3.14544700	0.14009300	-0.60429700
H	-2.76843000	-1.10743400	-0.58630600

3b'

C	4.00070600	-1.60042200	0.46043000
C	3.80651200	-1.75015600	-0.92422500
C	2.56010700	-1.51121000	-1.50388300
C	1.51785100	-1.11503600	-0.66759200
C	1.71205400	-0.96374700	0.70872700
C	2.95296000	-1.20743700	1.29367300
C	0.11997400	-0.76865500	-1.03166900
C	-0.62708500	-0.37365700	0.26599200
C	0.45458000	-0.51169100	1.35635700
O	0.28579100	-0.32231200	2.54465800
O	-0.35657300	-0.78761900	-2.14870600
C	-1.22570800	1.01328900	0.13722900
C	-2.57086600	0.97371500	0.05410900
C	-0.42417400	2.23774500	-0.00715500
O	-0.85643200	3.36955700	-0.09951800
O	0.89597800	1.92764400	-0.03807100
C	3.21101400	2.41523000	-0.25509300
C	1.82033200	3.01958800	-0.20513700
H	4.98268700	-1.79389000	0.88247300
H	4.64183400	-2.05663500	-1.54723900
H	2.39377600	-1.62120400	-2.57085400
H	3.08683400	-1.08484400	2.36383700
H	-3.17920400	1.85605600	-0.10508600
H	3.95807900	3.20442700	-0.39080900
H	3.43430100	1.87870800	0.67204800
H	3.29562300	1.70714100	-1.08546000
H	1.56644900	3.55666900	-1.12443400
H	1.69809000	3.71473600	0.63158100
C	-1.88499800	-1.25730900	0.58749900
H	-1.89830400	-1.32801700	1.68392600
C	-3.07175900	-0.38394900	0.17934400
C	-4.33276800	-0.80850100	-0.01318700
H	-4.53797200	-1.87187200	0.10245700
C	-5.51082100	0.03963100	-0.38690500
H	-5.25297100	1.09678100	-0.49009900
H	-6.30626900	-0.04367300	0.36590100
C	-1.86121200	-2.66807100	0.00198600
H	-1.89639500	-2.64260900	-1.09016200
H	-2.71747600	-3.24787500	0.36122500
H	-0.95475100	-3.20254500	0.30939600
H	-5.94465200	-0.29565900	-1.33861400

INT0

C	0.63706000	-1.97304900	3.47775400
C	0.44129200	-0.64988000	3.91032200

C	-0.33279600	0.23964100	3.16371800
C	-0.87807500	-0.21220100	1.96421000
C	-0.67519200	-1.52916600	1.52987300
C	0.06702900	-2.43178300	2.29032300
C	-1.75584600	0.51576300	1.00999100
C	-1.83471700	-0.36053900	-0.28705000
C	-1.40894700	-1.76164000	0.26196700
O	-1.66344300	-2.83069600	-0.25428400
O	-2.31803900	1.56771400	1.22059900
C	-0.81930300	0.07129700	-1.34417900
C	-0.16863700	-0.92669500	-2.12545200
O	-3.13887400	-0.38119700	-0.87525400
O	-5.26943300	-0.69967200	-0.71065400
C	-6.53392600	-1.06390200	-0.04585300
C	-7.55944000	-0.85176900	-1.15835500
C	-6.79215600	-0.11510600	1.12581800
C	-6.48884300	-2.53077000	0.38513500
C	-4.12293900	-0.76183800	-0.04109200
O	-3.94727800	-1.09712500	1.11594400
C	-0.86212400	1.45623200	-1.90329600
O	-0.03173100	1.93569600	-2.66142100
O	-1.94020700	2.13639000	-1.48191100
C	-3.27525800	4.07132300	-1.19082800
C	-2.07299500	3.49989300	-1.91753100
H	1.22720300	-2.65001100	4.08957500
H	0.88413800	-0.32477900	4.84745800

H	-0.51535300	1.25523400	3.49876600
H	0.19172800	-3.45500500	1.95271400
H	0.26715500	-0.61785800	-3.07229400
H	-0.49873600	-1.95846000	-2.07356200
H	-7.33680500	-1.49636200	-2.01418300
H	-8.56265500	-1.09093000	-0.79125400
H	-7.54943800	0.18888700	-1.49684300
H	-6.05042900	-0.25203900	1.91374700
H	-7.78788300	-0.30968700	1.53895900
H	-6.75901900	0.92483800	0.78447200
H	-7.47232200	-2.82837900	0.76493900
H	-6.24357600	-3.16885100	-0.47023700
H	-5.74613900	-2.68829600	1.16826200
H	-3.41766800	5.12275500	-1.46400400
H	-4.18085100	3.51541400	-1.45270200
H	-3.13342400	3.99563000	-0.10913600
H	-1.15152700	4.04239900	-1.68035600
H	-2.19355400	3.51470900	-3.00588600
C	0.36303200	3.34436700	1.20187000
C	1.76743600	5.29938100	0.49420400
C	2.62495700	3.66631500	2.22030300
C	1.86885600	-3.92551900	-0.71281500
C	4.22553700	-4.77230900	-0.70089000
C	3.53225500	-2.97599600	-2.33709700
C	3.64834700	1.16260400	-0.04162200
C	3.99513500	-0.25919100	0.06870800

O	4.58343400	2.05390800	-0.41412700
C	3.85637700	3.30729100	-0.60927500
C	2.38526800	2.97942500	-0.21878800
N	2.43379200	1.54082400	0.11421000
N	3.08756000	-1.15391300	-0.08978700
C	3.72017800	-2.46801600	0.14470300
C	5.22761900	-2.10420300	0.22691100
O	5.25298500	-0.64677600	0.32559300
C	1.79516300	3.82689100	0.93657500
C	3.34077500	-3.53107200	-0.91699900
H	0.36377700	2.30306700	1.52316200
H	-0.11158000	3.94700400	1.98442300
H	-0.25919600	3.41024300	0.30543700
H	2.77276200	5.69891900	0.31330000
H	1.30382300	5.91889200	1.26980300
H	1.18345900	5.42203600	-0.42608900
H	2.18174600	4.25687400	3.02976900
H	2.64548200	2.61873700	2.53925000
H	3.65990000	4.00714100	2.09781100
H	1.21051400	-3.05641300	-0.78587000
H	1.56093400	-4.65335300	-1.47164400
H	1.72047600	-4.38602000	0.27145100
H	5.28475000	-4.57051700	-0.89987700
H	3.91461000	-5.57538400	-1.37759300
H	4.13665100	-5.15038700	0.32534000
H	3.28942800	-3.74816400	-3.07514600
H	2.87419200	-2.11868600	-2.50787200
H	4.56573600	-2.65859800	-2.52328300
H	3.97105600	3.58787200	-1.65789300
H	4.33111600	4.05866100	0.02359500
H	1.72252200	3.07656500	-1.08583400
H	3.36264000	-2.82652500	1.11935400
H	5.73264200	-2.50329600	1.10652600
H	5.78832800	-2.37377700	-0.67154600
Pd	1.09704000	-0.32245800	-0.56107500

INT1

C	5.57453700	-0.30340600	1.79100200
C	5.72333800	-0.31694200	0.39275800
C	4.67768700	0.07296000	-0.44656500
C	3.48463500	0.47703200	0.14864800
C	3.33546900	0.48540600	1.54587800
C	4.37653700	0.09851100	2.38541400
C	2.22043400	0.88568600	-0.50224700
C	1.24607800	1.18985800	0.61075700
C	1.95930600	0.89423200	1.92068300
O	1.50310500	0.97974300	3.04568900
O	1.97581200	0.89918300	-1.69753200
C	0.17888600	2.12772700	0.47904000
C	-0.81960200	2.09283400	1.46799700
C	-0.05572200	2.90274900	-0.79969300
O	-1.10979700	2.88957400	-1.39884900
O	1.02528800	3.60445900	-1.11184600

C	2.35066200	4.94844900	-2.58058900	C	-4.04842700	-0.66049800	0.65236300
C	0.97986600	4.34324600	-2.37030100	C	-4.75576300	-1.42289000	-0.49309600
H	6.40637100	-0.61411200	2.41538400	O	-3.67421900	-2.16692700	-1.15592000
H	6.66834900	-0.63468500	-0.03668200	C	1.54160100	-3.41210900	-0.21452800
H	4.78151200	0.06947800	-1.52679800	C	-4.60016000	0.75215200	0.94655500
H	4.24716400	0.10543300	3.46257100	H	1.23925100	-2.13739600	1.54465900
H	-1.72565000	2.66329900	1.30137800	H	2.42288600	-3.43165200	1.76756700
H	-0.55577200	1.83224400	2.48950900	H	2.85465300	-2.00883000	0.81727500
H	2.36061100	5.51203500	-3.51896600	H	2.44968100	-4.66920300	-1.78461100
H	2.60848700	5.63194000	-1.76601500	H	3.21025400	-4.80106300	-0.20037100
H	3.11347200	4.16614200	-2.63945800	H	3.49771100	-3.36430700	-1.19346700
H	0.70756500	3.63574700	-3.15760900	H	0.92241600	-5.19447500	0.85150500
H	0.19161500	5.09671700	-2.29170400	H	-0.35827400	-3.99086500	0.70869500
C	2.03996500	-2.69772000	1.05107300	H	0.08336600	-4.99933000	-0.68569600
C	2.73970000	-4.09855100	-0.89554300	H	-2.94334500	1.10263700	2.34195300
C	0.48061200	-4.45428600	0.17731800	H	-4.27406800	2.26614400	2.48192000
C	-4.02829200	1.21487400	2.29876600	H	-4.45239800	0.62564400	3.11984000
C	-6.13270400	0.64852900	1.08429500	H	-6.62074800	0.39841800	0.13573700
C	-4.24602100	1.73355200	-0.18128200	H	-6.53863300	1.61096300	1.41148500
C	-1.24870500	-2.05539000	-1.21380300	H	-6.42059200	-0.10206200	1.83010400
C	-2.55980800	-1.61515100	-0.70905900	H	-4.64959400	2.72465400	0.05101300
O	-1.13254400	-2.89544700	-2.22589100	H	-3.16893900	1.83380700	-0.33350100
C	0.31758800	-2.97151200	-2.48453300	H	-4.68419900	1.42797800	-1.13868600
C	0.96388300	-2.36570800	-1.21270100	H	0.50414400	-2.38184400	-3.38458400
N	-0.18499200	-1.63091400	-0.62503500	H	0.55143500	-4.01830900	-2.66387800
N	-2.61506700	-0.73044100	0.22712100	H	1.74106000	-1.64696100	-1.47929000

H	-4.11091100	-1.24529300	1.57962800
H	-5.49240900	-2.15059600	-0.15861000
H	-5.18915700	-0.76613800	-1.25018900
Pd	-0.62285900	0.14210500	0.49887900

INT2

C	-2.55214100	3.70080600	3.45466100
C	-3.93179200	3.60924900	3.20653100
C	-4.44639700	2.66289400	2.31399700
C	-3.54825800	1.80908800	1.68759600
C	-2.16532700	1.89910600	1.93783500
C	-1.64840400	2.84771300	2.81756100
C	-3.80923500	0.73422500	0.68319600
C	-2.47331300	0.12092200	0.39435500
C	-1.45956200	0.91511300	1.09959800
O	-0.22356300	0.83598300	0.97511300
O	-4.88062300	0.44076800	0.18678200
C	-2.22703100	-0.98078600	-0.35769900
C	-0.91028200	-1.63877100	-0.50765100
C	-3.36209700	-1.62110000	-1.12081700
O	-3.45080900	-1.51689800	-2.32560600
O	-4.15573500	-2.32166100	-0.32347600
C	-6.17859200	-3.49686900	0.17091600
C	-5.36598200	-2.86067100	-0.93586800
H	-2.18633100	4.44959600	4.15016100
H	-4.60745600	4.28999700	3.71508000
H	-5.50940700	2.59053300	2.10894100
H	-0.58058100	2.91624400	2.99788100
H	-0.60059700	-2.07741700	0.44786700
H	-0.94191600	-2.41195200	-1.27134900
H	-7.10339500	-3.90947200	-0.24505100
H	-5.62410800	-4.30952200	0.65043900
H	-6.44171100	-2.75441400	0.93012500
H	-5.88954000	-2.02840100	-1.41235400
H	-5.06955500	-3.57576200	-1.70830700
C	1.41918300	-4.04072200	0.80914100
C	2.89184600	-4.51139100	2.77595900
C	3.87674400	-3.57157700	0.64895000
C	0.46720900	3.24365700	-1.58110900
C	2.45797900	4.72369200	-1.86447200
C	1.89204600	3.77606800	0.41074800
C	3.13460500	-0.31317500	1.11948400
C	3.27747300	0.78319900	0.14451800
O	4.04534000	-0.41972400	2.09037400
C	3.56971700	-1.51207000	2.93945000
C	2.39074700	-2.13720500	2.14001800
N	2.16764700	-1.14899300	1.05668100
N	2.36871500	1.04766700	-0.72571100
C	2.79217100	2.27602600	-1.44613500
C	4.27216100	2.40740600	-1.00112300
O	4.38119100	1.52250500	0.16160900
C	2.65724000	-3.56492800	1.58498600
C	1.90182400	3.50573200	-1.10331600

H	1.23314300	-3.40884000	-0.06161900
H	1.56345300	-5.06825700	0.45888700
H	0.52364300	-4.02939200	1.44268500
H	3.79049900	-4.25300100	3.34655200
H	3.02099100	-5.53775500	2.41768400
H	2.03827800	-4.50348000	3.46421600
H	3.71213900	-2.92374600	-0.21925300
H	4.79192100	-3.24055400	1.15257800
H	4.05953800	-4.58553200	0.27889500
H	0.01784800	2.40970500	-1.04035200
H	-0.15259000	4.13109400	-1.41289700
H	0.43921800	3.00790400	-2.64970400
H	3.46111000	5.01078500	-1.53149700
H	1.80638600	5.58850900	-1.70409000
H	2.49851500	4.53400800	-2.94372000
H	1.29438100	4.66915900	0.62139500
H	1.44420400	2.93856600	0.95538000
H	2.89619200	3.95663200	0.81114700
H	3.26234600	-1.07130900	3.89018600
H	4.41288100	-2.18173200	3.10144000
H	1.48518400	-2.17657100	2.75467600
H	2.71953900	2.08792300	-2.52161200
H	4.98352200	2.04356500	-1.74639100
H	4.56081000	3.40295900	-0.67102100
Pd	0.64476100	-0.33397600	-0.75104800
C	-0.69736700	0.38475300	-3.51881200
H	-0.59963400	0.26089000	-4.60058300
C	0.22222000	-0.22692900	-2.79832400
C	1.29395900	-1.09642600	-2.65044000
H	2.29987100	-0.67745100	-2.71757700
C	1.22561700	-2.58006200	-2.93047900
H	1.60333600	-2.75447800	-3.94698700
H	0.20535200	-2.96442300	-2.88863400
C	-1.85618300	1.21551300	-3.05705800
H	-1.82499400	1.38888800	-1.98131200
H	-2.79428900	0.70315500	-3.29301400
H	-1.86058100	2.18476300	-3.56869100
H	1.85779200	-3.15189500	-2.24435700

INT2'

C	-7.02879800	-0.87923200	-1.25242500
C	-7.18724100	0.09471000	-0.25162700
C	-6.12545900	0.44844300	0.58451900
C	-4.91026100	-0.20391300	0.40005100
C	-4.75131500	-1.17633900	-0.60138400
C	-5.80483100	-1.52580600	-1.44113000
C	-3.62109300	0.00107400	1.11148200
C	-2.66031400	-0.98673100	0.52574700
C	-3.35180800	-1.68089300	-0.60653200
O	-2.88321900	-2.49289200	-1.39175200
O	-3.37096900	0.87227500	1.93182500
C	-1.36365400	-1.14976200	0.89590200
C	-0.34422100	-1.85530500	0.11587900

C	-0.78083500	-0.37322300	2.03892400
O	0.20893300	0.34001200	1.83654900
O	-1.34054500	-0.57186500	3.20459000
C	-1.92865500	0.07939400	5.42585500
C	-0.93335900	0.29633300	4.30740300
H	-7.87287600	-1.12902600	-1.88788900
H	-8.15149200	0.57872200	-0.13068900
H	-6.23423100	1.20299700	1.35674000
H	-5.66812600	-2.27682600	-2.21241900
H	0.41186200	-2.35962900	0.71779500
H	-0.74279800	-2.47802600	-0.67519700
H	-1.66866400	0.71906600	6.27536500
H	-2.93680200	0.33735300	5.08974800
H	-1.92306800	-0.96154700	5.76261800
H	0.08942400	0.02828600	4.58751700
H	-0.94111300	1.32326200	3.93535400
C	2.54030900	-3.68644500	-0.52782000
C	4.68491000	-4.15812700	0.66425300
C	4.64086600	-2.54292700	-1.27652100
C	-0.41795800	3.32386200	-0.10452900
C	0.96898900	5.31091500	-0.70767000
C	1.65046200	3.81971700	1.21613300
C	3.61058100	0.18854500	0.27141600
C	3.19034300	1.44044400	-0.37921200
O	4.82891900	0.09736200	0.79779100
C	4.84375300	-1.19689600	1.48653900
C	3.57859000	-1.92602800	0.95767700
N	2.81009300	-0.81139800	0.34685300
N	1.97974500	1.59854100	-0.78563200
C	1.84443700	3.01902400	-1.20302800
C	3.33064100	3.44223500	-1.32317400
O	4.05569000	2.43258900	-0.54061900
C	3.86692000	-3.07112100	-0.05729700
C	1.01799600	3.86236700	-0.18541100
H	1.92467100	-2.95129300	-1.05010500
H	2.73469900	-4.51694000	-1.21451900
H	1.96261700	-4.08416700	0.31497000
H	5.67499500	-3.80647300	0.97343400
H	4.84048200	-5.01052600	-0.00463000
H	4.16066500	-4.52519900	1.55456700
H	4.05305200	-1.80103200	-1.82854800
H	5.59718200	-2.08547300	-0.99978400
H	4.86188600	-3.36652500	-1.96278500
H	-0.44327500	2.31231800	0.30578200
H	-1.01726100	3.96487900	0.55078400
H	-0.89246400	3.31052900	-1.09087800
H	1.95462100	5.78803200	-0.73270600
H	0.33552900	5.91717100	-0.05260200
H	0.54297300	5.35608300	-1.71710000
H	1.07629200	4.45699200	1.89630700
H	1.63430700	2.80515400	1.62761700
H	2.68321100	4.18573300	1.22418900

H	4.80578200	-0.98813700	2.55817200
H	5.78621200	-1.67975500	1.23686800
H	2.99207300	-2.33669500	1.78535600
H	1.34300800	3.05220500	-2.17421300
H	3.71588700	3.38773400	-2.34444500
H	3.56705100	4.41021700	-0.88780900
Pd	0.72461700	-0.18547600	-0.50421300
C	-1.85592700	0.83382700	-1.93246600
H	-2.24124100	1.17408800	-0.97483300
C	-0.67780900	0.24586800	-1.97925300
C	0.32892200	-0.45430900	-2.62580600
H	1.15874500	0.11565600	-3.04875500
C	0.14165400	-1.83698800	-3.20352300
H	1.01959300	-2.46954500	-3.04036300
H	0.01469400	-1.73444600	-4.28998700
C	-2.73074100	1.07321900	-3.13592800
H	-2.26830100	0.69947000	-4.05306800
H	-2.94091200	2.14263800	-3.25886800
H	-3.69489200	0.56702200	-3.00225500
H	-0.74624500	-2.32790600	-2.80282000

INT3

C	7.16930800	2.94945800	0.62115700
C	7.97928700	1.80560300	0.51193500
C	7.43259100	0.56366600	0.18172100
C	6.05936100	0.49663700	-0.03323200
C	5.24989700	1.63976000	0.07311700
C	5.79231000	2.87930000	0.40350600
C	5.22956500	-0.68104100	-0.39926000
C	3.81490200	-0.17642500	-0.50791300
C	3.83456500	1.29291100	-0.20537100
O	2.87944600	2.05873100	-0.14955400
O	5.62080300	-1.81497100	-0.61092500
C	2.75401400	-0.92922300	-0.84993300
C	1.36278500	-0.39131200	-1.09603500
C	2.88839100	-2.41577200	-1.05414200
O	2.68691900	-2.94148100	-2.12840400
O	3.14790100	-3.04603200	0.09149000
C	3.89472200	-4.92990600	1.35457200
C	3.46618900	-4.46122000	-0.01957200
H	7.62645900	3.90042100	0.87685700
H	9.04711400	1.89368600	0.68720200
H	8.04724500	-0.32608200	0.09283600
H	5.15686500	3.75543300	0.48167200
H	1.39762900	0.69313000	-1.18056500
H	1.00107500	-0.82359000	-2.03282300
H	4.15462200	-5.99276300	1.31737500
H	4.77185700	-4.37034600	1.69242400
H	3.09084800	-4.79729900	2.08614200
H	2.58160800	-4.99009700	-0.38796800
H	4.26288500	-4.56286700	-0.76027900
C	-0.03756200	3.29297100	-0.38792400
C	-1.20880600	5.44643800	0.09533400

C	-2.04085700	3.93434100	-1.74803100	H	-3.84425000	-3.75254900	2.02012400
C	-3.56588900	-3.11397400	1.17541400	H	-2.96588200	-3.71841100	0.48710600
C	-5.67821300	-3.76244400	0.00187600	H	-6.62061600	-3.44642200	-0.45851100
C	-5.62787700	-1.74137700	1.51305700	H	-5.93333600	-4.41087800	0.84595800
C	-3.79521100	1.77025900	0.01986000	H	-5.12901600	-4.36687500	-0.72955600
C	-4.32231800	0.48351600	-0.46192700	H	-5.89822200	-2.36901600	2.36796400
O	-4.57631000	2.83750100	0.10414400	H	-5.03421400	-0.90472200	1.89866700
C	-3.75579500	3.84882600	0.78557200	H	-6.55885300	-1.34085000	1.09861200
C	-2.31241300	3.28502800	0.70632800	H	-4.13623800	3.92729200	1.80671800
N	-2.56513100	1.85398600	0.38697900	H	-3.90822200	4.78820500	0.25912900
N	-3.58029600	-0.56589500	-0.39645000	H	-1.81662200	3.33691500	1.67967300
C	-4.44313500	-1.72067900	-0.75426300	H	-3.90486600	-2.35457300	-1.46351900
C	-5.63833300	-1.00799500	-1.43530400	H	-5.55118200	-0.96251500	-2.52357900
O	-5.55142900	0.37670200	-0.94478600	H	-6.61952100	-1.38372800	-1.15486400
C	-1.40589300	3.98475100	-0.34825600	Pd	-1.53867300	-0.10097300	0.22650700
C	-4.83182600	-2.57319800	0.49233600	C	0.38989500	0.06856800	1.19258300
H	-0.12541600	2.26457900	-0.74507300	H	0.90325600	1.01913800	1.06696500
H	0.64112400	3.82313300	-1.06255400	C	0.49292700	-0.80648800	0.07778000
H	0.43651000	3.27377600	0.59893700	C	-0.39708100	-1.91711900	0.04187700
H	-2.14413100	6.01677800	0.10450800	H	-0.63129000	-2.39602200	0.99054000
H	-0.52883300	5.95342600	-0.59643400	C	-0.58214500	-2.79780800	-1.16732700
H	-0.76638300	5.50144000	1.09700300	H	0.23815600	-3.52045500	-1.25572600
H	-2.16341300	2.90150800	-2.09330000	H	-0.61186600	-2.22803600	-2.09959200
H	-3.01760400	4.42903400	-1.78750300	C	0.18109200	-0.33329500	2.63255700
H	-1.39097200	4.44355000	-2.46656500	H	-0.29849000	-1.30682100	2.75389200
H	-2.94225900	-2.30065900	1.55705800	H	-0.41580700	0.41368000	3.16619100

H	1.16116100	-0.37789300	3.12804800
H	-1.51326400	-3.36370000	-1.08450500

INT4

C	-5.69254200	2.35367200	-2.93692000
C	-6.63897500	1.32960700	-2.83405900
C	-6.34920300	0.15249400	-2.12435300
C	-5.10254100	0.04448300	-1.53241800
C	-4.15481600	1.06492900	-1.63887200
C	-4.42973900	2.23095300	-2.33421100
C	-4.50460300	-1.05729400	-0.70399700
C	-3.13182900	-0.65484700	-0.37687100
C	-2.90647400	0.65981000	-0.90250200
O	-1.92183700	1.43188500	-0.80277900
O	-5.08951000	-2.09444600	-0.38913800
C	-2.38311000	-1.46496800	0.55848500
C	-1.09235800	-1.92071700	0.56694400
C	-3.17272300	-1.99320300	1.74466300
O	-2.94393300	-3.04013900	2.31917600
O	-4.11316600	-1.11223200	2.13221500
C	-6.45079700	-1.04662500	2.60493100
C	-5.10425200	-1.57256500	3.07096000
H	-5.93972300	3.25589600	-3.49054300
H	-7.61016300	1.44938800	-3.30700900
H	-7.07406300	-0.65091000	-2.03071300
H	-3.68583600	3.01930700	-2.40400000
H	-0.84057600	-2.55554900	1.40883900
H	-7.23932300	-1.36094800	3.29872800
H	-6.44687300	0.04750800	2.55681200
H	-6.67220200	-1.43603200	1.60746900
H	-5.07615900	-2.66344400	3.10576500
H	-4.83263100	-1.18952400	4.06177800
C	4.64159300	-2.55398700	-1.24748400
C	7.01905100	-1.97322600	-0.74852400
C	5.57799200	-0.38296400	-2.08017300
C	-0.50069900	1.26735800	2.04019500
C	-1.03210100	3.69625900	1.76863500
C	1.13319600	2.97371500	2.85726900
C	4.01284900	1.17095700	0.39754700
C	2.87006900	2.08585700	0.27446500
O	5.24223600	1.64534000	0.60627600
C	6.06657900	0.46317200	0.85054500
C	5.17367900	-0.72933900	0.41182300
N	3.83121900	-0.10274300	0.33735500
N	1.69716800	1.58639000	0.14001400
C	0.73126900	2.69173500	0.33104300
C	1.63828300	3.91368000	0.06903900
O	2.99995600	3.41271600	0.34940800
C	5.60133400	-1.39892600	-0.92645900
C	0.10078600	2.65387900	1.76214100
H	3.62381600	-2.18827400	-1.39366500
H	4.96118700	-3.06579400	-2.16205400
H	4.62462800	-3.29258500	-0.43742500

H	7.77327700	-1.19617900	-0.58202700
H	7.31318600	-2.52068700	-1.65011400
H	7.06040500	-2.67367900	0.09438200
H	4.56515200	-0.00106800	-2.24654400
H	6.24182900	0.47033300	-1.89891800
H	5.90876000	-0.86264600	-3.00737200
H	0.27513600	0.49718300	2.10604600
H	-1.04015400	1.28478600	2.99433000
H	-1.19363600	0.98461000	1.24875600
H	-0.66646800	4.70996500	1.55908900
H	-1.51457900	3.71972100	2.75178800
H	-1.78734400	3.43503000	1.02053300
H	0.64877200	2.91437300	3.83769400
H	1.95458400	2.24698100	2.86041600
H	1.56361500	3.97720500	2.76722900
H	6.30405500	0.45092500	1.91742700
H	6.98061100	0.58571300	0.27232300
H	5.14846800	-1.50147600	1.18673700
H	-0.07303200	2.58840100	-0.39751300
H	1.63196600	4.23517600	-0.97664600
H	1.46999000	4.76583800	0.72400700
Pd	1.65570200	-0.56156000	-0.19405200
C	-0.06543100	-0.80582700	-1.46564800
H	-0.73561200	0.03138000	-1.30435200
C	-0.09202500	-1.83575500	-0.47320900
C	1.12772500	-2.60831000	-0.43128500
H	1.54513900	-2.89331900	-1.39495000
C	1.45398700	-3.56866700	0.68525400
H	1.25662900	-3.13981000	1.67193400
H	2.50976700	-3.85448100	0.64348200
C	0.40150800	-0.96274400	-2.89101400
H	1.09235100	-1.79431300	-3.05075000
H	0.87613900	-0.04299200	-3.24968500
H	-0.48196500	-1.13364900	-3.52329400
H	0.85581400	-4.48669600	0.59992100

INT5

C	-6.10340900	-0.15354200	-2.95262600
C	-6.51505800	-1.39418500	-2.43454400
C	-5.72365300	-2.08786600	-1.51803600
C	-4.51596200	-1.51078200	-1.13187400
C	-4.10739500	-0.27718500	-1.64621500
C	-4.89186800	0.41872300	-2.56410400
C	-3.51769900	-2.00976600	-0.14749400
C	-2.35505000	-0.99314800	-0.08308400
C	-2.80860000	0.11702000	-1.04722100
O	-2.19672700	1.14104300	-1.30552700
O	-3.60980600	-3.03554900	0.49686200
C	-2.12268600	-0.51413100	1.33738700
C	-0.90349600	-0.87852300	1.79656900
C	-3.11965900	0.20610500	2.11790000
O	-2.98567900	0.65116600	3.24424200
O	-4.27369200	0.33834200	1.39867200

C	-6.51464100	1.03771100	1.05207100
C	-5.35992800	1.02660500	2.03731200
H	-6.74197800	0.36328700	-3.66333800
H	-7.46470300	-1.81375700	-2.75428000
H	-6.02891300	-3.04414600	-1.10496500
H	-4.56149000	1.37774600	-2.95074900
H	-0.57475800	-0.67983100	2.80956200
H	-7.37894900	1.54967700	1.48876700
H	-6.23175200	1.55437700	0.12953400
H	-6.80889700	0.01649100	0.79009400
H	-5.61414000	0.50611000	2.96646100
H	-5.03327200	2.03730900	2.30408700
C	4.59868700	-2.89783800	0.50090700
C	6.84658500	-2.40376700	-0.48116700
C	4.75272500	-2.01531800	-1.84252600
C	-0.42809000	2.57958100	1.57423000
C	-0.80522100	4.52008300	0.04844000
C	1.41814200	4.24406500	1.21871000
C	4.01620600	1.15225100	-0.56819700
C	2.81780600	1.96240600	-0.81251800
O	5.20503600	1.56658900	-1.03337000
C	6.15544200	0.53829400	-0.63051200
C	5.30315000	-0.51830300	0.13417000
N	3.93990400	0.05081200	0.08357900
N	1.70361900	1.63941500	-0.27072300
C	0.71514100	2.66717800	-0.66209100
C	1.47941400	3.46397600	-1.75421400
O	2.87197500	3.03210000	-1.62285500
C	5.37320800	-1.96034900	-0.43767500
C	0.24171200	3.51153000	0.55503300
H	3.55848200	-2.57852500	0.59126100
H	4.61924100	-3.92378500	0.11528300
H	5.04420200	-2.90607400	1.50346300
H	7.44271600	-1.81855600	-1.19078300
H	6.91078700	-3.45235100	-0.79137500
H	7.31518800	-2.32068400	0.50732000
H	3.69031900	-1.75370500	-1.80462700
H	5.25020800	-1.33315400	-2.54319700
H	4.84144800	-3.02711100	-2.25324300
H	0.27752400	1.83904400	1.96027900
H	-0.82487800	3.15486200	2.41813200
H	-1.25445300	2.03663500	1.11166400
H	-0.38510200	5.23980200	-0.66484000
H	-1.20969300	5.09386900	0.88965500
H	-1.63799700	4.00007300	-0.43871900
H	1.05821200	4.83695100	2.06673400
H	2.15659500	3.53147000	1.60194300
H	1.92830500	4.93063000	0.53235100
H	6.91521500	1.01618700	-0.00910200
H	6.62010400	0.15741200	-1.54129100
H	5.60567300	-0.56403000	1.18714800
H	-0.15795100	2.15602300	-1.07295200

H	1.15811500	3.21209900	-2.76874600
H	1.46411800	4.54554900	-1.62419300
Pd	1.71166700	-0.47626400	0.53877000
C	-0.94413600	-1.57573900	-0.50756100
H	-0.52770700	-0.85094600	-1.21332500
C	-0.10034900	-1.54185900	0.79190400
C	1.01952800	-2.38262900	1.07607200
H	1.26558200	-3.12352700	0.31426800
C	1.35896300	-2.84091500	2.48220400
H	1.17218200	-2.06318100	3.22841200
H	2.41525600	-3.12058100	2.55754500
C	-0.98997900	-2.93791100	-1.20023100
H	-1.35289800	-3.71787700	-0.52638100
H	0.01156500	-3.21392900	-1.54589700
H	-1.64487800	-2.91076700	-2.07989100
H	0.76419700	-3.72269900	2.76422600

INT6

C	3.60603900	0.46237100	1.01735600
C	5.05445800	-1.37405200	0.12615100
C	3.14187000	-1.91353700	1.68183300
C	-3.61231900	0.09770300	-1.22591000
C	-3.68399200	0.86440200	1.16899600
C	-5.21850500	-0.96632400	0.40764500
C	0.62708400	-1.93813800	-0.53760800
C	-0.79309900	-1.99945400	-0.18112400
O	1.25547800	-3.01739000	-1.03250400
C	2.61436800	-2.56405700	-1.31383300
C	2.69289800	-1.13659700	-0.69807700
N	1.28548100	-0.85381100	-0.35789000
N	-1.37398300	-0.98109200	0.33633100
C	-2.76011700	-1.39507300	0.64178600
C	-2.86884900	-2.78066100	-0.04936900
O	-1.49198100	-3.13071300	-0.38327500
C	3.62079900	-0.99943400	0.54243300
C	-3.82110300	-0.34594200	0.23032000
H	2.59454300	0.79572900	1.26460900
H	4.24172400	0.57593100	1.90332300
H	3.99339400	1.13046200	0.23977100
H	5.14909300	-2.42781800	-0.16094600
H	5.74198000	-1.20233300	0.96147500
H	5.39497500	-0.75991100	-0.71650900
H	3.82762900	-1.84160800	2.53295200
H	2.14644600	-1.61634600	2.02670800
H	3.10176300	-2.96781900	1.38161800
H	-3.69202100	-0.74160600	-1.92803600
H	-4.37516900	0.83209000	-1.50674300
H	-2.62835700	0.55950900	-1.35108800
H	-3.88795200	0.57649000	2.20806000
H	-4.40119900	1.64479800	0.89042600
H	-2.67570600	1.28445300	1.11891800
H	-5.98982600	-0.21453400	0.20807600
H	-5.39321400	-1.80267100	-0.27981500

H	-5.36618400	-1.32982900	1.43233800
H	2.74213200	-2.56942600	-2.39924700
H	3.29409700	-3.28759200	-0.86404800
H	3.01667900	-0.40649500	-1.44659300
H	-2.82325300	-1.51625500	1.73187600
H	-3.26558500	-3.56772900	0.59212700
H	-3.43102000	-2.75366700	-0.98679900
Pd	-0.01539000	0.92281600	0.13856400
C	-0.67320200	2.92671700	0.27254000
H	-0.89991000	3.17393900	1.31402800
C	0.67989800	2.78185800	-0.08324500
C	1.80325500	3.39930800	-0.43566900
H	2.68130700	2.81017700	-0.70006100
C	1.96594800	4.89900600	-0.48765200
H	2.76187400	5.24100500	0.18949700
H	1.03714800	5.40235300	-0.20092400
C	-1.70780500	3.42209000	-0.72616800
H	-1.43651600	3.13150000	-1.74581200
H	-1.77964800	4.52015000	-0.69821100
H	-2.70659900	3.02511400	-0.51435000
H	2.24109300	5.24042500	-1.49581400

TS1

C	3.18813600	3.51141000	-1.87774600
C	3.27226100	3.66541400	-0.48247700
C	2.73932600	2.70575500	0.37824600
C	2.12782700	1.58984900	-0.18648300
C	2.03871900	1.43861500	-1.57472300
C	2.56712400	2.39521300	-2.43816000
C	1.43809200	0.46662400	0.50003500
C	1.02564300	-0.50114600	-0.60735900
C	1.28343300	0.20457000	-1.92508700
O	0.92322600	-0.12751000	-3.03970300
O	1.04551600	0.45139300	1.65528800
C	0.21251800	-1.64059800	-0.38946900
C	-0.66818700	-2.10727200	-1.39384100
O	2.88270600	-1.34796400	-1.05128700
O	5.01717900	-1.61468500	-0.68318400
C	6.28670100	-1.26800600	-0.05796500
C	7.29498900	-2.06845300	-0.88590500
C	6.32026800	-1.72090200	1.40485500
C	6.55208900	0.23501900	-0.19803200
C	3.84790100	-1.04635900	-0.26224800
O	3.78543100	-0.33140200	0.75117600
C	0.21335700	-2.32572500	0.95240900
O	-0.78209000	-2.78013100	1.48846000
O	1.44619300	-2.40202300	1.44155100
C	3.05144900	-2.69331900	3.16213900
C	1.58497500	-2.83945800	2.81062900
H	3.61143000	4.27504600	-2.52378600
H	3.76489500	4.54286800	-0.07327600
H	2.81088600	2.79946700	1.45691900

H	2.48503700	2.26382600	-3.51223100
H	-1.23358900	-3.00945100	-1.18596100
H	-0.44882900	-1.88145300	-2.43292200
H	7.24097600	-1.77621000	-1.93933800
H	8.31320900	-1.89055800	-0.52302700
H	7.08044600	-3.13972200	-0.81636600
H	5.61989700	-1.14020900	2.00461000
H	7.33141300	-1.59682700	1.81025300
H	6.05146300	-2.78064600	1.47427100
H	7.55682100	0.47370300	0.16962500
H	6.49305200	0.52865000	-1.25198300
H	5.81875300	0.80952400	0.36942600
H	3.21194700	-2.94831500	4.21554200
H	3.66632800	-3.35539600	2.54470200
H	3.37528900	-1.66609800	2.97878300
H	0.94562900	-2.20438600	3.43158700
H	1.22675700	-3.87065500	2.89183000
C	-0.70771400	3.61221200	-1.07449800
C	-0.51588900	5.04141600	0.97054300
C	-2.78530400	4.39609600	0.07633800
C	-3.98942900	-2.71921100	-2.33061500
C	-6.14427700	-3.26053100	-1.18339000
C	-3.99798200	-3.28242800	0.13681600
C	-3.06002000	1.35173600	1.16703200
C	-4.04002100	0.38997800	0.64100000
O	-3.32545500	2.08586900	2.25019600
C	-2.06213000	2.78515600	2.52790100
C	-1.27074900	2.66823500	1.20545200
N	-1.93869700	1.51395600	0.56301000
N	-3.75087700	-0.34783300	-0.36984100
C	-4.98463700	-1.09683000	-0.71482300
C	-5.92231800	-0.77843300	0.47964400
O	-5.25870400	0.31720800	1.18468500
C	-1.33509400	3.92951200	0.29204300
C	-4.76394700	-2.59940000	-1.00692600
H	-1.22596700	2.78705300	-1.57210700
H	-0.76455100	4.49341700	-1.72358500
H	0.34297700	3.34064700	-0.97270300
H	-0.92251400	5.32184800	1.94960300
H	-0.51545000	5.94189600	0.34667600
H	0.52508000	4.72801700	1.10805400
H	-2.79385000	5.28644000	-0.56135200
H	-3.37909900	3.62698400	-0.42997300
H	-3.29090100	4.65924700	1.01176000
H	-3.05865300	-2.14978600	-2.30137300
H	-3.74544800	-3.76707300	-2.53710000
H	-4.59008200	-2.34294000	-3.16787600
H	-6.72847100	-3.25550700	-0.25557300
H	-6.02022300	-4.30669400	-1.48263800
H	-6.73269100	-2.75937300	-1.96211400
H	-3.83825400	-4.33909500	-0.10499700
H	-3.02157900	-2.82769900	0.31981100

H	-4.55936700	-3.25223000	1.07865100
H	-1.57979900	2.25431700	3.35279600
H	-2.31616600	3.79980700	2.82914600
H	-0.23011900	2.40516500	1.39828900
H	-5.37853300	-0.63752100	-1.63157500
H	-6.91223200	-0.42920300	0.18709800
H	-6.01518200	-1.60353500	1.18928300
Pd	-1.45648700	-0.30888400	-0.59083700

TS2

C	-2.16134300	2.10306300	4.86038600
C	-3.50994700	1.71527300	4.93929200
C	-4.09516000	0.92471200	3.94555700
C	-3.29744100	0.53716100	2.87622600
C	-1.94743800	0.92081400	2.79867200
C	-1.36114300	1.71094400	3.78565700
C	-3.65075700	-0.29418800	1.69358800
C	-2.39295900	-0.40132400	0.86700900
C	-1.35153100	0.38712200	1.56275700
O	-0.19395500	0.64259000	1.18706100
O	-4.72205800	-0.82503000	1.47192400
C	-2.26920700	-1.17288000	-0.24396400
C	-1.00281300	-1.53175600	-0.91433400
C	-3.48848500	-1.87481200	-0.79975200
O	-3.56962800	-3.08255300	-0.80029300
O	-4.36384400	-1.01926400	-1.32121300
C	-6.45924000	-0.45817200	-2.32636500
C	-5.62979500	-1.59195600	-1.76356800
H	-1.73879600	2.71675600	5.64970300
H	-4.10495300	2.03829900	5.78787000
H	-5.13521500	0.62004800	3.99593300
H	-0.31874600	2.00415200	3.71678700
H	-0.23944400	-1.85068900	-0.18201200
H	-1.12701200	-2.37719200	-1.58594900
H	-7.42868400	-0.84313500	-2.65838800
H	-6.63442300	0.30812400	-1.56539600
H	-5.96284700	0.00585700	-3.18493600
H	-5.41697600	-2.36904100	-2.50260500
H	-6.09475100	-2.05758700	-0.89119200
C	1.91884700	-3.93635700	0.47838800
C	3.87878000	-4.49814400	1.93858300
C	4.23524500	-3.33965700	-0.27536000
C	-0.06794100	3.29818400	-1.55527800
C	1.67872500	5.07604000	-1.62077600
C	1.19186900	3.86307500	0.54497100
C	3.44085600	-0.11749200	0.76823800
C	3.25845400	1.10571300	-0.03861900
O	4.56316100	-0.19580400	1.50188300
C	4.47401300	-1.47354600	2.20342700
C	3.15532900	-2.11209200	1.67628400
N	2.59556500	-1.07238900	0.78670900
N	2.20149500	1.36543300	-0.72517900

C	2.38197000	2.68733700	-1.37326100
C	3.83000200	3.06430200	-0.95030700
O	4.25332400	1.98677600	-0.06075000
C	3.30921900	-3.47202900	0.94406900
C	1.29427200	3.72701800	-0.98275700
H	1.50572000	-3.24368500	-0.25986600
H	1.98138400	-4.92771400	0.01727600
H	1.21764000	-4.00237400	1.31930000
H	4.88068400	-4.22382400	2.28786100
H	3.95859000	-5.48110800	1.46296700
H	3.23012800	-4.60330800	2.81634600
H	3.84158900	-2.60897500	-0.98996100
H	5.24972000	-3.03263300	0.00261100
H	4.31842900	-4.30224700	-0.79043200
H	-0.42502400	2.36652000	-1.10823100
H	-0.81543700	4.07432000	-1.35849600
H	-0.01614900	3.15164400	-2.64104200
H	2.61022400	5.48534100	-1.21562800
H	0.89333100	5.81394000	-1.42903000
H	1.79086200	4.98557600	-2.70799800
H	0.45230800	4.62912300	0.80095600
H	0.87738300	2.92090100	1.00415200
H	2.14248300	4.16824800	0.99830300
H	4.45658400	-1.25846300	3.27332400
H	5.37493700	-2.03815700	1.96134700
H	2.44559700	-2.26005800	2.49878000
H	2.32683000	2.53609200	-2.45727300
H	4.53514700	3.09337000	-1.78235000
H	3.90036800	3.98956800	-0.37997400
Pd	0.55592600	0.02821700	-0.80406400
C	-1.78631000	0.11531900	-3.04339900
H	-2.67372500	0.23343000	-2.42864400
C	-0.67431800	-0.40378200	-2.52531100
C	0.72505800	-0.47754900	-2.79592200
H	1.12900500	0.38868000	-3.32415500
C	1.45262300	-1.77306000	-3.07914800
H	2.51968100	-1.67521100	-2.85842100
H	1.35216300	-2.03235700	-4.14214000
C	-1.90505900	0.52098700	-4.48605300
H	-0.96522900	0.38845400	-5.02897600
H	-2.20736500	1.57343700	-4.55851400
H	-2.67959100	-0.06801300	-4.99349900
H	1.05931300	-2.60871500	-2.49467000

TS2'

C	-7.77437900	-0.48356300	-0.77173900
C	-7.77804000	-0.13183700	0.58983200
C	-6.58536100	0.00246400	1.30339600
C	-5.39382300	-0.23334600	0.62346700
C	-5.39026400	-0.57957900	-0.73854200
C	-6.57772100	-0.71015400	-1.45405900
C	-4.00211500	-0.15132000	1.13236500
C	-3.11483000	-0.55763400	-0.01601100

C	-3.99770700	-0.73935300	-1.22300000
O	-3.64942800	-0.99529100	-2.36579700
O	-3.64653200	0.25064600	2.22733000
C	-1.77609500	-0.76640900	0.02653000
C	-0.92767700	-0.90805300	-1.17734000
C	-1.04708100	-0.79500900	1.34282800
O	0.01782500	-0.19160000	1.51841400
O	-1.60332200	-1.57403900	2.23596700
C	-2.05966200	-2.28164800	4.47018400
C	-1.07665600	-1.53123000	3.59898200
H	-8.71938800	-0.57559100	-1.29801400
H	-8.72621200	0.03804400	1.09049900
H	-6.57563100	0.27590300	2.35333700
H	-6.56100600	-0.97578900	-2.50591200
H	-0.09118700	-1.60374000	-1.03935300
H	-1.49920700	-1.21046400	-2.04910100
H	-1.71153100	-2.26759900	5.50793100
H	-3.04345600	-1.80633800	4.42546900
H	-2.15393400	-3.32413100	4.15160600
H	-0.08059400	-1.98257600	3.58769600
H	-0.98527800	-0.48016200	3.88181100
C	2.06151500	-3.70062800	-1.31343500
C	4.08435300	-4.88972300	-0.42986300
C	4.34794900	-2.84559300	-1.88666800
C	0.26231200	3.64741900	0.38639900
C	2.07338300	5.23992800	1.01700800
C	1.71295600	3.13368800	2.37212200
C	3.76930200	-0.45790700	0.54650200
C	3.59152500	0.99996700	0.39799200
O	4.94103300	-0.87312000	1.05553300
C	4.82912400	-2.32588500	1.13819200
C	3.45083100	-2.64331000	0.48790000
N	2.87535300	-1.30752900	0.22034200
N	2.50674300	1.55467900	-0.01599600
C	2.69238000	3.02592800	0.02608100
C	4.18437800	3.15354000	0.44458500
O	4.61608300	1.78502000	0.71494400
C	3.49762400	-3.51551600	-0.79559900
C	1.68469200	3.74968000	0.96421000
H	1.63475500	-2.74112700	-1.61746500
H	2.05051800	-4.36896000	-2.18097800
H	1.41385300	-4.13896500	-0.54409200
H	5.11822100	-4.81714200	-0.07387500
H	4.08860500	-5.54437900	-1.30752700
H	3.49052700	-5.38053600	0.35045500
H	3.93965000	-1.86592000	-2.15695300
H	5.38974900	-2.70727200	-1.57668500
H	4.35839800	-3.46558000	-2.78908500
H	-0.10175000	2.61614500	0.37618300
H	-0.42938500	4.24005800	0.99467900
H	0.21972200	4.03072600	-0.64051000
H	3.05052400	5.40683600	1.48230800

H	1.33741900	5.79338700	1.60864600	C	4.83004800	-0.78464000	4.18601900
H	2.09360500	5.68254300	0.01370600	C	5.26942200	-2.02905500	3.72109200
H	1.03475300	3.68302000	3.03317100	C	4.95500500	-2.46406300	2.42417700
H	1.38843100	2.08838600	2.35260000	C	4.20732800	-1.61964600	1.61914900
H	2.71108400	3.17705000	2.82400300	C	3.76611400	-0.37472700	2.08491200
H	4.88475000	-2.59851200	2.19384700	C	4.06392500	0.05951700	3.36775000
H	5.68455900	-2.74671700	0.60927200	C	3.72378700	-1.80218800	0.20204500
H	2.79498600	-3.14919900	1.20606800	C	2.89042000	-0.63576800	-0.09751400
H	2.54404900	3.41039700	-0.98898000	C	2.95803800	0.29131800	1.00666500
H	4.82719100	3.54550400	-0.34537000	O	2.38688600	1.38991800	1.14276200
H	4.34405600	3.72047700	1.36050800	O	3.96639000	-2.77375000	-0.50891200
Pd	0.79057800	0.44283100	-0.55108700	C	2.46343600	-0.31727200	-1.47065200
C	-1.71038000	1.56327400	-2.21485400	C	1.21540500	-0.58365600	-1.91474000
H	-2.50993000	1.41757300	-1.49363400	C	3.43572100	0.24218800	-2.45340900
C	-0.61295400	0.81459500	-2.13368900	O	3.17275200	0.55414700	-3.60159800
C	0.74984200	0.87012700	**-2.56367000**	O	4.66283100	0.37454700	-1.91105100
H	1.13452000	1.88078200	-2.71681400	C	6.98212100	0.88491600	-1.99469100
C	1.38488600	-0.17784800	-3.44845000	C	5.69043200	0.86730900	-2.78931300
H	2.46780700	-0.21512400	-3.29508700	H	5.08730300	-0.46999400	5.19400300
H	1.20484600	0.07069300	-4.50360000	H	5.86229800	-2.66407500	4.37401200
C	-1.94733900	2.58858600	-3.28561000	H	5.28675200	-3.42782600	2.04916400
H	-1.07306000	2.71802900	-3.92927600	H	3.71458500	1.02731500	3.71554900
H	-2.20259900	3.55622300	-2.83586200	H	0.94999300	-0.37954400	-2.94573900
H	-2.79596100	2.29432600	-3.91637300	H	7.80328600	1.25233400	-2.61992400
H	0.97718300	-1.17537800	-3.26707400	H	6.89045300	1.53819100	-1.12107900
				H	7.23253200	-0.12167900	-1.64549000

TS3

H	5.75234100	0.21299800	-3.66497900
H	5.40695700	1.86473900	-3.14206900
C	-4.32501500	-2.95587300	-0.95727200
C	-6.64302800	-2.67029000	-0.06276300
C	-4.64854800	-2.31383000	1.44808000
C	0.40555900	2.71590600	-1.26621600
C	0.67954600	4.56081300	0.39211900
C	-1.50846600	4.29135100	-0.85207200
C	-4.01900800	0.99320600	0.53312900
C	-2.87532500	1.82939000	0.93075600
O	-5.24624300	1.29841800	0.96726200
C	-6.12251300	0.27468700	0.40669100
C	-5.17728800	-0.65938400	-0.40636000
N	-3.85152100	-0.02890800	-0.22511500
N	-1.71317300	1.57221800	0.46134900
C	-0.79392600	2.62486200	0.94757300
C	-1.65398500	3.31464200	2.03973600
O	-3.02521600	2.85101000	1.78082300
C	-5.19579700	-2.15230800	0.02120100
C	-0.32260000	3.55721600	-0.20669900
H	-3.29675000	-2.58889400	-0.95483300
H	-4.31713500	-4.01511900	-0.67637000
H	-4.71250400	-2.88234100	-1.98084000
H	-7.30662000	-2.18750700	0.66358700
H	-6.66588400	-3.74574700	0.14296400
H	-7.06280700	-2.51473400	-1.06418100
H	-3.60162500	-1.99740800	1.49996900
H	-5.21989900	-1.73056000	2.18070600
H	-4.70180200	-3.36400500	1.75452100
H	-0.26517900	1.98408800	-1.72721100
H	0.79468700	3.36580500	-2.05851300
H	1.24203600	2.17803400	-0.81497600
H	0.22515800	5.20291200	1.15718600
H	1.06356300	5.21743300	-0.39628100
H	1.52846200	4.03166200	0.83778500
H	-1.14736600	4.94005200	-1.65723400
H	-2.21842900	3.58322500	-1.29472100
H	-2.05318300	4.92535700	-0.14224400
H	-6.86795200	0.78209800	-0.20794600
H	-6.61630100	-0.21529800	1.24670200
H	-5.42313800	-0.61572700	-1.47376000
H	0.09460500	2.14843500	1.37052200
H	-1.39708000	2.99473900	3.05312400
H	-1.66916800	4.40200000	1.98883000
Pd	-1.57698400	-0.39571700	-0.58290500
C	0.54391900	-1.04215200	0.39773000
H	0.77747500	-0.03144800	0.69746000
C	0.29856600	-1.26245600	-1.00440300
C	-0.74012000	-2.16296600	-1.41253000
H	-0.99402000	-2.96028000	-0.71412200
C	-0.98490400	-2.51210600	-2.86108100
H	-0.86104300	-1.64972600	-3.52204300

H	-2.00125300	-2.89587800	-2.99732600
C	0.33634300	-2.03349000	1.50246200
H	0.09895200	-3.03310000	1.13075700
H	-0.45723000	-1.71137400	2.18736300
H	1.26009800	-2.09940900	2.09100300
H	-0.28795200	-3.29293000	-3.19707000

***ent*-TS3**

C	0.82928600	2.67990700	0.57154600
C	0.49102400	4.75411200	-0.78714600
C	0.47178400	2.51716700	-1.93416900
C	-4.48104800	-2.33924900	1.72419700
C	-6.67653000	-2.60955800	0.54867100
C	-6.01731900	-0.36862600	1.50610800
C	-3.10717800	1.87144700	-0.96424200
C	-3.99847800	0.70154000	-1.00476100
O	-3.50869200	3.03167600	-1.50206800
C	-2.34471400	3.91396000	-1.37967500
C	-1.42042200	3.18697900	-0.37434000
N	-1.96953200	1.80453200	-0.38052800
N	-3.70118900	-0.37005900	-0.36606300
C	-4.82761500	-1.30900400	-0.53890000
C	-5.73024700	-0.57587100	-1.57248900
O	-5.14214000	0.75580700	-1.69792000
C	0.09864800	3.27215000	-0.64662300
C	-5.50821700	-1.64475900	0.81676600
H	0.41744800	1.70826700	0.84956800
H	1.89400200	2.53880200	0.36252600
H	0.72464300	3.33812400	1.44276100
H	0.03545700	5.22400200	-1.66788200
H	1.57737600	4.83789600	-0.89470000
H	0.19908600	5.32977700	0.09986900
H	0.05615800	1.50586700	-1.93272600
H	0.10041700	3.02911000	-2.83080600
H	1.56022500	2.43582700	-2.01526900
H	-3.63162800	-1.68509900	1.93531300
H	-4.94541500	-2.62277600	2.67531300
H	-4.09321900	-3.25017500	1.25326600
H	-7.46399500	-2.15407000	-0.06291400
H	-7.13576500	-2.91379800	1.49520900
H	-6.33195700	-3.51708100	0.03825300
H	-6.50813200	-0.62443500	2.45105900
H	-5.18987100	0.31160300	1.73296600
H	-6.74903200	0.17282600	0.89494400
H	-2.70288200	4.88464700	-1.03931400
H	-1.90661700	4.00718600	-2.37730700
H	-1.59391300	3.57696800	0.63830500
H	-4.43284600	-2.23995000	-0.95883800
H	-5.70960800	-1.03393000	-2.56396800
H	-6.76385500	-0.44211100	-1.25425800
Pd	-1.50175700	-0.41439900	0.25811000
C	5.61061100	-1.53357700	-3.50604700
C	5.36541600	-2.86433400	-3.15352600

C	4.62386300	-3.17895600	-2.00293900
C	4.15686300	-2.13260200	-1.22433800
C	4.40290900	-0.79844800	-1.57737500
C	5.12210500	-0.47894400	-2.71783800
C	3.32671400	-2.16011500	0.03798700
C	3.08900500	-0.76775500	0.38540000
C	3.74222500	0.10107800	-0.56560100
O	3.72299300	1.34005100	-0.63187900
O	2.89691400	-3.18168000	0.57769600
C	2.43637900	-0.33260700	1.62549000
C	1.13979600	-0.62845700	1.85519400
C	3.16818700	0.47509300	2.64178900
O	2.63860800	1.05950600	3.57158200
O	4.48811900	0.51788800	2.37797300
C	5.08791100	2.86715600	2.48439000
C	5.25892800	1.49019200	3.11055700
H	6.18561000	-1.31524300	-4.40213200
H	5.75320200	-3.66342300	-3.77987800
H	4.41938500	-4.20716600	-1.71903800
H	5.29905900	0.55963800	-2.98191600
H	0.67119400	-0.39748800	2.80506000
H	5.78049100	3.58247400	2.94291200
H	4.06604900	3.22647400	2.63550000
H	5.28007300	2.81585900	1.40859400
H	6.28886800	1.13510800	3.03018400
H	4.94984900	1.48073000	4.15893100
C	0.80628500	-0.79943900	-0.54880100
H	1.17611800	0.21221200	-0.61460400
C	0.40068800	-1.25125100	0.74600300
C	-0.60326400	-2.25573700	0.87537900
H	-0.75034900	-2.92181200	0.02728200
C	-0.91563200	-2.87424400	2.21497000
H	-0.98526500	-2.12811700	3.01218400
H	-1.85735900	-3.42865200	2.18563400
C	0.71649300	-1.55194600	-1.83560200
H	0.32379000	-2.56223400	-1.71073300
H	0.10576100	-1.01306000	-2.56934200
H	1.72689200	-1.63162200	-2.25829600
H	-0.11671500	-3.57788400	2.48811100

TS3'

C	6.04966000	-2.90698300	2.05130400
C	6.33035400	-3.27284600	0.73058800
C	5.74248400	-2.58910100	-0.34521300
C	4.88215000	-1.54153900	-0.05765800
C	4.59989900	-1.17679300	1.26461200
C	5.17287300	-1.84762000	2.33334000
C	4.10869900	-0.64198200	-0.98887300
C	3.29353300	0.21431800	-0.14231100
C	3.63809600	-0.01662300	1.25669100
O	3.21819400	0.56365400	2.26063800
O	4.15160400	-0.69482300	-2.21932500
C	2.54420000	1.39847000	-0.59507200

C	1.24222500	1.51602400	-0.26323100
C	3.19083400	2.51641100	-1.33682500
O	2.58880800	3.44245100	-1.85313800
O	4.53116300	2.38309600	-1.36275700
C	5.50656500	4.61726200	-1.19878000
C	5.26241600	3.39886400	-2.07658000
H	6.51962200	-3.44957000	2.86747200
H	7.01457100	-4.09510700	0.53795800
H	5.95127800	-2.85999500	-1.37615000
H	4.94679500	-1.55028900	3.35327500
H	0.67144800	2.41324900	-0.47446900
H	6.13179700	5.34412500	-1.73036300
H	4.55763300	5.09656500	-0.94381400
H	6.01809700	4.32985000	-0.27447200
H	6.19790800	2.90995200	-2.35781800
H	4.70575600	3.66691700	-2.97814800
C	-1.26895700	-3.72809600	0.20120700
C	-2.63758300	-5.11578200	-1.36325500
C	-3.75501500	-3.82796800	0.49748200
C	-2.08683400	3.45833800	1.19552900
C	-4.29571200	4.63393700	1.19612900
C	-3.49179400	3.43654000	-0.87704500
C	-3.97158300	-0.86590300	-0.86267000
C	-4.36832600	0.32933300	-0.10710200
O	-4.87058800	-1.51422400	-1.61309900
C	-4.10576500	-2.56297300	-2.28632100
C	-2.76186200	-2.61314300	-1.51348100
N	-2.77189200	-1.31726600	-0.79759400
N	-3.55883100	0.89132200	0.71201500
C	-4.22786700	2.12651400	1.18333200
C	-5.68607500	1.93181500	0.68267300
O	-5.59467400	0.84249000	-0.28576500
C	-2.61553300	-3.81793600	-0.53541600
C	-3.52558600	3.41362400	0.66002400
H	-1.18889600	-2.79773400	0.77253000
H	-1.16670100	-4.57024200	0.89481800
H	-0.42844000	-3.76798200	-0.49971700
H	-3.59936900	-5.28253100	-1.86122300
H	-2.45747300	-5.97632300	-0.71032900
H	-1.85361300	-5.10981800	-2.12997400
H	-3.71787300	-2.93724400	1.13350600
H	-4.74456600	-3.87531600	0.02862600
H	-3.65899000	-4.70351200	1.14817000
H	-1.50187400	2.62450300	0.80334300
H	-1.60046600	4.39311500	0.89595800
H	-2.06628200	3.40165700	2.29075200
H	-5.32045200	4.68921900	0.81121600
H	-3.78661100	5.55566600	0.89556900
H	-4.34410600	4.62203400	2.29179400
H	-3.01247900	4.35725800	-1.22574200
H	-2.91234000	2.59416600	-1.26907400
H	-4.49484400	3.40208300	-1.31832500

H	-3.98605400	-2.25747200	-3.32933500
H	-4.69797100	-3.47502800	-2.23559800
H	-1.91372600	-2.62402600	-2.20412800
H	-4.18942700	2.14140100	2.27702800
H	-6.37080200	1.60902400	1.47169000
H	-6.10650700	2.79081000	0.16186100
Pd	-1.37838200	-0.13827500	0.47936000
C	0.11258700	0.58181500	1.79097300
H	0.01901300	1.62503400	2.08690800
C	0.71429700	0.37866300	0.51752300
C	1.13226500	-0.90550600	0.05707900
H	1.21184800	-1.70001200	0.79499100
C	1.16331100	-1.34672600	-1.37768000
H	1.25346500	-0.50250500	-2.06180400
H	0.23935600	-1.88872500	-1.61248000
C	0.19901900	-0.36681600	2.96629200
H	0.22267100	-1.41942600	2.67058700
H	-0.65629200	-0.22892600	3.63768300
H	1.11894500	-0.15304500	3.52356200
H	1.99867300	-2.02627400	-1.56385400

TS3-RE

C	0.64248100	-3.98006500	-1.75117200
C	0.74313500	-2.92879700	-2.67546100
C	0.16050300	-1.68321000	-2.41910500
C	-0.51133200	-1.50528700	-1.21382300
C	-0.61160900	-2.55642000	-0.29480200
C	-0.03975800	-3.80045200	-0.54543400
C	-1.29725600	-0.30844800	-0.75269100
C	-1.86049500	-0.68519900	0.65014300
C	-1.45100400	-2.14326400	0.85643000
O	-1.74681700	-2.83428100	1.81700000
O	-1.79526400	0.51251900	-1.55380900
C	-3.30039300	-0.29067800	0.72280600
C	-3.61355800	0.74275000	1.52512300
C	-4.27667900	-0.89839800	-0.20744700
O	-5.42905400	-0.54497300	-0.35965800
O	-3.70723800	-1.92951100	-0.87126000
C	-3.54822600	-3.50451900	-2.63721000
C	-4.48247200	-2.55770700	-1.90680100
H	1.09332900	-4.94084100	-1.98229100
H	1.27387800	-3.09166500	-3.60972000
H	0.20972500	-0.87317200	-3.14043800
H	-0.14717100	-4.60442100	0.17635100
H	-4.60272600	1.18470100	1.53405800
H	-4.08650700	-4.02002300	-3.44001900
H	-2.70864400	-2.95384400	-3.07257000
H	-3.14237800	-4.25451500	-1.95106100
H	-5.32896500	-3.07973900	-1.44788400
H	-4.88347600	-1.78006500	-2.56380600
C	-1.07326600	3.60075900	-0.37790600
C	-0.42310100	4.66092700	-2.54165400
C	1.19309600	4.66355400	-0.59973100

C	2.49972400	-2.36463800	2.13944300	H	2.65146600	-2.04698200	3.17869600
C	4.92228800	-2.96938900	1.86313000	H	5.84514000	-2.84651000	1.28352300
C	3.47359200	-2.54233400	-0.15650000	H	4.66387000	-4.03383200	1.84351000
C	2.49161900	1.52686200	-1.02465900	H	5.13702600	-2.69603000	2.90351100
C	3.39382100	0.74874200	-0.15971100	H	3.19150700	-3.59891100	-0.19643600
O	2.99869900	2.07430400	-2.14144000	H	2.63839600	-1.97048400	-0.56998400
C	1.85531200	2.63752000	-2.85405500	H	4.34339200	-2.40954000	-0.81030600
C	0.66663200	2.51510700	-1.86363200	H	1.71857600	2.04740100	-3.76401200
N	1.24117300	1.67693600	-0.78891100	H	2.11795000	3.66066600	-3.12180700
N	3.03071500	0.21992700	0.94266700	H	-0.16669200	1.95030100	-2.29245100
C	4.15417500	-0.62734400	1.38333000	H	4.37990100	-0.39866100	2.43046400
C	5.31707300	-0.18726800	0.45490300	H	6.01803200	0.49462800	0.94593300
O	4.65599500	0.55155800	-0.61023100	H	5.86995500	-1.00566500	-0.00662600
C	0.10420000	3.86522400	-1.33324100	Pd	0.05849700	0.62222600	0.63572200
C	3.76429800	-2.13208000	1.29568300	C	-1.47174500	0.09426300	2.37776000
H	-0.74380100	3.08981700	0.53236900	H	-1.92284800	-0.81716700	2.77538500
H	-1.53553400	4.55243000	-0.08756400	C	-2.50613300	1.17925300	2.36359700
H	-1.82975200	2.96420200	-0.84546500	C	-2.41313500	2.34845900	3.02625100
H	0.36905100	4.92244900	-3.25322200	H	-1.51282900	2.53975700	3.60717600
H	-0.87712800	5.59854700	-2.20284100	C	-3.42604400	3.45202700	3.01960900
H	-1.19103300	4.09186300	-3.07847800	H	-3.74451700	3.70417300	4.03984100
H	1.56130700	4.10982700	0.27080700	H	-4.31516100	3.20312000	2.43517700
H	2.04986200	4.89477300	-1.24449700	C	-0.20588900	0.30755500	3.22182100
H	0.78625400	5.61641400	-0.24338600	H	0.40479600	1.18111800	2.95607600
H	1.65630900	-1.80452100	1.72854800	H	0.43027500	-0.57914600	3.21200100
H	2.23547200	-3.42792000	2.14564700	H	-0.53942600	0.45942400	4.26009700

H	-2.99064100	4.36564600	2.59143700

TS3-inner-*syn*

C	0.88551800	4.27787300	-3.42288600
C	2.12079400	4.81046800	-3.03941700
C	2.83362400	4.26854400	-1.95793100
C	2.27710600	3.18987400	-1.29182800
C	1.04069900	2.65708000	-1.67550800
C	0.32462400	3.18938000	-2.73665900
C	2.78401900	2.40033000	-0.11839000
C	1.74602900	1.41815900	0.21917900
C	0.69691400	1.52082300	-0.76491100
O	-0.38326100	0.88938300	-0.86710100
O	3.86403800	2.56981400	0.44344000
C	2.10372800	0.24717800	1.05431600
C	1.42652000	-0.06209900	2.18652200
C	3.21357900	-0.59828600	0.55869300
O	3.59097200	-0.61944600	-0.60173100
O	3.76682000	-1.35986300	1.53456100
C	6.17345500	-1.30750800	1.12433000
C	4.91112900	-2.15494200	1.15190700
H	0.35449600	4.71358200	-4.26481900
H	2.53276800	5.65279600	-3.58833100
H	3.79395000	4.67014600	-1.64879400
H	-0.63416500	2.76827700	-3.02395900
H	1.66299000	-0.95517700	2.75301700
H	7.04040300	-1.93570300	0.88926400
H	6.09045400	-0.52811200	0.36271600
H	6.34101500	-0.83304300	2.09642400
H	4.96201000	-2.93590700	1.91423400
H	4.71953500	-2.61385500	0.17938900
C	1.66055900	-4.04125700	0.25178500
C	2.58998300	-3.81633000	-2.07370500
C	0.35449100	-4.92989500	-1.68958400
C	-4.13561900	1.83648300	1.36747400
C	-6.25012500	1.24427500	0.18749500
C	-4.11346800	1.55513600	-1.12891300
C	-1.50627400	-1.95840700	-1.25938200
C	-2.83316600	-1.55418700	-0.75587100
O	-1.31482300	-1.84444700	-2.59247600
C	0.11950300	-2.03888400	-2.77747700
C	0.64283100	-2.42021600	-1.36310200
N	-0.54338500	-2.28386800	-0.49670400
N	-2.98254200	-0.78274000	0.25847800
C	-4.42440800	-0.47226200	0.37742600
C	-5.05735200	-1.40076200	-0.69717700
O	-3.92467600	-1.95717800	-1.42200100
C	1.30479600	-3.81397500	-1.22808600
C	-4.72279700	1.04404200	0.18761300
H	0.75687700	-4.07776300	0.86655100
H	2.20241500	-4.98710700	0.37131300
H	2.29177700	-3.23629600	0.63937500
H	2.37326100	-3.70200100	-3.14298500

H	3.12840600	-4.76315000	-1.94924200
H	3.25350700	-2.99667100	-1.77658200
H	-0.57483400	-4.91511200	-1.11000600
H	0.09548300	-4.84315400	-2.75110000
H	0.82505300	-5.90959700	-1.54838100
H	-3.04689900	1.72070400	1.41532900
H	-4.36928100	2.90172900	1.25655300
H	-4.55528800	1.49440900	2.32195900
H	-6.73833300	0.77828700	-0.67547300
H	-6.48413600	2.31366300	0.15154200
H	-6.70514400	0.83484500	1.09817100
H	-4.37209900	2.61000800	-1.27434800
H	-3.02239200	1.47013200	-1.12040500
H	-4.49242800	1.00350000	-1.99809400
H	0.53083000	-1.09385300	-3.13599400
H	0.24995600	-2.81214100	-3.53642200
H	1.36944600	-1.67522700	-1.02991600
H	-4.75545700	-0.75971900	1.38125600
H	-5.61466300	-2.23642300	-0.26693500
H	-5.68168500	-0.88542800	-1.42647200
Pd	-1.18942100	0.10582600	1.14223200
C	0.47877100	2.18585600	2.26330200
H	-0.43419900	2.77333300	2.15361900
C	0.28245200	0.79719400	2.52466400
C	-0.94755900	0.33813700	3.17223900
H	-1.58969400	1.13615600	3.55365500
C	-0.96265100	-0.90144000	4.03631400
H	-1.99457000	-1.24580100	4.16753900
H	-0.54865400	-0.70834400	5.03763800
C	1.67443500	2.99403500	2.64134200
H	2.60532200	2.42885000	2.64082100
H	1.48323700	3.37290400	3.66090500
H	1.79340500	3.87037100	1.99781200
H	-0.39322600	-1.71842900	3.58389900

TS3-inner-*anti*

C	-1.15261000	4.51553700	-2.67498700
C	-0.16790100	5.46797100	-2.39971100
C	0.94439200	5.14227300	-1.60465900
C	1.03457500	3.85172900	-1.11333000
C	0.04564100	2.89672200	-1.38733300
C	-1.05775300	3.20956800	-2.16480800
C	2.06501200	3.22106300	-0.21111500
C	1.61350200	1.84831900	0.03048800
C	0.42207900	1.60769700	-0.71607600
O	-0.28046500	0.56949500	-0.75252400
O	3.04253300	3.79038800	0.26622500
C	2.36968400	0.84163300	0.80851300
C	1.89223700	0.40143700	2.00373600
C	3.53326500	0.20084200	0.16453500
O	3.73689500	0.19528700	-1.03933600
O	4.34311800	-0.42522100	1.05820600
C	6.62974400	-0.05556100	0.27557200

C	5.52016000	-1.06573500	0.52233200
H	-2.00318200	4.78972900	-3.29335000
H	-0.26558900	6.47144600	-2.80470600
H	1.71412400	5.87301700	-1.37472400
H	-1.81899200	2.46332200	-2.37170200
H	2.38960800	-0.40073500	2.53785700
H	7.53775900	-0.57133300	-0.05766200
H	6.33014600	0.65570100	-0.49817900
H	6.86001700	0.49591900	1.19262600
H	5.80013200	-1.79437700	1.28708500
H	5.25552000	-1.59369600	-0.39657700
C	2.23130600	-3.31086300	0.46360600
C	3.12370200	-3.39911600	-1.88312900
C	1.11615000	-4.78791100	-1.22298800
C	-4.09543000	0.44254100	2.08662400
C	-6.09359800	0.56242700	0.58763000
C	-3.81297000	1.19919100	-0.29395400
C	-1.26169800	-2.13226900	-1.44549400
C	-2.64014200	-1.88343200	-0.99039600
O	-1.01840900	-2.19252900	-2.76114900
C	0.43851100	-2.24002900	-2.88352100
C	0.95688400	-2.25385300	-1.41808800
N	-0.28352800	-2.18903900	-0.62556800
N	-2.92167300	-1.56610400	0.21728800
C	-4.36055500	-1.22895400	0.24185400
C	-4.84233000	-1.61106200	-1.18682900
O	-3.62124800	-1.92841000	-1.91025300
C	1.85036700	-3.45354900	-1.02078100
C	-4.58692200	0.25617800	0.64304700
H	1.34063600	-3.33517100	1.09986000
H	2.89089100	-4.13333400	0.76510600
H	2.75599800	-2.37087500	0.65546900
H	2.90567100	-3.57470000	-2.94362400
H	3.83443800	-4.17023400	-1.56448100
H	3.61013500	-2.42119300	-1.79714800
H	0.20511400	-4.82743200	-0.61592600
H	0.83547200	-4.95437700	-2.26950500
H	1.75955200	-5.62238500	-0.92242700
H	-3.03946600	0.17750400	2.16674700
H	-4.22054700	1.48478600	2.40227000
H	-4.66110200	-0.19017100	2.78220500
H	-6.49626000	0.49875600	-0.42965600
H	-6.28175700	1.58011600	0.94697800
H	-6.66403100	-0.12653800	1.22304100
H	-3.98150000	2.24138000	0.00012400
H	-2.73625400	1.00713600	-0.26396300
H	-4.14435700	1.10004100	-1.33526000
H	0.74544300	-1.35017400	-3.43423100
H	0.68392500	-3.13445200	-3.45835100
H	1.50922900	-1.33262700	-1.21410500
H	-4.85174400	-1.85642200	0.99433000
H	-5.47723300	-2.50057300	-1.20251600

H	-5.33749200	-0.80317000	-1.72639900
Pd	-0.61272100	-0.92406600	1.20744900
C	0.42446800	2.30056700	2.12046800
H	1.30933900	2.92767900	2.11719500
C	0.61022700	0.95810000	2.46051700
C	-0.44237300	0.11848800	2.97835300
H	-1.33311900	0.66758600	3.28666700
C	-0.12327800	-1.02912000	3.92282800
H	-0.99557700	-1.68035300	4.04786600
H	0.14479100	-0.64926300	4.92052400
C	-0.88144500	3.01704000	2.03775400
H	-0.87951400	3.66667200	1.15360000
H	-1.03079300	3.67246400	2.90768800
H	-1.73062200	2.33620500	1.95390300
H	0.71600700	-1.64211100	3.57536800

TS4

C	4.15718600	-2.47000600	-0.74173400
C	5.51257400	-2.71930600	1.35331100
C	5.97519600	-0.81742100	-0.24411400
C	-0.06717700	3.81676100	1.19323800
C	-0.97698200	3.04365500	-1.00487300
C	-0.42986000	5.46506100	-0.68565800
C	3.39936000	1.29896400	0.86136400
C	2.81365900	2.51374400	0.28669600
O	4.07063200	1.35524400	2.02174100
C	4.40358900	-0.02956200	2.33835700
C	3.84662200	-0.85790100	1.14331900
N	3.22067600	0.16824600	0.28904200
N	1.91560700	2.42394400	-0.62448300
C	1.42950000	3.80027400	-0.87716800
C	2.51865200	4.65948200	-0.18410900
O	3.19736900	3.72621100	0.71344500
C	4.88828700	-1.70978100	0.37497200
C	-0.01123800	4.02679000	-0.32994500
H	3.68203600	-1.77707000	-1.44102300
H	4.86008100	-3.10116000	-1.29739000
H	3.37536900	-3.11862900	-0.32866400
H	6.08089600	-2.22856000	2.15244800
H	6.20425100	-3.38119300	0.82110000
H	4.74252800	-3.34651400	1.81901500
H	6.70305100	-1.43276000	-0.78387900
H	5.53745100	-0.10962800	-0.95541500
H	6.52771100	-0.24673600	0.51198600
H	0.60270000	4.49426400	1.73591600
H	-1.08346000	4.00786700	1.55298300
H	0.18104300	2.78535400	1.45504800
H	-0.92674800	3.12426800	-2.09641100
H	-2.00709300	3.25537000	-0.70244200
H	-0.74960800	2.01644000	-0.72265900
H	-1.45892100	5.64310800	-0.35514700
H	0.20072600	6.22198100	-0.20420000
H	-0.39466700	5.63273000	-1.76926600

H	3.92721400	-0.27016800	3.29107800
H	5.48725100	-0.08429100	2.45170300
H	3.04984400	-1.53008900	1.48483900
H	1.41920300	3.96947500	-1.95785800
H	3.26992200	5.04086100	-0.88202600
H	2.13583800	5.47500100	0.42706800
Pd	1.61612500	0.32719700	-1.35346500
C	0.00482200	0.07189200	-2.60219100
H	-0.89144200	0.20603700	-1.99522200
C	0.67972000	-1.18732800	-2.48377300
C	0.94094700	-2.06596500	-3.48969800
H	0.39532000	-1.93020800	-4.42603900
C	1.73627600	-3.32450300	-3.39995200
H	1.05014300	-4.16067700	-3.18929900
H	2.49110300	-3.29146400	-2.61220300
C	-0.02352500	0.81060700	-3.92178900
H	0.93280600	0.73925800	-4.44744300
H	-0.26095100	1.87062100	-3.78141500
H	-0.80228500	0.38807900	-4.57682100
H	2.23295300	-3.54653300	-4.35188400
C	-0.58946800	-3.49630500	4.40684500
C	0.49084400	-2.59811400	4.38781700
C	0.57296200	-1.60315300	3.41279100
C	-0.43588700	-1.54359900	2.45407000
C	-1.51180200	-2.43889100	2.47137400
C	-1.60767300	-3.41983400	3.45402100

C	-0.59455400	-0.59970000	1.32961800
C	-1.71037700	-1.16042900	0.41260700
C	-2.48318400	-2.10077500	1.38900000
O	-3.62508200	-2.49593900	1.30853800
O	0.04088800	0.42201000	1.14232300
C	-1.12719000	-2.04213500	-0.65755700
C	0.26560000	-2.19680100	-0.84570400
O	-2.51051300	-0.10329600	-0.14800700
O	-3.94312900	1.49789500	0.09209800
C	-4.88755100	2.35891400	0.82473900
C	-5.60169700	3.09563500	-0.30725800
C	-4.12601300	3.33884200	1.71912200
C	-5.86571900	1.48770100	1.61484200
C	-3.16060200	0.64677200	0.75539200
O	-3.02569200	0.54551000	1.96122300
C	-1.95986300	-2.71729400	-1.62942900
O	-1.52592000	-3.45814600	-2.51360100
O	-3.28092200	-2.43122700	-1.50455400
C	-5.58184800	-2.75796500	-1.93125400
C	-4.17779000	-3.14994500	-2.35543200
H	-0.63448300	-4.25494100	5.18309700
H	1.26182800	-2.67643100	5.14913700
H	1.38634600	-0.88343500	3.39672900
H	-2.45626800	-4.09626400	3.46912200
H	0.58265600	-3.15397700	-1.24003200
H	0.92776000	-1.83319100	-0.06843700

H	-6.11010700	2.38565500	-0.96645600
H	-6.34477000	3.78536800	0.10578600
H	-4.88570800	3.66948500	-0.90423200
H	-3.56295500	2.80999100	2.48909300
H	-4.83699900	4.01774900	2.20253500
H	-3.43410900	3.94106800	1.12125200
H	-6.64835600	2.11936600	2.04878000
H	-6.33992300	0.75729200	0.95133500
H	-5.35720100	0.95391400	2.41885700
H	-6.32713200	-3.29674400	-2.52731900
H	-5.73207300	-2.99133000	-0.87291500
H	-5.73691600	-1.68233800	-2.06667000
H	-3.97546100	-2.89309800	-3.40168500
H	-4.00195100	-4.22591900	-2.24377900

***t*-BuO$^-$**

C	-0.00002400	0.00000400	0.16257600
C	0.71307900	1.27373600	-0.43753200
C	-1.45961300	-0.01935700	-0.43760600
C	0.74659700	-1.25439800	-0.43751000
O	-0.00007300	0.00001700	1.48569200
H	1.74489800	1.30501800	-0.05990300
H	0.20032400	2.16973800	-0.06003300
H	0.74403000	1.32884600	-1.54171800
H	-1.97918800	-0.91139200	-0.06004200
H	-2.00261600	0.85861800	-0.06006400
H	-1.52274800	-0.02020700	-1.54179500
H	0.25790000	-2.16364500	-0.05978400
H	1.77895600	-1.25819000	-0.06006300
H	0.77879300	-1.30883100	-1.54169600

***t*-BuOH**

C	-0.00557300	-0.00003800	0.01546600
C	0.68402700	1.26508500	-0.51257100
C	-1.48961300	-0.00369400	-0.35176300
C	0.69032200	-1.26171600	-0.51245100
O	0.02004500	0.00020800	1.45188500
H	1.74252800	1.27966800	-0.22123800
H	0.20344400	2.15757500	-0.09916700
H	0.64013100	1.31885500	-1.60638800
H	-1.97883400	-0.89202300	0.06042000
H	-1.98305900	0.88169100	0.06169200
H	-1.62107400	-0.00326700	-1.43889200
H	0.21444200	-2.15655200	-0.09856900
H	1.74908300	-1.27102200	-0.22183900
H	0.64608300	-1.31612500	-1.60625500
H	0.95191800	0.00172000	1.72307100

OBoc$^-$

O	2.51289400	-0.88926200	-0.00012900
O	0.28264700	-0.77738800	-0.00096100
C	-0.93957900	-0.05090300	0.00001200
C	-2.02094100	-1.14266000	-0.00063300
C	-1.08062300	0.81522200	-1.26494300
C	-1.07977500	0.81298600	1.26656800

C	1.58169000	-0.07901900	-0.00025200
O	1.54645500	1.17071700	0.00007300
H	-1.91295400	-1.77924900	0.88520600
H	-3.02980500	-0.70704800	-0.00026400
H	-1.91303000	-1.77798400	-0.88738800
H	-0.27352900	1.54895700	-1.27609500
H	-2.05555300	1.32338000	-1.29413500
H	-0.99493400	0.18080700	-2.15633300
H	-2.05474700	1.32094000	1.29749400
H	-0.99321200	0.17700400	2.15675800
H	-0.27283200	1.54689600	1.27838500

11.3 The absolute configuration of 20

20-9*S*-1

Zero-point correction = 0.589736 (Hartree/Particle)

Thermal correction to Energy = 0.624031

Thermal correction to Enthalpy = 0.624031

Thermal correction to Gibbs Free Energy = 0.52202

E(elec) = -1801.369014

C	2.75452700	0.67412800	3.46012500
C	2.23399400	-0.61919300	3.55391300
C	1.64399200	-1.24666600	2.44942800
C	1.60591600	-0.53624300	1.25746000
C	2.10538200	0.76790700	1.15441300
C	2.68175300	1.38336100	2.25296200
N	1.11647400	-0.95364100	0.00424600
C	1.29034900	0.01916000	-0.94608800
C	1.87837700	1.26069100	-0.25717200
C	0.92597000	2.46843600	-0.27397200
C	1.61605400	3.61511700	-0.41856300
C	3.02618000	3.35838700	-0.67411500
C	3.13076100	1.87081400	-0.97928600
O	0.96485100	-0.06801900	-2.12807100
C	0.47884300	-2.22648000	-0.27532800
C	-0.99201700	-2.28930200	0.09902300
C	-1.57383700	-1.41332400	1.01955300
C	-2.92454900	-1.53002500	1.35041300
C	-3.70952300	-2.52170600	0.76609000
C	-3.13516400	-3.40184300	-0.15356700
C	-1.78676200	-3.28360400	-0.48463500
C	4.05106400	4.21665500	-0.64971400
O	4.39140800	1.37043400	-0.62567800
C	4.88116600	0.29859400	-1.42622300
C	4.43130700	-1.07004900	-0.96886700
C	3.91037500	-1.99313100	-1.88047700
C	3.57003300	-3.28202600	-1.46666000
C	3.73771900	-3.65212400	-0.13118100
C	4.23691600	-2.72628600	0.78878100
C	4.58411100	-1.44344500	0.37145600
C	-0.55058200	2.41054700	-0.02862500

N	-1.20218700	1.52356900	-0.84897600
O	-1.09369300	3.11945100	0.81213200
C	-2.55185300	1.13518900	-0.83110100
C	-2.90982100	0.11397800	-1.75648800
C	-4.18668400	-0.37922300	-1.78746100
C	-5.18034800	0.10759200	-0.89699600
C	-4.81880900	1.14011100	0.02627200
C	-3.49501200	1.64998600	0.03702300
C	-6.50034000	-0.40861200	-0.87664400
C	-7.43184200	0.06916500	0.01909300
C	-7.07824300	1.09281900	0.93132200
C	-5.80435000	1.61623500	0.93231700
H	3.20653700	1.13851900	4.33096400
H	2.28135800	-1.15051000	4.49999500
H	1.23402500	-2.24786900	2.52500500
H	3.07286900	2.39233800	2.17257800
H	1.17447100	4.60378300	-0.35802300
H	2.94995400	1.72136400	-2.05587700
H	0.60489000	-2.40275200	-1.34605600
H	1.04860200	-3.00330700	0.24524000
H	-0.98445400	-0.62136700	1.46705500
H	-3.36835400	-0.82422200	2.04477400
H	-4.76592800	-2.59154300	1.00626700
H	-3.74096500	-4.17138100	-0.62432400
H	-1.34601800	-3.96329500	-1.21101900
H	5.06445700	3.87188700	-0.82505600
H	3.90317500	5.27118900	-0.43686200
H	4.60602300	0.45660500	-2.47859600
H	5.97202100	0.37237300	-1.35188900
H	3.76392600	-1.69910700	-2.91641900
H	3.16923900	-3.99246000	-2.18430600
H	3.47529700	-4.65554900	0.19321100
H	4.34977500	-3.00096200	1.83318600
H	4.95450200	-0.71613600	1.08727600
H	-0.63699200	1.07717800	-1.56476800
H	-2.14428300	-0.29187600	-2.41227000
H	-4.44593300	-1.17303100	-2.48239600
H	-3.21639900	2.42616100	0.73637200
H	-6.76355300	-1.19466900	-1.58057800
H	-8.43975500	-0.33591700	0.02787200
H	-7.81899600	1.46525200	1.63346800
H	-5.53034500	2.40134400	1.63234500

20-9*S*-2

Zero-point correction = 0.589136 (Hartree/Particle)

Thermal correction to Energy = 0.623678

Thermal correction to Enthalpy = 0.623678

Thermal correction to Gibbs Free Energy = 0.521079

E(elec) = -1801.378211

C	-2.15945900	-1.25829200	-2.27803000
C	-1.62879400	-2.34714600	-1.58486700
C	-1.13972300	-2.21366800	-0.27783900

C	-1.21241400	-0.95577400	0.30956100
C	-1.72664100	0.15410900	-0.38631400
C	-2.20404300	0.00753300	-1.67899700
N	-0.82557600	-0.56700700	1.60071700
C	-1.01541500	0.79221300	1.79333300
C	-1.61768000	1.37667100	0.49565400
C	-0.73471300	2.49842600	-0.05299800
C	-1.42496800	3.64357100	-0.21588000
C	-2.82368500	3.49314600	0.13918600
C	-2.98118500	2.13083900	0.79383300
O	-0.75588000	1.40409900	2.81388900
C	-0.14480900	-1.40955900	2.57797000
C	1.33023900	-1.57719700	2.27742000
C	2.18274600	-0.46728900	2.34703900
C	3.54018200	-0.60531400	2.06759200
C	4.06233600	-1.85297800	1.71924200
C	3.22028000	-2.96242000	1.65254500
C	1.85858900	-2.82276700	1.92823100
C	-3.82218500	4.35839200	-0.07548100
O	-4.12690700	1.48683900	0.29900900
C	-4.51984800	0.31882900	1.02233000
C	-4.94063100	-0.76472900	0.05977900
C	-4.65647500	-2.10356000	0.33639100
C	-5.03941100	-3.10854100	-0.55291600
C	-5.70786600	-2.77981700	-1.73192600
C	-5.99391300	-1.44176500	-2.01429800
C	-5.61510700	-0.44018200	-1.12255700
C	0.72157400	2.38614500	-0.35517500
N	1.13641600	1.10458300	-0.63463000
O	1.45870900	3.36732000	-0.35273800
C	2.42841800	0.63045300	-0.90220500
C	2.51511700	-0.73240000	-1.30428200
C	3.73376100	-1.31322700	-1.53469600
C	4.93716000	-0.57690400	-1.37333100
C	4.84547000	0.79735300	-0.98119500
C	3.57551400	1.38745400	-0.75795800
C	6.21576900	-1.15809900	-1.56725800
C	7.36302200	-0.41848000	-1.38081300
C	7.27545700	0.94169900	-0.99645800
C	6.04750400	1.53451400	-0.80437100
H	-2.54728600	-1.39348800	-3.28193100
H	-1.59555800	-3.32182200	-2.06223600
H	-0.71845500	-3.06128900	0.25045600
H	-2.61637900	0.86108900	-2.20612300
H	-0.98526900	4.55890500	-0.59566500
H	-3.04804100	2.26073800	1.88383800
H	-0.29032700	-0.91462400	3.54183200
H	-0.65011000	-2.38031700	2.61143500
H	1.77514500	0.50235400	2.61757900
H	4.19028500	0.26243200	2.10592300
H	5.11756800	-1.95188500	1.48476200
H	3.61941800	-3.93528100	1.37911000

H	1.20380400	-3.69047600	1.88024500
H	-4.84316400	4.11389100	0.19835700
H	-3.64441800	5.32186100	-0.54403500
H	-3.69941000	-0.04766500	1.65051000
H	-5.34592800	0.59251800	1.69636100
H	-4.11364000	-2.36100700	1.24245400
H	-4.80308300	-4.14532300	-0.32998300
H	-6.00124500	-3.55979400	-2.42899100
H	-6.51088800	-1.17855900	-2.93312200
H	-5.81471400	0.60230600	-1.34805500
H	0.40398600	0.41646500	-0.75108500
H	1.60136100	-1.31328700	-1.40240500
H	3.79149300	-2.35867800	-1.82542900
H	3.50149400	2.42539200	-0.46363100
H	6.27438900	-2.20265400	-1.86430300
H	8.33758000	-0.87465600	-1.52938900
H	8.18507500	1.51819100	-0.85311400
H	5.97801200	2.57813600	-0.50881700

20-9*S*-3

Zero-point correction = 0.589737 (Hartree/Particle)

Thermal correction to Energy = 0.624032

Thermal correction to Enthalpy = 0.624032

Thermal correction to Gibbs Free Energy = 0.522027

E(elec) = -1801.369024

C	-2.75464400	-0.67420100	3.45977500
C	-2.23449600	0.61927800	3.55346100
C	-1.64462800	1.24681300	2.44893800
C	-1.60631400	0.53629400	1.25704400
C	-2.10535600	-0.76803400	1.15410700
C	-2.68159000	-1.38354100	2.25268500
N	-1.11689800	0.95369100	0.00382500
C	-1.29018800	-0.01934600	-0.94635500
C	-1.87799800	-1.26091500	-0.25743500
C	-0.92541500	-2.46849000	-0.27394600
C	-1.61534100	-3.61531200	-0.41836500
C	-3.02546800	-3.35881600	-0.67416800
C	-3.13015700	-1.87133700	-0.97960600
O	-0.96447800	0.06776700	-2.12830300
C	-0.47959500	2.22666300	-0.27589100
C	0.99123300	2.28980200	0.09854200
C	1.78591700	3.28390100	-0.48557300
C	3.13429100	3.40237000	-0.15454100
C	3.70869800	2.52267700	0.76553600
C	2.92378900	1.53122800	1.35030200
C	1.57307100	1.41428900	1.01946800
C	-4.05020400	-4.21727700	-0.64996200
O	-4.39094300	-1.37094700	-0.62646900
C	-4.88046000	-0.29917500	-1.42730200
C	-4.43151500	1.06947700	-0.96907100
C	-3.91061100	1.99324900	-1.87997900
C	-3.57092700	3.28207500	-1.46534200

C	-3.73925600	3.65138700	-0.12974300
C	-4.23850800	2.72485900	0.78952200
C	-4.58505000	1.44212200	0.37139400
C	0.55101500	-2.41033400	-0.02810800
N	1.20266100	-1.52309100	-0.84815100
O	1.09400500	-3.11917400	0.81279100
C	2.55232500	-1.13472400	-0.83027400
C	2.91033300	-0.11381500	-1.75599400
C	4.18724200	0.37924500	-1.78718700
C	5.18094300	-0.10746900	-0.89669300
C	4.81935300	-1.13966100	0.02693200
C	3.49546400	-1.64930900	0.03800000
C	6.50105600	0.40843300	-0.87675700
C	7.43264000	-0.06932600	0.01891900
C	7.07898800	-1.09263200	0.93150200
C	5.80496400	-1.61575000	0.93291200
H	-3.20654500	-1.13864800	4.33064200
H	-2.28205200	1.15066800	4.49949200
H	-1.23499900	2.24816200	2.52441800
H	-3.07239400	-2.39264900	2.17241700
H	-1.17362200	-4.60389200	-0.35743000
H	-2.94912700	-1.72215400	-2.05620800
H	-0.60566100	2.40277300	-1.34663700
H	-1.04954700	3.00338100	0.24461900
H	1.34513600	3.96321900	-1.21228200
H	3.74005500	4.17175500	-0.62559700

H	4.76507900	2.59277100	1.00574000
H	3.36759300	0.82577700	2.04501500
H	0.98374300	0.62251900	1.46736800
H	-5.06362500	-3.87269100	-0.82549500
H	-3.90214600	-5.27176100	-0.43698500
H	-4.60427800	-0.45680700	-2.47945000
H	-5.97135200	-0.37345000	-1.35397800
H	-3.76360600	1.69985700	-2.91602200
H	-3.17007300	3.99300300	-2.18246500
H	-3.47736100	4.65473100	0.19532600
H	-4.35187900	2.99896700	1.83402200
H	-4.95537700	0.71425300	1.08667300
H	0.63755600	-1.07689200	-1.56414400
H	2.14477100	0.29194000	-2.41180600
H	4.44656000	1.17279500	-2.48239200
H	3.21679900	-2.42522900	0.73761100
H	6.76433500	1.19421400	-1.58097500
H	8.44065700	0.33550600	0.02733900
H	7.81978800	-1.46505100	1.63360600
H	5.53093400	-2.40060200	1.63321800

20-9*S*-4

Zero-point correction = 0.589736 (Hartree/Particle)

Thermal correction to Energy = 0.624031

Thermal correction to Enthalpy = 0.624031

Thermal correction to Gibbs Free Energy = 0.522019

E(elec) = -1801.369002

C	2.75420800	0.67370000	3.46019100	C	3.56976400	-3.28174200	-1.46708100
C	2.23361900	-0.61961400	3.55377900	C	3.73738800	-3.65212000	-0.13167300
C	1.64373800	-1.24695400	2.44915600	C	4.23668500	-2.72652500	0.78847800
C	1.60584800	-0.53640100	1.25726300	C	4.58405900	-1.44365000	0.37140600
C	2.10539300	0.76773900	1.15440200	C	-0.55046100	2.41038700	-0.02830000
C	2.68163200	1.38305700	2.25309000	N	-1.20215200	1.52384900	-0.84904300
N	1.11648300	-0.95362700	0.00395000	O	-1.09341900	3.11873000	0.81303700
C	1.29036900	0.01931000	-0.94620600	C	-2.55176600	1.13525400	-0.83110300
C	1.87846900	1.26070100	-0.25715300	C	-2.90963200	0.11400700	-1.75648700
C	0.92604000	2.46844600	-0.27384800	C	-4.18647800	-0.37924500	-1.78751200
C	1.61612600	3.61514200	-0.41835500	C	-5.18023100	0.10759200	-0.89715900
C	3.02626000	3.35842700	-0.67388200	C	-4.81879200	1.14015100	0.02611300
C	3.13083800	1.87087100	-0.97909300	C	-3.49499900	1.65002600	0.03697100
O	0.96492500	-0.06768200	-2.12824200	C	-6.50023500	-0.40857600	-0.87695600
C	0.47869700	-2.22633400	-0.27583300	C	-7.43184500	0.06926000	0.01864200
C	-0.99203800	-2.28917200	0.09896800	C	-7.07834700	1.09294100	0.93086900
C	-1.78677600	-3.28387000	-0.48403900	C	-5.80444100	1.61633500	0.93200400
C	-3.13507700	-3.40210900	-0.15262200	H	3.20611200	1.13798600	4.33114200
C	-3.70933400	-2.52161900	0.76678000	H	2.28085000	-1.15102800	4.49981400
C	-2.92436400	-1.52956600	1.35045300	H	1.23369800	-2.24813900	2.52457200
C	-1.57374200	-1.41282900	1.01919900	H	3.07280000	2.39203000	2.17287000
C	4.05115600	4.21668300	-0.64945500	H	1.17452800	4.60379400	-0.35767500
O	4.39142400	1.37042200	-0.62528400	H	2.95020600	1.72149600	-2.05573500
C	4.88132300	0.29872000	-1.42592200	H	0.60444700	-2.40223100	-1.34666700
C	4.43133400	-1.06997600	-0.96885000	H	1.04851000	-3.00339400	0.24433700
C	3.91030300	-1.99281800	-1.88064400	H	-1.34609700	-3.96385500	-1.21019100

H	-3.74088500	-4.17196500	-0.62285400
H	-4.76566100	-2.59153300	1.00728000
H	-3.36807200	-0.82349800	2.04460600
H	-0.98438500	-0.62056700	1.46619900
H	5.06454000	3.87185900	-0.82470300
H	3.90325500	5.27121700	-0.43664000
H	4.60637100	0.45689800	-2.47832600
H	5.97216900	0.37250800	-1.35139300
H	3.76391000	-1.69858400	-2.91653600
H	3.16886500	-3.99197300	-2.18487000
H	3.47483400	-4.65557400	0.19252300
H	4.34950200	-3.00142400	1.83283000
H	4.95451200	-0.71651500	1.08737400
H	-0.63693200	1.07763700	-1.56495200
H	-2.14403600	-0.29182600	-2.41221500
H	-4.44567300	-1.17306500	-2.48245700
H	-3.21643000	2.42620100	0.73634300
H	-6.76339000	-1.19465300	-1.58089000
H	-8.43976900	-0.33579900	0.02728500
H	-7.81917700	1.46542700	1.63290600
H	-5.53052100	2.40147600	1.63203100

20-9*S*-5

Zero-point correction = 0.589736 (Hartree/Particle)

Thermal correction to Energy = 0.624031

Thermal correction to Enthalpy = 0.624031

Thermal correction to Gibbs Free Energy = 0.522018

E(elec) = -1801.368995

C	2.75425400	0.67327700	3.45997100
C	2.23350900	-0.61997800	3.55344400
C	1.64351400	-1.24713100	2.44877300
C	1.60568000	-0.53645800	1.25695600
C	2.10534000	0.76765100	1.15422200
C	2.68169800	1.38278000	2.25295000
N	1.11631400	-0.95354000	0.00360600
C	1.29028600	0.01948500	-0.94646500
C	1.87840100	1.26079100	-0.25725000
C	0.92599200	2.46856000	-0.27376500
C	1.61607800	3.61525400	-0.41825200
C	3.02619400	3.35855700	-0.67391600
C	3.13073800	1.87102500	-0.97923000
O	0.96487500	-0.06738500	-2.12851000
C	0.47871100	-2.22629600	-0.27634000
C	-0.99205400	-2.28932500	0.09823000
C	-1.57381200	-1.41327600	1.01872100
C	-2.92441900	-1.53018500	1.34993200
C	-3.70931100	-2.52215400	0.76598800
C	-3.13500800	-3.40232600	-0.15367800
C	-1.78671300	-3.28388500	-0.48508900
C	4.05109100	4.21680700	-0.64950100
O	4.39137600	1.37054800	-0.62562300
C	4.88103800	0.29872800	-1.42623200
C	4.43135100	-1.06989900	-0.96865500

C	4.58435500	-1.44312300	0.37168500
C	4.23729500	-2.72594600	0.78921200
C	3.73803800	-3.65191900	-0.13056300
C	3.57015600	-3.28199100	-1.46607600
C	3.91037300	-1.99313400	-1.88009300
C	-0.55046000	2.41042900	-0.02793500
N	-1.20227300	1.52415200	-0.84888000
O	-1.09327600	3.11845400	0.81374800
C	-2.55186600	1.13549600	-0.83083600
C	-2.90982100	0.11453500	-1.75650200
C	-4.18666900	-0.37870700	-1.78756600
C	-5.18033900	0.10784700	-0.89696200
C	-4.81880400	1.14009300	0.02662000
C	-3.49500700	1.64996800	0.03751300
C	-6.50035500	-0.40830200	-0.87683000
C	-7.43188500	0.06926600	0.01899300
C	-7.07829000	1.09263700	0.93153300
C	-5.80437000	1.61599600	0.93275000
H	3.20625300	1.13741900	4.33095000
H	2.28070700	-1.15150100	4.49941900
H	1.23338400	-2.24828800	2.52408900
H	3.07295100	2.39172700	2.17283900
H	1.17451200	4.60391400	-0.35746200
H	2.95001200	1.72172200	-2.05585800
H	0.60467000	-2.40218600	-1.34714800
H	1.04851800	-3.00329400	0.24392500
H	-0.98447200	-0.62111800	1.46594000
H	-3.36818700	-0.82435600	2.04429000
H	-4.76561900	-2.59223900	1.00651600
H	-3.74075700	-4.17209500	-0.62412700
H	-1.34599800	-3.96363200	-1.21144100
H	5.06446600	3.87200500	-0.82486100
H	3.90322700	5.27132800	-0.43658000
H	4.60562400	0.45663900	-2.47855500
H	5.97191100	0.37259300	-1.35218000
H	4.95474400	-0.71569800	1.08738900
H	4.35032000	-3.00047000	1.83364000
H	3.47571500	-4.65532500	0.19397100
H	3.16928900	-3.99253400	-2.18357300
H	3.76377200	-1.69923900	-2.91605300
H	-0.63713700	1.07811500	-1.56495500
H	-2.14428900	-0.29109300	-2.41243100
H	-4.44593200	-1.17230200	-2.48274100
H	-3.21638000	2.42591100	0.73711300
H	-6.76357900	-1.19413800	-1.58100800
H	-8.43982200	-0.33576400	0.02758000
H	-7.81905800	1.46490700	1.63375000
H	-5.53037700	2.40089900	1.63301400

20-9*S*-6

Zero-point correction = 0.589307 (Hartree/Particle)

Thermal correction to Energy = 0.623853

Thermal correction to Enthalpy = 0.623853

Thermal correction to Gibbs Free Energy = 0.52069

E(elec) = -1801.374323

C	0.87546000	0.97902800	3.20703200
C	0.99755400	-0.40934400	3.12276600
C	0.94631400	-1.07424700	1.89107200
C	0.77434400	-0.29941400	0.75010500
C	0.68134300	1.10186100	0.82004600
C	0.71880200	1.74618100	2.04575300
N	0.63296000	-0.71377200	-0.58524400
C	0.47156900	0.36810900	-1.43330900
C	0.51338100	1.64893700	-0.57726300
C	-0.70732100	2.54520400	-0.76083000
C	-0.35176300	3.83159100	-0.95009900
C	1.09351600	3.99403900	-0.95452000
C	1.67991800	2.59910800	-1.07273900
O	0.35569900	0.30314900	-2.64542800
C	0.56699900	-2.09110600	-1.06029700
C	1.92223500	-2.66701800	-1.40763300
C	2.59734300	-2.20703700	-2.54706900
C	3.85568000	-2.71299500	-2.86935600
C	4.45035300	-3.68634600	-2.06039200
C	3.77818300	-4.15315800	-0.93066300
C	2.51948800	-3.64225000	-0.60682400
C	1.80095700	5.12546500	-0.85753600
O	2.89714700	2.49247200	-0.39083300
C	3.69349200	1.35235200	-0.72054300
C	4.04327900	0.53804400	0.50544300
C	4.40173600	-0.80620800	0.36309300
C	4.75829100	-1.56316300	1.47847400
C	4.75984400	-0.98331600	2.74795500
C	4.40497600	0.35871600	2.89241200
C	4.05159300	1.11714500	1.77677500
C	-2.12319700	2.09059600	-0.69332700
N	-2.28723500	0.89566200	-0.02717100
O	-3.03773800	2.73953100	-1.19069900
C	-3.46346900	0.16217900	0.19223000
C	-3.32869700	-0.99635300	1.01062000
C	-4.41133300	-1.79649700	1.27124200
C	-5.69032800	-1.49382600	0.73282100
C	-5.82253400	-0.32771800	-0.08814400
C	-4.69359000	0.49075100	-0.34634600
C	-6.83022800	-2.30021300	0.98056100
C	-8.05480300	-1.97377300	0.44169300
C	-8.18873100	-0.82127900	-0.37050300
C	-7.10019400	-0.01886000	-0.62829600
H	0.91486800	1.46681400	4.17574700
H	1.14060900	-0.99149100	4.02793000
H	1.05969200	-2.15073500	1.83427200
H	0.63364500	2.82737200	2.09491900
H	-1.06325000	4.64266800	-1.05642800
H	1.81289000	2.37176100	-2.14168400
H	0.07140400	-2.69153200	-0.29132400

H	-0.07606000	-2.07231600	-1.94447400
H	2.12585100	-1.45296700	-3.17069800
H	4.37196900	-2.35219000	-3.75444100
H	5.43042800	-4.08050900	-2.31297500
H	4.23206700	-4.91294300	-0.30067900
H	1.99950400	-4.00605800	0.27605000
H	2.88497200	5.10104300	-0.82535600
H	1.31395200	6.09389800	-0.79191800
H	3.18286400	0.71818900	-1.45521700
H	4.61064300	1.72250400	-1.20114300
H	4.39384300	-1.26754700	-0.61871200
H	5.02296800	-2.60856500	1.35043300
H	5.03200900	-1.57351300	3.61888400
H	4.39510900	0.81644900	3.87782300
H	3.75504300	2.15382500	1.88540500
H	-1.48306800	0.56435200	0.48987900
H	-2.35349500	-1.23515300	1.43074400
H	-4.29937600	-2.67732300	1.89834300
H	-4.79203200	1.37572900	-0.95964300
H	-6.71962600	-3.18324400	1.60549200
H	-8.92171900	-2.59828400	0.63745300
H	-9.15876600	-0.57177900	-0.79128800
H	-7.20064100	0.86596600	-1.25121600

20-9*R*-1

Zero-point correction = 0.589736 (Hartree/Particle)

Thermal correction to Energy = 0.624031

Thermal correction to Enthalpy = 0.624031

Thermal correction to Gibbs Free Energy = 0.522022

E(elec) = -1801.369006

C	-2.75426700	0.67421100	3.46010000
C	-2.23383400	-0.61915000	3.55382500
C	-1.64398500	-1.24666700	2.44928200
C	-1.60596600	-0.53624800	1.25731600
C	-2.10532200	0.76795900	1.15433500
C	-2.68153800	1.38344900	2.25293300
N	-1.11668900	-0.95368100	0.00405100
C	-1.29037700	0.01923000	-0.94619500
C	-1.87830800	1.26076800	-0.25725100
C	-0.92579400	2.46841700	-0.27404100
C	-1.61577400	3.61516500	-0.41871000
C	-3.02591600	3.35854500	-0.67431800
C	-3.13058800	1.87097600	-0.97937100
O	-0.96484200	-0.06789700	-2.12818300
C	-0.47914400	-2.22653100	-0.27561800
C	0.99168000	-2.28946100	0.09875700
C	1.57350000	-1.41341500	1.01920800
C	2.92417100	-1.53021800	1.35020400
C	3.70906400	-2.52207600	0.76607900
C	3.13469700	-3.40225300	-0.15354700
C	1.78634200	-3.28392000	-0.48473900
C	-4.05072100	4.21691500	-0.65009200
O	-4.39126200	1.37065100	-0.62571500

C	-4.88105800	0.29886300	-1.42631400
C	-4.43140500	-1.06980400	-0.96880500
C	-3.91059100	-1.99305200	-1.88031400
C	-3.57039900	-3.28194500	-1.46636100
C	-3.73817300	-3.65189400	-0.13085100
C	-4.23727800	-2.72590100	0.78900400
C	-4.58429300	-1.44305100	0.37155200
C	0.55067100	2.41031300	-0.02829300
N	1.20237600	1.52354200	-0.84879500
O	1.09361100	3.11880000	0.81292200
C	2.55200300	1.13502400	-0.83081200
C	2.90998900	0.11397700	-1.75638000
C	4.18686700	-0.37918200	-1.78744100
C	5.18055400	0.10756600	-0.89695500
C	4.81899300	1.13990600	0.02650900
C	3.49514800	1.64966400	0.03743000
C	6.50061900	-0.40845900	-0.87685000
C	7.43217100	0.06931900	0.01884000
C	7.07855000	1.09278500	0.93126200
C	5.80458200	1.61602900	0.93250000
H	-3.20615700	1.13863300	4.33098600
H	-2.28116500	-1.15047600	4.49990400
H	-1.23408300	-2.24790100	2.52482000
H	-3.07255500	2.39247100	2.17260800
H	-1.17411300	4.60378900	-0.35807600
H	-2.94980000	1.72149200	-2.05596900
H	-0.60526800	-2.40271800	-1.34635500
H	-1.04889000	-3.00337500	0.24494100
H	0.98416600	-0.62133400	1.46656500
H	3.36797600	-0.82438700	2.04453700
H	4.76541600	-2.59208500	1.00643800
H	3.74044900	-4.17193700	-0.62412900
H	1.34557500	-3.96365700	-1.21106700
H	-5.06413800	3.87220200	-0.82537800
H	-3.90273100	5.27145900	-0.43737100
H	-4.60573700	0.45680400	-2.47865300
H	-5.97191800	0.37280400	-1.35215700
H	-3.76405200	-1.69914700	-2.91627800
H	-3.16964500	-3.99248500	-2.18392500
H	-3.47590700	-4.65532600	0.19364700
H	-4.35020500	-3.00046100	1.83343300
H	-4.95455300	-0.71560400	1.08729800
H	0.63722000	1.07733700	-1.56475100
H	2.14445100	-0.29177700	-2.41222400
H	4.44616200	-1.17283300	-2.48254100
H	3.21651000	2.42568600	0.73694000
H	6.76386800	-1.19436500	-1.58094100
H	8.44014700	-0.33561500	0.02740700
H	7.81933900	1.46522100	1.63336900
H	5.53056700	2.40100600	1.63267200

20-9*R*-2

Zero-point correction = 0.589135 (Hartree/Particle)

Thermal correction to Energy = 0.623677

Thermal correction to Enthalpy = 0.623677

Thermal correction to Gibbs Free Energy = 0.521076

E(elec) = -1801.378202

C	2.15985900	-1.25701400	-2.27893500
C	1.62936900	-2.34624200	-1.58623100
C	1.14022500	-2.21339700	-0.27916500
C	1.21265600	-0.95572100	0.30872400
C	1.72667600	0.15455000	-0.38670400
C	2.20418300	0.00858400	-1.67941200
N	0.82574200	-0.56751900	1.60005500
C	1.01534400	0.79162000	1.79319300
C	1.61755900	1.37670800	0.49585600
C	0.73455900	2.49858200	-0.05253900
C	1.42485800	3.64368100	-0.21548100
C	2.82359600	3.49314700	0.13948000
C	2.98098400	2.13091600	0.79428100
O	0.75578700	1.40304900	2.81405500
C	0.14531300	-1.41068300	2.57704400
C	-1.32973600	-1.57851300	2.27661700
C	-2.18249100	-0.46883500	2.34674900
C	-3.53990300	-0.60708500	2.06732600
C	-4.06177800	-1.85473300	1.71848200
C	-3.21947200	-2.96394700	1.65130400
C	-1.85779400	-2.82407300	1.92697300
C	3.82211900	4.35837600	-0.07517600
O	4.12683400	1.48695500	0.29971700
C	4.51942900	0.31871700	1.02283500
C	4.94041800	-0.76460200	0.06010800
C	4.65618900	-2.10349800	0.33633400
C	5.03927000	-3.10825900	-0.55315600
C	5.70796400	-2.77925300	-1.73195100
C	5.99410200	-1.44113900	-2.01392200
C	5.61514000	-0.43977000	-1.12200500
C	-0.72184000	2.38635900	-0.35424400
N	-1.13668100	1.10490100	-0.63419500
O	-1.45901800	3.36747500	-0.35105300
C	-2.42871600	0.63085900	-0.90181800
C	-2.51546100	-0.73182200	-1.30442400
C	-3.73415100	-1.31253000	-1.53493500
C	-4.93751300	-0.57624900	-1.37318000
C	-4.84577300	0.79786100	-0.98049200
C	-3.57579300	1.38782800	-0.75711700
C	-6.21614200	-1.15733400	-1.56725600
C	-7.36337100	-0.41776600	-1.38043200
C	-7.27575800	0.94224600	-0.99553700
C	-6.04777900	1.53496300	-0.80329300
H	2.54778000	-1.39176300	-3.28286300
H	1.59634300	-3.32073300	-2.06399900
H	0.71906900	-3.06131300	0.24874600
H	2.61638200	0.86243400	-2.20617400
H	0.98523600	4.55906600	-0.59522400

H	3.04757800	2.26085600	1.88431300
H	0.29088200	-0.91616700	3.54112800
H	0.65084200	-2.38135200	2.60990700
H	-1.77510300	0.50079600	2.61767000
H	-4.19023100	0.26047700	2.10606700
H	-5.11700100	-1.95376600	1.48399700
H	-3.61837600	-3.93680300	1.37750700
H	-1.20281500	-3.69161400	1.87861800
H	4.84306400	4.11390200	0.19878000
H	3.64434700	5.32177500	-0.54385000
H	3.69877100	-0.04786700	1.65068600
H	5.34532600	0.59215800	1.69721000
H	4.11317500	-2.36117300	1.24222700
H	4.80286900	-4.14509200	-0.33053400
H	6.00145400	-3.55906000	-2.42916000
H	6.51127500	-1.17770900	-2.93257100
H	5.81481500	0.60277100	-1.34720400
H	-0.40423700	0.41686800	-0.75113100
H	-1.60173400	-1.31270200	-1.40284900
H	-3.79194000	-2.35786700	-1.82607500
H	-3.50168900	2.42562600	-0.46229500
H	-6.27482700	-2.20176100	-1.86474200
H	-8.33793800	-0.87388300	-1.52913300
H	-8.18534700	1.51870900	-0.85189100
H	-5.97826700	2.57847300	-0.50733500

20-9*R*-3

Zero-point correction = 0.589736 (Hartree/Particle)

Thermal correction to Energy = 0.624031

Thermal correction to Enthalpy = 0.624031

Thermal correction to Gibbs Free Energy = 0.522024

E(elec) = -1801.369006

C	-2.75471200	0.67401200	3.45992600
C	-2.23426200	-0.61934300	3.55365100
C	-1.64425800	-1.24678900	2.44915000
C	-1.60610300	-0.53630900	1.25722400
C	-2.10548100	0.76788700	1.15424400
C	-2.68184200	1.38331200	2.25280400
N	-1.11663700	-0.95365700	0.00400400
C	-1.29022500	0.01929400	-0.94621800
C	-1.87829500	1.26077500	-0.25728200
C	-0.92585200	2.46848600	-0.27385300
C	-1.61589700	3.61519900	-0.41845600
C	-3.02599100	3.35850400	-0.67428700
C	-3.13048900	1.87096500	-0.97956600
O	-0.96451900	-0.06775600	-2.12816300
C	-0.47899400	-2.22646400	-0.27564400
C	0.99184200	-2.28925900	0.09872500
C	1.78666500	-3.28347700	-0.48495800
C	3.13502800	-3.40169700	-0.15374100
C	3.70924700	-2.52163100	0.76607900
C	2.92420000	-1.52999700	1.35038100

C	1.57352100	-1.41331800	1.01937200	H	-3.07286800	2.39233000	2.17247800
C	-4.05087700	4.21677500	-0.65005500	H	-1.17430900	4.60384900	-0.35769100
O	-4.39119800	1.37046900	-0.62627300	H	-2.94944600	1.72163700	-2.05614000
C	-4.88062100	0.29859800	-1.42699100	H	-0.60511400	-2.40271100	-1.34637000
C	-4.43115200	-1.07000600	-0.96911500	H	-1.04869200	-3.00332000	0.24495100
C	-3.91030500	-1.99349600	-1.88036300	H	1.34601800	-3.96311400	-1.21145100
C	-3.57025700	-3.28232000	-1.46608300	H	3.74090600	-4.17118800	-0.62447400
C	-3.73818600	-3.65195200	-0.13050300	H	4.76560600	-2.59153200	1.00644100
C	-4.23733500	-2.72572100	0.78908500	H	3.36789500	-0.82423900	2.04485700
C	-4.58422700	-1.44294200	0.37130400	H	0.98406400	-0.62140500	1.46686200
C	0.55060600	2.41040400	-0.02806200	H	-5.06423800	3.87199600	-0.82554000
N	1.20233900	1.52371900	-0.84863900	H	-3.90301400	5.27130200	-0.43715400
O	1.09349700	3.11880800	0.81325400	H	-4.60483400	0.45641700	-2.47922500
C	2.55196300	1.13518100	-0.83068200	H	-5.97151800	0.37250900	-1.35333800
C	2.90998200	0.11440400	-1.75653300	H	-3.76362400	-1.69982700	-2.91637400
C	4.18685100	-0.37877000	-1.78767700	H	-3.16947500	-3.99305200	-2.18344200
C	5.18049300	0.10767400	-0.89697800	H	-3.47600500	-4.65532400	0.19425000
C	4.81890300	1.13974500	0.02677400	H	-4.35039000	-3.00003200	1.83356600
C	3.49507500	1.64954500	0.03776000	H	-4.95451400	-0.71530700	1.08684300
C	6.50053500	-0.40841300	-0.87692900	H	0.63721500	1.07763900	-1.56469500
C	7.43203900	0.06905600	0.01897600	H	2.14447700	-0.29113400	-2.41254800
C	7.07839000	1.09226100	0.93168200	H	4.44616100	-1.17222500	-2.48299500
C	5.80444400	1.61555600	0.93298200	H	3.21642400	2.42537600	0.73747400
H	-3.20672200	1.13838000	4.33077700	H	6.76380400	-1.19411800	-1.58123600
H	-2.28169900	-1.15072000	4.49969700	H	8.43999600	-0.33592400	0.02750000
H	-1.23434100	-2.24801600	2.52469300	H	7.81914200	1.46445500	1.63395600

H	5.53040600	2.40033000	1.63337300

20-9*R*-4

Zero-point correction = 0.589736 (Hartree/Particle)

Thermal correction to Energy = 0.624031

Thermal correction to Enthalpy = 0.624031

Thermal correction to Gibbs Free Energy = 0.522019

E(elec) = -1801.368997

C	-2.75414600	0.67358300	3.46016400
C	-2.23353800	-0.61972100	3.55369200
C	-1.64362800	-1.24700100	2.44904600
C	-1.60571600	-0.53640200	1.25718800
C	-2.10524000	0.76775200	1.15440500
C	-2.68152800	1.38300600	2.25309700
N	-1.11644000	-0.95361900	0.00385200
C	-1.29031100	0.01939100	-0.94627000
C	-1.87838300	1.26076300	-0.25713100
C	-0.92599500	2.46851500	-0.27386100
C	-1.61607000	3.61520000	-0.41853100
C	-3.02620000	3.35847800	-0.67412700
C	-3.13079100	1.87088700	-0.97912400
O	-0.96482800	-0.06753900	-2.12828200
C	-0.47870800	-2.22633500	-0.27596600
C	0.99202300	-2.28922800	0.09876600
C	1.78680800	-3.28377100	-0.48442900
C	3.13508800	-3.40207400	-0.15291900
C	3.70925600	-2.52177400	0.76671500
C	2.92424100	-1.52983700	1.35053600
C	1.57363800	-1.41307500	1.01923600
C	-4.05105500	4.21678600	-0.64991400
O	-4.39141800	1.37048800	-0.62536000
C	-4.88130500	0.29876700	-1.42600000
C	-4.43141100	-1.06992400	-0.96879300
C	-3.91069100	-1.99298600	-1.88054300
C	-3.57028400	-3.28190800	-1.46684800
C	-3.73774000	-3.65206900	-0.13135800
C	-4.23674700	-2.72625900	0.78873700
C	-4.58397400	-1.44338200	0.37154600
C	0.55048800	2.41047200	-0.02814900
N	1.20221300	1.52397600	-0.84893400
O	1.09339700	3.11868600	0.81331300
C	2.55180000	1.13531100	-0.83093300
C	2.90963600	0.11411200	-1.75639300
C	4.18646400	-0.37917300	-1.78746900
C	5.18024300	0.10758100	-0.89708600
C	4.81882900	1.14006200	0.02627600
C	3.49503500	1.64996500	0.03719400
C	6.50025600	-0.40858300	-0.87698200
C	7.43189700	0.06920500	0.01861100
C	7.07842200	1.09281400	0.93093100
C	5.80450600	1.61618700	0.93217000
H	-3.20608500	1.13781900	4.33112300

H	-2.28078400	-1.15119100	4.49969500
H	-1.23360000	-2.24819600	2.52442400
H	-3.07268600	2.39198300	2.17292200
H	-1.17450100	4.60386800	-0.35793200
H	-2.95009700	1.72140100	-2.05572900
H	-0.60454500	-2.40227900	-1.34677100
H	-1.04850800	-3.00335500	0.24426000
H	1.34619300	-3.96361300	-1.21075000
H	3.74094000	-4.17182500	-0.62325900
H	4.76555400	-2.59175900	1.00731600
H	3.36788900	-0.82392400	2.04488600
H	0.98420200	-0.62094900	1.46637900
H	-5.06445700	3.87201800	-0.82519500
H	-3.90313700	5.27134400	-0.43718800
H	-4.60626500	0.45688800	-2.47838200
H	-5.97215000	0.37258300	-1.35155300
H	-3.76438800	-1.69891100	-2.91649100
H	-3.16960800	-3.99230300	-2.18459900
H	-3.47531400	-4.65552500	0.19293900
H	-4.34942400	-3.00098000	1.83315100
H	-4.95415600	-0.71607900	1.08747700
H	0.63701100	1.07782000	-1.56487600
H	2.14402100	-0.29164200	-2.41214500
H	4.44564200	-1.17294100	-2.48247600
H	3.21650200	2.42606700	0.73665000
H	6.76338700	-1.19460300	-1.58098800
H	8.43983100	-0.33583300	0.02718400
H	7.81928200	1.46525400	1.63296100
H	5.53059900	2.40126700	1.63226700

20-9*R*-5

Zero-point correction = 0.589738 (Hartree/Particle)

Thermal correction to Energy = 0.624032

Thermal correction to Enthalpy = 0.624032

Thermal correction to Gibbs Free Energy = 0.522031

E(elec) = -1801.368997

C	-2.75435700	0.67333600	3.46014300
C	-2.23361300	-0.61991600	3.55364500
C	-1.64361300	-1.24709800	2.44899200
C	-1.60576600	-0.53645700	1.25715400
C	-2.10544100	0.76764300	1.15439200
C	-2.68180300	1.38280200	2.25310000
N	-1.11636800	-0.95355400	0.00381800
C	-1.29032700	0.01945700	-0.94627300
C	-1.87856600	1.26073100	-0.25711400
C	-0.92627900	2.46857300	-0.27371300
C	-1.61647000	3.61520400	-0.41822700
C	-3.02657100	3.35835300	-0.67385100
C	-3.13095800	1.87080600	-0.97913000
O	-0.96491300	-0.06743600	-2.12831300
C	-0.47867000	-2.22627000	-0.27609600
C	0.99214100	-2.28914900	0.09836400

C	1.57392000	-1.41305400	1.01879700
C	2.92459600	-1.52978900	1.34980100
C	3.70954400	-2.52161200	0.76569200
C	3.13522100	-3.40183700	-0.15391100
C	1.78685700	-3.28358100	-0.48510300
C	-4.05154600	4.21651300	-0.64947200
O	-4.39153100	1.37017000	-0.62552700
C	-4.88105600	0.29831000	-1.42618400
C	-4.43092200	-1.07025700	-0.96884000
C	-4.58338200	-1.44360600	0.37153400
C	-4.23584800	-2.72636000	0.78886100
C	-3.73666300	-3.65215500	-0.13114300
C	-3.56932800	-3.28210400	-1.46668300
C	-3.91001800	-1.99330400	-1.88050600
C	0.55020600	2.41055300	-0.02802300
N	1.20196500	1.52413400	-0.84885800
O	1.09309100	3.11871800	0.81349700
C	2.55156200	1.13549700	-0.83081900
C	2.90948500	0.11431600	-1.75626300
C	4.18632700	-0.37894200	-1.78724600
C	5.18002700	0.10782500	-0.89678800
C	4.81852600	1.14029300	0.02655600
C	3.49472700	1.65016900	0.03737700
C	6.50004000	-0.40832800	-0.87657000
C	7.43159700	0.06944500	0.01911700
C	7.07803500	1.09303500	0.93142300
C	5.80411900	1.61640600	0.93254800
H	-3.20636200	1.13749800	4.33110800
H	-2.28081200	-1.15141500	4.49963400
H	-1.23346900	-2.24824600	2.52434700
H	-3.07307900	2.39173800	2.17294900
H	-1.17500000	4.60391000	-0.35750500
H	-2.95016700	1.72151500	-2.05575100
H	-0.60467900	-2.40220100	-1.34688900
H	-1.04838900	-3.00330100	0.24421800
H	0.98455600	-0.62097900	1.46612100
H	3.36836900	-0.82391500	2.04410900
H	4.76590500	-2.59154500	1.00603600
H	3.74101500	-4.17149200	-0.62448900
H	1.34613000	-3.96335100	-1.21142500
H	-5.06489400	3.87162000	-0.82480700
H	-3.90377000	5.27105500	-0.43658900
H	-4.60586500	0.45641900	-2.47853200
H	-5.97193400	0.37189600	-1.35194100
H	-4.95370900	-0.71631100	1.08739900
H	-4.34843700	-3.00098600	1.83330900
H	-3.47398500	-4.65551400	0.19325000
H	-3.16851800	-3.99249500	-2.18436300
H	-3.76381300	-1.69930600	-2.91649100
H	0.63678200	1.07795500	-1.56480700
H	2.14392600	-0.29146200	-2.41206800
H	4.44556700	-1.17270700	-2.48223500

H	3.21611900	2.42626300	0.73681900
H	6.76323800	-1.19433800	-1.58056200
H	8.43953100	-0.33559100	0.02777400
H	7.81882600	1.46546600	1.63353000
H	5.53014700	2.40147300	1.63263500

20-9*R*-6

Zero-point correction = 0.589307 (Hartree/Particle)

Thermal correction to Energy = 0.623853

Thermal correction to Enthalpy = 0.623853

Thermal correction to Gibbs Free Energy = 0.52069

E(elec) = -1801.374323

C	-0.87546000	0.97902800	3.20703200
C	-0.99755400	-0.40934400	3.12276600
C	-0.94631400	-1.07424700	1.89107200
C	-0.77434400	-0.29941400	0.75010500
C	-0.68134300	1.10186100	0.82004600
C	-0.71880200	1.74618100	2.04575300
N	-0.63296000	-0.71377200	-0.58524400
C	-0.47156900	0.36810900	-1.43330900
C	-0.51338100	1.64893700	-0.57726300
C	0.70732100	2.54520400	-0.76083000
C	0.35176300	3.83159100	-0.95009900
C	-1.09351600	3.99403900	-0.95452000
C	-1.67991800	2.59910800	-1.07273900
O	-0.35569900	0.30314900	-2.64542800
C	-0.56699900	-2.09110600	-1.06029700
C	-1.92223500	-2.66701800	-1.40763300
C	-2.59734300	-2.20703700	-2.54706900
C	-3.85568000	-2.71299500	-2.86935600
C	-4.45035300	-3.68634600	-2.06039200
C	-3.77818300	-4.15315800	-0.93066300
C	-2.51948800	-3.64225000	-0.60682400
C	-1.80095700	5.12546500	-0.85753600
O	-2.89714700	2.49247200	-0.39083300
C	-3.69349200	1.35235200	-0.72054300
C	-4.04327900	0.53804400	0.50544300
C	-4.40173600	-0.80620800	0.36309300
C	-4.75829100	-1.56316300	1.47847400
C	-4.75984400	-0.98331600	2.74795500
C	-4.40497600	0.35871600	2.89241200
C	-4.05159300	1.11714500	1.77677500
C	2.12319700	2.09059600	-0.69332700
N	2.28723500	0.89566200	-0.02717100
O	3.03773800	2.73953100	-1.19069900
C	3.46346900	0.16217900	0.19223000
C	3.32869700	-0.99635300	1.01062000
C	4.41133300	-1.79649700	1.27124200
C	5.69032800	-1.49382600	0.73282100
C	5.82253400	-0.32771800	-0.08814400
C	4.69359000	0.49075100	-0.34634600
C	6.83022800	-2.30021300	0.98056100

C	8.05480300	-1.97377300	0.44169300	H	-1.31395200	6.09389800	-0.79191800
C	8.18873100	-0.82127900	-0.37050300	H	-3.18286400	0.71818900	-1.45521700
C	7.10019400	-0.01886000	-0.62829600	H	-4.61064300	1.72250400	-1.20114300
H	-0.91486800	1.46681400	4.17574700	H	-4.39384300	-1.26754700	-0.61871200
H	-1.14060900	-0.99149100	4.02793000	H	-5.02296800	-2.60856500	1.35043300
H	-1.05969200	-2.15073500	1.83427200	H	-5.03200900	-1.57351300	3.61888400
H	-0.63364500	2.82737200	2.09491900	H	-4.39510900	0.81644900	3.87782300
H	1.06325000	4.64266800	-1.05642800	H	-3.75504300	2.15382500	1.88540500
H	-1.81289000	2.37176100	-2.14168400	H	1.48306800	0.56435200	0.48987900
H	-0.07140400	-2.69153200	-0.29132400	H	2.35349500	-1.23515300	1.43074400
H	0.07606000	-2.07231600	-1.94447400	H	4.29937600	-2.67732300	1.89834300
H	-2.12585100	-1.45296700	-3.17069800	H	4.79203200	1.37572900	-0.95964300
H	-4.37196900	-2.35219000	-3.75444100	H	6.71962600	-3.18324400	1.60549200
H	-5.43042800	-4.08050900	-2.31297500	H	8.92171900	-2.59828400	0.63745300
H	-4.23206700	-4.91294300	-0.30067900	H	9.15876600	-0.57177900	-0.79128800
H	-1.99950400	-4.00605800	0.27605000	H	7.20064100	0.86596600	-1.25121600
H	-2.88497200	5.10104300	-0.82535600				
